# Substituent-rebound skeletal editing for precise boron-to-carbon single-atom swapping

**DOI:** 10.1038/s41467-026-75992-9

**Published:** 2026-07-25

**Authors:** Yan-Bo Li, Fu-Peng Wu, Jasper L. Tyler, Constantin G. Daniliuc, Frank Glorius

**Affiliations:** Organisch-Chemisches Institut, Universität Münster, Münster, Germany

**Keywords:** Photocatalysis, Synthetic chemistry methodology, Reaction mechanisms

## Abstract

Developing methods that enable single-atom exchange within an aromatic scaffold, while preserving its peripheral substitution, represents an important but highly ambitious goal in synthesis. In principle, such approaches would allow the impact of a single-atom change within a molecular framework to be distinguished from the effects of also altering the peripheral substituents. Yet despite this conceptual power, single-step methodologies for single-atom exchange in aromatic systems remain rare. Herein, we present a boron-to-carbon swapping reaction via a substituent-rebound process, converting 1,2-benzazaborines into the corresponding quinolines. The employment of glyoxylic acid as the carbon-atom source allows the original substituent on the boron atom to be recaptured and incorporated into the quinoline product, representing a rare example of true single-atom skeletal editing. The transformation exhibits high levels of functional group tolerance and is applicable to the late-stage modifications of natural product and pharmaceutical derivatives. Furthermore, comprehensive mechanistic investigations elucidate the intricacies of this process, establishing a foundation for future single-atom editing manifolds that can facilitate the interrogation of structure–function relationships with atom-level precision.

## Abstract (web summary)

Developing methods that enable single-atom exchange within an aromatic scaffold, while preserving its peripheral substitution, represents an important but highly ambitious goal in synthesis. Here the authors present a boron-to-carbon swapping reaction via a substituent-rebound process, converting 1,2-benzazaborines into the corresponding quinolines.

## Introduction

**Fig. 1:**
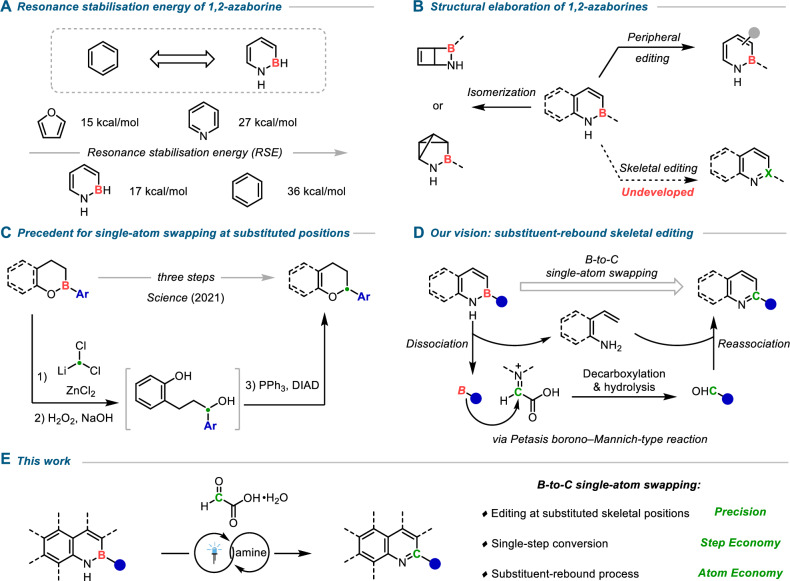
Substituent-rebound skeletal editing for B-to-C single-atom swapping. **A** Resonance stabilisation of 1,2-azaborine. **B** State of the art in 1,2-azaborine applications. **C** Three-step substituent-retaining single-atom swapping. **D** Our design for substituent-rebound skeletal editing. **E** This work: precise boron-to-carbon swapping via substituent-rebound process.

Bioisosteric replacement serves as an important strategy in drug discovery, enabling medicinal chemists to optimise the physicochemical properties, and increase the structural diversity, of lead compounds^1,2^. One such approach is BN/CC isosterism, which involves the substitution of a C=C unit with an isoelectronic and isosteric B–N unit (Fig. 1A). Among BN-heteroarenes, 1,2-azaborines have emerged as the most prominent benzene isosteres^3–7^. Their high resonance stabilisation energy (RSE, 17 kcal mol^−1^) confers exceptional thermal and hydrolytic robustness, making them suitable for physiological environments. From a pharmacological perspective, the N–H motif functions as a unique hydrogen-bond donor, often augmenting protein–ligand binding affinities in ways unattainable for their carbocyclic precursors^8–10^. Despite these findings, direct comparisons between 1,2-azaborines and alternative heteroaromatic scaffolds, such as CN-containing arenes, are rarely reported.

The aromatic stability of 1,2-azaborines presents a synthetic paradox: while it underpins their utility, it simultaneously renders the framework chemically inert towards skeletal diversification. Despite extensive studies on isomerisation^11–13^ and peripheral modifications, including substitution at boron or nitrogen, C–H functionalisation, and hydrogenation^6,14–36^, methods for directly modifying the 1,2-azaborine framework remain scarce (Fig. 1B). That being said, the lower resonance stabilisation energy compared to other 6-membered ring arenes, combined with the unique reactivity of the ring heteroatoms, provide opportunities for the skeletal editing of this framework.

Recently, atom swapping reactions have emerged as a powerful paradigm in skeletal editing, enabling the precise replacement of an individual atom in a molecule’s skeletal framework^37–50^. These transformations provide a ground-breaking avenue for synthetic strategies, particularly in facilitating systematic structure-activity relationship (SAR) investigations^51^. However, most current strategies are restricted to either the exchange of unsubstituted positions or the simultaneous removal of the substituent or functional handle appended to the atom in question. On the other hand, true single-atom swapping, where the substituent appended to the atom undergoing transmutation is fully retained in the final structure, provides the potential to disentangle the molecular property effects of changing an individual atom in the compound’s framework from the influence of simultaneously removing the attached substituent. Due to the practical complexity of precisely executing cleavage of the key atom from the ring system, transfer of the substituent to the new atom, and subsequent ring reassembly in the same reaction manifold, such transformations remain formidable and typically necessitate multi-step sequences (Fig. 1C)^52^.

Inspired by the conceptual framework of rebound mechanisms in which site-specific bond scission is followed by the controlled recombination of the fragments^53–56^, we envisioned a strategic disassembly of the 1,2-benzazaborine skeleton, harnessing its unique reactivity profile. Specifically, we targeted acid-promoted heterocycle cleavage to give 2-vinylaniline and a boron-based intermediate capable of transferring the boron substituent to a carbon-centred electrophile, such as an aldehyde^57^, via a Petasis borono–Mannich-type reaction^58^. This could then recombine with the 2-vinylaniline through a sequence of condensation, cyclisation, and oxidative rearomatisation (Fig. 1D).

Herein, we report a direct B-to-C single-atom edit of 1,2-benzazaborines to access the corresponding quinolines, utilising glyoxylic acid as the carbon-atom source (Fig. 1E). This transformation represents a rare example of substituent-rebound skeletal editing, where the original boron substituent is fully retained in the quinoline architecture. By consolidating this ‘rebound’ logic into a single operation protocol, the method achieves exceptional step and atom economy^59,60^, providing a streamlined and waste-minimised route for skeletal modification. The synthetic utility of this protocol is further underscored by its broad functional group tolerance and its successful application to the late-stage modification of complex drug candidates and natural products.

## Results and discussion

**Fig. 2:**
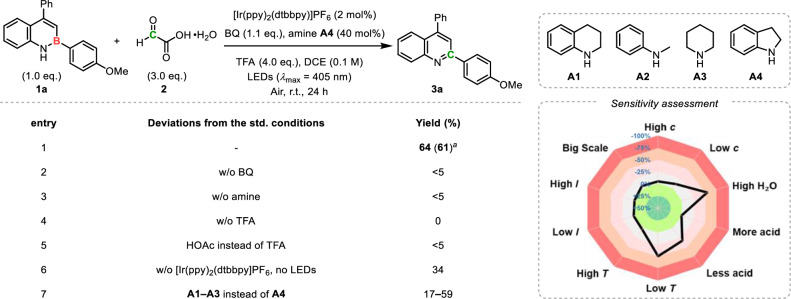
Reaction development and sensitivity assessment. The yield was determined by ^1^H NMR analysis using mesitylene as an internal standard. ^*a*^Isolated yield. BQ benzoquinone, TFA trifluoroacetic acid, DCE 1,2-dichloroethane.

Our investigation began with the reaction of 1,2-benzazaborine **1** and glyoxylic acid monohydrate (**2**) in the presence of trifluoroacetic acid (TFA), to facilitate the dissociation of the heterocycle, and an amine catalyst to promote the Petasis borono–Mannich-type transfer of the boron substituent to the aldehyde (Fig. 2)^57^. To satisfy the oxidative requirements of the proposed transformation, benzoquinone (BQ) was also employed. Additionally, a photocatalyst was introduced to facilitate the subsequent cyclisation reaction^61^. To our delight, the target substituent-rebound quinoline **3a** was obtained in promising yield (Entry 1, 64%). Control experiments revealed that the absence of BQ, amine, and TFA completely inhibited the reaction (Entries 2–4). Substitution of TFA with a weaker acid, such as acetic acid (HOAc), also suppressed product formation (Entry 5). Further control experiments revealed that while the reaction could proceed without irradiation and a photocatalyst, the yield was significantly lower, suggesting the presence of a background acid-promoted pathway (Entry 6). Finally, a screen of various amine catalysts confirmed that indoline (**A4**) remained the most effective (Entry 7). When investigating the sensitivity of the protocol towards perturbations in different conditions^62^, it was observed that the yield was reduced upon an increase in solvent moisture content and lower reaction temperature, whereas changes to concentration, scale, and light intensity had only a minimal effect.

**Fig. 3:**
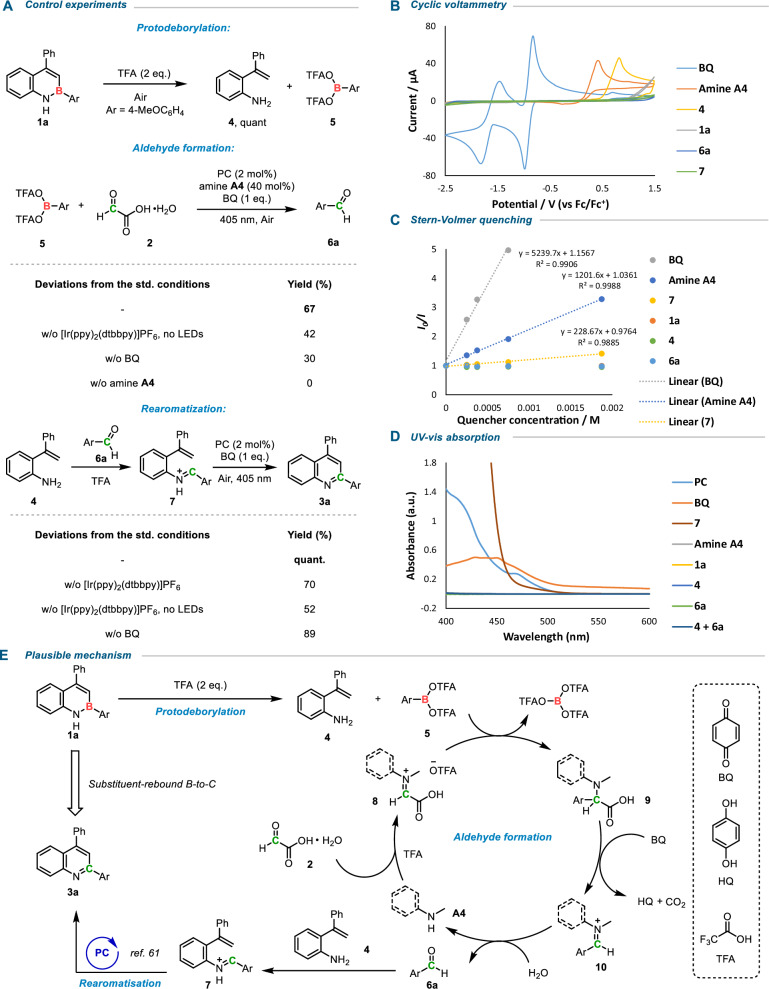
Mechanistic studies and plausible mechanisms. **A** Control experiments. The yield was determined by ^1^H NMR analysis using mesitylene as an internal standard. **B** Cyclic voltammetry measurement. Conditions: 0.1 M TBAPF_6_ in MeCN; scan rate: 0.1 V s^−1^. **C** Stern-Volmer quenching experiments. **D** UV–Vis absorption measurement. **E** Plausible mechanism.

To elucidate the mechanism of this transformation, a series of control experiments was conducted (Fig. 3A). Treatment of 1,2-benzazaborine **1** with TFA afforded 2-vinylaniline **4** in quantitative yield, together with boron species **5**, indicating an initial acid-promoted dissociation process. We next examined the aldehyde-formation step. In the presence of the photocatalyst, indoline (**A4**), and BQ under irradiation, the desired aldehyde was obtained from boronic ester **5** and glyoxylic acid (**2**) in 67% yield after 4 h. In contrast, only 42% yield was observed in the absence of the photocatalyst and LED irradiation over the same period. The aldehyde could also be generated without BQ with a diminished yield of 30%, demonstrating that multiple oxidative pathways can operate in this Petasis borono–Mannich reaction. No product could be observed in the absence of the amine catalyst. Subsequent investigation of the rearomatisation step revealed that the desired quinoline product could be formed quantitatively within 4 h upon mixing 2-vinylaniline **4**, aldehyde **6a**, and TFA in the presence of the photocatalyst, light, and oxidant. Again, product formation was observed upon irradiation in the absence of a photocatalyst, indicating the existence of a light-induced pathway to product formation as well as via a photocatalytic mechanism. Additionally, while BQ was found not to be strictly necessary in this step of the transformation, relying solely on aerobic oxidation resulted in a lower yield.

Cyclic voltammetry (CV) measurements indicated that BQ (*E* = − 0.54 V vs. SCE, CH_3_CN) and indoline (**A4**) (*E* = 0.63 V vs. SCE, CH_3_CN) are thermodynamically capable of undergoing single-electron transfer with the photocatalyst in its excited state (*Ir*^(III)*/(II)^_*1/2*_ = 0.66 V; *Ir*^(III)*/(IV)^_*1/2*_ = − 0.96 V vs. SCE, CH_3_CN)^63^ (Fig. 3B). Stern–Volmer luminescence quenching studies revealed that BQ, indoline (**A4**), and iminium ion **7** efficiently quench the excited state of [Ir(ppy)_2_(dtbbpy)]PF_6_, with BQ showing the strongest quenching effect, suggesting a more favourable interaction with the excited photocatalyst (Fig. 3C). Finally, UV–vis absorption spectroscopy indicated that direct photon absorption by the photocatalyst, BQ, and iminium ion **7** are all feasible under the reaction conditions (Fig. 3D). Based on these experimental results, a plausible mechanism is proposed (Fig. 3E). Following acid-promoted dissociation to generate 2-vinylaniline **4** and boronic ester **5**, a Petasis borono–Mannich-type reaction occurs to form intermediate **9**. This intermediate subsequently undergoes oxidative decarboxylation, followed by hydrolysis, to furnish the corresponding aldehyde **6a**. Finally, condensation of the aldehyde with 2-vinylaniline **4** is followed by cyclisation and subsequent oxidation. Consistent with the observations of Sherborne and Fallan^61^, this can occur via light-promoted 6-π electrocyclization or a photocatalytic single-electron transfer (SET) process, followed by oxidation to afford product **3a**.

**Fig. 4:**
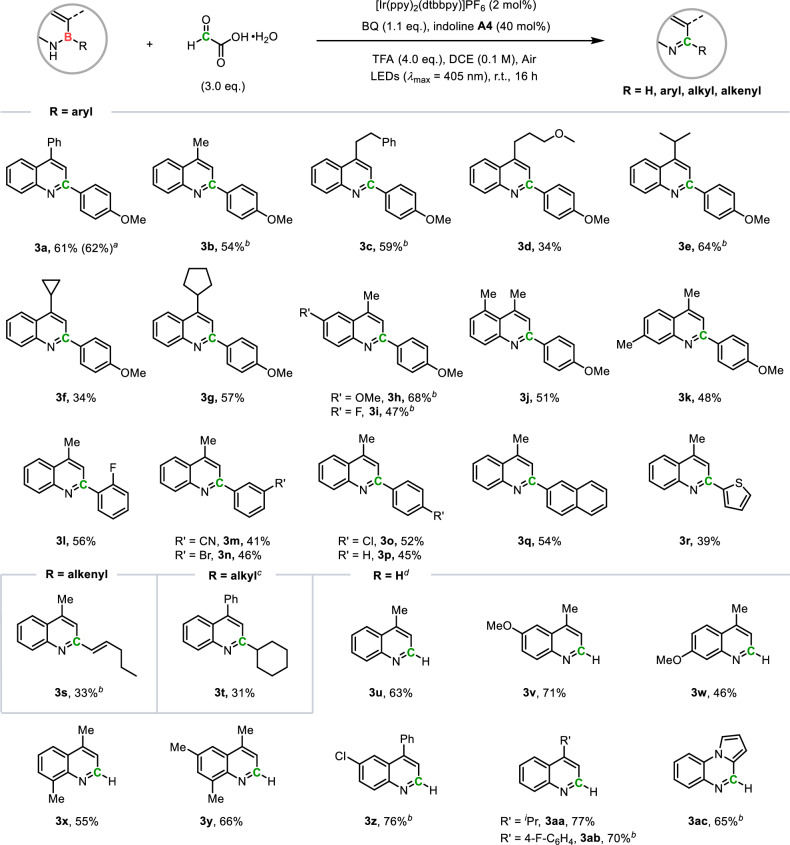
Scope of the substituent-rebound B-to-C single-atom swapping reaction. Isolated yields given. Reaction were performed on 0.2 mmol scale unless stated otherwise. ^*a*^Gram-scale reaction (4 mmol scale). ^*b*^0.1 mmol scale reaction. ^*c*^2-Cy-4-Ph-1,2-benzazaborine (0.2 mmol), glyoxylic acid monohydrate (0.6 mmol), [Ir-F] (5 mol%), TFA (0.8 mmol), Ni(5,5’-dmbpy)Br_2_ (10 mol%), DCE (2 mL), r.t., Ar, blue LEDs (*λ*_max_ = 405 nm), 16 h. ^*d*^1,2-benzazaborine (0.2 mmol), pivaldehyde (0.6 mmol), [Ir(ppy)_2_(dtbbpy)]PF_6_ (1 mol%), DQ (0.22 mmol), TFA (1.4 mmol), toluene (2 mL), r.t., blue LEDs (*λ*_max_ = 405 nm), 16 h. BQ benzoquinone, DQ duroquinone. [Ir-F] =  [Ir(dF(CF_3_)ppy)_2_(dtbbpy)]PF_6_.

With the optimised conditions in hand, we evaluated the scope of this substituent-rebound B-to-C swapping reaction using a variety of 1,2-benzazaborines (Fig. 4). Substrates bearing methyl (**3b**), primary alkyl (**3c** and **3 d**), secondary alkyl (**3e**), cyclopropyl (**3 f**), and cyclopentyl (**3 g**) groups at the 4-position were readily amenable to the standard conditions. Both electron-withdrawing and electron-donating groups at the 6-position were well tolerated, affording the corresponding products in moderate to good yields (**3 h** and **3i**). Moreover, 1,2-benzazaborines bearing substituents at the 5- and 7-position (**3j** and **3k**) were well tolerated, providing the desired products in moderate yields. By contrast, 1,2-benzazaborines bearing C8 substitution did not afford the corresponding quinoline product, presumably due to the increase in steric hindrance around the nitrogen atom. Next, we investigated various aryl substituents at the boron atom. A diverse array of aryl groups could be successfully retained in the single-atom-edited quinoline products, including those featuring methoxy (**3a**), cyano (**3 m**), and halogen (**3 l,** **3n**, and **3o**) substituents, as well as naphthalene (**3q**) and thiophene (**3r**) moieties. Moreover, alkenyl groups were also tolerated, albeit furnishing the product in a diminished yield (**3 s**). We then directed our attention to determining whether B-alkyl groups could be similarly retained in our single-atom skeletal editing transformation. Interestingly, under standard conditions, no product formation was observed. We hypothesised that this limitation stems from the fact that alkyl groups do not typically migrate in Petasis borono-Mannich reactions^64^. To address this, we explored metal-catalysed cross-coupling strategies to assemble the desired aldehyde intermediate in situ, taking inspiration from the work of MacMillan and coworkers^65^, while also considering the insights provided by Wu and coworkers^66^. To our delight, employing Ni(5,5’-dmbpy)Br_2_ as the metal catalyst and [Ir(dF(CF_3_)ppy)_2_(dtbbpy)]PF_6_ ([Ir-F]) as the photocatalyst enabled the formation of the desired product (**3t**) in 31% yield (see Supplementary Information for proposed mechanism).

Subsequently, we sought to develop a protocol for substrates bearing a hydrogen atom at the boron centre. As these species were not amenable to our previously developed standard conditions, further optimisation was required (detailed in the Supplementary Information). Pleasingly, utilising duroquinone (DQ) as the oxidant and pivaldehyde as the carbon source provided access to the desired product (**3 u**) in 63% yield. This transformation operates via a distinct mechanism involving acid-promoted heterocycle opening, condensation with pivaldehyde, and the selective cleavage of the *tert*-butyl group during rearomatisation (see Supplementary Information for proposed mechanism). Various substituents on the 1,2-benzazaborine backbone were well tolerated in this reaction regardless of their position or electronic properties (**3u**–**3ab**). Finally, this protocol could be extended to the more complex heterocyclic system BN-pyrrolo[*1,2-a*]quinoline, which was smoothly converted into the corresponding pyrrolo[*1,2-a*]quinoxaline (**3ac**).

**Fig. 5:**
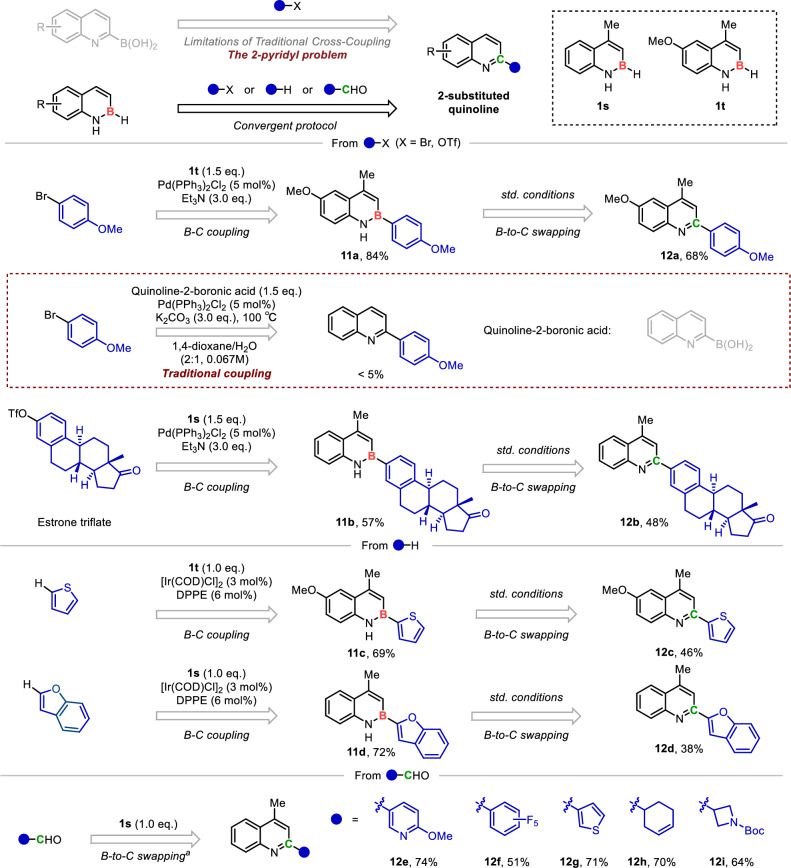
1,2-Azaborines as a convergent platform for the synthesis of 2-substituted quinolines. Isolated yields given. ^*a*^1,2-benzazaborine (0.2 mmol), aldehyde (0.6 mmol), [Ir(ppy)_2_(dtbbpy)]PF_6_ (1 mol%), DQ (0.22 mmol), TFA (1.4 mmol), toluene (2 mL, 0.1 M), r.t., blue LEDs (*λ*_max_ = 405 nm), 16 h. BQ benzoquinone, DQ duroquinone.

To further demonstrate the synthetic utility of this methodology, we explored its application in the preparation of 2-substituted quinolines (Fig. 5). These motifs represent privileged scaffolds that are widely prevalent in bioactive natural products and pharmaceutical agents^67,68^. However, the construction of 2-pyridine or 2-quinoline architectures via traditional cross-coupling frequently encounters the notorious ‘2-pyridyl problem'^69–71^. This fundamental challenge stems from the intrinsically low reactivity and poor stability of 2-pyridyl and 2-quinolinyl boronic acids, which typically lead to unsatisfactory coupling efficiencies when these species are employed as nucleophiles. Our strategy provides a conceptual circumvention of this longstanding limitation by utilising 1,2-azaborines as stable and robust nucleophilic equivalents. Specifically, 1,2-azaborines readily undergo palladium-catalysed cross-coupling with aryl halides and triflates (**11a** and **11b**)^17^, effectively bypassing the need for unstable 2-heteroaryl boronic acids. Following this initial transformation, our newly developed B-to-C single-atom swapping methodology was deployed to translate the 1,2-azaborines into the desired 2-substituted quinoline framework (**12a** and **12b**) in synthetically useful yields with complete retention of the original B-substituent. Notably, traditional cross-coupling methods to access related C2-arylated heteroarenes with quinoline-2-boronic acid failed to deliver more than trace product. This strategy extends well beyond pre-functionalised aryl electrophiles; simple arenes can also efficiently serve as coupling partners via dehydrogenative cross-coupling protocols (**11c** and **11 d**)^18^. These intermediates smoothly undergo the subsequent B-to-C swapping sequence to furnish the targeted 2-substituted quinolines. Furthermore, aldehyde substrates bearing diverse functional groups prove to be highly competent coupling partners (**12e**–**12i**). This broad compatibility and high modularity offer a robust, alternative strategy that significantly enriches the synthetic toolbox for the convergent construction of 2-substituted quinolines.

**Fig. 6:**
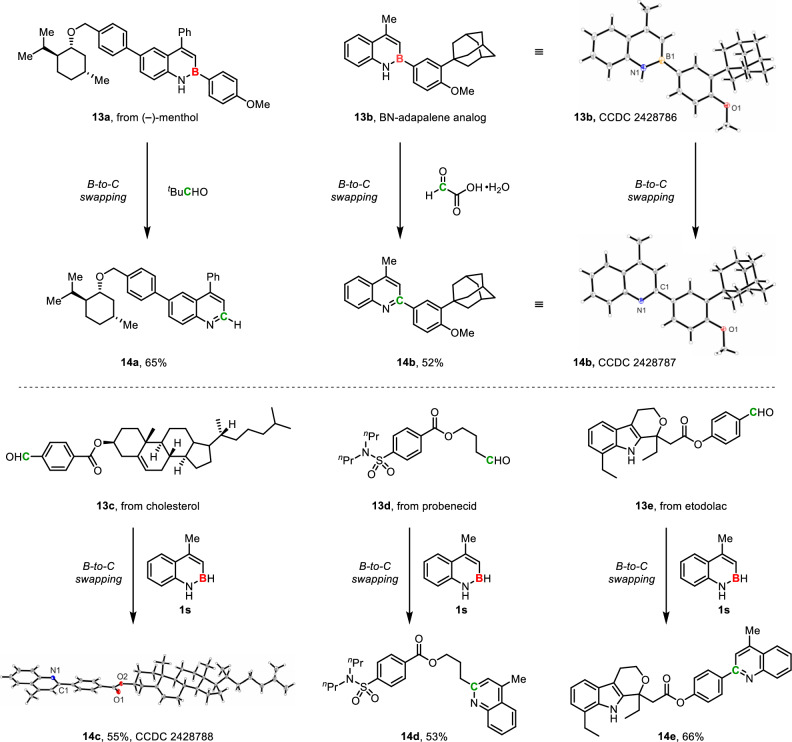
Applications of the B-to-C swapping reaction to late-stage skeletal editing. Access to carbon analogues of complex boron-containing scaffolds. Isolated yields given.

Given the broad applicability of these transformations, we then investigated the potential of applying B-to-C single-atom swapping to late-stage functionalisation (Fig. 6). To evaluate the functional group tolerance in structurally complex settings, we subjected various azaborine- and aldehyde-containing natural product analogues and pharmaceutical derivatives to our standard conditions. First, we demonstrated the ability of the B(aryl)-to-C(H) transformation, utilising pivaldehyde as the carbon source, to generate the C2-unsubstituted quinoline analogue (**14a**) of menthol-derived 1,2-benzazaborine **13a**. Subsequently, an azaborine analogue of the anti-inflammatory drug adapalene^72^ was successfully converted into the corresponding quinoline **14b** in 52% isolated yield. Notably, the core pharmaceutical scaffold remained fully intact during the transmutation, which was unequivocally confirmed by single-crystal X-ray diffraction analysis. Finally, we evaluated a representative set of complex aldehydes derived from cholesterol (**13c**), probenecid (**13 d**), and etodolac (**13e**). In all instances, these complex fragments effectively served as the carbon source in the B-to-C swapping reaction with 1,2-benzazaborines, affording modular access to highly functionalised quinoline derivatives **14c**, **14d**, and **14e**.

We have developed a substituent-rebound boron-to-carbon single-atom swapping protocol that enables the facile conversion of 1,2-benzazaborines into the corresponding quinolines. This unique reactivity represents a rare example of true single-atom swapping, wherein a core skeletal atom is edited while its original substituent is fully retained. Furthermore, this methodology demonstrates that 1,2-azaborines can serve as a robust alternative platform for the modular synthesis of 2-substituted quinolines. We anticipate that the strategy described herein will establish a new paradigm for utilising substituent-rebound single-atom swapping as a powerful tool in the ongoing development of skeletal editing transformations.

## Methods

In an oven-dried 10 mL Schlenk tube equipped with a PTFE-coated rare-earth extra power oval stirring bar, [Ir(ppy)_2_(dtbbpy)]PF_6_ (2 mol%, 3.6 mg), benzoquinone (BQ) (23.8 mg, 0.22 mmol, 1.1 eq.), 2-oxoacetic acid hydrate (55.0 mg, 0.60 mmol, 3.0 eq.), and 1,2-benzazaborine (**1**, 0.20 mmol, 1.0 eq.) were added under air. Then 1,2-dichloroethane (2.0 mL, 0.10 M), trifluoroacetic acid (TFA) (62 μL, 0.80 mmol, 4.0 eq.), and indoline (**A4**) (9.0 μL, 0.080 mmol, 0.40 eq.) were added under air. The vessel was sealed with a screw cap, and a needle was inserted through the cap. The reaction was then irradiated at 405 nm for 16 h at room temperature. After irradiation, sat. aq. Na_2_CO_3_ (2 mL) was slowly added to the reaction. The organic phase was separated and washed with brine, dried over Na_2_SO_4_ and concentrated under reduced pressure. Purification by flash column chromatography on SiO_2_, using pentane/EtOAc, afforded the corresponding product.

## Supplementary information

Supplementary Info

Transparent Peer Review file

## Data Availability

Materials and methods, experimental procedures, mechanistic studies and NMR spectra are available in the Supplementary Information and from the corresponding authors upon request. Crystallographic data for the structures reported in this Article have been deposited at the Cambridge Crystallographic Data Centre, under deposition numbers CCDC 2428786 (**13b**), 2428787 (**14b**), and 2428788 (**14c**). Copies of the data can be obtained free of charge via https://www.ccdc.cam.ac.uk/structures/.
