# Supplementary material for: Substituent-rebound skeletal editing for precise boron-to-carbon single-atom swapping
Source: Nat Commun. 2026 Jul 25;17:7268. doi: 10.1038/s41467-026-75992-9 (PMC13400731; doi:10.1038/s41467-026-75992-9)
Supplement: Supplementary file 1 — Supplementary Info [file 41467_2026_75992_MOESM1_ESM.pdf]

Supplementary Information

Substituent-rebound skeletal editing for precise boron-to-carbon single-atom swapping

Yan-Bo Li[1,#], Fu-Peng Wu[1,#], Jasper L. Tyler[1], Constantin G. Daniliuc[1], and Frank Glorius[1,*]

[1]*Organisch-Chemisches Institut, University of Münster, Münster, Germany.* [#]*These authors contributed equally.*

*Correspondence to: glorius@uni-muenster.de

Contents

1. General information 1
1.1 General remarks 1
1.2 Analytical techniques 1
1.3 Photochemical set-up 2
2. Preparation of starting materials 2
3. Reaction development 22
3.1 Optimisation of the substituent-rebound B-to-C single-atom swapping reaction 22
3.1.1 Reaction development 22
3.1.2 Optimisation of solvent 23
3.1.3 Sensitivity assessment 24
3.2 Optimisation of the alkyl-substituent-rebound B-to-C single-atom swapping reaction 25
3.2.1 Initial attempt at the reaction 25
3.2.2 Screening of the catalysts 25
3.3 Optimisation of the B-to-C swapping reaction using aldehydes as substrates 26
3.3.1 Optimisation of solvent 26
3.3.2 Optimisation of wavelength 27
3.3.3 Optimisation of stoichiometry and reaction concentration 28
3.4 General procedures for the B-to-C swapping reaction 29
4. Experimental data 31
4.1 Experimental data 31
4.2 Limitations of the B-to-C swapping reaction 48
5. Synthetic applications 49
6. Mechanistic investigations 52
6.1. UV-vis absorption spectrum 52
6.2. Stern-Volmer quenching studies 53
6.3. Cyclic voltammetry studies 55
6.4 Control experiments 57
6.4.1 Protodeborylation 57
6.4.2 Aldehyde formation 57
6.4.3 Rearomatisation 58
6.4.4 Control experiments to evaluate a possible CO-mediated pathway 58
6.5 Plausible mechanism for substituent-rebound B-to-C swapping reaction 59

6.6 Plausible mechanism for B-to-C swapping reaction using aldehydes as substrates 60
6.7 Plausible mechanism for alkyl-substituent-rebound B-to-C swapping reaction 60
6.8 A comparison experiment with classical Suzuki coupling conditions 62
7. X-Ray analysis 62
8. Copies of ^{1}H, ^{13}C, ^{19}F and ^{11}B NMR spectra 69
9. Supplementary references 162

1. General information

1.1 General remarks

All reagents were purchased from Alfa Aesar, Sigma-Aldrich, Merck, TCI, Fluorochem, Combi-blocks, VWR and used without further purification, except otherwise stated. All the solvents were bought from Acros in AcroSeal® bottles and were directly stored under 3 or 4 Å molecular sieves or dried using an activated alumina column drying system (MeCN, CH_2Cl_2, hexane, toluene, THF, Et_2O). All reactions were carried out in an oven-dried glassware under an atmosphere of argon using standard Schlenk technique, unless otherwise noted. Solvents for chromatographic purification (pentane, dichloromethane and EtOAc) were purchased as technical grade and purified by atmospheric pressure distillation. Reaction temperatures are referred to the temperature of the heating medium, unless otherwise stated. Flash chromatography was carried out using silica gel (Acros Organics, 0.035-0.070 mm, 60 Å) under a light positive pressure of argon, eluting with the specified solvent system as mentioned.

1.2 Analytical techniques

NMR-spectra were recorded on a Bruker Avance II 400 spectrometers. All spectral data was acquired at 295 K, unless otherwise stated. Deuterated solvents were purchased from Eurisotop ($CDCl_3$, deuteration >99.8%). ^1H and ^{13}C chemical shifts (δ) are quoted in parts per million (ppm) against tetramethylsilane (TMS, δ = 0.00 ppm) and were internally referenced to residual $CHCl_3$ (7.26 ppm for 1H, 77.0 ppm for ^{13}C) and d_6-Acetone (2.05 ppm for ^1H, 29.8 ppm for CH_3-^{13}C). Coupling constants (J) are reported in Hertz (Hz) to the nearest 0.1 Hz. The following abbreviations (or combinations thereof) were used to explain multiplicities: s = singlet, d = doublet, t = triplet, m = multiplet. **In the ^{13}C-NMR spectrum, the carbon directly connected to the boron atom appears as a broad peak, and the broad peak sometimes cannot be found in the ^{13}C NMR.** High-resolution mass spectra (HRMS) and gas chromatography mass spectrometry (GC-MS) were obtained by the MS service of the Organisch-Chemisches Institut, University of Münster, using electrospray ionisation (ESI) on a Bruker Daltonics, MicroToF spectrometer and calibrated using formate ion clusters. Exact GC-EI mass spectra were recorded on a Thermo Fisher Scientific Trace 1310 GC Exactive Orbitrap, equipped with a Thermo Gold TG-5SILMS (30 m, i.d. 0.25 mm, 0.25 µm) column. Thin layer chromatography was carried out on Merck silica gel 60 F254 pre-coated aluminium sheets and were visualized using UV light (254 nm) and stained with basic aqueous potassium permanganate.

1.3 Photochemical set-up

Photochemical reactions (Supplementary Figure 1-left), unless otherwise stated, were performed in a Hepatochem EvoluChem™ PhotoRedOx Box Duo device (Supplementary Figure 1-middle) and irradiated with two HepatoChem Inc HCK1012-01-010 (18 W, λ_{max} = 405 nm, Supplementary Figure 1-right). The reaction temperature was determined to be between 30 °C and 33 °C. A 10 mL Schlenk tube equipped with a PTFE-screw cap and a PTFE-coated rare-earth "extra power" oval stirring bar (10 x 5 mm) was used for small scale reactions.

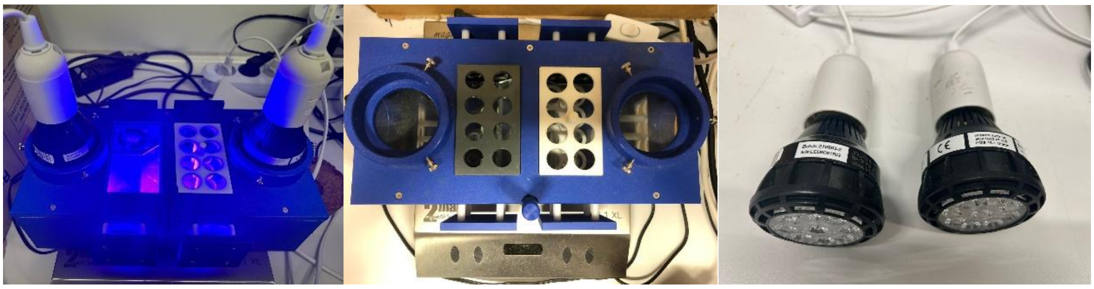

Supplementary Figure 1. Experimental set-ups: (left) EvoluChemTM PhotoRedOx Duo (courtesy of EvoluChemTM) with 2 × 18 W Blue LED; (middle) Hepatochem EvoluChem™ PhotoRedOx Box Duo device; (right) 18 W Blue LEDs (405 nm)

2. Preparation of starting materials

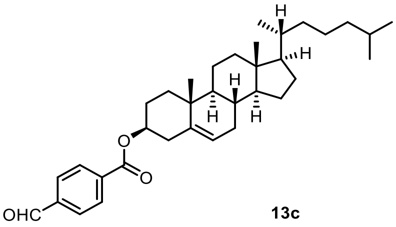

13c

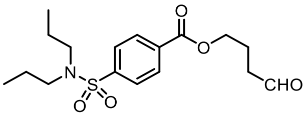

13d

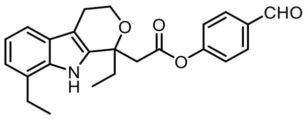

13e

Supplementary Figure 2. Preparation of aldehydes starting materials

Aldehydes **13d** and 1**3e** are known compounds and were prepared according to reported literature procedure.[1,2] The synthesis and characterization data for the new compound **13c** are provided below. All other aldehydes are commercially available.

(3*S*,8*S*,9*S*,10*R*,13*R*,14*S*,17*R*)-10,13-dimethyl-17-((*R*)-6-methylheptan-2-yl)-2,3,4,7,8,9,10,11,12,13,14,15,16,17-tetradecahydro-1*H*-cyclopenta[*a*]phenanthren-3-yl 4-formylbenzoate (13c)

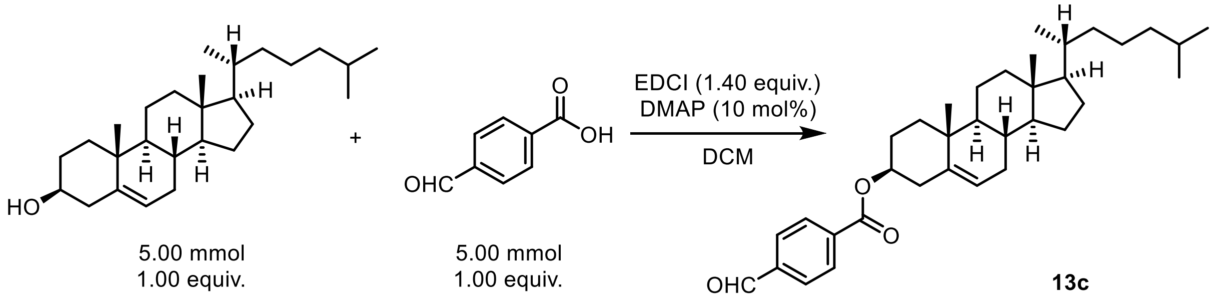

In a 25 mL round-bottom flask equipped with a PTFE-coated stirring bar, cholesterol (1.93 g, 5.00 mmol, 1.00 equiv.), EDCI hydrochloride (1.34 g, 7.00 mmol, 1.40 equiv.), 4-formylbenzoic acid (0.75 g, 5.00 mmol, 1.00 equiv.), DMAP (0.1 equiv.) were dissolved in DCM (10.0 mL, 0.5 M). The reaction was stirred at room temperature overnight, then poured in water (approx. 25 mL). The layers were separated and the aqueous layer was extracted with DCM (10 mL). The combined organic layers were washed with distilled water (25 mL), dried over $MgSO_4$ and concentrated *in vacuo*. The crude product was purified by column chromatography (SiO_2; pentane:EtOAc = 8:1) to afford **13c** (2.46 g, 95 %) as a white solid.

TLC: R_f = 0.3 (90:10 pentane:EtOAc).

NMR Spectroscopy:

^{1}H NMR (400 MHz, $CDCl_3$): δ_H 10.10 (s, 1H), 8.30–8.09 (m, 2H), 8.01–7.80 (m, 2H), 5.54–5.33 (m, 1H), 4.99–4.75 (m, 1H), 2.55–2.38 (m, 2H), 2.09–1.69 (m, 6H), 1.64–1.41 (m, 7H), 1.38–1.21 (m, 5H), 1.19–1.09 (m, 5H), 1.07 (s, 3H), 1.00 (ddd, *J* = 14.6, 8.7, 4.1 Hz, 3H), 0.92 (d, *J* = 6.5 Hz, 3H), 0.89–0.83 (m, 6H), 0.69 (s, 3H).

^{13}C NMR (101 MHz, $CDCl_3$): δ_C 191.84, 165.07, 139.53, 139.15, 135.97, 130.29, 129.59, 123.18, 75.51, 56.82, 56.27, 50.17, 42.46, 39.86, 39.66, 38.28, 37.13, 36.78, 36.32, 35.94, 32.07, 32.01, 28.37, 28.16, 27.97, 24.43, 23.97, 22.97, 22.71, 21.19, 19.51, 18.86, 12.01.

HRMS (ESI$^+$): m/z calculated for $C_{35}H_{50}O_3Na$ $[M+Na]^+$: 541.3652, found: 541.3653.

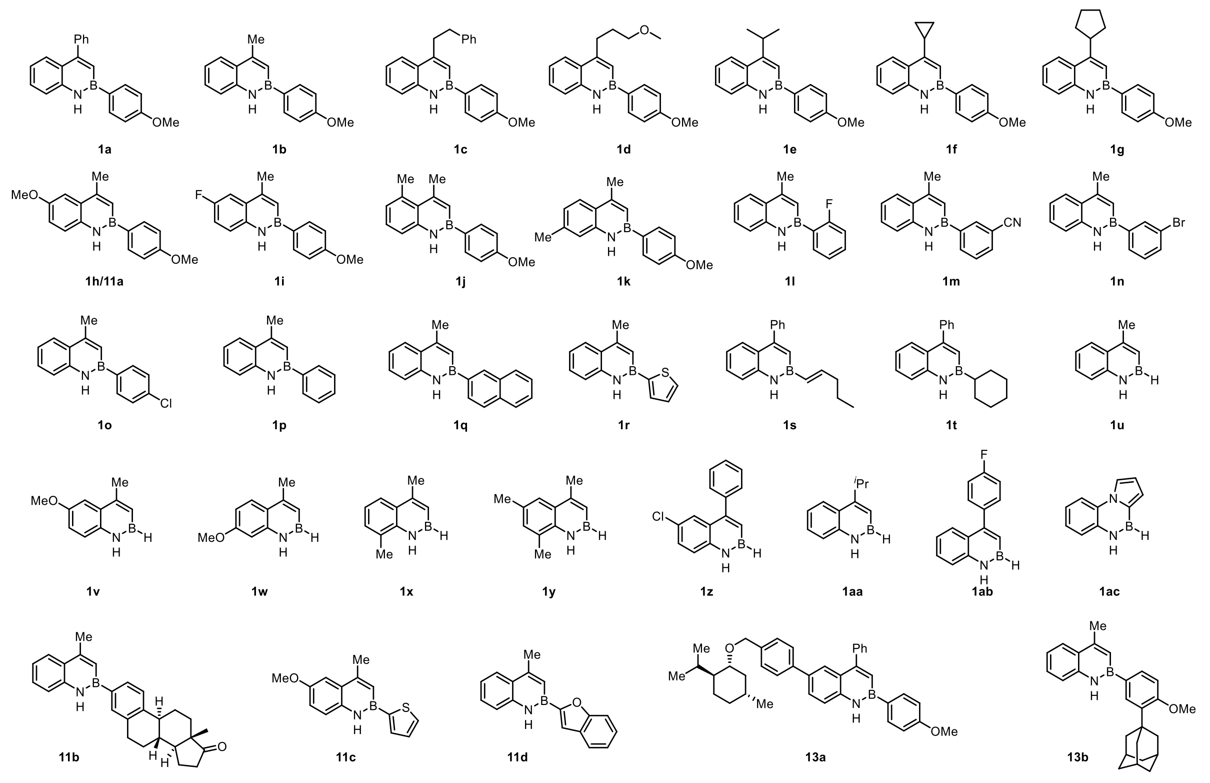

Supplementary Figure 3. Preparation of 1,2-benzazaborines starting materials

The 1,2-benzazaborines used in this study are listed above. 1,2-Benzazaborines **1n**, **1s**, and **1a'** are known compounds and were prepared according to reported literature procedure.[3–5] The synthesis and characterization data for the new compound are provided below.

General procedure 1

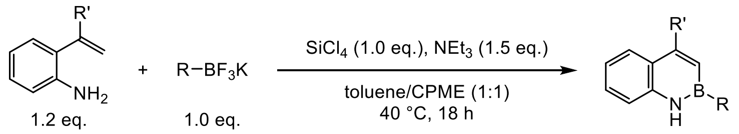

To an oven-dried vial equipped with a stir bar was added the potassium organotrifluoroborate (1 equiv). The vial was sealed with a cap lined with a disposable Teflon septum, evacuated under vacuum, and purged with Ar three times. CPME (1 mL/mmol) and toluene (1 ml/mmol) were added, followed by the 2-vinylaniline (1.2 eq.), $SiCl_4$ (1 eq.), and NEt_3 (1.5 eq.) under Ar. The resulting mixture was heated to 40 °C for 18 h, and then cooled to room temperature and diluted with *n*-pentanes (4 mL/mmol). The reaction mixture was filtered over a 2 *in.* plug of silica and flushed with 20% CH_2Cl_2/*n*-pentanes (10 mL/mmol). The solvent was removed and the residue was purified by flash column chromatography.

General procedure 2

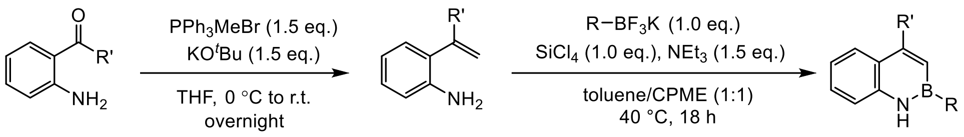

Step I: To a suspension of PPh_3MeBr (1.5 eq.) in dry THF (2.0 mL/mmol) at 0 °C was added KO^{t}Bu (1.5 eq.) in two portions. The resulting yellow mixture was allowed to stir for 30 minutes at room temperature before it was cooled to 0 °C again and the corresponding 2'-aminophenone (1 eq.) was added. Then, the reaction mixture was allowed to warm up to room temperature and stirred for overnight. After completion of the reaction, the mixture was diluted with EtOAc (2.0 mL/mmol) and saturated $NaHCO_3$ aqueous solution (2.0 mL/mmol). The phases were separated and the aqueous phase was extracted with EtOAc (2 x 4.0 mL/mmol). The combined organic layers were washed with distilled water (25 mL), dried over $MgSO_4$ and concentrated *in vacuo*. The residue was directly used for the next step without further purification.

To an oven-dried vial equipped with a stir bar was added the potassium organotrifluoroborate (1 equiv). The vial was sealed with a cap lined with a disposable Teflon septum, evacuated under vacuum, and purged with Ar three times. CPME (1 mL/mmol) and toluene (1 ml/mmol) were added, followed by the crude 2-vinylaniline (1.2 eq.), $SiCl_4$ (1 eq.), and NEt_3 (1.5 eq.) under Ar. The resulting mixture was heated to 40 °C for 18 h, and then cooled to room temperature and diluted with *n*-pentanes (4 mL/mmol). The reaction mixture was filtered over a 2 *in.* plug of silica and flushed with 20% CH_2Cl_2/*n*-pentanes (10 mL/mmol). The solvent was removed and the residue was purified by flash column chromatography.

General procedure 3

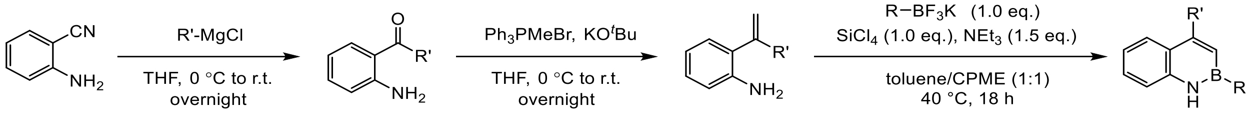

Step I: To a solution of 2-aminobenzonitrile (5.0 mmol) in Et_2O (15 mL) was added Grignard reagent (15 mmol, 3.0 eq.) dropwise at 0 °C. Then, the reaction mixture was allowed to warm up to room temperature and stirred overnight. After complete conversion of the nitrile, the suspension was cooled to 0 °C and 1 M HCl aqueous solution (20 mL) was added. The resulting mixture was vigorously stirred until complete hydrolysis of the corresponding imine. After saturated $NaHCO_3$ aqueous solution (20 mL) and EtOAc (20 mL) were added, the phases were separated and the aqueous phase was extracted with EtOAc (3 x 30 mL). The combined organic phases were washed with brine, dried over Na_2SO_4, filtered and concentrated under reduced pressure. The residue was directly used for the next step without further purification.

To a suspension of PPh_3MeBr (1.5 eq.) in dry THF (2.0 mL/mmol) at 0 °C was added KO^tBu (1.5 eq.) in two portions. The resulting yellow mixture was allowed to stir for 30 minutes at room temperature before it was cooled to 0 °C again and the crude 2'-aminophenone (1 eq.) was added. Then, the reaction mixture was allowed to warm up to room temperature and stirred for overnight. After completion of the reaction, the mixture was diluted with EtOAc (2.0 mL/mmol) and saturated $NaHCO_3$ aqueous solution (2.0 mL/mmol). The phases were separated and the aqueous phase was extracted with EtOAc (2 x 4.0 mL/mmol). The combined organic phases were washed with brine, dried over Na_2SO_4, filtered and concentrated under reduced pressure. The residue was directly used for the next step without further purification.

To an oven-dried vial equipped with a stir bar was added the potassium organotrifluoroborate (1 equiv). The vial was sealed with a cap lined with a disposable Teflon septum, evacuated under vacuum, and purged with Ar three times. CPME (1 mL/mmol) and toluene (1 ml/mmol) were added, followed by the crude 2-vinylaniline (1.2 eq.), $SiCl_4$ (1 eq.), and NEt_3 (1.5 eq.) under Ar. The resulting mixture was heated to 40 °C for 18 h, and then cooled to room temperature and diluted with *n*-pentanes (4 mL/mmol). The reaction mixture was filtered over a 2 *in.* plug of silica and flushed with 20% CH_2Cl_2/*n*-pentanes (10 mL/mmol). The solvent was removed and the residue was purified by flash column chromatography.

General procedure 4

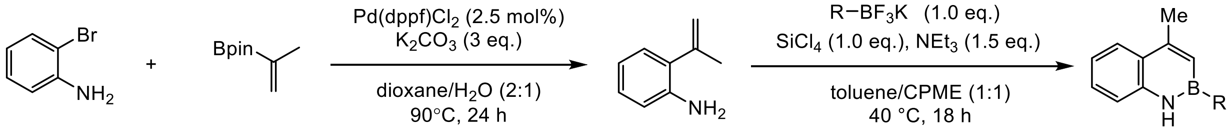

2-Bromoaniline derivatives (5 mmol, 1.0 eq.) were added to a suspension of potassium carbonate (2.07 g, 15 mmol, 3.0 eq.), isopropenylboronic acid pinacol ester (924 mg, 5.5 mmol, 1.1 eq.) and $Pd(dppf)Cl_2$ (91 mg, 0.25 mmol, 2.5 mol%) in a mixture of dioxane (20 mL) and H_2O (10 mL). The mixture of reaction was stirred for 24 h at 90 °C. After cooling to room temperature, the mixture was extracted with ethyl acetate (2 × 15 mL). The organic phases were combined, washed with brine, and dried over anhydrous Na_2SO_4. The solvent was removed under reduced pressure, and the residue was directly used for the next step without further purification.

To an oven-dried vial equipped with a stir bar was added the potassium organotrifluoroborate (1 equiv). The vial was sealed with a cap lined with a disposable Teflon septum, evacuated under vacuum, and purged with Ar three times. CPME (1 mL/mmol) and toluene (1 ml/mmol) were added, followed by the crude 2-vinylaniline (1.2 eq.), $SiCl_4$ (1 eq.), and NEt_3 (1.5 eq.) under Ar. The resulting mixture was heated to 40 °C for 18 h, and then cooled to room temperature and diluted with *n*-pentanes (4

mL/mmol). The reaction mixture was filtered over a 2 *in.* plug of silica and flushed with 20% CH_2Cl_2/*n*-pentanes (10 mL/mmol). The solvent was removed and the residue was purified by flash column chromatography.

General procedure 5

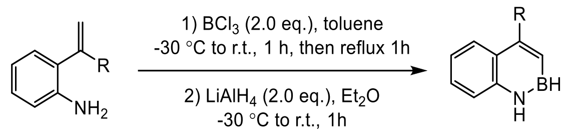

To a stirred solution of 2-vinylaniline derivatives (1.5 mmol, 1.0 eq.) in anhydrous toluene (30 mL), boron trichloride solution (1.0 M in hexane, 3.0 mL, 2.0 eq.) was added dropwise at -30 °C under an argon atmosphere. At the conclusion of the addition, the reaction mixture was allowed to warm to room temperature over 1 h, and then it was refluxed. After being stirred for 1 h, the mixture was concentrated under reduced pressure to afford the B–Cl intermediate. Then lithium aluminum hydride (114 mg, 3.0 mmol, 2.0 eq.) was added to Et_2O (3.0 mL) and the resulting suspension was cooled to −30°C. To this suspension, a solution of B–Cl intermediate in Et_2O (30 mL) was added dropwise. Then the reaction mixture was allowed to warm to room temperature. After being stirred for 1 h, a hydrogen chloride solution (4.0 M in 1,4-dioxane, 825 µL, 2.2 eq.) was added, and the resulting mixture was filtered through a pad of silica gel. The filtrate was concentrated in vacuo to afford desired benzazaborine.

General procedure 6

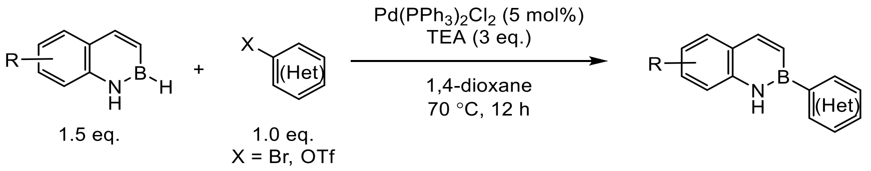

The starting materials were prepared following a modified procedure of reported protocols.[5] To an oven-dried 10 mL red-cap equipped with a stir bar was added the aryl halide or triflate (1 equiv.), 1,2-benzazaborine (1.5 eq.), and $Pd(PPh_3)_2Cl_2$ (5 mol%). The vial was sealed with a cap, evacuated under vacuum, and purged with Ar three times. 1,4-dioxane (0.1 ml/mmol) were added, followed by the NEt_3 (3 eq.) under Ar. The resulting mixture was heated to 70 °C for 12 h, and then cooled to room temperature and diluted with *n*-pentanes (4 mL/mmol). The reaction mixture was filtered over a 2 *in.* plug of silica and flushed with 20% CH_2Cl_2/*n*-pentanes (10 mL/mmol). The solvent was removed and the residue was purified by flash column chromatography.

General procedure 7

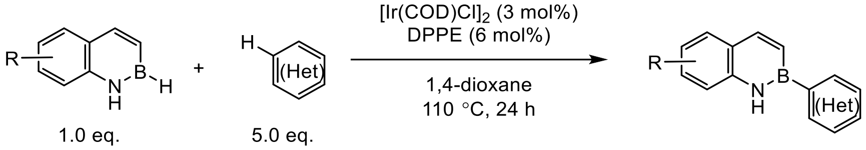

The starting materials were prepared following a modified procedure of reported protocols.[6] To an oven-dried 10 mL red-cap equipped with a stir bar was added 1,2-benzazaborine (1.0 eq.), DPPE (6 mol%), and $[Ir(COD)Cl]_2$ (3 mol%). The vial was sealed with a cap, evacuated under vacuum, and purged with Ar three times. 1,4-dioxane (1.0 ml/mmol) were added, followed by the arene (5 eq.) under Ar. The resulting mixture was heated to 110 °C for 24 h, and then cooled to room temperature and diluted with *n*-pentanes (4 mL/mmol). The reaction mixture was filtered over a 2 *in.* plug of silica and flushed with 20% CH_2Cl_2/*n*-pentanes (10 mL/mmol). The solvent was removed and the residue was purified by flash column chromatography.

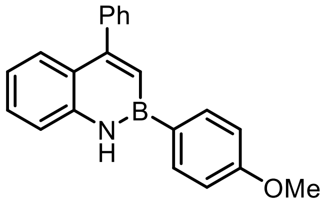

1a

2-(4-Methoxyphenyl)-4-phenyl-1,2-dihydrobenzo[*e*][*1,2*]azaborinine (1a)

The title compound was prepared according to **GP-1**. 1 mmol-scale reaction, 242 mg, 78% yield, white solid.

TLC: R_f = 0.3 (10:1 pentane:EtOAc).

^{1}H NMR (400 MHz, CDCl$_3$) δ 8.08 (s, 1H), 7.95–7.82 (m, 2H), 7.59 (d, *J* = 8.1 Hz, 1H), 7.53–7.35 (m, 7H), 7.15 (d, *J* = 2.0 Hz, 1H), 7.12–7.06 (m, 1H), 7.02 (dd, *J* = 8.6, 2.1 Hz, 2H), 3.87 (s, 3H).

^{13}C NMR (101 MHz, CDCl$_3$) δ 161.29, 156.60, 143.00, 141.01, 134.40, 129.17, 128.49, 128.38, 128.31, 127.52, 124.82, 120.88, 118.70, 114.00, 55.31.

^{11}B NMR (128 MHz, CDCl$_3$) δ 32.95.

HRMS (EI): m/z calculated for $C_{21}H_{18}BNO$ [M]$^+$: 311.1481, found: 311.1473.

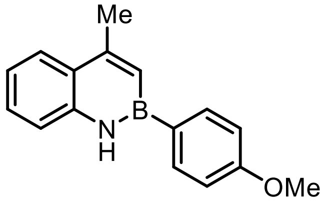

1b

2-(4-Methoxyphenyl)-4-methyl-1,2-dihydrobenzo[*e*][*1,2*]azaborinine (1b)

The title compound was prepared according to **GP-1**. 2 mmol-scale reaction, 470 mg, 94% yield, white solid.

TLC: R_f = 0.3 (10:1 pentane:EtOAc).

^{1}H NMR (400 MHz, CDCl$_3$) δ 8.07–7.76 (m, 4H), 7.45 (q, *J* = 6.3 Hz, 1H), 7.32 (d, *J* = 8.1 Hz, 1H), 7.29–7.17 (m, 1H), 7.17–6.98 (m, 3H), 3.88 (s, 3H), 2.67 (s, 3H).

^{13}C NMR (101 MHz, CDCl$_3$) δ 161.16, 151.45, 140.70, 134.29, 128.21, 125.85, 125.77, 120.88, 118.77, 113.97, 113.94, 55.27, 23.21.

^{11}B NMR (128 MHz, CDCl$_3$) δ 33.12.

HRMS (EI): m/z calculated for $C_{16}H_{16}BNO$ [M]$^+$: 249.1325, found: 249.1320.

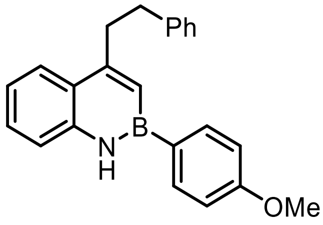

1c

2-(4-Methoxyphenyl)-4-phenethyl-1,2-dihydrobenzo[*e*][*1,2*]azaborinine (1c)

The title compound was prepared according to **GP-2**. 1 mmol-scale reaction, 241 mg, 71% yield, white solid.

TLC: R_f = 0.3 (10:1 pentane:EtOAc).

^{1}H NMR (400 MHz, CDCl$_3$) δ 8.04–7.90 (m, 2H), 7.91–7.74 (m, 2H), 7.49–7.42 (m, 1H), 7.39–7.29 (m, 5H), 7.27–7.20 (m, 2H), 7.12 (s, 1H), 7.08–6.97 (m, 2H), 3.89 (s, 3H), 3.30 (dd, *J* = 10.0, 6.6 Hz, 2H), 3.19–3.02 (m, 2H).

^{13}C NMR (101 MHz, CDCl$_3$) δ 161.18, 154.69, 142.29, 141.03, 134.30, 128.61, 128.55, 128.20, 126.16, 125.34, 124.75, 120.98, 119.14, 113.95, 55.29, 37.93, 36.31.

^{11}B NMR (128 MHz, CDCl$_3$) δ 33.25.

HRMS (EI): m/z calculated for $C_{23}H_{22}BNO$ $[M]^+$: 339.1794, found: 339.1785.

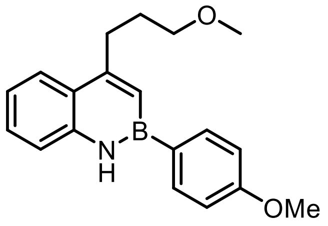

1d

2-(4-Methoxyphenyl)-4-(3-methoxypropyl)-1,2-dihydrobenzo[*e*][*1,2*]azaborinine (1d)

The title compound was prepared according to **GP-2**. 0.5 mmol-scale reaction, 147 mg, 96% yield, colorless oil.

TLC: R_f = 0.3 (5:1 pentane:EtOAc).

^{1}H NMR (400 MHz, CDCl$_3$) δ 7.93 (s, 1H), 7.91–7.83 (m, 3H), 7.42 (ddd, *J* = 8.2, 7.0, 1.4 Hz, 1H), 7.32 (dd, *J* = 8.1, 1.3 Hz, 1H), 7.19 (ddd, *J* = 8.3, 7.0, 1.3 Hz, 1H), 7.08 (d, *J* = 2.1 Hz, 1H), 7.04–6.99 (m, 2H), 3.87 (s, 3H), 3.51 (t, *J* = 6.3 Hz, 2H), 3.39 (s, 3H), 3.12–3.02 (m, 2H), 2.10–2.00 (m, 2H).

^{13}C NMR (101 MHz, CDCl$_3$) δ 161.18, 155.02, 141.03, 134.29, 128.15, 125.59, 124.89, 120.92, 119.05, 113.97, 72.48, 58.81, 55.30, 32.38, 29.84.

^{11}B NMR (128 MHz, CDCl$_3$) δ 33.41.

HRMS (EI): m/z calculated for $C_{19}H_{22}BNO_2$ $[M]^+$: 307.1744, found: 307.1736.

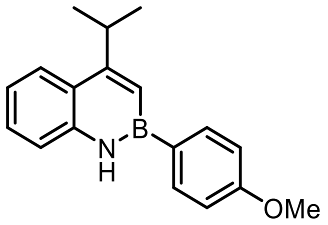

1e

4-Isopropyl-2-(4-methoxyphenyl)-1,2-dihydrobenzo[*e*][*1,2*]azaborinine (1e)

The title compound was prepared according to **GP-1**. 1 mmol-scale reaction, 230 mg, 83% yield, colorless oil.

TLC: R_f = 0.3 (10:1 pentane:EtOAc).

^{1}H NMR (400 MHz, CDCl$_3$) δ 8.17–7.82 (m, 4H), 7.46 (qd, *J* = 6.2, 2.8 Hz, 1H), 7.41–7.32 (m, 1H), 7.30–7.19 (m, 2H), 7.07 (dq, *J* = 8.1, 4.7 Hz, 2H), 3.90 (s, 3H), 3.62 (dt, *J* = 10.0, 6.8 Hz, 1H), 1.56–1.38 (m, 6H).

^{13}C NMR (101 MHz, CDCl$_3$) δ 161.63, 161.12, 141.11, 134.28, 127.91, 125.01, 124.61, 121.84, 120.80, 119.29, 113.95, 55.26, 30.10, 23.29.

^{11}B NMR (128 MHz, CDCl$_3$) δ 33.07.

HRMS (EI): m/z calculated for $C_{18}H_{20}BNO$ $[M]^+$: 277.1638, found: 277.1631.

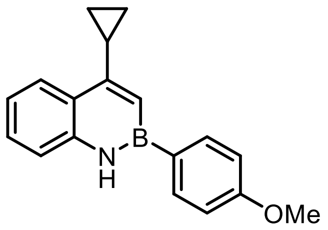

1f

4-Cyclopropyl-2-(4-methoxyphenyl)-1,2-dihydrobenzo[*e*][*1,2*]azaborinine (1f)

The title compound was prepared according to **GP-3**. 0.4 mmol-scale reaction, 90.0 mg, 82% yield, yellow oil.

TLC: R_f = 0.3 (10:1 pentane:EtOAc).

^{1}H NMR (400 MHz, $CDCl_3$) δ 8.26 (d, *J* = 8.1 Hz, 1H), 7.91 (s, 1H), 7.89–7.81 (m, 2H), 7.50–7.39 (m, 1H), 7.32 (dd, *J* = 8.1, 1.3 Hz, 1H), 7.26–7.19 (m, 1H), 7.04–6.99 (m, 2H), 6.98–6.94 (m, 1H), 3.88 (s, 3H), 2.33–2.22 (m, 1H), 1.10–1.02 (m, 2H), 0.84 (dtd, *J* = 5.4, 4.1, 2.0 Hz, 2H).

^{13}C NMR (101 MHz, $CDCl_3$) δ 161.14, 156.02, 140.75, 134.27, 128.19, 126.19, 126.02, 120.83, 118.76, 113.93, 55.29, 15.67, 6.98.

^{11}B NMR (128 MHz, $CDCl_3$) δ 33.16.

HRMS (EI): m/z calculated for $C_{18}H_{18}BNO$ $[M]^+$: 275.1481, found: 275.1476.

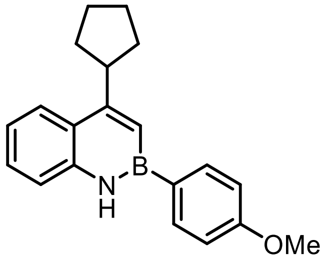

1g

4-Cyclopentyl-2-(4-methoxyphenyl)-1,2-dihydrobenzo[*e*][*1,2*]azaborinine (1g)

The title compound was prepared according to **GP-3**. 1 mmol-scale reaction, 184 mg, 61% yield, white solid.

TLC: R_f = 0.3 (10:1 pentane:EtOAc).

^{1}H NMR (400 MHz, $CDCl_3$) δ 8.00 (dd, *J* = 8.4, 3.8 Hz, 1H), 7.97–7.73 (m, 3H), 7.42 (dt, *J* = 8.0, 2.2 Hz, 1H), 7.32 (dq, *J* = 8.2, 1.5 Hz, 1H), 7.24–7.13 (m, 2H), 7.12–6.91 (m, 2H), 3.89 (s, 3H), 3.75–3.54 (m, 1H), 2.28–2.08 (m, 2H), 1.98–1.62 (m, 6H).

^{13}C NMR (101 MHz, $CDCl_3$) δ 161.10, 159.20, 141.07, 134.27, 127.91, 125.72, 125.47, 120.70, 119.09, 113.92, 55.29, 42.87, 33.33, 25.57.

^{11}B NMR (128 MHz, $CDCl_3$) δ 32.83.

HRMS (EI): m/z calculated for $C_{20}H_{22}BNO$ $[M]^+$: 303.1794, found: 303.1786.

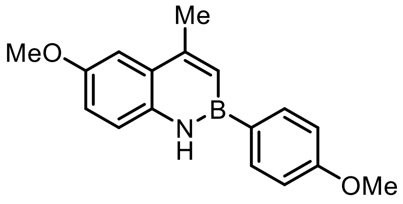

1h

6-methoxy-2-(4-methoxyphenyl)-4-methyl-1,2-dihydrobenzo[*e*][*1,2*]azaborinine (1h)

The title compound was prepared according to **GP-6**. 0.2 mmol-scale reaction, 46.7 mg, 84% yield, white solid.

TLC: R_f = 0.3 (8:1 pentane:EtOAc).

^{1}H NMR (400 MHz, CDCl$_3$) δ 7.95–7.77 (m, 3H), 7.29–7.24 (m, 2H), 7.14–7.08 (m, 2H), 7.07–6.97 (m, 2H), 3.93 (s, 3H), 3.90 (s, 3H), 2.66 (s, 3H).

^{13}C NMR (101 MHz, CDCl$_3$) δ 160.97, 153.90, 150.79, 135.35, 134.15, 126.17, 119.60, 116.69, 113.89, 108.31, 55.91, 55.26, 23.32.

^{11}B NMR (128 MHz, CDCl$_3$) δ 32.22.

HRMS (EI): m/z calculated for $C_{17}H_{18}NO_2B$ $[M]^+$: 279.1428, found: 279.1424.

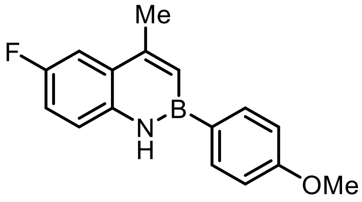

1i

6-Fluoro-2-(4-methoxyphenyl)-4-methyl-1,2-dihydrobenzo[*e*][*1,2*]azaborinine (1i)

The title compound was prepared according to **GP-4**. 1.0 mmol-scale reaction, 235.0 mg, 88% yield, white solid.

TLC: R_f = 0.3 (10:1 pentane:EtOAc).

^{1}H NMR (400 MHz, CDCl$_3$) δ 7.91–7.82 (m, 3H), 7.48 (dd, *J* = 10.5, 2.7 Hz, 1H), 7.25–7.12 (m, 3H), 7.03 (dd, *J* = 8.5, 2.2 Hz, 2H), 3.88 (s, 3H), 2.60 (s, 3H).

^{13}C NMR (101 MHz, CDCl$_3$) δ 161.15, 157.32 (d, *J* = 238.1 Hz), 150.60 (d, *J* = 3.2 Hz), 137.06, 134.22, 129.59, 126.34 (d, *J* = 7.6 Hz), 119.66 (d, *J* = 8.3 Hz), 115.96 (d, *J* = 24.1 Hz), 113.92, 110.79 (d, *J* = 22.3 Hz), 55.22, 23.10.

^{11}B NMR (128 MHz, CDCl$_3$) δ 32.79.

^{19}F NMR (376 MHz, CDCl$_3$) δ -122.22.

HRMS (EI): m/z calculated for $C_{16}H_{15}NOBF$ $[M]^+$: 267.1228, found: 267.1225.

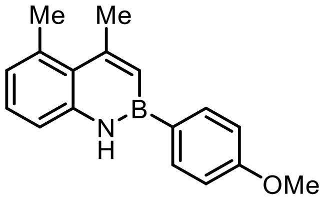

1j

2-(4-Methoxyphenyl)-4,5-dimethyl-1,2-dihydrobenzo[*e*][*1,2*]azaborinine (1j)

The title compound was prepared according to **GP-4**. 1.0 mmol-scale reaction, 189.1 mg, 72% yield, white solid.

TLC: R_f = 0.4 (10:1 pentane:EtOAc).

^{1}H NMR (400 MHz, CDCl$_3$) δ 8.14 (s, 1H), 8.12 – 8.05 (m, 2H), 7.50 – 7.46 (m, 1H), 7.40 (dd, *J* = 8.2, 1.5 Hz, 1H), 7.29 – 7.14 (m, 4H), 4.11 (s, 3H), 3.12 (s, 3H), 3.08 (s, 3H).

^{13}C NMR (101 MHz, CDCl$_3$) δ 161.10, 153.50, 142.51, 137.35, 134.21, 127.56, 125.57, 125.51, 118.33, 113.92, 55.28, 30.23, 26.27.

^{11}B NMR (128 MHz, CDCl$_3$) δ 32.91.

HRMS (EI): m/z calculated for $C_{17}H_{18}NOB$ $[M]^+$: 263.1479, found: 263.1476.

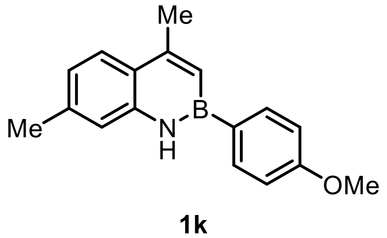

1k

2-(4-Methoxyphenyl)-4,7-dimethyl-1,2-dihydrobenzo[*e*][*1,2*]azaborinine (1k)

The title compound was prepared according to **GP-4**. 1.0 mmol-scale reaction, 205.4 mg, 78% yield, white solid.

TLC: R_f = 0.4 (10:1 pentane:EtOAc).

^{1}H NMR (400 MHz, CDCl$_3$) δ 7.95 – 7.83 (m, 3H), 7.75 – 7.68 (m, 1H), 7.12 (s, 1H), 7.07 – 6.99 (m, 4H), 3.88 (s, 3H), 2.64 (s, 3H), 2.48 (s, 3H).

^{13}C NMR (101 MHz, CDCl$_3$) δ 161.08, 151.41, 140.80, 138.36, 134.23, 125.67, 123.61, 122.34, 118.87, 113.91, 55.26, 23.15, 21.54.

^{11}B NMR (128 MHz, CDCl$_3$) δ 32.91.

HRMS (EI): m/z calculated for $C_{17}H_{18}NOB$ $[M]^+$: 263.1479, found: 263.1476.

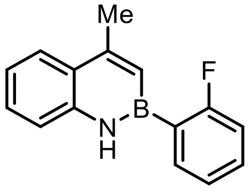

1l

2-(2-Fluorophenyl)-4-methyl-1,2-dihydrobenzo[*e*][*1,2*]azaborinine (1l)

The title compound was prepared according to **GP-1**. 1 mmol-scale reaction, 156 mg, 66% yield, white solid.

TLC: R_f = 0.3 (15:1 pentane:EtOAc).

^{1}H NMR (400 MHz, CDCl$_3$) δ 8.51 (s, 1H), 7.84 (ddt, *J* = 7.1, 4.7, 2.4 Hz, 1H), 7.71 (dd, *J* = 8.1, 2.2 Hz, 1H), 7.29 (ddd, *J* = 9.8, 4.6, 1.8 Hz, 2H), 7.20 (dd, *J* = 8.2, 1.5 Hz, 1H), 7.14–7.05 (m, 2H), 7.03–6.91 (m, 2H), 2.53 (s, 3H).

^{13}C NMR (101 MHz, CDCl$_3$) δ 167.40 (d, *J* = 243.9 Hz), 151.93, 140.38, 136.41 (d, *J* = 9.6 Hz), 131.64 (d, *J* = 9.4 Hz), 128.33, 125.77, 125.69, 124.36 (d, *J* = 2.8 Hz), 121.24, 119.30, 115.33 (d, *J* = 25.8 Hz), 23.21.

^{11}B NMR (128 MHz, CDCl$_3$) δ 32.14.

^{19}F NMR (377 MHz, CDCl$_3$) δ -106.78.

HRMS (EI): m/z calculated for $C_{15}H_{13}BFN$ $[M]^+$: 237.1125, found: 237.1116.

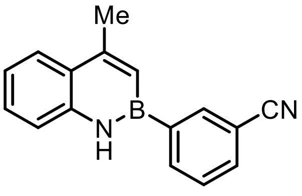

1m

3-(4-Methylbenzo[*e*][*1,2*]azaborinin-2(1*H*)-yl)benzonitrile (1m)

The title compound was prepared according to **GP-1**. 2 mmol-scale reaction, 363 mg, 74% yield, white solid.

TLC: R_f = 0.25 (10:1 pentane:EtOAc).

^{1}H NMR (400 MHz, CDCl$_3$) δ 8.17 (s, 1H), 8.09 (dt, *J* = 7.5, 1.3 Hz, 2H), 7.90–7.84 (m, 1H), 7.70 (dt, *J* = 7.7, 1.5 Hz, 1H), 7.55 (td, *J* = 7.6, 0.7 Hz, 1H), 7.49 (ddd, *J* = 8.3, 7.0, 1.4 Hz, 1H), 7.39 (dd, *J* = 8.1, 1.3 Hz, 1H), 7.27 (ddd, *J* = 8.2, 7.0, 1.3 Hz, 1H), 7.03 (d, *J* = 1.1 Hz, 1H), 2.68 (d, *J* = 1.1 Hz, 3H).

^{13}C NMR (101 MHz, CDCl$_3$) δ 152.98, 140.23, 136.90, 136.41, 132.68, 128.84, 128.63, 125.96, 125.92, 121.63, 119.53, 119.10, 112.41, 23.21.

^{11}B NMR (128 MHz, CDCl$_3$) δ 32.45.

HRMS (EI): m/z calculated for $C_{16}H_{13}BN_2$ $[M]^+$: 244.1172, found: 244.1165.

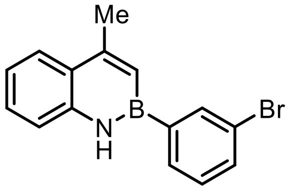

1n

2-(3-Bromophenyl)-4-methyl-1,2-dihydrobenzo[*e*][*1,2*]azaborinine (1n)

The title compound was prepared according to **GP-1**. 2 mmol-scale reaction, 526 mg, 51% yield, white solid.

TLC: R_f = 0.5 (20:1 pentane:EtOAc).

^{1}H NMR (400 MHz, CDCl$_3$) δ 8.03–7.97 (m, 1H), 7.95 (s, 1H), 7.85 (d, *J* = 8.1 Hz, 1H), 7.82–7.74 (m, 1H), 7.63–7.52 (m, 1H), 7.46 (ddd, *J* = 8.3, 7.0, 1.4 Hz, 1H), 7.33 (t, *J* = 7.6 Hz, 2H), 7.28–7.19 (m, 1H), 7.09–6.94 (m, 1H), 2.66 (d, *J* = 1.2 Hz, 3H).

^{13}C NMR (101 MHz, CDCl$_3$) δ 152.43, 140.29, 135.52, 132.39, 131.15, 131.13, 130.03, 128.44, 125.91, 123.21, 121.38, 118.99, 23.19.

^{11}B NMR (128 MHz, CDCl$_3$) δ 32.63.

HRMS (EI): m/z calculated for $C_{15}H_{13}BBrN$ $[M]^+$: 297.0324, found: 297.0320.

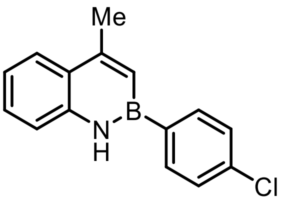

1o

2-(4-Chlorophenyl)-4-methyl-1,2-dihydrobenzo[*e*][*1,2*]azaborinine (1o)

The title compound was prepared according to **GP-1**. 1 mmol-scale reaction, 220 mg, 87% yield, white solid.

TLC: R_f = 0.4 (20:1 pentane:EtOAc).

^{1}H NMR (400 MHz, $CDCl_3$) δ 7.96 (s, 1H), 7.91–7.75 (m, 3H), 7.50–7.42 (m, 3H), 7.38–7.30 (m, 1H), 7.29–7.22 (m, 1H), 7.08–6.98 (m, 1H), 2.67 (s, 3H).

^{13}C NMR (101 MHz, $CDCl_3$) δ 152.24, 140.39, 135.83, 134.07, 128.51, 128.42, 125.92, 125.87, 121.29, 118.94, 118.94, 23.20.

^{11}B NMR (128 MHz, $CDCl_3$) δ 33.00.

HRMS (EI): m/z calculated for $C_{15}H_{13}BClN$ $[M]^+$: 253.0830, found: 253.0824.

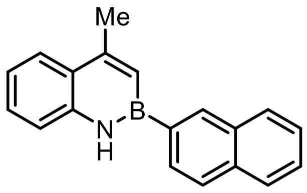

1q

4-Methyl-2-(naphthalen-2-yl)-1,2-dihydrobenzo[*e*][*1,2*]azaborinine (1q)

The title compound was prepared according to **GP-1**. 1 mmol-scale reaction, 236 mg, 88% yield, white solid.

TLC: R_f = 0.3 (10:1 pentane:EtOAc).

^{1}H NMR (400 MHz, $CDCl_3$) δ 8.45 (d, *J* = 4.2 Hz, 1H), 8.14 (s, 1H), 8.04–7.86 (m, 5H), 7.61–7.46 (m, 3H), 7.38 (dd, *J* = 8.1, 2.6 Hz, 1H), 7.32–7.23 (m, 2H), 2.73 (s, 3H).

^{13}C NMR (101 MHz, $CDCl_3$) δ 151.92, 140.62, 134.32, 133.55, 129.30, 129.27, 128.62, 128.61, 128.33, 127.89, 127.87, 127.54, 126.54, 126.05, 125.93, 121.16, 23.25.

^{11}B NMR (128 MHz, $CDCl_3$) δ 33.36.

HRMS (EI): m/z calculated for $C_{19}H_{16}BN$ $[M]^+$: 269.1376, found: 269.1369.

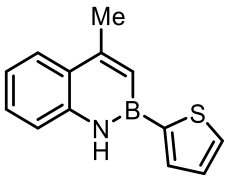

1r

4-Methyl-2-(thiophen-2-yl)-1,2-dihydrobenzo[*e*][*1,2*]azaborinine (1r)

The title compound was prepared according to **GP-1**. 2 mmol-scale reaction, 244 mg, 54% yield, white solid.

TLC: R_f = 0.5 (20:1 pentane:EtOAc).

^{1}H NMR (400 MHz, $CDCl_3$) δ 7.89 (s, 1H), 7.83 (d, *J* = 8.0 Hz, 1H), 7.72 (dt, *J* = 3.3, 1.8 Hz, 1H), 7.66 (dt, *J* = 4.6, 1.6 Hz, 1H), 7.45 (ddd, *J* = 8.3, 7.0, 1.4 Hz, 1H), 7.30 (tt, *J* = 6.7, 2.3 Hz, 2H), 7.25–7.18 (m, 1H), 7.03 (s, 1H), 2.66 (s, 3H).

^{13}C NMR (101 MHz, $CDCl_3$) δ 151.96, 140.36, 133.48, 130.56, 128.91, 128.40, 125.89, 125.85, 121.09, 118.79, 23.10.

^{11}B NMR (128 MHz, $CDCl_3$) δ 30.53.

HRMS (EI): m/z calculated for $C_{13}H_{12}BNS$ $[M]^+$: 225.0784, found: 225.0786.

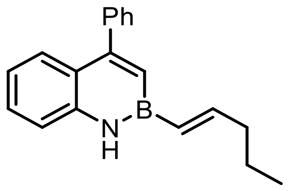

1s

(*E*)-2-(pent-1-en-1-yl)-4-phenyl-1,2-dihydrobenzo[*e*][*1,2*]azaborinine (1s)

The title compound was prepared according to **GP-1**. 1 mmol-scale reaction, 133.1 mg, 82% yield, colorless oil.

TLC: R_f = 0.5 (20:1 pentane:EtOAc).

^{1}H NMR (400 MHz, CDCl$_3$) δ 7.71 (s, 1H), 7.60–7.54 (m, 1H), 7.51–7.36 (m, 6H), 7.30–7.26 (m, 1H), 7.11–7.02 (m, 1H), 6.93 (d, *J* = 1.9 Hz, 1H), 6.76 (dt, *J* = 17.6, 6.5 Hz, 1H), 6.07 (dt, *J* = 17.5, 1.5 Hz, 1H), 2.44–2.00 (m, 2H), 1.67–1.34 (m, 2H), 0.98 (t, *J* = 7.4 Hz, 3H).

^{13}C NMR (101 MHz, CDCl$_3$) δ 155.74, 150.22, 143.08, 140.90, 129.12, 128.24, 128.22, 127.36, 124.86, 120.61, 118.47, 38.47, 22.16, 13.96.

^{11}B NMR (128 MHz, CDCl$_3$) δ 31.85.

HRMS (EI): m/z calculated for $C_{19}H_{20}NB$ $[M]^+$: 273.1687, found: 273.1685.

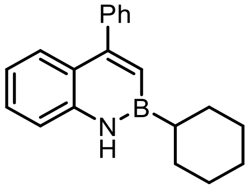

1t

2-cyclohexyl-4-phenyl-1,2-dihydrobenzo[*e*][*1,2*]azaborinine (1t)

The title compound was prepared according to **GP-1**. 3 mmol-scale reaction, 657.6 mg, 76% yield, colorless oil.

TLC: R_f = 0.5 (20:1 pentane:EtOAc).

^{1}H NMR (400 MHz, CDCl$_3$) δ 7.70 (s, 1H), 7.57 (d, *J* = 8.2 Hz, 1H), 7.47–7.37 (m, 6H), 7.32–7.27 (m, 1H), 7.06 (ddd, *J* = 8.2, 7.1, 1.4 Hz, 1H), 6.78–6.73 (m, 1H), 1.99–1.90 (m, 2H), 1.81–1.74 (m, 3H), 1.44–1.33 (m, 6H).

^{13}C NMR (101 MHz, CDCl$_3$) δ 155.76, 143.08, 140.69, 129.18, 128.38, 128.21, 128.10, 127.35, 125.44, 124.52, 120.61, 118.48, 29.94, 27.95, 27.19.

^{11}B NMR (128 MHz, CDCl$_3$) δ 37.49.

HRMS (EI): m/z calculated for $C_{20}H_{22}NB$ $[M]^+$: 287.1843, found: 287.1843.

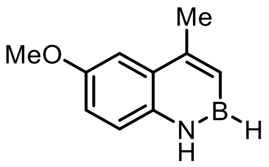

1v

6-Methoxy-4-methyl-1,2-dihydrobenzo[*e*][*1,2*]azaborinine (1v)

The title compound was prepared according to **GP-5**. 5 mmol-scale reaction, 0.64 g, 74% yield for two steps, white solid.

TLC: R_f = 0.30 (10:1 pentane:EtOAc).

^{1}H NMR (400 MHz, CDCl$_3$) δ 8.01 (br, 1H), 7.28–7.26 (m, 1H), 7.21 (d, *J* = 8.8 Hz, 1H), 7.09 (dd, *J* = 8.8, 2.8 Hz, 1H), 6.83 (dt, *J* = 2.7, 1.6 Hz, 1H), 5.03 (br, 1H), 3.89 (s, 3H), 2.59 (d, *J* = 1.1 Hz, 3H).

^{13}C NMR (101 MHz, CDCl$_3$) δ 154.15, 150.61, 135.05, 126.46, 119.78, 116.81, 108.03, 55.89, 23.12.

^{11}B NMR (128 MHz, CDCl$_3$) δ 30.92.

HRMS (EI): m/z calculated for $C_{10}H_{12}NOB$ [M]$^+$: 172.1043, found: 172.1045.

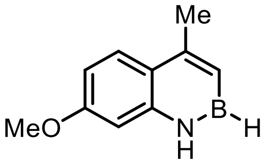

1w

7-Methoxy-4-methyl-1,2-dihydrobenzo[*e*][*1,2*]azaborinine (1w)

The title compound was prepared according to **GP-5**. 5 mmol-scale reaction, 0.60 g, 70% yield for two steps, white solid.

TLC: R_f = 0.30 (10:1 pentane:EtOAc).

^{1}H NMR (400 MHz, CDCl$_3$) δ 7.99 (br, 1H), 7.74 (d, *J* = 8.9 Hz, 1H), 6.85 (dt, *J* = 8.9, 2.0 Hz, 1H), 6.72 (d, *J* = 2.5 Hz, 1H), 6.68–6.61 (m, 1H), 4.92 (br, 1H), 3.88 (s, 3H), 2.58 (d, *J* = 1.2 Hz, 3H).

^{13}C NMR (101 MHz, CDCl$_3$) δ 159.67, 151.37, 141.74, 127.24, 120.41, 110.16, 101.48, 55.53, 23.01.

^{11}B NMR (128 MHz, CDCl$_3$) δ 31.93 ppm.

HRMS (EI): m/z calculated for $C_{10}H_{12}NOB$ [M]$^+$: 173.1008, found: 173.1004.

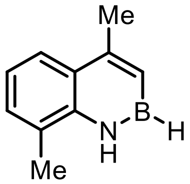

1x

4,8-Dimethyl-1,2-dihydrobenzo[*e*][*1,2*]azaborinine (1x)

The title compound was prepared according to **GP-5**, 5 mmol-scale reaction, 0.23 g, 29% yield for two steps, yellow solid.

TLC: R_f = 0.65 (20:1 pentane:EtOAc).

^{1}H NMR (400 MHz, CDCl$_3$) δ 8.05 (br, 1H), 7.31–7.18 (m, 1H), 7.12 (dd, *J* = 8.2, 1.4 Hz, 1H), 7.00 (ddd, *J* = 7.2, 1.6, 0.9 Hz, 1H), 6.78 (tt, *J* = 2.1, 1.2 Hz, 1H), 4.85 (br, 1H), 2.92–2.77 (m, 6H).

^{13}C NMR (101 MHz, CDCl$_3$) δ 153.30, 142.07, 137.45, 127.35, 125.87, 125.85, 118.40, 30.05, 26.33.

^{11}B NMR (128 MHz, CDCl$_3$) δ 30.86.

HRMS (EI): m/z calculated for $C_{10}H_{12}NB$ [M]$^+$: 157.1059, found: 157.1055.

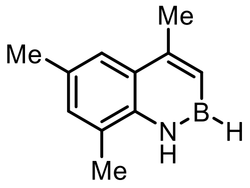

1y

4,6,8-Trimethyl-1,2-dihydrobenzo[*e*][*1,2*]azaborinine (1y)

The title compound was prepared according to **GP-5**. 5 mmol-scale reaction, 0.37 g, 44% yield for two steps, yellow solid.

TLC: R_f = 0.70 (20:1 pentane:EtOAc).

^{1}H NMR (400 MHz, CDCl$_3$) δ 8.10 (br, 1H), 7.72–7.46 (m, 1H), 7.22–7.08 (m, 1H), 6.96–6.72 (m, 1H), 4.99 (br, 1H), 2.61 (d, *J* = 1.1 Hz, 3H), 2.52 (s, 3H), 2.44 (s, 3H).

^{13}C NMR (101 MHz, CDCl$_3$) δ 151.56, 136.87, 130.65, 129.73, 125.56, 124.63, 123.90, 23.48, 21.31, 17.46.

^{11}B NMR (128 MHz, CDCl$_3$) δ 31.29.

HRMS (EI): m/z calculated for $C_{11}H_{14}NB$ $[M]^+$: 170.1250, found: 170.1247.

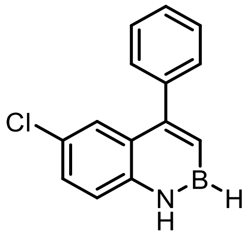

1z

6-Chloro-4-phenyl-1,2-dihydrobenzo[*e*][*1,2*]azaborinine (1z)

The title compound was prepared according to **GP-5**. 3.5 mmol-scale reaction, 260 mg, 31% yield for two steps, white solid.

TLC: R_f = 0.5 (20:1 pentane:EtOAc).

^{1}H NMR (400 MHz, CDCl$_3$) δ 8.23 (s, 1H), 7.66–7.55 (m, 1H), 7.52–7.43 (m, 3H), 7.43–7.37 (m, 3H), 7.29 (d, *J* = 8.6 Hz, 1H), 6.98–6.91 (m, 1H), 5.12 (br, 1H).

^{13}C NMR (101 MHz, CDCl$_3$) δ 155.41, 141.97, 138.94, 129.05, 128.48, 128.46, 127.83, 127.55, 126.61, 126.05, 120.07.

^{11}B NMR (128 MHz, CDCl$_3$) δ 31.56.

HRMS (EI): m/z calculated for $C_{14}H_{11}BClN$ $[M]^+$: 239.0673, found: 239.0666.

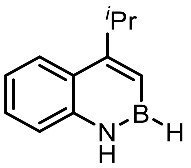

1aa

4-Isopropyl-1,2-dihydrobenzo[*e*][*1,2*]azaborinine (1aa)

The title compound was prepared according to **GP-5**. 2.3 mmol-scale reaction, 209 mg, 54% yield for three steps, colorless oil.

TLC: R_f = 0.5 (20:1 pentane:EtOAc).

^{1}H NMR (400 MHz, CDCl$_3$) δ 8.15–7.93 (m, 2H), 7.43 (ddd, J = 8.3, 7.0, 1.4 Hz, 1H), 7.30 (dd, J = 8.1, 1.4 Hz, 1H), 7.26–7.18 (m, 1H), 7.01–6.87 (m, 1H), 3.76–3.23 (m, 1H), 1.35 (d, J = 6.9 Hz, 6H).

^{13}C NMR (101 MHz, CDCl$_3$) δ 161.46, 140.63, 127.69, 125.07, 124.82, 121.19, 119.36, 29.96, 23.28.

^{11}B NMR (128 MHz, CDCl$_3$) δ 31.81.

HRMS (EI): m/z calculated for $C_{11}H_{14}BN$ [M]$^+$: 171.1219, found: 171.1214.

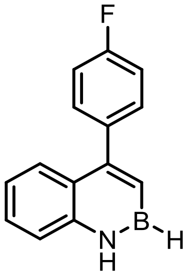

1ab

4-(4-Fluorophenyl)-1,2-dihydrobenzo[*e*][*1,2*]azaborinine (1ab)

The title compound was prepared according to **GP-5**. 2 mmol-scale reaction, 253 mg, 55% yield for two steps, white solid.

TLC: R_f = 0.6 (20:1 pentane:EtOAc).

^{1}H NMR (400 MHz, CDCl$_3$) δ 8.25 (s, 1H), 7.58 (d, *J* = 8.2 Hz, 1H), 7.45 (ddt, *J* = 8.3, 7.0, 1.5 Hz, 1H), 7.42–7.29 (m, 3H), 7.22–7.07 (m, 3H), 6.87 (s, 1H).

^{13}C NMR (101 MHz, CDCl$_3$) δ 162.43 (d, *J* = 246.1 Hz), 155.21, 140.53, 138.62, 130.76 (d, *J* = 7.9 Hz), 128.29 (d, *J* = 19.1 Hz), 124.96, 121.40, 118.85, 115.19 (d, *J* = 21.4 Hz).

^{11}B NMR (128 MHz, CDCl$_3$) δ 31.38.

^{19}F NMR (376 MHz, CDCl$_3$) δ -115.19.

HRMS (EI): m/z calculated for $C_{14}H_{11}BFN$ [M]$^+$: 223.0969, found: 223.0957.

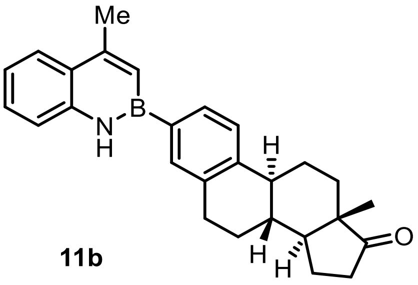

11b

(8*R*,9*S*,13*S*,14*S*)-13-Methyl-3-(4-methylbenzo[*e*][*1,2*]azaborinin-2(1*H*)-yl)-6,7,8,9,11,12,13,14,15,16-decahydro-17*H*-cyclopenta[*a*]phenanthren-17-one (11b)

The title compound was prepared according to **GP-6**. 0.2 mmol-scale reaction, 45.1 mg, 57% yield, white solid.

TLC: R_f = 0.25 (15:1 pentane:EtOAc).

^{1}H NMR (400 MHz, CDCl$_3$) δ 7.99 (s, 1H), 7.84 (d, *J* = 8.1 Hz, 1H), 7.71 (d, *J* = 7.7 Hz, 1H), 7.66 (s, 1H), 7.48–7.38 (m, 2H), 7.34 (d, *J* = 8.1 Hz, 1H), 7.22 (ddd, *J* = 8.3, 4.9, 3.3 Hz, 1H), 7.09 (s, 1H), 3.03 (d, *J* = 5.4 Hz, 2H), 2.66 (s, 3H), 2.51 (ddd, *J* = 13.1, 9.0, 6.6 Hz, 2H), 2.44–2.32 (m, 1H), 2.23–2.12 (m, 1H), 2.11–1.98 (m, 3H), 1.74–1.49 (m, 6H), 0.94 (s, 3H).

^{13}C NMR (101 MHz, CDCl$_3$) δ 221.07, 151.68, 141.44, 140.60, 136.24, 133.75, 130.17, 128.24, 125.87, 125.35, 121.00, 118.86, 50.72, 48.17, 44.80, 38.30, 36.02, 31.78, 29.63, 26.75, 25.81, 23.23, 21.76, 14.01.

^{11}B NMR (128 MHz, CDCl$_3$) δ 33.29.

HRMS (ESI): m/z calculated for $C_{27}H_{30}NOBNa$ [M+Na]$^+$: 418.2318, found: 418.2310.

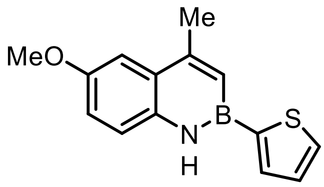

11c

6-methoxy-4-methyl-2-(thiophen-2-yl)-1,2-dihydrobenzo[*e*][*1,2*]azaborinine (11c)

The title compound was prepared according to **GP-7**. 0.1 mmol-scale reaction, 17.7 mg, 69% yield, white solid.

TLC: R_f = 0.35 (10:1 pentane:EtOAc).

^{1}H NMR (400 MHz, CDCl$_3$) δ 7.88–7.76 (m, 1H), 7.69 (dd, *J* = 3.4, 1.0 Hz, 1H), 7.64 (dd, *J* = 4.7, 0.9 Hz, 1H), 7.29 (dd, *J* = 4.7, 3.4 Hz, 1H), 7.26–7.21 (m, 2H), 7.10 (dd, *J* = 8.8, 2.8 Hz, 1H), 7.05–7.02 (m, 1H), 3.90 (s, 3H), 2.62 (d, *J* = 1.2 Hz, 3H).

^{13}C NMR (101 MHz, CDCl$_3$) δ 154.04, 151.27, 134.99, 133.18, 130.26, 128.86, 126.31, 119.64, 116.87, 108.33, 55.87, 23.20.

^{11}B NMR (128 MHz, CDCl$_3$) δ 30.01.

HRMS (EI): m/z calculated for $C_{14}H_{14}NOSB$ [M]$^+$: 255.0886, found: 255.0884.

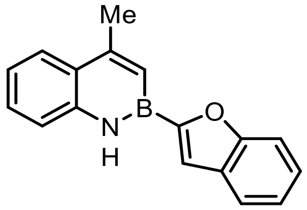

11d

2-(Benzofuran-2-yl)-4-methyl-1,2-dihydrobenzo[*e*][*1,2*]azaborinine (11d)

The title compound was prepared according to **GP-7**. 0.1 mmol-scale reaction, 18.7 mg, 72% yield, white solid.

TLC: R_f = 0.5 (20:1 pentane:EtOAc).

^{1}H NMR (400 MHz, CDCl$_3$) δ 8.51–8.31 (m, 1H), 7.93–7.78 (m, 1H), 7.74–7.64 (m, 1H), 7.63–7.57 (m, 1H), 7.52–7.42 (m, 2H), 7.43–7.32 (m, 2H), 7.28–7.22 (m, 2H), 7.10–7.01 (m, 1H), 2.68 (d, *J* = 1.2 Hz, 3H).

^{13}C NMR (101 MHz, CDCl$_3$) δ 157.41, 152.35, 140.20, 128.90, 128.54, 126.06, 125.96, 125.32, 122.67, 121.74, 121.35, 119.01, 116.71, 111.50, 23.22.

^{11}B NMR (128 MHz, CDCl$_3$) δ 28.98.

HRMS (EI): m/z calculated for $C_{17}H_{14}NOB$ [M]$^+$: 258.1199, found: 258.1199.

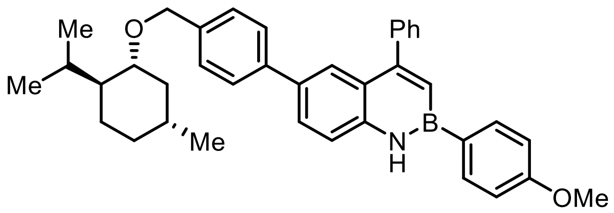

13a

6-(4-((((1*R*,2*S*,5*R*)-2-Isopropyl-5-methylcyclohexyl)oxy)methyl)phenyl)-2-(4-methoxyphenyl)-4-phenyl-1,2-dihydrobenzo[*e*][*1,2*]azaborinine (13a)

The title compound was prepared according to **GP-1**. 0.5 mmol-scale reaction, 85 mg, 43% yield, white solid.

TLC: R_f = 0.20 (10:1 pentane:EtOAc).

^{1}H NMR (400 MHz, CDCl$_3$) δ 8.12 (s, 1H), 7.92 (d, *J* = 8.6 Hz, 2H), 7.82 (s, 1H), 7.69 (dd, *J* = 8.4, 2.1 Hz, 1H), 7.53–7.48 (m, 4H), 7.48–7.43 (m, 4H), 7.37 (d, *J* = 7.9 Hz, 2H), 7.19 (d, *J* = 1.9 Hz, 1H), 7.03 (d, *J* = 8.5 Hz, 2H), 4.67 (d, *J* = 11.4 Hz, 1H), 4.41 (d, *J* = 11.4 Hz, 1H), 3.88 (s, 3H), 3.19 (td, *J* = 10.6, 4.2 Hz, 1H), 2.31 (dd, *J* = 8.0, 5.5 Hz, 1H), 2.21 (d, *J* = 12.6 Hz, 1H), 1.70–1.62 (m, 2H), 1.40–1.26 (m, 2H), 0.92 (dd, *J* = 15.7, 6.8 Hz, 9H), 0.74 (d, *J* = 6.7 Hz, 3H).

^{13}C NMR (101 MHz, CDCl$_3$) δ 161.34, 156.72, 142.87, 140.47, 140.35, 137.88, 134.43, 133.84, 129.17, 128.39, 127.61, 127.56, 127.07, 126.63, 125.02, 119.15, 114.03, 78.99, 70.29, 55.32, 48.49, 40.49, 34.74, 31.74, 25.69, 23.42, 22.53, 21.19, 16.26.

^{11}B NMR (128 MHz, CDCl$_3$) δ 33.94.

HRMS (ESI): m/z calculated for $C_{38}H_{42}NO_2BNa$ [M+Na]$^+$: 578.3207, found: 578.3205.

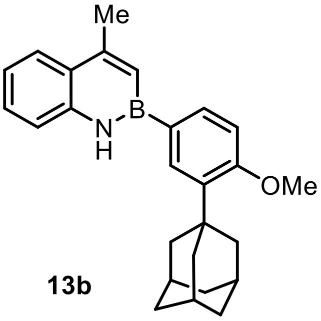

13b

2-(3-(Adamantan-1-yl)-4-methoxyphenyl)-4-methyl-1,2-dihydrobenzo[*e*][*1,2*]azaborinine (13b)

The title compound was prepared according to **GP-1**. 1 mmol-scale reaction, 250 mg, 65% yield, white solid.

TLC: R_f = 0.3 (10:1 pentane:EtOAc).

^{1}H NMR (400 MHz, CDCl$_3$) δ 7.91 (s, 1H), 7.86–7.81 (m, 1H), 7.79 (d, *J* = 1.8 Hz, 1H), 7.75 (dd, *J* = 8.0, 1.8 Hz, 1H), 7.47–7.40 (m, 1H), 7.33 (dd, *J* = 8.2, 1.3 Hz, 1H), 7.20 (ddd, *J* = 8.2, 7.0, 1.3 Hz, 1H), 7.10–7.06 (m, 1H), 6.99 (d, *J* = 8.0 Hz, 1H), 3.90 (s, 3H), 2.66 (s, 3H), 2.21 (d, *J* = 3.0 Hz, 6H), 2.12 (s, 3H), 1.82 (s, 6H).

^{13}C NMR (101 MHz, CDCl$_3$) δ 160.52, 151.22, 140.77, 138.05, 131.78, 131.02, 128.12, 125.83, 125.77, 120.75, 118.76, 111.68, 55.06, 40.81, 37.35, 37.26, 29.31, 23.21.

^{11}B NMR (128 MHz, CDCl$_3$) δ 33.88.

HRMS (EI): m/z calculated for $C_{26}H_{30}BNO$ [M]$^+$: 383.2420, found: 383.2411.

3. Reaction development

3.1 Optimisation of the substituent-rebound B-to-C single-atom swapping reaction

3.1.1 Reaction development

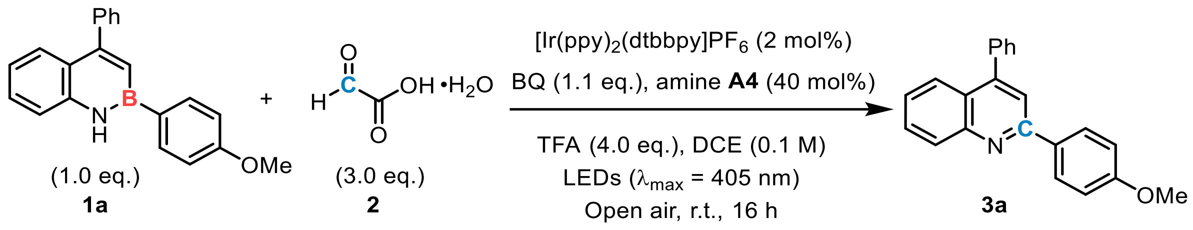

Entry	deviation from standard conditions	**1a** (%)	**3a** (%)
1	-	0	**64**
2	w/o amine	0	<5
3	w/o BQ	0	<5
4	w/o TFA	99	0
5	HOAc instead of TFA	88	<5
6	w/o $[Ir(ppy)_2(dtbbpy]PF_6$	0	45
7	w/o $[Ir(ppy)_2(dtbbpy]PF_6$, no LEDs	0	34
8	amine **A1** instead of **A4**	0	59
9	amine **A2** instead of **A4**	0	23
10	amine **A3** instead of **A4**	0	17
11	2.0 eq. of **2**	0	62
12	30 mol% of **A4**	0	55
13	50 mol% of **A4**	0	48
14	5 eq. of TFA	0	64
15	3 eq. of TFA	0	42

The yield was determined by ^{1}H NMR analysis using mesitylene as an internal standard.

Supplementary Table 1. Control experiments and optimisation of amines.

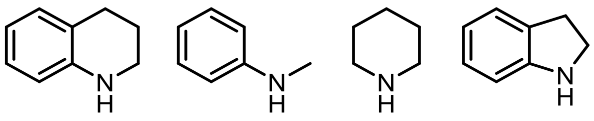

3.1.2 Optimisation of solvent

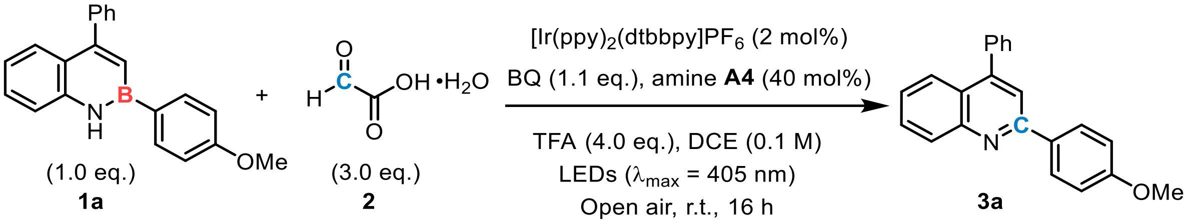

Entry	Solvent	**1a** (%)	**3a** (%)
1	Toluene	0	19
2	CH_2Cl_2	0	58
3	DCE	0	64
4	$CHCl_3$	0	33
5	*n*-Hexane	0	15
6	HFIP	0	<5
7	MeCN	45	<5
8	DMF	80	<5

The yield was determined by ^{1}H NMR analysis using mesitylene as an internal standard.

Supplementary Table 2. Optimisation of solvent.

3.1.3 Sensitivity assessment

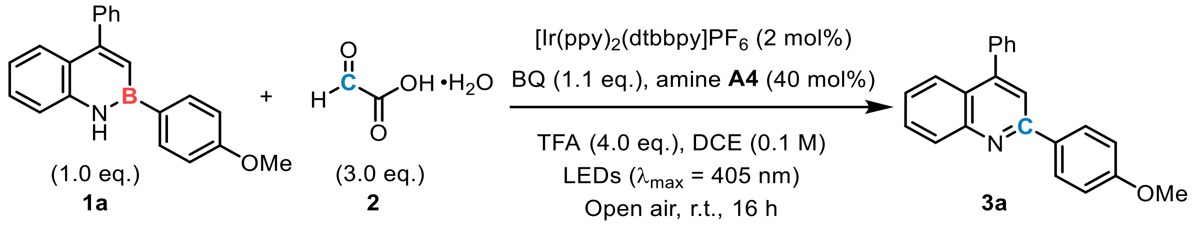

Entry	Modification	Execution	Yield (%)	Deviation (%)
1	standard	-	64	0
2	high *c*	0.2 M	60	-6
3	low *c*	0.05 M	56	-12
4	high H_2O	5 uL H_2O	29	-55
5	more TFA	5 eq.	64	0
6	less TFA	3 eq.	42	-34
7	high *T*	45 °C	61	-5
8	low *T*	15 °C	32	-50
9	low *I*	*d* = 32 cm	63	-2
10	high *I*	*d* = 2 cm	64	0
11	large scale (1 mmol)	Isolated yield	60	-6

The yield was determined by ^1H NMR analysis using mesitylene as an internal standard.

Supplementary Table 3. Sensitivity assessment.

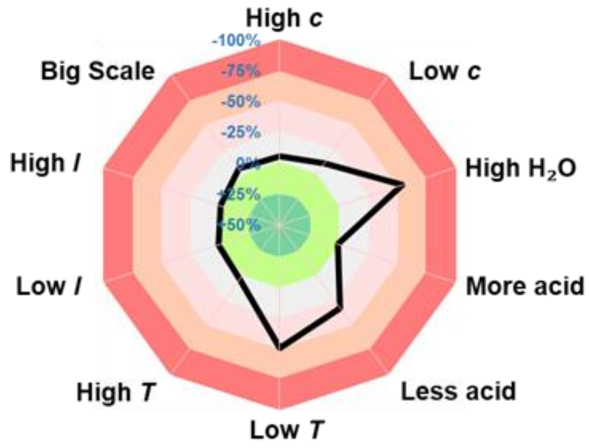

Supplementary Figure 4. Radar diagram representation of sensitivity screen[7].

3.2 Optimisation of the alkyl-substituent-rebound B-to-C single-atom swapping reaction

3.2.1 Initial attempt at the reaction

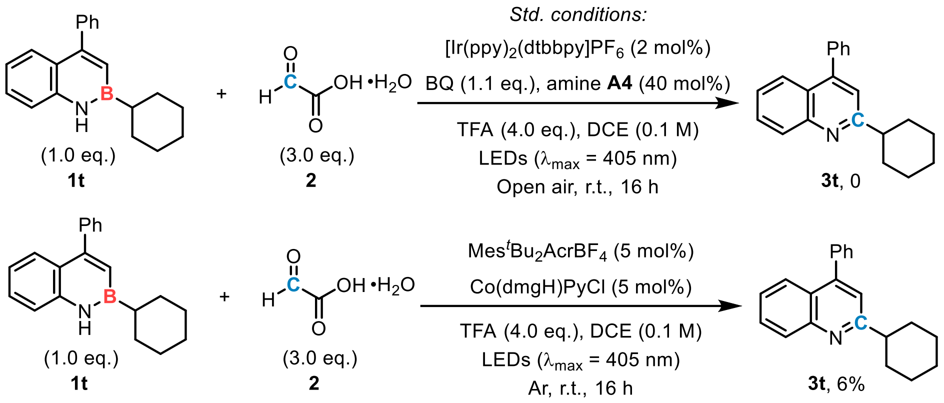

The yield was determined by ^{1}H NMR analysis using mesitylene as an internal standard.

3.2.2 Screening of the catalysts

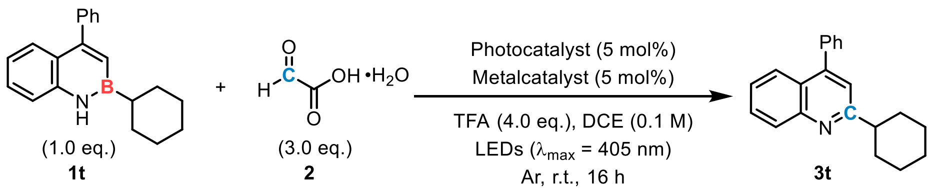

Entry	Photocatalyst	Metalcatalyst	**3t** (%)
1	$Mes^{t}Bu_2AcrBF_4$	Co(dmgH)PyCl	6
2	$Mes^{t}Bu_2AcrClO_4$	Co(dmgH)PyCl	5
3	$[Ir[dF(CF_3)ppy]_2(dtbbpy)]PF_6$	Co(dmgH)PyCl	11
4	4CzIPN	Co(dmgH)PyCl	6
5	$Mes^{t}Bu_2AcrBF_4$	-	<5
6	$[Ir[dF(CF_3)ppy]_2(dtbbpy)]PF_6$	Ni(5,5'-dmbpy)Br[b]	33 (31)[a]
7	$[Ir[dF(CF_3)ppy]_2(dtbbpy)]PF_6$	$Ni(4,4'\text{-}dtbbpy)Br_2$[b]	10
8	$[Ir[dF(CF_3)ppy]_2(dtbbpy)]PF_6$	$Ni(bpy)Br_2$[b]	14
9	$[Ir[dF(CF_3)ppy]_2(dtbbpy)]PF_6$	$Ni[5,5'\text{-}(CF_3)_2bpy]Br_2$[b]	10
10	$[Ir[dF(CF_3)ppy]_2(dtbbpy)]PF_6$	$Ni[2,9\text{-}dm\text{-}1,10\text{-}phen]Br_2$[b]	22

The yield was determined by ^{1}H NMR analysis using mesitylene as an internal standard. [a]Isolated yield. [b]10 mol%.

Supplementary Table 4. Optimisation of catalysts.

3.3 Optimisation of the B-to-C swapping reaction using aldehydes as substrates

3.3.1 Optimisation of solvent

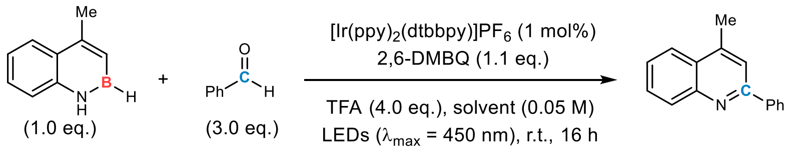

Entry	Solvent	Product (%)
1	CH_2Cl_2	54
2	DCE	55
3	Toluene	53
4	THF	0
5	CH_3CN	16
6	EtOAc	17
7	HFIP/Toluene = 1:1	54
8[a]	Toluene	62

The yields were determined by GC-FID analysis using hexadecane as internal standard. [a]Duroquinone (1.1 eq.) as oxidant reagent.

Supplementary Table 5. Optimisation of solvent.

3.3.2 Optimisation of wavelength

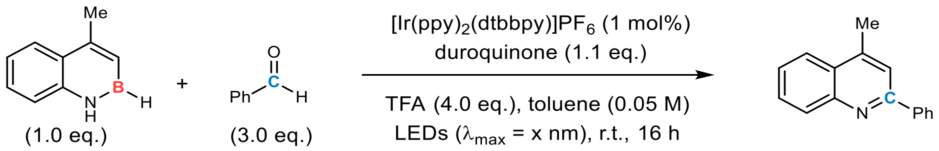

Entry	LED (λ_{max} = x nm)	Product (%)
1	450	62
2	425	67
3	405	71
4	380	59

The yields were determined by GC-FID analysis using hexadecane as internal standard.

Supplementary Table 6. Optimisation of wavelength.

3.3.3 Optimisation of stoichiometry and reaction concentration

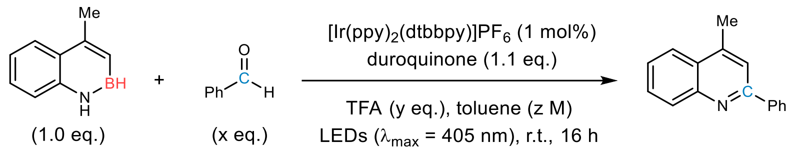

Entry	PhCHO (x eq.)	TFA (y eq.)	Toluene (z M)	Product (%)
1	2	4.0	0.05	67
2	4	4.0	0.05	66
3	3	2.6	0.05	44
4	3	4.0	0.05	71
5	3	5.0	0.05	75
6	3	7.0	0.05	84
7	3	10.0	0.05	82
8	3	13	0.05	78
9	3	7.0	0.1	88 (77)[a]
10	3	4.0	0.1	78
11	3	7.0	0.1	76
12	3	7.0	0.2	79
13	3	7.0	0.5	82

The yields were determined by GC-FID analysis using hexadecane as internal standard. [a]Isolated yield.

Supplementary Table 7. Optimisation of stoichiometry and reaction concentration.

3.4 General procedures for the B-to-C swapping reaction

General procedure A

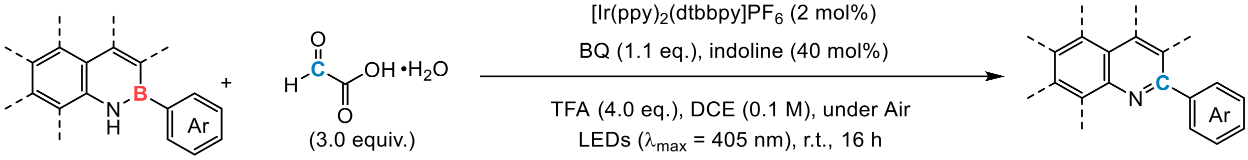

In an oven-dried 10 ml Schlenk tube equipped with a PTFE-coated rare-earth “extra power" oval stirring bar, $[Ir(ppy)_2(dtbbpy)]PF_6$ (2 mol%, 3.6 mg), benzoquinone (BQ) (23.8 mg, 0.22 mmol, 1.1 eq.), 2-oxoacetic acid hydrate (55.0 mg, 0.6 mmol, 3.0 eq.) and 1,2-benzazaborine (0.2 mmol, 1.0 eq.) were charged under air. Then 1,2-dichloroethane (2.0 mL, 0.1 M), trifluoroacetic acid (TFA) (62 µL, 0.8 mmol, 4.0 eq.), and indoline (9.0 µL, 0.08 mmol, 0.4 eq.) were added under air. The vessel was sealed with the screw cap and a needle was inserted through the cap. The reaction was then irradiated at 405 nm using the described set-up for 16 hours at room temperature. After irradiation, Na_2CO_3 aq. was slowly added into the reaction. The organic phase was further washed with brine, dried over Na_2SO_4 and concentrated under reduced pressure. Purification by flash column chromatography on SiO_2, using pentane/EtOAc mixtures, afforded the corresponding product.

General procedure B

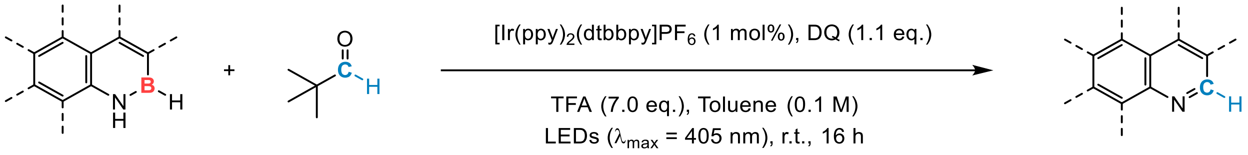

In an oven-dried 10 ml Schlenk tube equipped with a PTFE-coated rare-earth “extra power" oval stirring bar, $[Ir(ppy)_2(dtbbpy)]PF_6$ (1 mol%, 1.8 mg), duroquinone (DQ) (36.0 mg, 0.22 mmol, 1.1 eq.) and 1,2-benzazaborine (0.2 mmol, 1.0 eq.) were charged under air, then the vessel was evacuated and re-filled with argon three times. Dry toluene (2.0 mL, 0.1 M), pivalaldehyde (67 µL, 0.6 mmol, 3.0 eq.) and trifluoroacetic acid (TFA) (104 µL, 1.4 mmol, 7.0 eq.) were added under argon counter flow. The vessel was sealed with the screw cap, then irradiated at 405 nm using the described set-up for 16 hours at room temperature. After irradiation, Na_2CO_3 aq. slowly added into the reaction. The organic phase was further washed with brine, dried over Na_2SO_4 and concentrated under reduced pressure. Purification by flash column chromatography on SiO_2, using pentane/EtOAc mixtures, afforded the corresponding product.

General procedure C

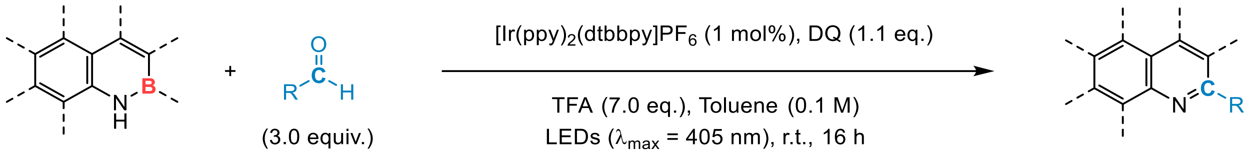

In an oven-dried 10 ml Schlenk tube equipped with a PTFE-coated rare-earth "extra power" oval stirring bar, $[Ir(ppy)_2(dtbbpy)]PF_6$ (1 mol%, 1.8 mg), duroquinone (DQ) (36.0 mg, 0.22 mmol, 1.1 eq.) and benzazaborine (0.2 mmol, 1.0 eq.) were charged under air, then the vessel was evacuated and re-filled with argon three times. Dry toluene (2.0 mL, 0.1 M), aldehyde (0.6 mmol, 3.0 eq.) and trifluoroacetic acid (TFA) (104 µL, 1.4 mmol, 7.0 eq.) were added under argon counter flow. The vessel was sealed with the screw cap, then irradiated at 405 nm using the described set-up for 16 hours at room temperature. After irradiation, Na_2CO_3 aq. slowly added into the reaction. The organic phase was further washed with brine, dried over Na_2SO_4 and concentrated under reduced pressure. Purification by flash column chromatography on SiO_2, using pentane/EtOAc mixtures, afforded the corresponding product.

4. Experimental data

4.1 Experimental data

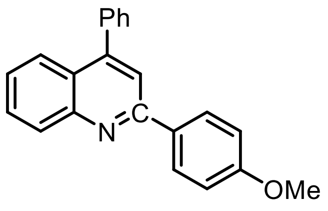

3a

2-(4-Methoxyphenyl)-4-phenylquinoline (3a)

The title compound was prepared according to the **General procedure A**. The crude residue (0.2 mmol) was purified by column chromatography to give the product as a colorless oil (38.0 mg, 61%).

Gram scale synthesis: The reaction was carried out according to the **General procedure A** at 4.0 mmol scale. In an oven-dried 150 ml Schlenk tube equipped with a PTFE-coated rare-earth "extra power" oval stirring bar, $[Ir(ppy)_2(dtbbpy)]PF_6$ (2 mol%, 73 mg), benzoquinone (BQ) (476 mg, 4.4 mmol, 1.1 eq.), 2-oxoacetic acid hydrate (1.1 g, 12 mmol, 3.0 eq.) and benzazaborine (1.24 g, 4.0 mmol, 1.0 eq.) were charged under air. Then 1,2-dichloroethane (40.0 mL, 0.1 M), trifluoroacetic acid (TFA) (1.22 mL, 16 mmol, 4.0 eq.), and indoline (180 µL, 1.6 mmol, 0.4 eq.) were added under air. The reaction was then irradiated at 405 nm using the described set-up for 16 hours at room temperature. After irradiation, Na_2CO_3 aq was slowly added into the reaction. The organic phase was further washed with brine, dried over Na_2SO_4 and concentrated under reduced pressure. Purification by flash column chromatography on SiO_2, using pentane/EtOAc mixtures (15:1), afforded the corresponding product. The product was isolated as a colorless oil (774.1 mg, 62%).

TLC: R_f = 0.35 (20:1 pentane:EtOAc).

^{1}H NMR (400 MHz, $CDCl_3$) δ 8.21 (d, *J* = 8.5 Hz, 1H), 8.18 (d, *J* = 9.0 Hz, 2H), 7.88 (d, *J* = 8.6 Hz, 1H), 7.78 (s, 1H), 7.76–7.68 (m, 1H), 7.59–7.49 (m, 5H), 7.45 (t, *J* = 7.0 Hz, 1H), 7.08–7.02 (m, 2H), 3.89 (s, 3H).

^{13}C NMR (101 MHz, $CDCl_3$) δ 160.97, 156.58, 149.13, 148.95, 138.66, 132.37, 130.04, 129.70, 129.58, 129.04, 128.71, 128.49, 126.10, 125.76, 125.65, 119.05, 114.36, 55.54.

HRMS (ESI^+): m/z calculated for $C_{22}H_{17}NONa$ $[M+Na]^+$: 334.1202, found: 334.1202.

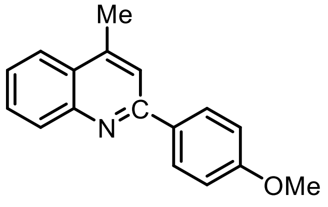

3b

2-(4-Methoxyphenyl)-4-methyl-quinoline (3b)

The title compound **3b** was also prepared according to the **General procedure A**. The crude residue (0.1 mmol) was purified by column chromatography to give the product as a colorless oil (13.5 mg, 54%).

TLC: R_f = 0.33 (20:1 pentane:EtOAc).

^{1}H NMR (400 MHz, $CDCl_3$) δ 8.20–8.05 (m, 3H), 7.97 (dd, J = 8.5, 1.4 Hz, 1H), 7.76–7.62 (m, 2H), 7.51 (ddd, J = 8.3, 6.9, 1.3 Hz, 1H), 7.11–6.92 (m, 2H), 3.88 (s, 3H), 2.75 (d, J = 1.0 Hz, 3H).

^{13}C NMR (101 MHz, $CDCl_3$) δ 160.84, 156.74, 148.27, 144.70, 132.52, 130.19, 129.37, 128.96, 127.13, 125.78, 123.71, 119.42, 114.28, 55.51, 19.14.

HRMS (ESI^+): m/z calculated for $C_{17}H_{16}NO$ $[M+H]^+$: 250.1226, found: 250.1226.

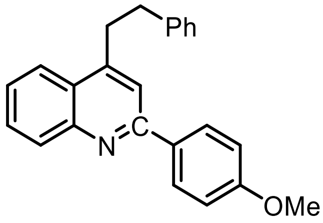

3c

2-(4-Methoxyphenyl)-4-phenethylquinoline (3c)

The title compound was prepared according to the **General procedure A**. The crude residue (0.1 mmol) was purified by column chromatography to give the product as a colorless oil (19.9 mg, 59%).

TLC: R_f = 0.35 (20:1 pentane:EtOAc).

^{1}H NMR (400 MHz, $CDCl_3$) δ 8.17 (dd, J = 8.5, 1.3 Hz, 1H), 8.11–8.01 (m, 3H), 7.71 (ddd, J = 8.4, 6.8, 1.4 Hz, 1H), 7.59 (s, 1H), 7.53 (ddd, J = 8.2, 6.8, 1.3 Hz, 1H), 7.35–7.30 (m, 2H), 7.27–7.21 (m, 3H), 7.08–6.96 (m, 2H), 3.89 (s, 3H), 3.76–3.32 (m, 2H), 3.22–2.78 (m, 2H).

^{13}C NMR (101 MHz, $CDCl_3$) δ 160.85, 156.75, 148.66, 147.80, 141.27, 132.54, 130.51, 129.33, 128.98, 128.69, 128.58, 126.44, 126.21, 125.93, 123.27, 118.61, 114.28, 55.53, 36.48, 34.61.

HRMS (ESI^+): m/z calculated for $C_{24}H_{21}NONa$ $[M+Na]^+$: 362.1515, found: 362.1515.

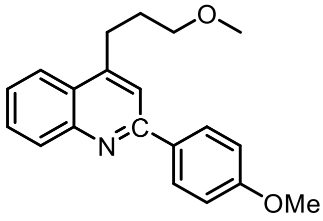

3d

2-(4-Methoxyphenyl)-4-(3-methoxypropyl)quinoline (3d)

The title compound was prepared according to the **General procedure A**. The crude residue (0.2 mmol) was purified by column chromatography to give the product as a yellow oil (20.6 mg, 34%).

TLC: R_f = 0.35 (5:1 pentane:EtOAc).

^{1}H NMR (400 MHz, $CDCl_3$) δ 8.21 (d, *J* = 8.4 Hz, 1H), 8.14 (d, *J* = 8.8 Hz, 2H), 8.04 (d, *J* = 8.4 Hz, 1H), 7.76–7.64 (m, 2H), 7.59–7.42 (m, 1H), 7.05 (d, *J* = 8.8 Hz, 2H), 3.89 (s, 3H), 3.48 (t, *J* = 6.1 Hz, 2H), 3.39 (s, 3H), 3.22 (t, *J* = 7.8 Hz, 2H), 2.18–2.00 (m, 2H).

^{13}C NMR (101 MHz, CDCl$_3$) δ 161.00, 156.62, 130.14, 129.52, 129.11, 126.41, 125.97, 123.54, 118.63, 114.36, 71.85, 58.87, 55.55, 30.28, 29.17.

HRMS (ESI$^+$): m/z calculated for $C_{20}H_{22}NO_2$ [M+H]$^+$: 308.1645, found: 308.1647.

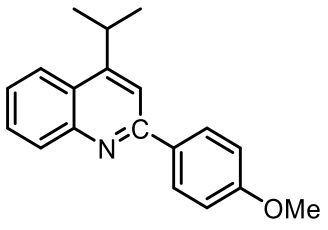

3e

4-Isopropyl-2-(4-methoxyphenyl)quinoline (3e)

The title compound was prepared according to the **General procedure A**. The crude residue (0.1 mmol) was purified by column chromatography to give the product as a colorless oil (17.7 mg, 64%).

TLC: R_f = 0.35 (20:1 pentane:EtOAc).

^{1}H NMR (400 MHz, CDCl$_3$) δ 8.17 (d, *J* = 8.4 Hz, 1H), 8.13 (dd, *J* = 8.7, 1.8 Hz, 2H), 8.08 (d, *J* = 8.5 Hz, 1H), 7.74 (s, 1H), 7.69 (ddd, *J* = 8.4, 6.8, 1.4 Hz, 1H), 7.52 (ddd, *J* = 8.3, 6.8, 1.4 Hz, 1H), 7.06 (d, *J* = 8.8 Hz, 2H), 3.89 (s, 3H), 3.78 (hept, *J* = 6.9 Hz, 1H), 1.46 (d, *J* = 6.9 Hz, 6H).

^{13}C NMR (101 MHz, CDCl$_3$) δ 160.82, 157.00, 154.89, 148.63, 132.86, 130.54, 129.12, 129.05, 125.75, 123.03, 114.58, 114.30, 55.54, 28.67, 23.15.

HRMS (ESI$^+$): m/z calculated for $C_{19}H_{20}NO$ [M+H]$^+$: 278.1539, found: 278.1541.

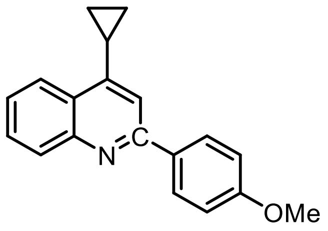

3f

4-Cyclopropyl-2-(4-methoxyphenyl)quinoline (3f)

The title compound was prepared according to the **General procedure A**. The crude residue (0.2 mmol) was purified by column chromatography to give the product as a colorless oil (18.7 mg, 34%).

TLC: R_f = 0.35 (20:1 pentane:EtOAc).

^{1}H NMR (400 MHz, CDCl$_3$) δ 8.31 (d, *J* = 8.3 Hz, 1H), 8.16 (d, *J* = 8.4 Hz, 1H), 8.10 (d, *J* = 8.9 Hz, 2H), 7.71 (t, *J* = 7.6 Hz, 1H), 7.59–7.52 (m, 1H), 7.49 (s, 1H), 7.04 (d, *J* = 8.9 Hz, 2H), 3.88 (s, 3H), 2.46 (ddd, *J* = 13.8, 8.4, 5.4 Hz, 1H), 1.28–1.13 (m, 2H), 1.01–0.84 (m, 2H).

^{13}C NMR (101 MHz, CDCl$_3$) δ 160.85, 156.96, 149.96, 148.22, 132.64, 130.09, 129.41, 129.02, 127.65, 125.81, 123.93, 115.07, 114.29, 55.54, 12.64, 7.69.

HRMS (ESI$^+$): m/z calculated for $C_{19}H_{17}NONa$ [M+Na]$^+$: 298.1202, found: 298.1200.

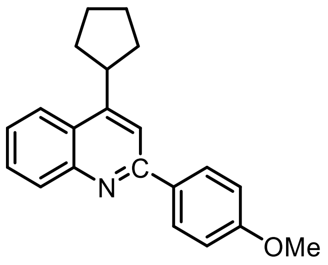

3g

4-Cyclopentyl-2-(4-methoxyphenyl)quinoline (3g)

The title compound was prepared according to the **General procedure A**. The crude residue (0.2 mmol) was purified by column chromatography to give the product as a colorless oil (34.7 mg, 57%).

TLC: R_f = 0.35 (20:1 pentane:EtOAc).

^{1}H NMR (400 MHz, CDCl$_3$) δ 8.16 (dd, *J* = 8.6, 1.3 Hz, 1H), 8.11 (dd, *J* = 10.5, 8.4 Hz, 3H), 7.74 (s, 1H), 7.69 (ddd, *J* = 8.3, 6.7, 1.4 Hz, 1H), 7.57–7.43 (m, 1H), 7.05 (d, *J* = 8.7 Hz, 2H), 3.89 (s, 3H), 3.85–3.75 (m, 1H), 2.35–2.15 (m, 2H), 1.97–1.80 (m, 6H).

^{13}C NMR (101 MHz, CDCl$_3$) δ 160.77, 156.93, 152.67, 148.66, 132.96, 130.41, 129.08, 129.01, 126.63, 125.59, 123.67, 114.78, 114.28, 55.52, 40.94, 33.49, 25.61.

HRMS (ESI$^+$): m/z calculated for $C_{21}H_{21}NONa$ [M+Na]$^+$: 326.1515, found: 326.1515.

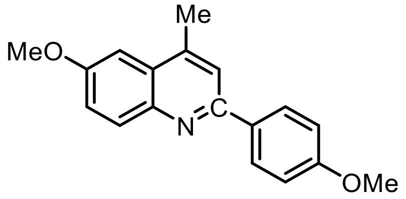

3h/12a

6-Methoxy-2-(4-methoxyphenyl)-4-methylquinoline (3h/12a)

The title compound was prepared according to the **General procedure A**. The crude residue (0.1 mmol) was purified by column chromatography to give the product as a yellow solid (18.9 mg, 68%).

TLC: R_f = 0.25 (10:1 pentane:EtOAc).

^{1}H NMR (400 MHz, CDCl$_3$) δ 8.16–8.00 (m, 3H), 7.63 (d, J = 1.3 Hz, 1H), 7.41–7.32 (m, 1H), 7.18 (d, J = 2.8 Hz, 1H), 7.07–6.95 (m, 2H), 3.96 (s, 3H), 3.88 (s, 3H), 2.70 (s, 3H).

^{13}C NMR (101 MHz, CDCl$_3$) δ 160.59, 157.45, 154.44, 144.01, 143.39, 132.47, 131.54, 128.69, 127.91, 121.49, 119.69, 114.26, 102.10, 55.65, 55.50, 19.37.

HRMS (ESI$^+$): m/z calculated for $C_{18}H_{17}NO_2Na$ [M+Na]$^+$: 302.1152, found: 302.1150.

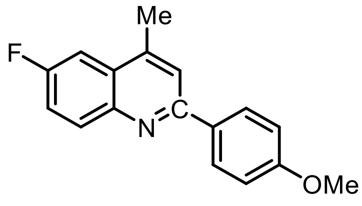

3i

6-Fluoro-2-(4-methoxyphenyl)-4-methylquinoline (3i)

The title compound was prepared according to the **General procedure A**. The crude residue (0.1 mmol) was purified by column chromatography to give the product as a white solid (12.6 mg, 47%).

TLC: R_f = 0.35 (15:1 pentane:EtOAc).

^{1}H NMR (400 MHz, CDCl$_3$) δ 8.18–8.07 (m, 3H), 7.69 (t, J = 1.1 Hz, 1H), 7.56 (dd, J = 9.8, 2.8 Hz, 1H), 7.51–7.42 (m, 1H), 7.09–6.96 (m, 2H), 3.89 (s, 3H), 2.70 (d, J = 1.1 Hz, 3H).

^{13}C NMR (101 MHz, CDCl$_3$) δ 160.95, 160.28 (d, *J* = 246.9 Hz), 156.09 (d, *J* = 2.7 Hz), 145.18, 144.34, 132.42 (d, *J* = 9.1 Hz), 132.01, 128.91, 127.80 (d, *J* = 9.3 Hz), 119.98, 119.42 (d, *J* = 25.4 Hz), 114.35, 107.40 (d, *J* = 22.1 Hz), 55.54, 19.22.

^{19}F NMR (376 MHz, CDCl$_3$) δ -113.82.

HRMS (ESI$^+$): m/z calculated for $C_{17}H_{14}NOFNa$ [M+Na]$^+$: 290.0963, found: 290.0951.

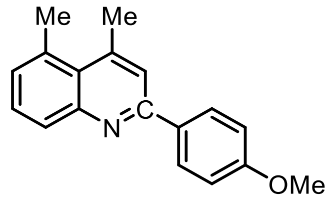

3j

2-(4-Methoxyphenyl)-4,5-dimethylquinoline (3j)

The title compound was prepared according to the **General procedure A**. The crude residue (0.2 mmol) was purified by column chromatography to give the product as a white solid (27.0 mg, 51%).

TLC: R_f = 0.35 (15:1 pentane:EtOAc).

^{1}H NMR (400 MHz, CDCl$_3$) δ 8.16 – 8.11 (m, 2H), 8.02 (d, *J* = 8.4 Hz, 1H), 7.58 (d, *J* = 1.1 Hz, 1H), 7.51 (dd, *J* = 8.4, 7.0 Hz, 1H), 7.25 (d, *J* = 5.5 Hz, 1H), 7.08 – 7.01 (m, 2H), 3.88 (s, 3H), 2.97 (s, 3H), 2.91 (s, 3H).

^{13}C NMR (101 MHz, CDCl$_3$) δ 160.90, 155.74, 148.56, 146.18, 135.44, 129.29 – 129.15 (m), 128.89, 128.76, 127.41, 121.43, 114.30, 55.54, 25.79, 25.63.

HRMS (ESI$^+$): m/z calculated for $C_{18}H_{17}NONa$ [M+Na]$^+$: 286.1202, found: 286.1201.

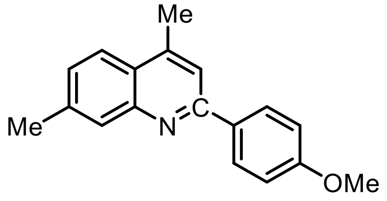

3k

2-(4-Methoxyphenyl)-4,7-dimethylquinoline (3k)

The title compound was prepared according to the **General procedure A**. The crude residue (0.2 mmol) was purified by column chromatography to give the product as a yellow oil (25.4 mg, 48%).

TLC: R_f = 0.35 (15:1 pentane:EtOAc).

^{1}H NMR (400 MHz, CDCl$_3$) δ 8.15 – 8.08 (m, 2H), 7.95 (s, 1H), 7.86 (d, *J* = 8.4 Hz, 1H), 7.60 (d, *J* = 1.1 Hz, 1H), 7.35 (dd, *J* = 8.5, 1.8 Hz, 1H), 7.08 – 7.00 (m, 2H), 3.88 (s, 3H), 2.72 (s, 3H), 2.57 (s, 3H).

^{13}C NMR (101 MHz, CDCl$_3$) δ 160.82, 156.66, 148.36, 144.63, 139.60, 129.15, 128.95, 128.01, 125.13, 123.43, 118.71, 114.26, 55.51, 21.83, 19.10.

HRMS (ESI$^+$): m/z calculated for $C_{17}H_{14}NOFNa$ [M+Na]$^+$: 286.1202, found: 286.1199.

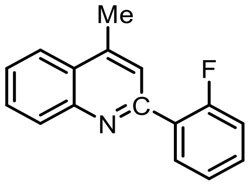

3l

2-(2-Fluorophenyl)-4-methylquinoline (3l)

The title compound was prepared according to the **General procedure A**. The crude residue (0.2 mmol) was purified by column chromatography to give the product as a colorless oil (26.4 mg, 56%).

TLC: R_f = 0.50 (30:1 pentane:EtOAc).

^{1}H NMR (400 MHz, CDCl$_3$) δ 8.19 (d, *J* = 8.5 Hz, 1H), 8.10–8.00 (m, 2H), 7.77–7.70 (m, 2H), 7.58 (ddd, *J* = 8.3, 6.9, 1.3 Hz, 1H), 7.47–7.39 (m, 1H), 7.31 (td, *J* = 7.5, 1.3 Hz, 1H), 7.20 (ddd, *J* = 11.1, 8.2, 1.3 Hz, 1H), 2.77 (s, 3H).

^{13}C NMR (101 MHz, CDCl$_3$) δ 160.83 (d, *J* = 249.5 Hz), 153.95, 148.20, 144.53, 131.66 (d, *J* = 3.1 Hz), 130.83 (d, *J* = 8.5 Hz), 130.36, 129.48, 128.28, 127.43, 126.54, 124.80 (d, *J* = 3.6 Hz), 123.81, 123.27 (d, *J* = 7.5 Hz), 116.32 (d, *J* = 22.8 Hz), 19.10.

^{19}F NMR (376 MHz, CDCl$_3$) δ -117.12.

HRMS (ESI$^+$): m/z calculated for $C_{16}H_{12}FNNa$ [M+Na]$^+$: 260.0846, found: 260.0846.

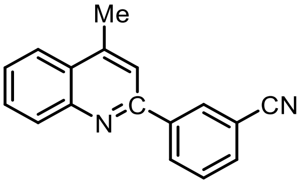

3m

3-(4-Methylquinolin-2-yl)benzonitrile (3m)

The title compound **3m** was prepared according to the **General procedure A**. The crude residue (0.2 mmol) was purified by column chromatography to give the product as a yellow oil (19.8 mg, 41%).

TLC: R_f = 0.30 (5:1 pentane:EtOAc).

^{1}H NMR (400 MHz, CDCl$_3$) δ 8.48 (d, *J* = 1.9 Hz, 1H), 8.38 (dd, *J* = 7.8, 1.5 Hz, 1H), 8.16 (dd, *J* = 8.4, 1.2 Hz, 1H), 8.01 (d, *J* = 8.4 Hz, 1H), 7.78–7.70 (m, 2H), 7.69–7.67 (m, 1H), 7.64–7.56 (m, 2H), 2.78 (s, 3H).

^{13}C NMR (101 MHz, CDCl$_3$) δ 154.46, 148.13, 145.79, 140.99, 132.51, 131.75, 131.34, 130.46, 129.93, 129.69, 127.64, 126.87, 123.84, 119.20, 118.96, 113.12, 19.21.

HRMS (ESI$^+$): m/z calculated for $C_{17}H_{13}N_2$ [M+H]$^+$: 245.1073, found: 245.1073.

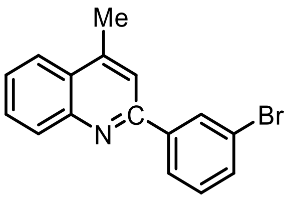

3n

2-(3-Bromophenyl)-4-methylquinoline (3n)

The title compound was prepared according to the **General procedure A**. The crude residue (0.2 mmol) was purified by column chromatography to give the product as a colorless oil (27.6 mg, 46%).

TLC: R_f = 0.40 (20:1 pentane:EtOAc).

^{1}H NMR (400 MHz, CDCl$_3$) δ 8.34 (s, 1H), 8.17 (d, *J* = 7.7 Hz, 1H), 8.07 (d, *J* = 7.9 Hz, 1H), 8.00 (d, *J* = 8.3 Hz, 1H), 7.73 (t, *J* = 7.7 Hz, 1H), 7.67 (s, 1H), 7.56 (t, *J* = 7.6 Hz, 2H), 7.38 (t, *J* = 7.8 Hz, 1H), 2.77 (s, 3H).

^{13}C NMR (101 MHz, CDCl$_3$) δ 155.46, 148.16, 145.32, 141.96, 132.21, 130.71, 130.46, 130.40, 129.68, 127.54, 126.52, 126.16, 123.78, 123.21, 119.56, 19.18.

HRMS (ESI$^+$): m/z calculated for $C_{16}H_{12}BrNNa$ [M+Na]$^+$: 320.0045, found: 320.0046.

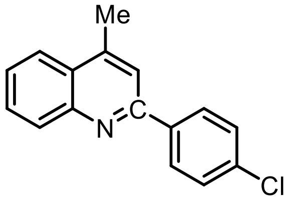

3o

2-(4-Chlorophenyl)-4-methylquinoline (3o)

The title compound **3o** was prepared according to the **General procedure A**. The crude residue (0.2 mmol) was purified by column chromatography to give the product as a white solid (26.2 mg, 52%).

TLC: R_f = 0.40 (20:1 pentane:EtOAc).

^{1}H NMR (400 MHz, CDCl$_3$) δ 8.16 (d, *J* = 6.6 Hz, 1H), 8.13–8.06 (m, 2H), 7.98 (d, *J* = 8.3 Hz, 1H), 7.72 (ddd, *J* = 8.4, 6.9, 1.4 Hz, 1H), 7.65 (s, 1H), 7.55 (ddd, *J* = 8.2, 6.8, 1.3 Hz, 1H), 7.52–7.42 (m, 2H), 2.75 (s, 3H).

^{13}C NMR (101 MHz, CDCl$_3$) δ 155.84, 148.14, 145.30, 138.26, 135.55, 130.33, 129.68, 129.09, 128.94, 127.43, 126.40, 123.79, 119.48, 19.20.

HRMS (ESI$^+$): m/z calculated for $C_{16}H_{13}ClN$ [M+H]$^+$: 254.0731, found: 254.0731.

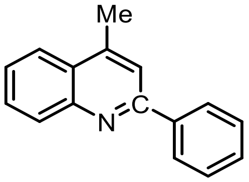

3p

4-Methyl-2-phenylquinoline (3p)

The title compound **3p** was prepared according to the **General procedure A**. The crude residue (0.2 mmol) was purified by column chromatography to give the product as a yellow oil (19.7 mg, 45%).

TLC: R_f = 0.30 (20:1 pentane:EtOAc).

^{1}H NMR (400 MHz, CDCl$_3$) δ 8.23–8.11 (m, 3H), 8.00 (dd, *J* = 8.4, 1.4 Hz, 1H), 7.76–7.67 (m, 2H), 7.59–7.50 (m, 3H), 7.49–7.40 (m, 1H), 2.77 (d, *J* = 1.0 Hz, 3H).

^{13}C NMR (101 MHz, CDCl$_3$) δ 157.23, 148.27, 144.93, 139.97, 130.43, 129.45, 129.31, 128.91, 127.67, 127.39, 126.15, 123.74, 119.91, 19.15.

HRMS (ESI$^+$): m/z calculated for $C_{16}H_{13}NNa$ [M+Na]$^+$: 242.0940, found: 242.0937.

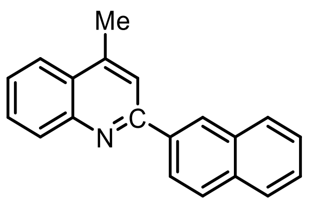

3q

4-Methyl-2-(naphthalen-2-yl)quinoline (3q)

The title compound **3q** was prepared according to the **General procedure A**. The crude residue (0.2 mmol) was purified by column chromatography to give the product as a white solid (29.2 mg, 54%).

TLC: R_f = 0.40 (30:1 pentane:EtOAc).

^{1}H NMR (400 MHz, CDCl$_3$) δ 8.62 (s, 1H), 8.38 (dd, *J* = 8.6, 1.8 Hz, 1H), 8.24 (d, *J* = 1.1 Hz, 1H), 8.04–7.97 (m, 3H), 7.94–7.88 (m, 1H), 7.86 (s, 1H), 7.75 (t, *J* = 6.9 Hz, 1H), 7.61–7.49 (m, 3H), 2.78 (s, 3H).

^{13}C NMR (101 MHz, CDCl$_3$) δ 156.93, 148.31, 144.94, 137.19, 133.92, 133.61, 130.39, 129.49, 128.91, 128.59, 127.82, 127.41, 127.14, 126.72, 126.38, 126.18, 125.21, 123.77, 119.97, 19.16.

HRMS (ESI$^+$): m/z calculated for $C_{20}H_{16}N$ [M+H]$^+$: 270.1277, found: 270.1278.

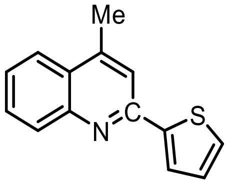

3r

4-Methyl-2-(thiophen-2-yl)quinoline (3r)

The title compound was prepared according to the **General procedure A**. The crude residue (0.2 mmol) was purified by column chromatography to give the product as a colorless oil (17.5 mg, 39%).

TLC: R_f = 0.40 (30:1 pentane:EtOAc).

^{1}H NMR (400 MHz, CDCl$_3$) δ 8.09 (d, *J* = 8.5 Hz, 1H), 7.94 (d, *J* = 8.4 Hz, 1H), 7.73 (dd, *J* = 3.7, 1.2 Hz, 1H), 7.68 (ddd, *J* = 8.4, 6.9, 1.4 Hz, 1H), 7.64 (d, *J* = 1.0 Hz, 1H), 7.50 (ddd, *J* = 8.2, 6.8, 1.3 Hz, 1H), 7.46 (dd, *J* = 5.0, 1.2 Hz, 1H), 7.16 (dd, *J* = 5.1, 3.7 Hz, 1H), 2.73 (d, *J* = 1.1 Hz, 3H).

^{13}C NMR (101 MHz, CDCl$_3$) δ 152.14, 148.09, 145.65, 144.82, 129.96, 129.59, 128.47, 128.14, 127.47, 125.98, 125.77, 123.75, 118.41, 19.07.

HRMS (ESI$^+$): m/z calculated for $C_{14}H_{11}NSNa$ [M+Na]$^+$: 248.0504, found: 248.0504.

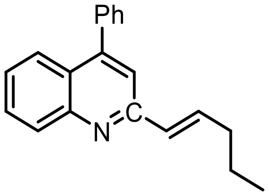

3s

(*E*)-2-(pent-1-en-1-yl)-4-phenylquinoline (3s)

The title compound was prepared according to the **General procedure A**. The crude residue (0.1 mmol) was purified by column chromatography to give the product as a yellow oil (9.0 mg, 33%).

TLC: R_f = 0.50 (30:1 pentane:EtOAc).

^{1}H NMR (400 MHz, CDCl$_3$) δ 8.08 (s, 1H), 7.76 (dd, *J* = 8.4, 1.4 Hz, 1H), 7.62 (t, *J* = 7.7 Hz, 1H), 7.48–7.40 (m, 6H), 7.36 (t, *J* = 7.7 Hz, 1H), 6.85–6.67 (m, 2H), 2.34–2.18 (m, 2H), 1.60–1.42 (m, 2H), 0.92 (t, *J* = 7.4 Hz, 3H).

^{13}C NMR (101 MHz, CDCl$_3$) δ 155.92, 138.37, 130.97, 129.62, 128.91, 128.71, 128.57, 126.77, 126.21, 125.92, 125.82, 119.03, 118.46, 116.27, 115.71, 35.34, 22.23, 13.98.

HRMS (ESI$^+$): m/z calculated for $C_{20}H_{19}NNa$ [M+Na]$^+$: 296.1410, found: 296.1409.

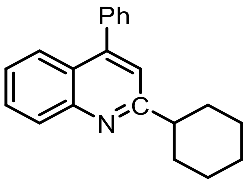

3t

2-Cyclohexyl-4-phenylquinoline (3t)

The title compound was prepared according to the following procedure:

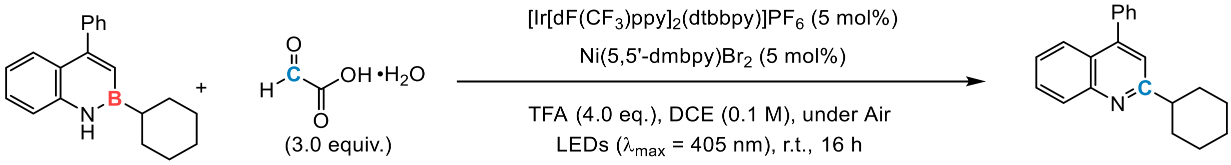

In an oven-dried 10 ml Schlenk tube equipped with a PTFE-coated rare-earth “extra power" oval stirring bar, [Ir[dF(CF$_3$)ppy]$_2$(dtbbpy)]PF$_6$ ([Ir-F], 5 mol%, 10.5 mg), Ni(5,5'-dmbpy)Br$_2$ (5 mol%, 8 mg), 2-oxoacetic acid hydrate (55.0 mg, 0.6 mmol, 3.0 eq.) and 1,2-benzazaborine (0.2 mmol, 1.0 eq.) were charged under air. The tube was evacuated and refilled with argon three times. Then 1,2-dichloroethane (2.0 mL, 0.1 M), trifluoroacetic acid (TFA) (62 µL, 0.8 mmol, 4.0 eq.) were added under argon counter flow. The reaction was then irradiated at 405 nm using the described set-up for 16 hours at room temperature. After irradiation, Na_2CO_3 aq was slowly added into the reaction. The organic phase was further washed with brine, dried over Na_2SO_4 and concentrated under reduced pressure. Purification by flash column chromatography on SiO_2, using pentane/EtOAc mixtures, afforded the corresponding product. The crude residue (0.2 mmol) was purified by column chromatography to give the product as a yellow oil (17.8 mg, 31%).

TLC: R_f = 0.50 (20:1 pentane:EtOAc).

^{1}H NMR (400 MHz, CDCl$_3$) δ 8.11 (d, *J* = 8.4 Hz, 1H), 7.92–7.79 (m, 1H), 7.68 (ddd, *J* = 8.4, 6.7, 1.4 Hz, 1H), 7.51 (d, *J* = 4.3 Hz, 5H), 7.43 (ddd, *J* = 8.3, 6.8, 1.3 Hz, 1H), 7.27 (s, 1H), 2.96 (tt, *J* = 12.0, 3.4 Hz, 1H), 2.07 (d, *J* = 12.8 Hz, 2H), 1.90 (d, *J* = 13.0 Hz, 2H), 1.79 (d, *J* = 13.0 Hz, 1H), 1.67 (qd, *J* = 12.6, 3.3 Hz, 2H), 1.47 (tt, *J* = 12.6, 3.3 Hz, 2H), 1.35 (tt, *J* = 12.7, 3.4 Hz, 1H).

^{13}C NMR (101 MHz, CDCl$_3$) δ 166.54, 148.77, 148.47, 138.74, 129.73, 129.54, 129.26, 128.65, 128.38, 125.81, 125.75, 125.69, 120.01, 47.85, 33.06, 26.72, 26.27.

HRMS (ESI$^+$): m/z calculated for $C_{21}H_{21}NNa$ [M+Na]$^+$: 310.1566, found: 310.1566.

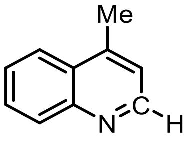

3u

4-Methylquinoline (3u)

The title compound was prepared according to the **General procedure B**. The crude residue (0.2 mmol) was purified by column chromatography to give the product as a yellow oil (18.0 mg, 63%).

TLC: R_f = 0.45 (5:1 pentane:EtOAc).

^{1}H NMR (400 MHz, CDCl$_3$) δ 8.76 (d, *J* = 4.3 Hz, 1H), 8.10 (dd, *J* = 8.4, 1.3 Hz, 1H), 8.01–7.91 (m, 1H), 7.69 (ddd, *J* = 8.4, 6.8, 1.5 Hz, 1H), 7.59–7.49 (m, 1H), 7.24–7.07 (m, 1H), 2.67 (s, 3H).

^{13}C NMR (101 MHz, CDCl$_3$) δ 150.24, 148.03, 144.38, 130.06, 129.18, 128.34, 126.35, 123.89, 121.93, 18.72.

HRMS (ESI$^+$): m/z calculated for $C_{10}H_9NNa$ [M+Na]$^+$: 166.0627, found: 166.0627.

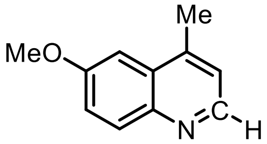

3v

6-Methoxy-4-methylquinoline (3v)

The title compound was prepared according to the **General procedure B**. The crude residue (0.2 mmol) was purified by column chromatography to give the product as a yellow oil (24.6 mg, 71%).

TLC: R_f = 0.30 (1:1 pentane:EtOAc).

^{1}H NMR (400 MHz, CDCl$_3$) δ 8.63 (d, *J* = 4.4 Hz, 1H), 8.00 (d, *J* = 9.2 Hz, 1H), 7.35 (dd, *J* = 9.2, 2.8 Hz, 1H), 7.23–7.11 (m, 2H), 3.94 (s, 3H), 2.64 (d, *J* = 1.0 Hz, 3H).

^{13}C NMR (101 MHz, CDCl$_3$) δ 157.73, 147.79, 144.09, 142.84, 131.56, 129.28, 122.22, 121.51, 101.99, 55.62, 18.96.

HRMS (ESI$^+$): m/z calculated for $C_{11}H_{11}NONa$ [M+Na]$^+$: 196.0733, found: 196.0733.

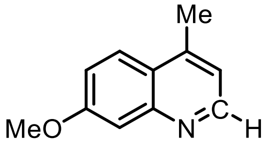

3w

7-Methoxy-4-methylquinoline (3w)

The title compound was prepared according to the **General procedure B**. The crude residue (0.2 mmol) was purified by column chromatography to give the product as a yellow oil (15.9 mg, 46%).

TLC: R_f = 0.30 (1:1 pentane:EtOAc).

^{1}H NMR (400 MHz, CDCl$_3$) δ 8.69 (d, *J* = 4.4 Hz, 1H), 7.89 (d, *J* = 9.2 Hz, 1H), 7.44 (d, *J* = 2.6 Hz, 1H), 7.22 (dd, *J* = 9.2, 2.6 Hz, 1H), 7.13–7.05 (m, 1H), 3.96 (s, 3H), 2.67 (d, *J* = 1.0 Hz, 3H).

^{13}C NMR (101 MHz, $CDCl_3$) δ 160.59, 150.34, 149.68, 144.64, 125.13, 123.52, 120.22, 119.48, 107.80, 55.66, 18.80.

HRMS (ESI$^+$): m/z calculated for $C_{11}H_{11}NONa$ [M+Na]$^+$: 196.0733, found: 196.0733.

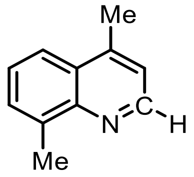

3x

4,8-Dimethylquinoline (3x)

The title compound was prepared according to the **General procedure B**. The crude residue (0.2 mmol) was purified by column chromatography to give the product as a white solid (17.2 mg, 55%).

TLC: R_f = 0.50 (4:1 pentane:EtOAc).

^{1}H NMR (400 MHz, $CDCl_3$) δ 8.68 (d, J = 4.4 Hz, 1H), 7.95 (dd, J = 8.4, 1.4 Hz, 1H), 7.52 (dd, J = 8.4, 7.0 Hz, 1H), 7.30 (dt, J = 7.0, 1.2 Hz, 1H), 7.14 (dd, J = 4.4, 1.2 Hz, 1H), 2.95–2.88 (m, 6H).

^{13}C NMR (101 MHz, $CDCl_3$) δ 150.12, 149.57, 145.68, 135.70, 129.64, 129.27, 128.79, 128.57, 124.18, 25.71, 25.44.

HRMS (ESI$^+$): m/z calculated for $C_{11}H_{11}NNa$ [M+Na]$^+$: 180.0784, found: 180.0783.

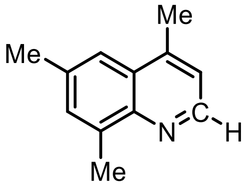

3y

4,6,8-Trimethylquinoline (3y)

The title compound was prepared according to the **General procedure B**. The crude residue (0.2 mmol) was purified by column chromatography to give the product as a yellow solid (22.6 mg, 66%).

TLC: R_f = 0.30 (9:1 pentane:EtOAc).

^{1}H NMR (400 MHz, $CDCl_3$) δ 8.73 (d, J = 4.4 Hz, 1H), 7.67–7.54 (m, 1H), 7.40 (s, 1H), 7.18 (dd, J = 4.4, 1.0 Hz, 1H), 2.79 (s, 3H), 2.66 (d, J = 1.0 Hz, 3H), 2.51 (d, J = 1.0 Hz, 3H).

^{13}C NMR (101 MHz, $CDCl_3$) δ 148.13, 145.79, 143.69, 137.26, 135.70, 131.75, 128.37, 121.88, 120.87, 21.98, 19.10, 18.60.

HRMS (ESI$^+$): m/z calculated for $C_{12}H_{14}N$ [M+H]$^+$: 172.1121, found: 172.1120.

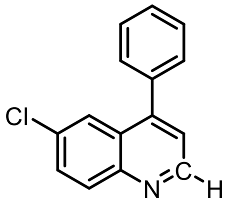

3z

6-Chloro-4-phenylquinoline (3z)

The title compound was prepared according to the **General procedure B**. The crude residue (0.1 mmol) was purified by column chromatography to give the product as a yellow oil (18.3 mg, 76%).

TLC: R_f = 0.30 (4:1 pentane:EtOAc).

^{1}H NMR (400 MHz, CDCl$_3$) δ 8.93 (d, *J* = 4.4 Hz, 1H), 8.13 (d, *J* = 9.0 Hz, 1H), 7.89 (d, *J* = 2.3 Hz, 1H), 7.67 (dd, *J* = 9.0, 2.3 Hz, 1H), 7.60–7.51 (m, 3H), 7.48 (dd, *J* = 7.7, 1.8 Hz, 2H), 7.36 (d, *J* = 4.4 Hz, 1H).

^{13}C NMR (101 MHz, CDCl$_3$) δ 150.14, 148.07, 147.04, 137.39, 132.83, 131.52, 130.51, 129.54, 128.95, 128.91, 127.62, 124.82, 122.19.

HRMS (ESI$^+$): m/z calculated for $C_{15}H_{11}NCl$ [M+H]$^+$: 240.0575, found: 240.0574.

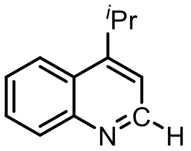

3aa

4-Isopropylquinoline (3aa)

The title compound was prepared according to the **General procedure B**. The crude residue (0.2 mmol) was purified by column chromatography to give the product as a yellow oil (26.6 mg, 77%).

TLC: R_f = 0.30 (9:1 pentane:EtOAc).

^{1}H NMR (400 MHz, CDCl$_3$) δ 8.85 (d, *J* = 4.6 Hz, 1H), 8.11 (td, *J* = 8.8, 1.4 Hz, 2H), 7.69 (ddd, *J* = 8.4, 6.8, 1.4 Hz, 1H), 7.55 (ddd, *J* = 8.4, 6.8, 1.4 Hz, 1H), 7.30 (d, *J* = 4.6 Hz, 1H), 3.86–3.63 (m, 1H), 1.40 (d, *J* = 6.8 Hz, 6H).

^{13}C NMR (101 MHz, CDCl$_3$) δ 154.55, 150.55, 148.47, 130.49, 128.94, 127.05, 126.32, 123.21, 117.05, 28.43, 23.05.

HRMS (ESI$^+$): m/z calculated for $C_{12}H_{13}NNa$ [M+Na]$^+$: 194.0940, found: 194.0939.

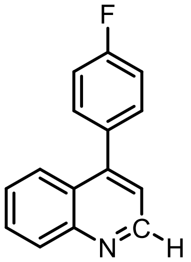

3ab

4-(4-Fluorophenyl)quinoline (3ab)

The title compound was prepared according to the **General procedure B**. The crude residue (0.1 mmol) was purified by column chromatography to give the product as a yellow oil (15.7 mg, 70%).

TLC: R_f = 0.30 (4:1 pentane:EtOAc).

^{1}H NMR (400 MHz, CDCl$_3$) δ 8.94 (d, *J* = 4.4 Hz, 1H), 8.19 (dt, *J* = 8.5, 1.0 Hz, 1H), 7.98–7.83 (m, 1H), 7.74 (ddd, *J* = 8.4, 6.8, 1.4 Hz, 1H), 7.56–7.41 (m, 3H), 7.31 (d, *J* = 4.4 Hz, 1H), 7.25–7.19 (m, 2H).

^{13}C NMR (101 MHz, CDCl$_3$) δ 163.05 (d, *J* = 248.3 Hz), 149.97, 148.68, 147.64, 134.01 (d, *J* = 3.4 Hz), 131.36 (d, *J* = 8.2 Hz), 129.97, 129.61, 126.95, 126.84, 125.71, 121.50, 115.80 (d, *J* = 21.6 Hz).

^{19}F NMR (376 MHz, CDCl$_3$) δ -113.18.

HRMS (ESI$^+$): m/z calculated for $C_{15}H_{11}NF$ $[M+H]^+$: 224.0870, found: 224.0870.

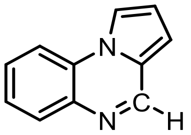

3ac

Pyrrolo[*1,2-a*]quinoxaline (3ac)

The title compound was prepared according to the **General procedure B**. The crude residue (0.1 mmol) was purified by column chromatography to give the product as a pink solid (10.9 mg, 65%).

TLC: R_f = 0.30 (4:1 pentane:EtOAc).

^{1}H NMR (400 MHz, CDCl$_3$) δ 8.80 (s, 1H), 7.96 (dd, *J* = 8.0, 1.6 Hz, 1H), 7.91 (dt, *J* = 2.6, 1.1 Hz, 1H), 7.84 (dd, *J* = 8.2, 1.5 Hz, 1H), 7.51 (ddd, *J* = 8.3, 7.2, 1.6 Hz, 1H), 7.43 (td, *J* = 7.7, 1.4 Hz, 1H), 6.94–6.81 (m, 2H).

^{13}C NMR (101 MHz, CDCl$_3$) δ 145.80, 135.78, 130.13, 128.06, 127.91, 126.50, 125.28, 114.35, 114.15, 113.88, 107.51.

HRMS (ESI$^+$): m/z calculated for $C_{11}H_9N_2$ $[M+H]^+$: 169.0760, found: 169.0760.

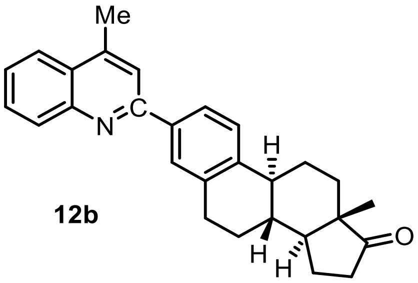

12b

(8*R*,9*S*,13*S*,14*S*)-13-Methyl-3-(4-methylquinolin-2-yl)-6,7,8,9,11,12,13,14,15,16-decahydro-17*H*-cyclopenta[*a*]phenanthren-17-one (12b)

The title compound was prepared according to the **General procedure A**. The crude residue (0.2 mmol) was purified by column chromatography to give the product as a colorless oil (38.3 mg, 48%).

TLC: R_f = 0.20 (5:1 pentane:EtOAc).

^{1}H NMR (400 MHz, CDCl$_3$) δ 8.21 (d, *J* = 9.1 Hz, 1H), 8.00 (d, *J* = 10.0 Hz, 1H), 7.94 (s, 1H), 7.91 (d, *J* = 8.1 Hz, 1H), 7.72 (qd, *J* = 5.9, 1.5 Hz, 2H), 7.54 (t, *J* = 7.6 Hz, 1H), 7.44 (d, *J* = 8.1 Hz, 1H), 3.06 (dt, *J* = 11.1, 4.9 Hz, 2H), 2.77 (s, 3H), 2.58–2.47 (m, 2H), 2.42–2.35 (m, 1H), 2.24–2.14 (m, 1H), 2.09 (ddd, *J* = 12.6, 6.0, 2.0 Hz, 2H), 2.03–1.97 (m, 1H), 1.71–1.50 (m, 6H), 0.94 (s, 3H).

^{13}C NMR (101 MHz, CDCl$_3$) δ 221.07, 157.01, 141.33, 137.17, 130.08, 129.56, 128.25, 127.33, 126.55, 126.11, 125.94, 125.03, 123.76, 119.84, 115.56, 50.70, 48.14, 44.65, 38.25, 36.01, 31.74, 29.65, 26.65, 25.86, 21.75, 19.21, 14.01.

HRMS (ESI$^+$): m/z calculated for $C_{28}H_{30}NO$ [M+H]$^+$: 396.2322, found: 396.2321.

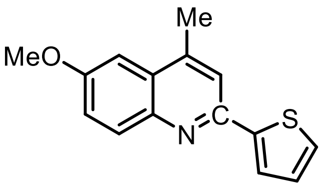

12c

6-Methoxy-4-methyl-2-(thiophen-2-yl)quinoline (12c)

The title compound was prepared according to the **General procedure A**. The crude residue (0.1 mmol) was purified by column chromatography to give the product as a yellow solid (11.7 mg, 46%).

TLC: R_f = 0.30 (10:1 pentane:EtOAc).

^{1}H NMR (400 MHz, CDCl$_3$) δ 8.02 (d, *J* = 8.9 Hz, 1H), 7.70 (s, 1H), 7.60 (d, *J* = 1.2 Hz, 1H), 7.42 (dd, *J* = 5.0, 1.1 Hz, 1H), 7.35 (dd, *J* = 9.1, 2.8 Hz, 1H), 7.19–7.06 (m, 2H), 3.95 (s, 3H), 2.68 (d, *J* = 1.0 Hz, 3H).

^{13}C NMR (101 MHz, CDCl$_3$) δ 157.62, 131.34, 128.30, 128.13, 127.95–127.77 (m), 125.12, 121.65, 118.72, 102.28, 55.65, 19.27.

HRMS (ESI$^+$): m/z calculated for $C_{15}H_{13}NOSNa$ [M+Na]$^+$: 278.0621, found: 278.0609.

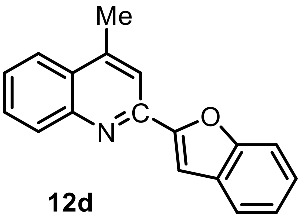

12d

2-(Benzofuran-2-yl)-4-methylquinoline (12d)

The title compound was prepared according to the **General procedure A**. The crude residue (0.1 mmol) was purified by column chromatography to give the product as a white solid (9.9 mg, 38%).

TLC: R_f = 0.40 (20:1 pentane:EtOAc).

^{1}H NMR (400 MHz, CDCl$_3$) δ 8.22 (d, *J* = 8.5 Hz, 1H), 7.99 (dd, *J* = 8.4, 1.4 Hz, 1H), 7.88 (d, *J* = 1.3 Hz, 1H), 7.76–7.61 (m, 4H), 7.56 (ddd, *J* = 8.3, 6.9, 1.3 Hz, 1H), 7.37 (ddd, *J* = 8.4, 7.3, 1.4 Hz, 1H), 7.28 (td, *J* = 7.8, 1.3 Hz, 1H), 2.78 (d, *J* = 1.0 Hz, 3H).

^{13}C NMR (101 MHz, CDCl$_3$) δ 155.70, 148.75, 148.06, 145.34, 130.23, 129.86, 128.94, 127.84, 126.63, 125.61, 123.87, 123.41, 121.92, 118.87, 111.93, 106.34, 19.14.

HRMS (EI$^+$): m/z calculated for $C_{18}H_{13}NO$ [M]$^+$: 259.0992, found: 259.0990.

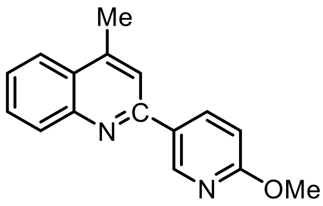

12e

2-(6-Methoxypyridin-3-yl)-4-methylquinoline (12e)

The title compound was prepared according to the **General procedure C**. The crude residue (0.2 mmol) was purified by column chromatography to give the product as a colorless oil (37.1 mg, 74%).

TLC: R_f = 0.40 (5:1 pentane:EtOAc).

^{1}H NMR (400 MHz, CDCl$_3$) δ 8.89 (d, *J* = 1.8 Hz, 1H), 8.45 (dd, *J* = 8.7, 2.5 Hz, 1H), 8.13 (d, *J* = 8.4 Hz, 1H), 7.99 (d, *J* = 9.0 Hz, 1H), 7.71 (ddd, *J* = 8.4, 6.8, 1.4 Hz, 1H), 7.68–7.62 (m, 1H), 7.54 (ddd, *J* = 8.2, 6.8, 1.3 Hz, 1H), 6.89 (dd, *J* = 8.7, 0.8 Hz, 1H), 4.02 (s, 3H), 2.76 (s, 3H).

^{13}C NMR (101 MHz, CDCl$_3$) δ 164.94, 154.63, 148.25, 146.29, 145.20, 138.06, 130.19, 129.63, 129.06, 127.29, 126.18, 123.79, 119.02, 111.12, 53.86, 19.21.

HRMS (ESI$^+$): m/z calculated for $C_{16}H_{15}N_2O$ [M+H]$^+$: 251.1179, found: 251.1179.

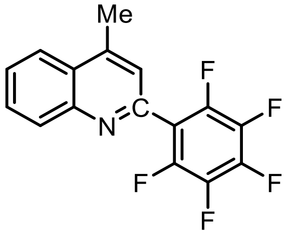

12f

4-Methyl-2-(perfluorophenyl)quinoline (12f)

The title compound was prepared according to the **General procedure C**. The crude residue (0.2 mmol) was purified by column chromatography to give the product as a yellow oil (31.5 mg, 51%).

TLC: R_f = 0.55 (30:1 pentane:EtOAc).

^{1}H NMR (400 MHz, CDCl$_3$) δ 8.16 (d, *J* = 8.4 Hz, 1H), 8.07 (d, *J* = 8.4 Hz, 1H), 7.78 (ddd, *J* = 8.4, 6.9, 1.4 Hz, 1H), 7.66 (ddd, *J* = 8.2, 6.9, 1.3 Hz, 1H), 7.38 (s, 1H), 2.79 (s, 3H).

^{13}C NMR (101 MHz, CDCl$_3$) δ 148.13, 146.93, 145.71, 130.46, 130.11, 127.74, 127.60, 123.96, 123.52, 19.01.

^{19}F NMR (376 MHz, CDCl$_3$) δ -138.65–-144.59 (m), -153.76 (d, *J* = 1.9 Hz), -159.50–-166.60 (m).

HRMS (ESI$^+$): m/z calculated for $C_{16}H_9F_5N$ [M+H]$^+$: 310.0650, found: 310.0650.

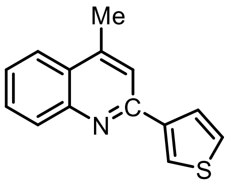

12g

4-Methyl-2-(naphthalen-2-yl)quinoline (12g)

The title compound was prepared according to the **General procedure C**. The crude residue (0.2 mmol) was purified by column chromatography to give the product as a colorless oil (32.0 mg, 71%).

TLC: R_f = 0.35 (20:1 pentane:EtOAc).

^{1}H NMR (400 MHz, CDCl$_3$) δ 8.12 (d, *J* = 8.5 Hz, 1H), 8.06–8.00 (m, 1H), 7.96 (d, *J* = 8.4 Hz, 1H), 7.87 (dt, *J* = 5.1, 1.3 Hz, 1H), 7.73–7.67 (m, 1H), 7.61 (s, 1H), 7.52 (ddt, *J* = 8.3, 6.9, 1.4 Hz, 1H), 7.43 (ddd, *J* = 4.9, 3.0, 1.2 Hz, 1H), 2.74 (s, 3H).

^{13}C NMR (101 MHz, CDCl$_3$) δ 153.15, 148.23, 144.84, 142.89, 130.14, 129.47, 127.35, 126.98, 126.38, 125.96, 124.57, 123.74, 119.89, 19.06.

HRMS (ESI$^+$): m/z calculated for $C_{14}H_{11}NSNa$ [M+Na]$^+$: 248.0504, found: 248.0504.

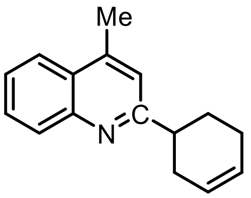

12h

2-(Cyclohex-3-en-1-yl)-4-methylquinoline (12h)

The title compound was prepared according to the **General procedure C**. The crude residue (0.2 mmol) was purified by column chromatography to give the product as a colorless oil (31.1 mg, 70%).

TLC: R_f = 0.50 (30:1 pentane:EtOAc).

^{1}H NMR (400 MHz, CDCl$_3$) δ 8.10 (d, *J* = 8.5 Hz, 1H), 7.95 (d, *J* = 8.3 Hz, 1H), 7.68 (t, *J* = 7.7 Hz, 1H), 7.58–7.41 (m, 1H), 7.19 (s, 1H), 6.02–5.72 (m, 2H), 3.19 (ddd, *J* = 9.6, 6.8, 2.9 Hz, 1H), 2.69 (s, 3H), 2.49–2.36 (m, 2H), 2.33–2.14 (m, 2H), 2.08 (ddd, *J* = 12.9, 5.4, 2.6 Hz, 1H), 1.94 (tdd, *J* = 12.4, 4.7, 2.0 Hz, 1H).

^{13}C NMR (101 MHz, CDCl$_3$) δ 165.89, 147.22, 145.12, 129.37, 129.26, 127.14, 127.05, 126.43, 125.77, 123.71, 120.49, 42.97, 31.43, 28.68, 25.71, 19.03.

HRMS (ESI$^+$): m/z calculated for $C_{16}H_{18}N$ [M+H]$^+$: 224.1434, found: 224.1432.

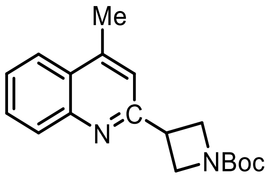

12i

***tert*-Butyl 3-(4-methylquinolin-2-yl)azetidine-1-carboxylate (12i)**

The title compound was prepared according to the **General procedure C**. The crude residue (0.2 mmol) was purified by column chromatography to give the product as a colorless oil (37.9 mg, 64%).

TLC: R_f = 0.20 (5:1 pentane:EtOAc).

^{1}H NMR (400 MHz, CDCl$_3$) δ 8.04 (dd, *J* = 8.5, 1.3 Hz, 1H), 7.95 (dd, *J* = 8.2, 1.5 Hz, 1H), 7.68 (ddd, *J* = 8.4, 6.9, 1.5 Hz, 1H), 7.52 (ddd, *J* = 8.3, 6.8, 1.3 Hz, 1H), 7.26 (s, 1H), 4.38 (t, *J* = 8.7 Hz, 2H), 4.27 (dd, *J* = 8.7, 5.9 Hz, 2H), 4.04–3.96 (m, 1H), 2.69 (s, 3H), 1.47 (s, 9H).

^{13}C NMR (101 MHz, $CDCl_3$) δ 160.99, 156.65, 147.54, 145.35, 129.69, 129.50, 127.23, 126.15, 123.74, 120.03, 79.63, 54.61, 35.84, 28.53, 18.90.

HRMS (ESI^+): m/z calculated for $C_{18}H_{22}N_2O_2Na$ $[M+Na]^+$: 321.1571, found: 321.1573.

4.2 Limitations of the B-to-C swapping reaction

General procedure A:

no desired product:

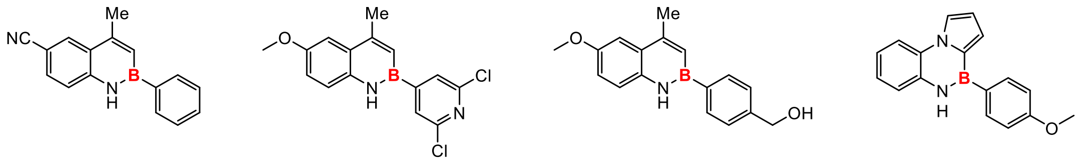

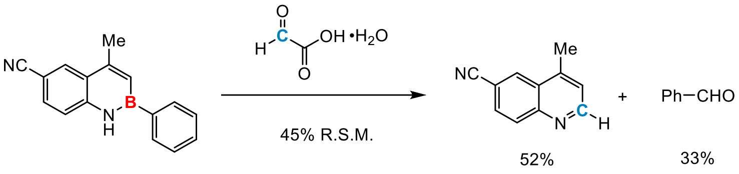

low yield:

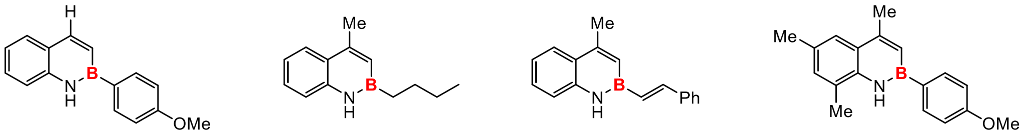

< 10%   < 10%   15%   < 5%

General procedure C:

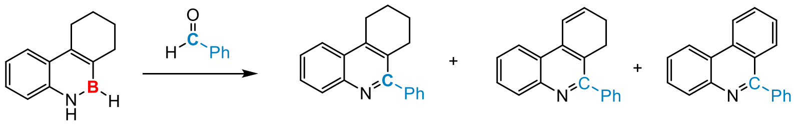

mixture

For **General Procedure A**, when a strong electron-withdrawing group is present on the phenyl ring of the 1,2-benzazaborine (for example, a cyano group at the 6-position), the expected substituent-rebound product is not obtained. Instead, only the quinoline without substitution at the C2 position is formed. We speculate that the strong electron-withdrawing effect may decelerate the final cyclization step, thereby suppressing formation of the desired product. Similarly, when the aryl group attached to boron bears a strong electron-withdrawing substituent, the anticipated product is not observed.

In addition, if there is no substituent at the 4-position of the 1,2-benzazaborine, the cyclization step cannot proceed smoothly.

Furthermore, when a substituent is present at the 3-position, both **General Procedure A** and **General Procedure C** fail to deliver the desired products efficiently.

5. Synthetic applications

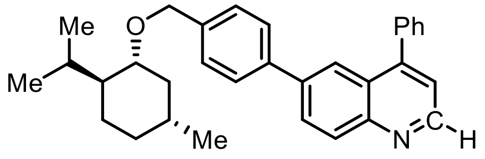

14a

6-(4-((((1*R*,2*S*,5*R*)-2-Isopropyl-5-methylcyclohexyl)oxy)methyl)phenyl)-4-phenylquinoline (14a)

The title compound was prepared according to the **General procedure B**. The crude residue (0.2 mmol) was purified by column chromatography to give the product as a colorless oil (58.4 mg, 65%).

TLC: R_f = 0.50 (30:1 pentane:EtOAc).

^{1}H NMR (400 MHz, CDCl$_3$) δ 8.94 (d, *J* = 4.4 Hz, 1H), 8.26 (dd, *J* = 8.8, 2.1 Hz, 1H), 8.10 (d, *J* = 2.2 Hz, 1H), 8.00 (dd, *J* = 8.7, 2.2 Hz, 1H), 7.61–7.50 (m, 7H), 7.42 (dd, *J* = 8.2, 2.1 Hz, 2H), 7.37 (d, *J* = 4.3 Hz, 1H), 4.70 (d, *J* = 11.5 Hz, 1H), 4.43 (d, *J* = 11.5 Hz, 1H), 3.20 (td, *J* = 10.7, 4.1 Hz, 1H), 2.32 (td, *J* = 7.0, 2.7 Hz, 1H), 2.25–2.15 (m, 1H), 1.70–1.59 (m, 2H), 1.42–1.24 (m, 2H), 0.92 (dd, *J* = 15.4, 6.8 Hz, 9H), 0.74 (d, *J* = 7.0 Hz, 3H).

^{13}C NMR (101 MHz, CDCl$_3$) δ 149.76, 149.04, 147.91, 139.75, 139.50, 138.95, 138.04, 130.21, 129.69, 129.33, 128.85, 128.71, 128.47, 127.58, 127.06, 123.69, 121.90, 79.12, 70.18, 48.46, 40.46, 34.70, 31.72, 25.69, 23.38, 22.53, 21.18, 16.25.

HRMS (ESI$^+$): m/z calculated for $C_{32}H_{35}NONa$ [M+Na]$^+$: 472.2611, found: 472.2611.

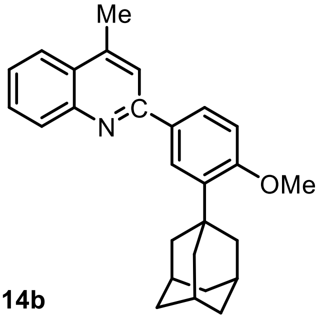

14b

2-(3-((3*R*,5*R*,7*R*)-Adamantan-1-yl)-4-methoxyphenyl)-4-methylquinoline (14b)

The title compound was prepared according to the **General procedure A**. The crude residue (0.2 mmol) was purified by column chromatography to give the product as a white solid (40.0 mg, 52%).

TLC: R_f = 0.30 (20:1 pentane:EtOAc).

^{1}H NMR (400 MHz, CDCl$_3$) δ 8.17 (d, *J* = 8.4 Hz, 1H), 8.08 (d, *J* = 2.2 Hz, 1H), 7.96 (td, *J* = 6.1, 2.9 Hz, 2H), 7.68 (dd, *J* = 8.4, 1.6 Hz, 2H), 7.57–7.44 (m, 1H), 7.00 (d, *J* = 8.5 Hz, 1H), 3.91 (s, 3H), 2.76 (s, 3H), 2.21 (s, 6H), 2.12 (d, *J* = 5.2 Hz, 3H), 1.81 (s, 6H).

^{13}C NMR (101 MHz, CDCl$_3$) δ 160.19, 157.56, 148.24, 144.55, 138.81, 132.01, 130.24, 129.26, 127.05, 126.33, 126.26, 125.65, 123.68, 119.70, 111.92, 55.28, 40.65, 37.35, 37.30, 29.27, 19.17.

HRMS (ESI$^+$): m/z calculated for $C_{27}H_{29}NONa$ [M+Na]$^+$: 406.2141, found: 406.2142.

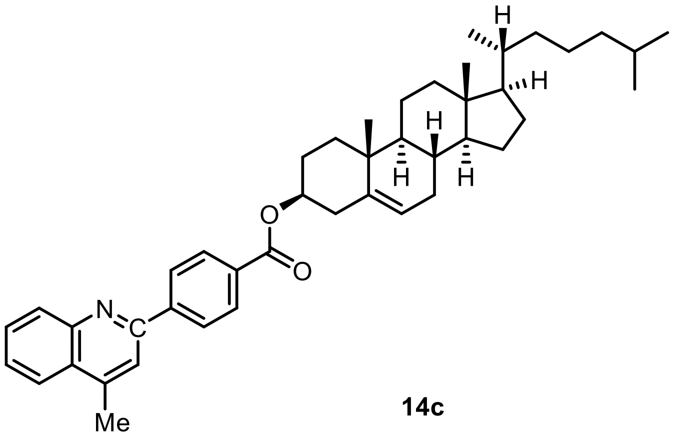

14c

(3*S*,8*S*,9*S*,10*R*,13*R*,14*S*,17*R*)-10,13-Dimethyl-17-((*R*)-6-methylheptan-2-yl)-2,3,4,7,8,9,10,11,12,13,14,15,16,17-tetradecahydro-1*H*-cyclopenta[*a*]phenanthren-3-yl 4-(4-methylquinolin-2-yl)benzoate (14c)

The title compound was prepared according to the **General procedure C**. The crude residue (0.1 mmol) was purified by column chromatography to give the product as a white solid (33.7 mg, 55%).

TLC: R_f = 0.35 (20:1 pentane:EtOAc).

^{1}H NMR (400 MHz, CDCl$_3$) δ 8.30–8.13 (m, 5H), 8.01 (d, *J* = 8.4 Hz, 1H), 7.81–7.69 (m, 2H), 7.57 (t, *J* = 7.6 Hz, 1H), 5.44 (s, 1H), 4.90 (dtd, *J* = 12.2, 8.3, 4.4 Hz, 1H), 2.78 (s, 3H), 2.51 (d, *J* = 7.4 Hz, 2H), 2.06–1.72 (m, 7H), 1.63–1.46 (m, 6H), 1.35 (d, *J* = 9.6 Hz, 3H), 1.28–1.11 (m, 6H), 1.09 (s, 3H), 1.00 (dt, *J* = 11.6, 6.1 Hz, 4H), 0.93 (d, *J* = 6.5 Hz, 3H), 0.87 (dt, *J* = 6.7, 1.4 Hz, 6H), 0.69 (s, 3H).

^{13}C NMR (101 MHz, CDCl$_3$) δ 165.97, 155.90, 148.16, 145.35, 143.75, 139.80, 131.38, 130.50, 130.13, 129.72, 127.60, 127.50, 126.64, 123.79, 122.93, 119.88, 74.89, 56.82, 56.26, 50.17, 42.45, 39.87, 39.66, 38.38, 37.18, 36.80, 36.32, 35.94, 32.08, 32.01, 28.38, 28.16, 28.05, 24.43, 23.98, 22.97, 22.71, 21.19, 19.54, 19.21, 18.86, 12.00.

HRMS (ESI$^+$): m/z calculated for $C_{44}H_{57}NO_2Na$ [M+Na]$^+$: 654.4282, found: 654.4282.

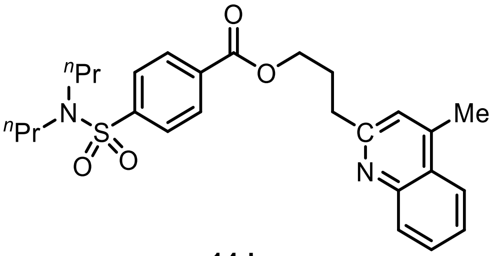

14d

3-(4-Methylquinolin-2-yl)propyl 4-(*N*,*N*-dipropylsulfamoyl)benzoate (14d)

The title compound was prepared according to the **General procedure C**. The crude residue (0.1 mmol) was purified by column chromatography to give the product as a colorless oil (24.6 mg, 53%).

TLC: R_f = 0.30 (3:1 pentane:EtOAc).

^{1}H NMR (400 MHz, CDCl$_3$) δ 8.06–7.98 (m, 3H), 7.96–7.91 (m, 1H), 7.78 (d, *J* = 8.6 Hz, 2H), 7.68 (ddd, *J* = 8.5, 6.9, 1.6 Hz, 1H), 7.52 (ddd, *J* = 8.5, 6.9, 1.4 Hz, 1H), 7.16 (d, *J* = 1.8 Hz, 1H), 4.52–4.43 (m, 2H), 3.14–3.06 (m, 6H), 2.65 (s, 3H), 2.43–2.31 (m, 2H), 1.53 (tdd, *J* = 9.3, 7.5, 3.9 Hz, 4H), 0.87 (td, *J* = 7.3, 1.6 Hz, 6H).

^{13}C NMR (101 MHz, CDCl$_3$) δ 165.36, 161.10, 147.89, 144.74, 144.24, 133.65, 130.27, 129.47, 129.39, 127.00, 125.86, 123.78, 122.14, 65.55, 50.05, 35.79, 28.54, 22.06, 18.83, 11.30.

HRMS (ESI$^+$): m/z calculated for $C_{26}H_{32}N_2O_4SNa$ [M+Na]$^+$: 491.1975, found: 491.1978.

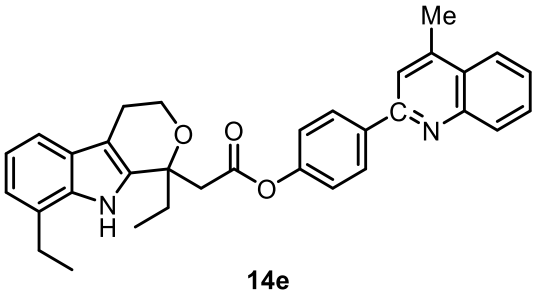

14e

4-(4-Methylquinolin-2-yl)phenyl 2-(1,8-diethyl-1,3,4,9-tetrahydropyrano[*3,4-b*]indol-1-yl)acetate (14e)

The title compound was prepared according to the **General procedure C**. The crude residue (0.1 mmol) was purified by column chromatography to give the product as a colorless oil (33.0 mg, 66%).

TLC: R_f = 0.40 (3:1 pentane:EtOAc).

^{1}H NMR (400 MHz, CDCl$_3$) δ 8.84 (s, 1H), 8.20 (d, *J* = 8.6 Hz, 3H), 8.02 (dd, *J* = 8.4, 1.4 Hz, 1H), 7.75 (ddd, *J* = 8.4, 6.9, 1.4 Hz, 1H), 7.72–7.70 (m, 1H), 7.58 (ddd, *J* = 8.3, 6.8, 1.3 Hz, 1H), 7.40 (d, *J* = 7.7 Hz, 1H), 7.20 (d, *J* = 8.7 Hz, 2H), 7.09 (t, *J* = 7.5 Hz, 1H), 7.04–7.01 (m, 1H), 4.12 (dt, *J* = 11.3, 4.8 Hz, 1H), 4.04 (ddd, *J* = 11.5, 7.5, 4.4 Hz, 1H), 3.37–3.19 (m, 2H), 2.94–2.81 (m, 4H), 2.79 (s, 3H), 2.25 (dt, *J* = 14.7, 7.3 Hz, 1H), 2.20–2.11 (m, 1H), 1.33 (t, *J* = 7.6 Hz, 3H), 0.94 (t, *J* = 7.4 Hz, 3H).

^{13}C NMR (101 MHz, CDCl$_3$) δ 171.52, 155.98, 151.41, 135.61, 134.64, 130.16, 129.81, 129.05, 127.38, 126.82, 126.47, 126.27, 123.81, 122.03, 120.68, 119.88, 119.72, 116.12, 108.92, 74.89, 60.89, 43.34, 30.96, 24.23, 22.54, 19.26, 13.89, 7.85.

HRMS (ESI$^+$): m/z calculated for $C_{33}H_{32}N_2O_3Na$ [M+Na]$^+$: 527.2305, found: 527.2302.

6. Mechanistic investigations

6.1. UV-vis absorption spectrum

UV/vis absorption spectra were recorded on a Jasco V-730 spectrophotometer, equipped with a temperature control unit at 25 °C. The samples were measured in Starna® fluorescence quartz cuvettes (type: 29-F, chamber volume = 1.400 mL, H × W × D = 48 mm × 12.5 mm × 12.5 mm, path length = 10 mm)

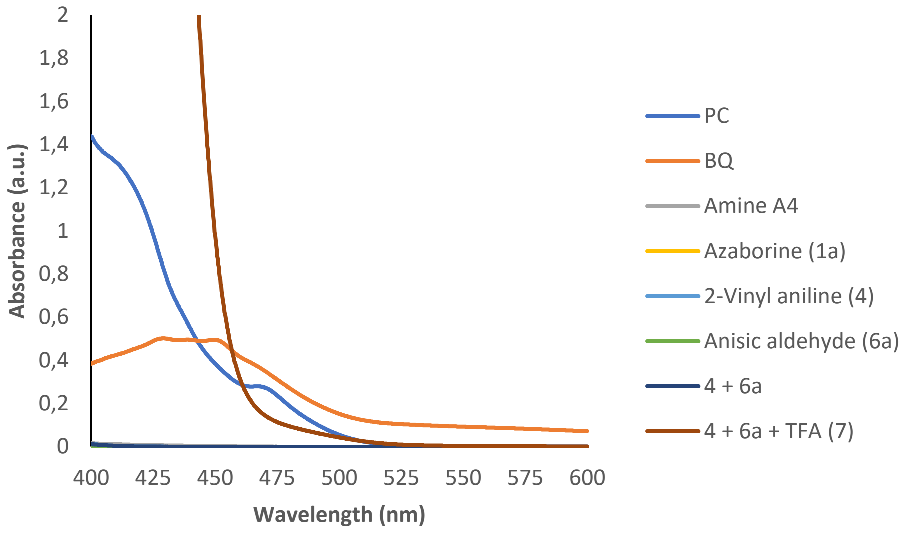

Supplementary Figure 5. UV-vis absorption spectrum in 1,2-dichloroethane.

As shown in **Supplementary Figure 5**, the UV–vis absorption spectra of photocatalyst $[Ir(ppy)_2dtbbpy]PF_6$ (2×10^{-4} mol L^{-1}), BQ (2×10^{-2} mol L^{-1}), amine **A4** (indoline, 2×10^{-2} mol L^{-1}), 1,2-benzazaborine **1a** (2×10^{-2} mol L^{-1}), 2-vinylaniline **4** (2×10^{-2} mol L^{-1}), anisic aldehyde **6a** (2×10^{-2} mol L^{-1}), a mixture of 2-vinylaniline **4** and anisic aldehyde **6a** (each 2×10^{-2} mol L^{-1}), and the corresponding imine **7** generated from 2-vinylaniline **4** (2×10^{-2} mol L^{-1}), anisic aldehyde **6a** (2×10^{-2} mol L^{-1}), and trifluoroacetic acid (2×10^{-2} mol L^{-1}) were recorded in 1,2-dichloroethane.

6.2. Stern-Volmer quenching studies

Stern-Volmer luminescence quenching analysis was conducted using a Jasco FP-8300 spectrofluorometer using Starna® fluorescence quartz cuvettes (type: 29-F, chamber volume = 1.400 mL, H × W × D = 48 mm × 12.5 mm × 12.5 mm, path length = 10 mm). The following parameters were set: data interval = 0.5 nm, scan-speed = 500 nm/min, excitation wavelength λ_{ex} = 405 nm, measured luminescence wavelength λ = 585 nm. All samples used in the luminescence quenching-based screening studies were prepared in an argon-filled glovebox with degassed and dry solvents. Stock solutions of potential quenchers (0.005 M each) and the photocatalyst $[Ir(ppy)_2(dtbbpy)]PF_6$ ($5*10^{-5}$ M) were prepared initially. Stern-Volmer luminescence quenching studies were performed using a stock solution of the photocatalyst and variable concentrations of the potential quenchers at room temperature under an argon atmosphere. The samples were prepared by dilution in the 1.4 mL quartz cuvettes inside the argon-filled glovebox. The solutions were irradiated at 405 nm and the luminescence was measured at 585 nm. The ratio of I_0/I was plotted as a function of the quencher concentration [Quencher] (I_0 = emission intensity of the photocatalyst in isolation at the specified wavelength; I = observed emission intensity of the photocatalyst with added quencher).

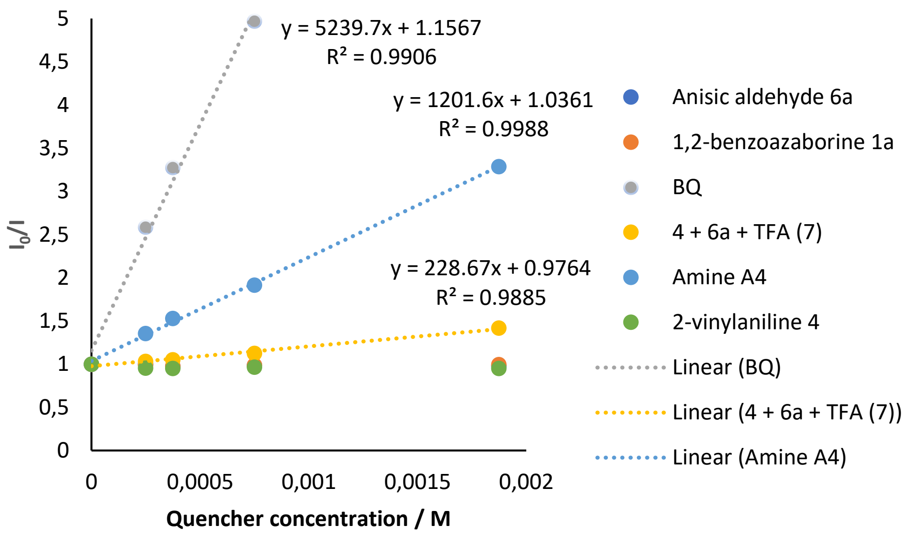

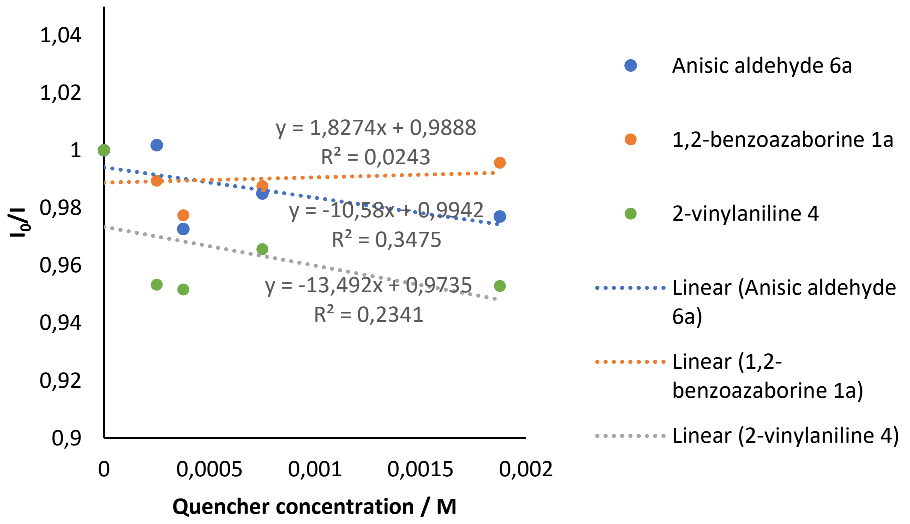

Supplementary Figure 6. Stern-Volmer luminescence quenching analysis using $[Ir(ppy)_2(dtbbpy)]PF_6$ as photocatalyst with BQ, amine **A4** (indoline), 1,2-benzazaborine **1a**, 2-vinylaniline **4**, anisic aldehyde **6a**, and the corresponding imine **7** generated from 2-vinylaniline **4** (2×10^{-2} mol L^{-1}), anisic aldehyde **6a** (2×10^{-2} mol L^{-1}), and trifluoroacetic acid (2×10^{-2} mol L^{-1}) as quenchers in 1,2-dichloroethane. The lower plot shows the same quenching data to better visualize partially overlapping data points in the upper Stern–Volmer plot.

6.3. Cyclic voltammetry studies

To determine the redox potentials cyclic voltammetry studies (CV) with a standard three electrode setup was carried out. The setup was equipped with a reference electrode (Ag/AgCl; aq. sat. KCl solution), a working electrode (3 mm glassy carbon disc electrode) and counter electrode (platinum wire) on a CHI600e electrochemical workstation (CH Instruments, Austin, Texas, USA). The electrolyte solution contained 0.1 M tetrabutylammonium hexafluorophosphate ($TBAPF_6$) and 1 mM of the given substrate in MeCN. Before each measurement, the solution was purged with N_2 gas to avoid the interference of atmospheric oxygen. First a blank CV and ferrocene was measured, followed by the first substrate (0.005 M), which was dissolved in previously prepared electrolyte solution (0.1 M). The process was repeated independently for all reagents of the described reactions. In between the measurements all electrodes were washed with respective solvent and a new 20 mL vial as experimental setup was taken. No additional blank CV was performed. All CV studies were carried out under argon atmosphere at rt and with a scan rate of 0.1 $V \cdot s^{-1}$. Fc/Fc^+ was found to be +0.51 V vs Ag/Ag^+ (sat. KCl) and hence the CV measurements of starting materials were referenced against Fc/Fc^+. The scan rate was set at 0.1 V/s (E_{step} = 0.002 V) and 10 scans were taken for each compound in the potential window of −2.0 V to +2.0 V.

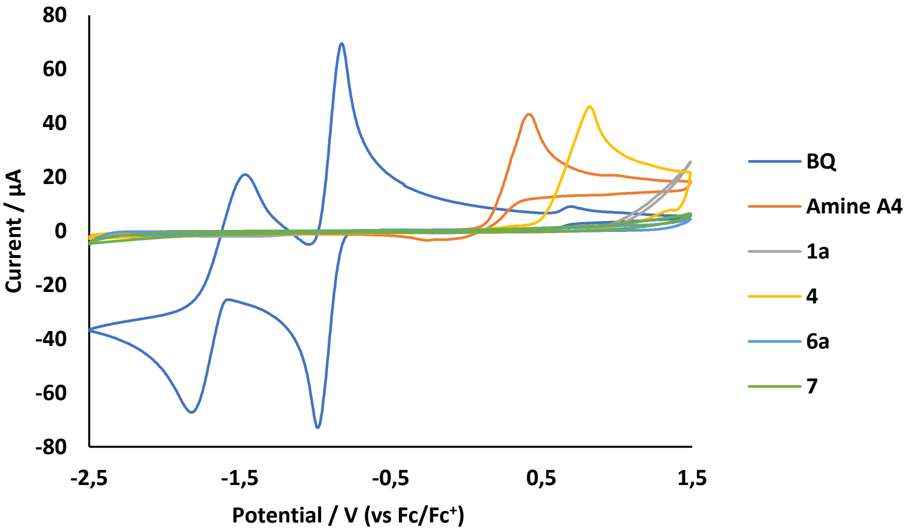

Supplementary Figure 7. Cyclic voltammetry spectrum of BQ, amine **A4**, 1,2-benzazaborine **1a**, 2-vinyl-aniline **4**, anisic aldehyde **6a** (2×10^{-2} mol L^{-1}), and the corresponding imine **7** generated from 2-vinyl-aniline **4**, anisic aldehyde **6a**, and trifluoroacetic acid (all 0.005 M in MeCN (0.1 M $^{n}Bu_4N(PF_6)$)). The scanning speed was set at 0.1 V/s.

To compare the results, the given potentials were recalculated according to the determined half peak potential of saturated calomel electrode (in MeCN) as internal standard by equation:[8]

$$E^{1/2\prime} \text{ (vs SCE)} = E^{1/2} \text{ (vs Fc/Fc}^{+}\text{)} + 0.382 \text{ V}$$

Substrate	$E^{1/2}$ / V (vs SCE)
BQ	-0.54
amine **A4**	0.63
2-vinyl-aniline **4**	0.94

Supplementary Table 8. Results of the cyclic voltammetry measurements.

According to the results, the determined half peak potentials of the measured substrates could be determined and are shown in **Supplementary Table 8**. The half peak potentials of BQ and amine **A4** are located well in the redox region of excited photocatalyst $[Ir(ppy)_2(dtbbpy)]PF_6$ ($Ir(III)^*/(II)^{1/2}$ = 0.66 V; $Ir(III)^*/(IV)^{1/2}$ = −0.96 V)[9].

6.4 Control experiments

6.4.1 Protodeborylation

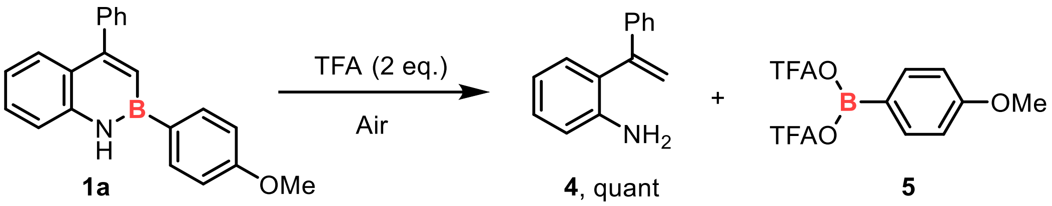

In an oven-dried 10 ml Schlenk tube equipped with a PTFE-coated rare-earth “extra power" oval stirring bar, 1,2-benzazaborine (0.2 mmol, 1.0 eq.) were charged under air. Then chloroform-*d* (2.0 mL, 0.1 M) and trifluoroacetic acid (TFA) (31 µL, 0.4 mmol, 2.0 eq.) were added under air. The vessel was sealed with the screw cap and a needle was inserted through the cap. The reaction was running for 16 hours at room temperature. After the reaction, mesitylene was added to the reaction mixture as an internal standard, and a crude NMR spectrum was recorded (mesitylene as an internal standard).

6.4.2 Aldehyde formation

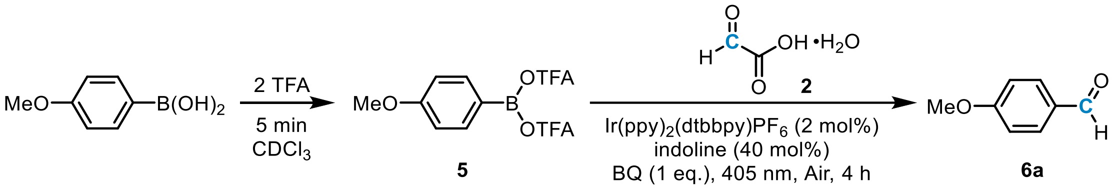

Entry	deviation from standard conditions	**6a** (%)
1	-	67
2	w/o indoline	0
3	w/o BQ	30
4	w/o $[Ir(ppy)_2(dtbbpy)]PF_6$, no LEDs	42
5	Aniline (40 mol%) as catalyst	6

The yield was determined by ^{1}H NMR analysis using mesitylene as an internal standard.

Supplementary Table 9. Control experiments for aldehyde formation.

6.4.3 Rearomatisation

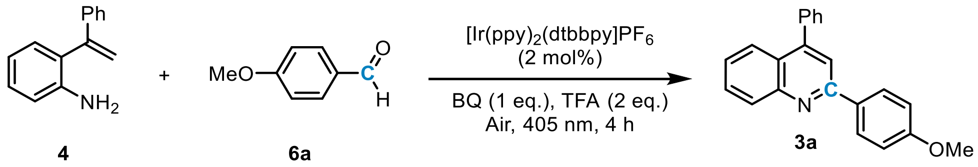

Entry	deviation from standard conditions	**6a** (%)
1	-	100
2	w/o BQ	89
3	w/o $[Ir(ppy)_2(dtbbpy)]PF_6$	70
4	w/o $[Ir(ppy)_2(dtbbpy)]PF_6$, no LEDs	52
5	w/o BQ, 16 h	89
6	*p*-$MeOC_6H_4BF_3K$ (1 eq.), indoline (0.4 eq.), and **2** (3 eq.) instead of **6a**	33

The yield was determined by ^1H NMR analysis using mesitylene as an internal standard.

Supplementary Table 10. Control experiments for rearomatisation.

6.4.4 Control experiments to evaluate a possible CO-mediated pathway

A possible alternative pathway involving in situ generation of CO from glyoxylic acid was considered, since glyoxylic acid has been reported to undergo acid-promoted decomposition to release CO[10]. However, the reported CO-generation process requires relatively harsh conditions, such as concentrated sulfuric acid at 130 °C. In contrast, the present reaction is carried out at room temperature using TFA as the acid and DCE as the solvent under 405 nm irradiation. Therefore, efficient CO formation from glyoxylic acid under our standard reaction conditions was considered unlikely.

To experimentally evaluate this possibility, we attempted to perform a representative carbonylation reaction from this report under our standard conditions[10]. Indeed, no corresponding amide product was detected, indicating that CO formation is not occurring to a meaningful extent under our conditions. In addition, we carried out a further control experiment using CO gas (1 atm) as an alternative carbon source in our model reaction. However, no desired product was observed. Taken together, these control experiments suggest that a CO-mediated pathway is unlikely to account for the observed product formation under the present reaction conditions.

Control experiment 1:

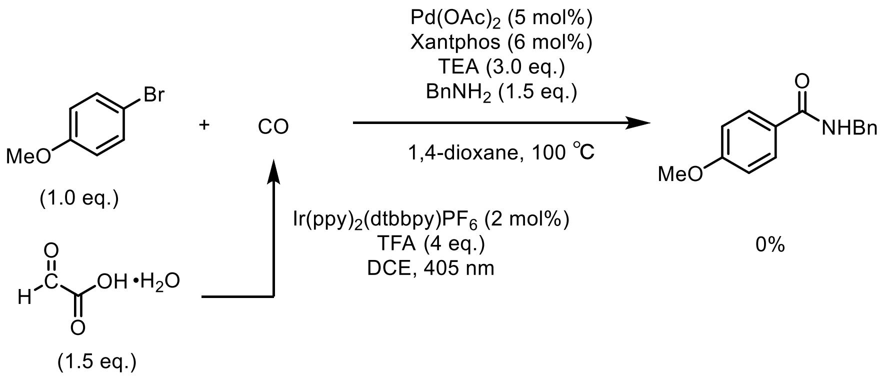

Control experiment 2:

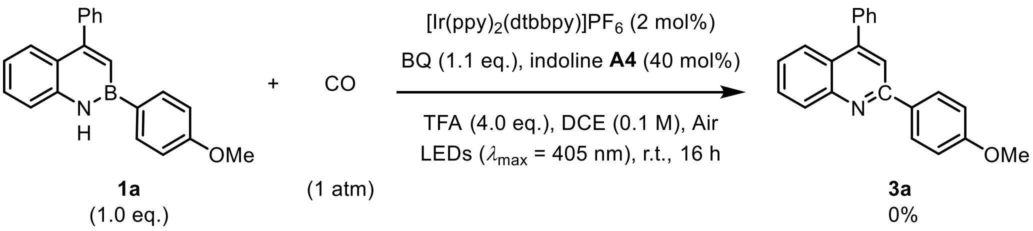

6.5 Plausible mechanism for substituent-rebound B-to-C swapping reaction

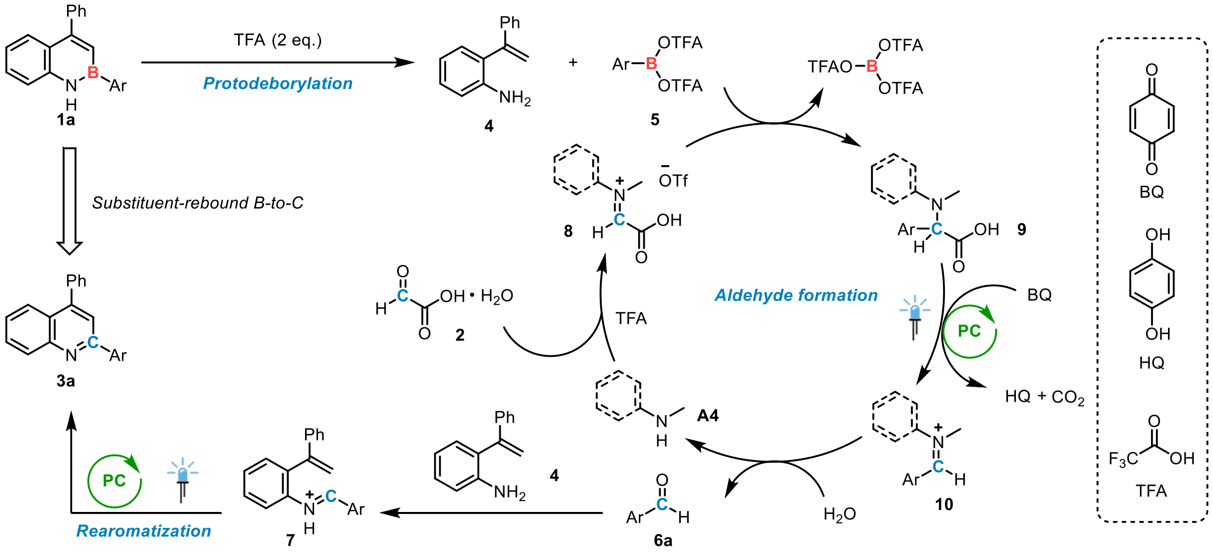

Supplementary Figure 8. Plausible mechanism for substituent rebound B-to-C swapping reaction

6.6 Plausible mechanism for B-to-C swapping reaction using aldehydes as substrates

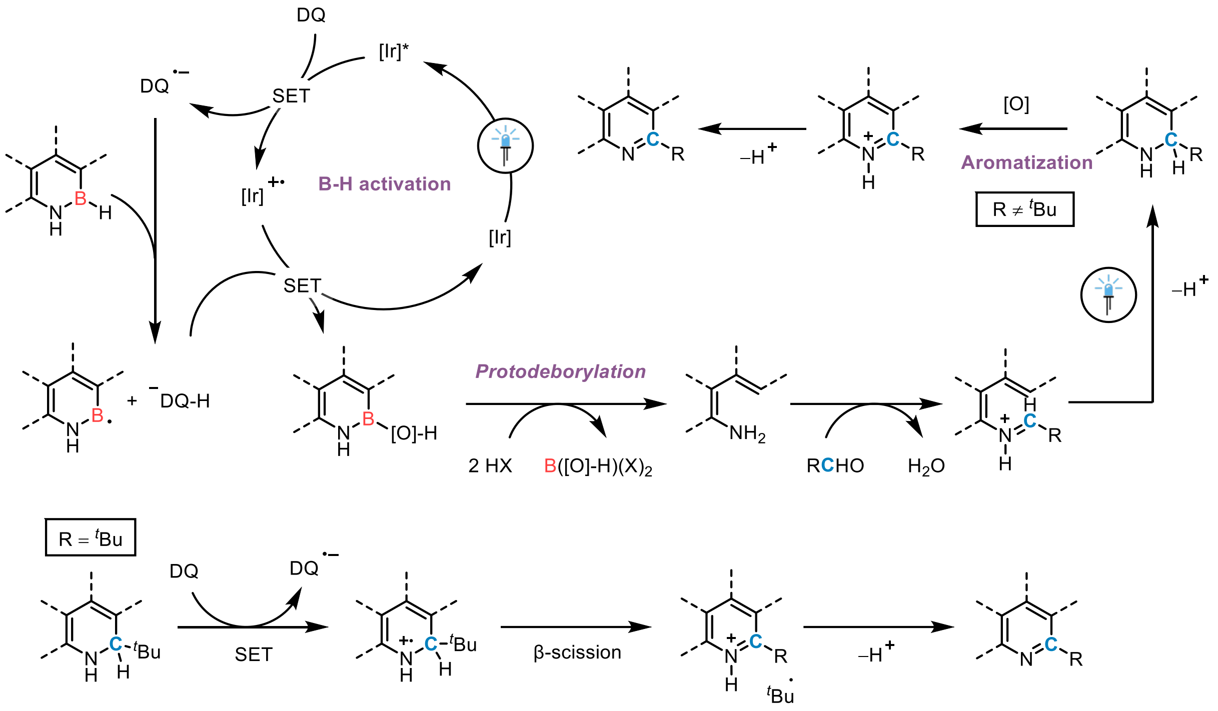

Supplementary Figure 9. Plausible mechanism for B-to-C swapping reaction using aldehydes as substrates

6.7 Plausible mechanism for alkyl-substituent-rebound B-to-C swapping reaction

Based on prior literature reports[11], we tentatively propose that a formyl radical, generated upon photocatalytic SET followed by decarboxylation of glyoxylic acid, can capture a Ni(II)-alkyl intermediate generated from transmetallation with the corresponding alkyl boronic ester. The resulting Ni(III) complex can then undergo reductive elimination to form the desired aldehyde species which can react with 2-vinylaniline **4**, followed by cyclisation and rearomatisation to furnish the quinoline product.

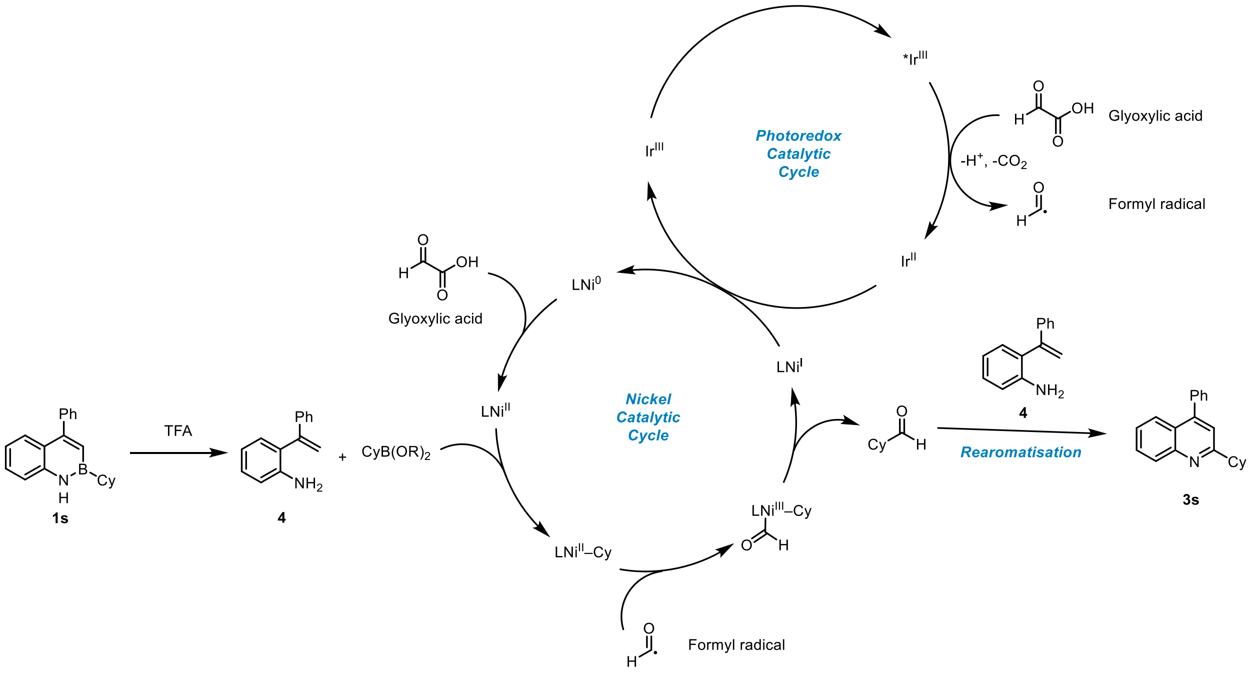

Supplementary Figure 10. Plausible mechanism for alkyl-substituent-rebound B-to-C swapping reaction

Notably, α-keto acids have been reported to oxidise low-valent metal species under irradiation through ligand-to-metal charge transfer (LMCT) processes[12]. Therefore, we speculate that glyoxylic acid may also serve as an oxidant to regenerate the active Ni catalyst under our reaction conditions. In support of this possibility, our control experiments showed that the use of external oxidants, such as air or 1,4-benzoquinone, significantly decreased the efficiency of the Ni-cocatalyzed B-to-C atom exchange reaction. These results suggest that common external oxidants are not suitable for this transformation and that glyoxylic acid itself may serve as a plausible oxidant in the present system. In addition, no desired product was observed when indoline was added as an additive, suggesting that a Petasis borono–Mannich-type pathway is unlikely to account for the present transformation under these conditions.

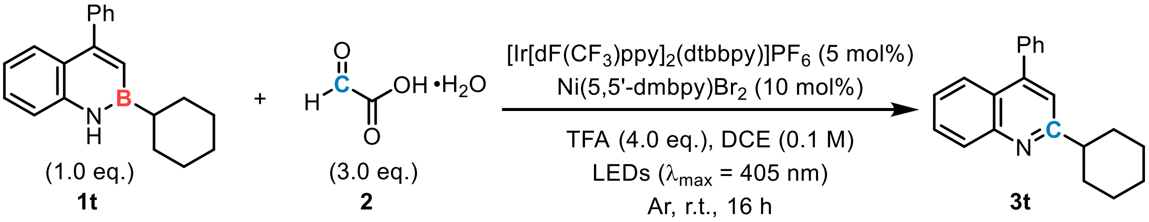

Entry	Deviations from the std. conditions	**3t** (%)
1	-	33
2	Under air	15

3	1,4-Benzoquinone (1.0 eq.) as the additive	0
4	Duroquinone (1.0 eq.) as the additive	0
5	Indoline (40 mol%) as the additive	0

The yield was determined by ^1H NMR analysis using mesitylene as an internal standard.

Supplementary Table 11. Control experiments for alkyl-substituent-rebound B-to-C swapping reaction.

Although the precise manner in which glyoxylic acid oxidizes Ni(0) to Ni(II) remains unclear at this stage, these findings provide useful mechanistic insight into the role of glyoxylic acid beyond serving as the carbon atom source. We hope that these observations may also provide a useful basis for future mechanistic studies.

6.8 A comparison experiment with classical Suzuki coupling conditions

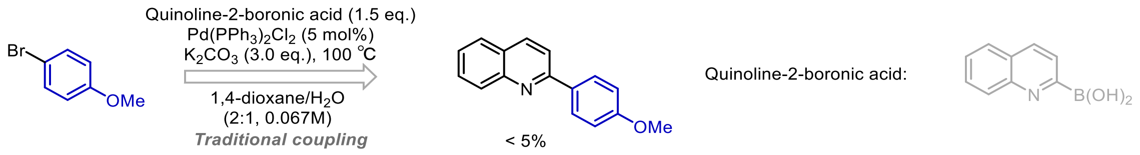

To compare the present B-to-C atom-swapping methodology with a conventional cross-coupling approach, a control experiment was performed using quinoline-2-boronic acid as the nucleophilic coupling partner under classical Suzuki coupling conditions. Specifically, the aryl bromide substrate was treated with quinoline-2-boronic acid (1.5 equiv.), $Pd(PPh_3)_2Cl_2$ (5 mol%), and K_2CO_3 (3.0 equiv.) in 1,4-dioxane/H_2O (2:1, 0.067 M) at 100 °C. Under these conditions, only a trace amount of the corresponding cross-coupled product was observed, with an estimated yield of <5% as determined by crude ^1H NMR analysis using CH_2Br_2 as an internal standard. This result suggests that, under the tested conditions, direct Suzuki coupling with quinoline-2-boronic acid is inefficient for accessing this 2-substituted quinoline product.

7. X-Ray analysis

X-Ray diffraction: Data sets for compounds **13b**, **14b** and **14c** were collected with a Bruker D8 Venture Photon III Diffractometer. Programs used: data collection: *APEX6* Version 2024.9-0[13] (Bruker AXS Inc., **2024**); cell refinement: *SAINT* Version 8.41 (Bruker AXS Inc., **2024**); data reduction: *SAINT* Version 8.41 (Bruker AXS Inc., **2024**); absorption correction, *SADABS* Version 2016/2 (Bruker AXS Inc., **2024**); structure solution *SHELXT*-Version 2018-3[14] (Sheldrick, G. M. *Acta Cryst.,* **2015**, *A71*, 3-8); structure refinement *SHELXL*- Version 2019-2[15] (Sheldrick, G. M. *Acta Cryst.,* **2015**, *C71* (1), 3-8) and graphics,

XP[16] (Version 5.1, Bruker AXS Inc., Madison, Wisconsin, USA, **1998**). *R*-values are given for observed reflections, and wR^2 values are given for all reflections.

Exceptions and special features: For compound **14c** the 1,5-dimethyl-hexyl-group was found disordered over two positions in the asymmetric unit. Several restraints (SADI, SAME, ISOR and SIMU) were used in order to improve refinement stability.

X-ray crystal structure analysis of 13b: A colourless, needle-shaped crystal was mounted on a loop with perfluoroether oil. The crystals were recrystallised from a mixture of DCM and pentane. Data for glo10770 were collected from a single crystal in 6.04 hours at 100(2) K on a Bruker D8 VENTURE KAPPA diffractometer with a microfocus sealed tube using a multilayer mirror as monochromator and a Bruker PHOTON III CPAD detector. The diffractometer was equipped with an Oxford Cryostream 1000 low temperature device and used Mo K_α radiation (λ = 0.71073 Å). All data were integrated with SAINT V8.41, yielding 58166 reflections of which 4377 where independent and 77.6% were greater than $2\sigma(F^2)$. A Multi-Scan absorption correction using SADABS 2016/2 was applied. The structure was solved by Intrinsic Phasing methods with SHELXT 2018/2 and refined by full-matrix least-squares methods against F^2 using SHELXL-2019/2. All non-hydrogen atoms were refined with anisotropic displacement parameters. All hydrogen atoms were refined with isotropic displacement parameters. Some of their coordinates were refined freely and some on calculated positions using a riding model with their U_{iso} values constrained to 1.5 times the U_{eq} of their pivot atoms for terminal sp^3 carbon atoms and 1.2 times for all other carbon atoms (the hydrogen at N1 atom was refined freely). Crystallographic data for the structures reported in this paper have been deposited with the Cambridge Crystallographic Data Centre. CCDC 2428786 contain the supplementary crystallographic data for this paper. These data can be obtained free of charge from The Cambridge Crystallographic Data Centre via www.ccdc.cam.ac.uk/structures.

CCDC number	2428786
Empirical formula	$C_{26}H_{30}BNO$
Formula weight	383.32
Temperature [K]	100(2)
Crystal system	monoclinic
Space group (number)	$P2_1/n$ (14)
a [Å]	15.0980(7)
b [Å]	6.5115(4)
c [Å]	21.6909(12)
α [°]	90
β [°]	105.108(2)
γ [°]	90
Volume [Å^3]	2058.7(2)

Z	4
ρ_{calc} [gcm^{-3}]	1.237
μ [mm^{-1}]	0.073
F(000)	824
Crystal size [mm^3]	0.058×0.06×0.126
Crystal colour	colourless
Crystal shape	needle
Radiation	Mo K_α (λ=0.71073 Å)
2θ range [°]	3.80 to 53.56 (0.79 Å)
Index ranges	−19 ≤ h ≤ 19<br>−8 ≤ k ≤ 8<br>−27 ≤ l ≤ 27
Reflections collected	58166
Independent reflections	4377<br>R_{int} = 0.0844<br>R_{sigma} = 0.0360
Completeness to θ = 25.242°	99.7
Data / Restraints / Parameters	4377 / 0 / 268
Goodness-of-fit on F^2	1.072
Final *R* indexes<br>[$I \geq 2\sigma(I)$]	R_1 = 0.0513<br>wR_2 = 0.1300
Final *R* indexes<br>[all data]	R_1 = 0.0682<br>wR_2 = 0.1402
Largest peak/hole [eÅ^{-3}]	0.23/−0.22

Supplementary Table 12. Crystal data and structure refinement for **13b.**

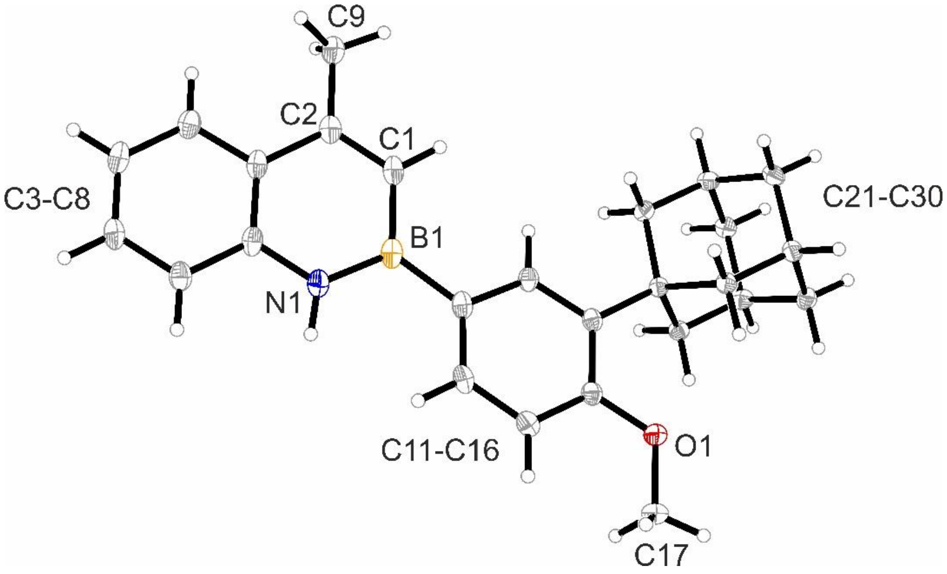

Supplementary Figure 11. Crystal structure of compound **13b**. Thermal ellipsoids are shown at 30% probability.

X-ray crystal structure analysis of 14b: A colourless, prism-shaped crystal was mounted on the goniometer. The crystals were recrystallised from a mixture of DCM and pentane. Data for glo10768

were collected from a single crystal in 14.10 hours at 100(2) K on a Bruker D8 VENTURE KAPPA diffractometer with a microfocus sealed tube using a multilayer mirror as monochromator and a Bruker PHOTON III CPAD detector. The diffractometer used Mo K_α radiation (λ = 0.71073 Å). All data were integrated with SAINT V8.41, yielding 56781 reflections of which 4265 where independent and 78.1% were greater than $2\sigma(F^2)$. A Multi-Scan absorption correction using SADABS 2016/2 was applied. The structure was solved by Intrinsic Phasing methods with SHELXT 2018/2 and refined by full-matrix least-squares methods against F^2 using SHELXL-2019/2. All non-hydrogen atoms were refined with anisotropic displacement parameters. All hydrogen atoms were refined isotropic on calculated positions using a riding model with their U_{iso} values constrained to 1.5 times the U_{eq} of their pivot atoms for terminal sp^3 carbon atoms and 1.2 times for all other carbon atoms. Crystallographic data for the structures reported in this paper have been deposited with the Cambridge Crystallographic Data Centre. CCDC 2428787 contain the supplementary crystallographic data for this paper. These data can be obtained free of charge from The Cambridge Crystallographic Data Centre via www.ccdc.cam.ac.uk/structures.

CCDC number	2428787
Empirical formula	$C_{27}H_{29}NO$
Formula weight	383.51
Temperature [K]	100(2)
Crystal system	monoclinic
Space group (number)	$P2_1/n$ (14)
a [Å]	14.7954(12)
b [Å]	6.6524(4)
c [Å]	21.2207(16)
α [°]	90
β [°]	105.620(3)
γ [°]	90
Volume [Å^3]	2011.5(3)
Z	4
ρ_{calc} [gcm^{-3}]	1.266
μ [mm^{-1}]	0.076
F(000)	824
Crystal size [mm^3]	0.035×0.073×0.106
Crystal colour	colourless
Crystal shape	prism
Radiation	Mo K_α (λ=0.71073 Å)
2θ range [°]	5.72 to 53.46 (0.79 Å)
Index ranges	$-18 \le h \le 18$<br>$-8 \le k \le 8$<br>$-26 \le l \le 26$
Reflections collected	56781
Independent reflections	4265

	R_{int} = 0.0855<br>R_{sigma} = 0.0356
Completeness to θ = 25.242°	99.6
Data / Restraints / Parameters	4265 / 0 / 264
Goodness-of-fit on F^2	1.073
Final *R* indexes<br>[$I \geq 2\sigma(I)$]	R_1 = 0.0614<br>wR_2 = 0.1723
Final *R* indexes<br>[all data]	R_1 = 0.0773<br>wR_2 = 0.1831
Largest peak/hole [eÅ^{-3}]	0.40/−0.27

Supplementary Table 13. Crystal data and structure refinement for **14b**

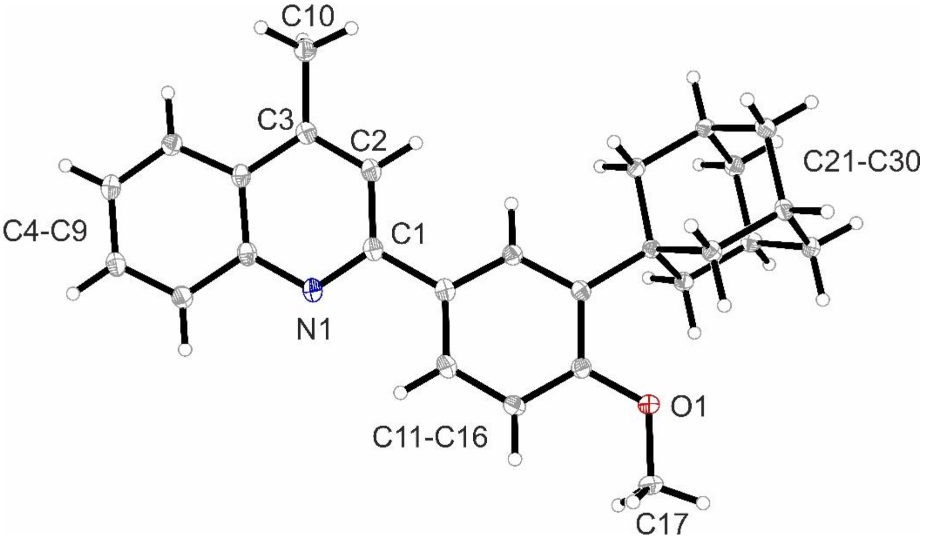

Supplementary Figure 12. Crystal structure of compound **14b**. Thermal ellipsoids are shown at 30% probability.

X-ray crystal structure analysis of 14c: A colourless, prism-shaped crystal was mounted on a loop with perfluoroether oil. The crystals were recrystallised from ethyl acetate. Data for glo10769 were collected from a single crystal in 20.65 hours at 100(2) K on a Bruker D8 VENTURE KAPPA diffractometer with a microfocus sealed tube using a multilayer mirror as monochromator and a Bruker PHOTON III CPAD detector. The diffractometer was equipped with an Oxford Cryostream 1000 low temperature device and used Cu K_α radiation (λ = 1.54178 Å). All data were integrated with SAINT V8.41, yielding 58294 reflections of which 13607 where independent and 83.3% were greater than $2\sigma(F^2)$. A Multi-Scan absorption correction using SADABS 2016/2 was applied. The structure was solved by Intrinsic Phasing methods with SHELXT 2018/2 and refined by full-matrix least-squares methods against F^2 using SHELXL-2019/2. All non-hydrogen atoms were refined with anisotropic displacement parameters. All hydrogen atoms were refined isotropic on calculated positions using a riding model with their U_{iso} values constrained to 1.5 times the U_{eq} of their pivot atoms for terminal sp^3 carbon

atoms and 1.2 times for all other carbon atoms. Crystallographic data for the structures reported in this paper have been deposited with the Cambridge Crystallographic Data Centre. CCDC 2428788 contain the supplementary crystallographic data for this paper. These data can be obtained free of charge from The Cambridge Crystallographic Data Centre via www.ccdc.cam.ac.uk/structures.

CCDC number	2428788
Empirical formula	$C_{44}H_{57}NO_2$
Formula weight	631.9
Temperature [K]	100(2)
Crystal system	orthorhombic
Space group (number)	$P2_12_12$ (18)
a [Å]	16.4551(5)
b [Å]	60.8428(16)
c [Å]	7.2239(2)
α [°]	90
β [°]	90
γ [°]	90
Volume [Å^3]	7232.4(4)
Z	8
ρ_{calc} [gcm^{-3}]	1.161
μ [mm^{-1}]	0.528
F(000)	2752
Crystal size [mm^3]	0.077×0.079×0.151
Crystal colour	colourless
Crystal shape	prism
Radiation	Cu K_α (λ=1.54178 Å)
2θ range [°]	5.56 to 140.24 (0.82 Å)
Index ranges	−20 ≤ h ≤ 20<br>−73 ≤ k ≤ 74<br>−8 ≤ l ≤ 8
Reflections collected	58294
Independent reflections	13607<br>R_{int} = 0.0836<br>R_{sigma} = 0.0805
Completeness to θ = 67.679°	99.8
Data / Restraints / Parameters	13607 / 130 / 916
Goodness-of-fit on F^2	1.066
Final R indexes [I≥2σ(I)]	R_1 = 0.0492<br>wR_2 = 0.1209
Final R indexes [all data]	R_1 = 0.0604<br>wR_2 = 0.1275
Largest peak/hole [eÅ^{-3}]	0.25/−0.19
Flack X parameter	0.05(16)

Supplementary Table 14. Crystal data and structure refinement for **14c**.

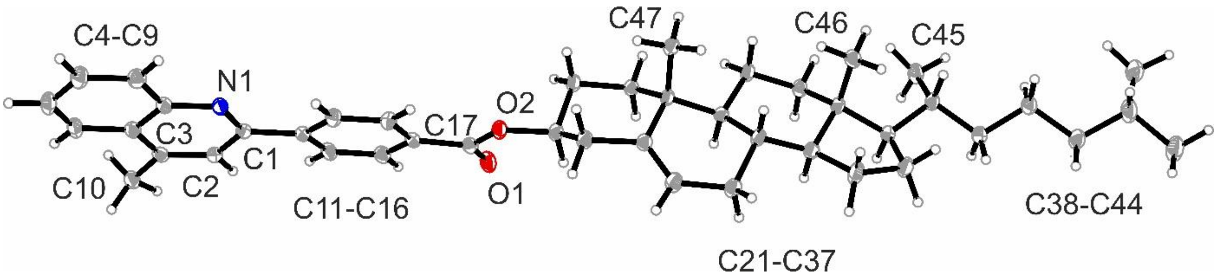

Supplementary Figure 13. Crystal structure of compound **14c**. Only one molecule (molecule named with suffix "A") of two independent molecules found in the asymmetric unit is shown.

8. Copies of ^{1}H, ^{13}C, ^{19}F and ^{11}B NMR spectra

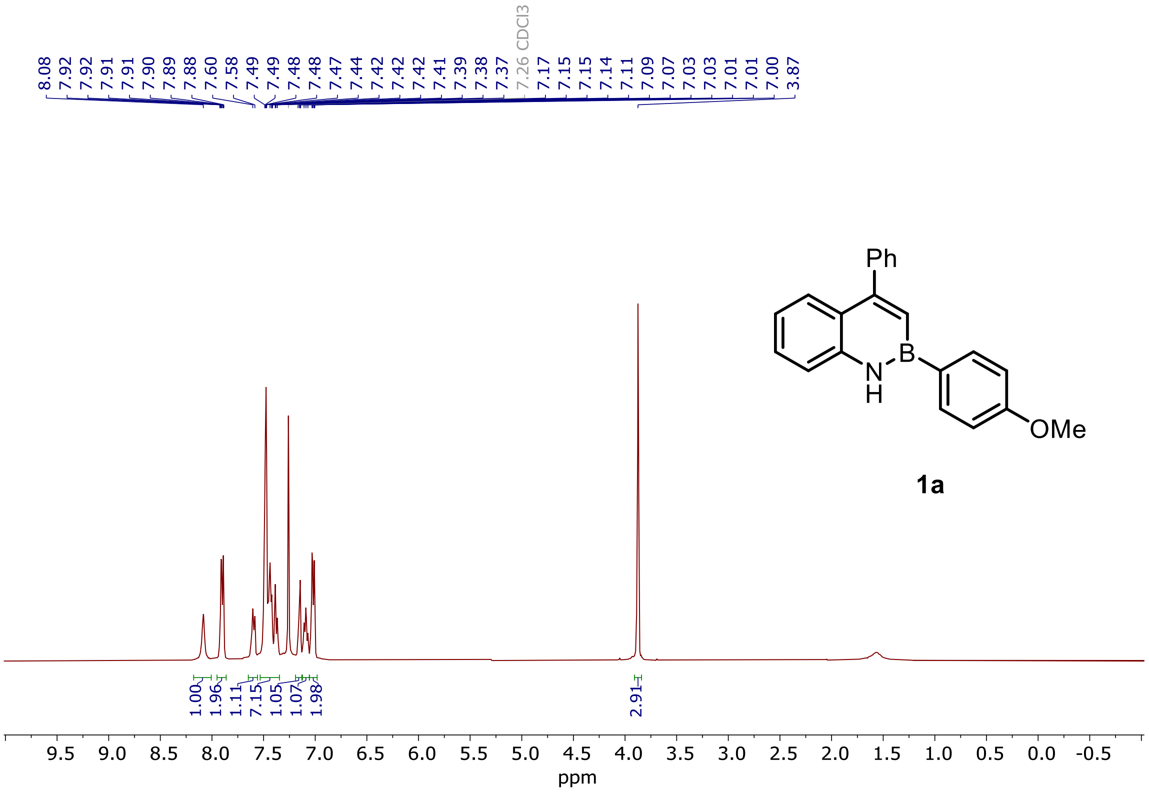

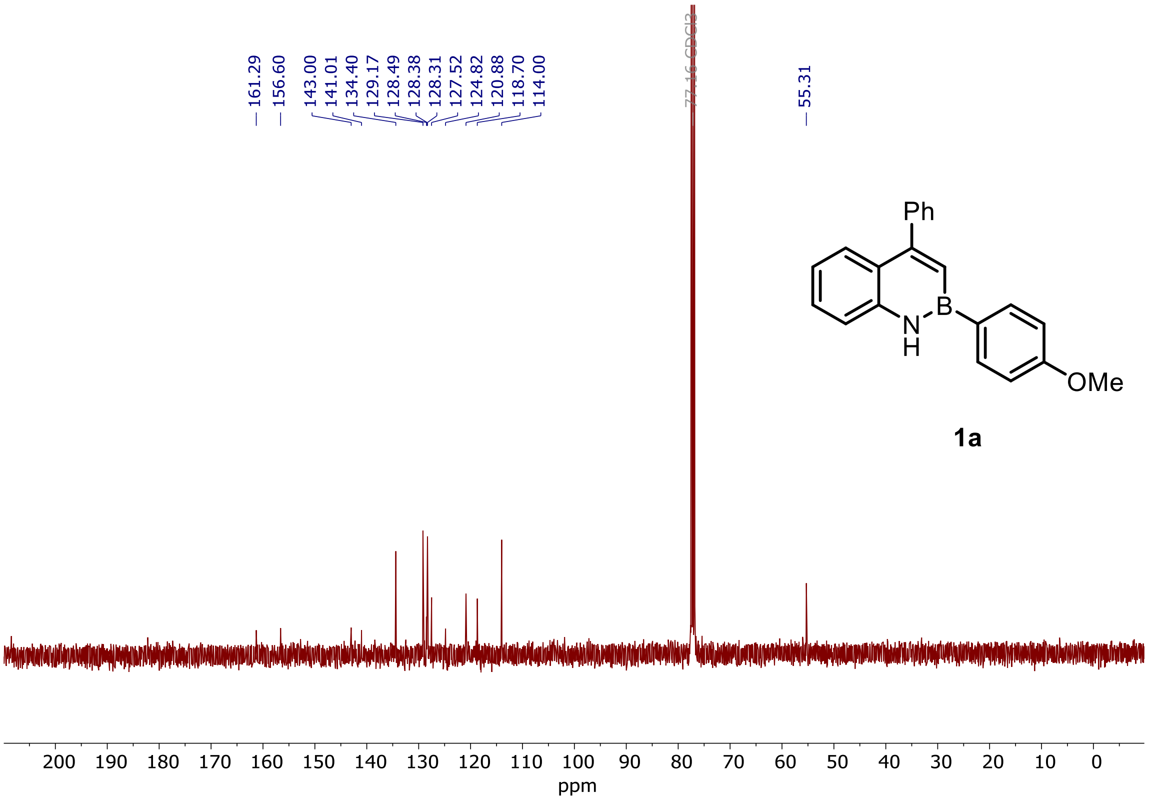

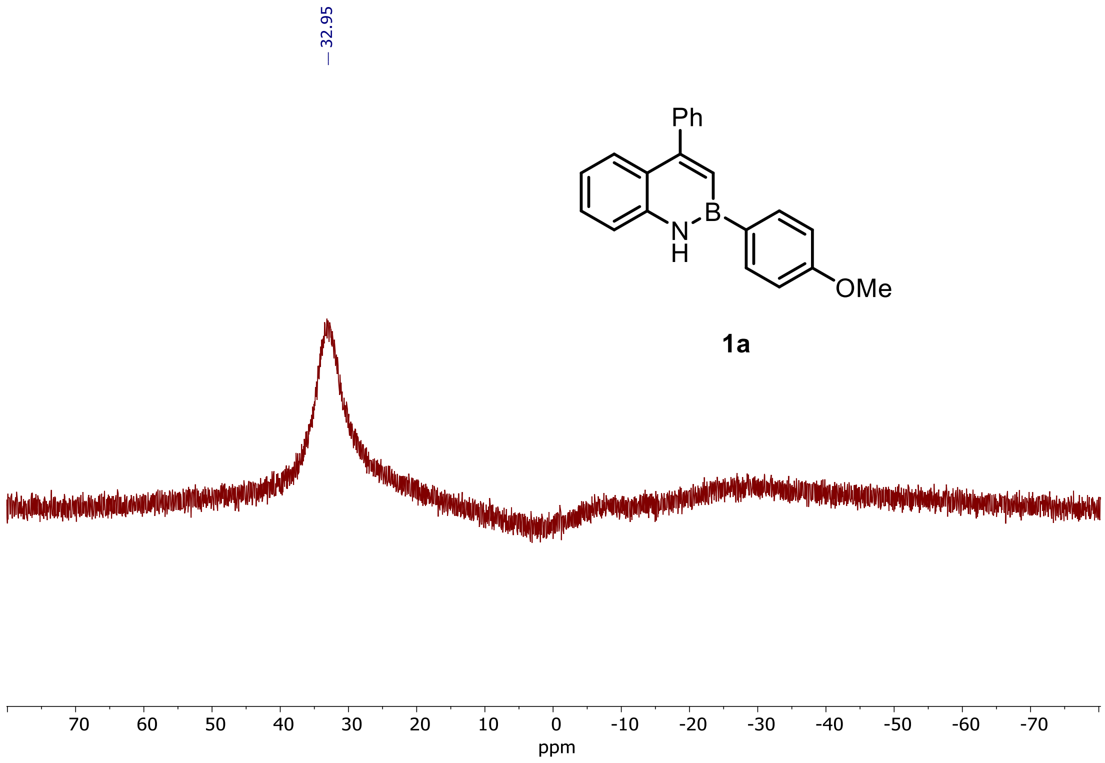
32.95
Ph
N
H
B
OMe
1a
70 60 50 40 30 20 10 0 -10 -20 -30 -40 -50 -60 -70
ppm

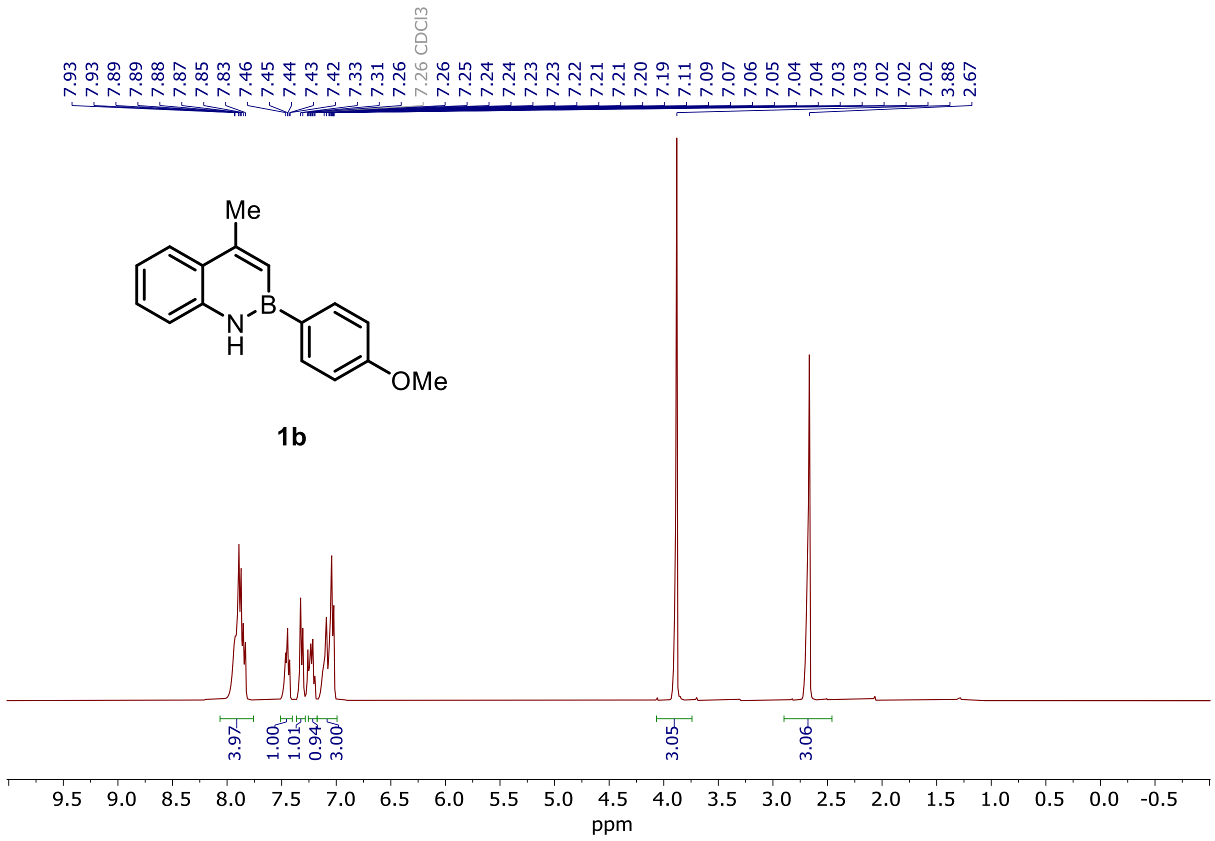
7.93 7.93 7.89 7.89 7.88 7.87 7.85 7.83 7.46 7.45 7.44 7.43 7.42 7.33 7.31 7.26 7.26 CDCl3 7.26 7.25 7.24 7.24 7.23 7.23 7.22 7.21 7.21 7.20 7.19 7.11 7.09 7.07 7.06 7.05 7.04 7.04 7.03 7.03 7.02 7.02 7.02 3.88 2.67
Me
N
H
B
OMe
1b
3.97
1.00
1.01
0.94
3.00
3.05
3.06
9.5 9.0 8.5 8.0 7.5 7.0 6.5 6.0 5.5 5.0 4.5 4.0 3.5 3.0 2.5 2.0 1.5 1.0 0.5 0.0 -0.5
ppm

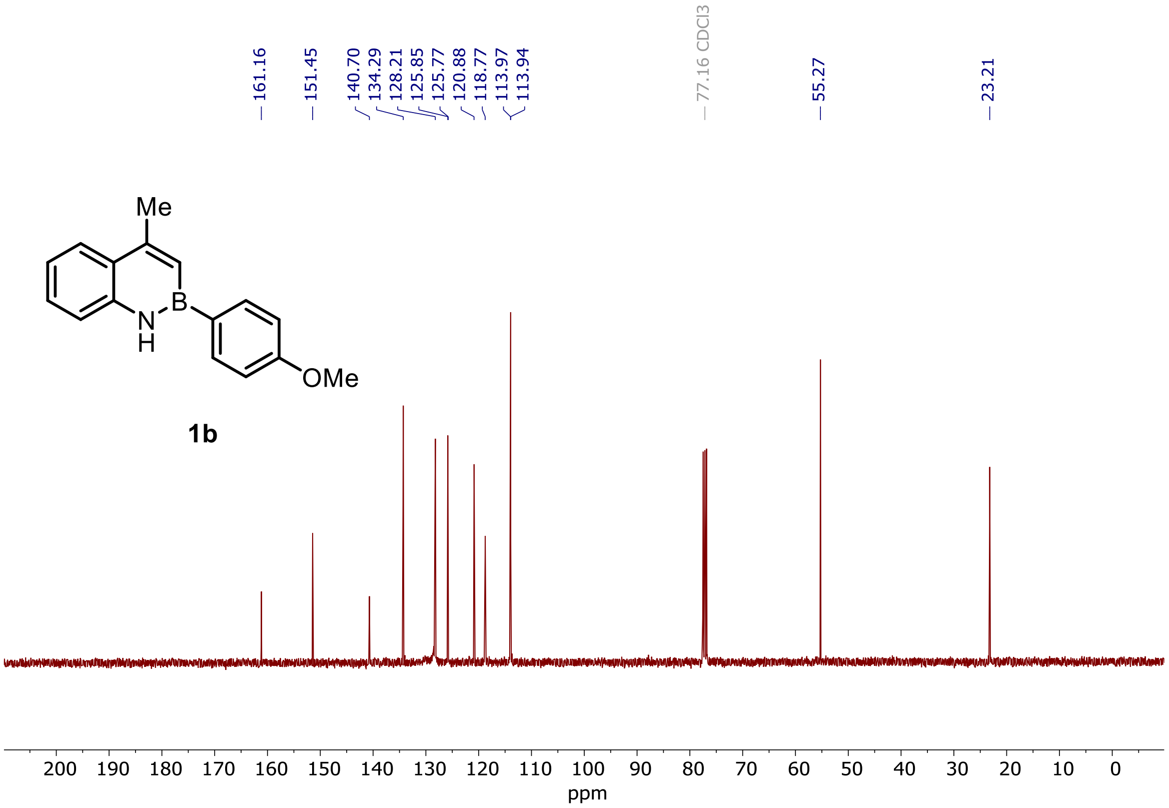
161.16
151.45
140.70
134.29
128.21
125.85
125.77
120.88
118.77
113.97
113.94
77.16 CDCl3
55.27
23.21
Me
N
H
B
OMe
1b
200 190 180 170 160 150 140 130 120 110 100 90 80 70 60 50 40 30 20 10 0
ppm

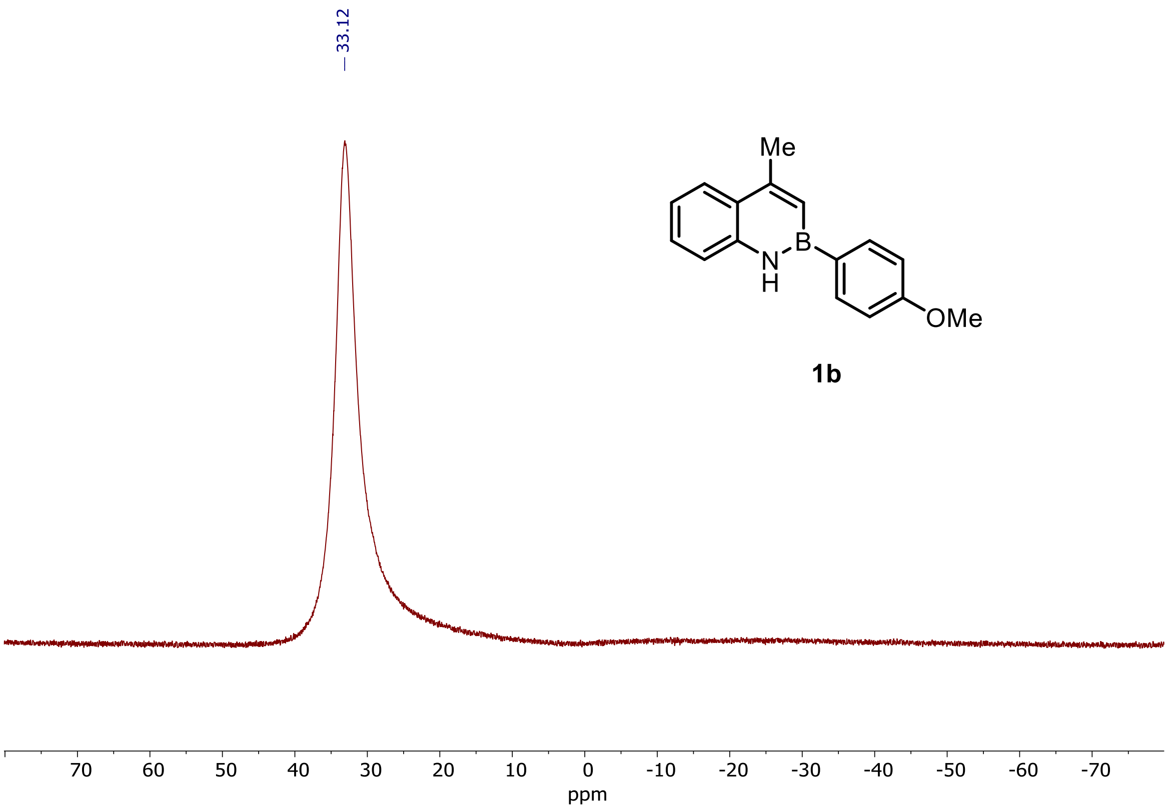
33.12
Me
N
H
B
OMe
1b
70 60 50 40 30 20 10 0 -10 -20 -30 -40 -50 -60 -70
ppm

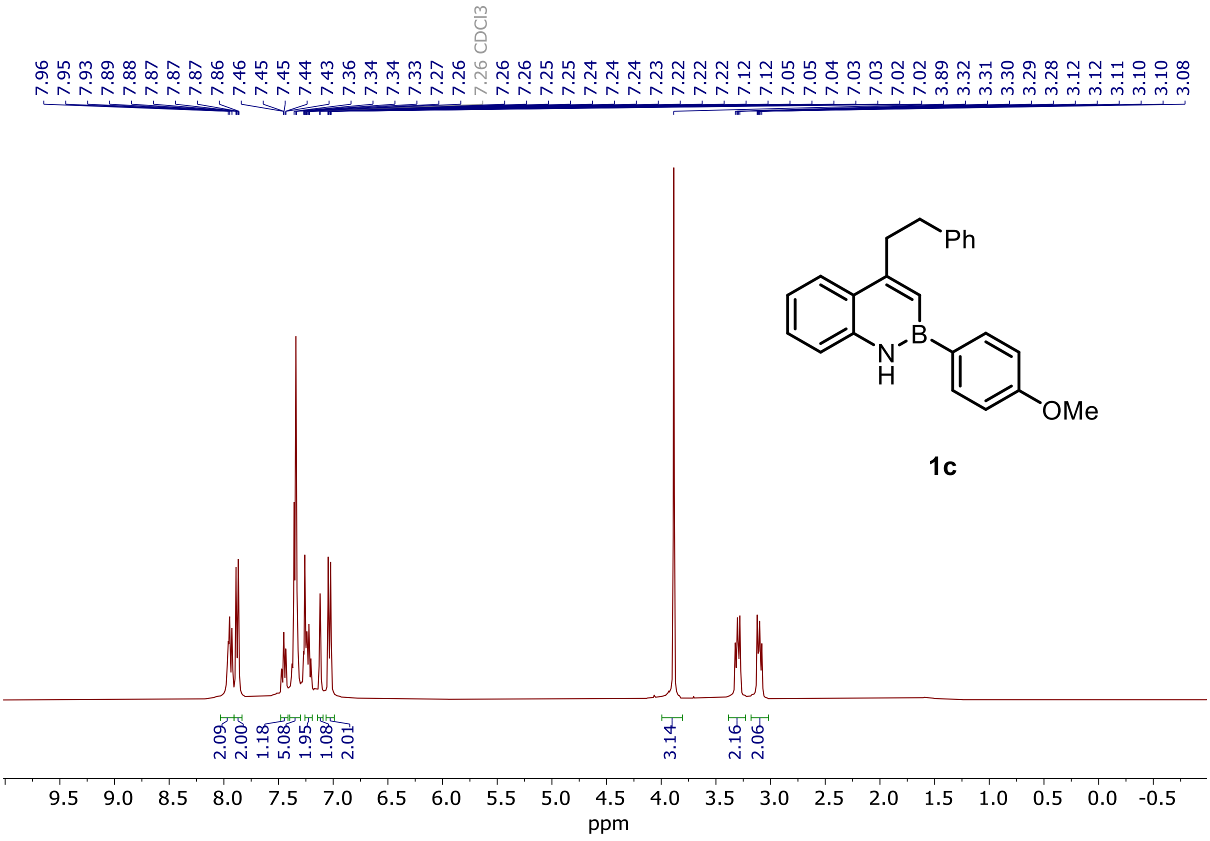

7.96
7.95
7.93
7.89
7.88
7.87
7.87
7.87
7.86
7.46
7.45
7.45
7.44
7.43
7.36
7.34
7.34
7.33
7.27
7.26
7.26 CDCl3
7.26
7.26
7.25
7.25
7.24
7.24
7.24
7.23
7.22
7.22
7.22
7.12
7.12
7.12
7.05
7.05
7.04
7.03
7.03
7.02
7.02
3.89
3.32
3.31
3.30
3.29
3.28
3.12
3.12
3.11
3.10
3.10
3.08
Ph
N
H
B
OMe
1c
2.09
2.00
1.18
5.08
1.95
1.08
2.01
3.14
2.16
2.06
9.5
9.0
8.5
8.0
7.5
7.0
6.5
6.0
5.5
5.0
4.5
4.0
3.5
3.0
2.5
2.0
1.5
1.0
0.5
0.0
-0.5
ppm

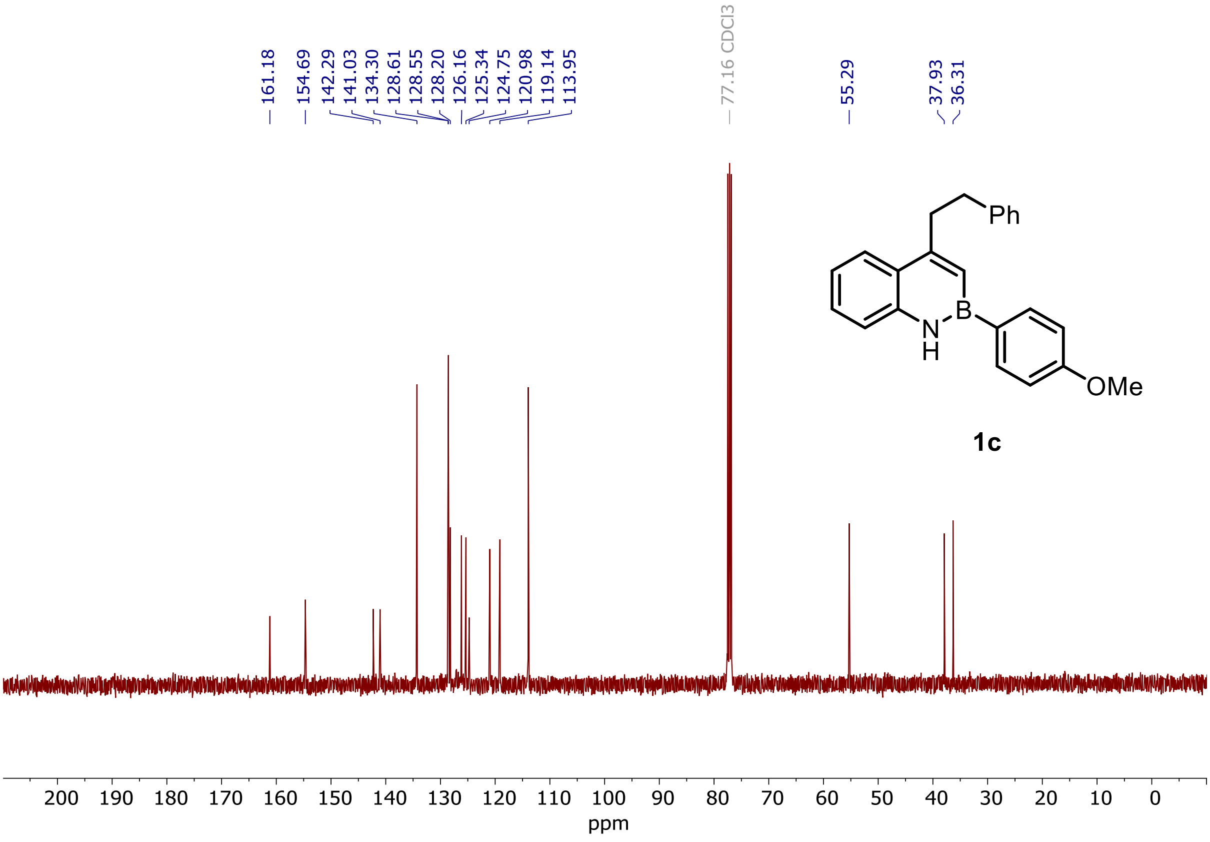

161.18
154.69
142.29
141.03
134.30
128.61
128.55
128.20
126.16
125.34
124.75
120.98
119.14
113.95
77.16 CDCl3
55.29
37.93
36.31
Ph
N
H
B
OMe
1c
200
190
180
170
160
150
140
130
120
110
100
90
80
70
60
50
40
30
20
10
0
ppm

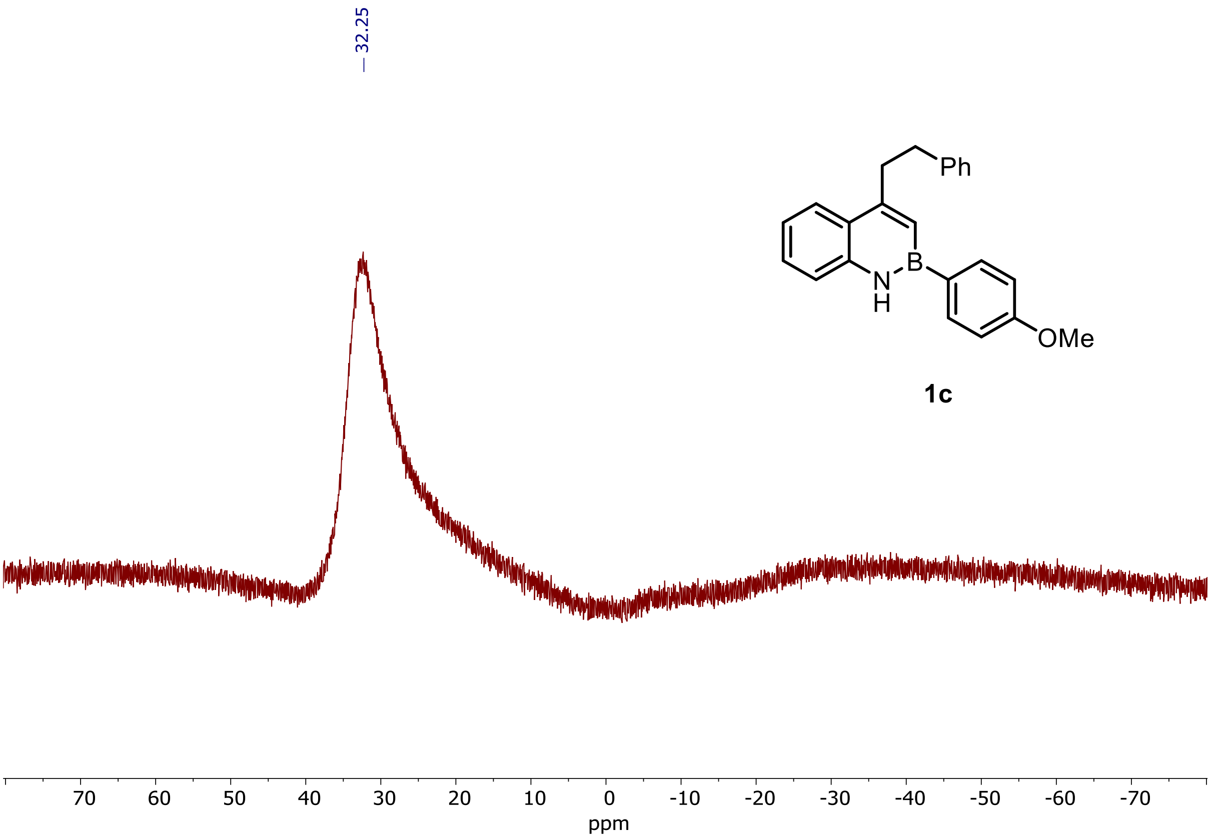

32.25
Ph
N
B
H
OMe
1c
70 60 50 40 30 20 10 0 -10 -20 -30 -40 -50 -60 -70
ppm

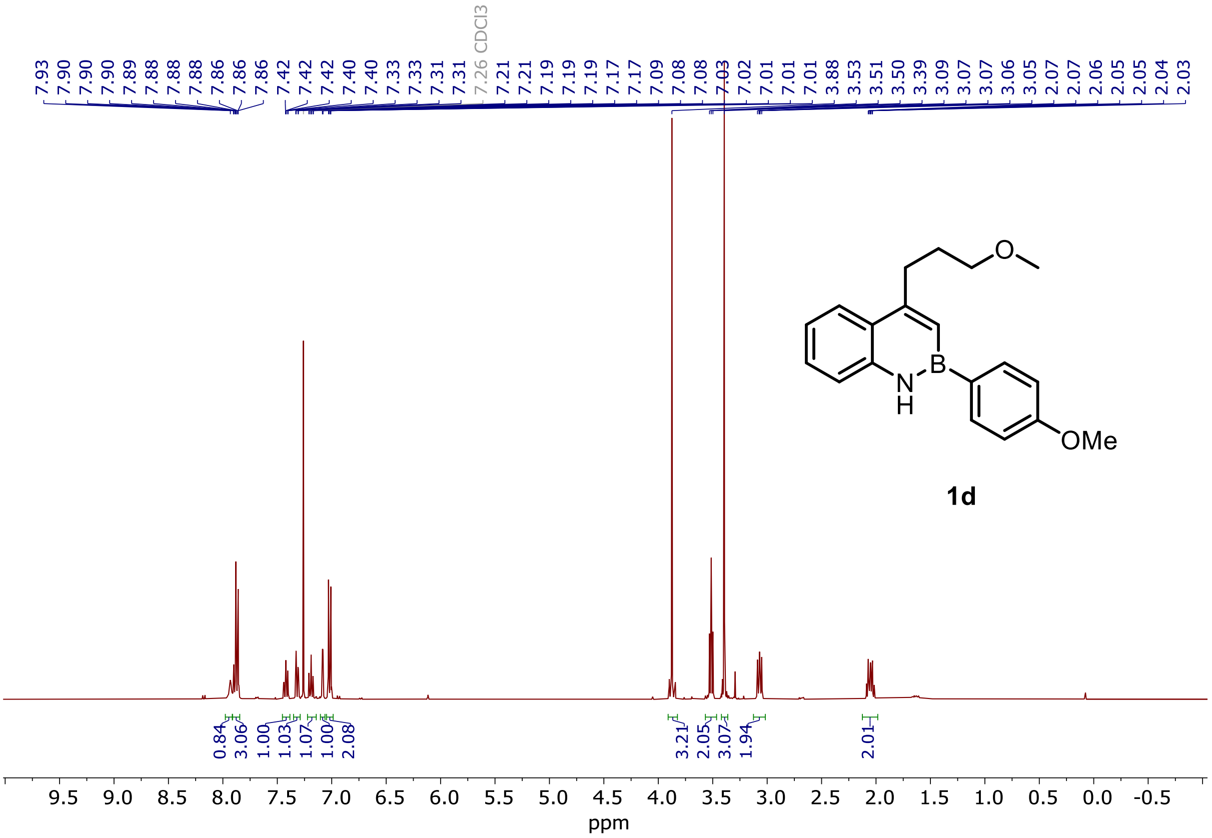

7.93 7.90 7.90 7.90 7.89 7.88 7.88 7.88 7.86 7.86 7.86 7.42 7.42 7.42 7.40 7.40 7.33 7.33 7.31 7.31 7.26 CDCl3 7.21 7.21 7.19 7.19 7.19 7.17 7.17 7.09 7.08 7.08 7.03 7.02 7.01 7.01 7.01 3.88 3.53 3.51 3.50 3.39 3.09 3.07 3.07 3.06 3.05 2.07 2.07 2.06 2.05 2.05 2.04 2.03
O
N
B
H
OMe
1d
0.84 3.06 1.00 1.03 1.07 1.00 2.08 3.21 2.05 3.07 1.94 2.01
9.5 9.0 8.5 8.0 7.5 7.0 6.5 6.0 5.5 5.0 4.5 4.0 3.5 3.0 2.5 2.0 1.5 1.0 0.5 0.0 -0.5
ppm

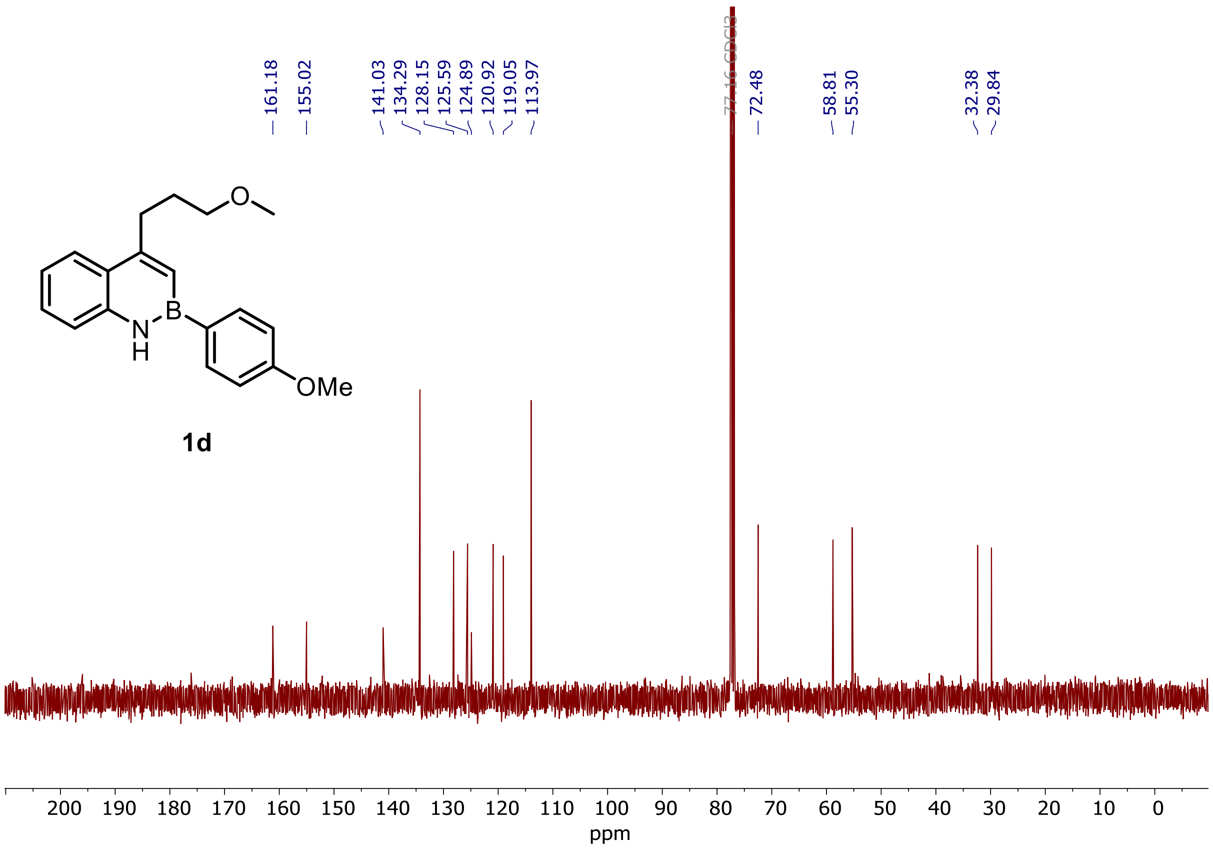
161.18
155.02
141.03
134.29
128.15
125.59
124.89
120.92
119.05
113.97
77.16 CDCl3
72.48
58.81
55.30
32.38
29.84
N
H
B
O
OMe
1d
200
190
180
170
160
150
140
130
120
110
100
90
80
70
60
50
40
30
20
10
0
ppm

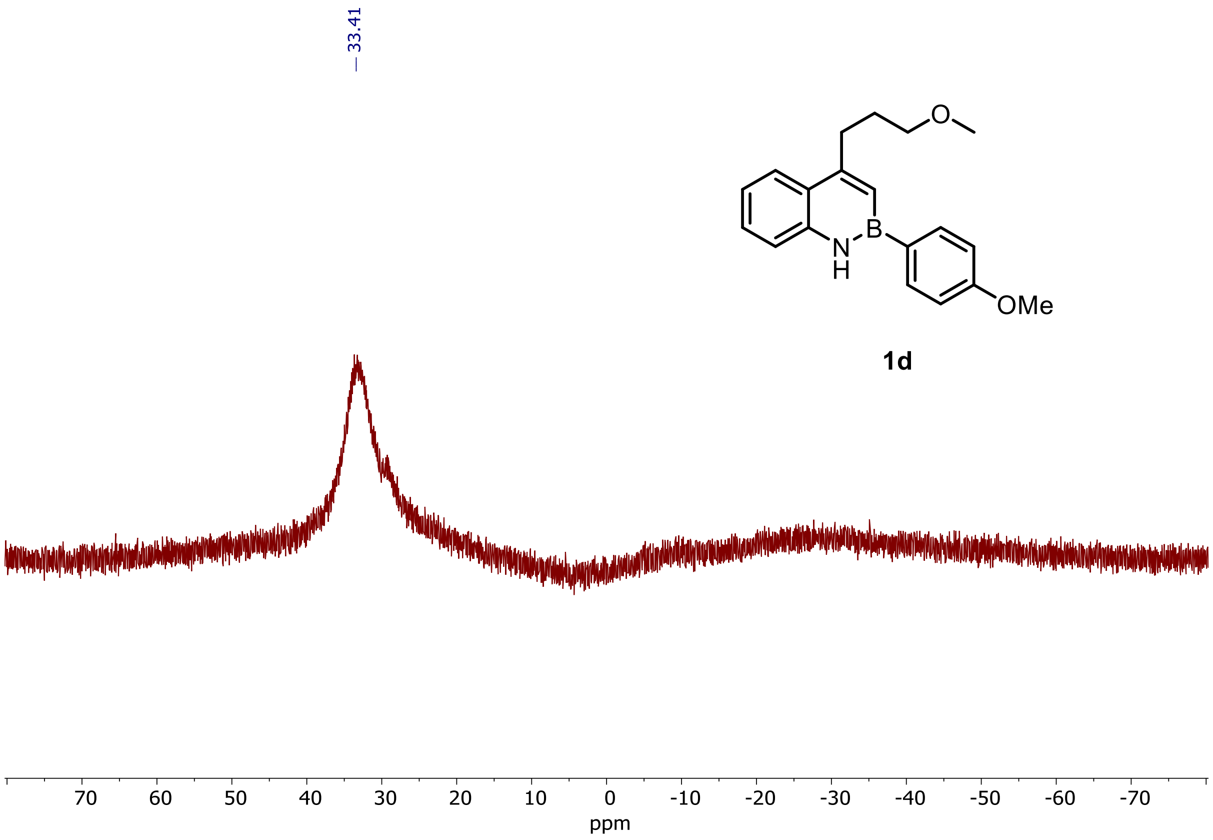
33.41
N
H
B
O
OMe
1d
70
60
50
40
30
20
10
0
-10
-20
-30
-40
-50
-60
-70
ppm

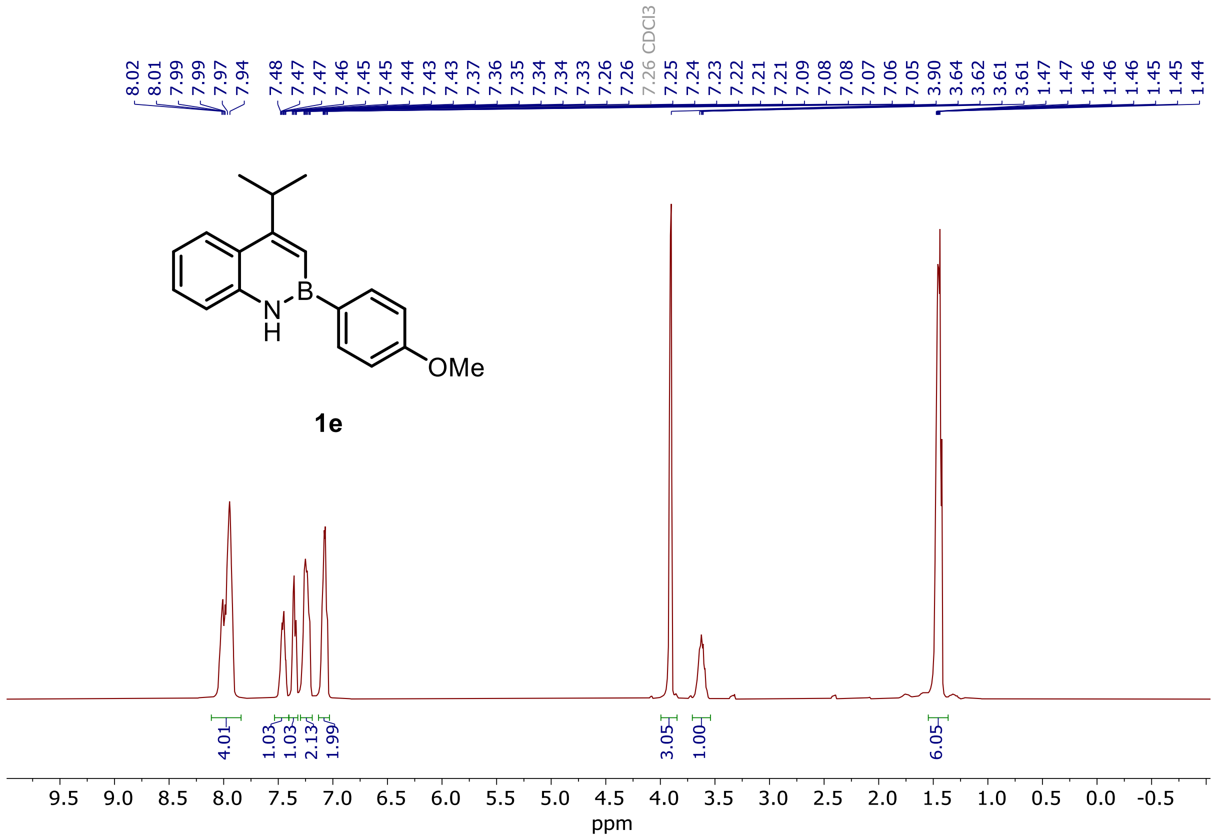
8.02
8.01
7.99
7.99
7.97
7.94
7.48
7.47
7.47
7.46
7.45
7.45
7.44
7.43
7.43
7.37
7.36
7.35
7.34
7.34
7.33
7.26
7.26
7.26 CDCl3
7.25
7.24
7.23
7.22
7.21
7.21
7.09
7.08
7.07
7.06
7.05
3.90
3.64
3.62
3.61
3.61
1.47
1.47
1.46
1.46
1.46
1.45
1.45
1.44
N
B
H
OMe
1e
4.01
1.03
1.03
2.13
1.99
3.05
1.00
6.05
9.5 9.0 8.5 8.0 7.5 7.0 6.5 6.0 5.5 5.0 4.5 4.0 3.5 3.0 2.5 2.0 1.5 1.0 0.5 0.0 -0.5
ppm

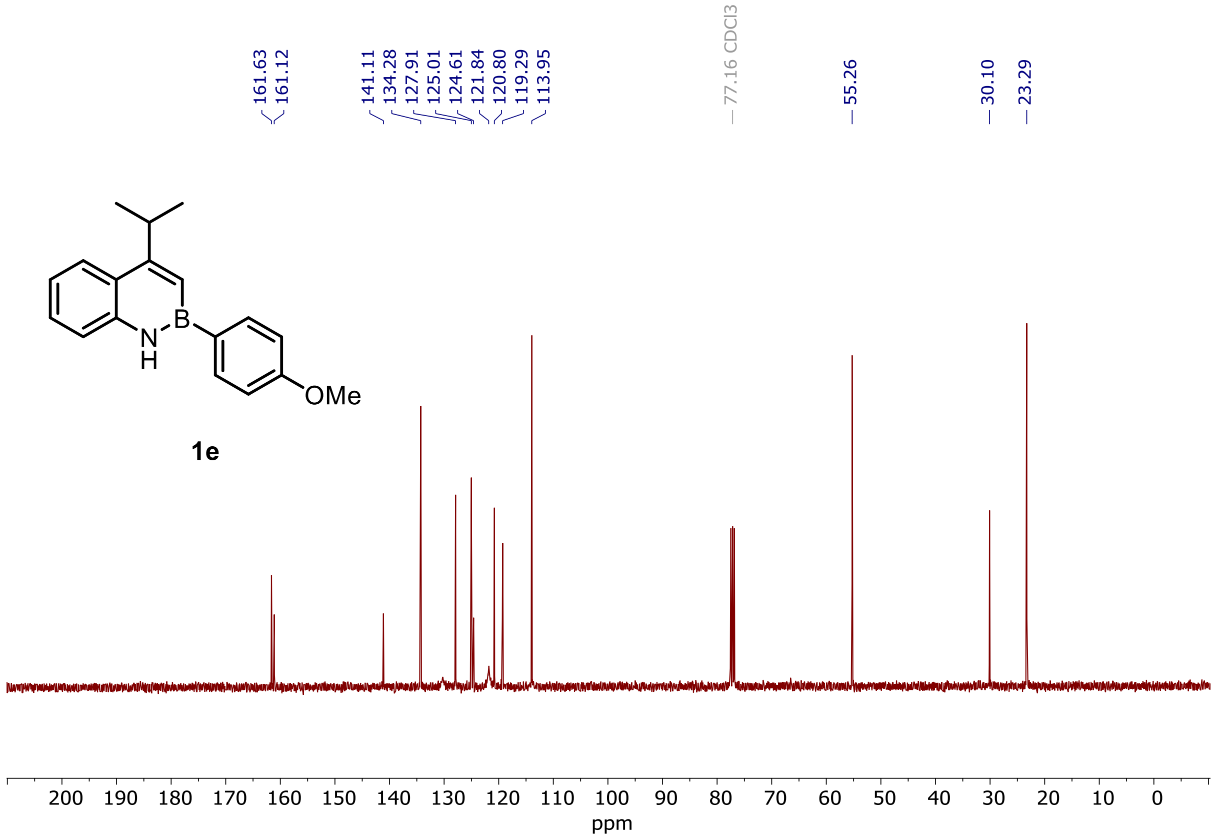
161.63
161.12
141.11
134.28
127.91
125.01
124.61
121.84
120.80
119.29
113.95
77.16 CDCl3
55.26
30.10
23.29
N
B
H
OMe
1e
200 190 180 170 160 150 140 130 120 110 100 90 80 70 60 50 40 30 20 10 0
ppm

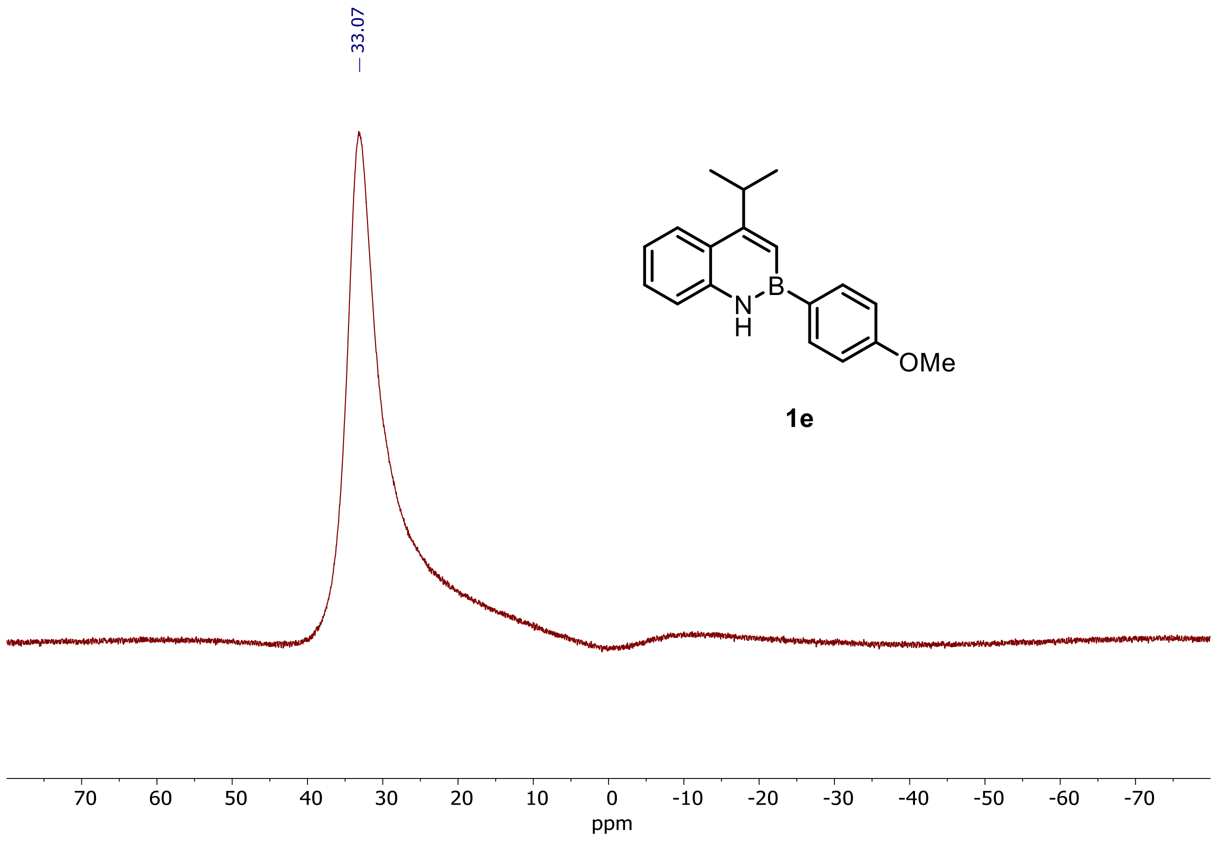

33.07
OMe
1e
70 60 50 40 30 20 10 0 -10 -20 -30 -40 -50 -60 -70
ppm

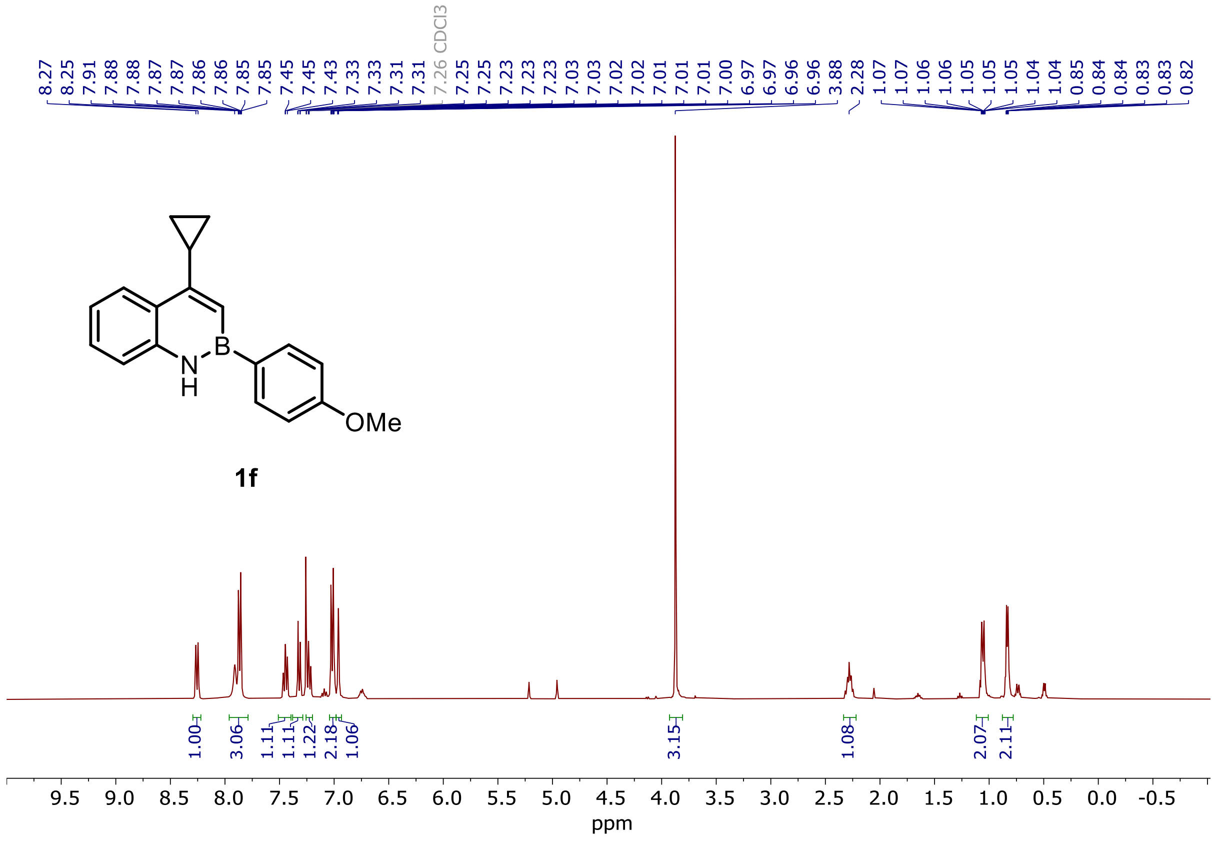

8.27 8.25 7.91 7.88 7.88 7.87 7.87 7.86 7.86 7.85 7.85 7.45 7.45 7.43 7.33 7.33 7.31 7.31 7.26 CDCl3 7.25 7.25 7.23 7.23 7.23 7.03 7.03 7.02 7.02 7.01 7.01 7.01 7.00 6.97 6.97 6.96 6.96 3.88 2.28 1.07 1.07 1.06 1.06 1.05 1.05 1.05 1.05 1.04 1.04 0.85 0.84 0.84 0.83 0.83 0.82
OMe
1f
1.00 3.06 1.11 1.11 1.22 2.18 1.06 3.15 1.08 2.07 2.11
9.5 9.0 8.5 8.0 7.5 7.0 6.5 6.0 5.5 5.0 4.5 4.0 3.5 3.0 2.5 2.0 1.5 1.0 0.5 0.0 -0.5
ppm

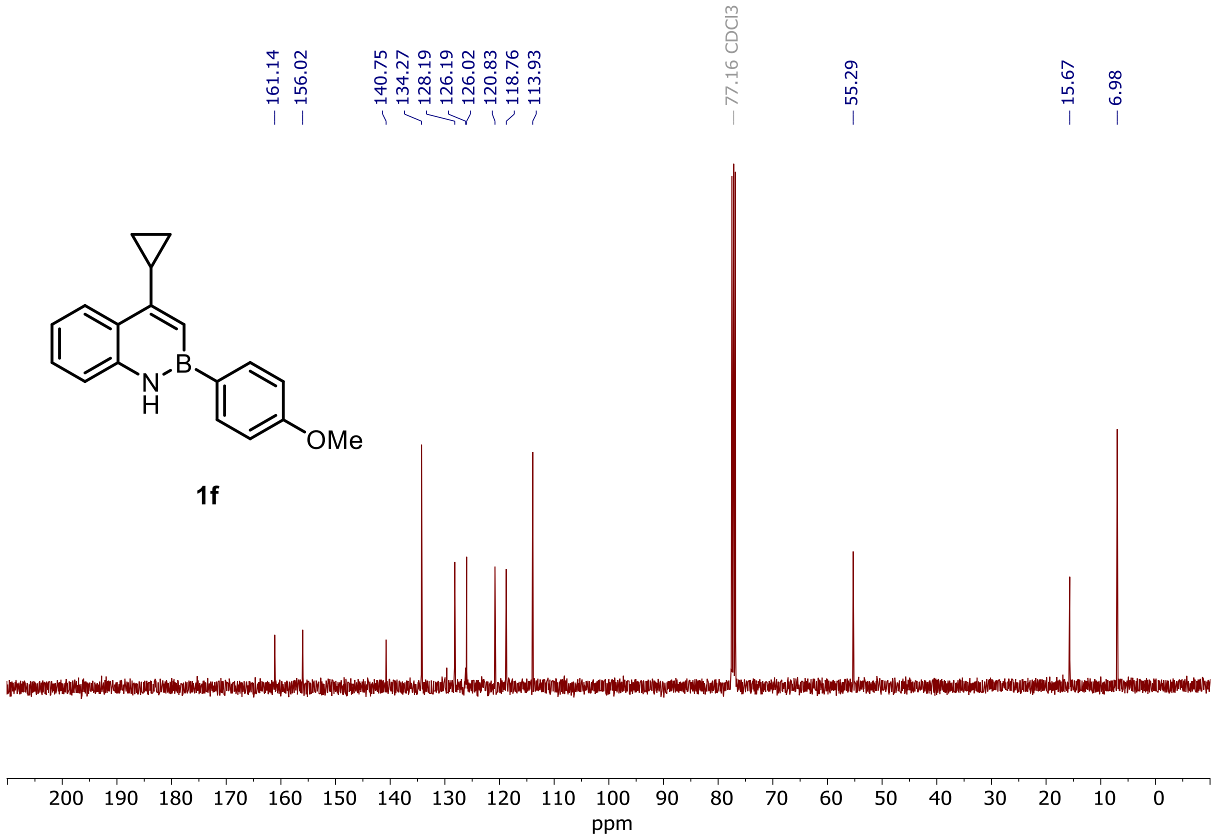
161.14
156.02
140.75
134.27
128.19
126.19
126.02
120.83
118.76
113.93
77.16 CDCl3
55.29
15.67
6.98
B
N
H
OMe
200 190 180 170 160 150 140 130 120 110 100 90 80 70 60 50 40 30 20 10 0
ppm

1f

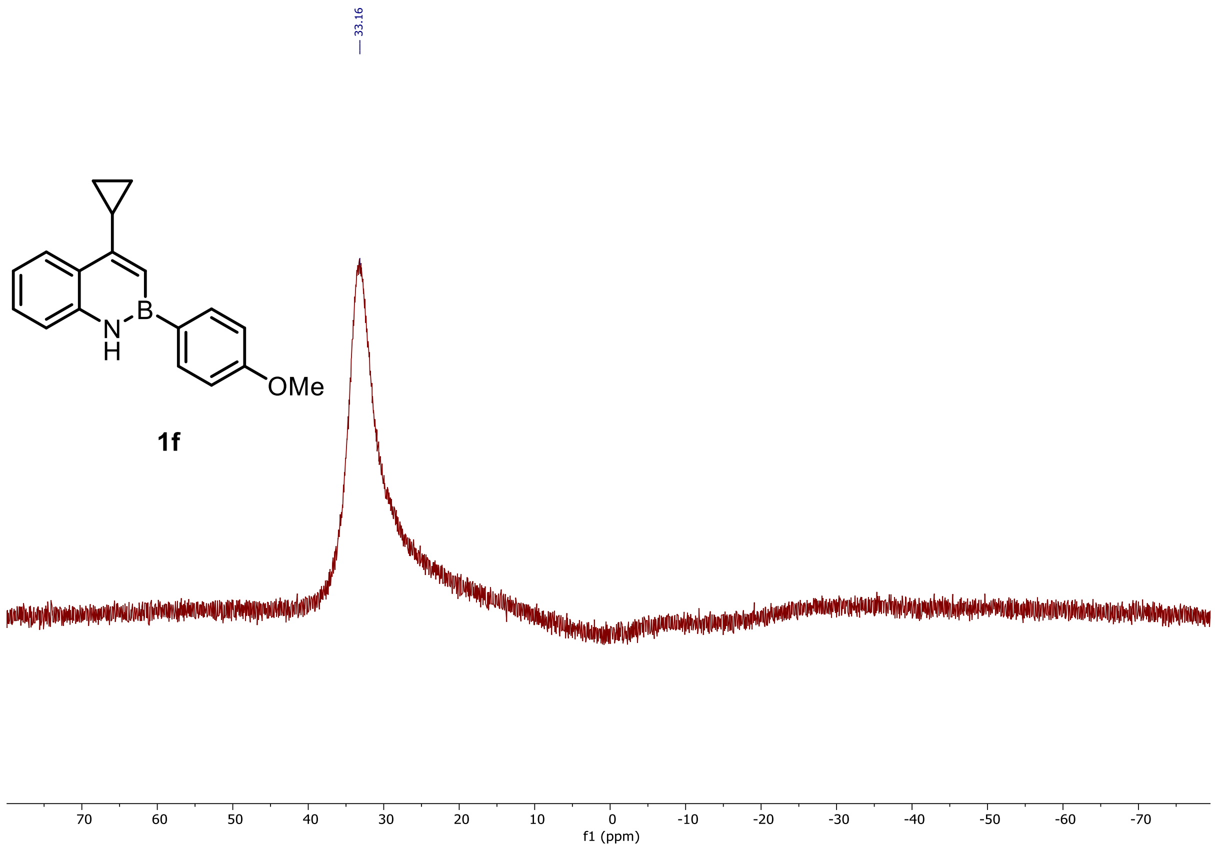
33.16
B
N
H
OMe
70 60 50 40 30 20 10 0 -10 -20 -30 -40 -50 -60 -70
f1 (ppm)

1f

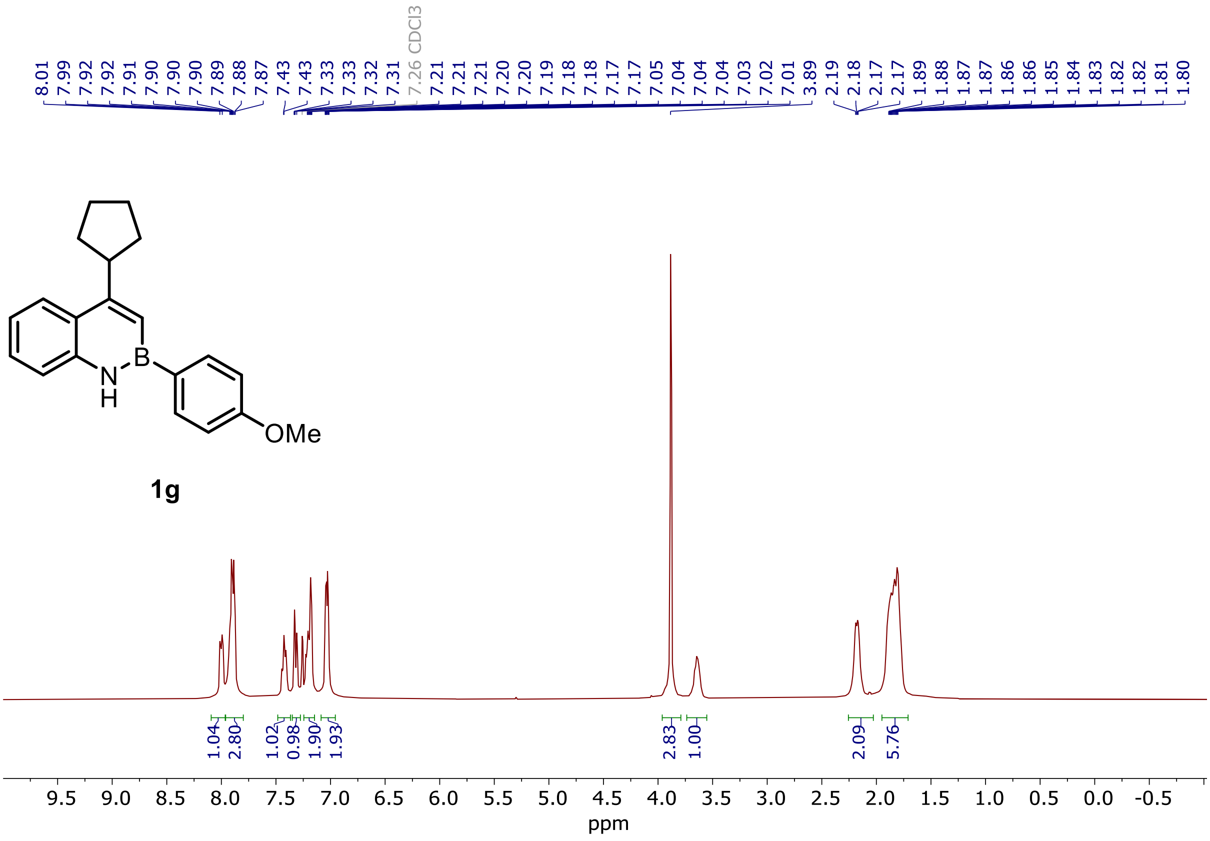
8.01 7.99 7.92 7.92 7.91 7.90 7.90 7.90 7.89 7.88 7.87 7.43 7.43 7.33 7.33 7.32 7.31 7.26 CDCl3 7.21 7.21 7.21 7.20 7.20 7.19 7.18 7.18 7.17 7.17 7.05 7.04 7.04 7.04 7.03 7.02 7.01 3.89 2.19 2.18 2.17 2.17 1.89 1.88 1.87 1.87 1.86 1.86 1.85 1.84 1.83 1.82 1.82 1.81 1.80
N
H
B
OMe
1g
1.04 2.80 1.02 0.98 1.90 1.93 2.83 1.00 2.09 5.76
9.5 9.0 8.5 8.0 7.5 7.0 6.5 6.0 5.5 5.0 4.5 4.0 3.5 3.0 2.5 2.0 1.5 1.0 0.5 0.0 -0.5
ppm

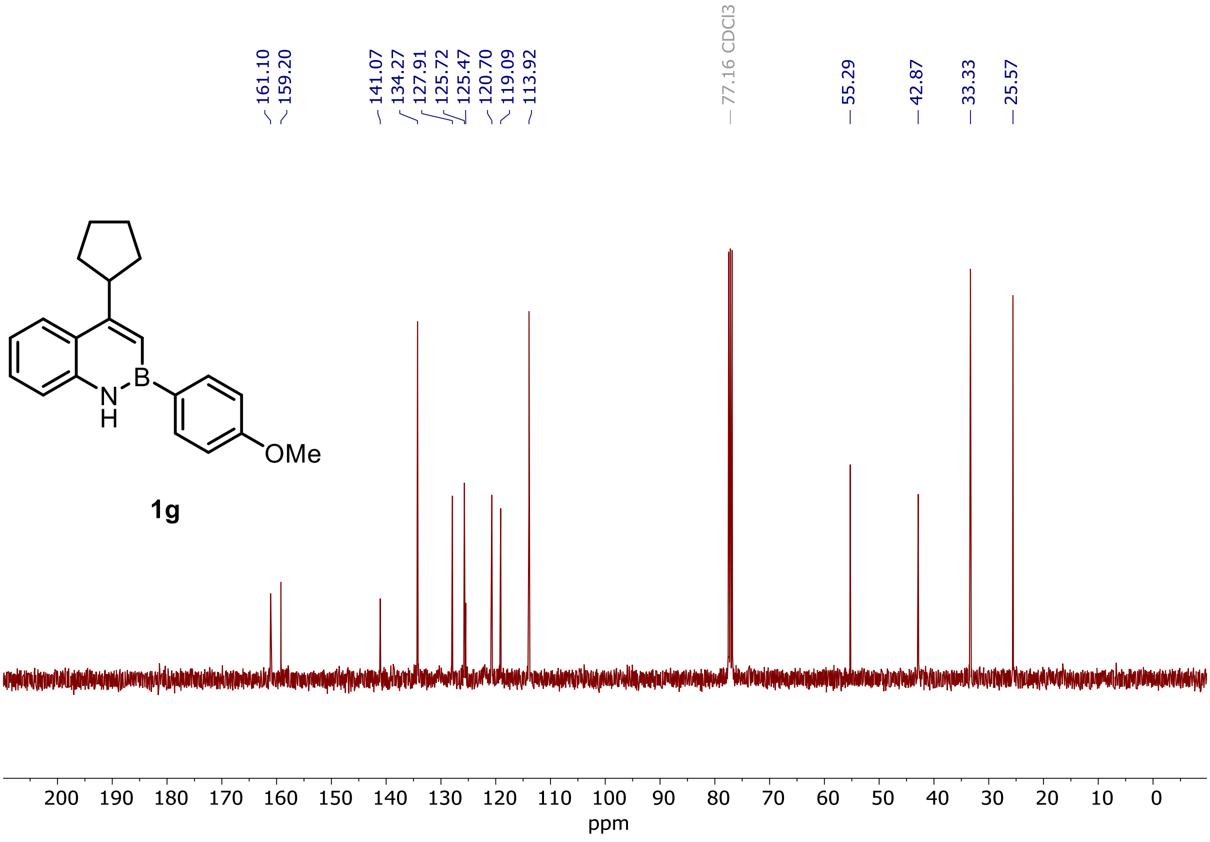
161.10 159.20 141.07 134.27 127.91 125.72 125.47 120.70 119.09 113.92 77.16 CDCl3 55.29 42.87 33.33 25.57
N
H
B
OMe
1g
200 190 180 170 160 150 140 130 120 110 100 90 80 70 60 50 40 30 20 10 0
ppm

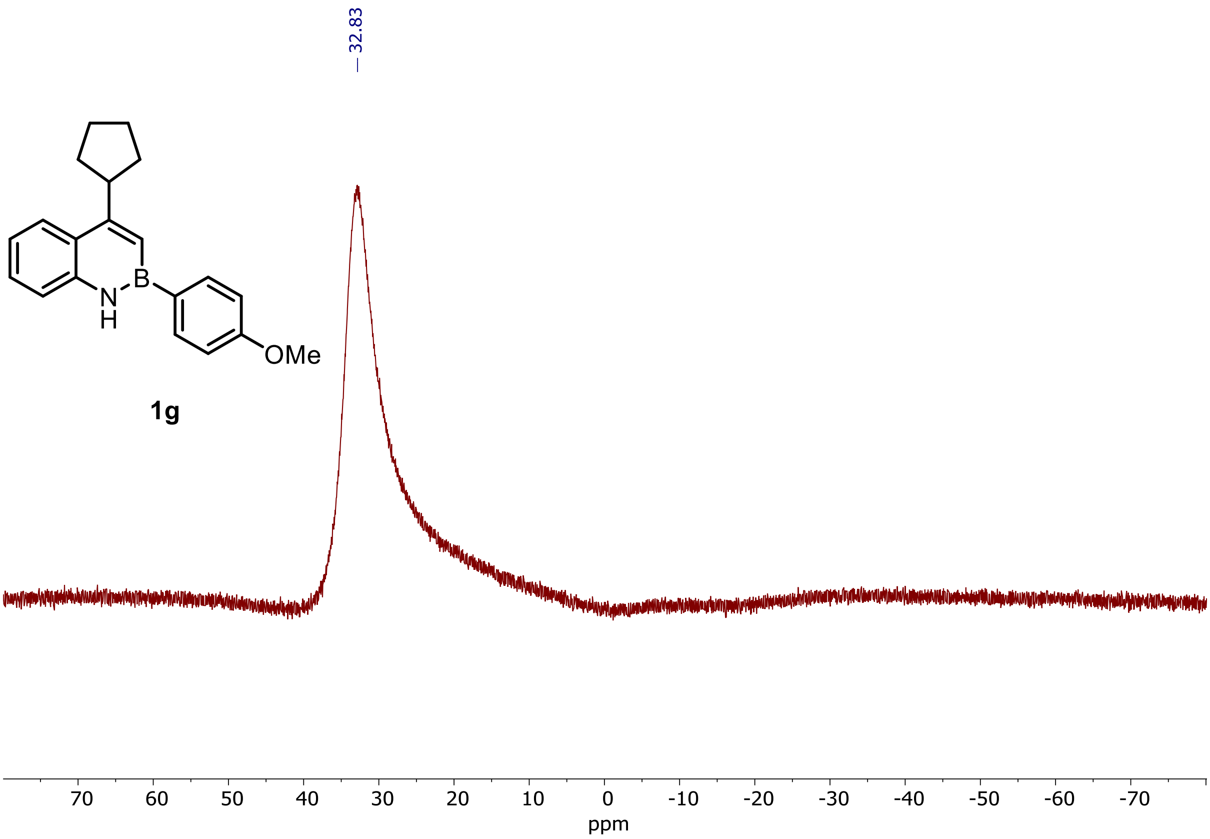

32.83
OMe
1g
70 60 50 40 30 20 10 0 -10 -20 -30 -40 -50 -60 -70
ppm

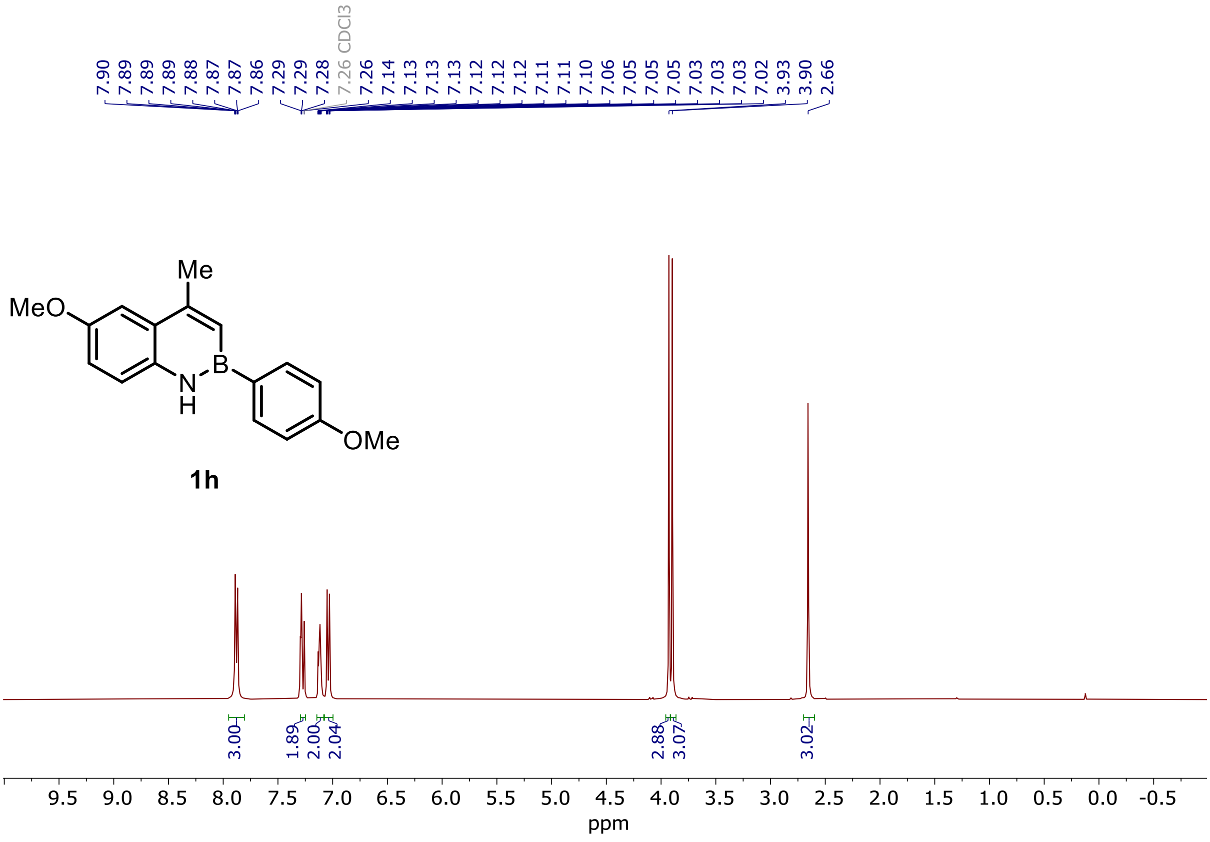

7.90 7.89 7.89 7.89 7.88 7.87 7.87 7.86 7.29 7.29 7.28 7.26 CDCl3 7.26 7.14 7.13 7.13 7.13 7.12 7.12 7.12 7.11 7.11 7.10 7.06 7.05 7.05 7.05 7.03 7.03 7.03 7.02 3.93 3.90 2.66
Me
MeO
OMe
1h
3.00 1.89 2.00 2.04 2.88 3.07 3.02
9.5 9.0 8.5 8.0 7.5 7.0 6.5 6.0 5.5 5.0 4.5 4.0 3.5 3.0 2.5 2.0 1.5 1.0 0.5 0.0 -0.5
ppm

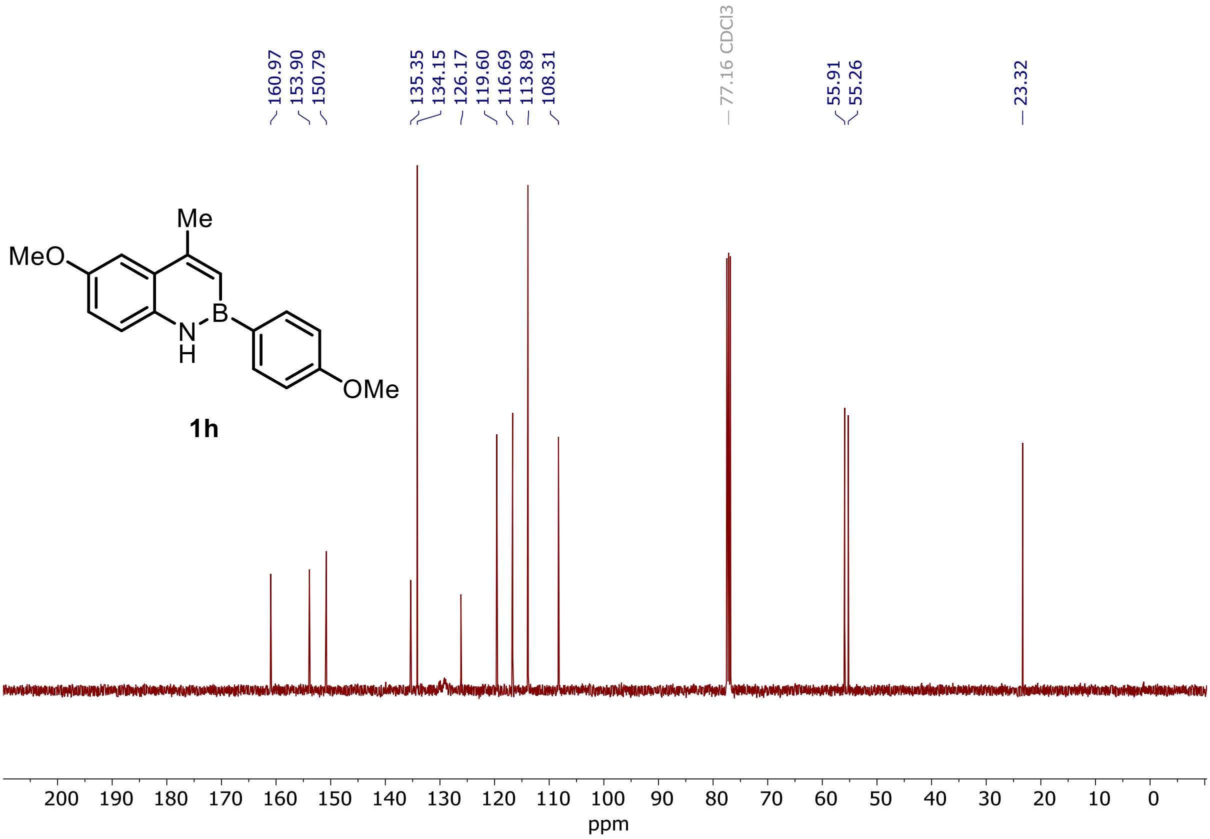
160.97
153.90
150.79
135.35
134.15
126.17
119.60
116.69
113.89
108.31
77.16 CDCl3
55.91
55.26
23.32
MeO
Me
B
N
H
OMe
1h
200 190 180 170 160 150 140 130 120 110 100 90 80 70 60 50 40 30 20 10 0
ppm

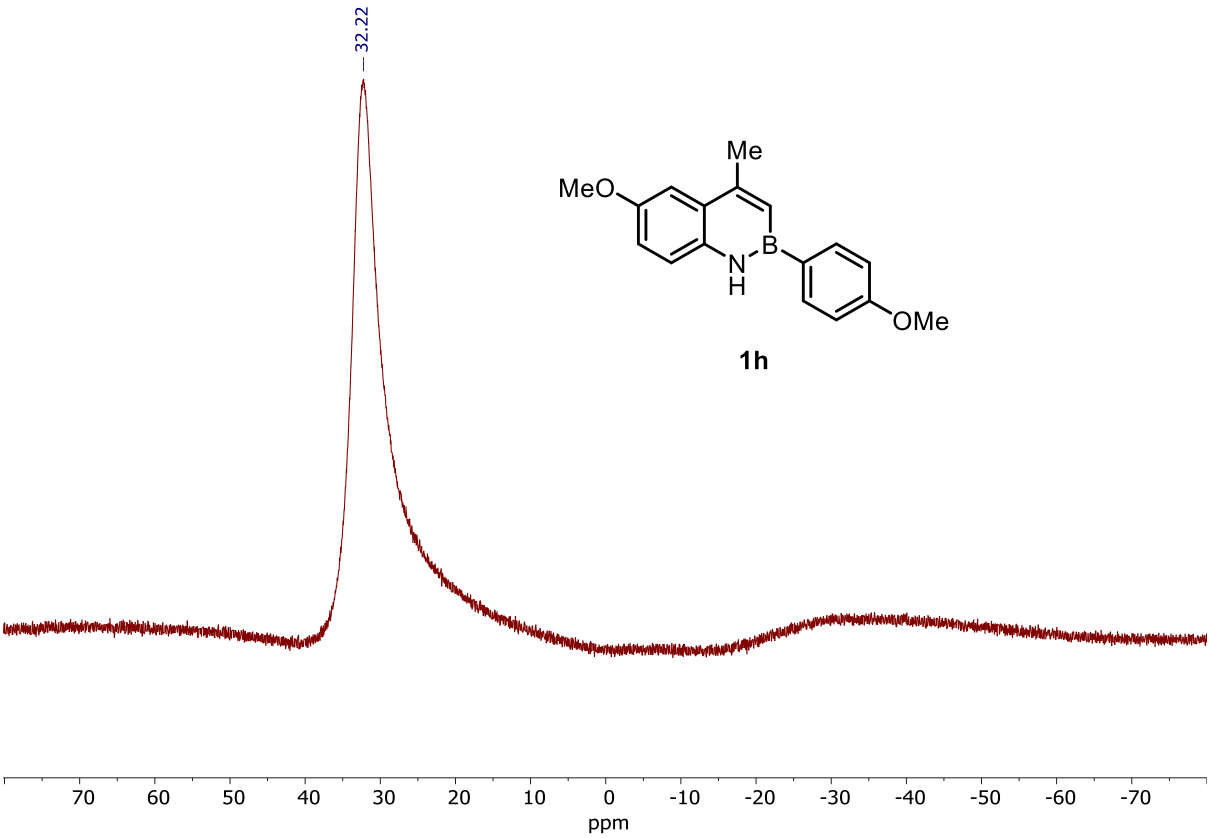
32.22
MeO
Me
B
N
H
OMe
1h
70 60 50 40 30 20 10 0 -10 -20 -30 -40 -50 -60 -70
ppm

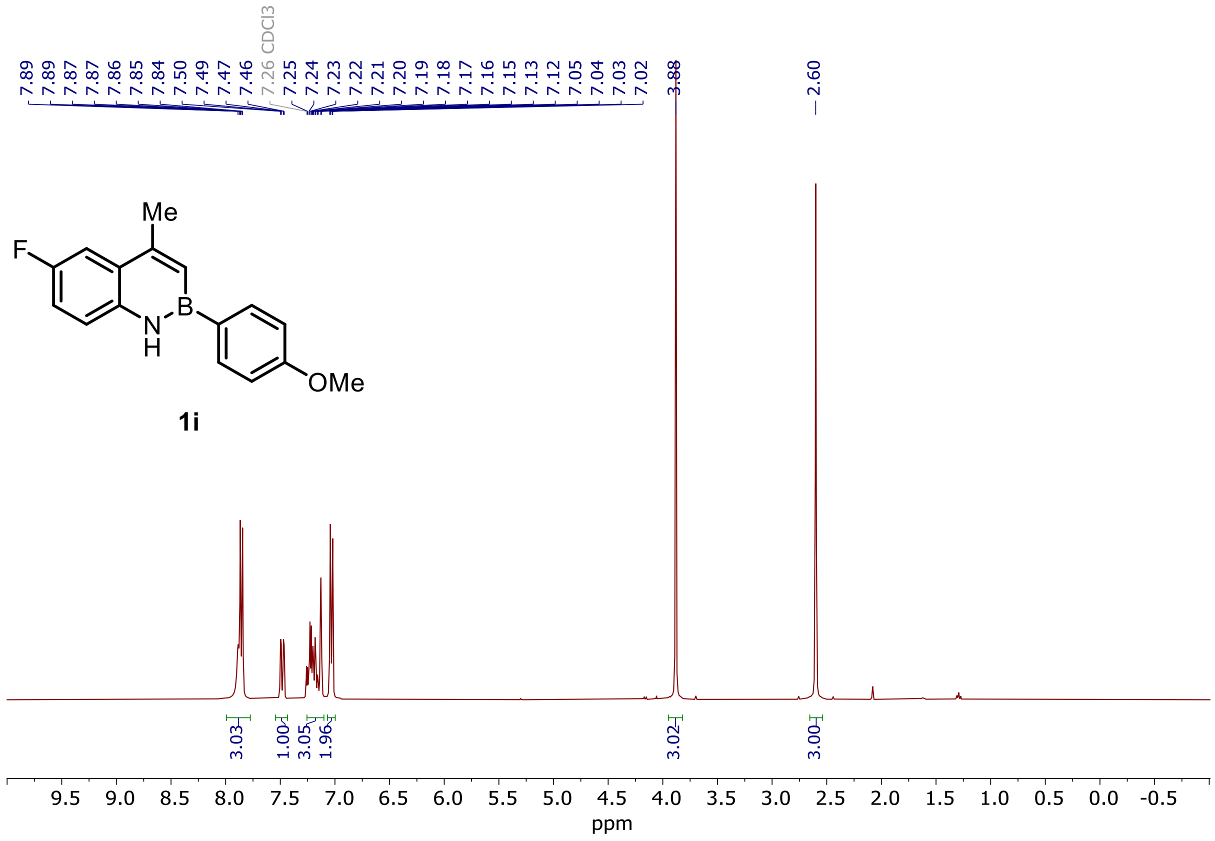
7.89
7.89
7.87
7.87
7.86
7.85
7.84
7.50
7.49
7.47
7.46
7.26 CDCl3
7.25
7.24
7.23
7.22
7.21
7.20
7.19
7.18
7.17
7.16
7.15
7.13
7.12
7.05
7.04
7.03
7.02
3.88
2.60
Me
F
B
N
H
OMe
1i
3.03
1.00
3.05
1.96
3.02
3.00
9.5 9.0 8.5 8.0 7.5 7.0 6.5 6.0 5.5 5.0 4.5 4.0 3.5 3.0 2.5 2.0 1.5 1.0 0.5 0.0 -0.5
ppm

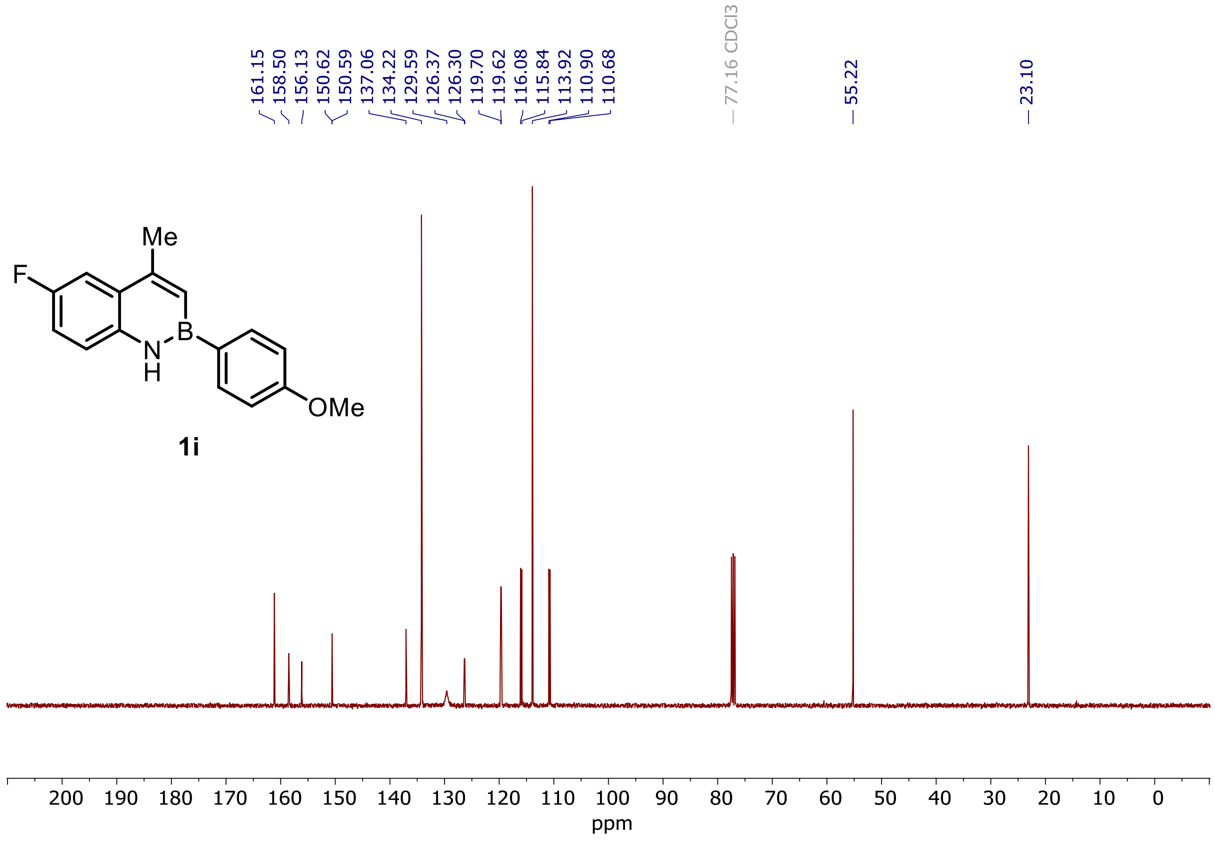
161.15
158.50
156.13
150.62
150.59
137.06
134.22
129.59
126.37
126.30
119.70
119.62
116.08
115.84
113.92
110.90
110.68
77.16 CDCl3
55.22
23.10
Me
F
B
N
H
OMe
1i
200 190 180 170 160 150 140 130 120 110 100 90 80 70 60 50 40 30 20 10 0
ppm

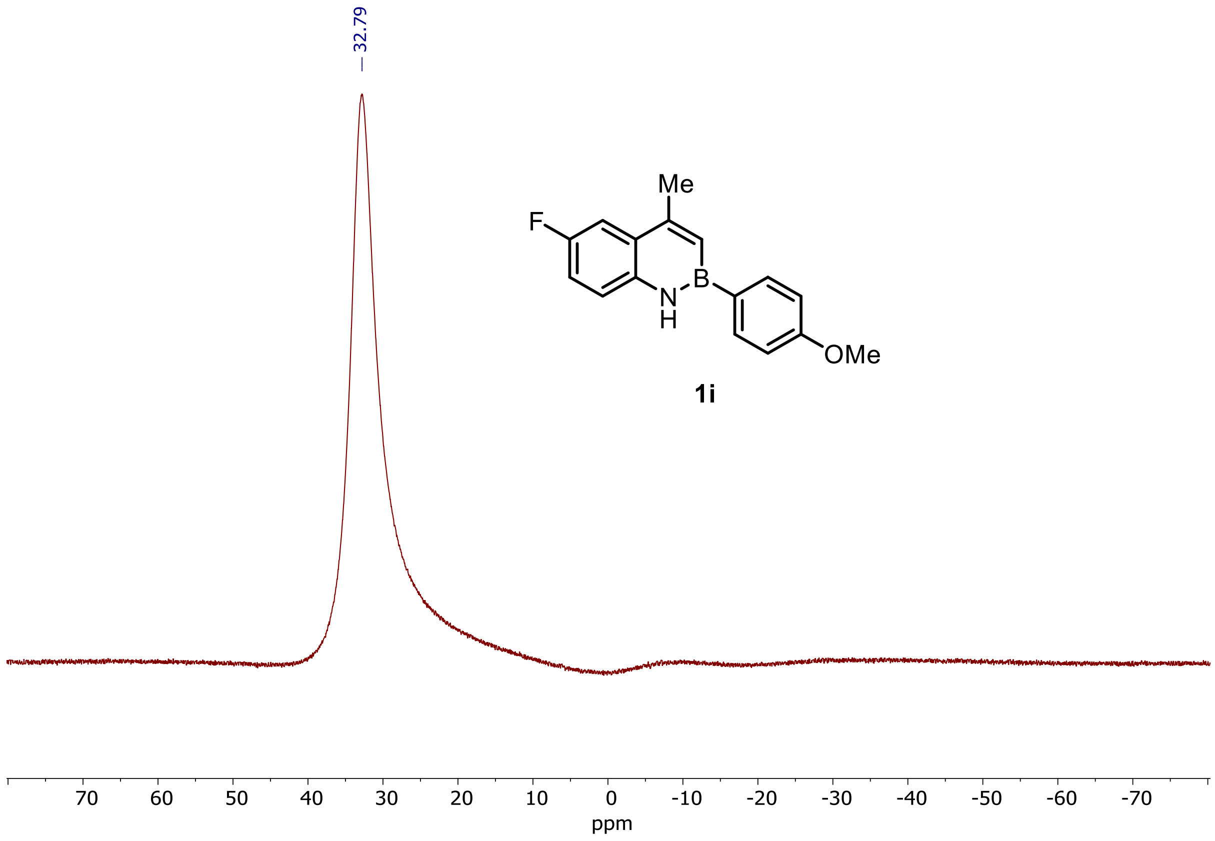
32.79
Me
F
N
H
B
OMe
1i
70 60 50 40 30 20 10 0 -10 -20 -30 -40 -50 -60 -70
ppm

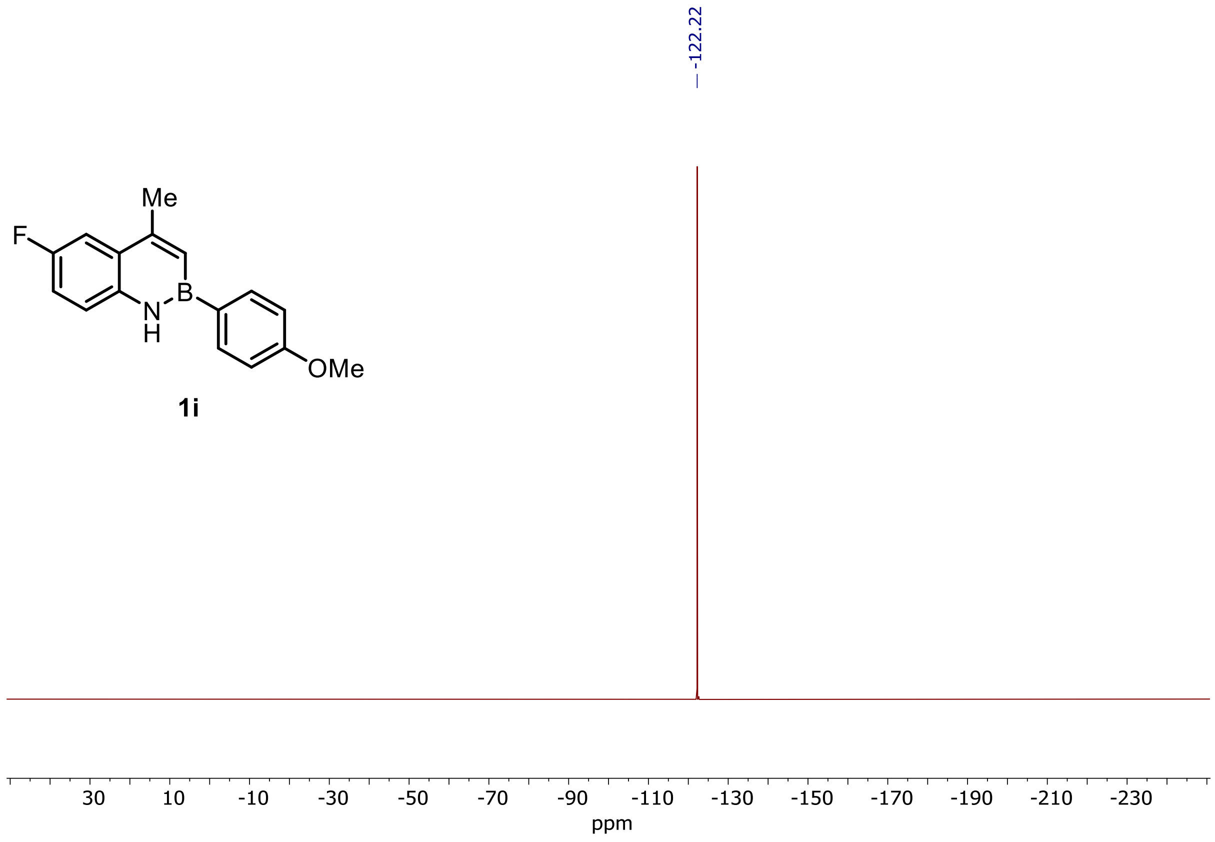
-122.22
Me
F
N
H
B
OMe
1i
30 10 -10 -30 -50 -70 -90 -110 -130 -150 -170 -190 -210 -230
ppm

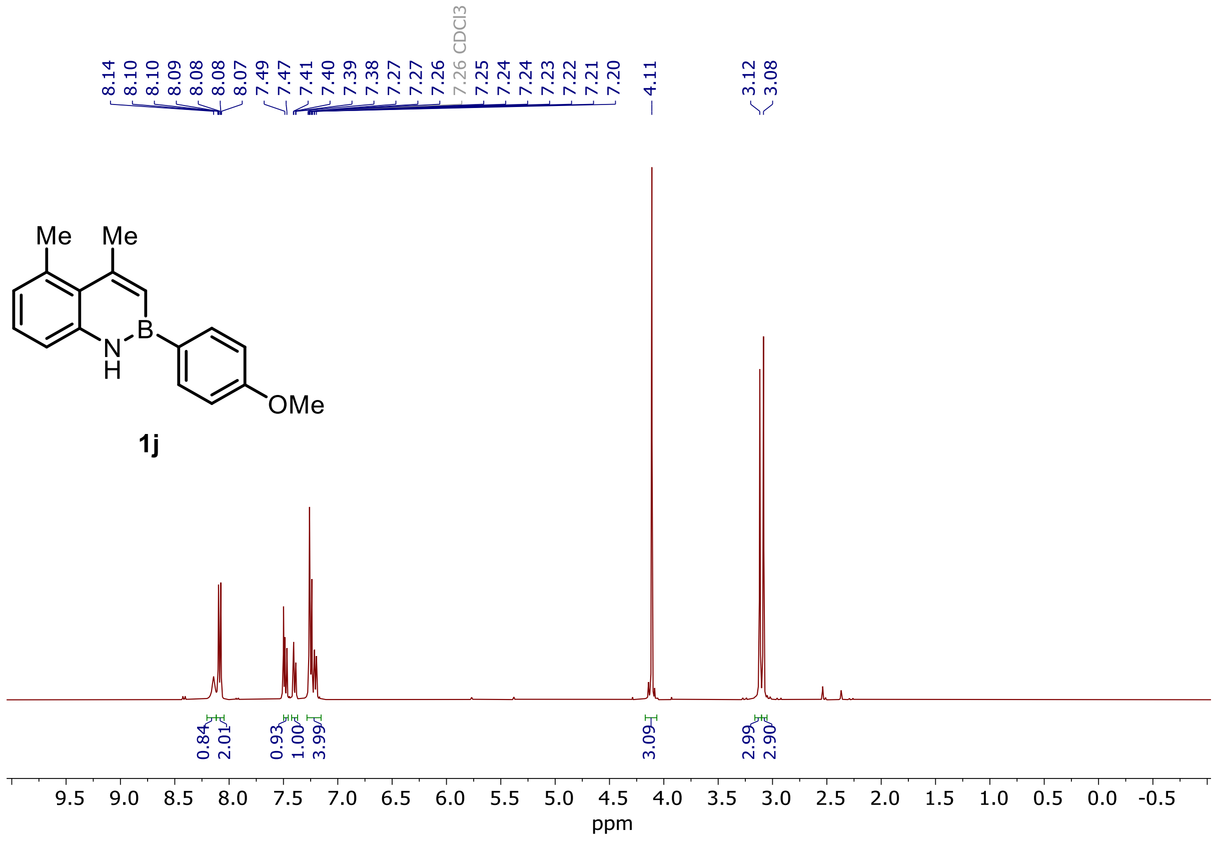
8.14 8.10 8.10 8.09 8.08 8.08 8.07 7.49 7.47 7.41 7.40 7.39 7.38 7.27 7.27 7.26 7.26 CDCl3 7.25 7.24 7.24 7.23 7.22 7.21 7.20
4.11
3.12 3.08
Me Me
N B H
OMe
1j
0.84 2.01 0.93 1.00 3.99 3.09 2.99 2.90
9.5 9.0 8.5 8.0 7.5 7.0 6.5 6.0 5.5 5.0 4.5 4.0 3.5 3.0 2.5 2.0 1.5 1.0 0.5 0.0 -0.5
ppm

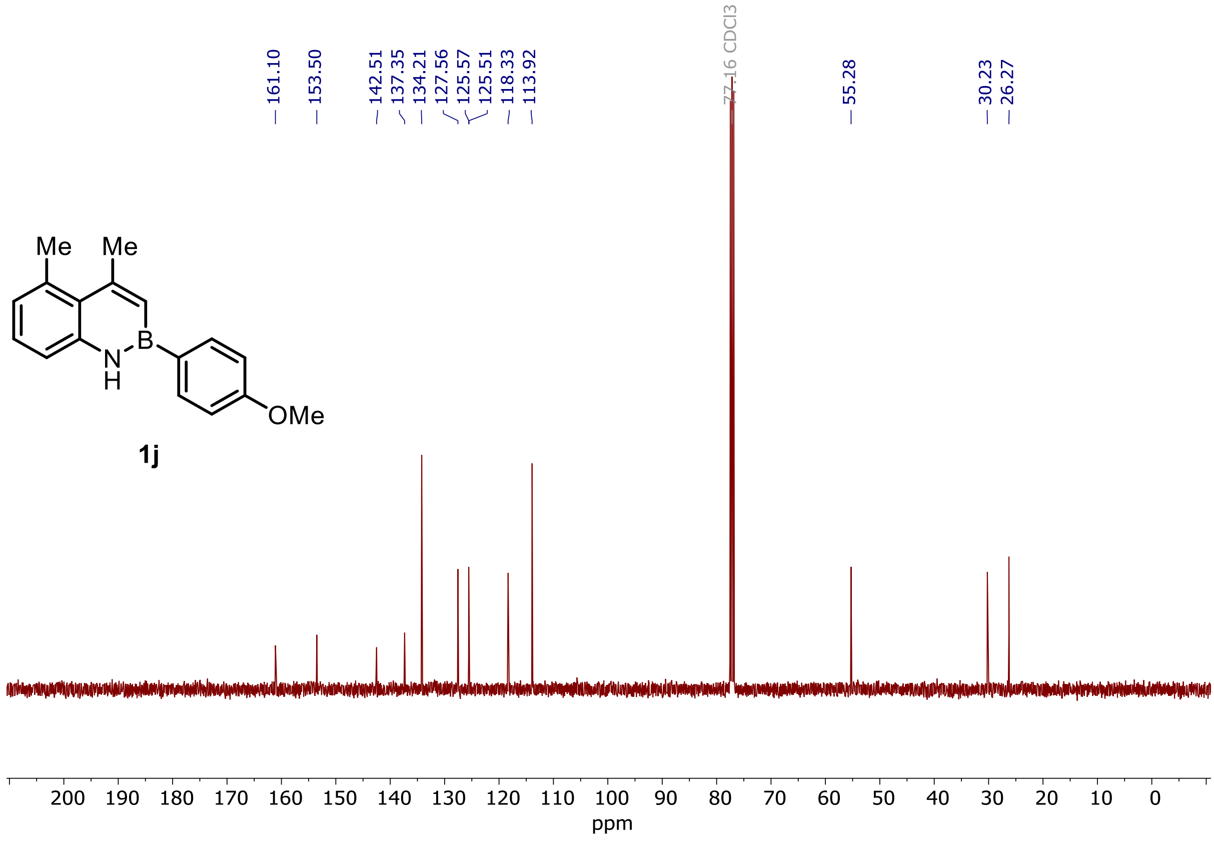
161.10
153.50
142.51 137.35 134.21 127.56 125.57 125.51 118.33 113.92
77.16 CDCl3
55.28
30.23 26.27
Me Me
N B H
OMe
1j
200 190 180 170 160 150 140 130 120 110 100 90 80 70 60 50 40 30 20 10 0
ppm

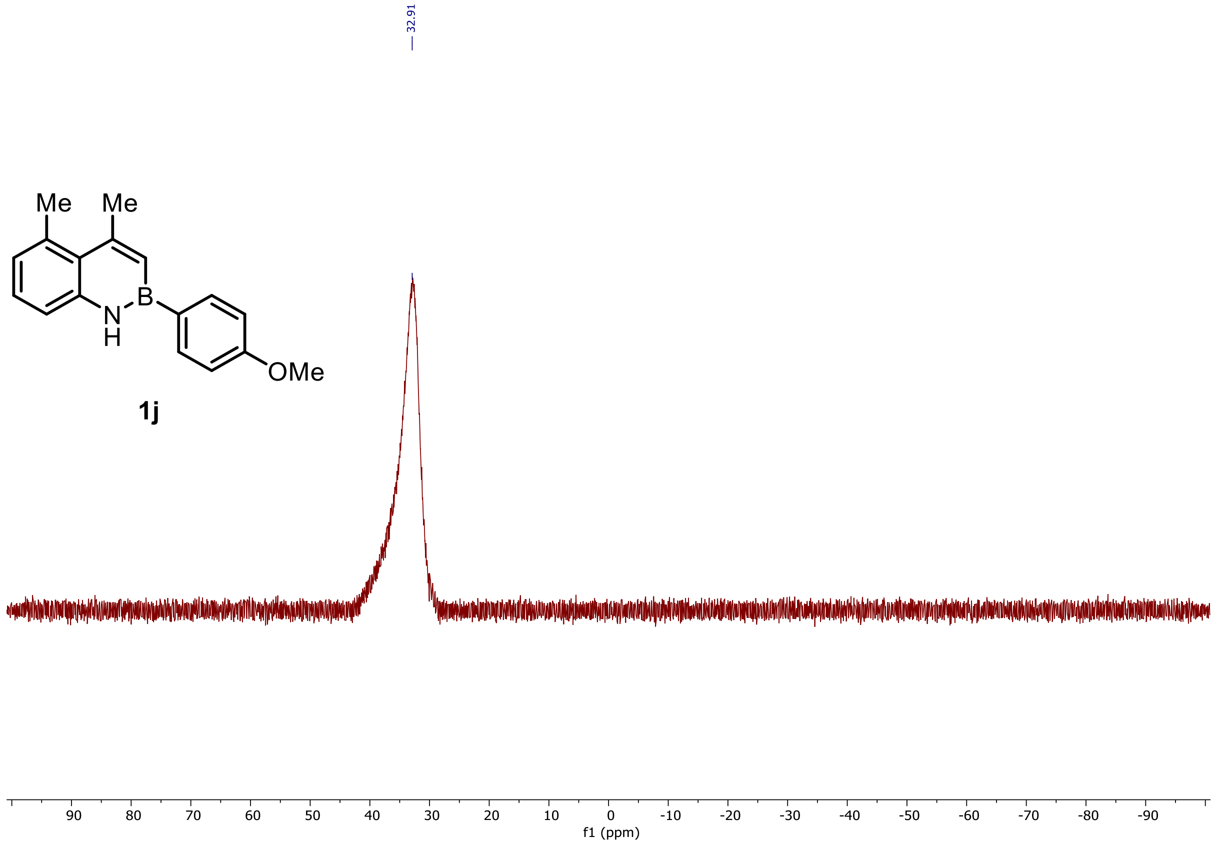

32.91
Me
Me
N
H
B
OMe
90 80 70 60 50 40 30 20 10 0 -10 -20 -30 -40 -50 -60 -70 -80 -90
f1 (ppm)

1j

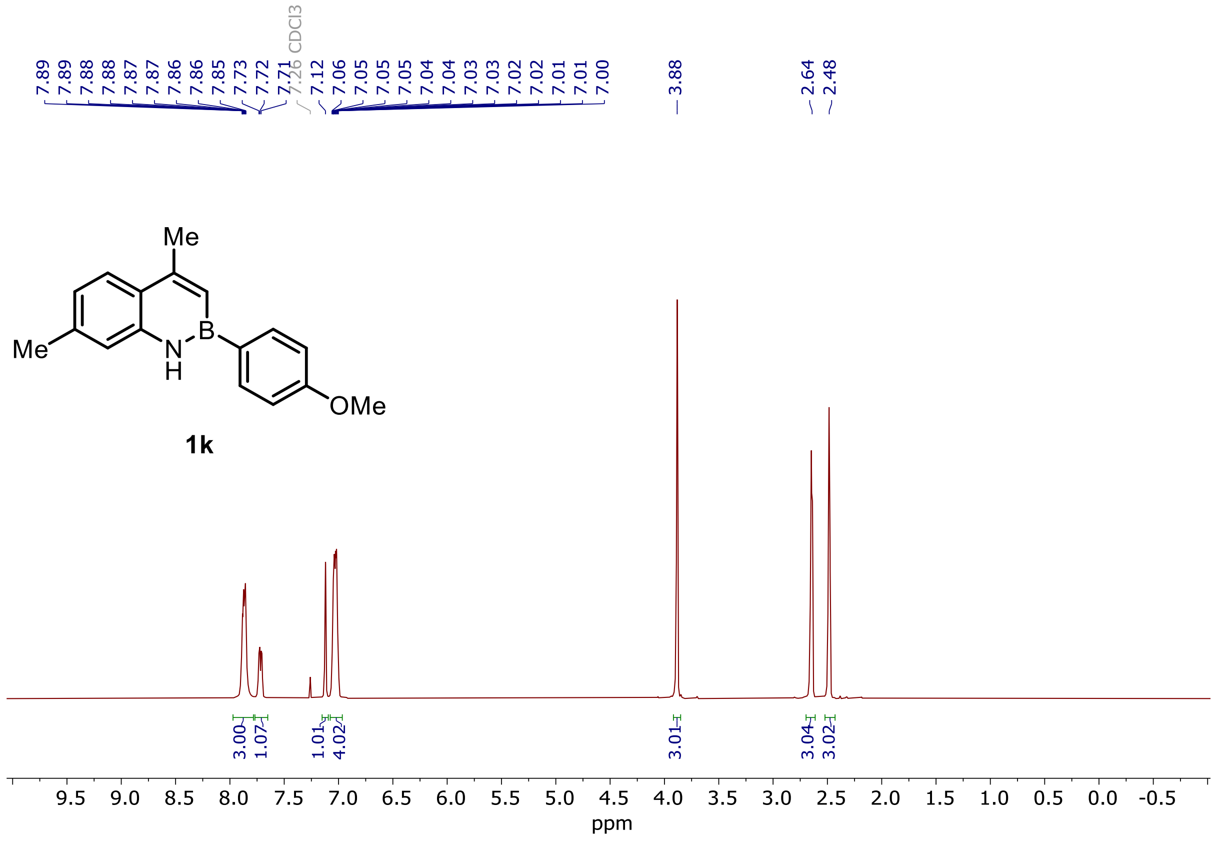

7.89 7.89 7.88 7.88 7.87 7.87 7.86 7.86 7.85 7.73 7.72 7.71 7.26 CDCl3 7.12 7.06 7.05 7.05 7.05 7.04 7.04 7.03 7.03 7.02 7.02 7.01 7.01 7.00
3.88
2.64 2.48
Me
Me
N
H
B
OMe
3.00 1.07 1.01 4.02 3.01 3.04 3.02
9.5 9.0 8.5 8.0 7.5 7.0 6.5 6.0 5.5 5.0 4.5 4.0 3.5 3.0 2.5 2.0 1.5 1.0 0.5 0.0 -0.5
ppm

1k

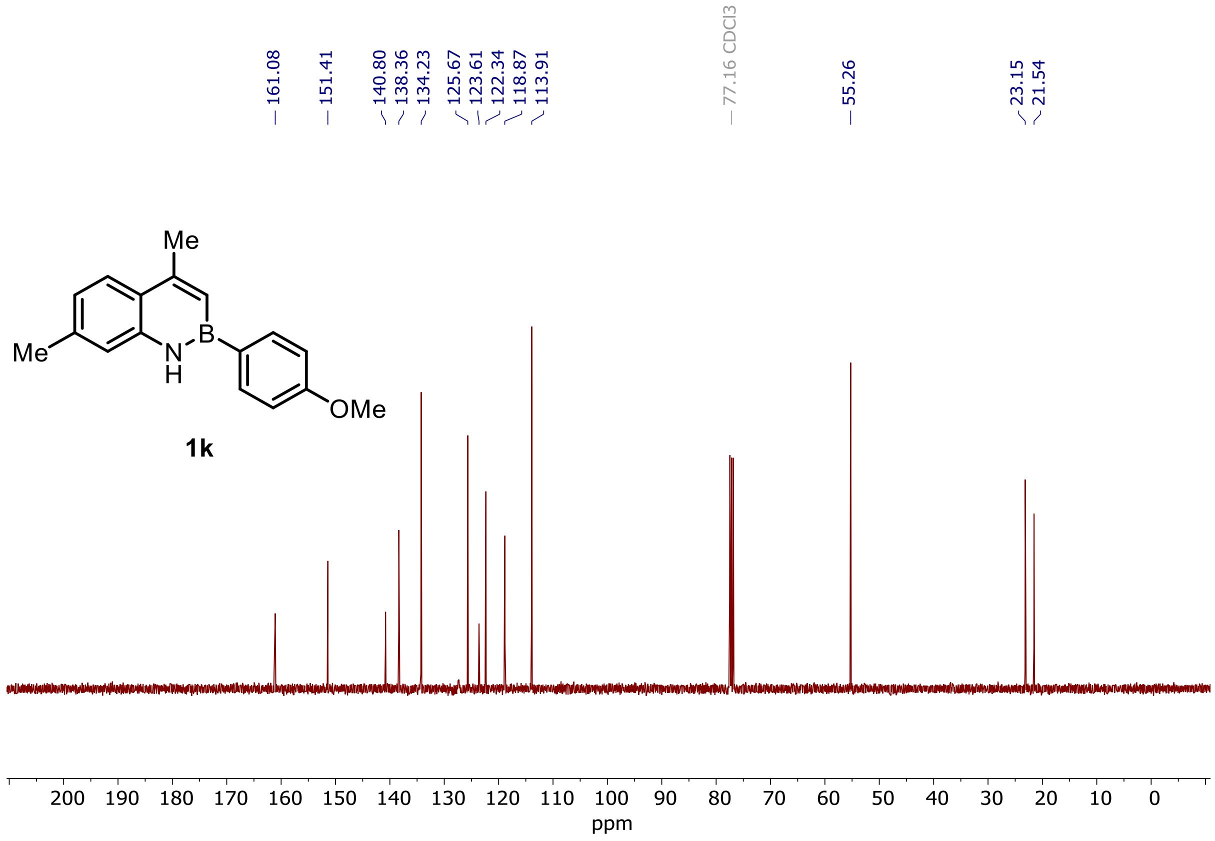
161.08
151.41
140.80
138.36
134.23
125.67
123.61
122.34
118.87
113.91
77.16 CDCl3
55.26
23.15
21.54
Me
Me
N
H
B
OMe
1k
200 190 180 170 160 150 140 130 120 110 100 90 80 70 60 50 40 30 20 10 0
ppm

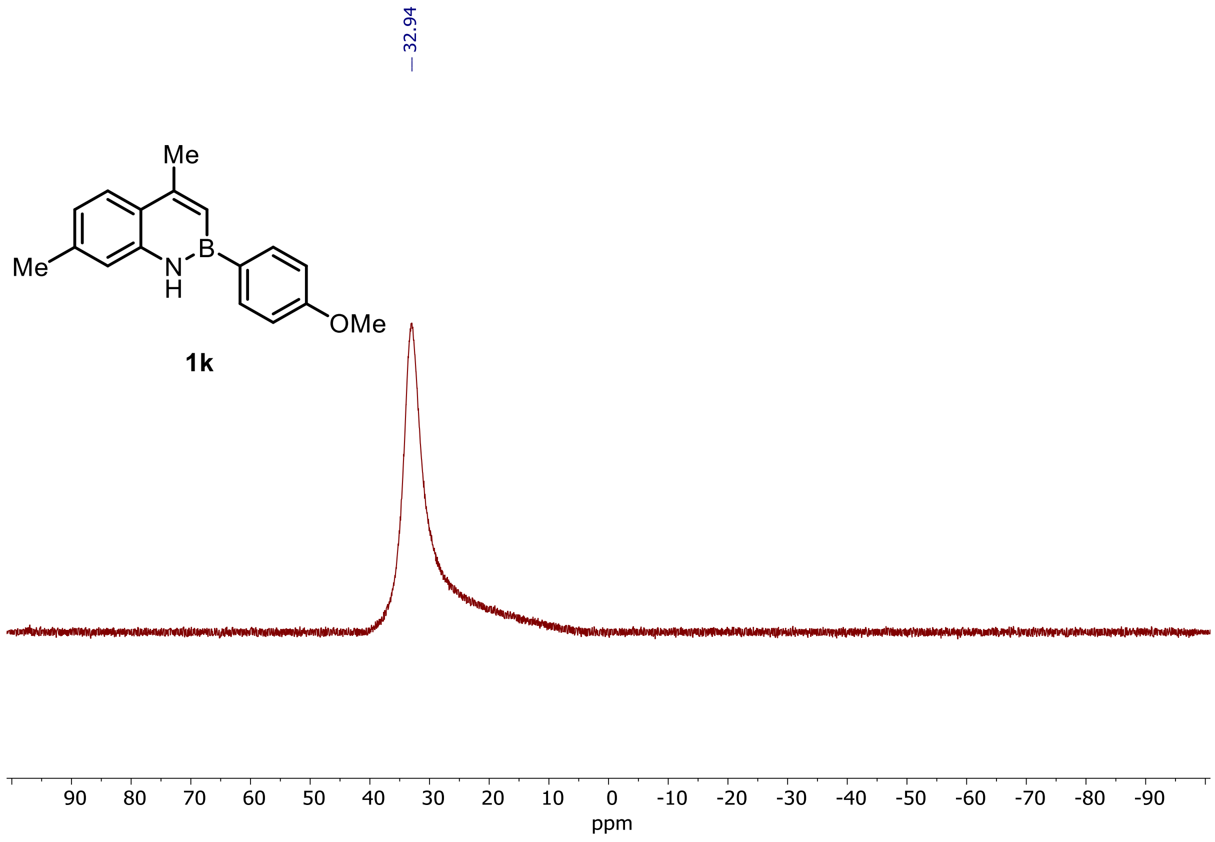
32.94
Me
Me
N
H
B
OMe
1k
90 80 70 60 50 40 30 20 10 0 -10 -20 -30 -40 -50 -60 -70 -80 -90
ppm

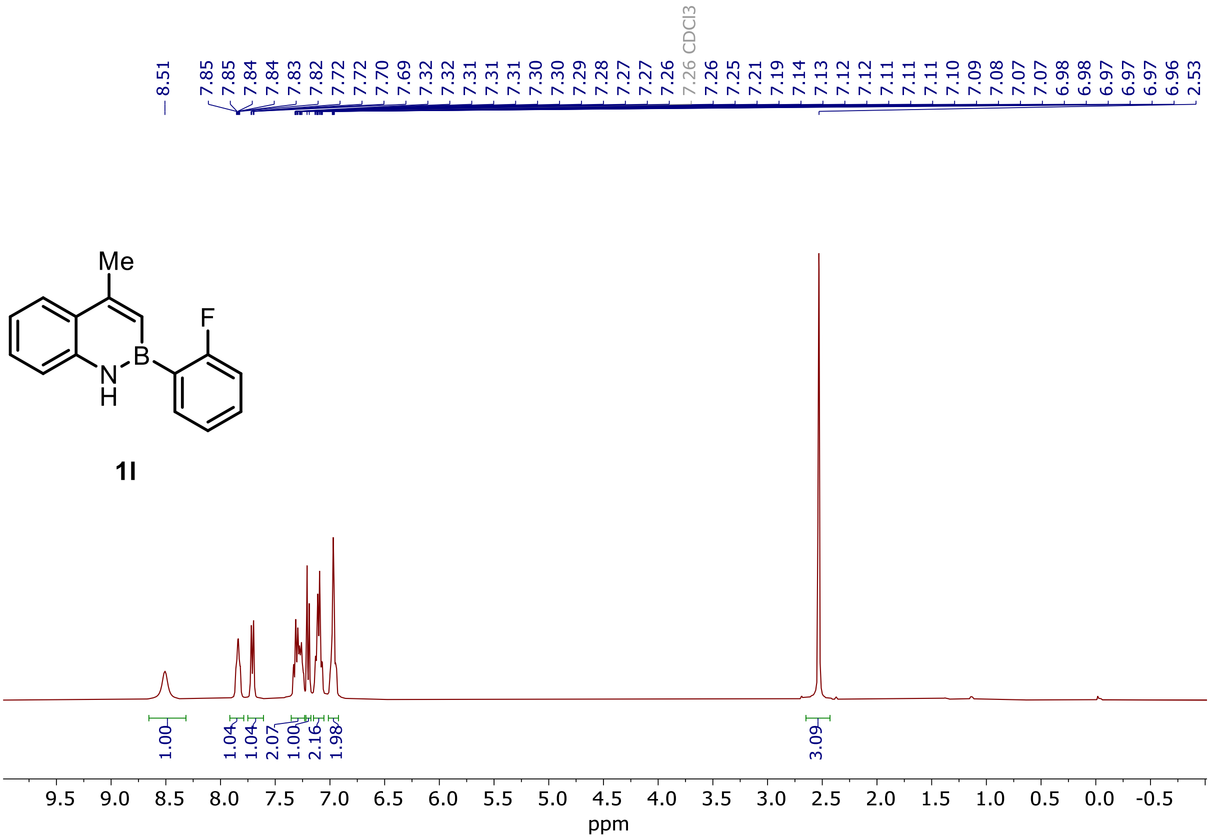
8.51
7.85 7.85 7.84 7.84 7.83 7.82 7.72 7.72 7.70 7.69 7.32 7.32 7.31 7.31 7.30 7.30 7.29 7.28 7.27 7.27 7.26 7.26 CDCl3 7.26 7.25 7.21 7.19 7.14 7.13 7.12 7.12 7.11 7.11 7.11 7.10 7.09 7.08 7.07 7.07 6.98 6.98 6.97 6.97 6.97 6.96 2.53
Me
F
B
N
H
1l
1.00 1.04 1.04 2.07 1.00 2.16 1.98 3.09
9.5 9.0 8.5 8.0 7.5 7.0 6.5 6.0 5.5 5.0 4.5 4.0 3.5 3.0 2.5 2.0 1.5 1.0 0.5 0.0 -0.5
ppm

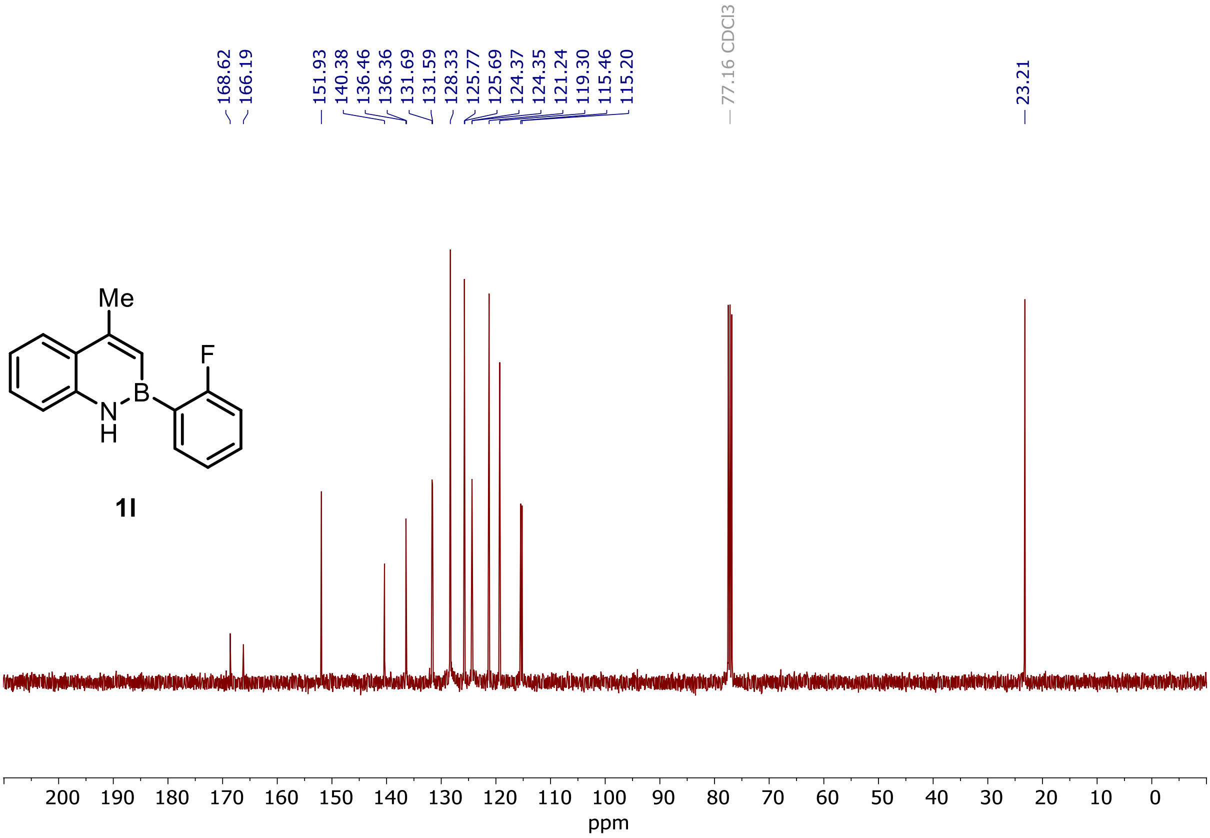
168.62 166.19 151.93 140.38 136.46 136.36 131.69 131.59 128.33 125.77 125.69 124.37 124.35 121.24 119.30 115.46 115.20
77.16 CDCl3
23.21
Me
F
B
N
H
1l
200 190 180 170 160 150 140 130 120 110 100 90 80 70 60 50 40 30 20 10 0
ppm

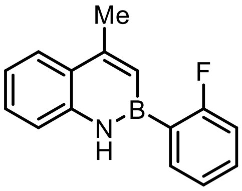
Me
F
B
N
H

1l

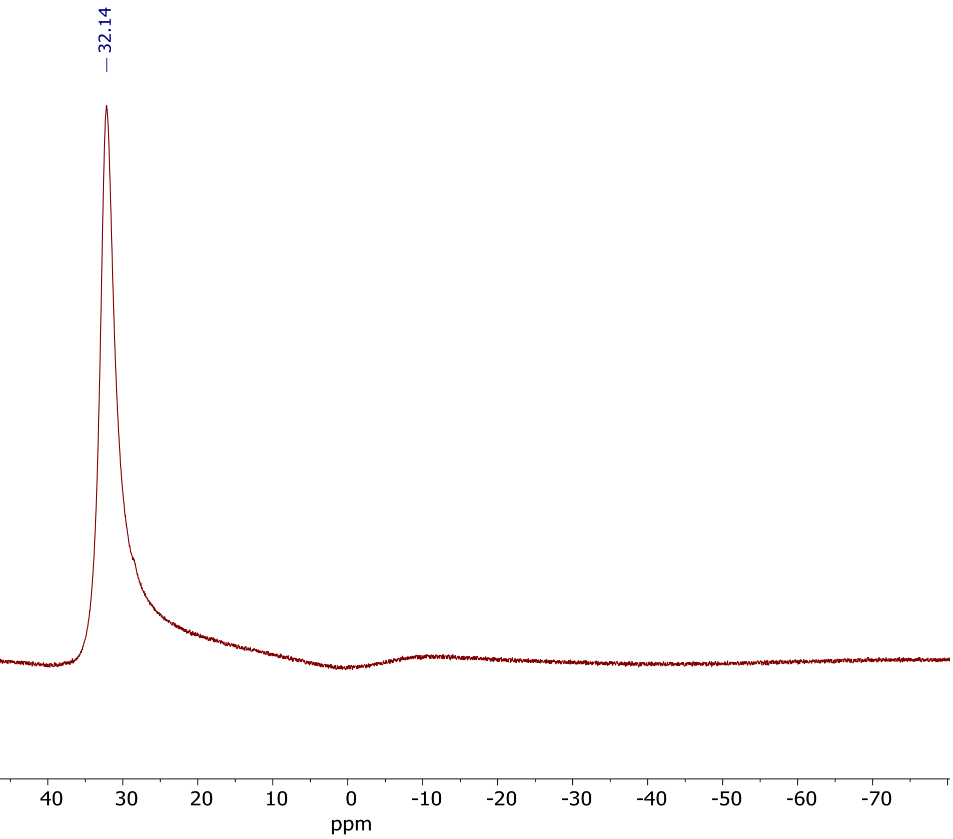
32.14
70 60 50 40 30 20 10 0 -10 -20 -30 -40 -50 -60 -70
ppm

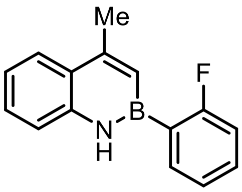
Me
F
B
N
H

1l

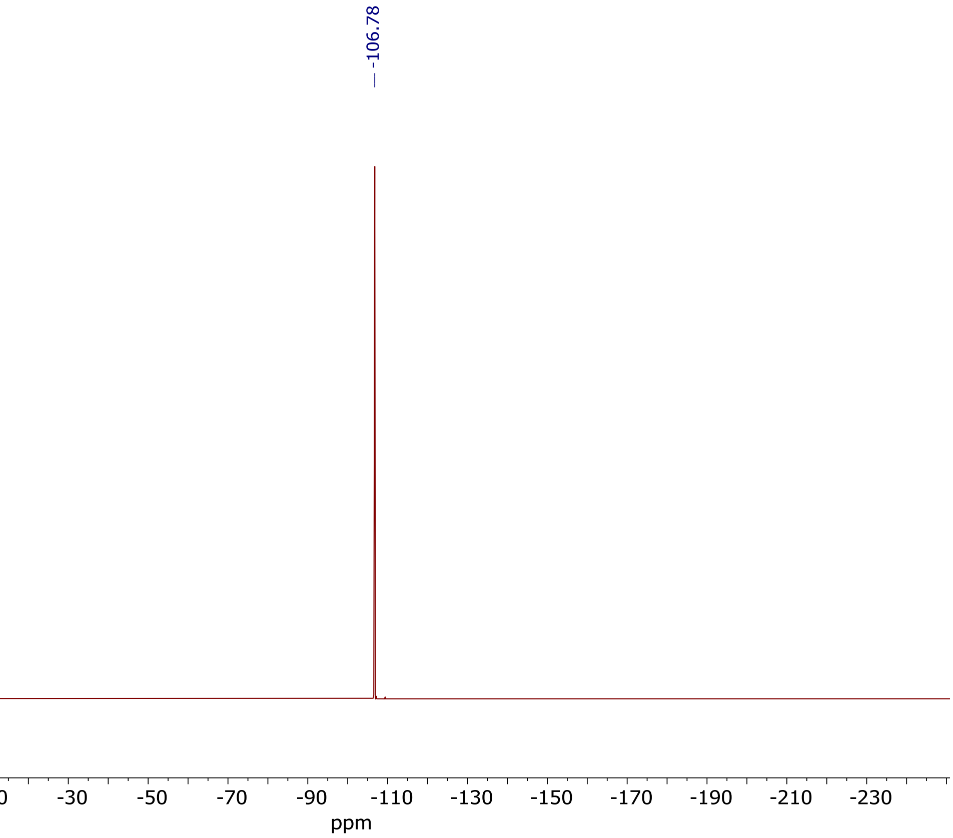
-106.78
30 10 -10 -30 -50 -70 -90 -110 -130 -150 -170 -190 -210 -230
ppm

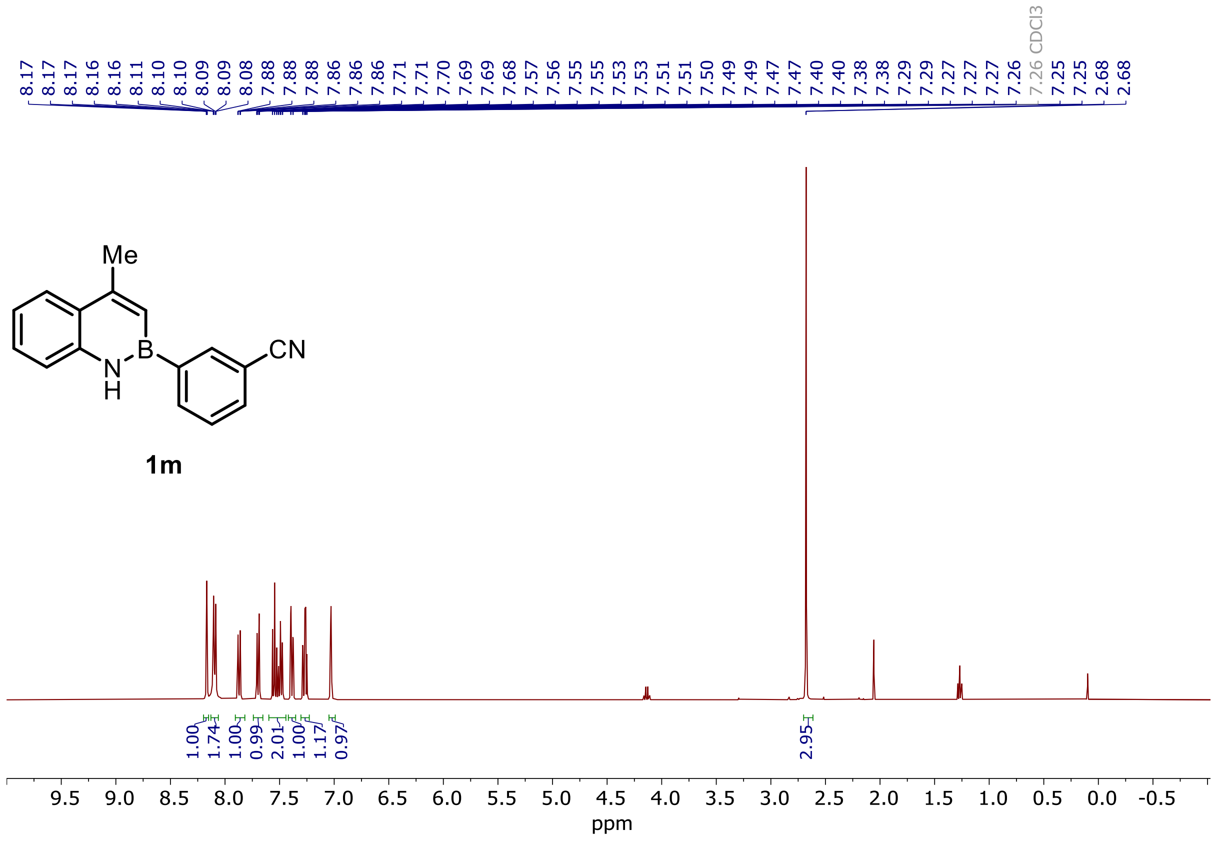

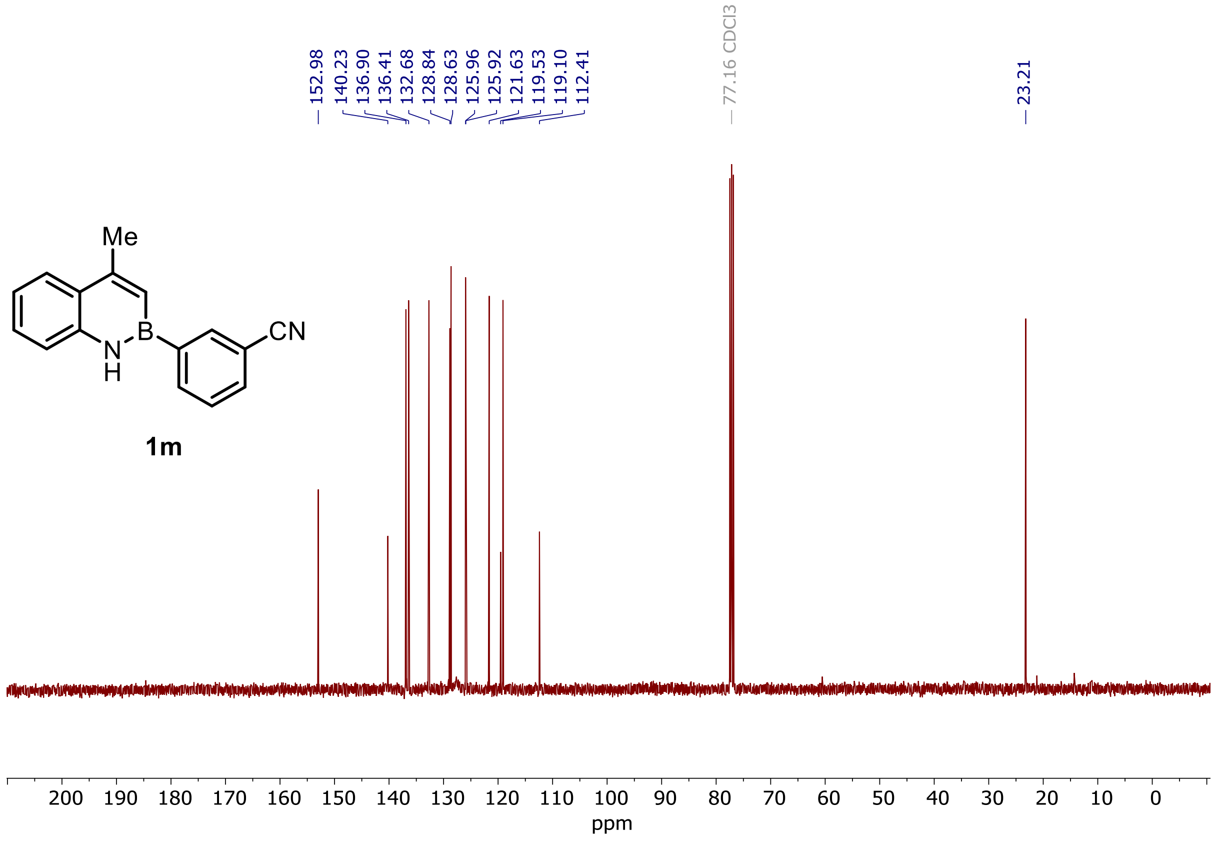

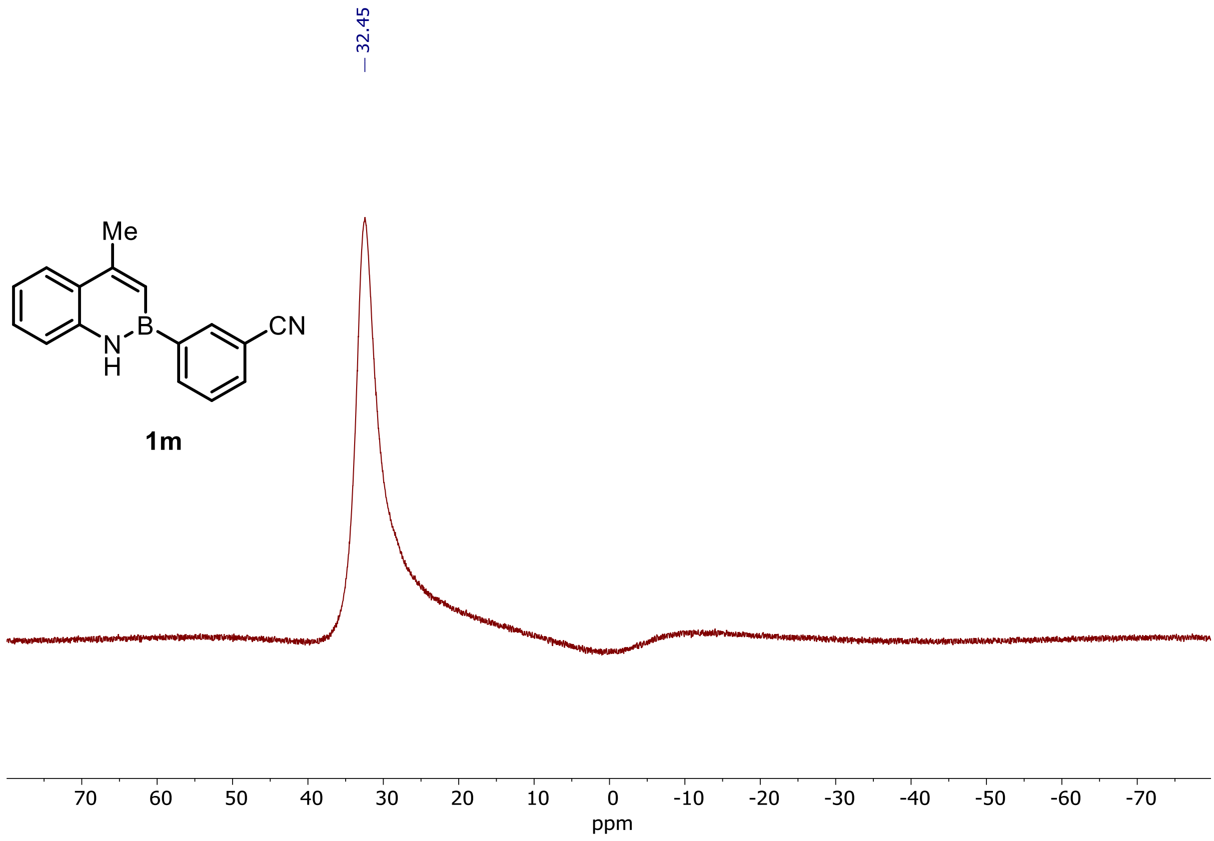
32.45
Me
B
N
H
CN
1m
70 60 50 40 30 20 10 0 -10 -20 -30 -40 -50 -60 -70
ppm

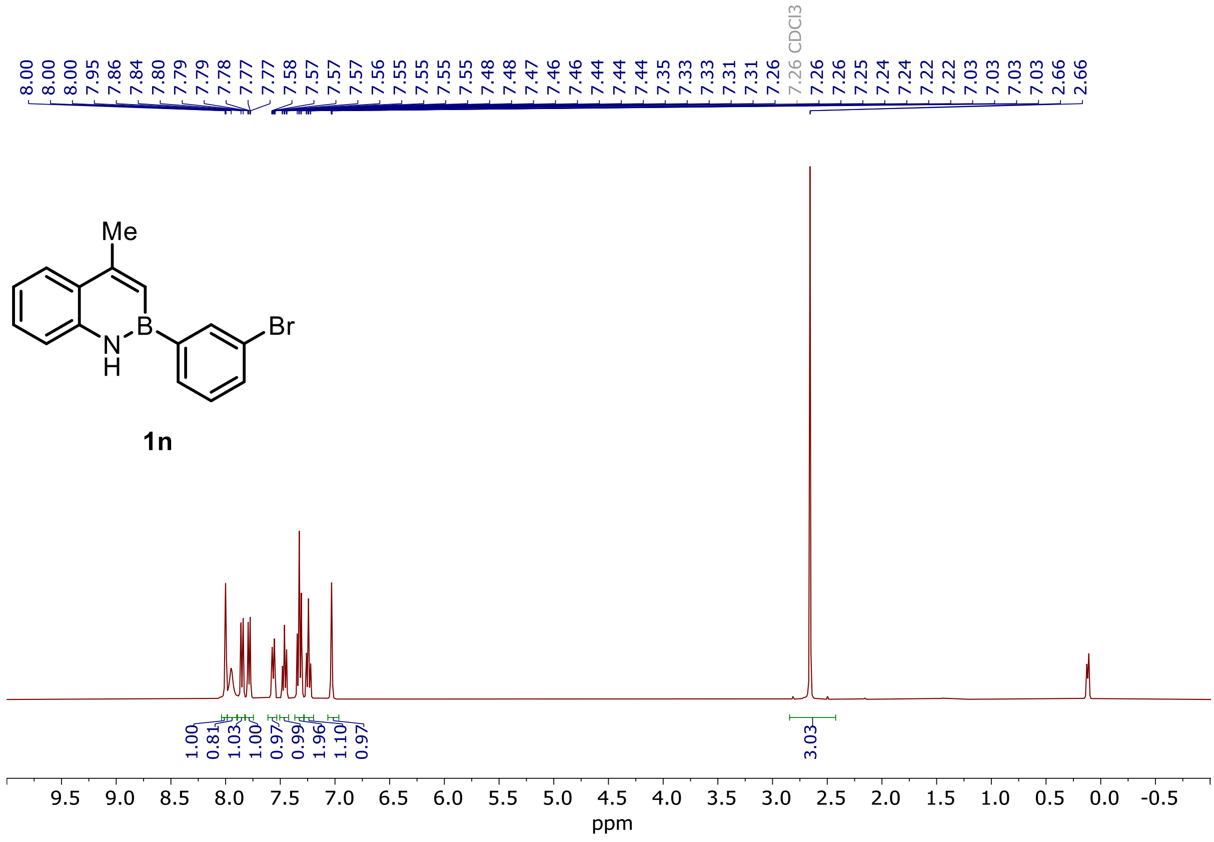
8.00 8.00 8.00 7.95 7.86 7.84 7.80 7.79 7.79 7.78 7.77 7.77 7.58 7.57 7.57 7.56 7.55 7.55 7.55 7.48 7.48 7.47 7.46 7.44 7.44 7.44 7.35 7.33 7.31 7.31 7.26 7.26 CDCl3 7.26 7.25 7.24 7.24 7.22 7.22 7.03 7.03 7.03 7.03 2.66 2.66
Me
B
N
H
Br
1n
1.00 0.81 1.03 1.00 0.97 0.99 1.96 1.10 0.97
3.03
9.5 9.0 8.5 8.0 7.5 7.0 6.5 6.0 5.5 5.0 4.5 4.0 3.5 3.0 2.5 2.0 1.5 1.0 0.5 0.0 -0.5
ppm

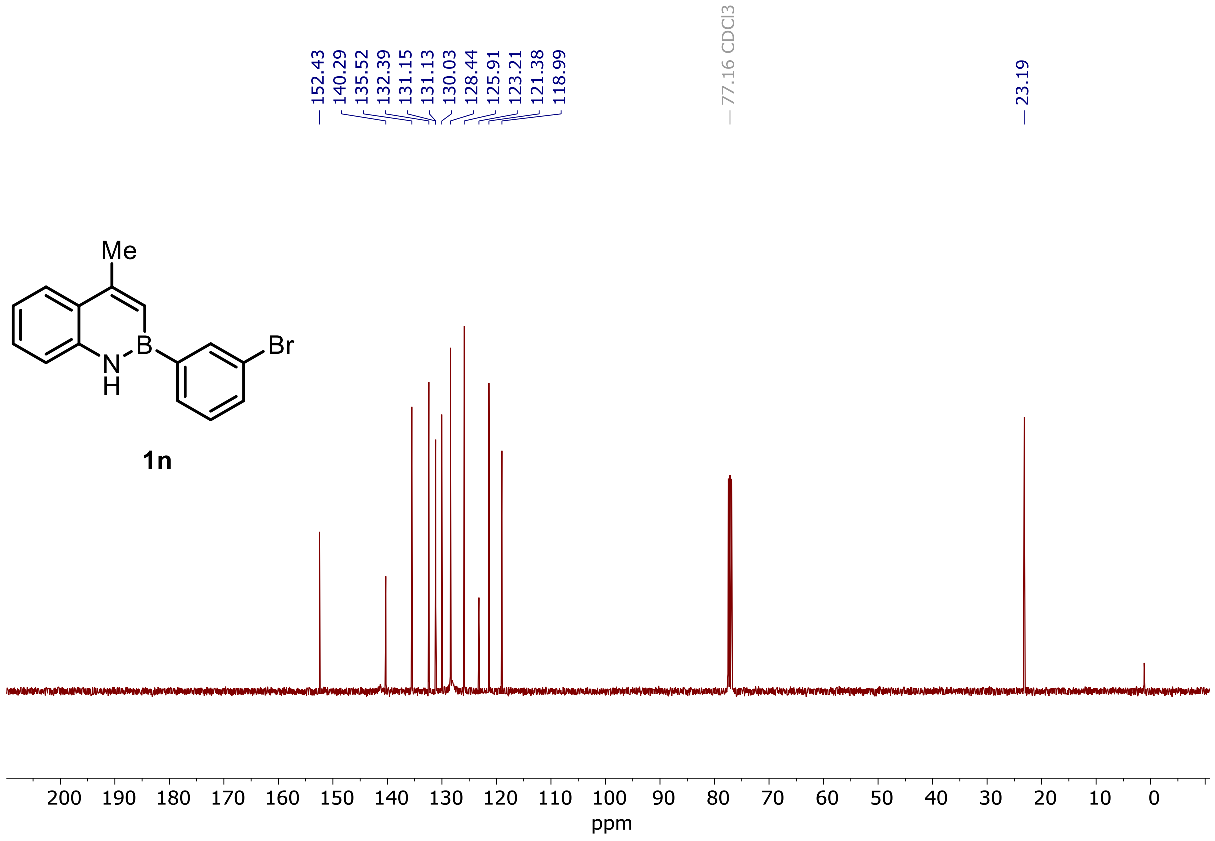
152.43
140.29
135.52
132.39
131.15
131.13
130.03
128.44
125.91
123.21
121.38
118.99
77.16 CDCl3
23.19
Me
Br
N
B
H
1n
200 190 180 170 160 150 140 130 120 110 100 90 80 70 60 50 40 30 20 10 0
ppm

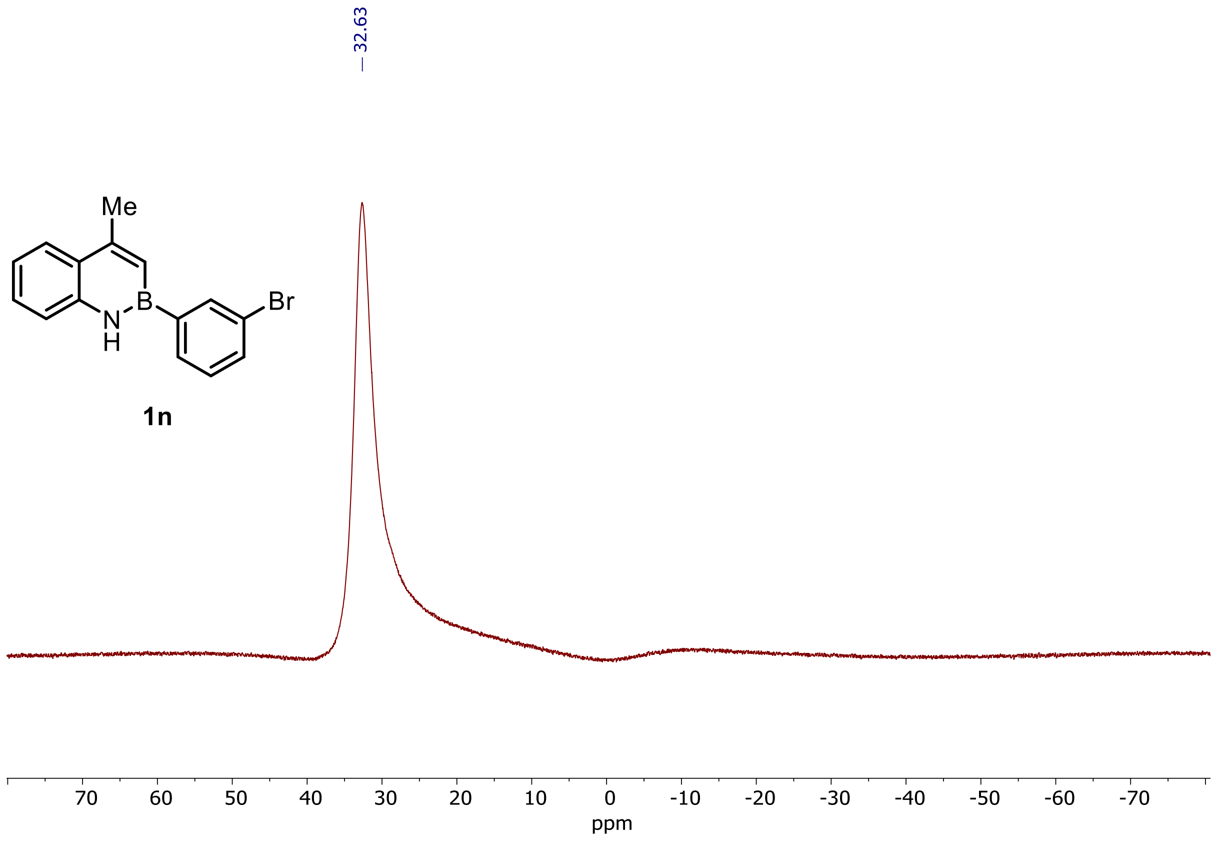
32.63
Me
Br
N
B
H
1n
70 60 50 40 30 20 10 0 -10 -20 -30 -40 -50 -60 -70
ppm

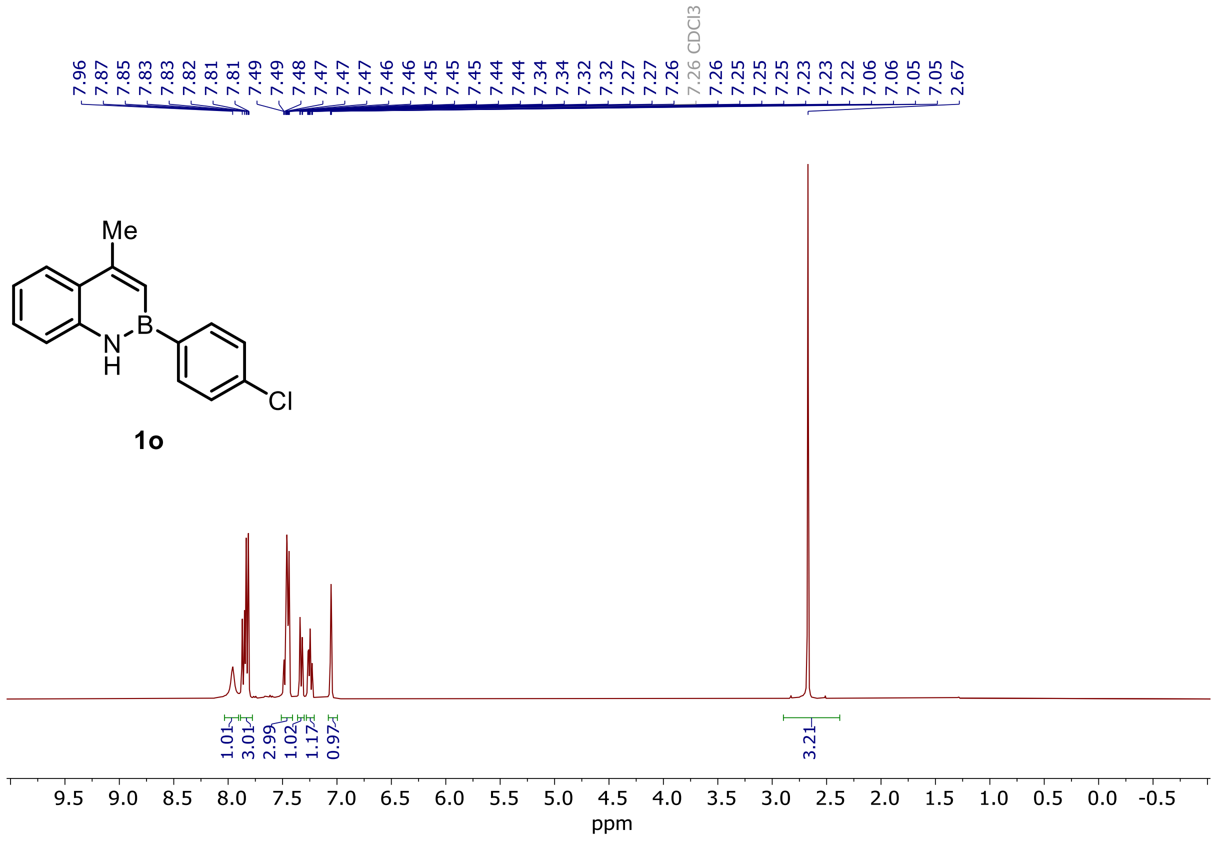
7.96
7.87
7.85
7.83
7.83
7.82
7.81
7.81
7.49
7.49
7.48
7.47
7.47
7.47
7.46
7.46
7.45
7.45
7.45
7.44
7.44
7.34
7.34
7.32
7.32
7.27
7.27
7.26
7.26 CDCl3
7.26
7.25
7.25
7.25
7.23
7.23
7.22
7.06
7.06
7.05
7.05
2.67
Me
N
H
B
Cl
1o
1.01
3.01
2.99
1.02
1.17
0.97
3.21
9.5 9.0 8.5 8.0 7.5 7.0 6.5 6.0 5.5 5.0 4.5 4.0 3.5 3.0 2.5 2.0 1.5 1.0 0.5 0.0 -0.5
ppm

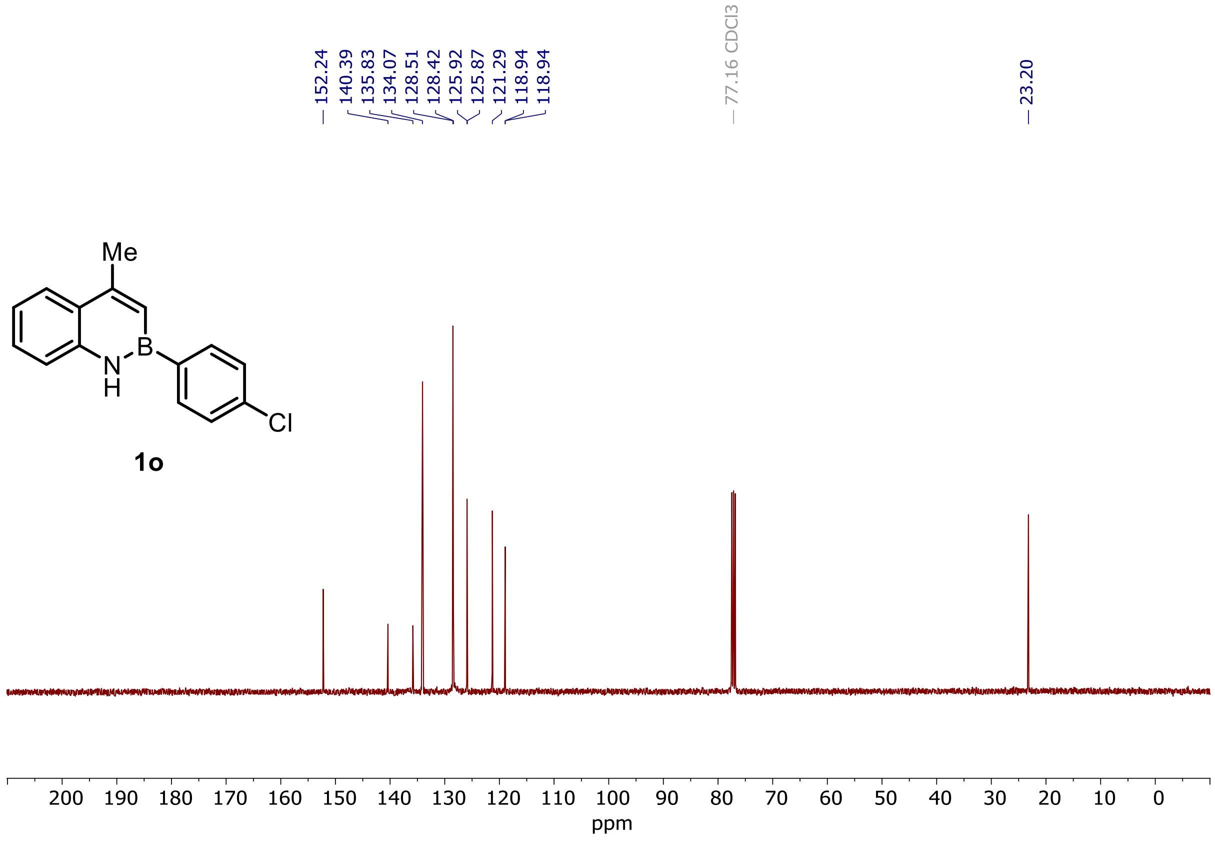
152.24
140.39
135.83
134.07
128.51
128.42
125.92
125.87
121.29
118.94
118.94
77.16 CDCl3
23.20
Me
N
H
B
Cl
1o
200 190 180 170 160 150 140 130 120 110 100 90 80 70 60 50 40 30 20 10 0
ppm

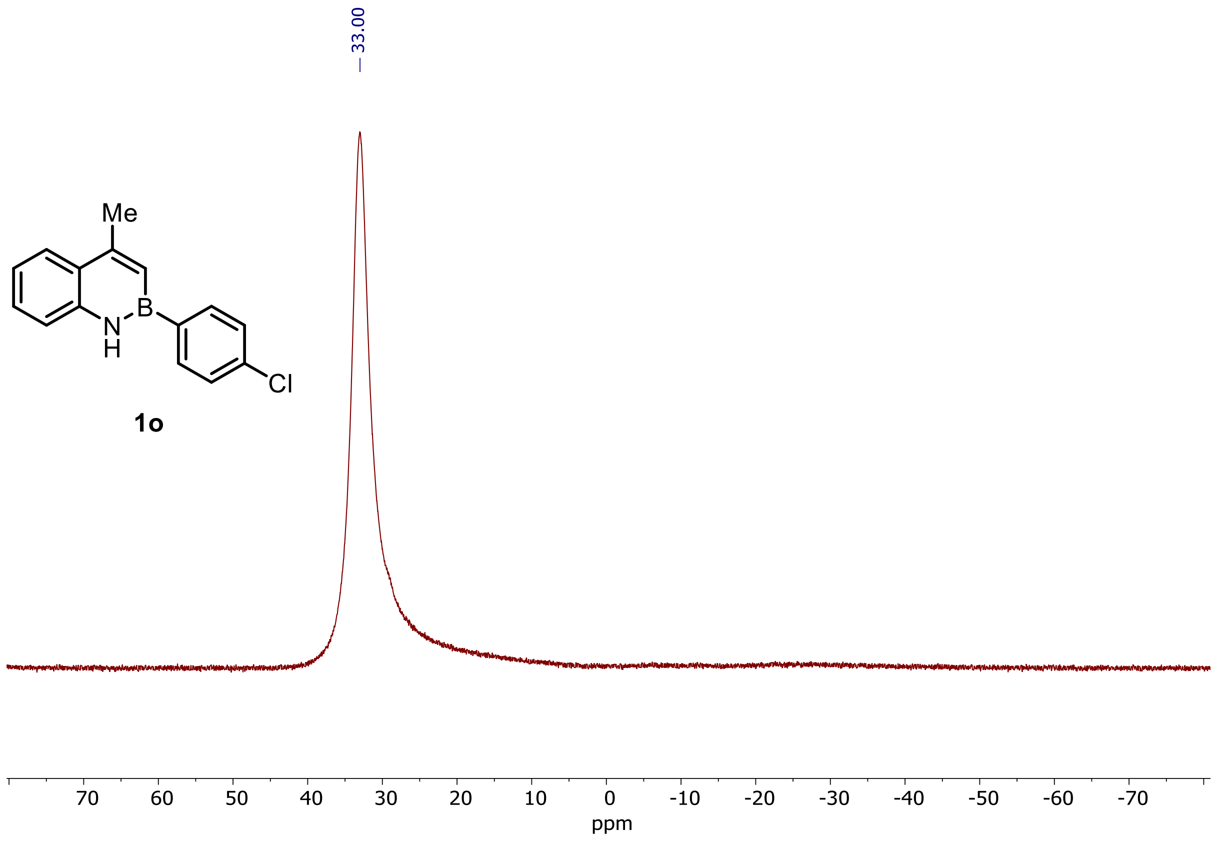

33.00
Me
B
N
H
Cl
1o
70 60 50 40 30 20 10 0 -10 -20 -30 -40 -50 -60 -70
ppm

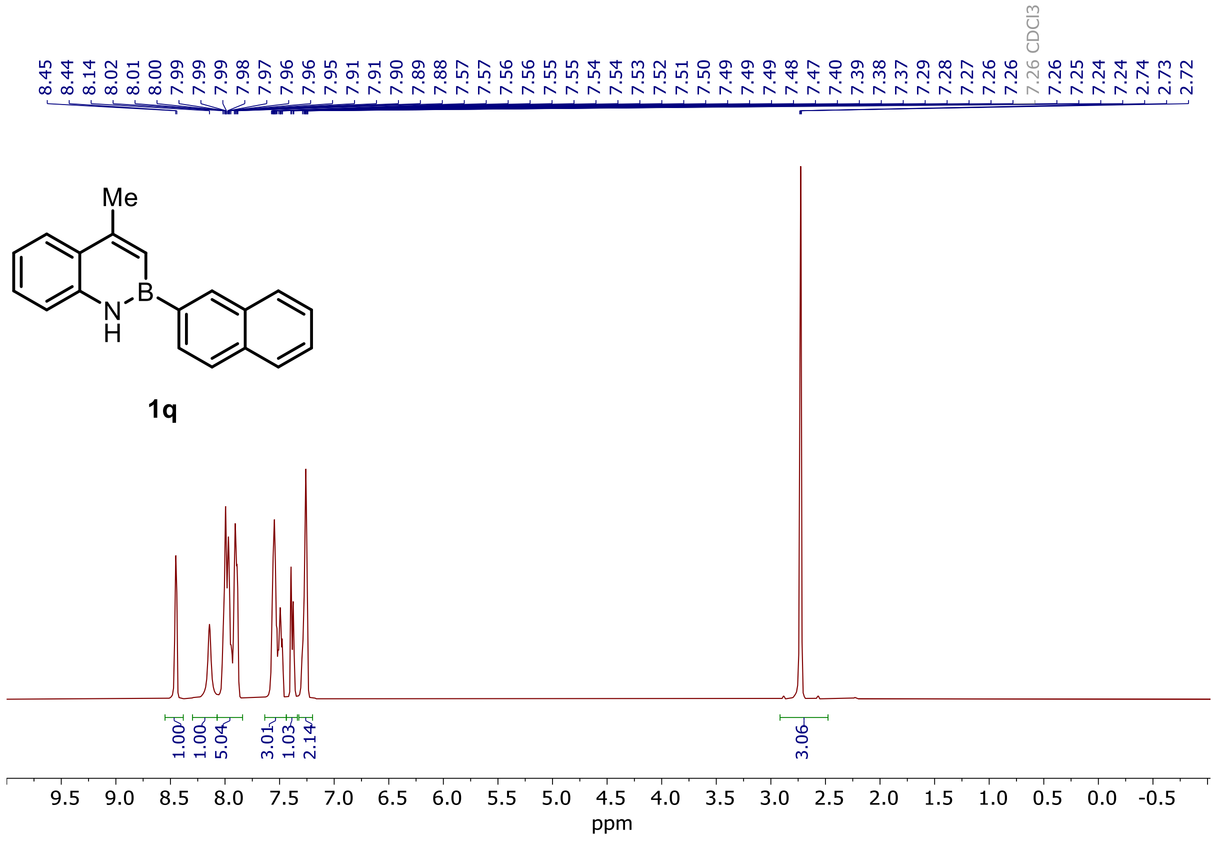

8.45 8.44 8.14 8.02 8.01 8.00 7.99 7.99 7.99 7.98 7.97 7.96 7.96 7.95 7.91 7.91 7.90 7.89 7.88 7.57 7.57 7.56 7.56 7.55 7.54 7.54 7.53 7.52 7.51 7.50 7.49 7.49 7.49 7.48 7.47 7.40 7.39 7.38 7.37 7.29 7.28 7.27 7.26 7.26 7.26 CDCl3 7.26 7.25 7.24 7.24 2.74 2.73 2.72
Me
B
N
H
1q
1.00
1.00
5.04
3.01
1.03
2.14
3.06
9.5 9.0 8.5 8.0 7.5 7.0 6.5 6.0 5.5 5.0 4.5 4.0 3.5 3.0 2.5 2.0 1.5 1.0 0.5 0.0 -0.5
ppm

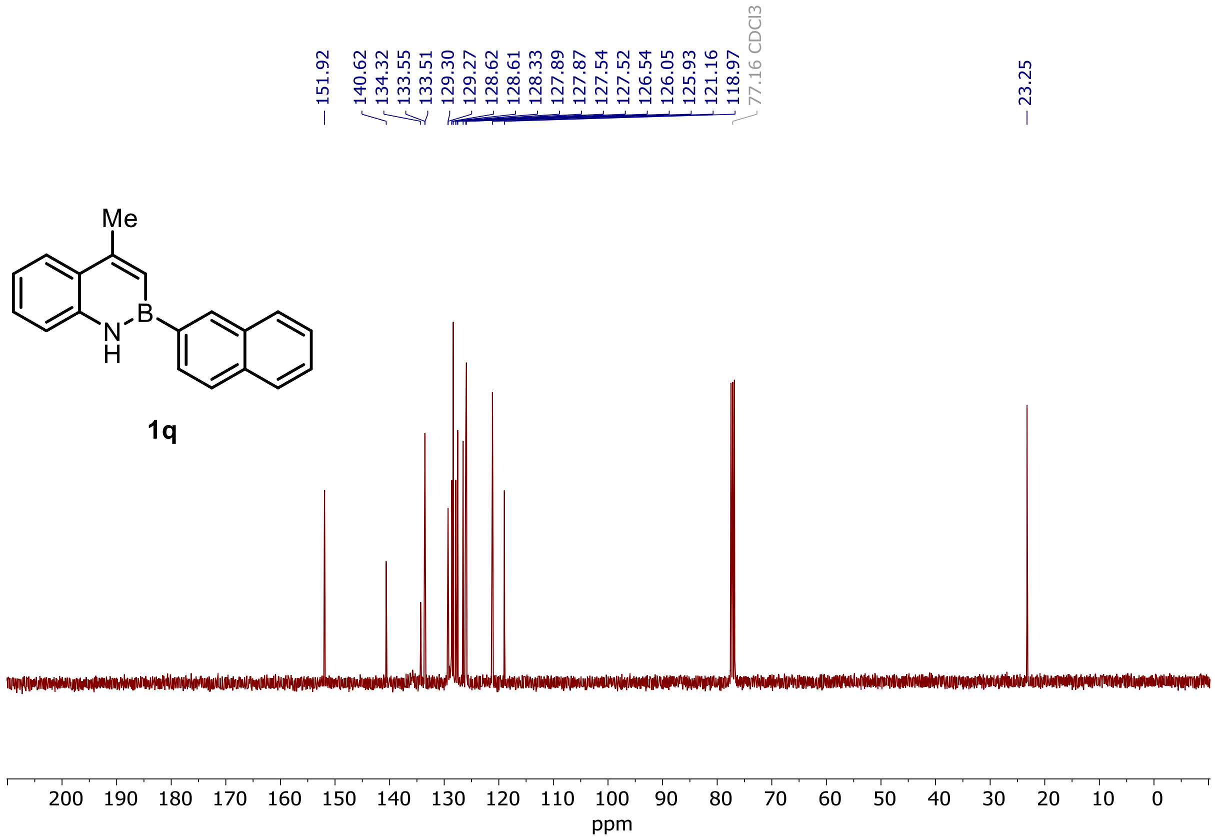
151.92
140.62
134.32
133.55
133.51
129.30
129.27
128.62
128.61
128.33
127.89
127.87
127.54
127.52
126.54
126.05
125.93
121.16
118.97
77.16 CDCl3
23.25
Me
B
N
H
200 190 180 170 160 150 140 130 120 110 100 90 80 70 60 50 40 30 20 10 0
ppm

1q

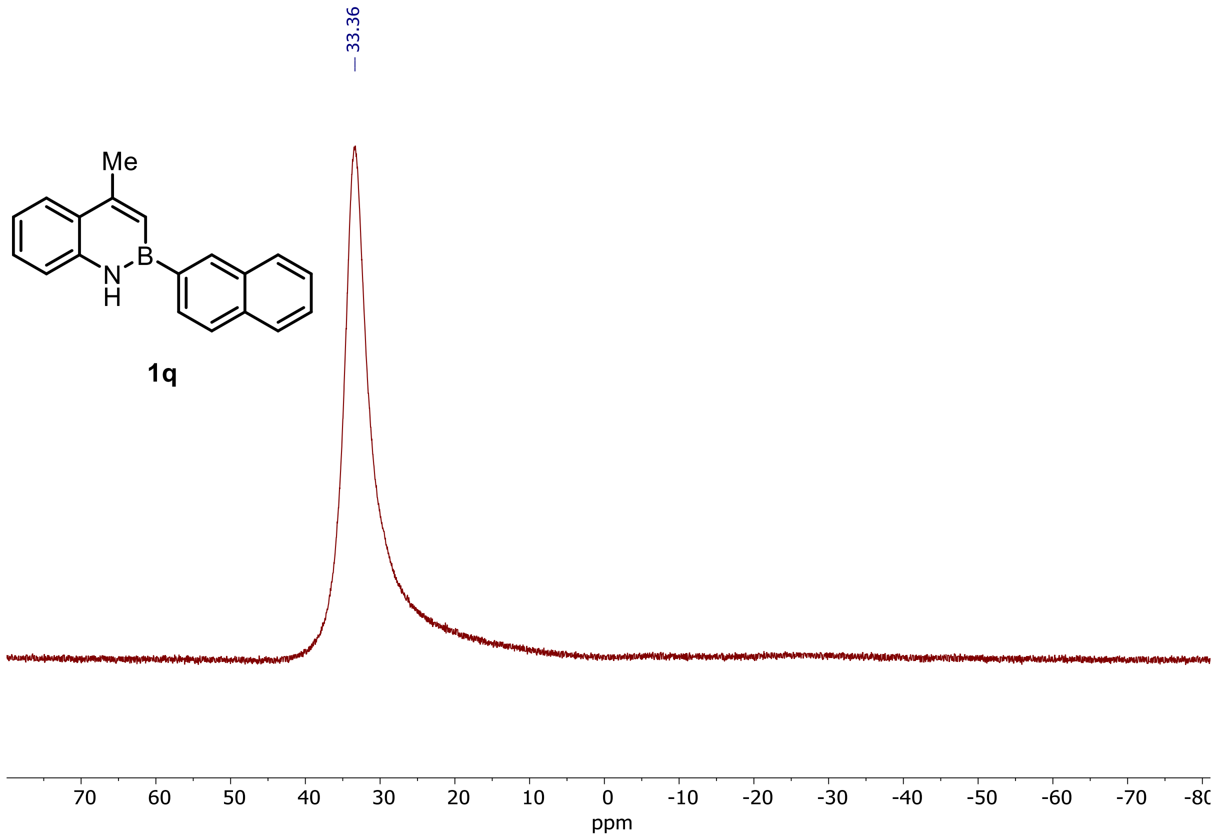
33.36
Me
B
N
H
70 60 50 40 30 20 10 0 -10 -20 -30 -40 -50 -60 -70 -80
ppm

1q

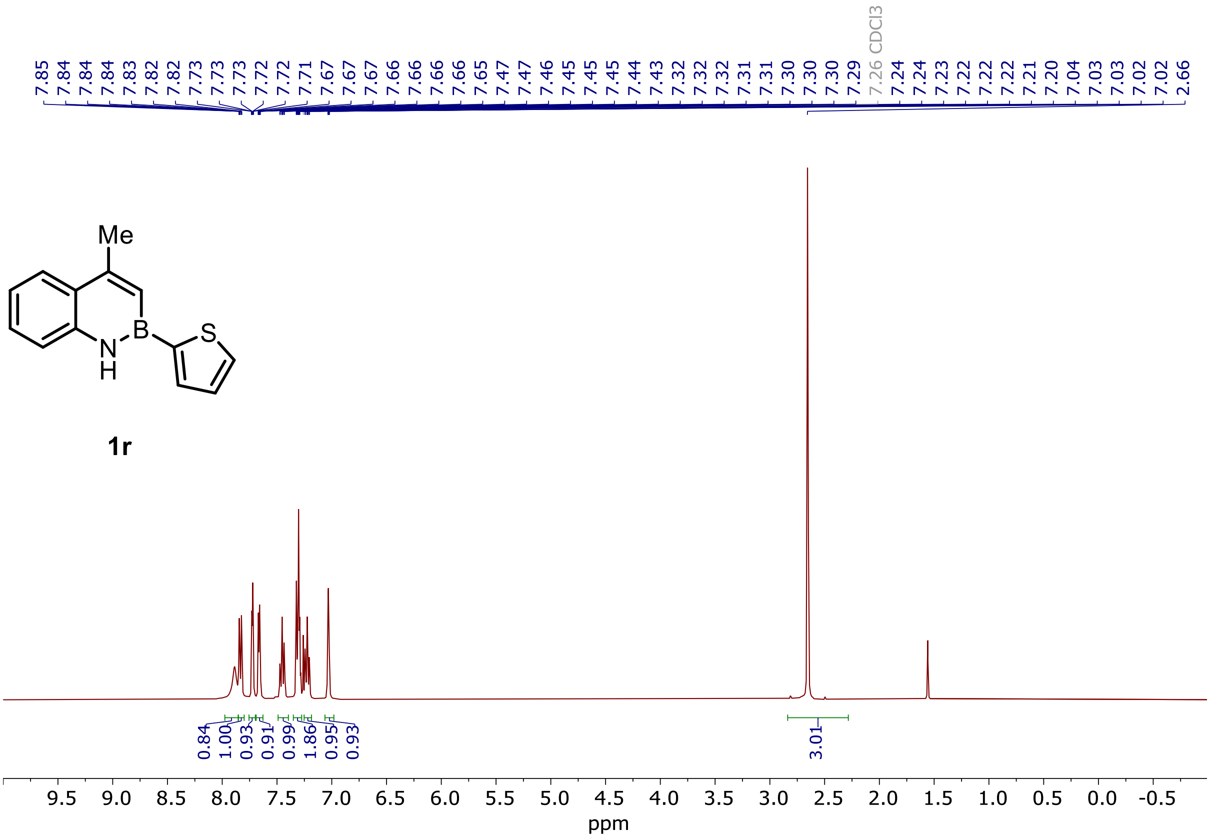
7.85
7.84
7.84
7.84
7.83
7.82
7.82
7.73
7.73
7.73
7.72
7.72
7.71
7.67
7.67
7.66
7.66
7.66
7.65
7.47
7.47
7.46
7.45
7.45
7.45
7.44
7.43
7.32
7.32
7.32
7.31
7.30
7.30
7.30
7.29
7.26 CDCl3
7.24
7.24
7.23
7.22
7.22
7.22
7.21
7.20
7.04
7.03
7.02
7.02
2.66
Me
N
H
B
S
1r
0.84
1.00
0.93
0.91
0.99
1.86
0.95
0.93
3.01
9.5
9.0
8.5
8.0
7.5
7.0
6.5
6.0
5.5
5.0
4.5
4.0
3.5
3.0
2.5
2.0
1.5
1.0
0.5
0.0
-0.5
ppm

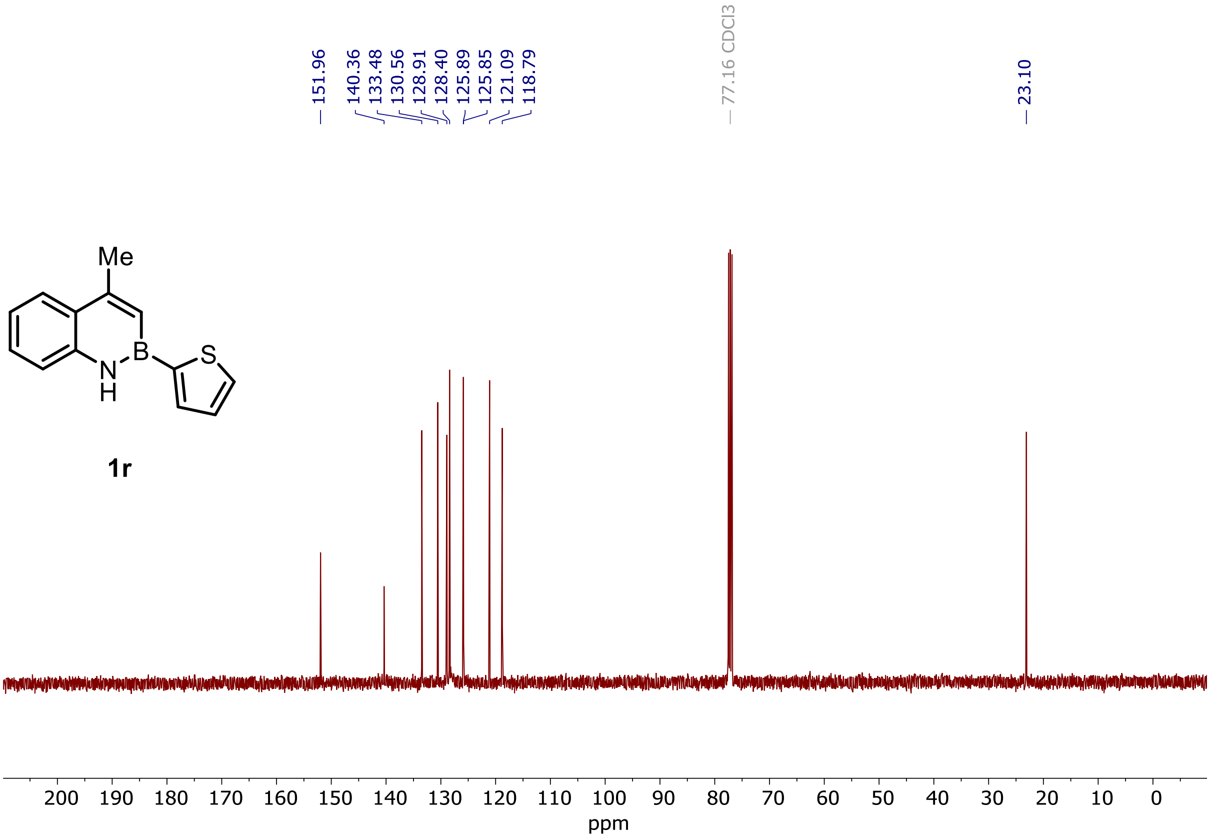
151.96
140.36
133.48
130.56
128.91
128.40
125.89
125.85
121.09
118.79
77.16 CDCl3
23.10
Me
N
H
B
S
1r
200
190
180
170
160
150
140
130
120
110
100
90
80
70
60
50
40
30
20
10
0
ppm

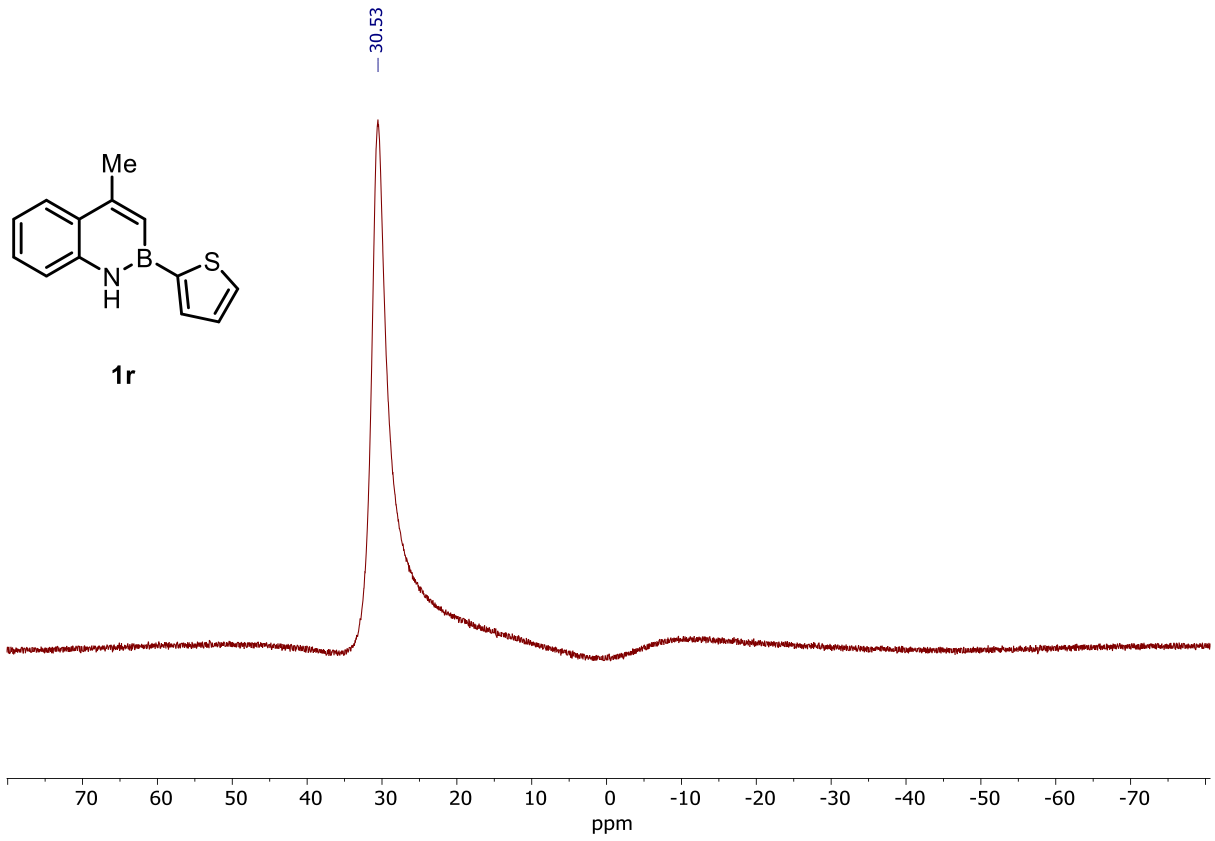

30.53
Me
B
S
N
H
1r
70 60 50 40 30 20 10 0 -10 -20 -30 -40 -50 -60 -70
ppm

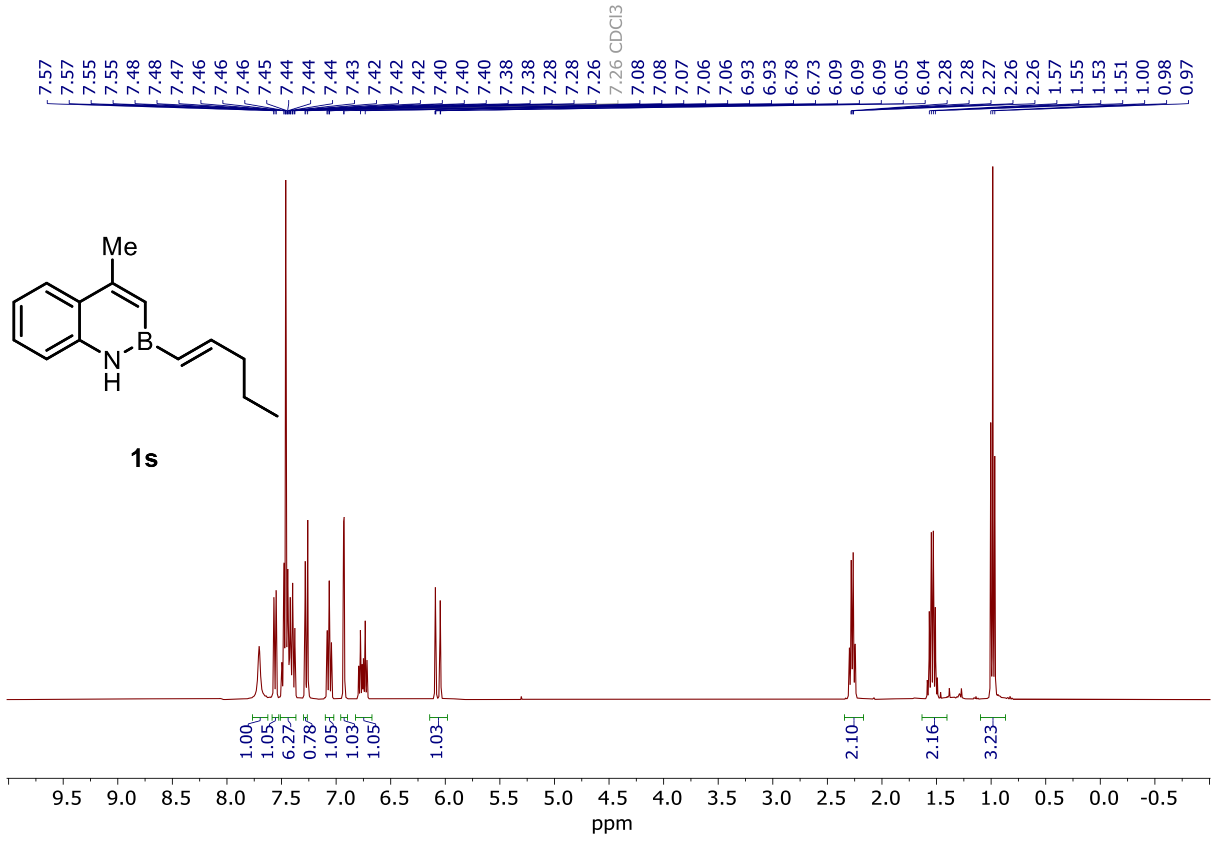

7.57 7.57 7.55 7.55 7.48 7.48 7.47 7.46 7.46 7.45 7.44 7.44 7.44 7.43 7.42 7.42 7.42 7.40 7.40 7.40 7.38 7.38 7.28 7.28 7.26 7.26 CDCl3 7.08 7.08 7.07 7.06 7.06 6.93 6.93 6.78 6.73 6.09 6.09 6.09 6.05 6.04 2.28 2.28 2.27 2.26 2.26 1.57 1.55 1.53 1.51 1.00 0.98 0.97
Me
B
N
H
1s
1.00 1.05 6.27 0.78 1.05 1.03 1.05 1.03 2.10 2.16 3.23
9.5 9.0 8.5 8.0 7.5 7.0 6.5 6.0 5.5 5.0 4.5 4.0 3.5 3.0 2.5 2.0 1.5 1.0 0.5 0.0 -0.5
ppm

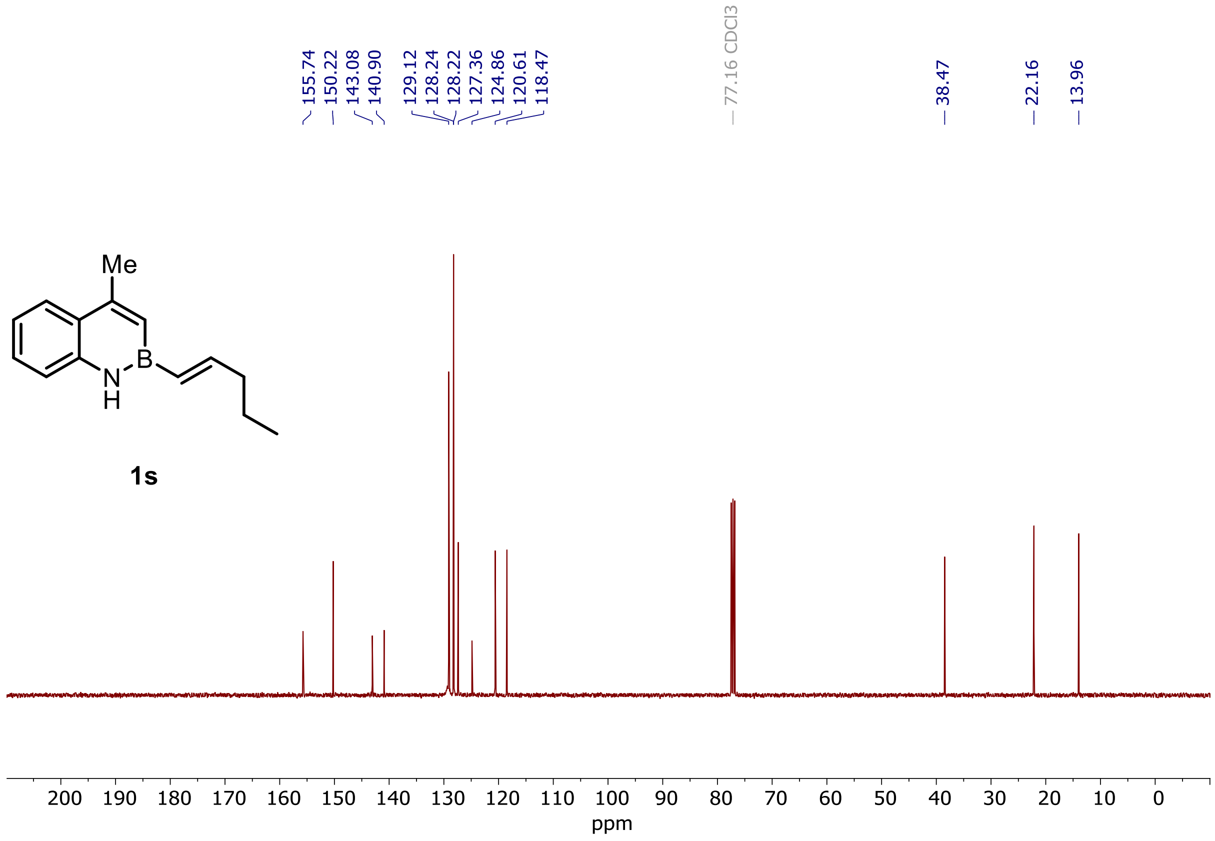
155.74
150.22
143.08
140.90
129.12
128.24
128.22
127.36
124.86
120.61
118.47
77.16 CDCl3
38.47
22.16
13.96
Me
N
H
B
1s
200 190 180 170 160 150 140 130 120 110 100 90 80 70 60 50 40 30 20 10 0
ppm

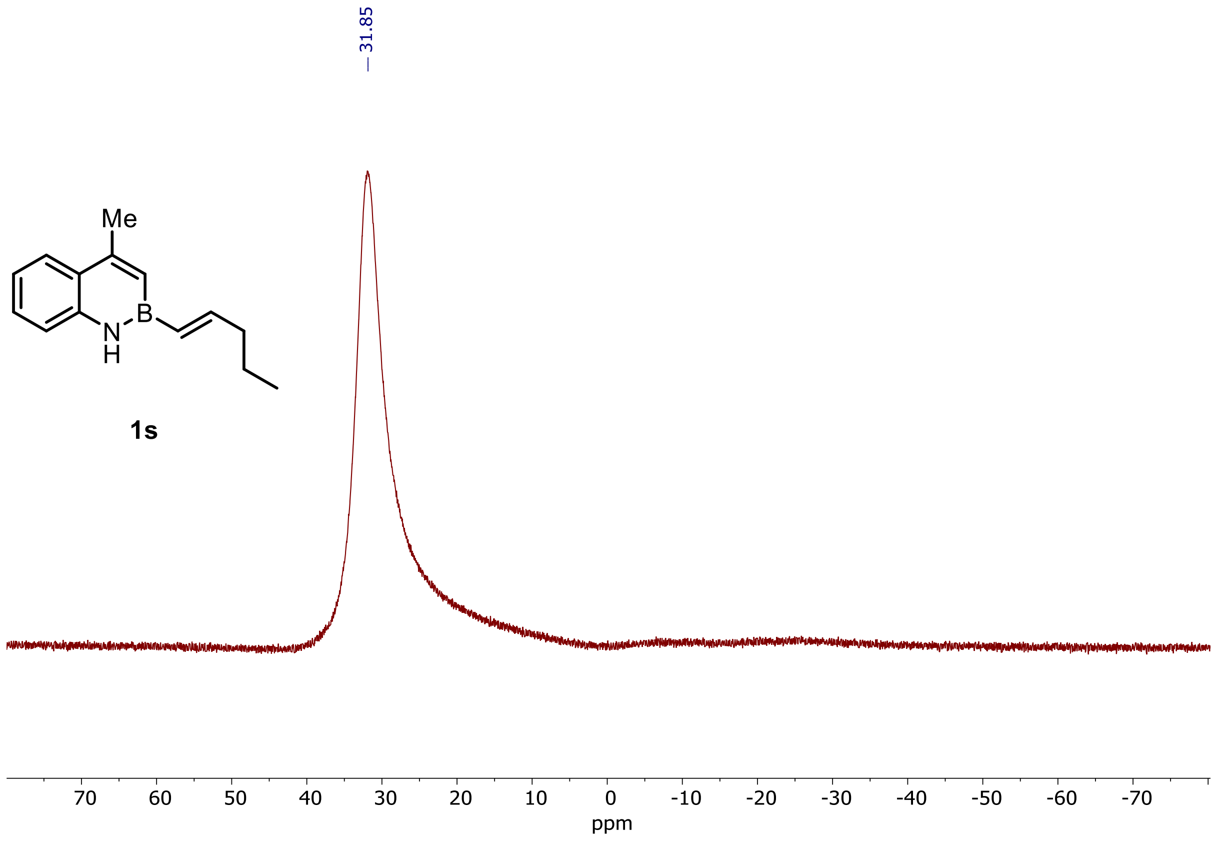
31.85
Me
N
H
B
1s
70 60 50 40 30 20 10 0 -10 -20 -30 -40 -50 -60 -70
ppm

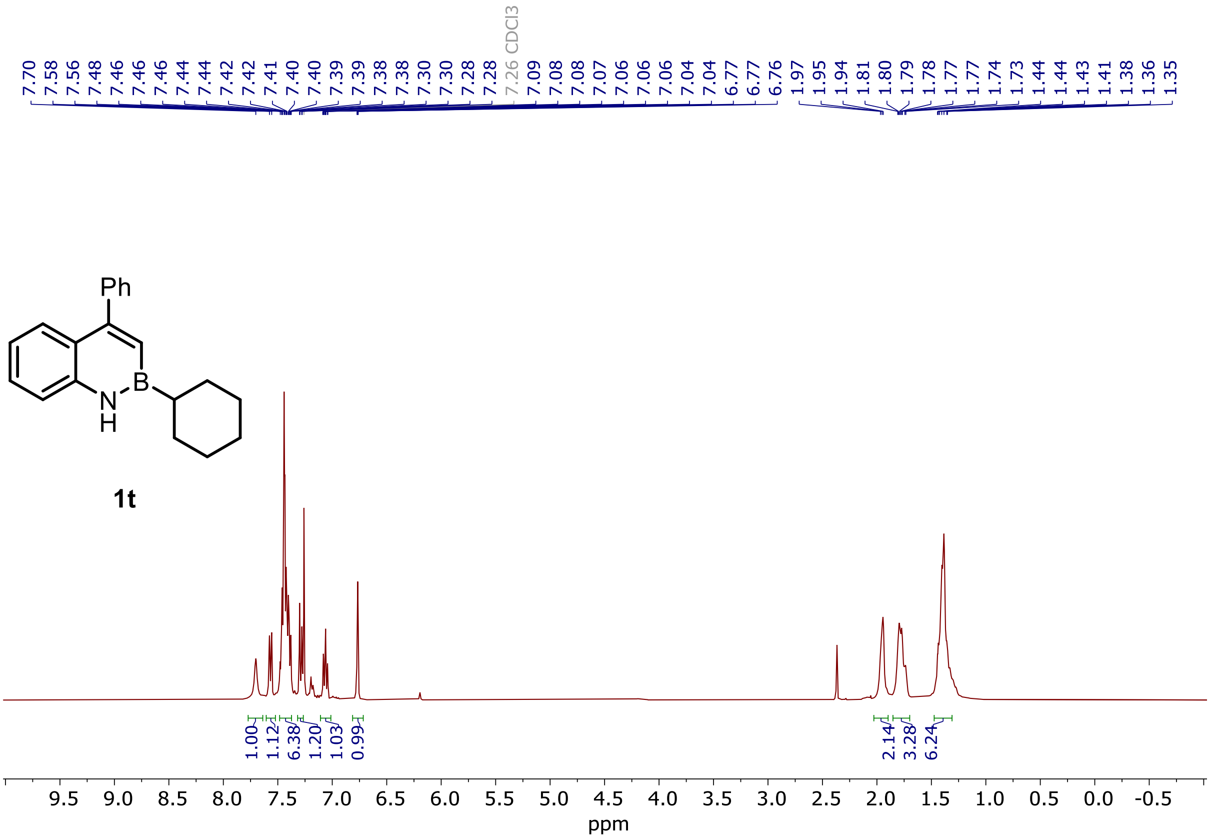
7.70
7.58
7.56
7.48
7.46
7.46
7.46
7.44
7.44
7.42
7.42
7.41
7.40
7.40
7.39
7.39
7.38
7.38
7.30
7.30
7.28
7.28
7.26 CDCl3
7.09
7.08
7.08
7.07
7.06
7.06
7.06
7.04
7.04
6.77
6.77
6.76
1.97
1.95
1.94
1.81
1.80
1.79
1.78
1.77
1.77
1.74
1.73
1.44
1.44
1.43
1.41
1.38
1.36
1.35
Ph
N
H
B
1t
1.00
1.12
6.38
1.20
1.03
0.99
2.14
3.28
6.24
9.5 9.0 8.5 8.0 7.5 7.0 6.5 6.0 5.5 5.0 4.5 4.0 3.5 3.0 2.5 2.0 1.5 1.0 0.5 0.0 -0.5
ppm

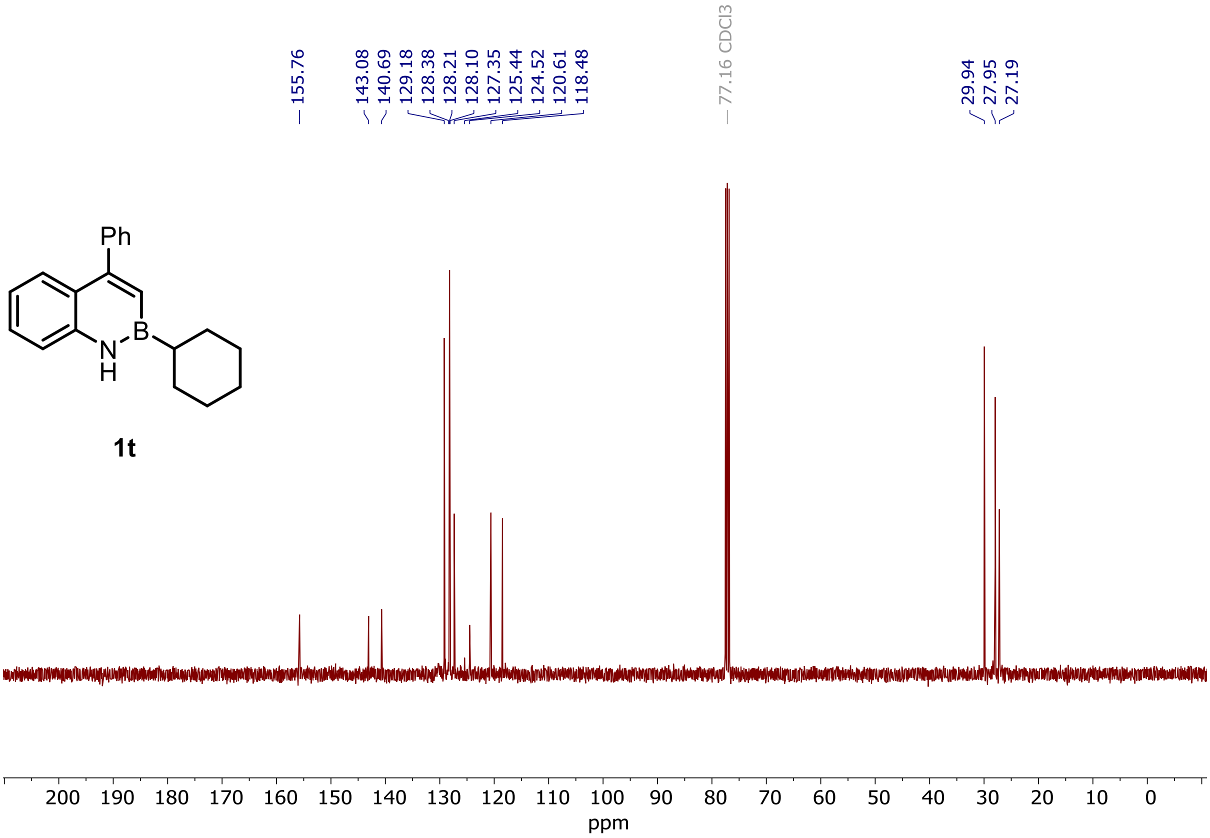
155.76
143.08
140.69
129.18
128.38
128.21
128.10
127.35
125.44
124.52
120.61
118.48
77.16 CDCl3
29.94
27.95
27.19
Ph
N
H
B
1t
200 190 180 170 160 150 140 130 120 110 100 90 80 70 60 50 40 30 20 10 0
ppm

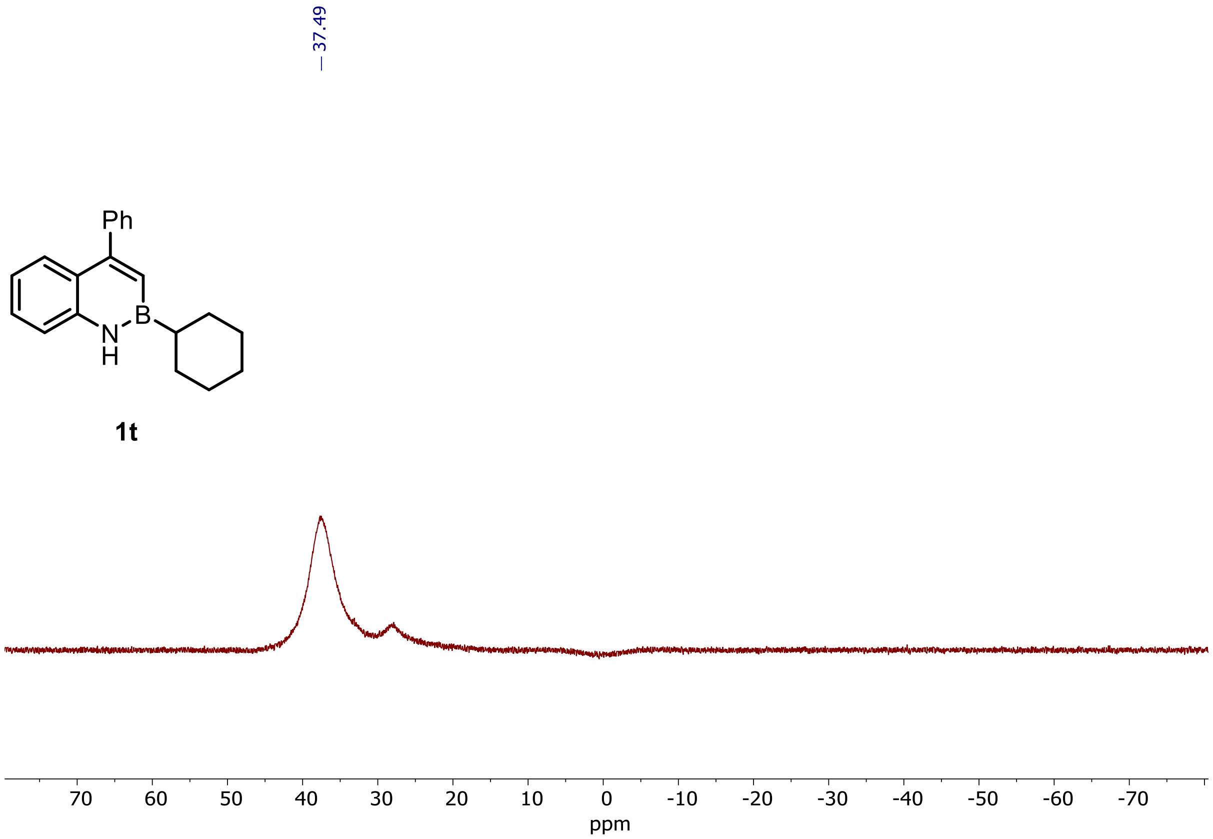
— 37.49
70 60 50 40 30 20 10 0 -10 -20 -30 -40 -50 -60 -70
ppm
Ph
N
H
B

1t

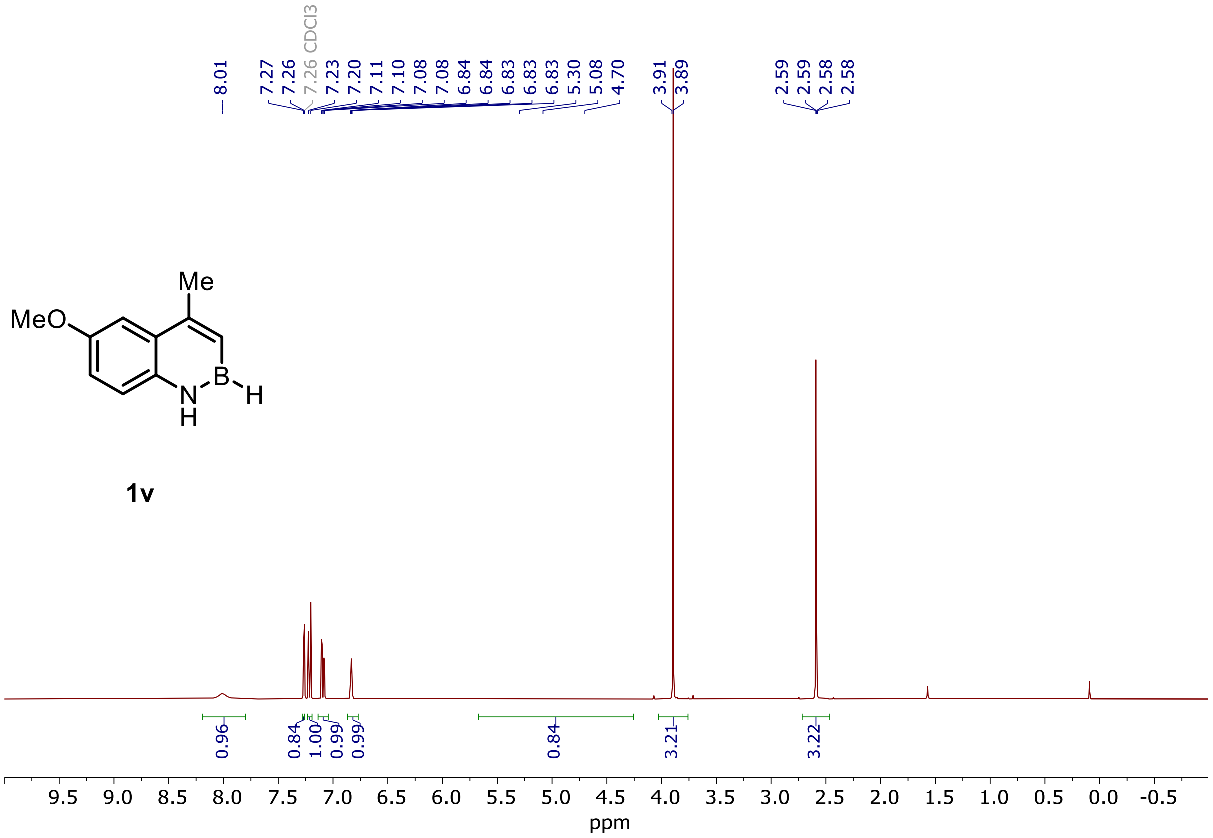
8.01
7.27
7.26
7.26 CDCl3
7.23
7.20
7.11
7.10
7.08
7.08
6.84
6.83
6.83
6.83
5.30
5.08
4.70
3.91
3.89
2.59
2.59
2.58
2.58
0.96
0.84
1.00
0.99
0.99
0.84
3.21
3.22
9.5 9.0 8.5 8.0 7.5 7.0 6.5 6.0 5.5 5.0 4.5 4.0 3.5 3.0 2.5 2.0 1.5 1.0 0.5 0.0 -0.5
ppm
Me
MeO
N
H
B
H

1v

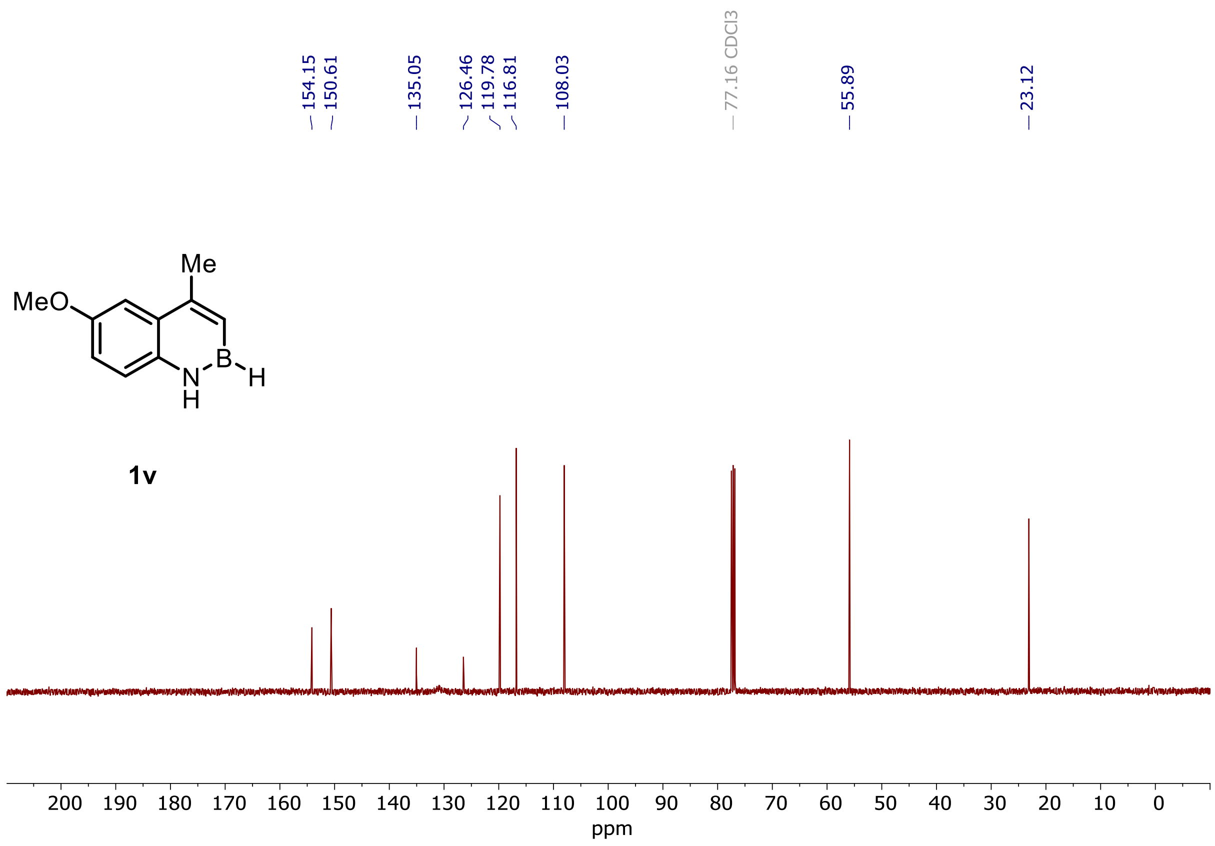

154.15
150.61
135.05
126.46
119.78
116.81
108.03
77.16 CDCl3
55.89
23.12
MeO
Me
N
H
B
H
1v
200 190 180 170 160 150 140 130 120 110 100 90 80 70 60 50 40 30 20 10 0
ppm

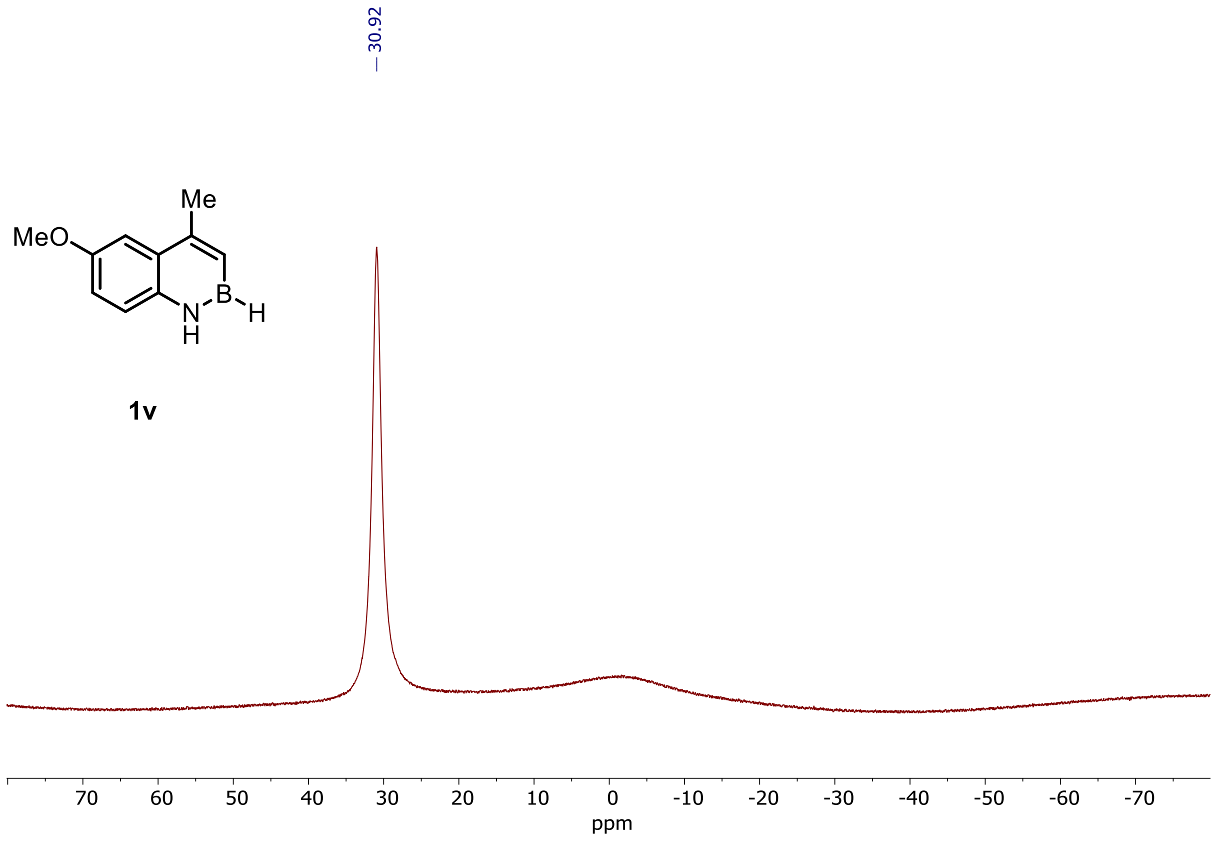

30.92
MeO
Me
N
H
B
H
1v
70 60 50 40 30 20 10 0 -10 -20 -30 -40 -50 -60 -70
ppm

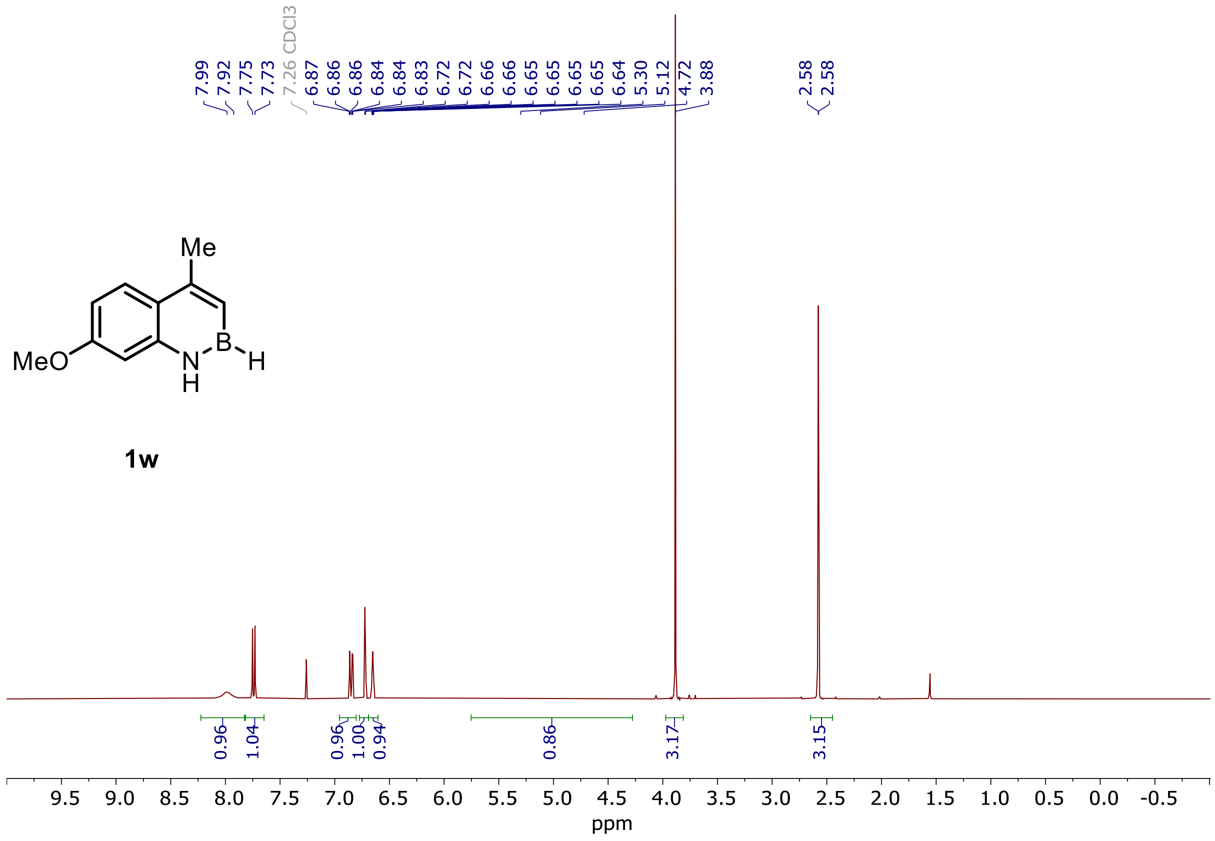
7.99
7.92
7.75
7.73
7.26 CDCl3
6.87
6.86
6.86
6.84
6.84
6.83
6.72
6.72
6.66
6.66
6.65
6.65
6.65
6.65
6.64
5.30
5.12
4.72
3.88
2.58
2.58
Me
MeO
B
H
N
H
1w
0.96
1.04
0.96
1.00
0.94
0.86
3.17
3.15
9.5 9.0 8.5 8.0 7.5 7.0 6.5 6.0 5.5 5.0 4.5 4.0 3.5 3.0 2.5 2.0 1.5 1.0 0.5 0.0 -0.5
ppm

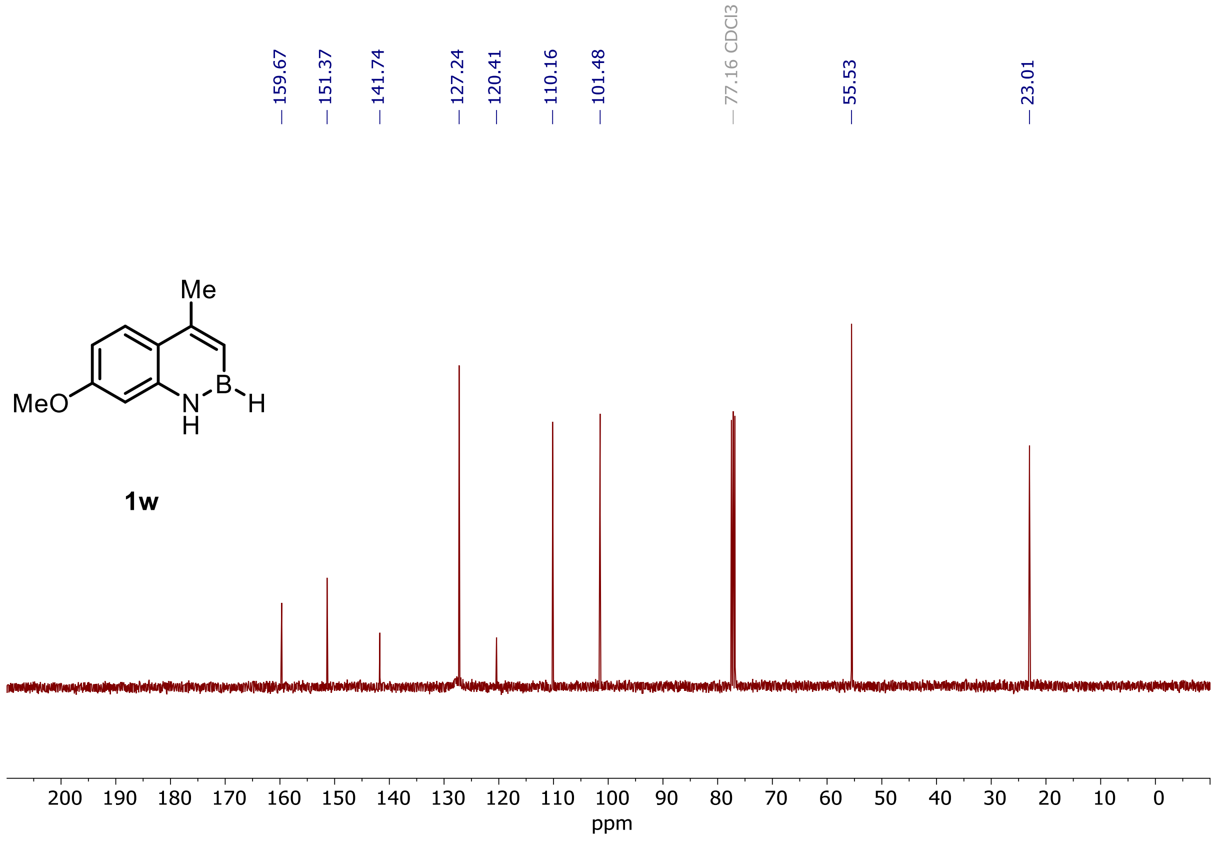
159.67
151.37
141.74
127.24
120.41
110.16
101.48
77.16 CDCl3
55.53
23.01
Me
MeO
B
H
N
H
1w
200 190 180 170 160 150 140 130 120 110 100 90 80 70 60 50 40 30 20 10 0
ppm

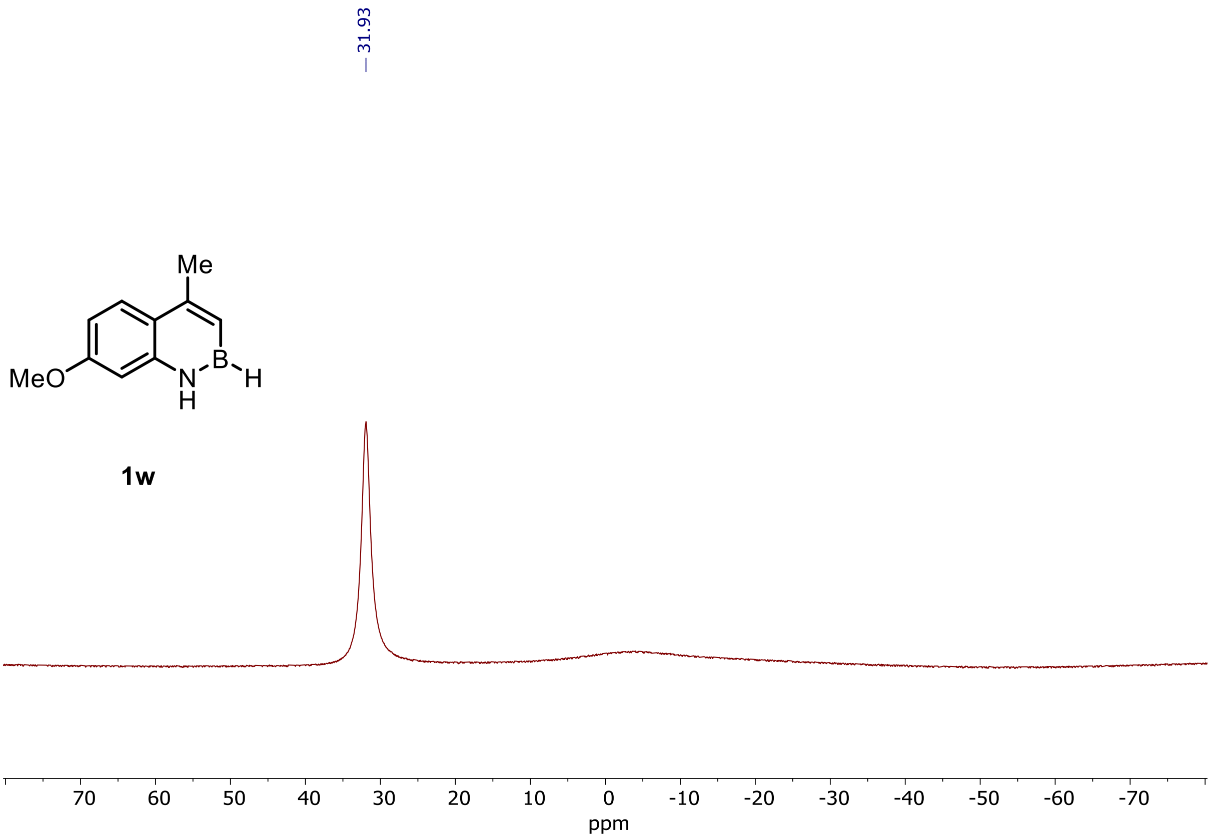

31.93
Me
MeO
N
H
B
H
1w
70 60 50 40 30 20 10 0 -10 -20 -30 -40 -50 -60 -70
ppm

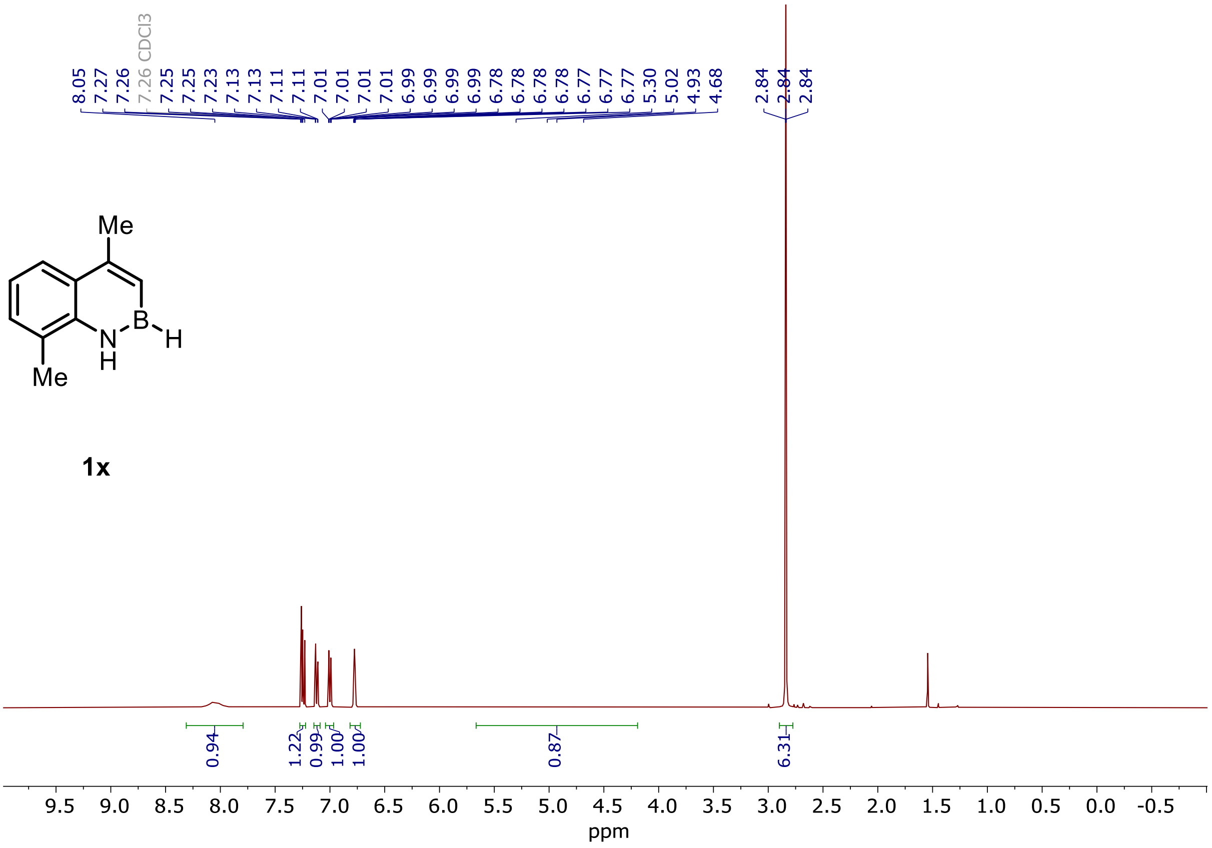

8.05 7.27 7.26 7.26 CDCl3 7.25 7.25 7.23 7.13 7.13 7.11 7.11 7.01 7.01 7.01 7.01 6.99 6.99 6.99 6.99 6.78 6.78 6.78 6.77 6.77 6.77 5.30 5.02 4.93 4.68
2.84 2.84 2.84
Me
Me
N
H
B
H
1x
0.94 1.22 0.99 1.00 1.00 0.87 6.31
9.5 9.0 8.5 8.0 7.5 7.0 6.5 6.0 5.5 5.0 4.5 4.0 3.5 3.0 2.5 2.0 1.5 1.0 0.5 0.0 -0.5
ppm

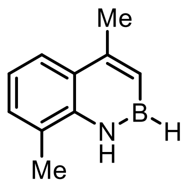
Me
B
H
N
H
Me

1x

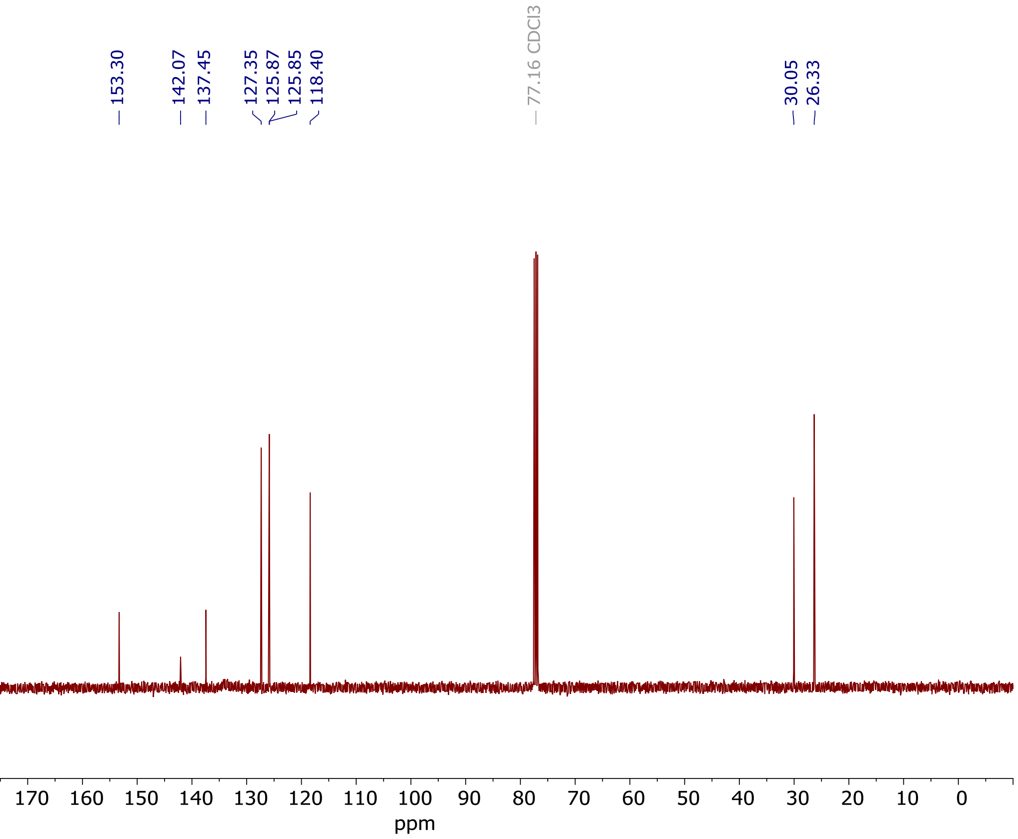
153.30
142.07
137.45
127.35
125.87
125.85
118.40
77.16 CDCl3
30.05
26.33
200 190 180 170 160 150 140 130 120 110 100 90 80 70 60 50 40 30 20 10 0
ppm

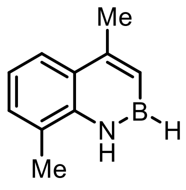
Me
B
H
N
H
Me

1x

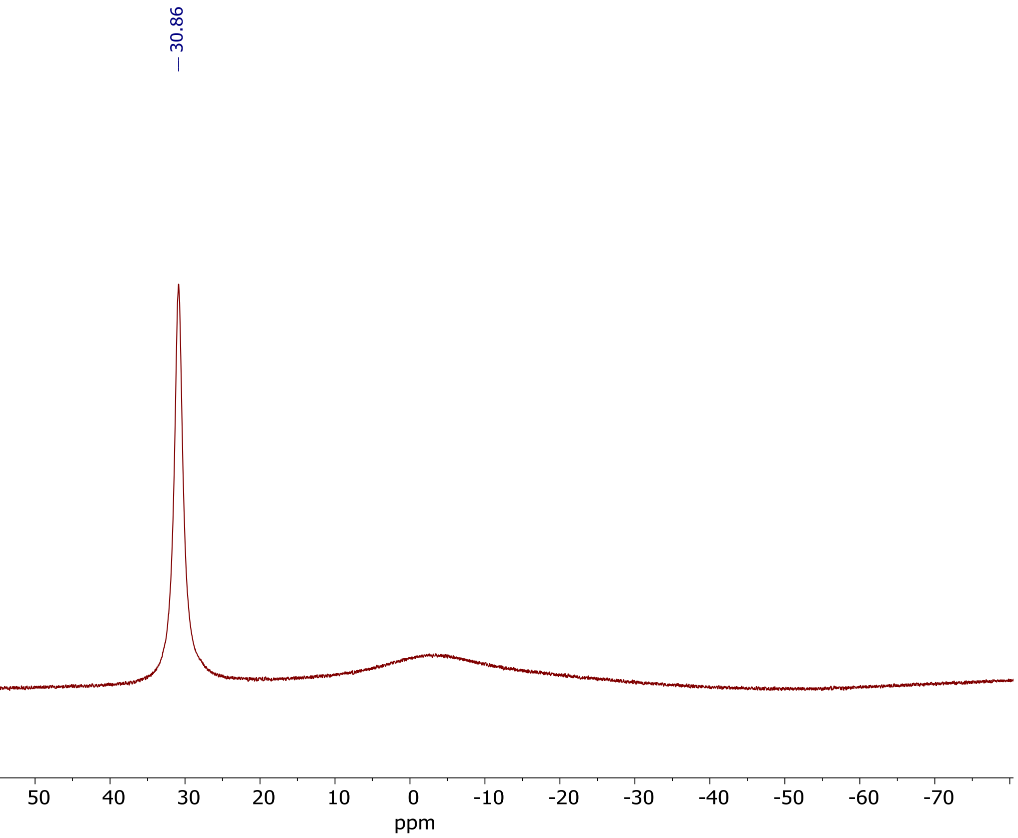
30.86
70 60 50 40 30 20 10 0 -10 -20 -30 -40 -50 -60 -70
ppm

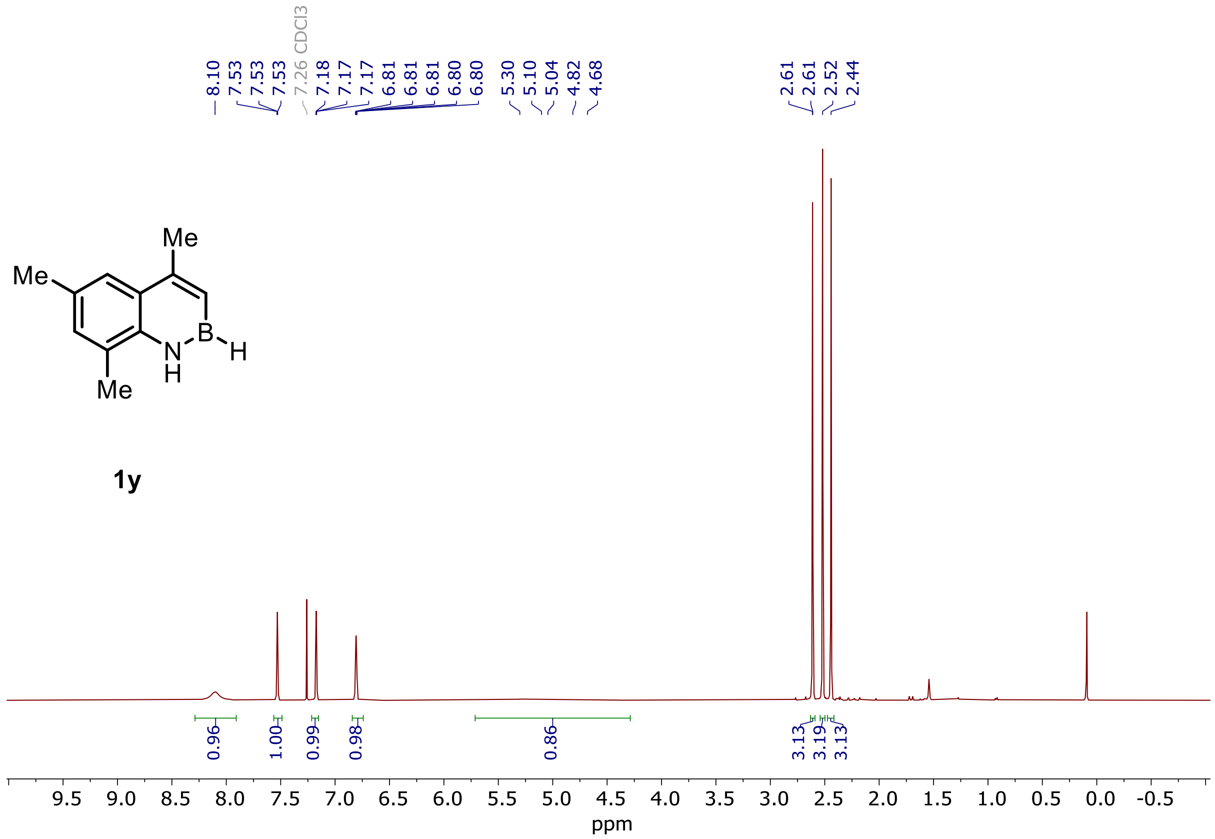
8.10
7.53
7.53
7.53
7.26 CDCl3
7.18
7.17
7.17
6.81
6.81
6.81
6.80
6.80
5.30
5.10
5.04
4.82
4.68
2.61
2.61
2.52
2.44
Me
Me
B
H
N
H
Me
1y
0.96
1.00
0.99
0.98
0.86
3.13
3.19
3.13
9.5 9.0 8.5 8.0 7.5 7.0 6.5 6.0 5.5 5.0 4.5 4.0 3.5 3.0 2.5 2.0 1.5 1.0 0.5 0.0 -0.5
ppm

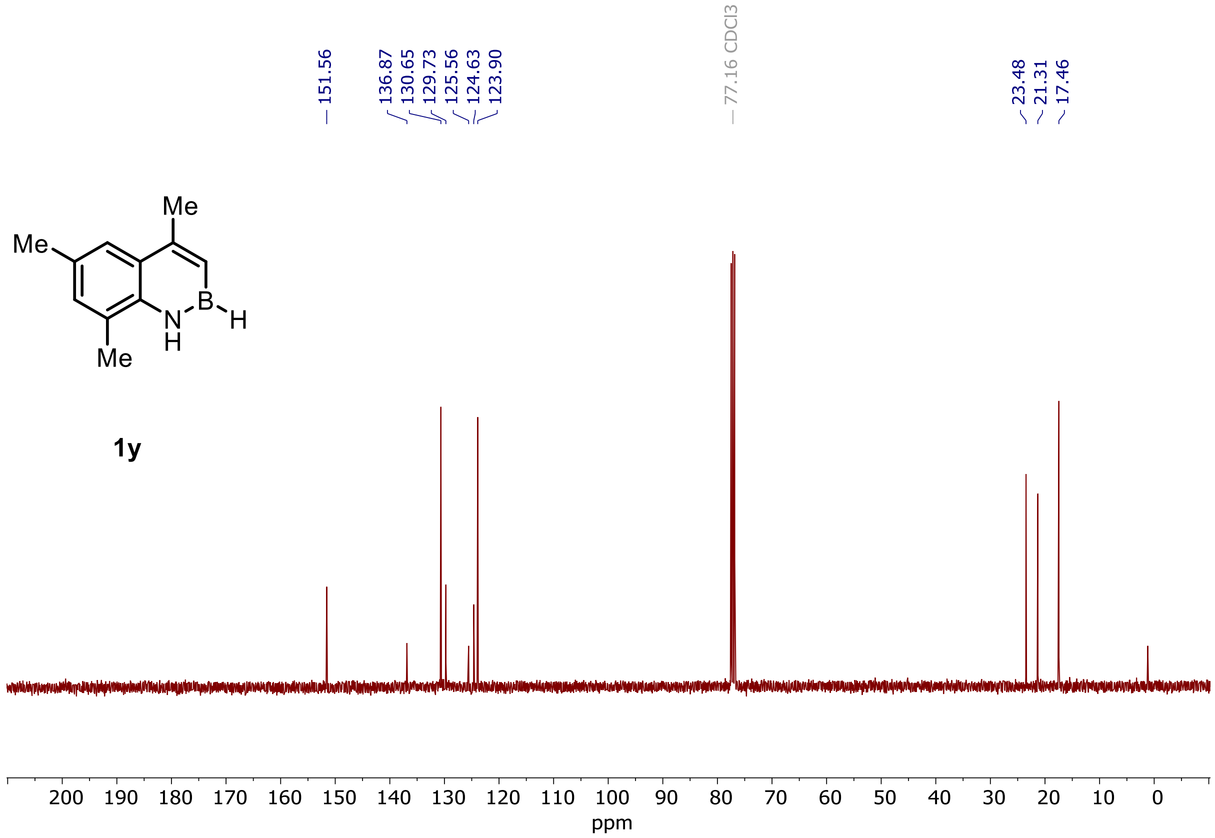
151.56
136.87
130.65
129.73
125.56
124.63
123.90
77.16 CDCl3
23.48
21.31
17.46
Me
Me
B
H
N
H
Me
1y
200 190 180 170 160 150 140 130 120 110 100 90 80 70 60 50 40 30 20 10 0
ppm

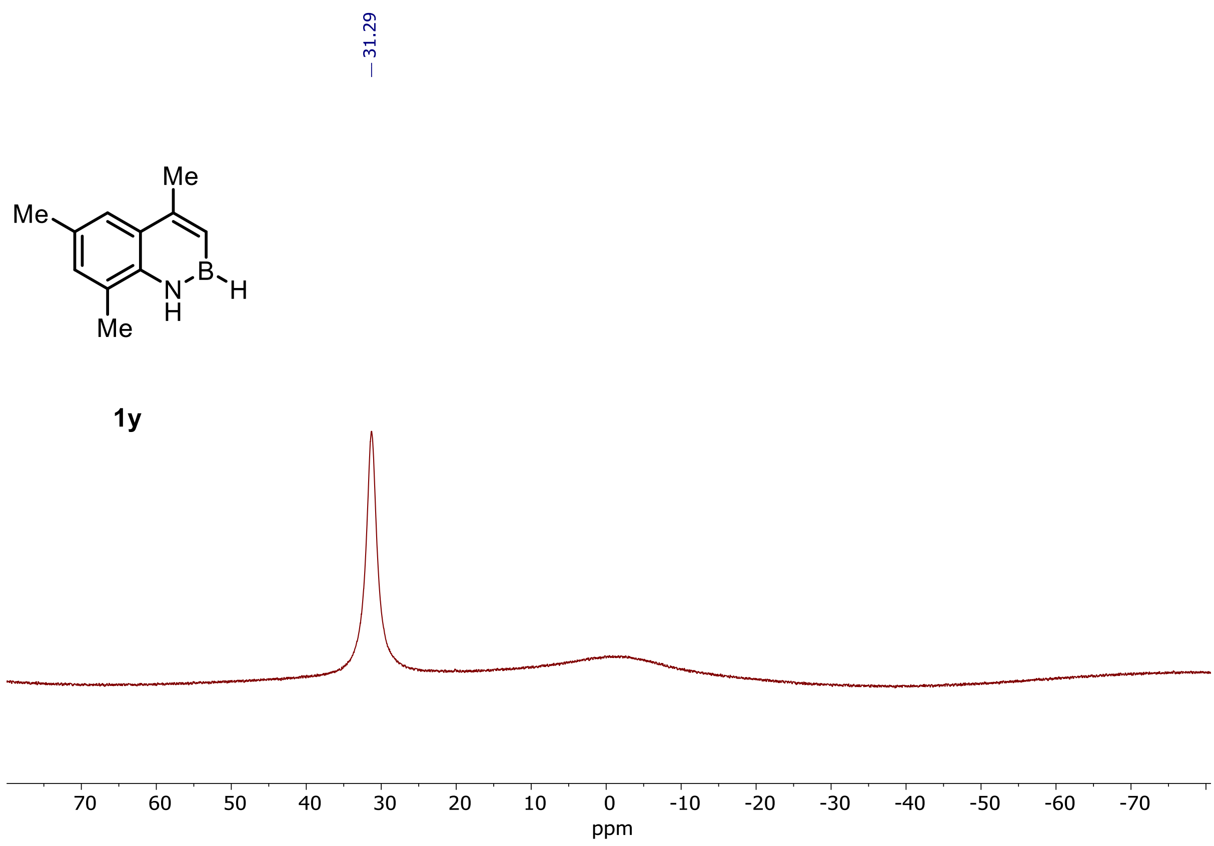

31.29
Me
Me
B
H
N
H
Me
1y
70 60 50 40 30 20 10 0 -10 -20 -30 -40 -50 -60 -70
ppm

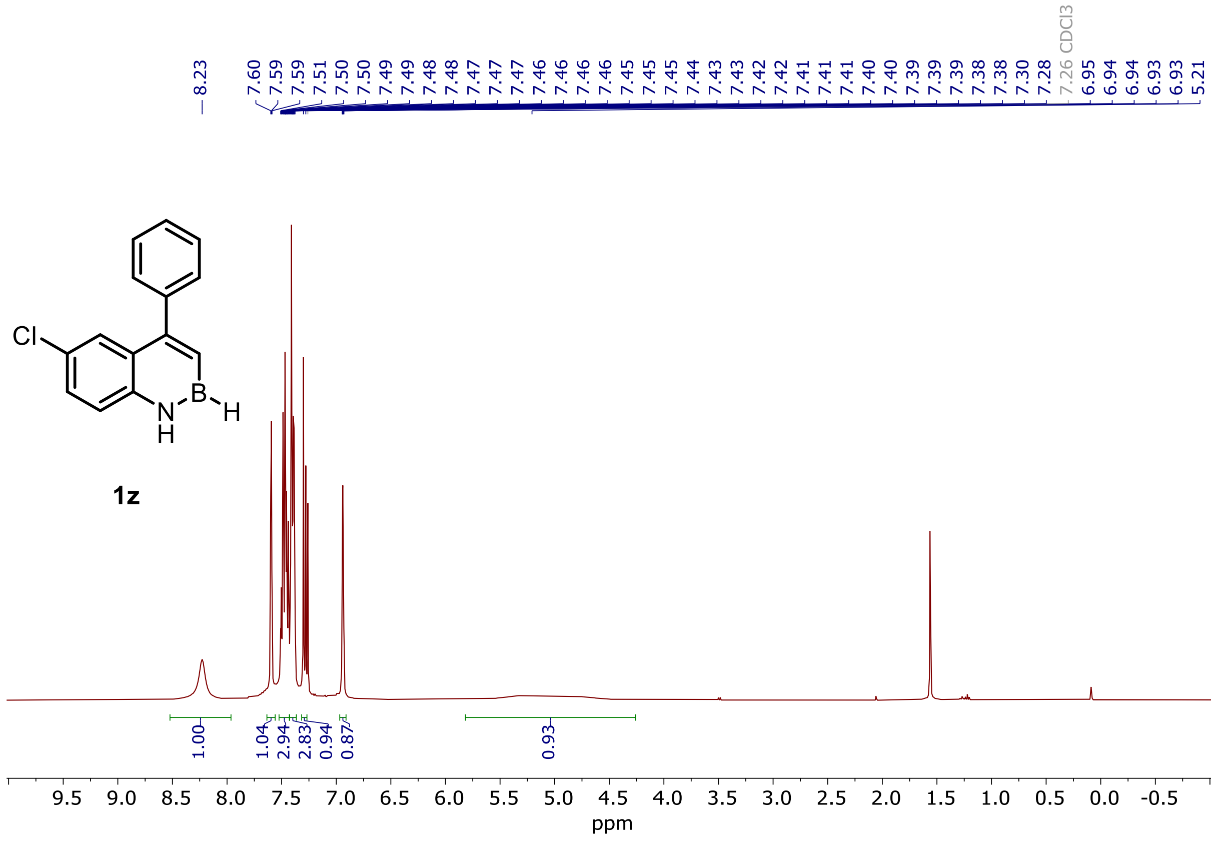

8.23
7.60 7.59 7.59 7.51 7.50 7.50 7.49 7.49 7.48 7.48 7.47 7.47 7.47 7.46 7.46 7.46 7.45 7.45 7.45 7.44 7.43 7.43 7.42 7.42 7.41 7.41 7.41 7.40 7.40 7.39 7.39 7.39 7.38 7.38 7.30 7.28
7.26 CDCl3
6.95 6.94 6.94 6.93 6.93 5.21
Cl
B
H
N
H
1z
1.00
1.04
2.94
2.83
0.94
0.87
0.93
9.5 9.0 8.5 8.0 7.5 7.0 6.5 6.0 5.5 5.0 4.5 4.0 3.5 3.0 2.5 2.0 1.5 1.0 0.5 0.0 -0.5
ppm

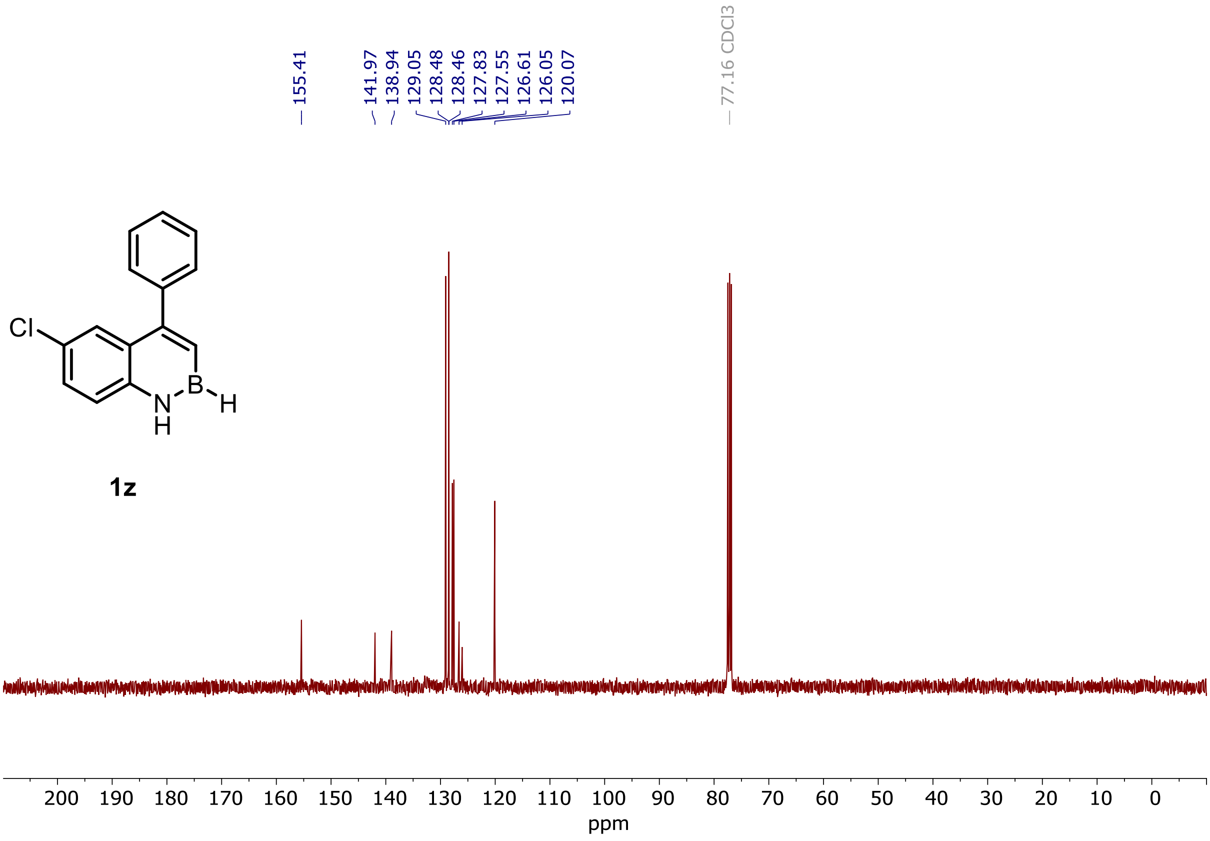

155.41
141.97
138.94
129.05
128.48
128.46
127.83
127.55
126.61
126.05
120.07
77.16 CDCl3
200
190
180
170
160
150
140
130
120
110
100
90
80
70
60
50
40
30
20
10
0
ppm

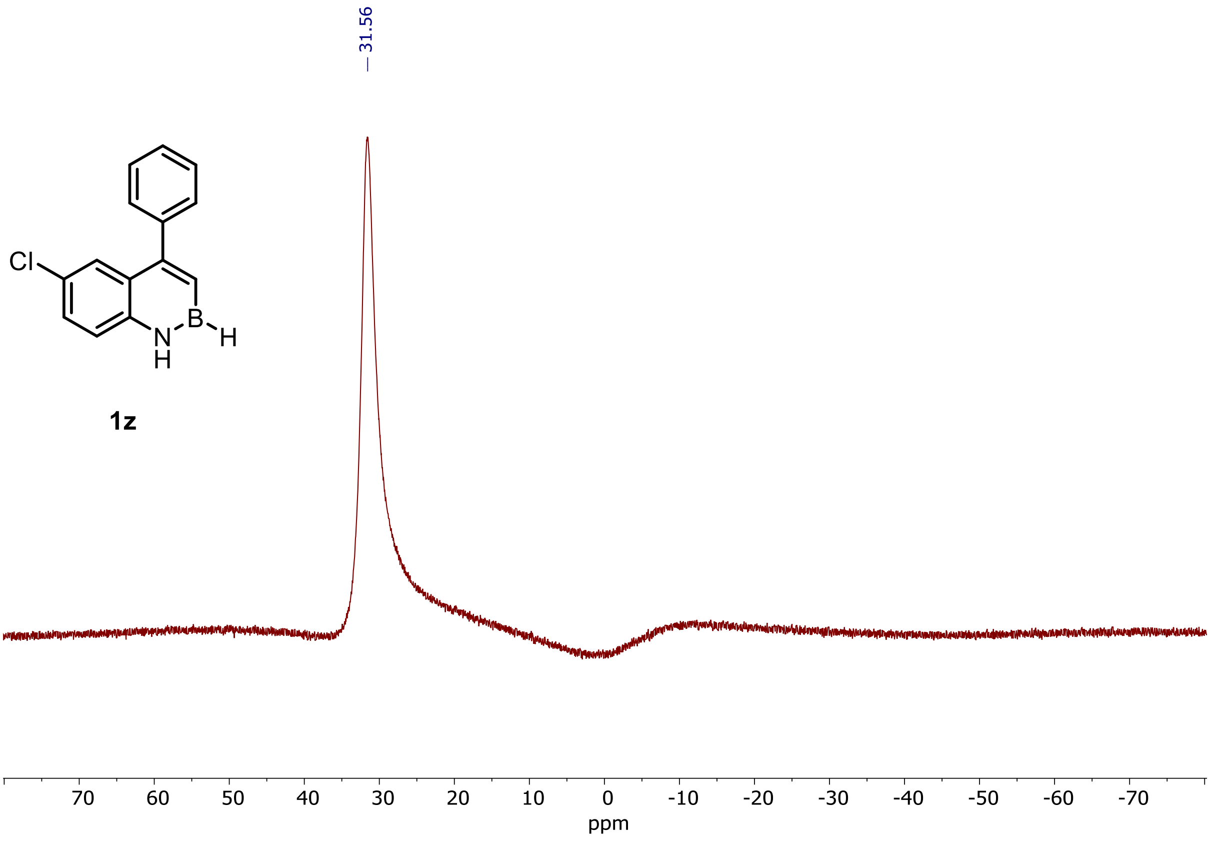

31.56
70
60
50
40
30
20
10
0
-10
-20
-30
-40
-50
-60
-70
ppm

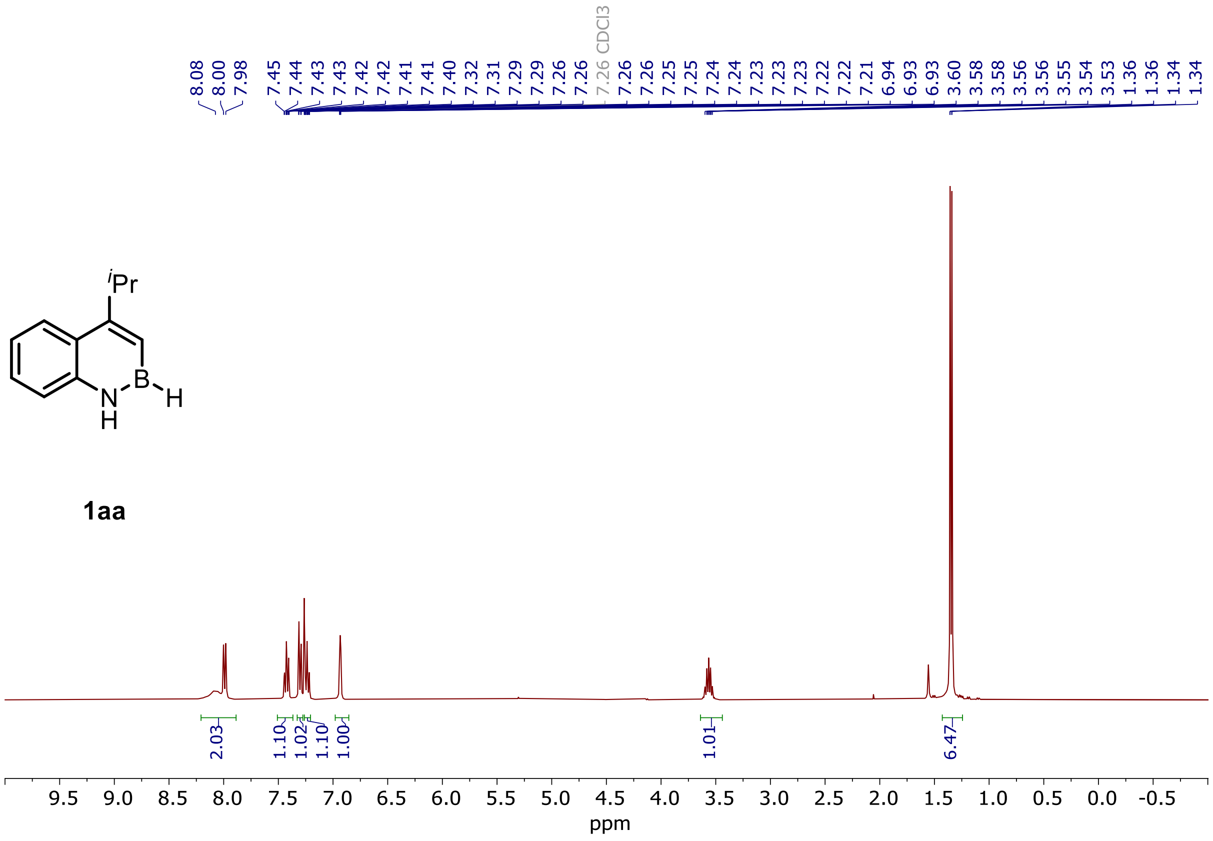
8.08
8.00
7.98
7.45
7.44
7.43
7.43
7.42
7.42
7.41
7.41
7.40
7.32
7.31
7.29
7.29
7.26
7.26
7.26 CDCl3
7.26
7.26
7.25
7.25
7.24
7.24
7.23
7.23
7.23
7.22
7.22
7.21
6.94
6.93
6.93
3.60
3.58
3.58
3.56
3.56
3.55
3.54
3.53
1.36
1.36
1.34
1.34
iPr
N
H
B
H
2.03
1.10
1.02
1.10
1.00
1.01
6.47
9.5 9.0 8.5 8.0 7.5 7.0 6.5 6.0 5.5 5.0 4.5 4.0 3.5 3.0 2.5 2.0 1.5 1.0 0.5 0.0 -0.5
ppm

1aa

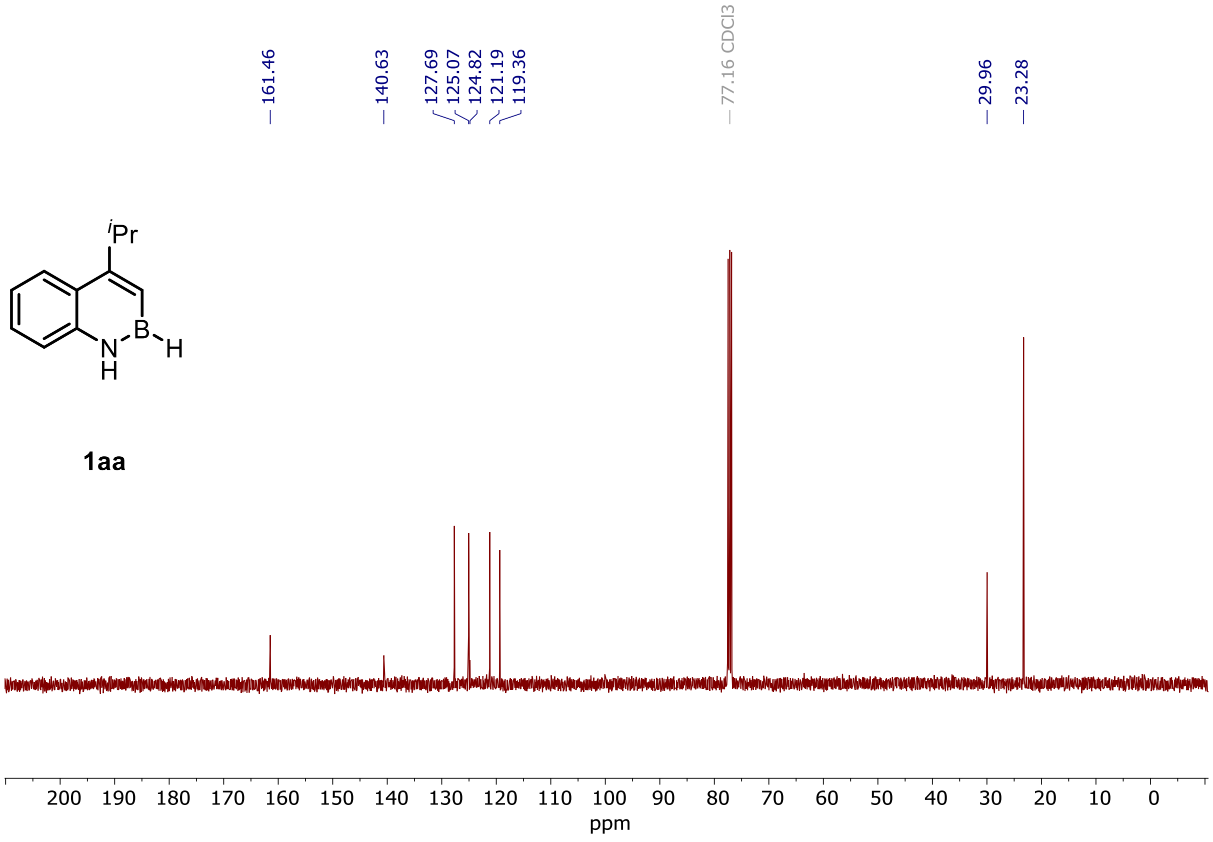
161.46
140.63
127.69
125.07
124.82
121.19
119.36
77.16 CDCl3
29.96
23.28
iPr
N
H
B
H
200 190 180 170 160 150 140 130 120 110 100 90 80 70 60 50 40 30 20 10 0
ppm

1aa

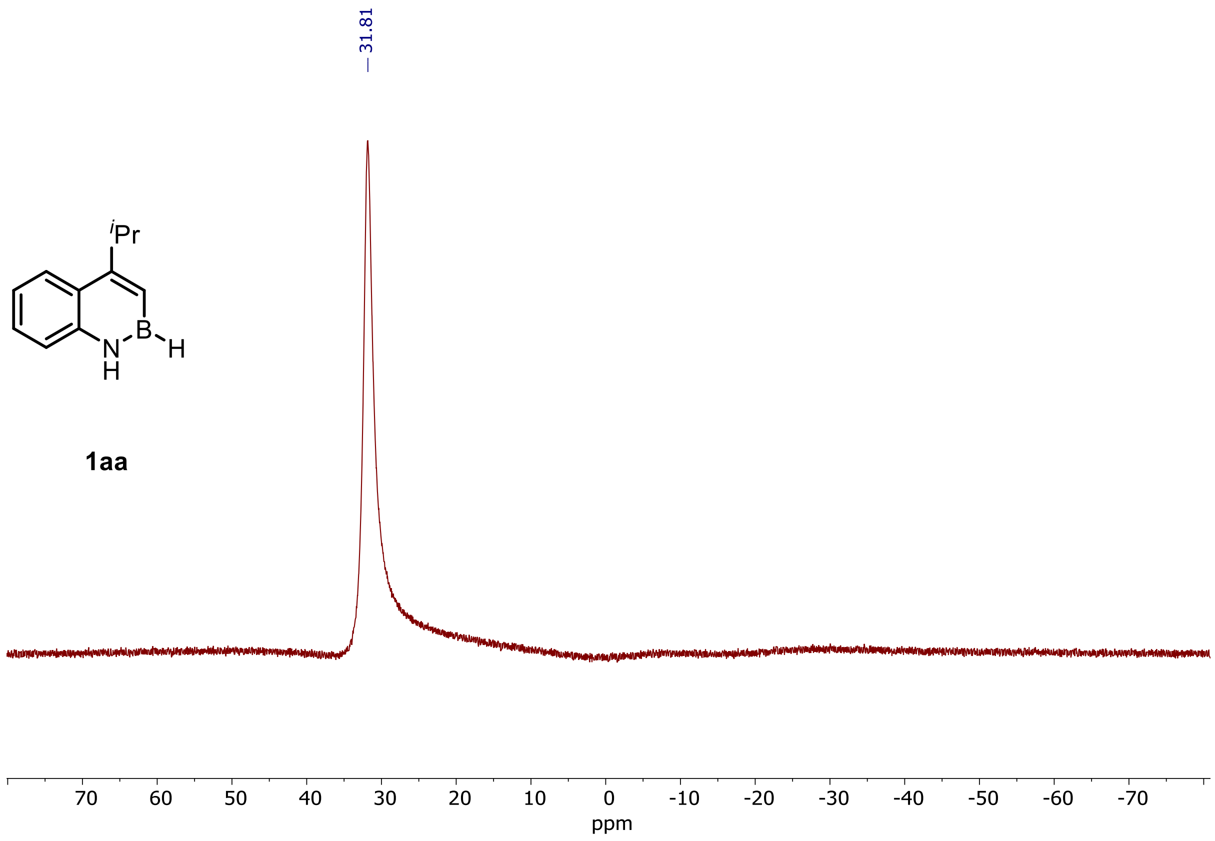

31.81
iPr
N
H
B
H
1aa
70 60 50 40 30 20 10 0 -10 -20 -30 -40 -50 -60 -70
ppm

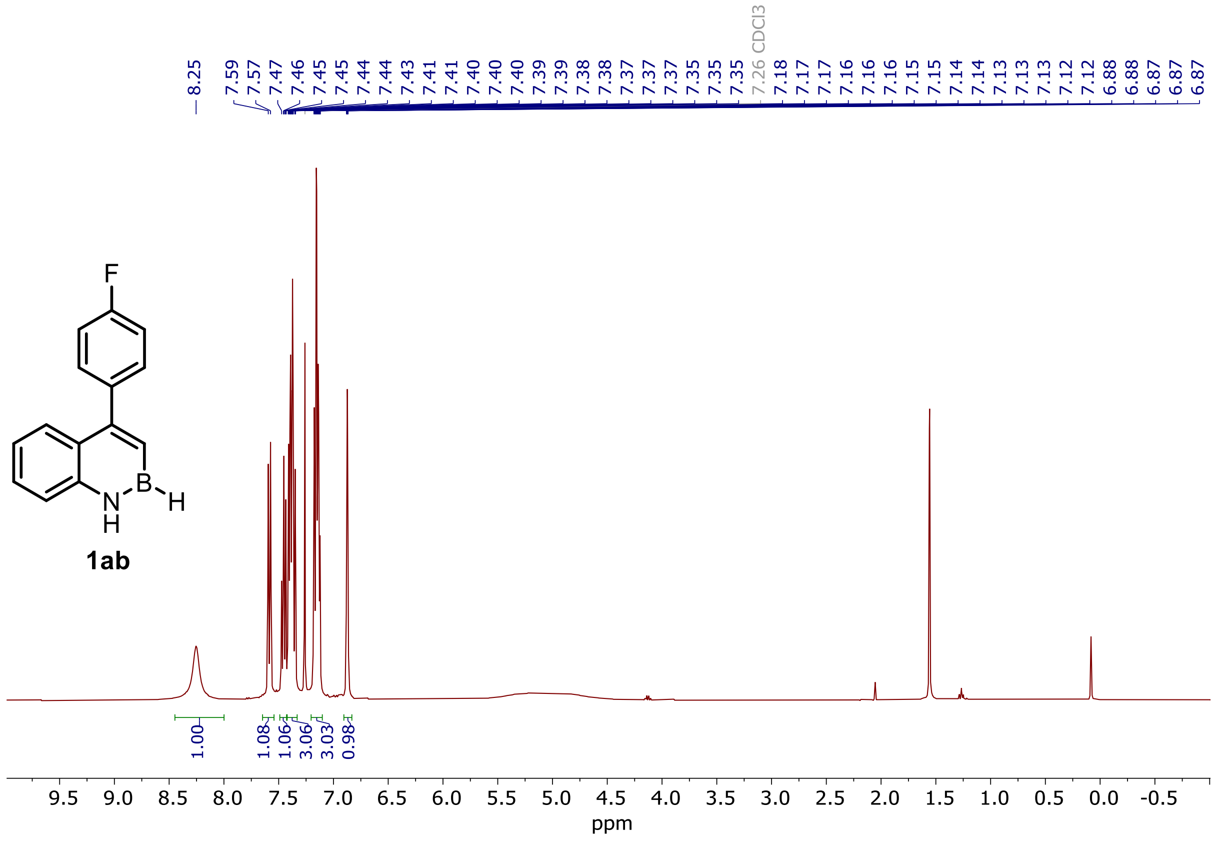

8.25
7.59 7.57 7.47 7.46 7.45 7.45 7.44 7.44 7.43 7.41 7.41 7.40 7.40 7.40 7.39 7.39 7.38 7.38 7.37 7.37 7.37 7.35 7.35 7.35
7.26 CDCl3
7.18 7.17 7.17 7.16 7.16 7.16 7.15 7.15 7.14 7.14 7.13 7.13 7.13 7.12 7.12 6.88 6.88 6.87 6.87 6.87
F
N
H
B
H
1ab
1.00
1.08
1.06
3.06
3.03
0.98
9.5 9.0 8.5 8.0 7.5 7.0 6.5 6.0 5.5 5.0 4.5 4.0 3.5 3.0 2.5 2.0 1.5 1.0 0.5 0.0 -0.5
ppm

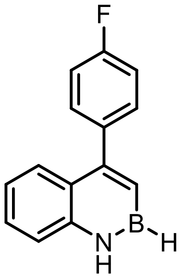

1ab

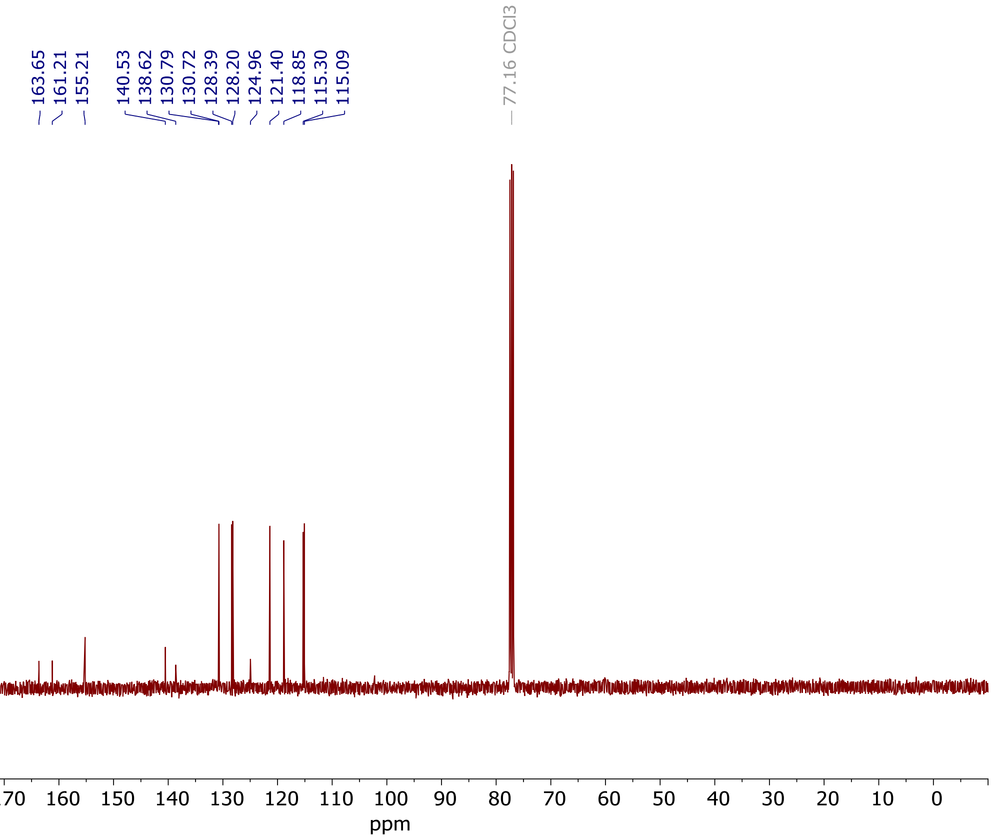

163.65
161.21
155.21
140.53
138.62
130.79
130.72
128.39
128.20
124.96
121.40
118.85
115.30
115.09
77.16 CDCl3
200 190 180 170 160 150 140 130 120 110 100 90 80 70 60 50 40 30 20 10 0
ppm

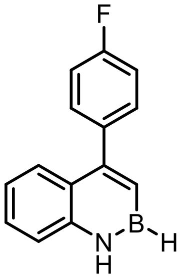

1ab

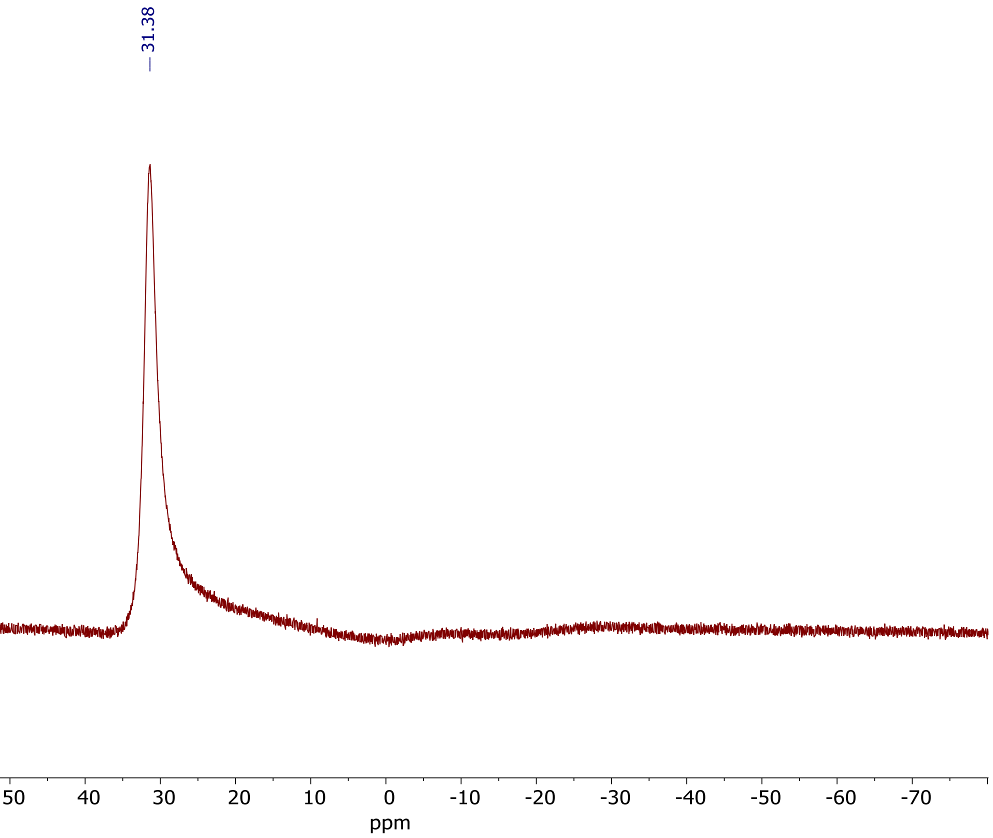

31.38
70 60 50 40 30 20 10 0 -10 -20 -30 -40 -50 -60 -70
ppm

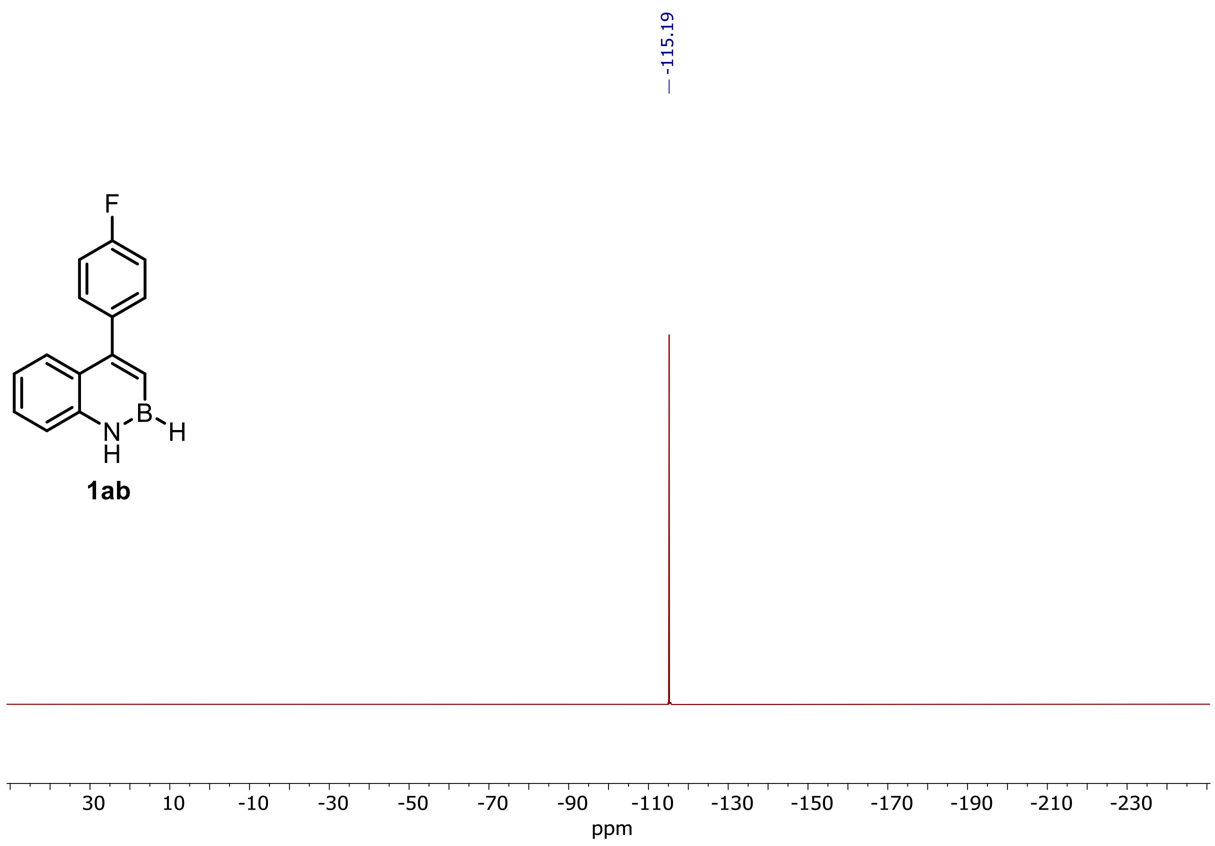

-115.19
F
N
H
B
H
1ab
30 10 -10 -30 -50 -70 -90 -110 -130 -150 -170 -190 -210 -230
ppm

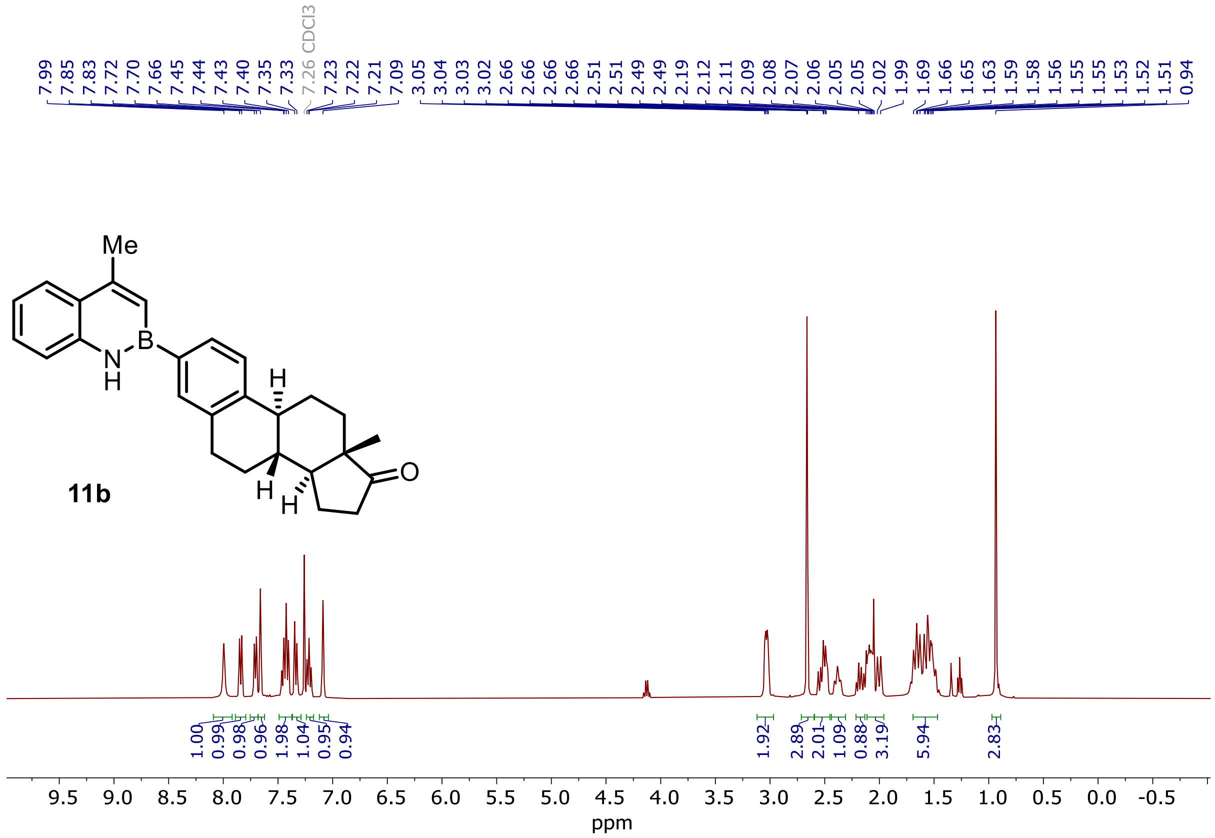

7.99 7.85 7.83 7.72 7.70 7.66 7.45 7.44 7.43 7.40 7.35 7.33 7.26 CDCl3 7.23 7.22 7.21 7.09 3.05 3.04 3.03 3.02 2.66 2.66 2.66 2.66 2.51 2.51 2.49 2.49 2.19 2.12 2.11 2.09 2.08 2.07 2.06 2.05 2.05 2.02 1.99 1.69 1.66 1.65 1.63 1.59 1.58 1.56 1.55 1.55 1.53 1.52 1.51 0.94
Me
N
H
B
H
H
H
O
11b
1.00 0.99 0.98 0.96 1.98 1.04 0.95 0.94
1.92 2.89 2.01 1.09 0.88 3.19 5.94 2.83
9.5 9.0 8.5 8.0 7.5 7.0 6.5 6.0 5.5 5.0 4.5 4.0 3.5 3.0 2.5 2.0 1.5 1.0 0.5 0.0 -0.5
ppm

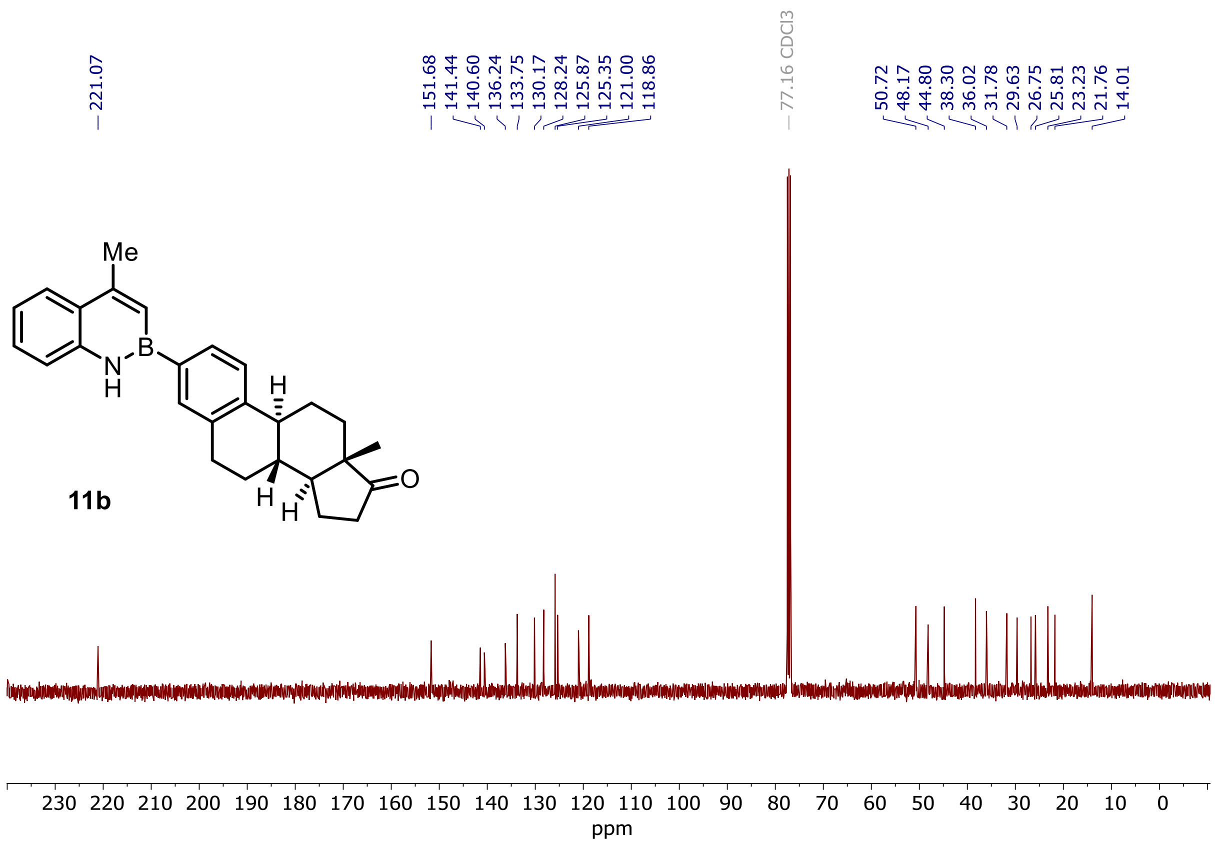
221.07
151.68
141.44
140.60
136.24
133.75
130.17
128.24
125.87
125.35
121.00
118.86
77.16 CDCl3
50.72
48.17
44.80
38.30
36.02
31.78
29.63
26.75
25.81
23.23
21.76
14.01
Me
B
N
H
H
H
H
O
11b
230 220 210 200 190 180 170 160 150 140 130 120 110 100 90 80 70 60 50 40 30 20 10 0
ppm

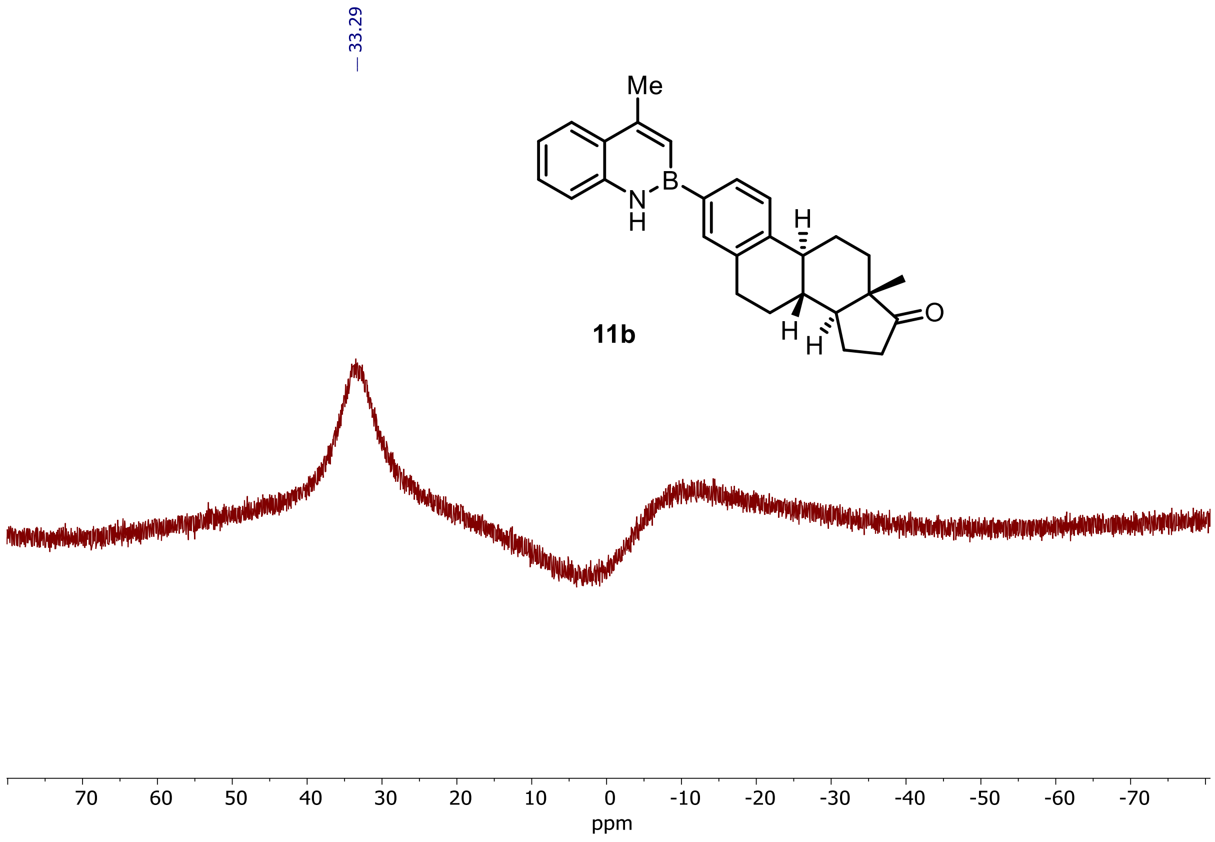
33.29
Me
B
N
H
H
H
H
O
11b
70 60 50 40 30 20 10 0 -10 -20 -30 -40 -50 -60 -70
ppm

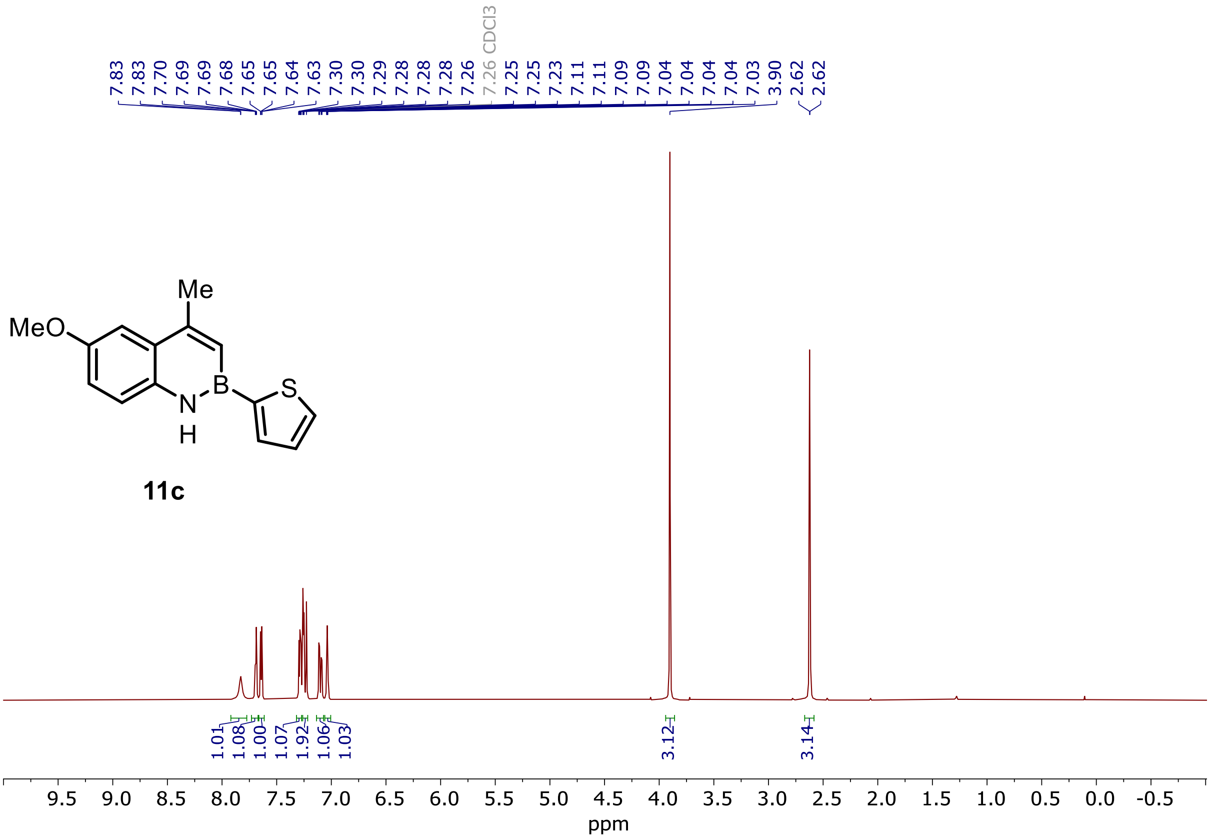
7.83
7.83
7.70
7.69
7.69
7.68
7.65
7.65
7.64
7.63
7.30
7.30
7.29
7.28
7.28
7.28
7.26
7.26 CDCl3
7.25
7.25
7.23
7.11
7.11
7.09
7.09
7.04
7.04
7.04
7.04
7.03
3.90
2.62
2.62
Me
MeO
N
H
B
S
11c
1.01
1.08
1.00
1.07
1.92
1.06
1.03
3.12
3.14
9.5 9.0 8.5 8.0 7.5 7.0 6.5 6.0 5.5 5.0 4.5 4.0 3.5 3.0 2.5 2.0 1.5 1.0 0.5 0.0 -0.5
ppm

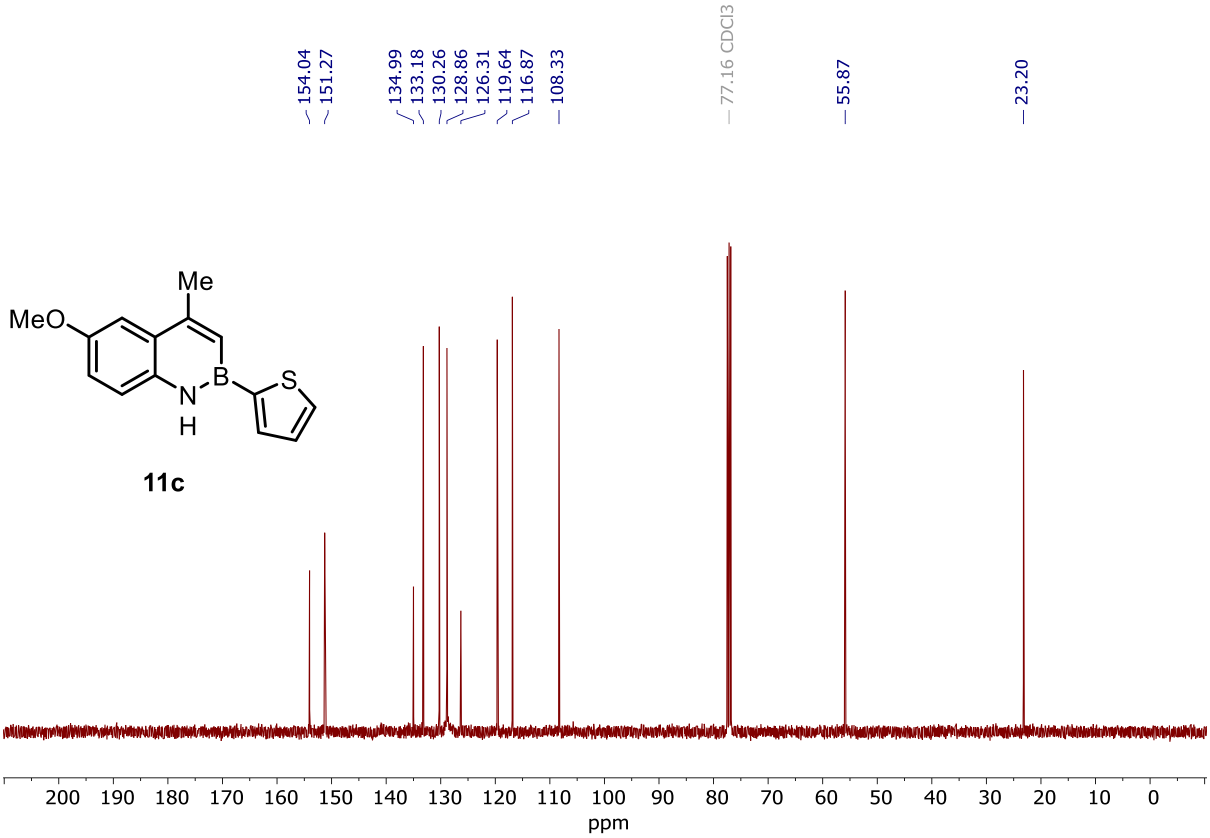
154.04
151.27
134.99
133.18
130.26
128.86
126.31
119.64
116.87
108.33
77.16 CDCl3
55.87
23.20
Me
MeO
N
H
B
S
11c
200 190 180 170 160 150 140 130 120 110 100 90 80 70 60 50 40 30 20 10 0
ppm

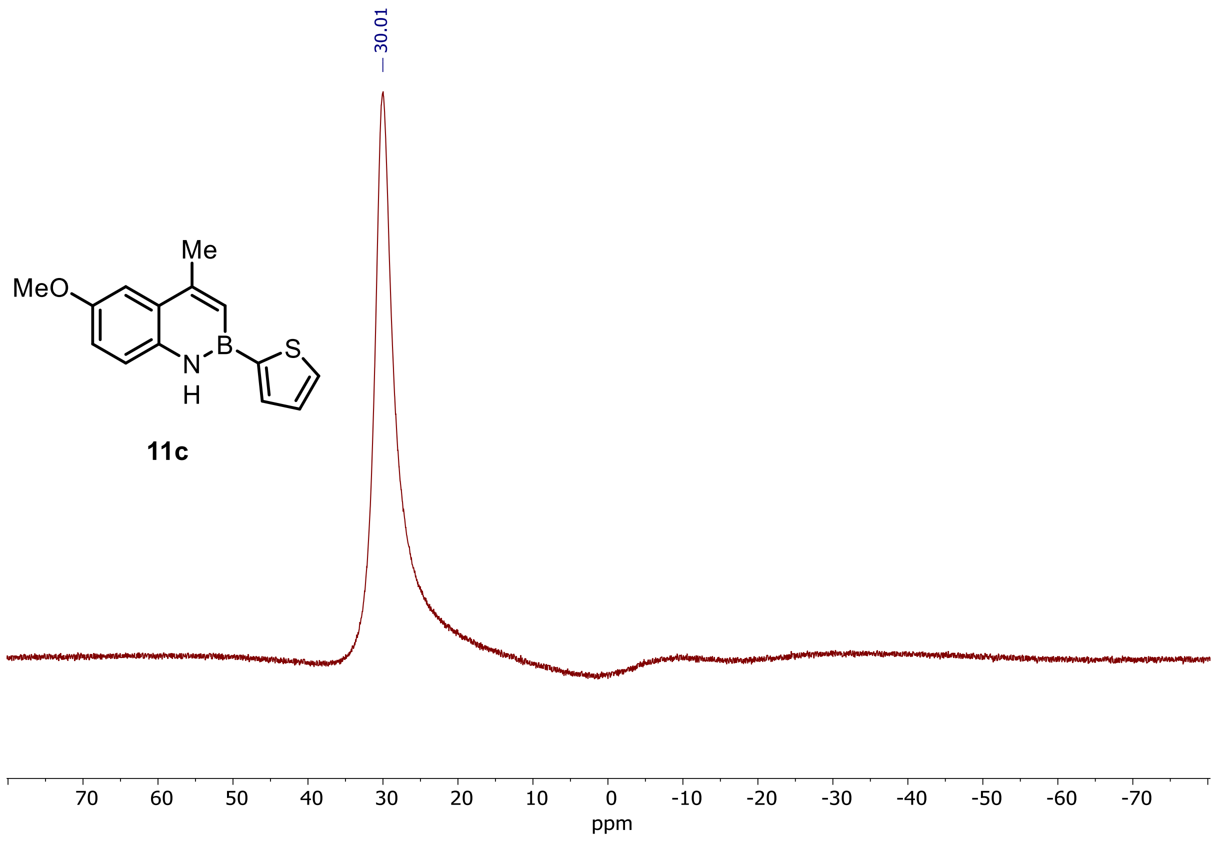
30.01
Me
MeO
N
B
S
H
11c
70 60 50 40 30 20 10 0 -10 -20 -30 -40 -50 -60 -70
ppm

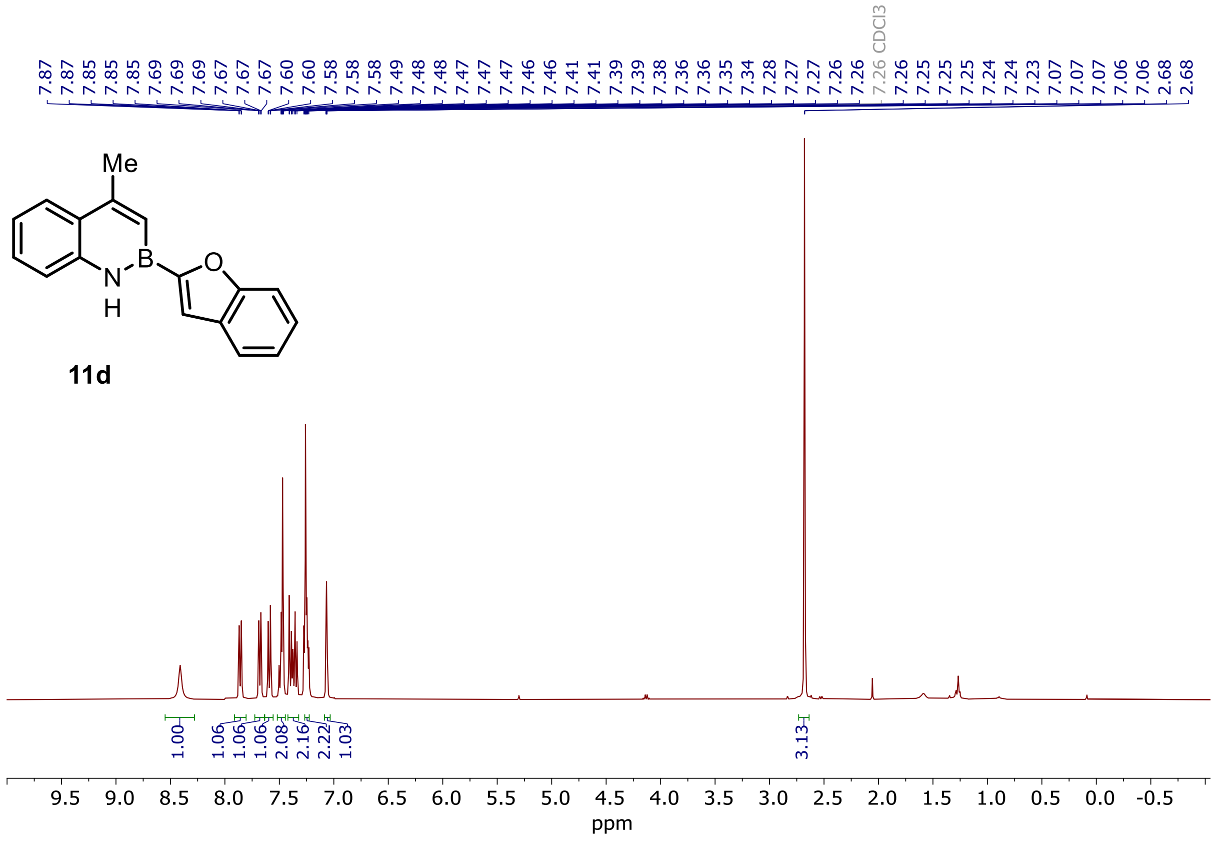
7.87 7.87 7.85 7.85 7.85 7.69 7.69 7.69 7.67 7.67 7.67 7.60 7.60 7.58 7.58 7.58 7.49 7.48 7.48 7.47 7.47 7.47 7.46 7.46 7.41 7.41 7.39 7.39 7.38 7.36 7.36 7.35 7.34 7.28 7.27 7.27 7.26 7.26 7.26 CDCl3 7.26 7.25 7.25 7.25 7.24 7.24 7.23 7.07 7.07 7.07 7.06 7.06 2.68 2.68
Me
N
B
O
H
11d
1.00 1.06 1.06 1.06 2.08 2.16 2.22 1.03 3.13
9.5 9.0 8.5 8.0 7.5 7.0 6.5 6.0 5.5 5.0 4.5 4.0 3.5 3.0 2.5 2.0 1.5 1.0 0.5 0.0 -0.5
ppm

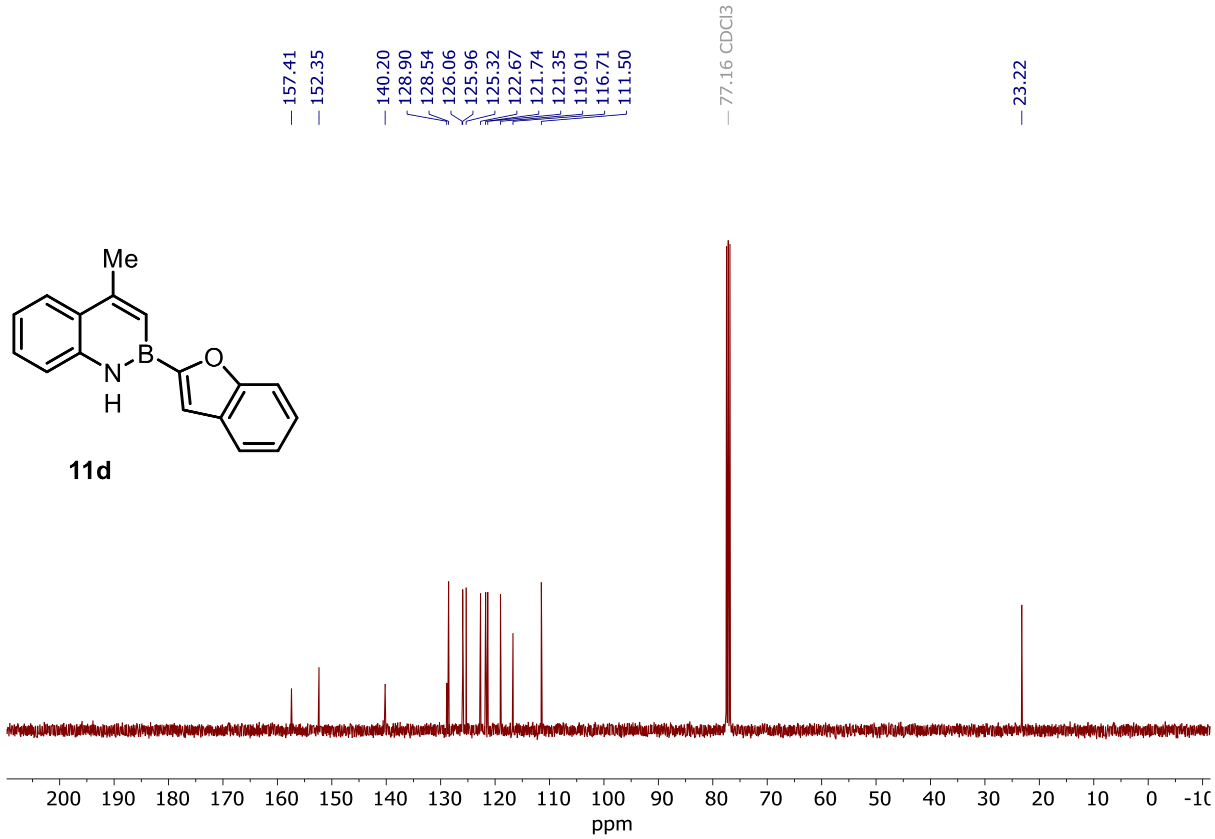

157.41
152.35
140.20
128.90
128.54
126.06
125.96
125.32
122.67
121.74
121.35
119.01
116.71
111.50
77.16 CDCl3
23.22
Me
N
H
B
O
11d
200 190 180 170 160 150 140 130 120 110 100 90 80 70 60 50 40 30 20 10 0 -10
ppm

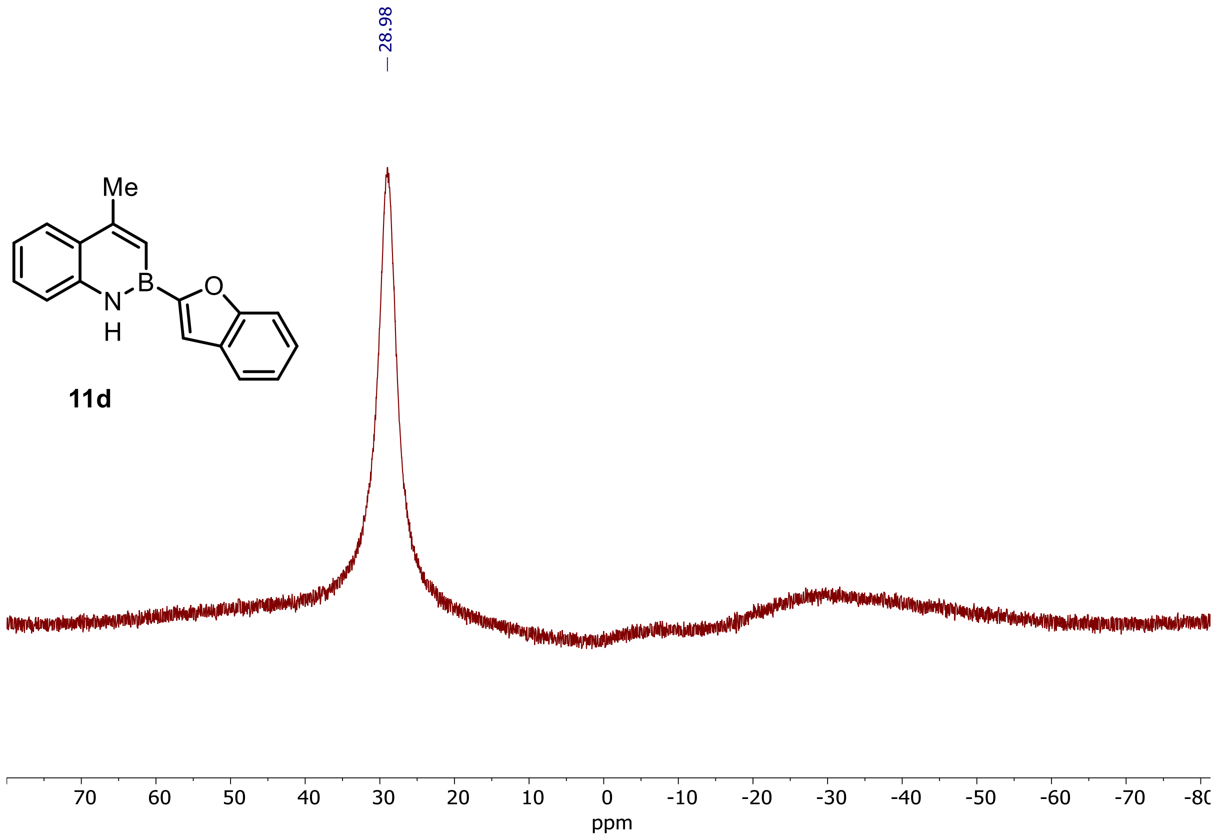

28.98
Me
N
H
B
O
11d
70 60 50 40 30 20 10 0 -10 -20 -30 -40 -50 -60 -70 -80
ppm

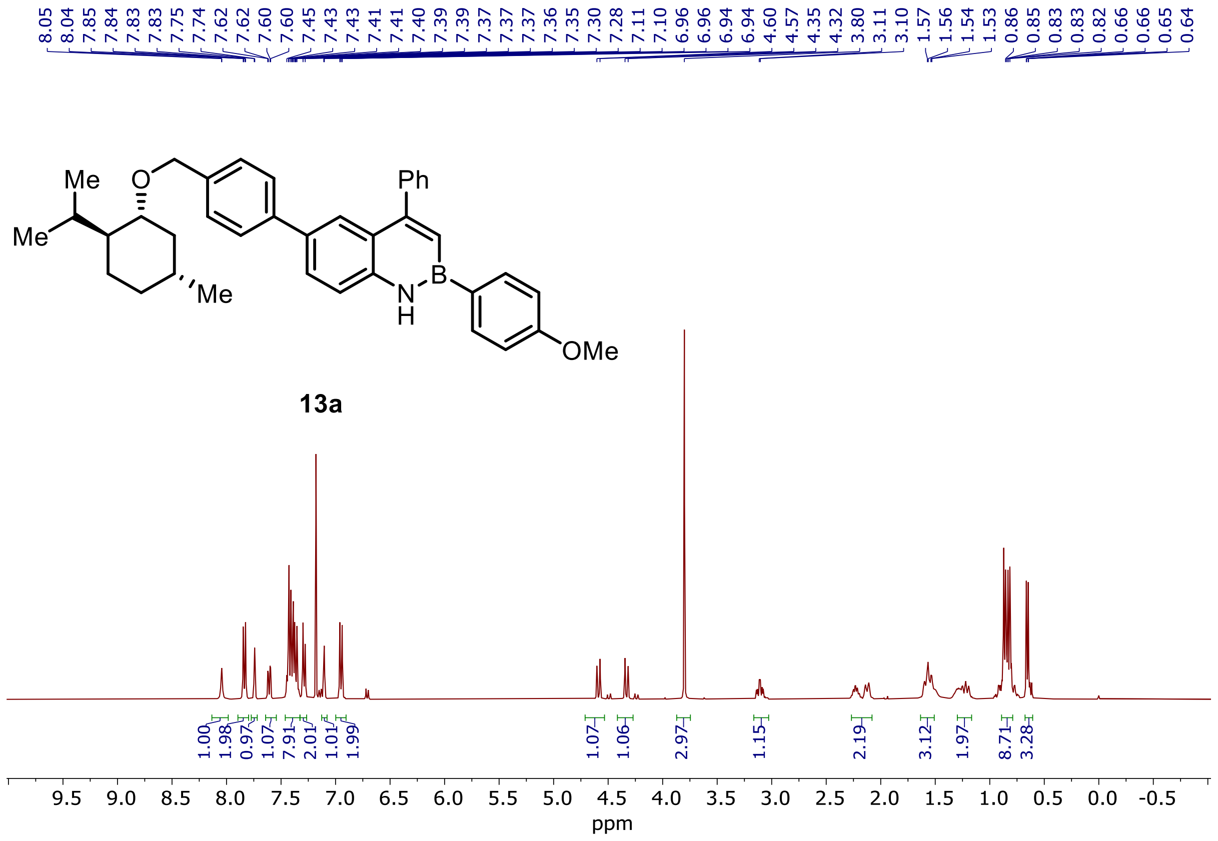
8.05 8.04 7.85 7.84 7.83 7.83 7.75 7.74 7.62 7.62 7.60 7.60 7.45 7.43 7.43 7.41 7.41 7.40 7.39 7.39 7.37 7.37 7.37 7.36 7.35 7.30 7.28 7.11 7.10 6.96 6.96 6.94 6.94 4.60 4.57 4.35 4.32 3.80 3.11 3.10 1.57 1.56 1.54 1.53 0.86 0.85 0.83 0.83 0.82 0.66 0.66 0.65 0.64
Me
O
Me
Me
Ph
N
B
H
OMe
13a
1.00 1.98 0.97 1.07 7.91 2.01 1.01 1.99 1.07 1.06 2.97 1.15 2.19 3.12 1.97 8.71 3.28
9.5 9.0 8.5 8.0 7.5 7.0 6.5 6.0 5.5 5.0 4.5 4.0 3.5 3.0 2.5 2.0 1.5 1.0 0.5 0.0 -0.5
ppm

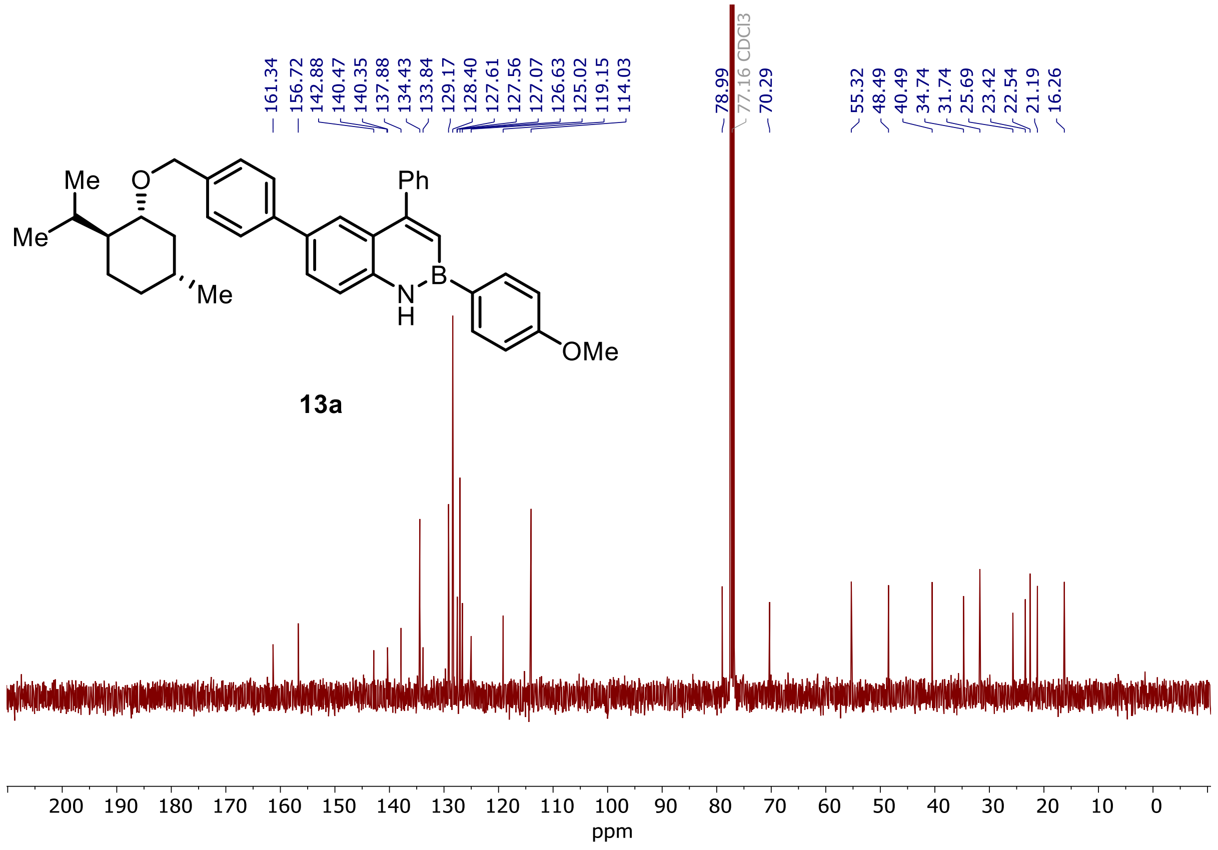
161.34 156.72 142.88 140.47 140.35 137.88 134.43 133.84 129.17 128.40 127.61 127.56 127.07 126.63 125.02 119.15 114.03
78.99 77.16 CDCl3 70.29
55.32 48.49 40.49 34.74 31.74 25.69 23.42 22.54 21.19 16.26
Me
O
Me
Me
Ph
N
B
H
OMe
13a
200 190 180 170 160 150 140 130 120 110 100 90 80 70 60 50 40 30 20 10 0
ppm

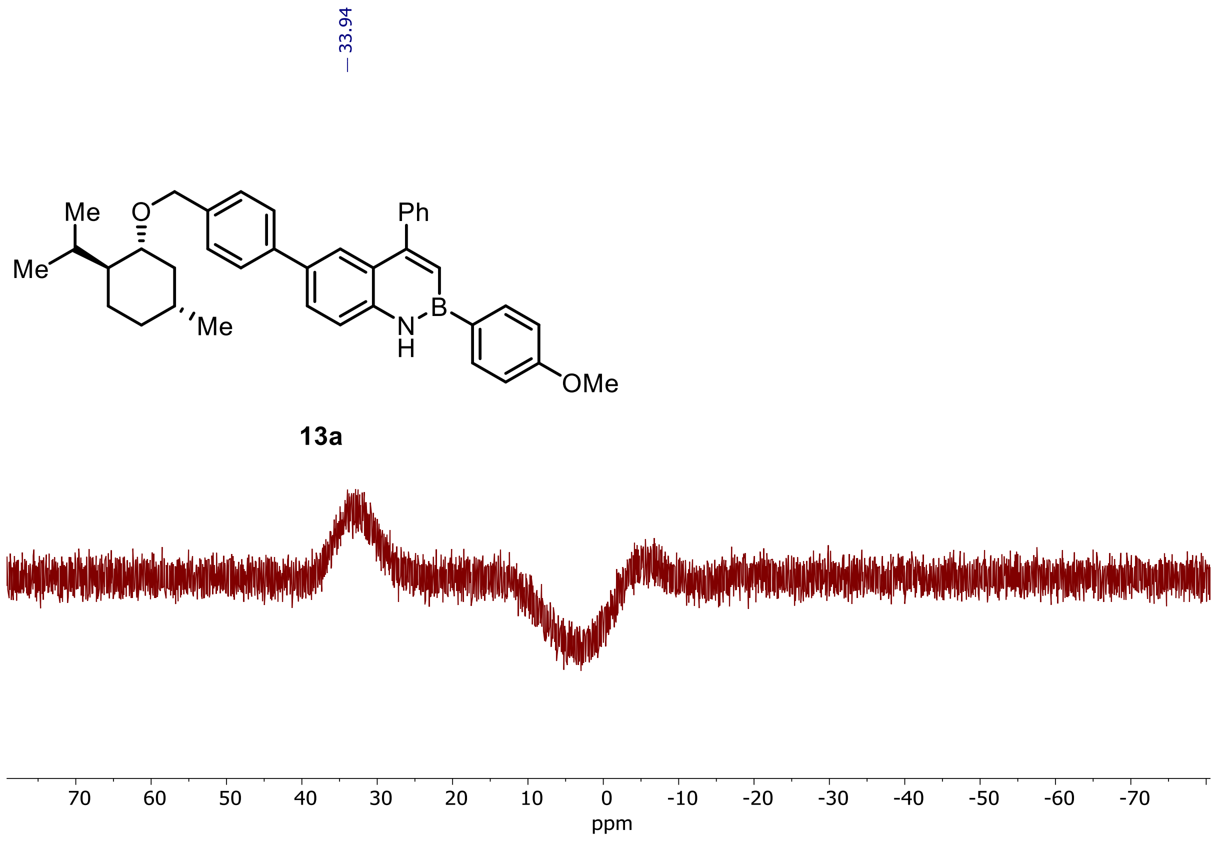

13a

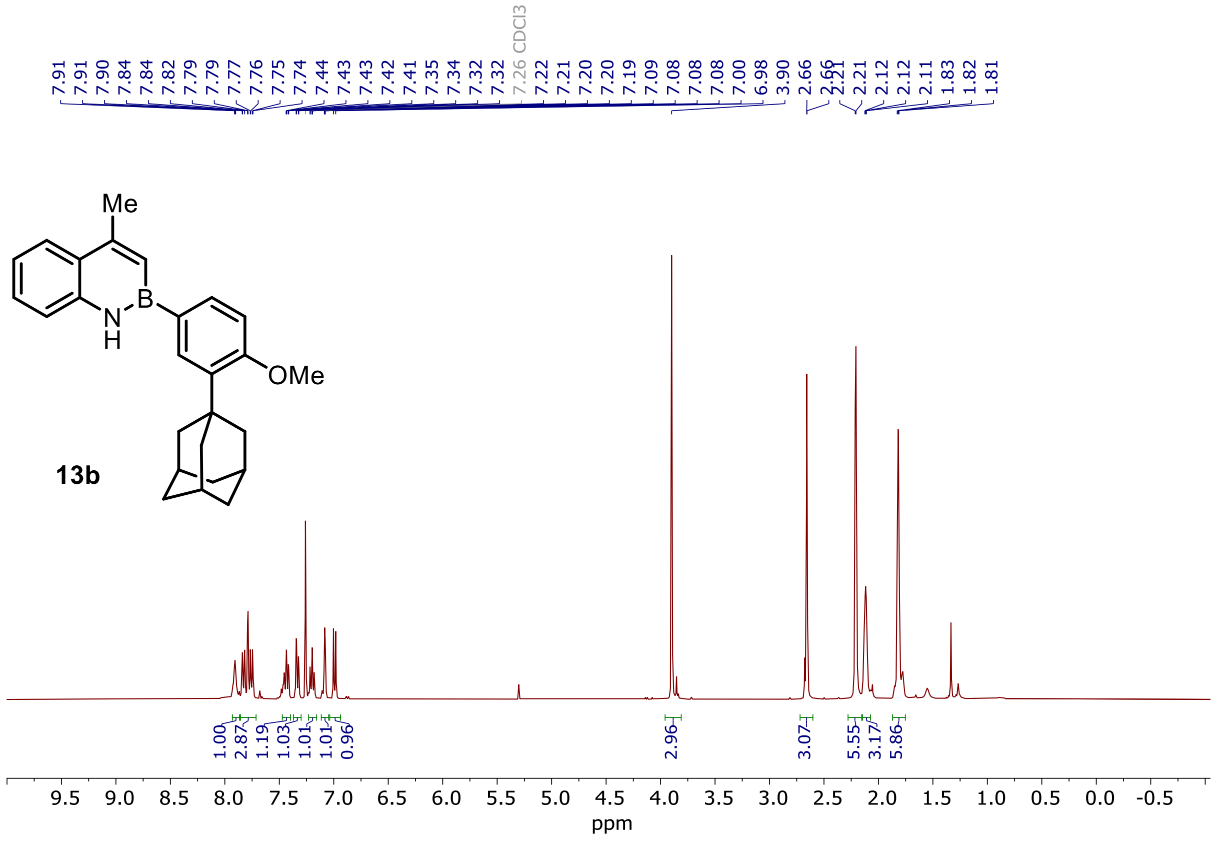

13b

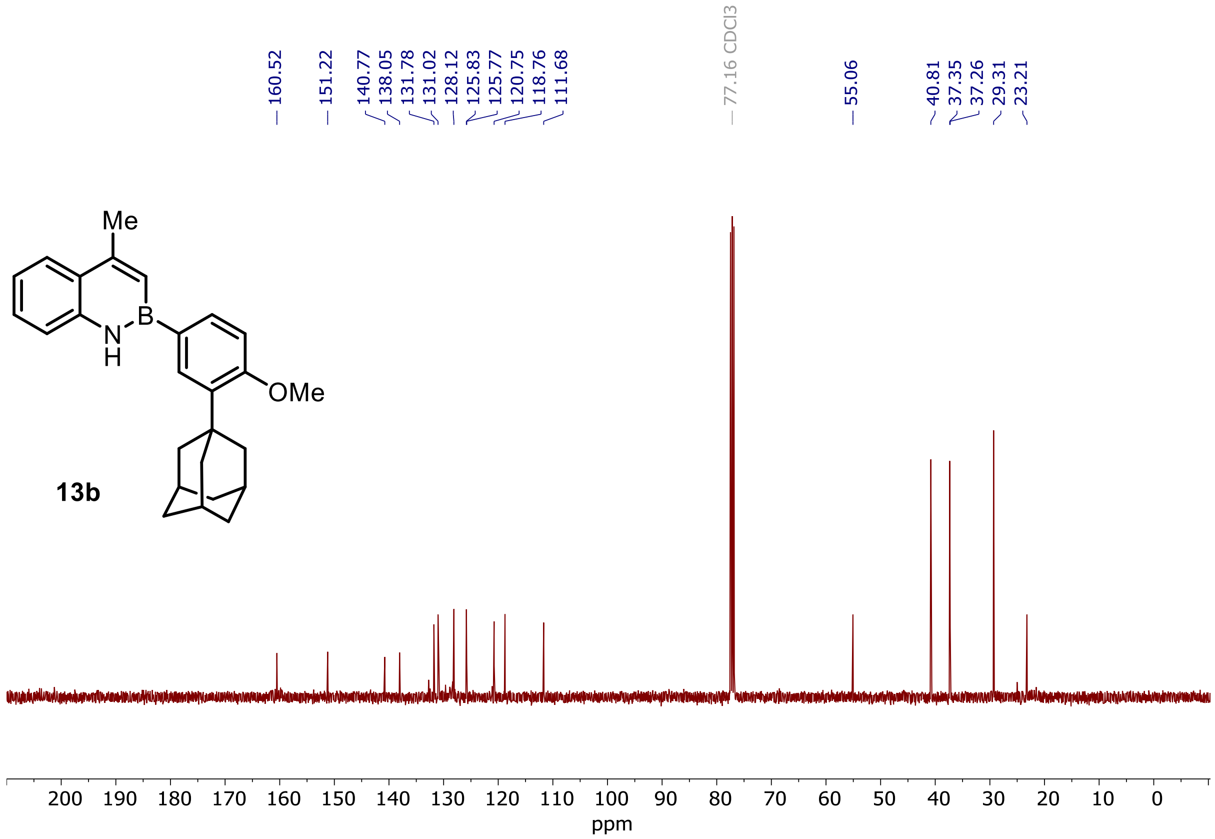

160.52
151.22
140.77
138.05
131.78
131.02
128.12
125.83
125.77
120.75
118.76
111.68
77.16 CDCl3
55.06
40.81
37.35
37.26
29.31
23.21
Me
B
N
H
OMe
13b
200 190 180 170 160 150 140 130 120 110 100 90 80 70 60 50 40 30 20 10 0
ppm

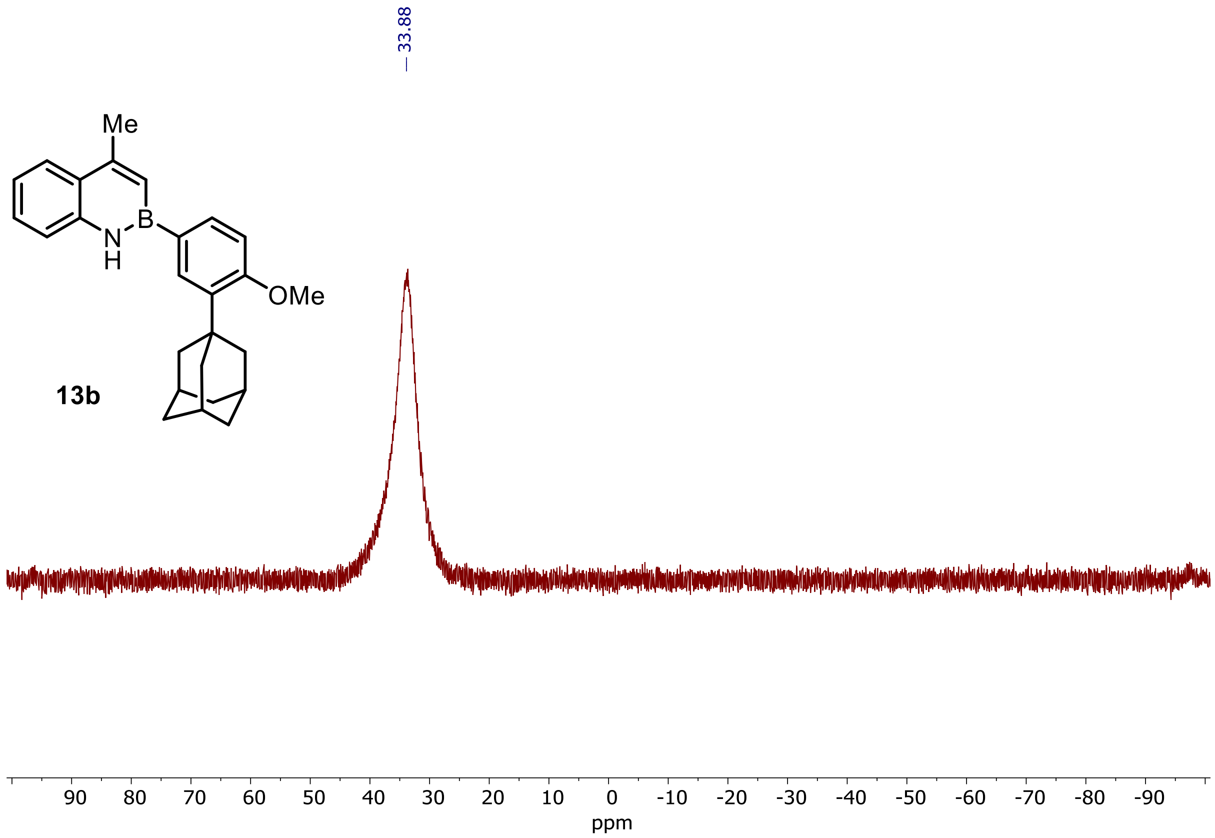

33.88
Me
B
N
H
OMe
13b
90 80 70 60 50 40 30 20 10 0 -10 -20 -30 -40 -50 -60 -70 -80 -90
ppm

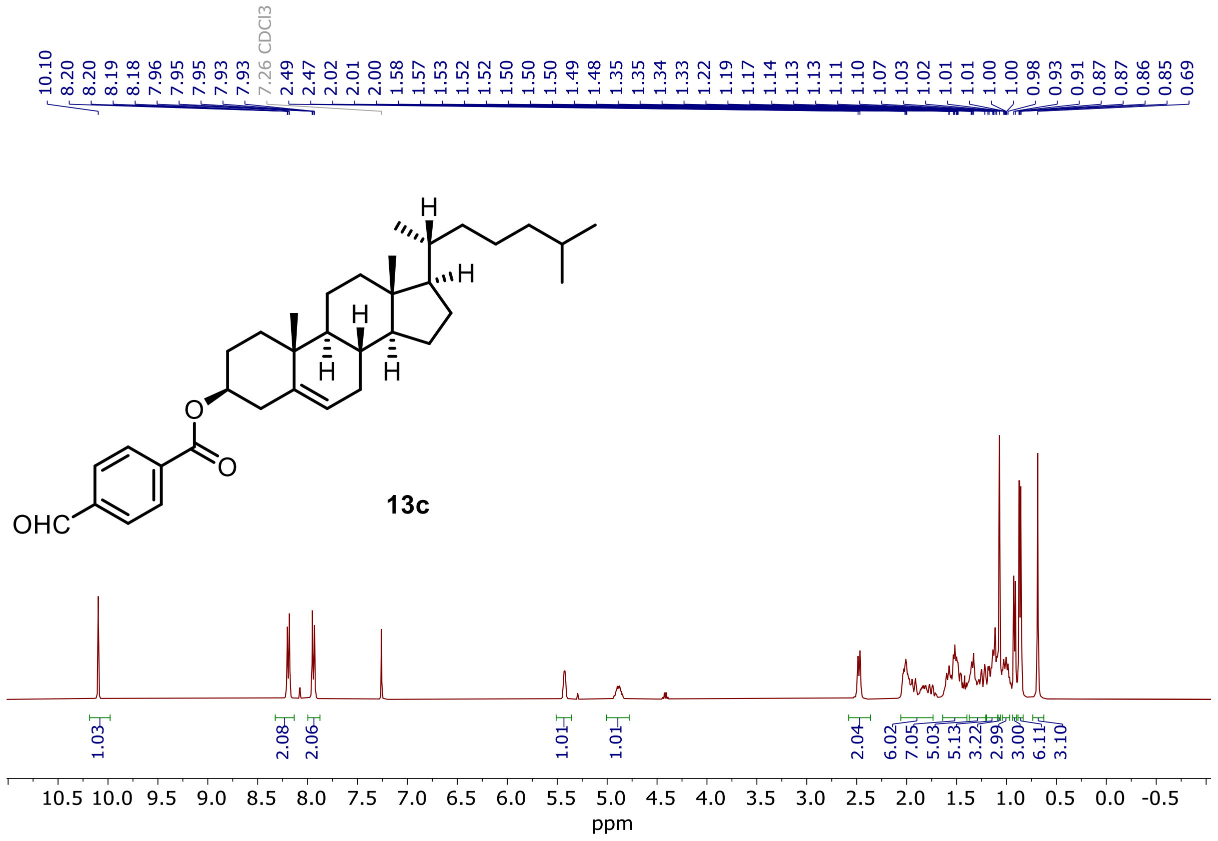
13c
OHC
ppm

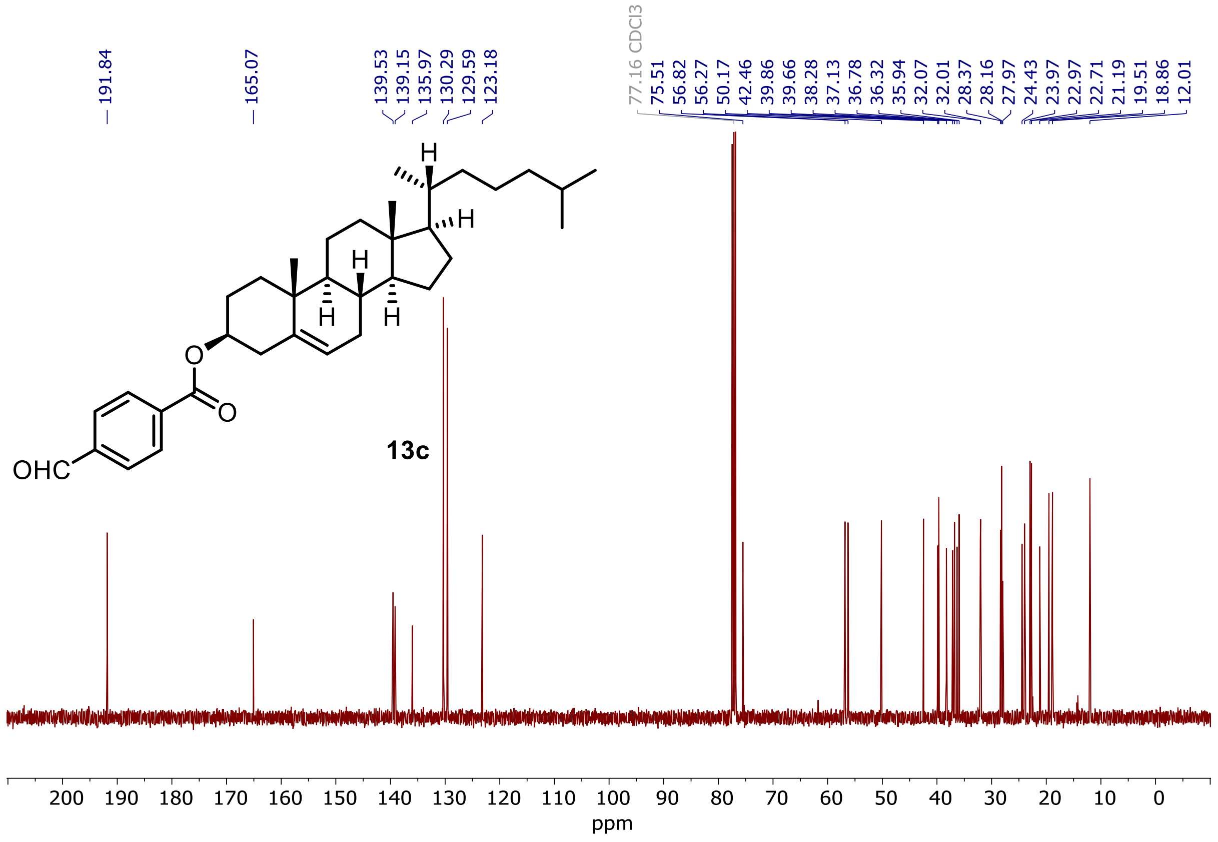
13c
OHC
ppm

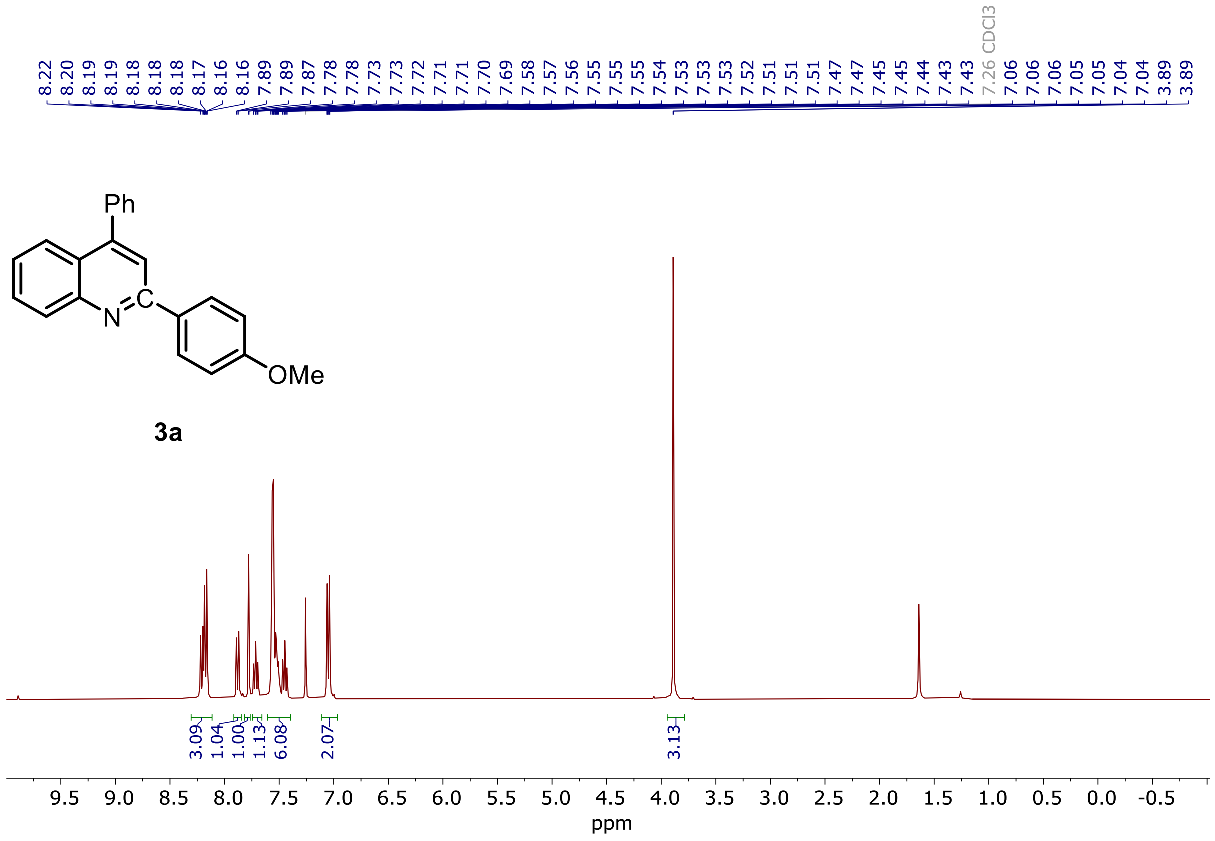
8.22
8.20
8.19
8.19
8.18
8.18
8.18
8.17
8.16
8.16
7.89
7.89
7.87
7.78
7.78
7.73
7.73
7.72
7.71
7.71
7.70
7.69
7.58
7.57
7.56
7.55
7.55
7.55
7.54
7.53
7.53
7.53
7.52
7.51
7.51
7.51
7.47
7.47
7.45
7.45
7.44
7.43
7.43
7.26 CDCl3
7.06
7.06
7.06
7.05
7.05
7.04
7.04
3.89
3.89
Ph
N
C
OMe
3a
3.09
1.04
1.00
1.13
6.08
2.07
3.13
ppm

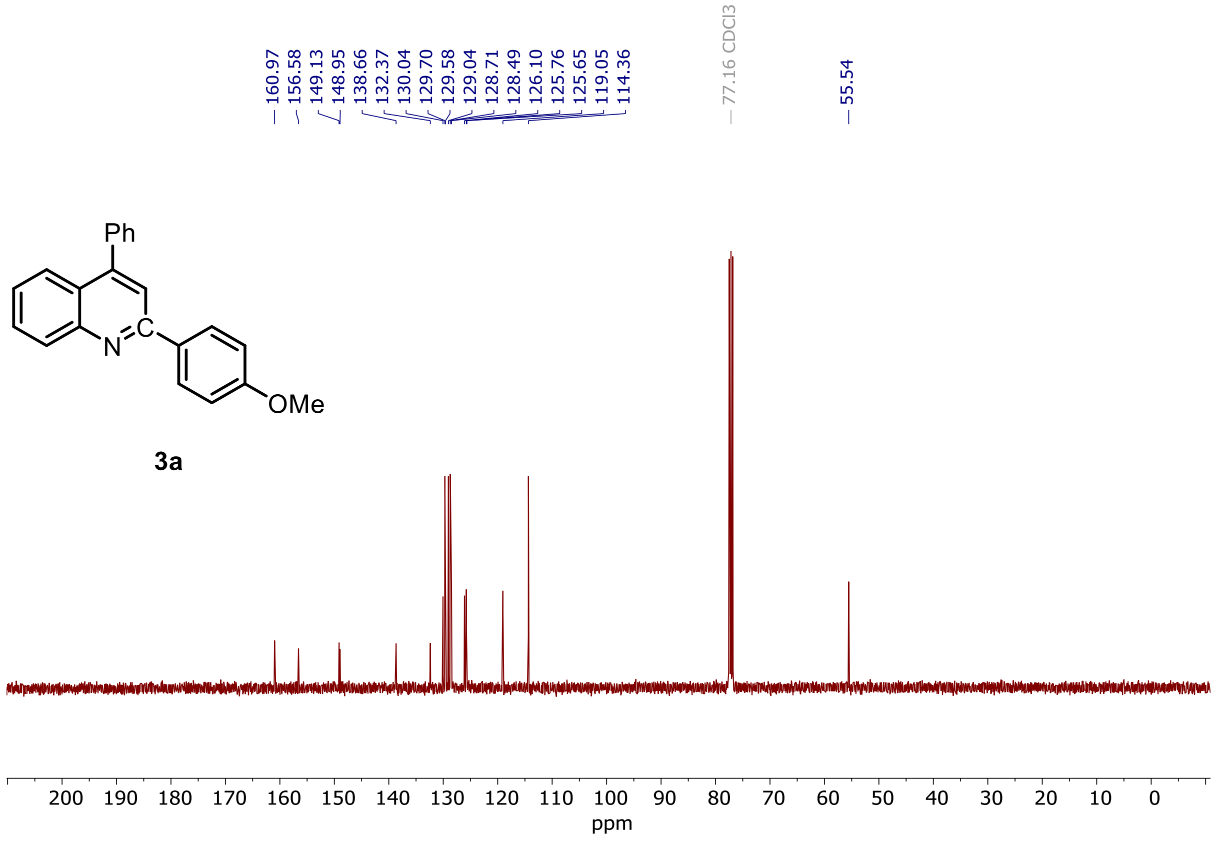
160.97
156.58
149.13
148.95
138.66
132.37
130.04
129.70
129.58
129.04
128.71
128.49
126.10
125.76
125.65
119.05
114.36
77.16 CDCl3
55.54
Ph
N
C
OMe
3a
ppm

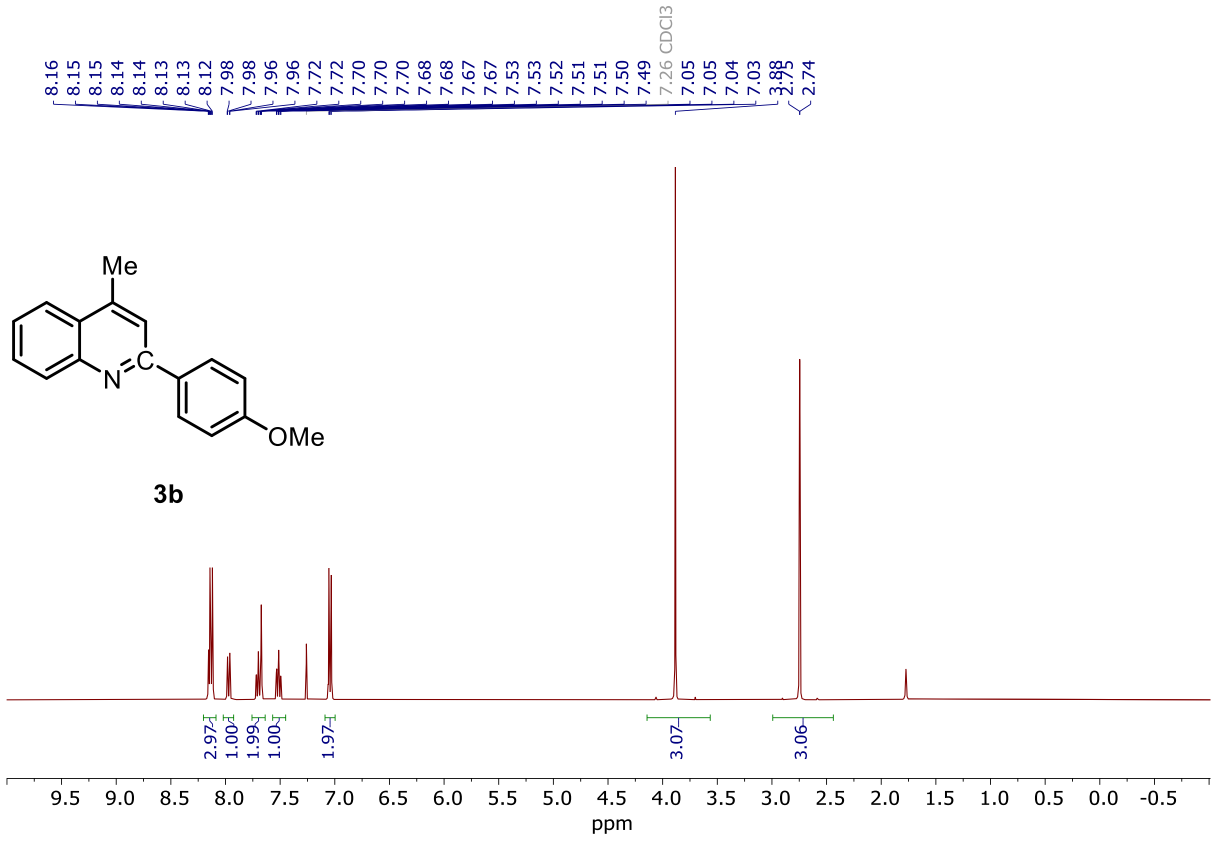

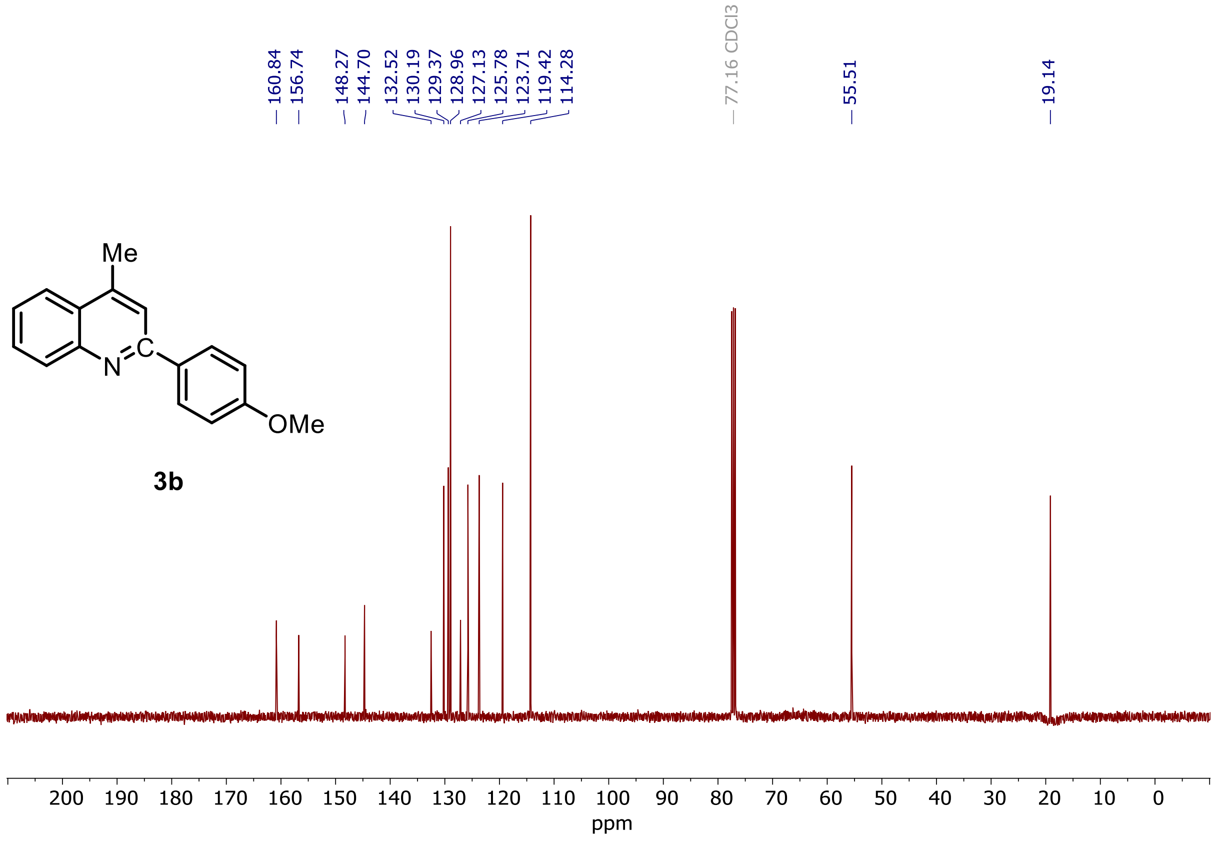

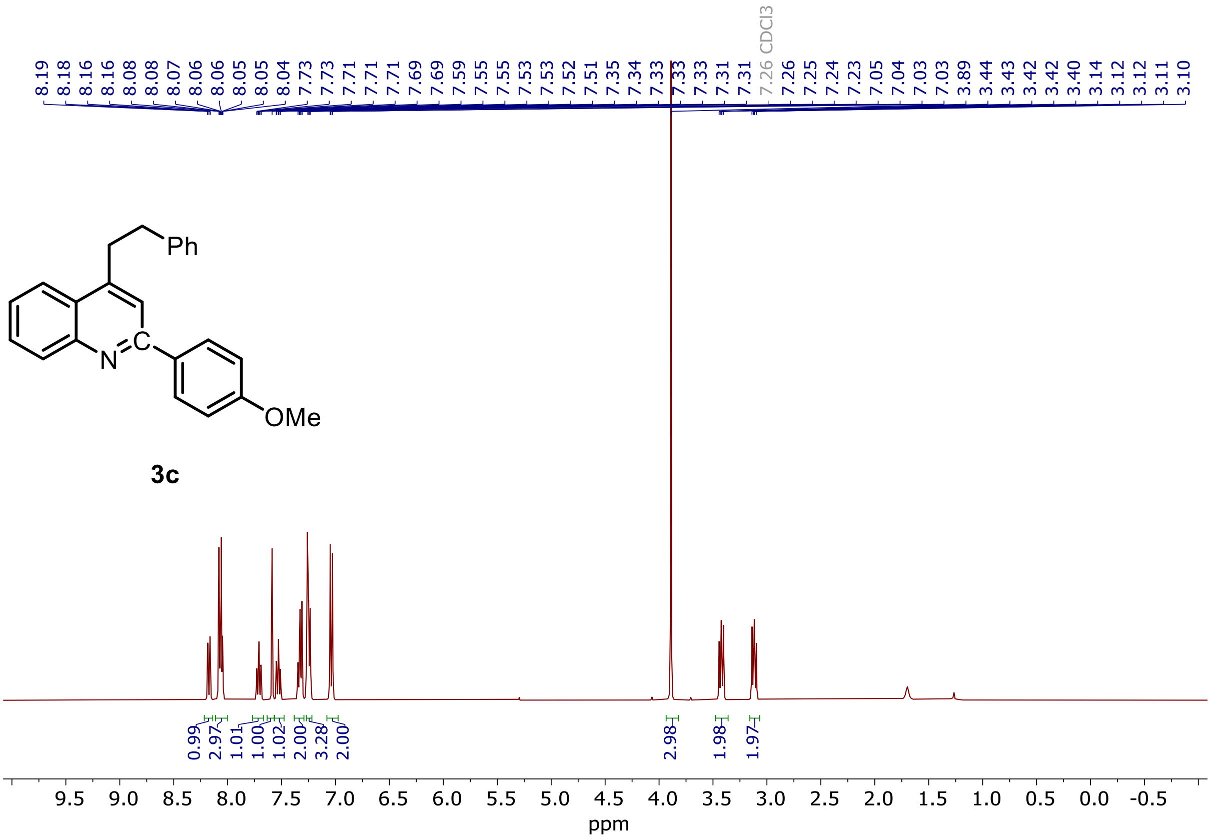
8.19 8.18 8.16 8.16 8.08 8.08 8.07 8.06 8.06 8.05 8.05 8.04 7.73 7.73 7.71 7.71 7.71 7.69 7.69 7.59 7.55 7.55 7.53 7.53 7.52 7.51 7.35 7.34 7.33 7.33 7.33 7.31 7.31 7.26 CDCl3 7.26 7.25 7.24 7.23 7.05 7.04 7.03 7.03 3.89 3.44 3.43 3.42 3.42 3.40 3.14 3.12 3.11 3.10
Ph
N
C
OMe
3c
0.99 2.97 1.01 1.00 1.02 2.00 3.28 2.00 2.98 1.98 1.97
9.5 9.0 8.5 8.0 7.5 7.0 6.5 6.0 5.5 5.0 4.5 4.0 3.5 3.0 2.5 2.0 1.5 1.0 0.5 0.0 -0.5
ppm

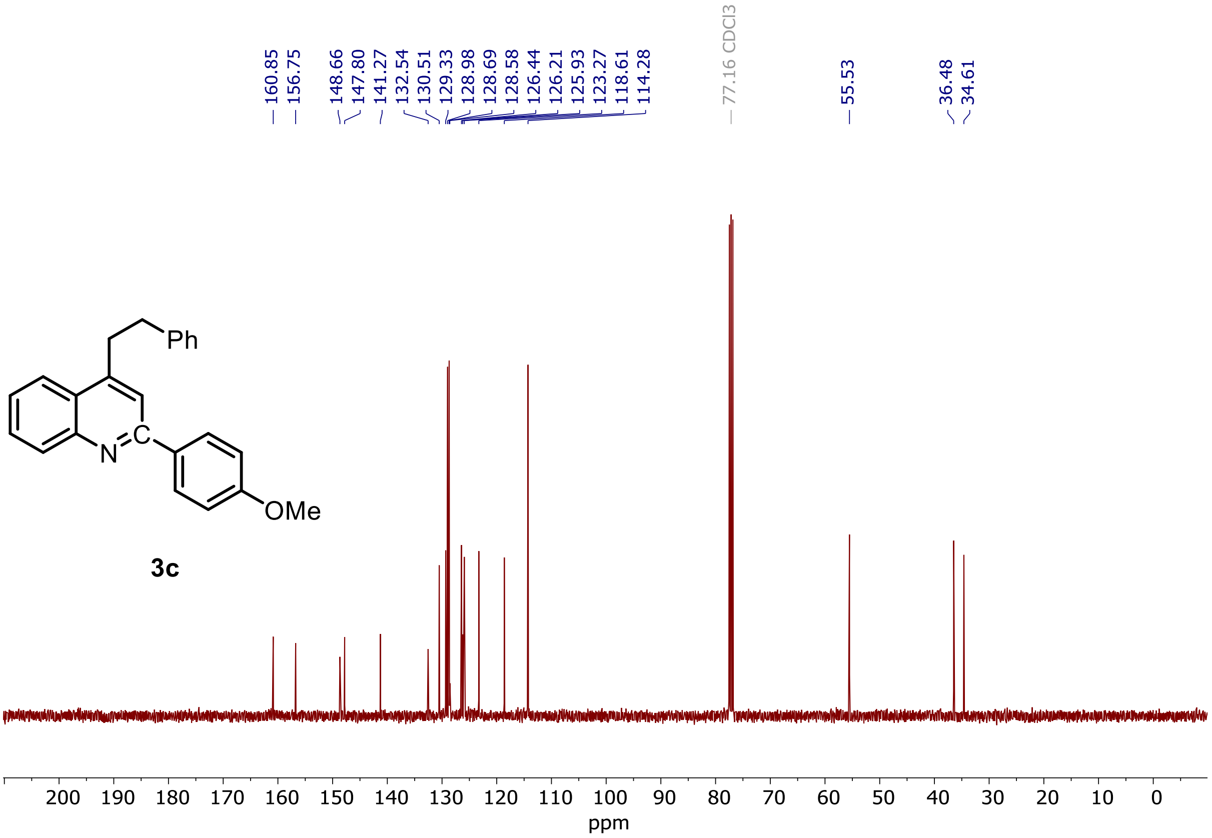
160.85 156.75 148.66 147.80 141.27 132.54 130.51 129.33 128.98 128.69 128.58 126.44 126.21 125.93 123.27 118.61 114.28 77.16 CDCl3 55.53 36.48 34.61
Ph
N
C
OMe
3c
200 190 180 170 160 150 140 130 120 110 100 90 80 70 60 50 40 30 20 10 0
ppm

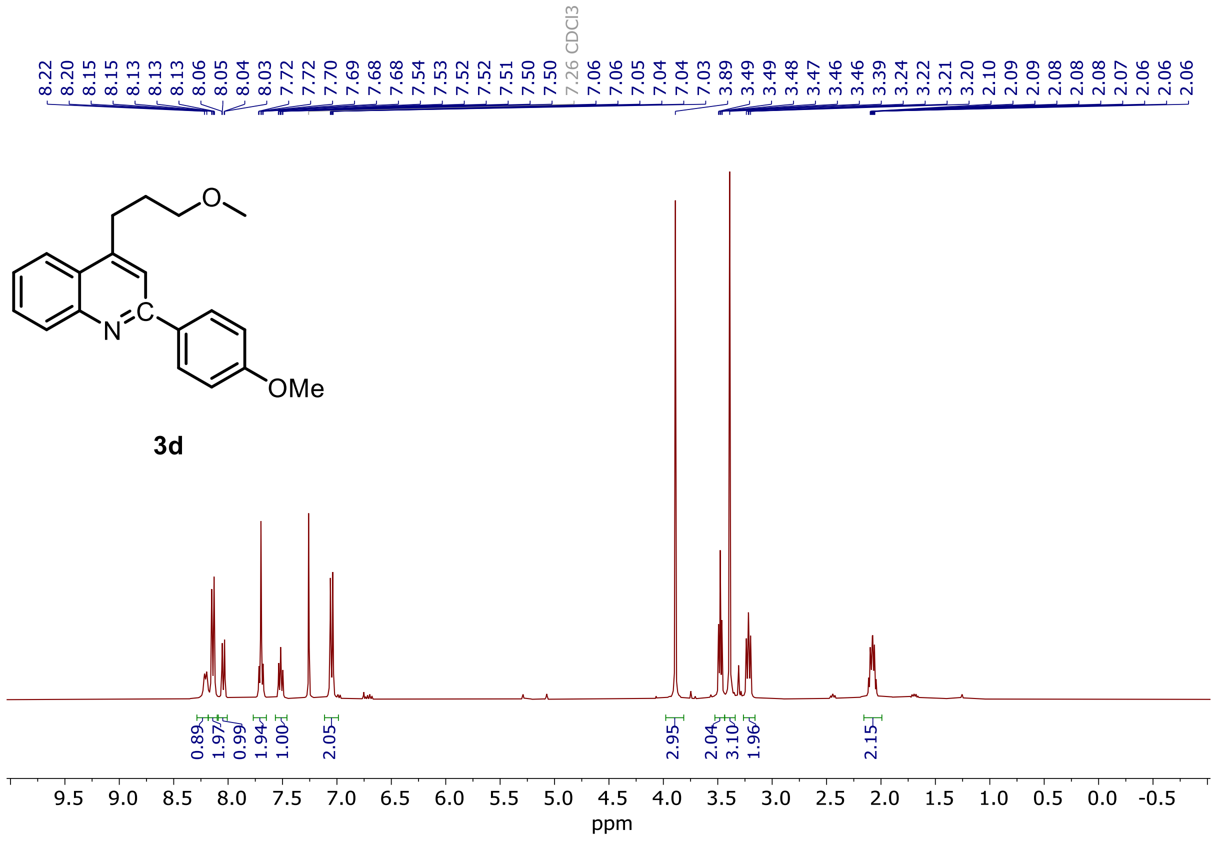
8.22
8.20
8.15
8.15
8.13
8.13
8.13
8.06
8.05
8.04
8.03
7.72
7.72
7.70
7.69
7.68
7.68
7.54
7.53
7.52
7.52
7.51
7.50
7.50
7.26 CDCl3
7.06
7.06
7.05
7.04
7.04
7.03
3.89
3.49
3.49
3.48
3.47
3.46
3.46
3.39
3.24
3.22
3.21
3.20
2.10
2.09
2.09
2.08
2.08
2.08
2.07
2.06
2.06
2.06
N
C
O
OMe
3d
0.89
1.97
0.99
1.94
1.00
2.05
2.95
2.04
3.10
1.96
2.15
9.5 9.0 8.5 8.0 7.5 7.0 6.5 6.0 5.5 5.0 4.5 4.0 3.5 3.0 2.5 2.0 1.5 1.0 0.5 0.0 -0.5
ppm

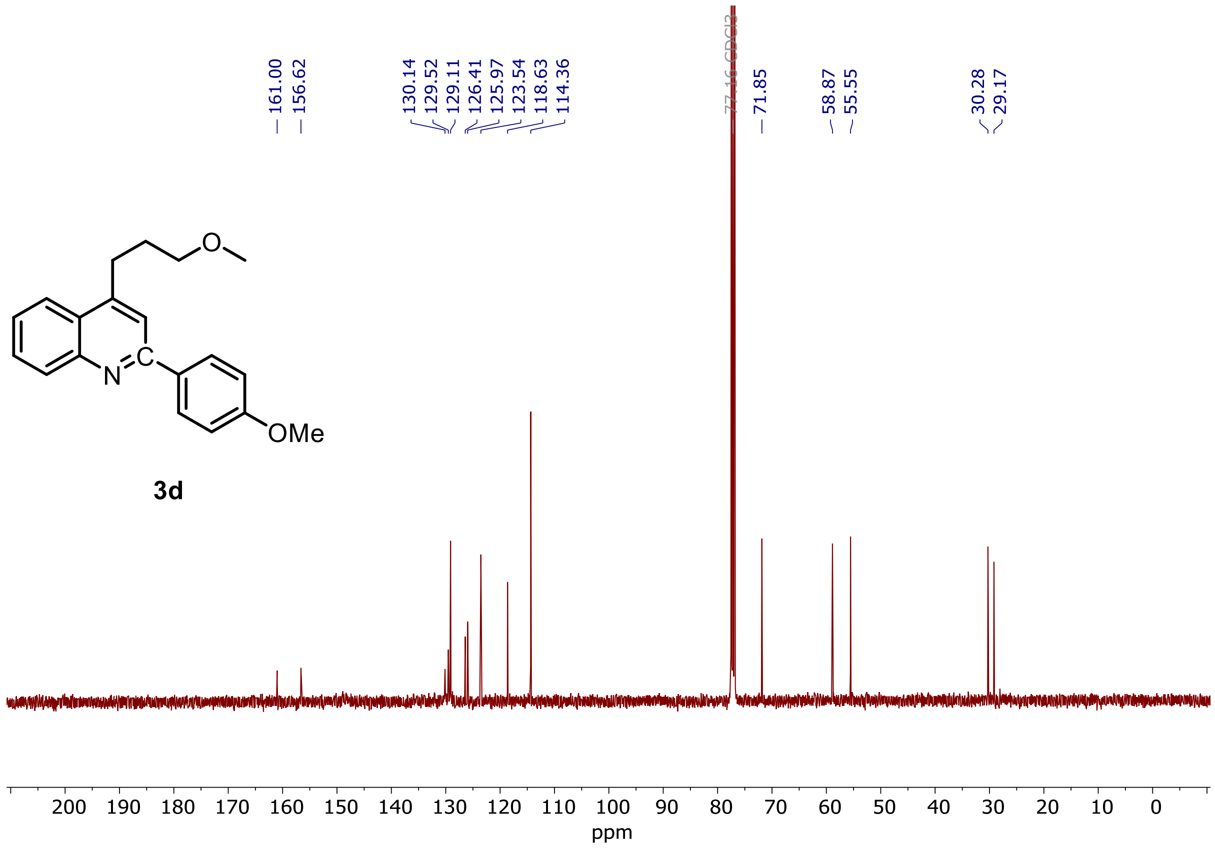
161.00
156.62
130.14
129.52
129.11
126.41
125.97
123.54
118.63
114.36
77.16 CDCl3
71.85
58.87
55.55
30.28
29.17
N
C
O
OMe
3d
200 190 180 170 160 150 140 130 120 110 100 90 80 70 60 50 40 30 20 10 0
ppm

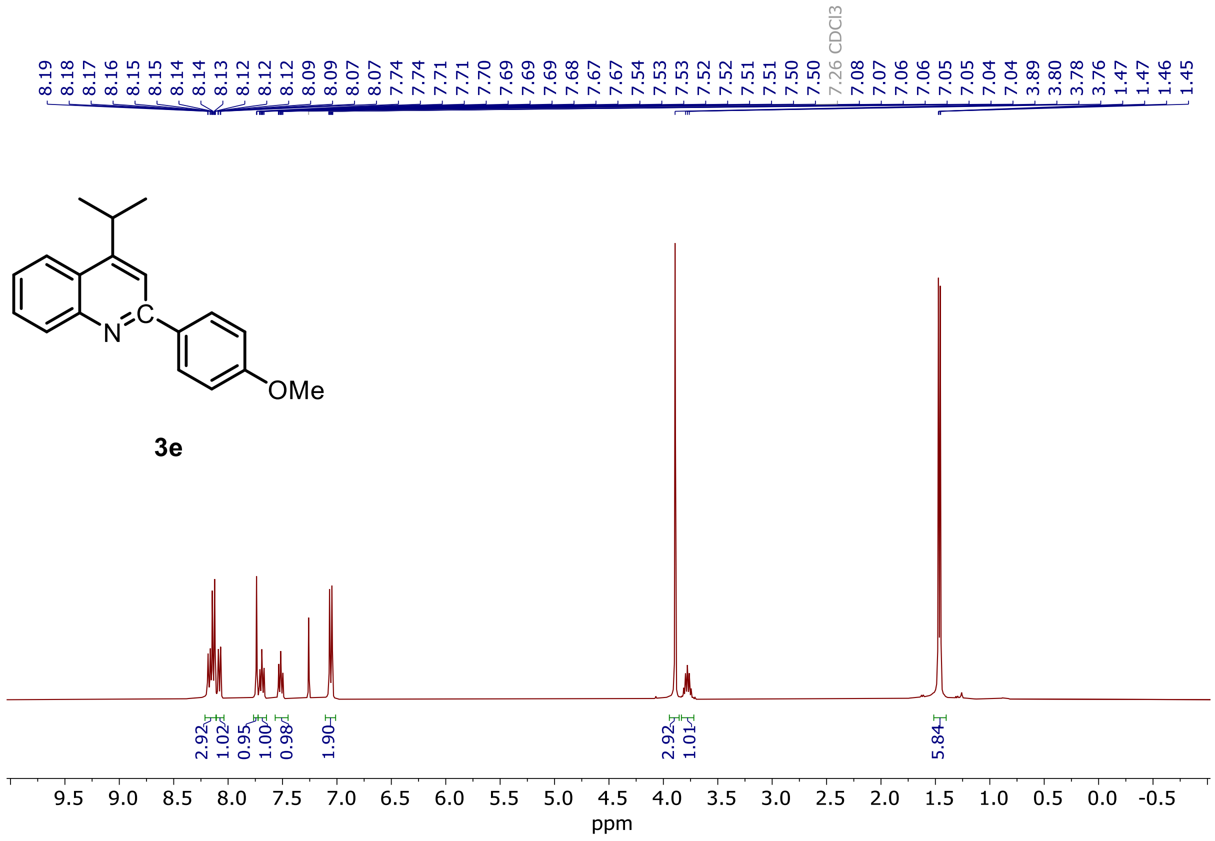
8.19 8.18 8.17 8.16 8.15 8.15 8.14 8.14 8.13 8.12 8.12 8.12 8.09 8.09 8.07 8.07 7.74 7.74 7.71 7.71 7.70 7.69 7.69 7.69 7.68 7.67 7.67 7.54 7.53 7.53 7.52 7.52 7.51 7.51 7.50 7.50 7.26 CDCl3 7.08 7.07 7.06 7.06 7.05 7.05 7.04 7.04 3.89 3.80 3.78 3.76 1.47 1.47 1.46 1.45
OMe
3e
2.92 1.02 0.95 1.00 0.98 1.90 2.92 1.01 5.84
9.5 9.0 8.5 8.0 7.5 7.0 6.5 6.0 5.5 5.0 4.5 4.0 3.5 3.0 2.5 2.0 1.5 1.0 0.5 0.0 -0.5
ppm

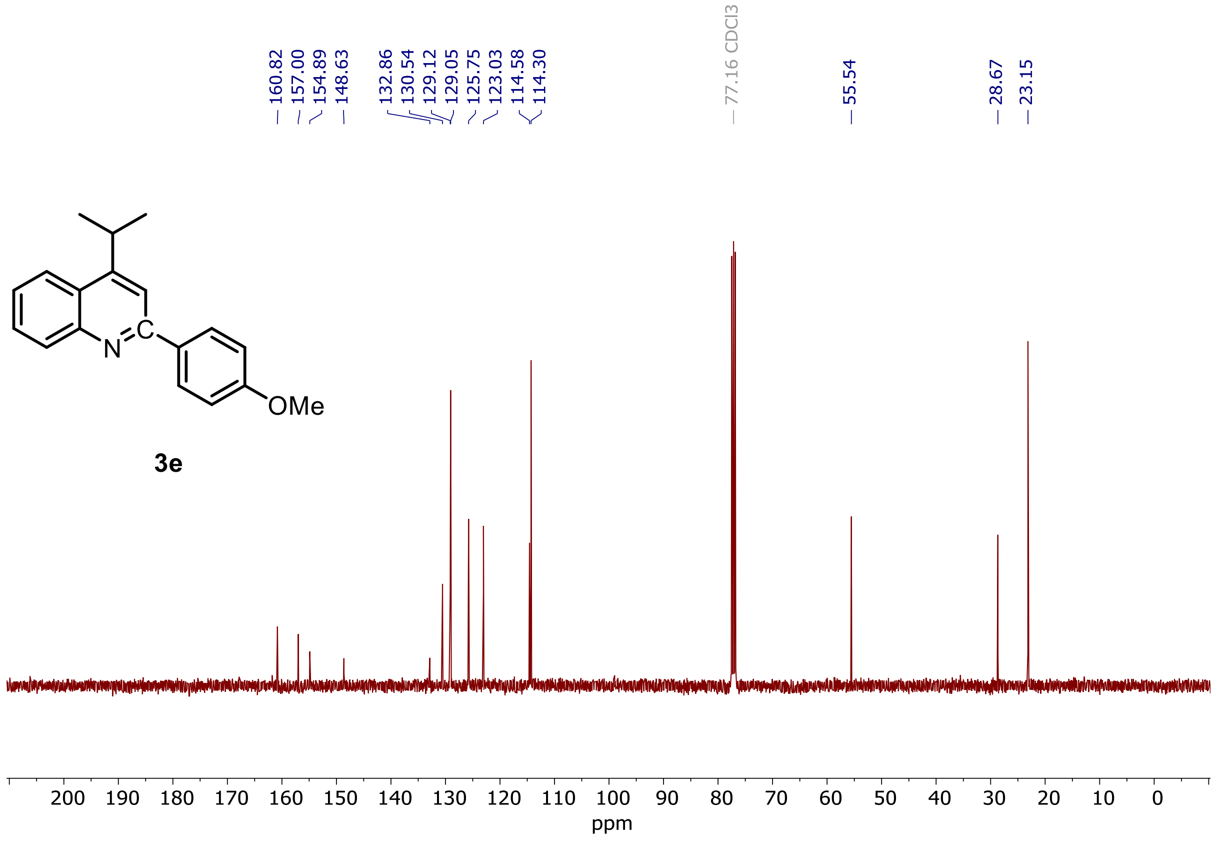
160.82 157.00 154.89 148.63 132.86 130.54 129.12 129.05 125.75 123.03 114.58 114.30 77.16 CDCl3 55.54 28.67 23.15
OMe
3e
200 190 180 170 160 150 140 130 120 110 100 90 80 70 60 50 40 30 20 10 0
ppm

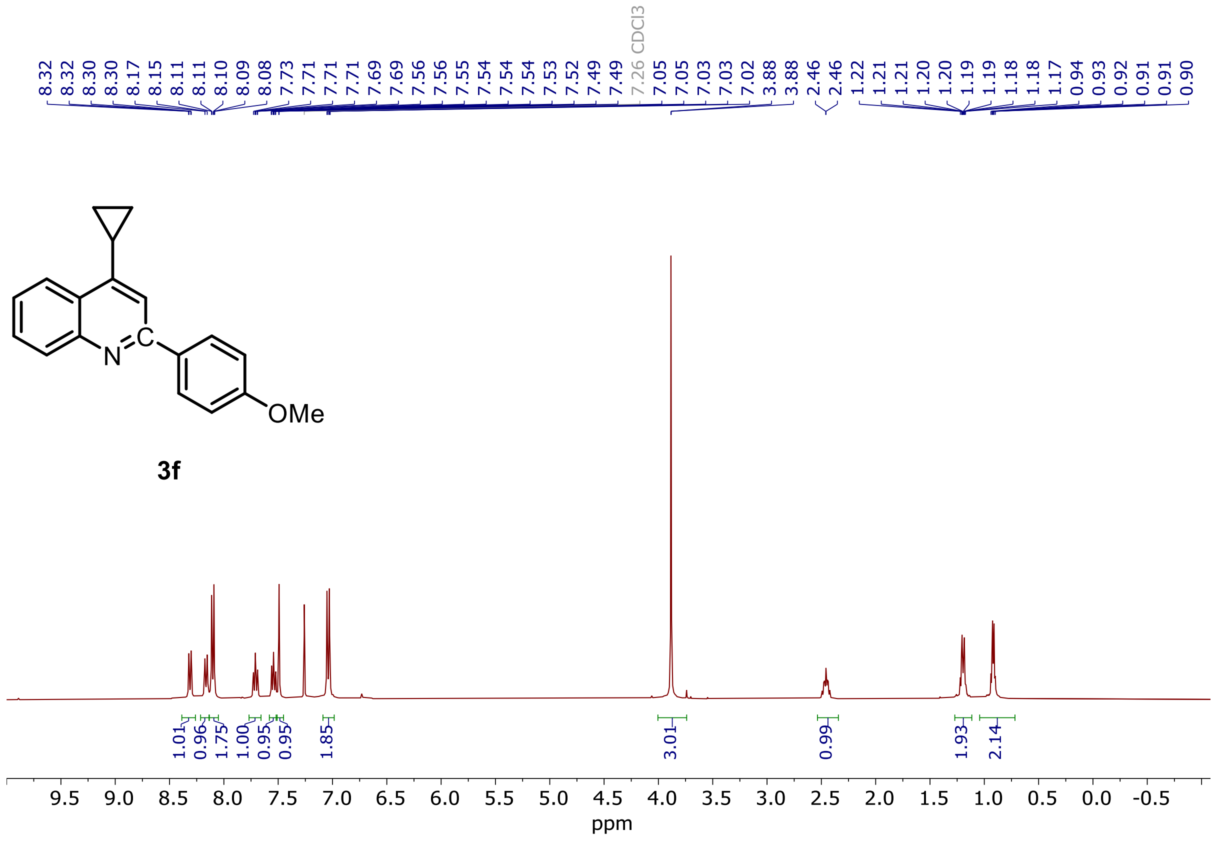
8.32 8.32 8.30 8.30 8.17 8.15 8.11 8.11 8.10 8.09 8.08 7.73 7.71 7.71 7.71 7.69 7.69 7.56 7.56 7.55 7.54 7.54 7.54 7.53 7.52 7.49 7.49 7.26 CDCl3 7.05 7.05 7.03 7.03 7.02 3.88 3.88 2.46 2.46 1.22 1.21 1.21 1.20 1.20 1.19 1.19 1.18 1.18 1.17 0.94 0.93 0.92 0.91 0.91 0.90
N
C
OMe
3f
1.01 0.96 1.75 1.00 0.95 0.95 1.85 3.01 0.99 1.93 2.14
9.5 9.0 8.5 8.0 7.5 7.0 6.5 6.0 5.5 5.0 4.5 4.0 3.5 3.0 2.5 2.0 1.5 1.0 0.5 0.0 -0.5
ppm

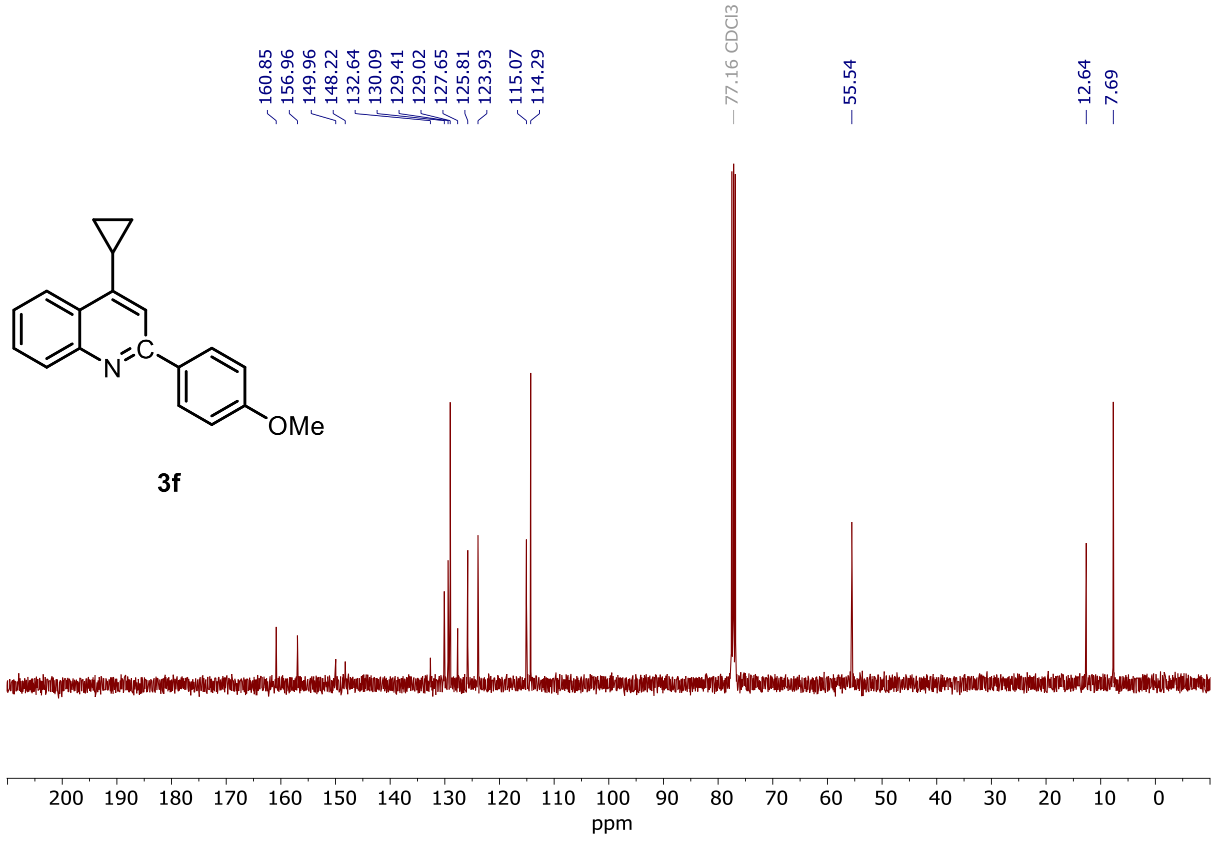
160.85 156.96 149.96 148.22 132.64 130.09 129.41 129.02 127.65 125.81 123.93 115.07 114.29
77.16 CDCl3
55.54
12.64 7.69
N
C
OMe
3f
200 190 180 170 160 150 140 130 120 110 100 90 80 70 60 50 40 30 20 10 0
ppm

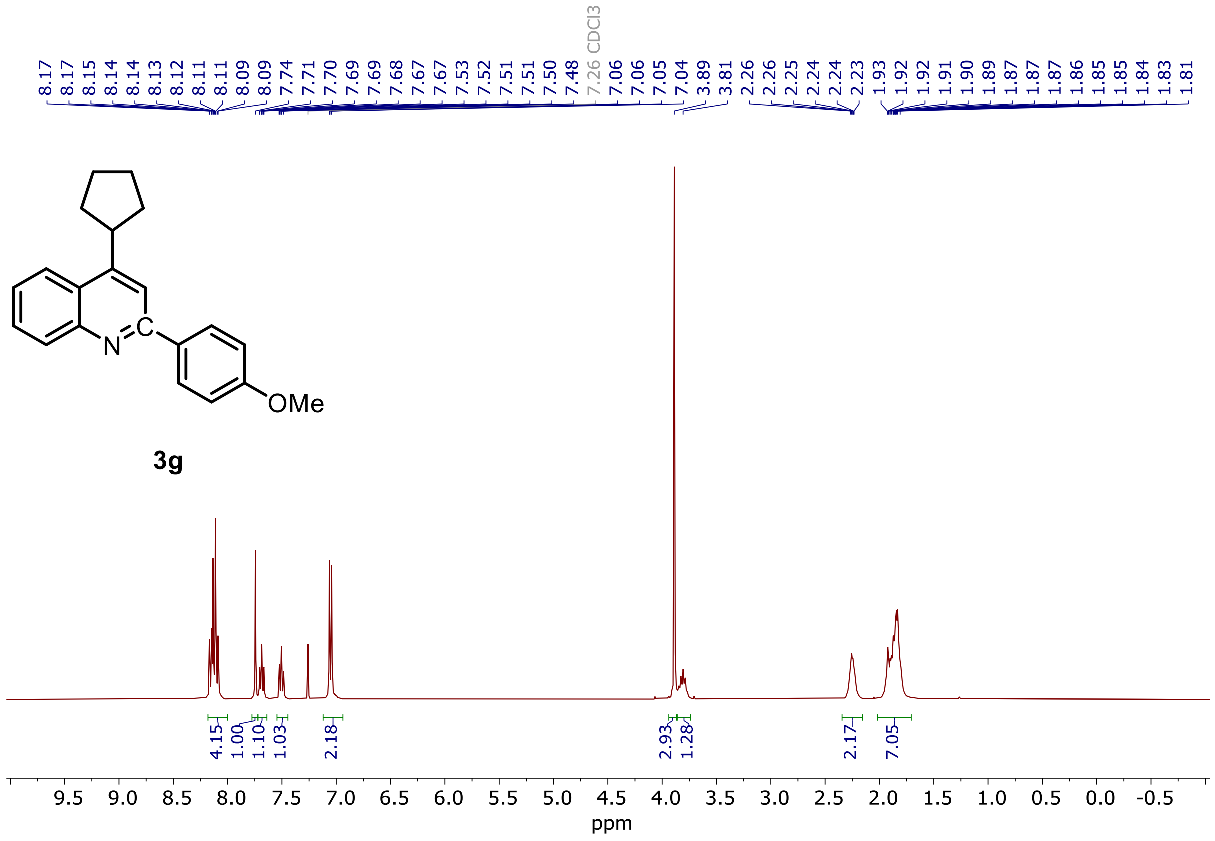
8.17 8.17 8.15 8.14 8.14 8.13 8.12 8.11 8.11 8.09 8.09 7.74 7.71 7.70 7.69 7.69 7.68 7.67 7.67 7.53 7.52 7.51 7.51 7.50 7.48 7.26 CDCl3 7.06 7.06 7.05 7.04 3.89 3.81 2.26 2.26 2.25 2.24 2.24 2.23 1.93 1.92 1.92 1.91 1.90 1.89 1.87 1.87 1.87 1.86 1.85 1.85 1.84 1.83 1.81
N
C
OMe
3g
4.15 1.00 1.10 1.03 2.18 2.93 1.28 2.17 7.05
9.5 9.0 8.5 8.0 7.5 7.0 6.5 6.0 5.5 5.0 4.5 4.0 3.5 3.0 2.5 2.0 1.5 1.0 0.5 0.0 -0.5
ppm

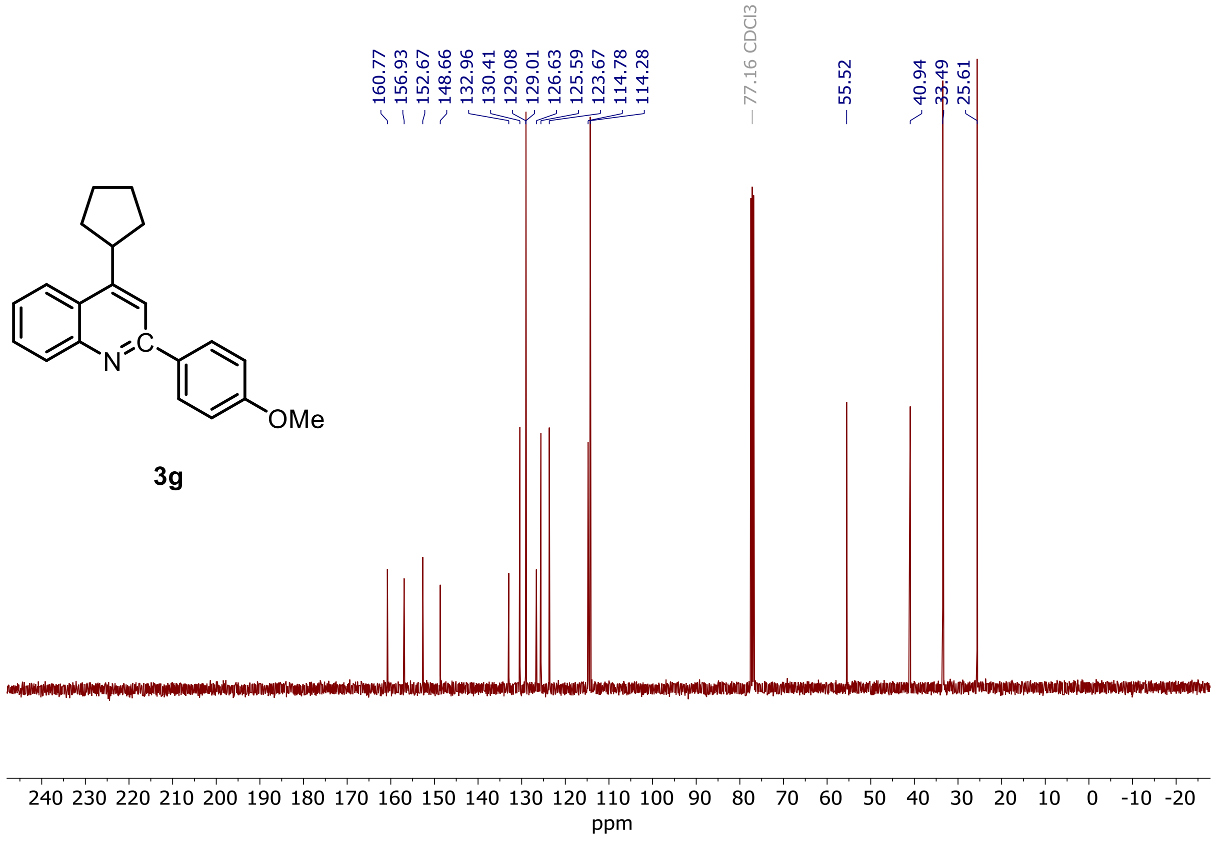
160.77 156.93 152.67 148.66 132.96 130.41 129.08 129.01 126.63 125.59 123.67 114.78 114.28
77.16 CDCl3
55.52
40.94 33.49 25.61
N
C
OMe
3g
240 230 220 210 200 190 180 170 160 150 140 130 120 110 100 90 80 70 60 50 40 30 20 10 0 -10 -20
ppm

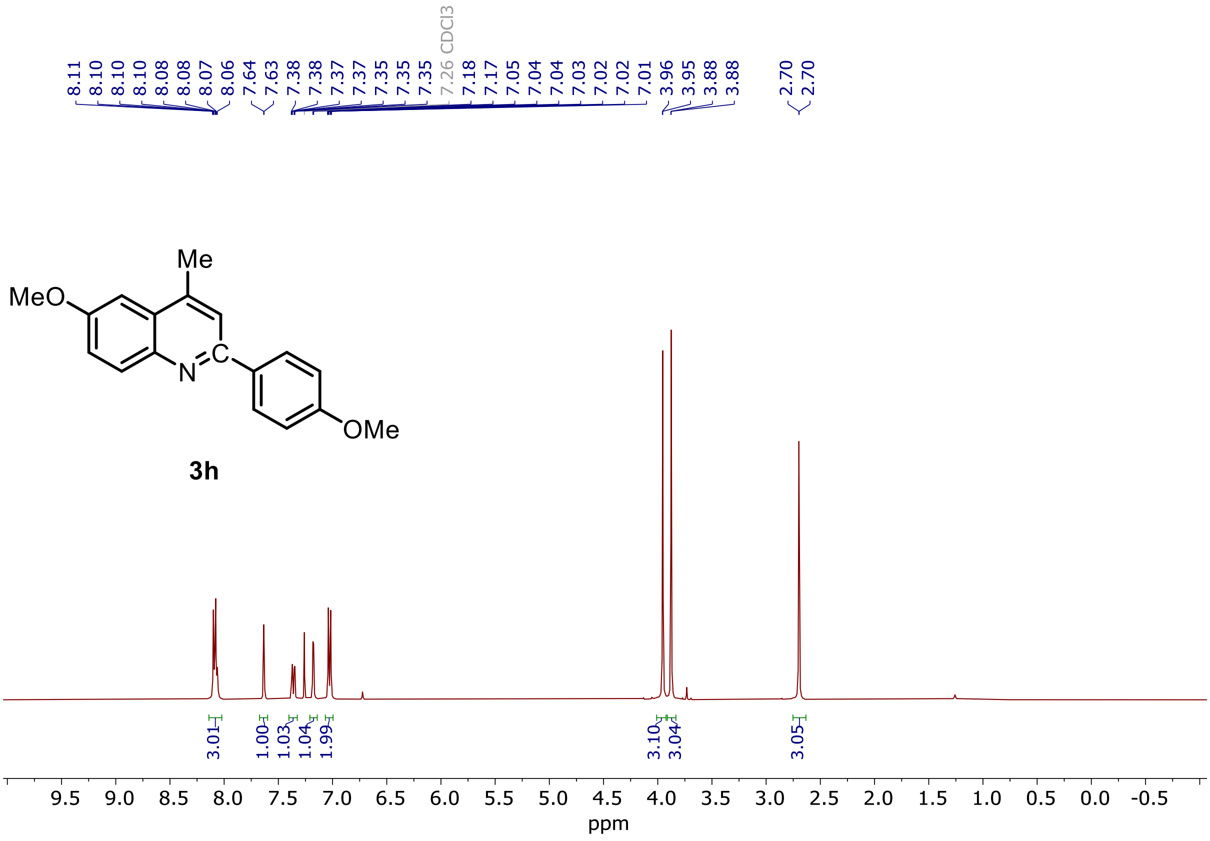
8.11
8.10
8.10
8.10
8.08
8.08
8.07
8.06
7.64
7.63
7.38
7.38
7.37
7.37
7.35
7.35
7.35
7.26 CDCl3
7.18
7.17
7.05
7.04
7.04
7.03
7.02
7.02
7.01
3.96
3.95
3.88
3.88
2.70
2.70
Me
MeO
N
C
OMe
3.01
1.00
1.03
1.04
1.99
3.10
3.04
3.05
9.5 9.0 8.5 8.0 7.5 7.0 6.5 6.0 5.5 5.0 4.5 4.0 3.5 3.0 2.5 2.0 1.5 1.0 0.5 0.0 -0.5
ppm

3h

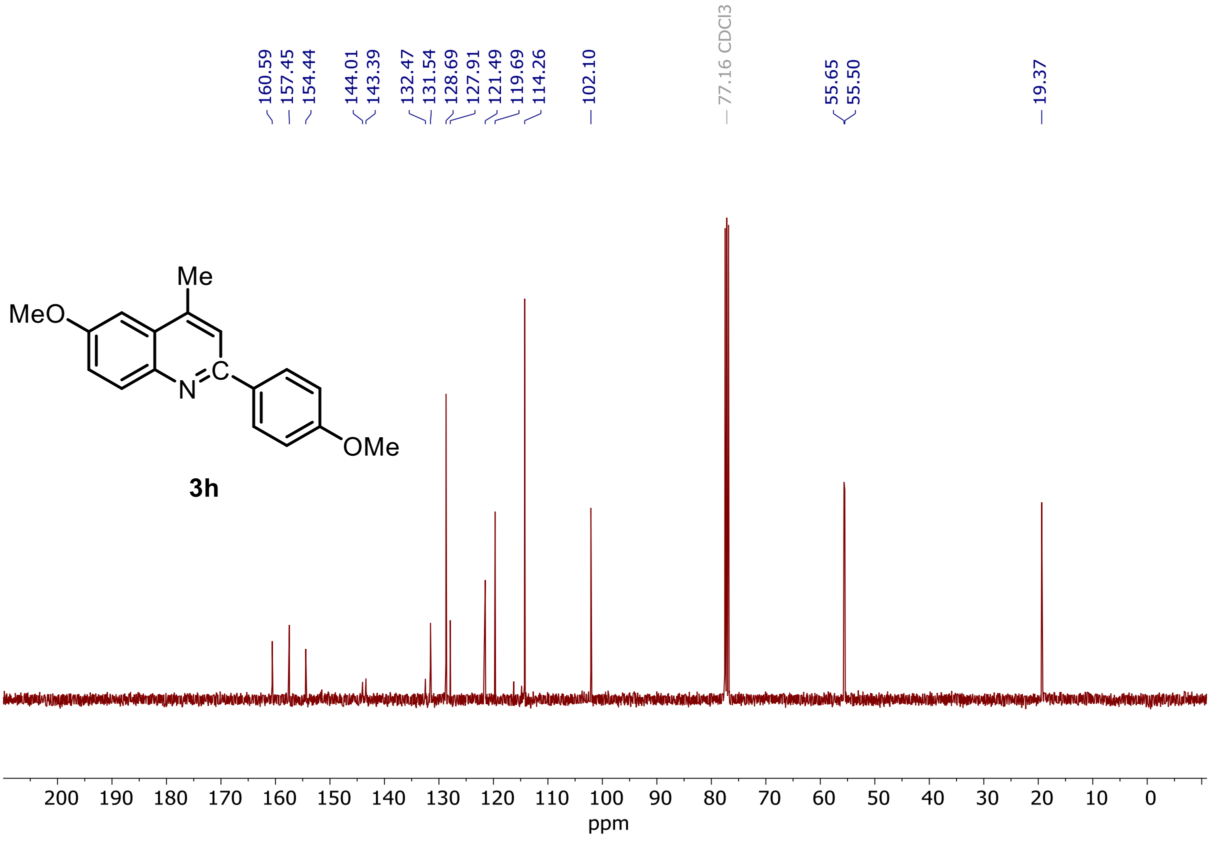
160.59
157.45
154.44
144.01
143.39
132.47
131.54
128.69
127.91
121.49
119.69
114.26
102.10
77.16 CDCl3
55.65
55.50
19.37
Me
MeO
N
C
OMe
200 190 180 170 160 150 140 130 120 110 100 90 80 70 60 50 40 30 20 10 0
ppm

3h

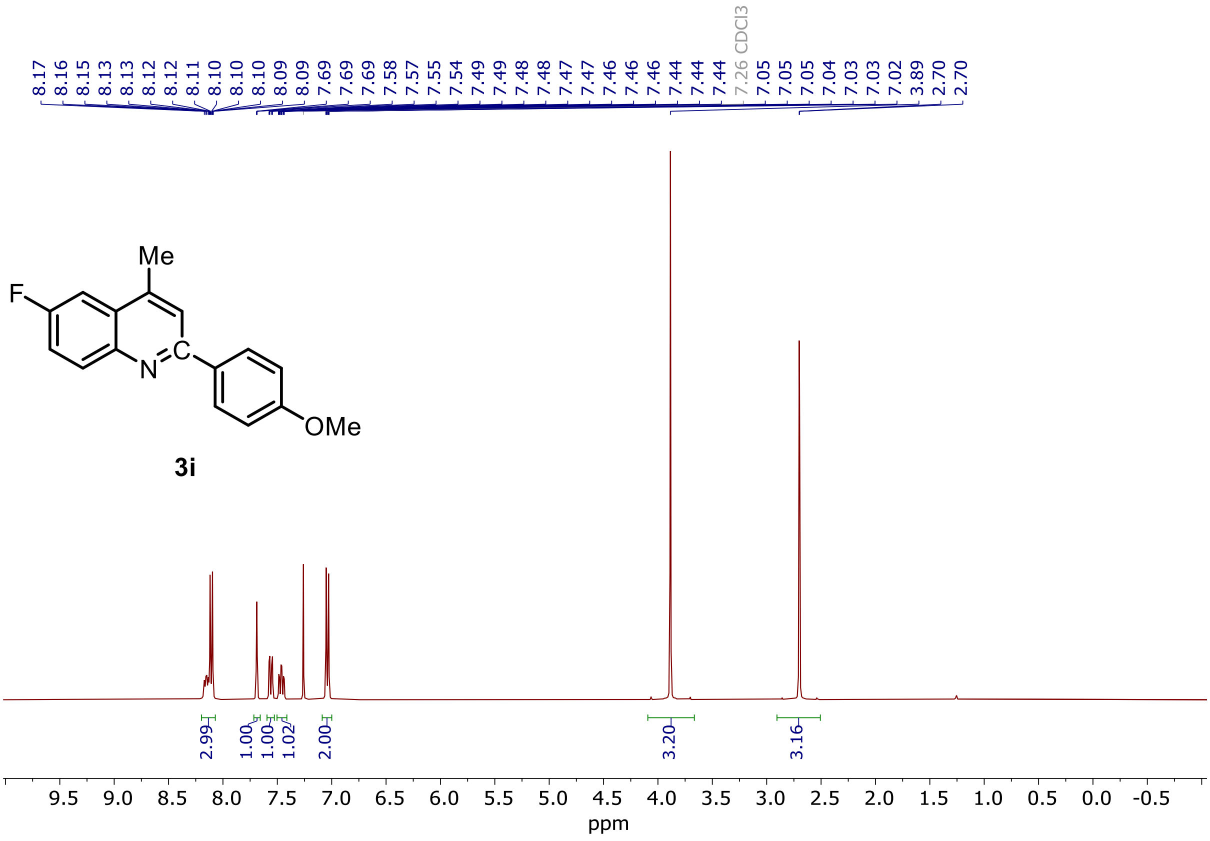

8.17
8.16
8.15
8.13
8.13
8.12
8.12
8.11
8.10
8.10
8.10
8.09
8.09
7.69
7.69
7.69
7.58
7.57
7.55
7.54
7.49
7.49
7.48
7.48
7.47
7.47
7.46
7.46
7.44
7.44
7.44
7.26 CDCl3
7.05
7.05
7.05
7.04
7.03
7.03
7.02
3.89
2.70
2.70
Me
F
N
C
OMe
3i
2.99
1.00
1.00
1.02
2.00
3.20
3.16
9.5 9.0 8.5 8.0 7.5 7.0 6.5 6.0 5.5 5.0 4.5 4.0 3.5 3.0 2.5 2.0 1.5 1.0 0.5 0.0 -0.5
ppm

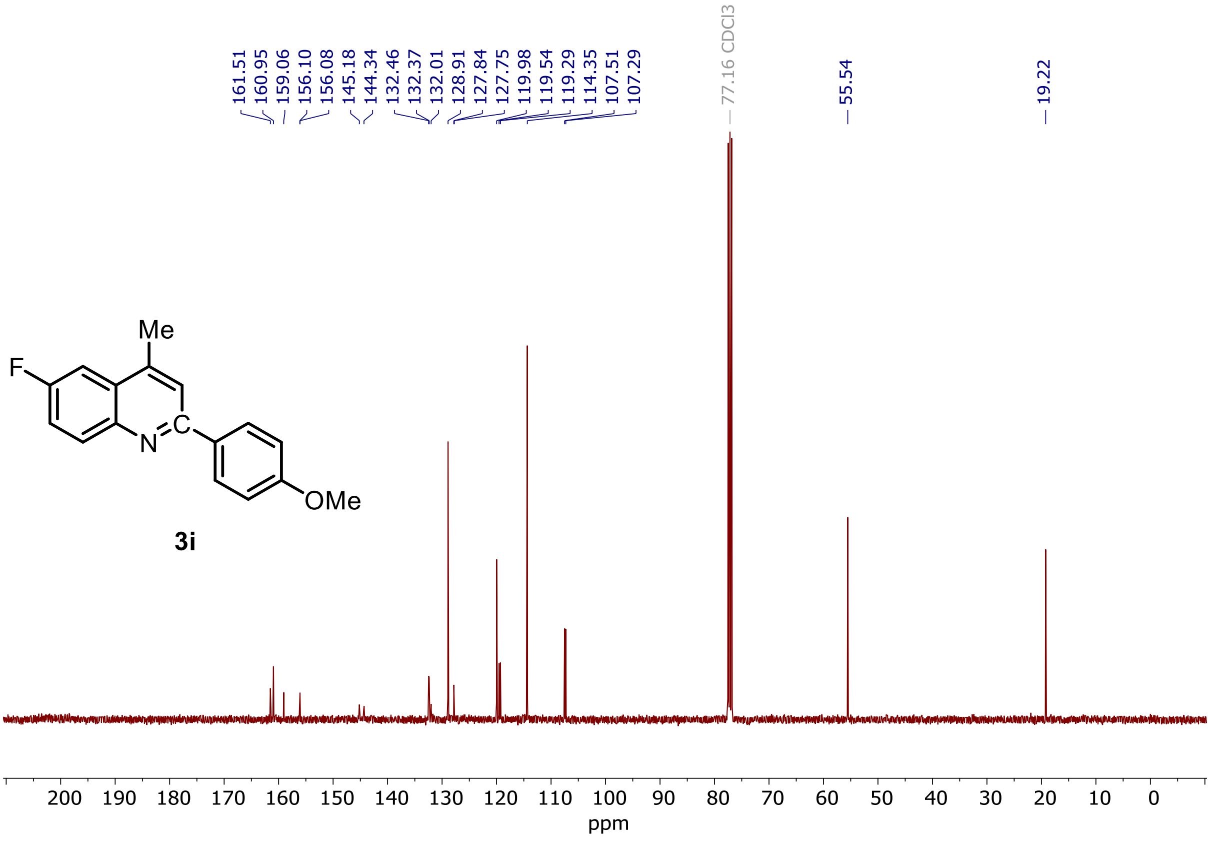

161.51
160.95
159.06
156.10
156.08
145.18
144.34
132.46
132.37
132.01
128.91
127.84
127.75
119.98
119.54
119.29
114.35
107.51
107.29
77.16 CDCl3
55.54
19.22
Me
F
N
C
OMe
3i
200 190 180 170 160 150 140 130 120 110 100 90 80 70 60 50 40 30 20 10 0
ppm

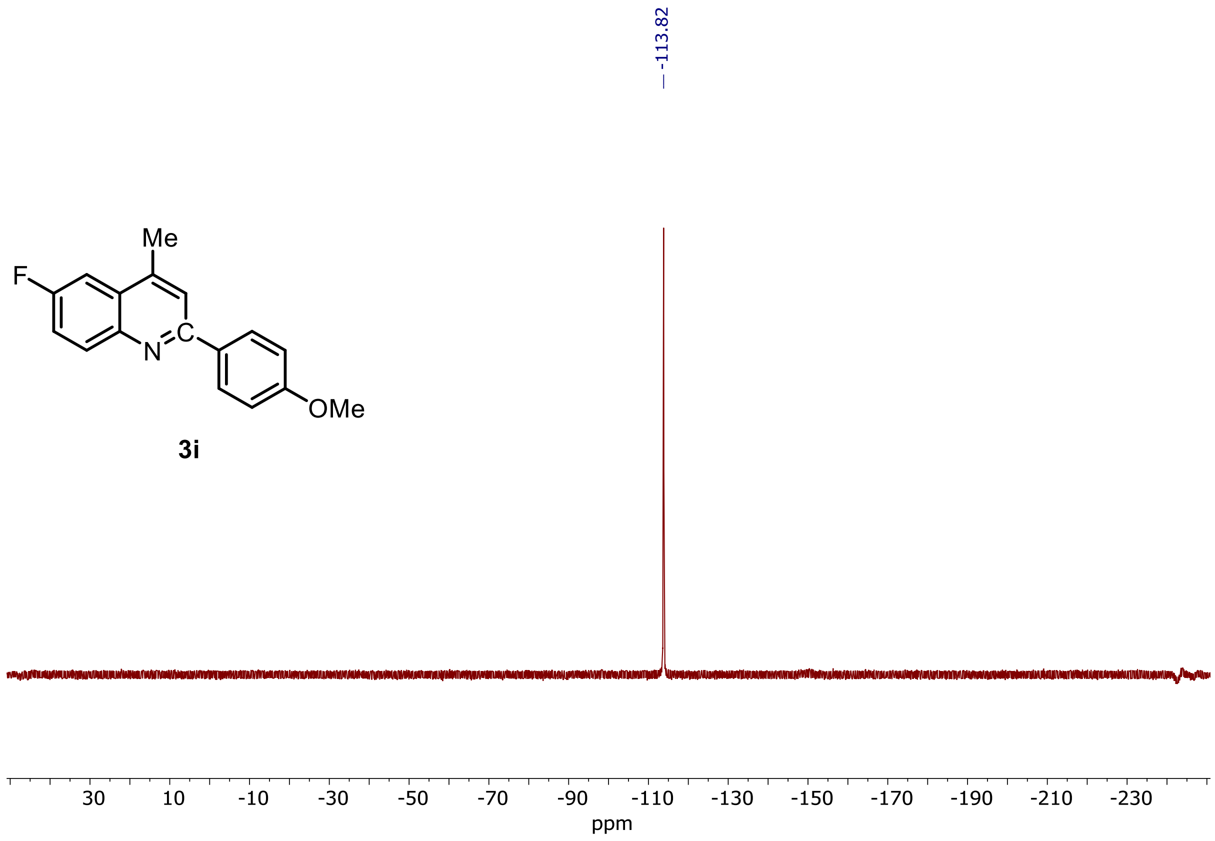

-113.82
Me
F
N
C
OMe
3i
30 10 -10 -30 -50 -70 -90 -110 -130 -150 -170 -190 -210 -230
ppm

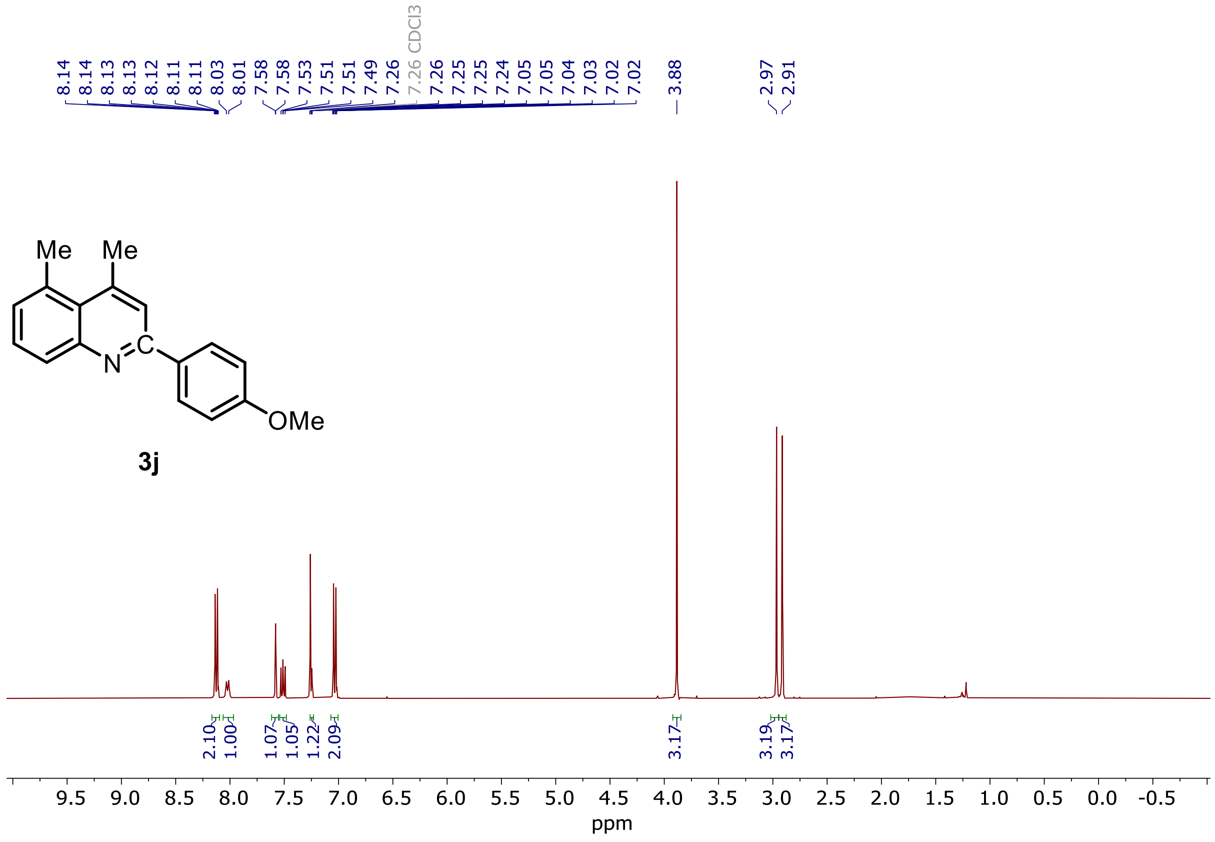

8.14 8.14 8.13 8.13 8.12 8.11 8.11 8.03 8.01 7.58 7.58 7.53 7.51 7.51 7.49 7.26 7.26 CDCl3 7.26 7.25 7.25 7.24 7.05 7.05 7.04 7.03 7.02 7.02
3.88
2.97 2.91
Me Me
N
C
OMe
3j
2.10 1.00 1.07 1.05 1.22 2.09 3.17 3.19 3.17
9.5 9.0 8.5 8.0 7.5 7.0 6.5 6.0 5.5 5.0 4.5 4.0 3.5 3.0 2.5 2.0 1.5 1.0 0.5 0.0 -0.5
ppm

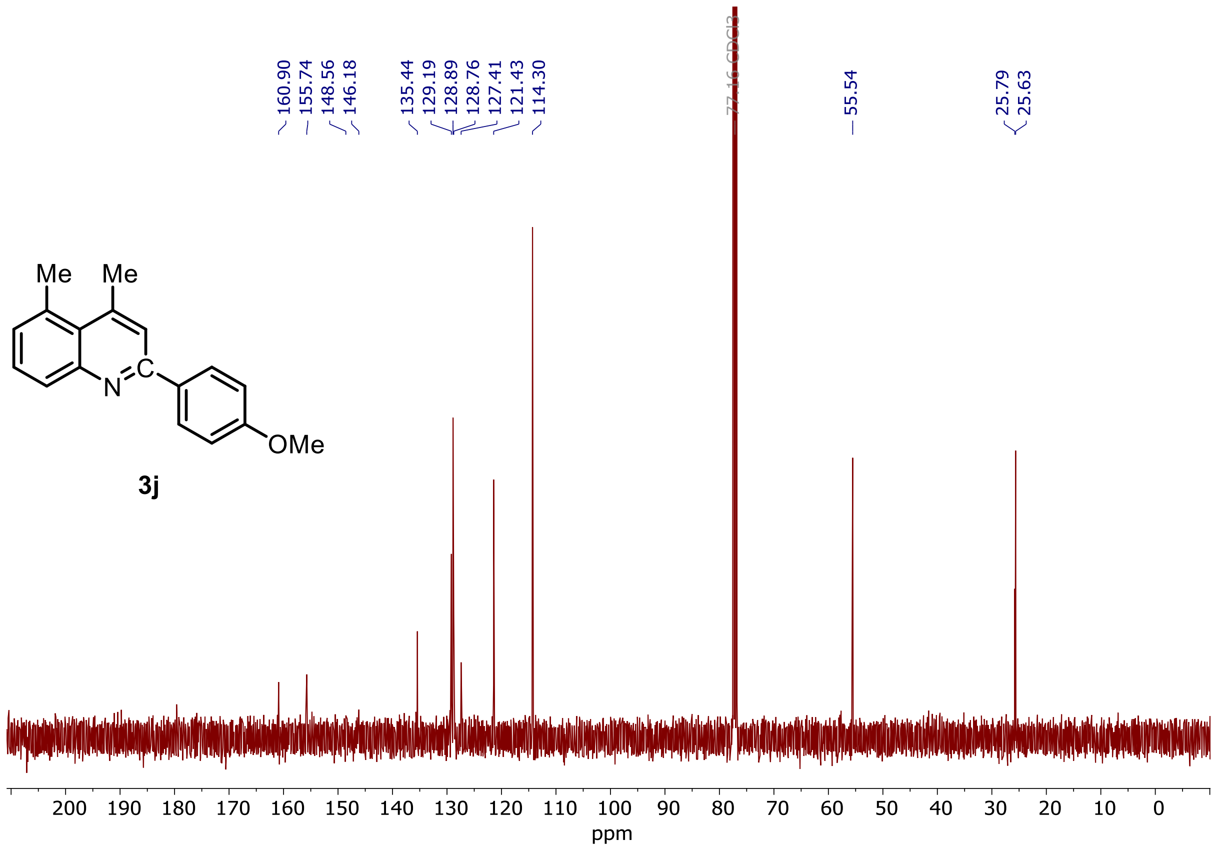
160.90
155.74
148.56
146.18
135.44
129.19
128.89
128.76
127.41
121.43
114.30
77.16 CDCl3
55.54
25.79
25.63
Me
Me
N
C
OMe
3j
200 190 180 170 160 150 140 130 120 110 100 90 80 70 60 50 40 30 20 10 0
ppm

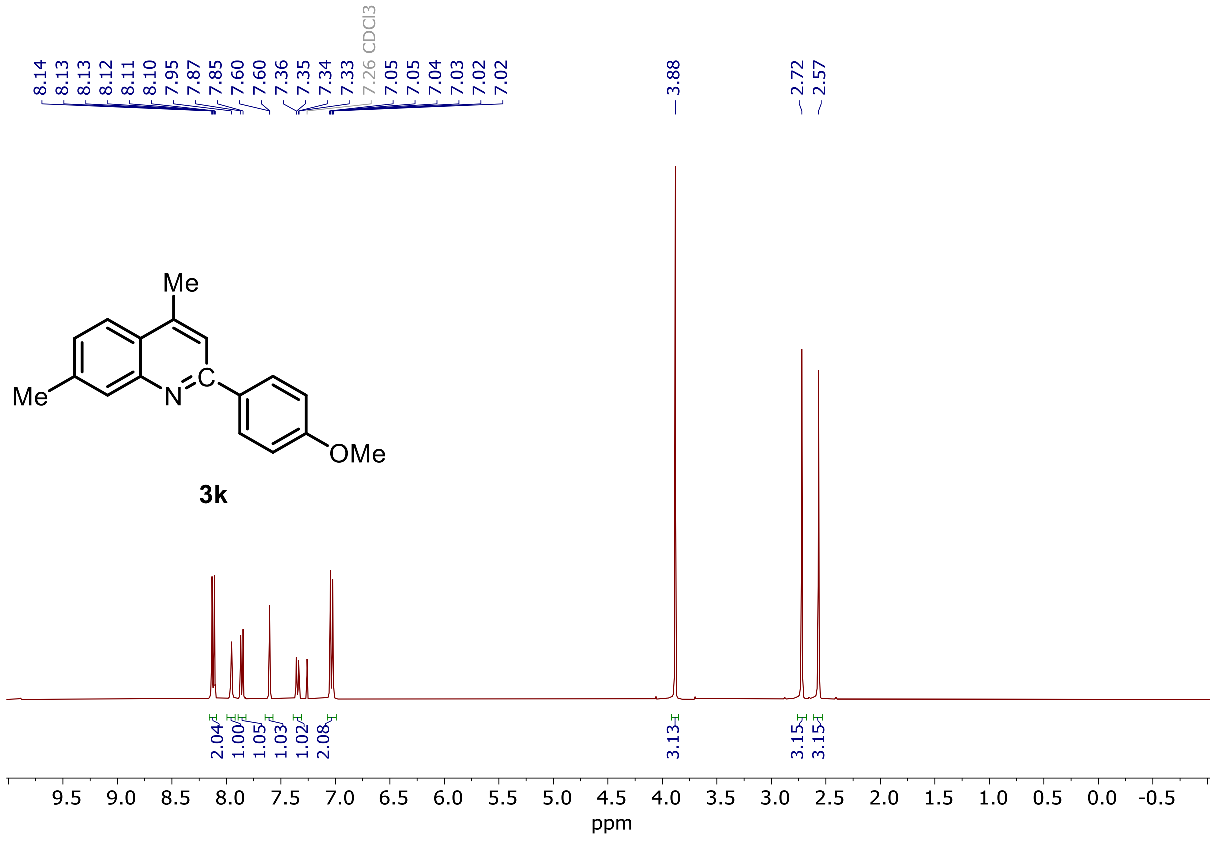
8.14
8.13
8.13
8.12
8.11
8.10
7.95
7.87
7.85
7.60
7.60
7.36
7.35
7.34
7.33
7.26 CDCl3
7.05
7.05
7.04
7.03
7.02
7.02
3.88
2.72
2.57
Me
Me
N
C
OMe
3k
2.04
1.00
1.05
1.03
1.02
2.08
3.13
3.15
3.15
9.5 9.0 8.5 8.0 7.5 7.0 6.5 6.0 5.5 5.0 4.5 4.0 3.5 3.0 2.5 2.0 1.5 1.0 0.5 0.0 -0.5
ppm

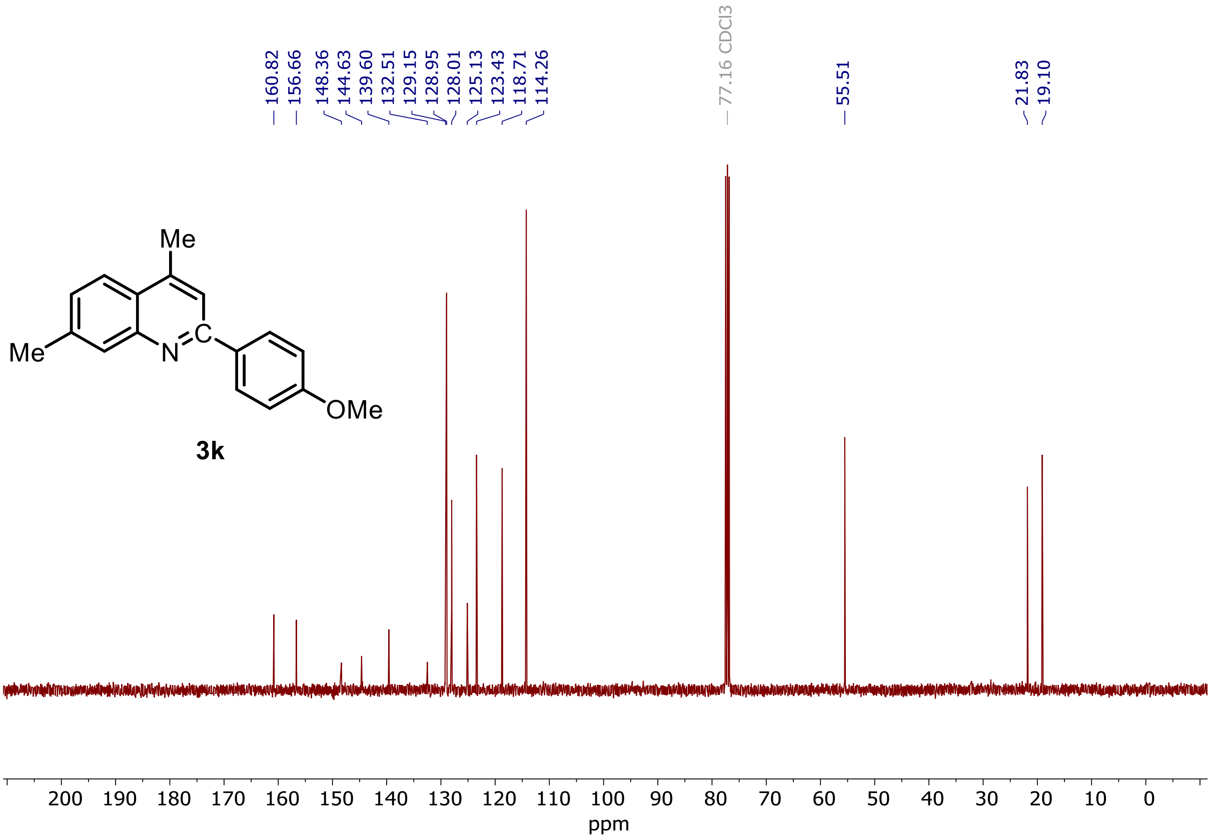

160.82
156.66
148.36
144.63
139.60
132.51
129.15
128.95
128.01
125.13
123.43
118.71
114.26
77.16 CDCl3
55.51
21.83
19.10
Me
Me
N
C
OMe
200 190 180 170 160 150 140 130 120 110 100 90 80 70 60 50 40 30 20 10 0
ppm

3k

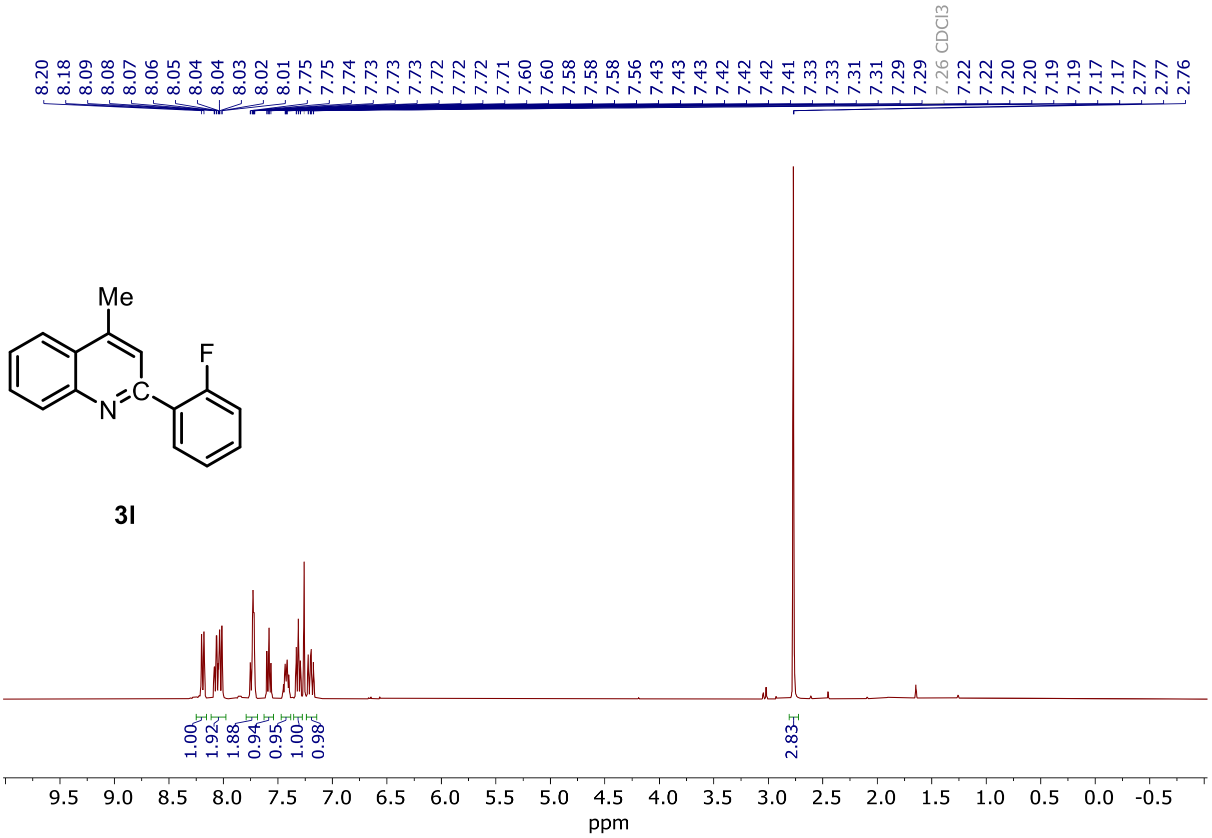

8.20 8.18 8.09 8.08 8.07 8.06 8.05 8.04 8.04 8.03 8.02 8.01 7.75 7.75 7.74 7.73 7.73 7.73 7.72 7.72 7.72 7.71 7.60 7.60 7.58 7.58 7.56 7.43 7.43 7.43 7.42 7.42 7.42 7.41 7.33 7.33 7.31 7.31 7.29 7.29 7.26 CDCl3 7.22 7.22 7.20 7.20 7.19 7.19 7.17 7.17 2.77 2.77 2.76
Me
F
N
C
1.00 1.92 1.88 0.94 0.95 1.00 0.98
2.83
9.5 9.0 8.5 8.0 7.5 7.0 6.5 6.0 5.5 5.0 4.5 4.0 3.5 3.0 2.5 2.0 1.5 1.0 0.5 0.0 -0.5
ppm

3l

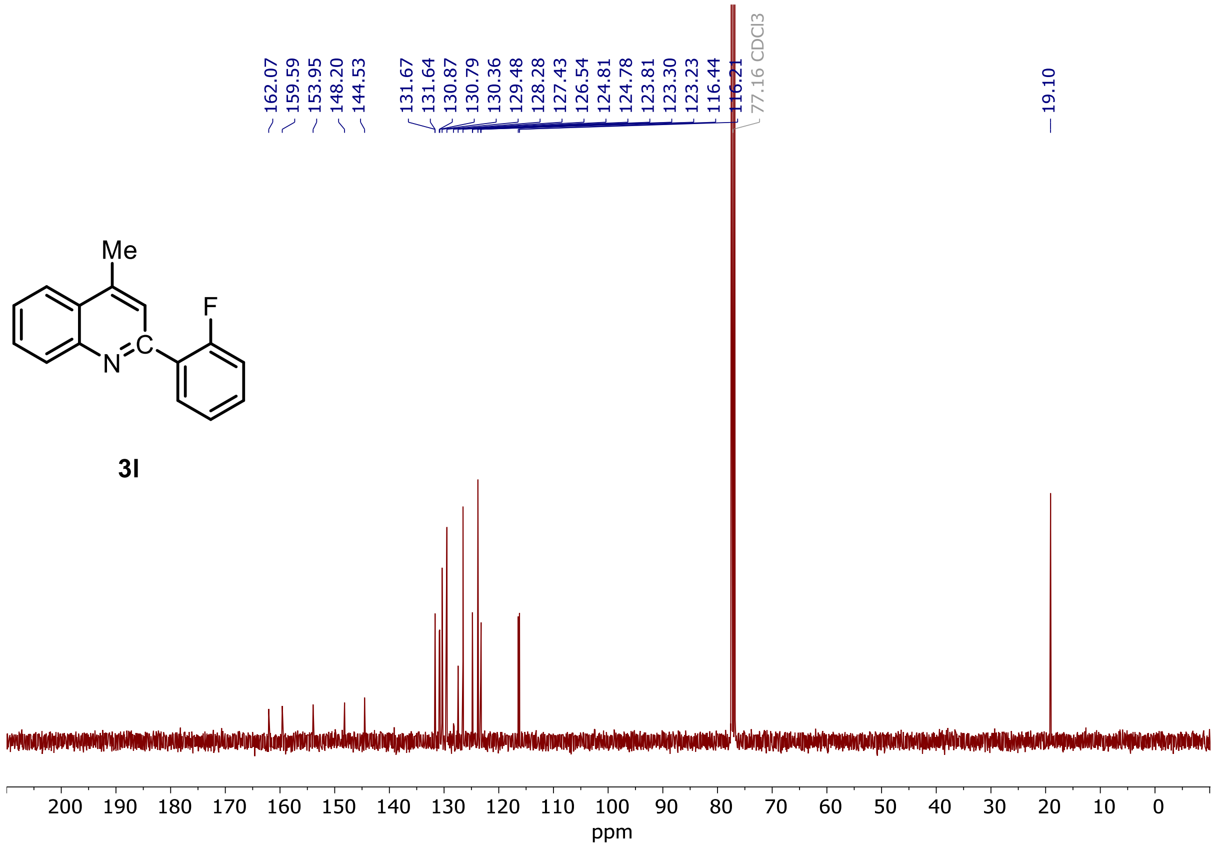
162.07
159.59
153.95
148.20
144.53
131.67
131.64
130.87
130.79
130.36
129.48
128.28
127.43
126.54
124.81
124.78
123.81
123.30
123.23
116.44
116.21
77.16 CDCl3
19.10
Me
F
N
C
3l
200 190 180 170 160 150 140 130 120 110 100 90 80 70 60 50 40 30 20 10 0
ppm

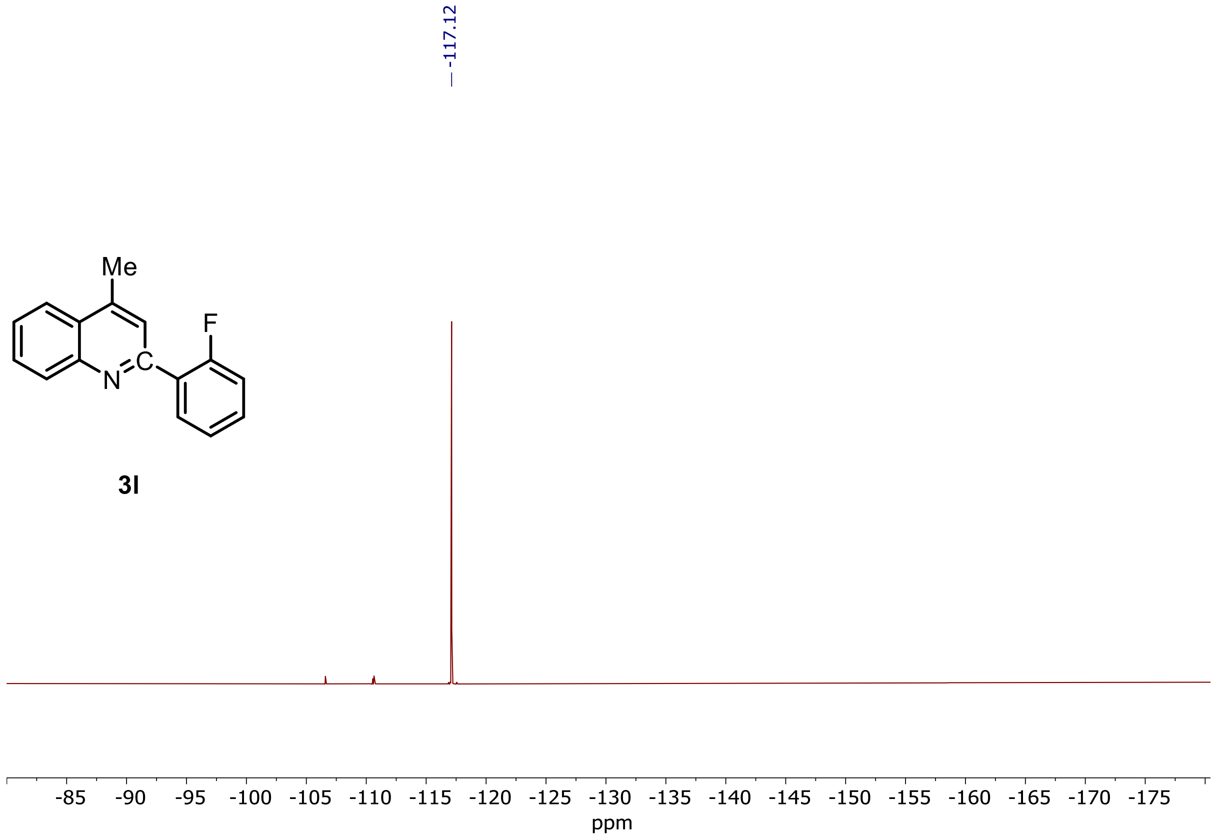
-117.12
Me
F
N
C
3l
-85 -90 -95 -100 -105 -110 -115 -120 -125 -130 -135 -140 -145 -150 -155 -160 -165 -170 -175
ppm

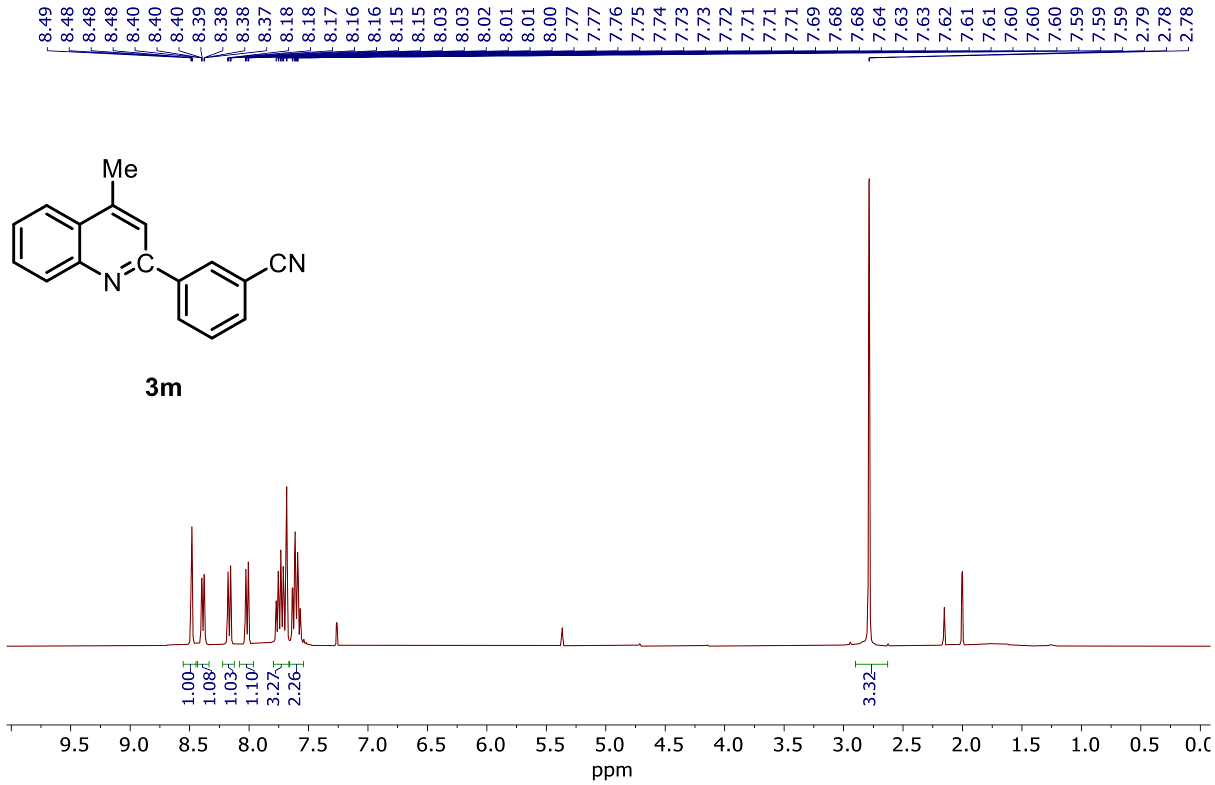
8.49
8.48
8.48
8.40
8.40
8.40
8.39
8.38
8.38
8.37
8.18
8.18
8.17
8.16
8.16
8.15
8.15
8.03
8.03
8.02
8.01
8.01
8.00
7.77
7.77
7.76
7.75
7.74
7.73
7.73
7.72
7.71
7.71
7.71
7.69
7.68
7.68
7.64
7.63
7.63
7.62
7.61
7.61
7.60
7.60
7.60
7.59
7.59
7.59
2.79
2.78
2.78
Me
N
C
CN
3m
1.00
1.08
1.03
1.10
3.27
2.26
3.32
9.5 9.0 8.5 8.0 7.5 7.0 6.5 6.0 5.5 5.0 4.5 4.0 3.5 3.0 2.5 2.0 1.5 1.0 0.5 0.0
ppm

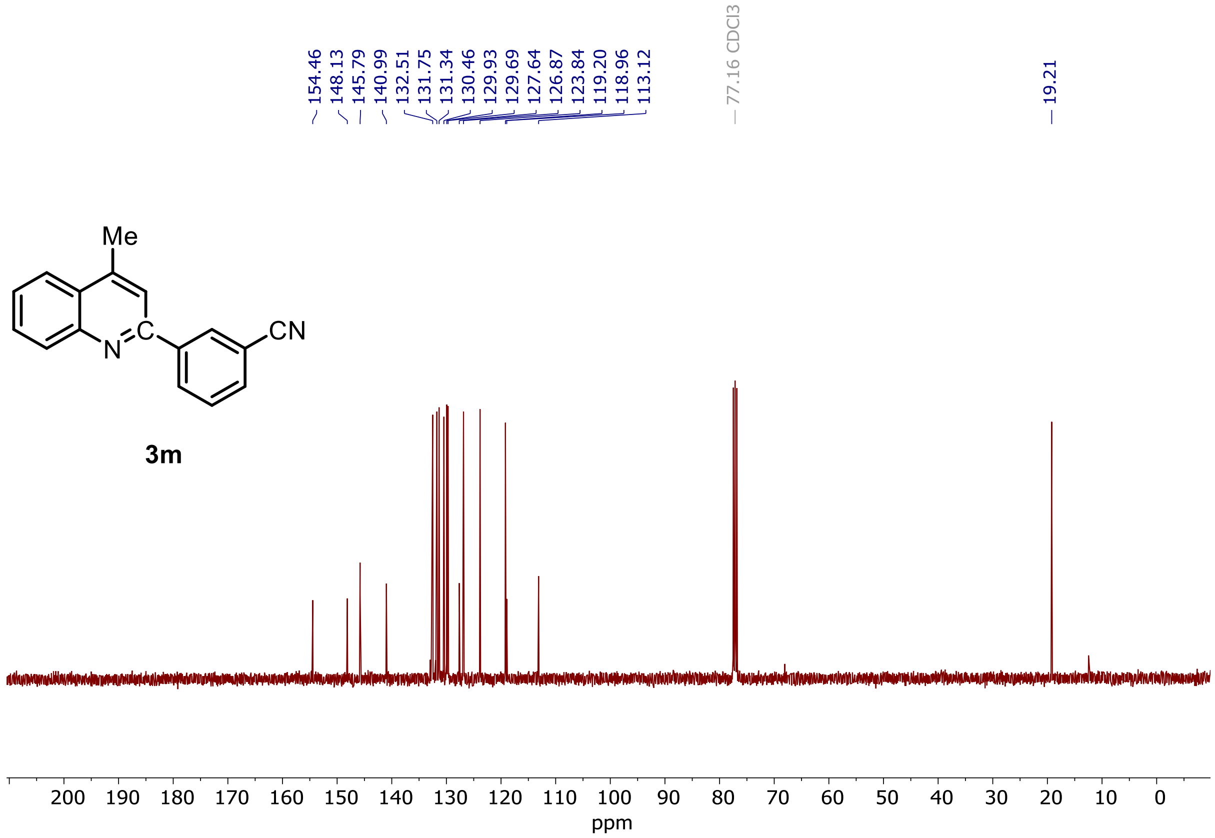
154.46
148.13
145.79
140.99
132.51
131.75
131.34
130.46
129.93
129.69
127.64
126.87
123.84
119.20
118.96
113.12
77.16 CDCl3
19.21
Me
N
C
CN
3m
200 190 180 170 160 150 140 130 120 110 100 90 80 70 60 50 40 30 20 10 0
ppm

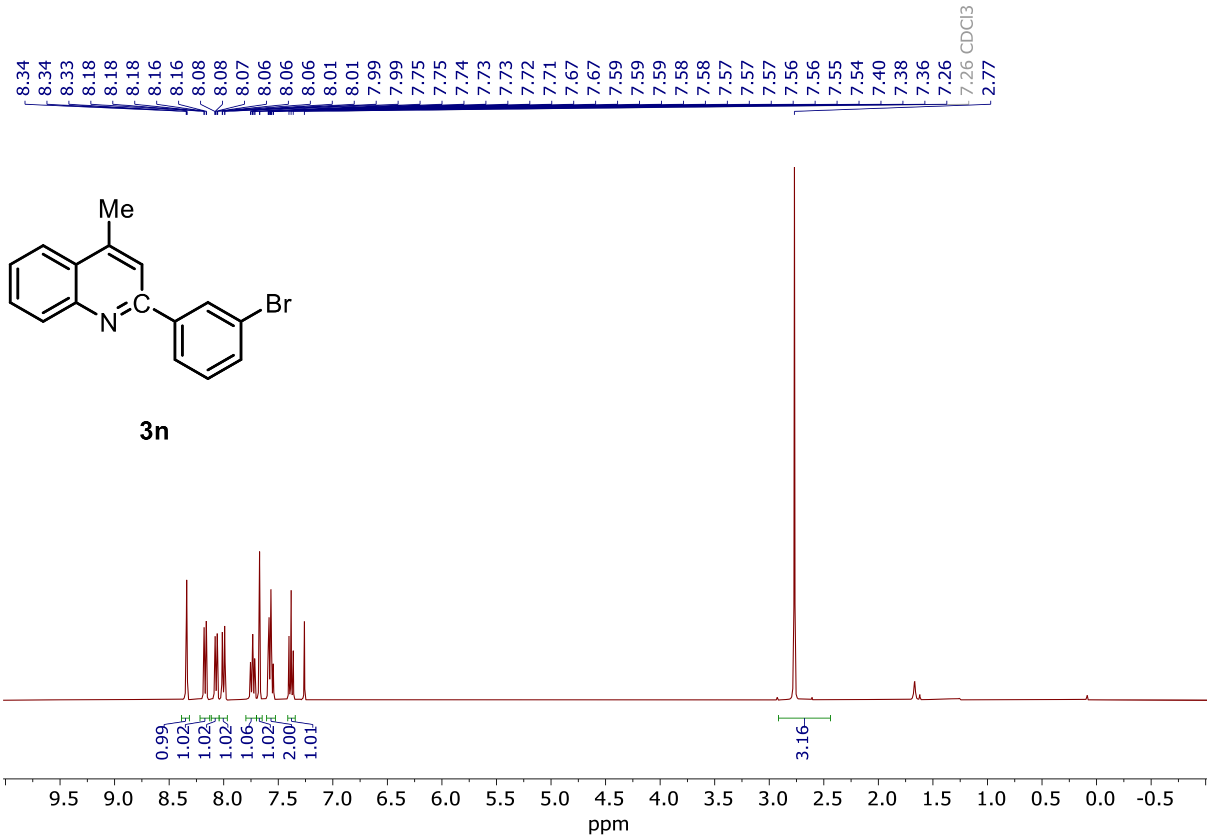
8.34 8.34 8.33 8.18 8.18 8.18 8.16 8.16 8.08 8.08 8.07 8.06 8.06 8.06 8.01 7.99 7.99 7.75 7.75 7.74 7.73 7.73 7.72 7.71 7.67 7.67 7.59 7.59 7.59 7.58 7.58 7.57 7.57 7.57 7.56 7.56 7.55 7.54 7.40 7.38 7.36 7.26 7.26 CDCl3 2.77
Me
N
C
Br
3n
0.99 1.02 1.02 1.02 1.06 1.02 2.00 1.01
3.16
9.5 9.0 8.5 8.0 7.5 7.0 6.5 6.0 5.5 5.0 4.5 4.0 3.5 3.0 2.5 2.0 1.5 1.0 0.5 0.0 -0.5
ppm

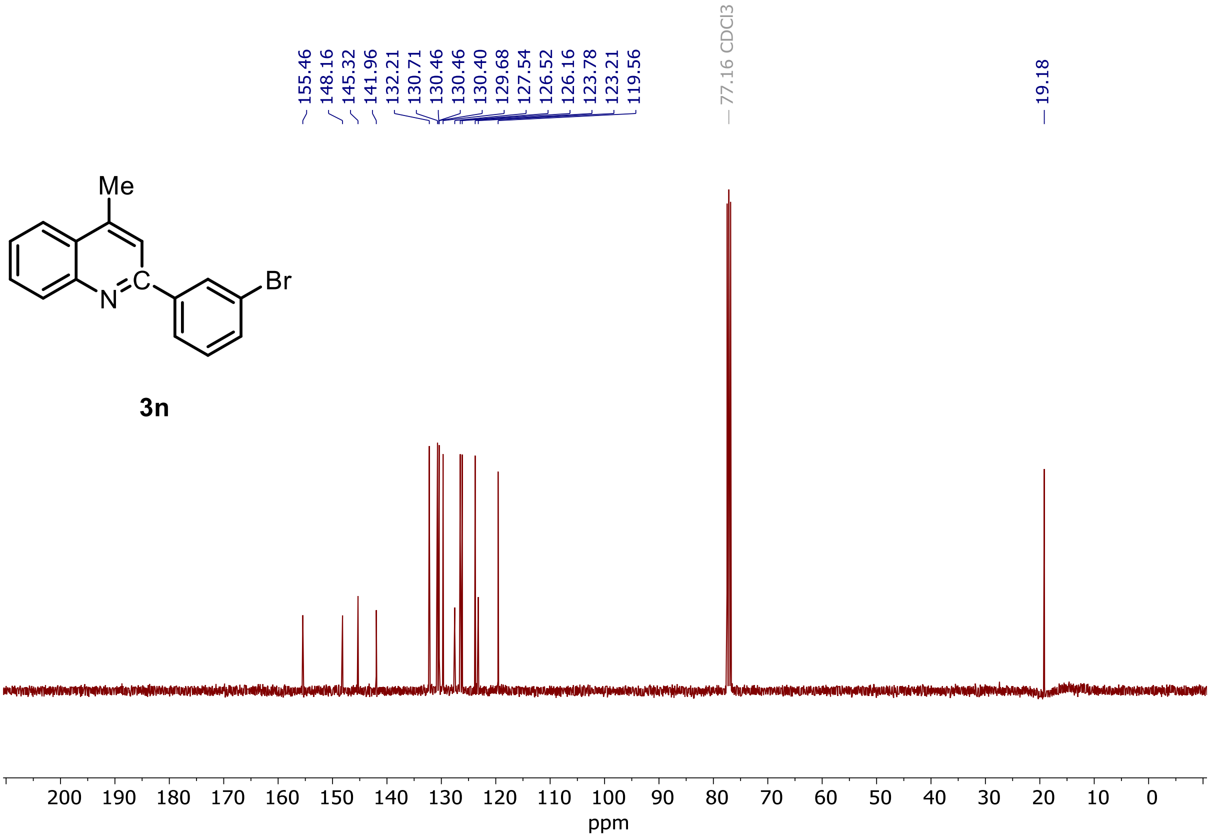
155.46 148.16 145.32 141.96 132.21 130.71 130.46 130.46 130.40 129.68 127.54 126.52 126.16 123.78 123.21 119.56
77.16 CDCl3
19.18
Me
N
C
Br
3n
200 190 180 170 160 150 140 130 120 110 100 90 80 70 60 50 40 30 20 10 0
ppm

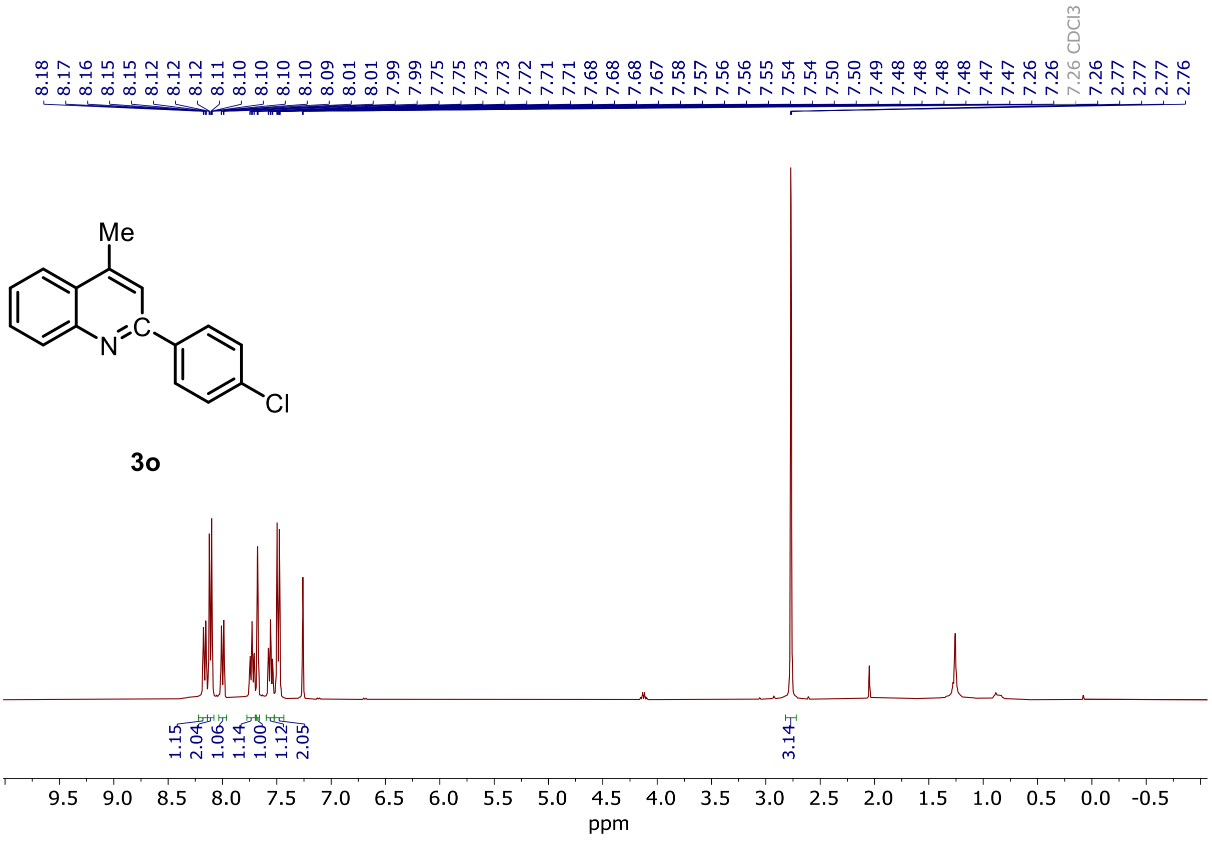
Me
N
C
Cl
3o
ppm

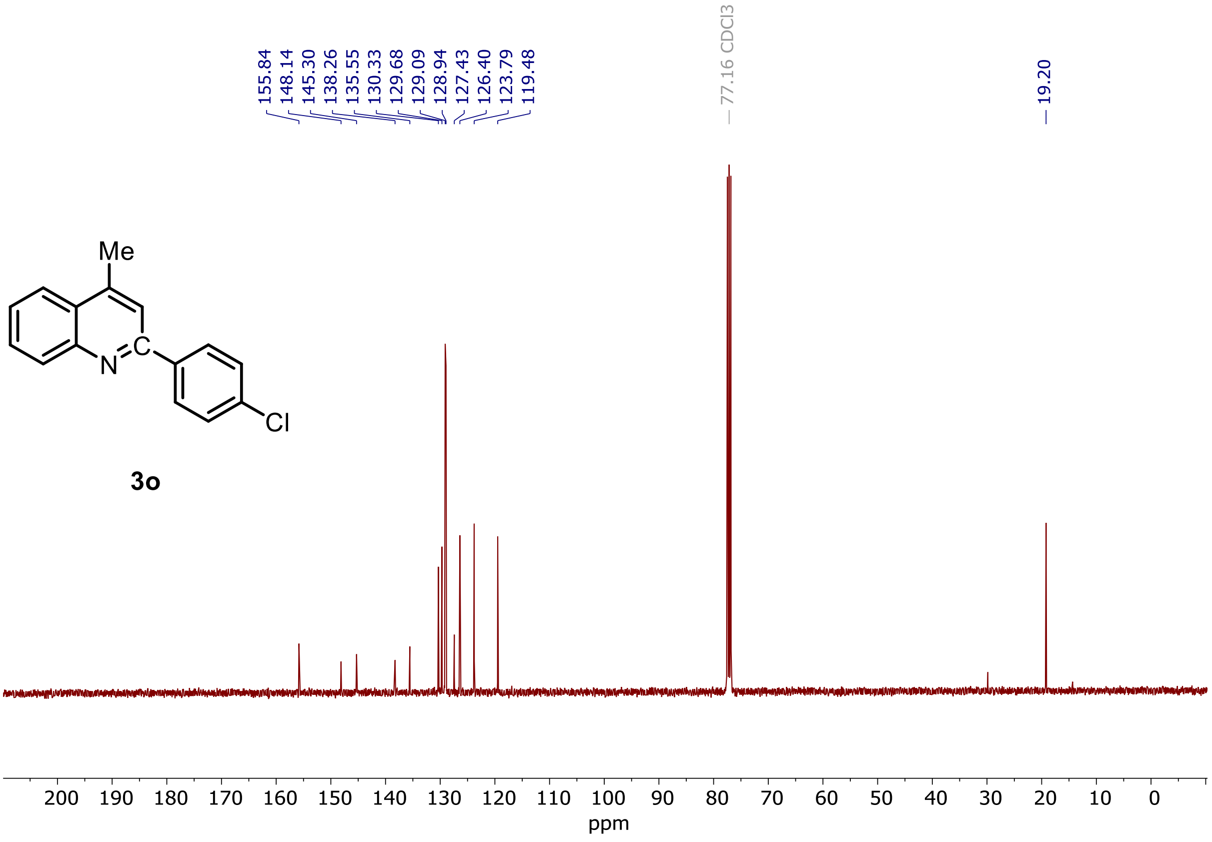
Me
N
C
Cl
3o
ppm

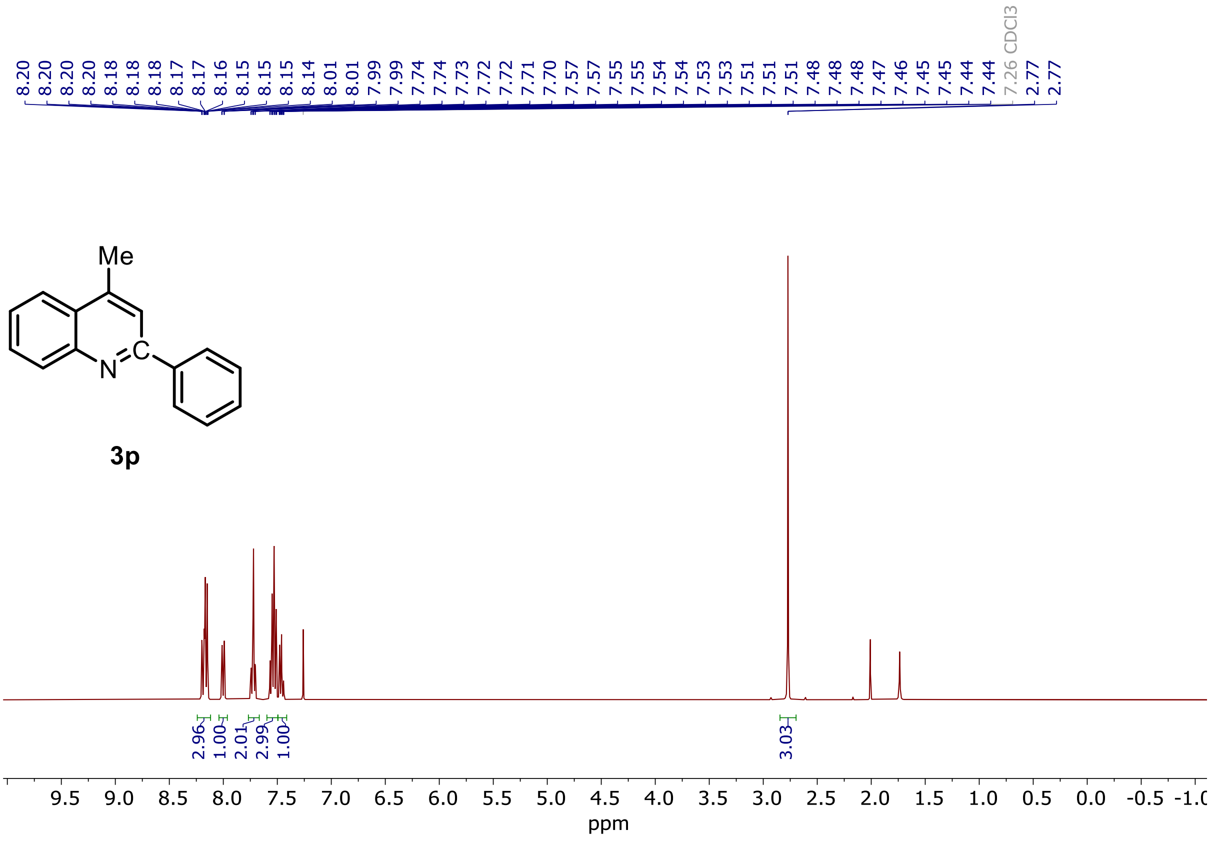
8.20
8.20
8.20
8.20
8.18
8.18
8.18
8.17
8.17
8.16
8.15
8.15
8.15
8.14
8.01
8.01
7.99
7.99
7.74
7.74
7.73
7.72
7.72
7.71
7.70
7.57
7.57
7.55
7.55
7.54
7.54
7.53
7.53
7.51
7.51
7.51
7.48
7.48
7.48
7.47
7.46
7.45
7.45
7.44
7.44
7.26 CDCl3
2.77
2.77
Me
N
C
3p
2.96
1.00
2.01
2.99
1.00
3.03
ppm

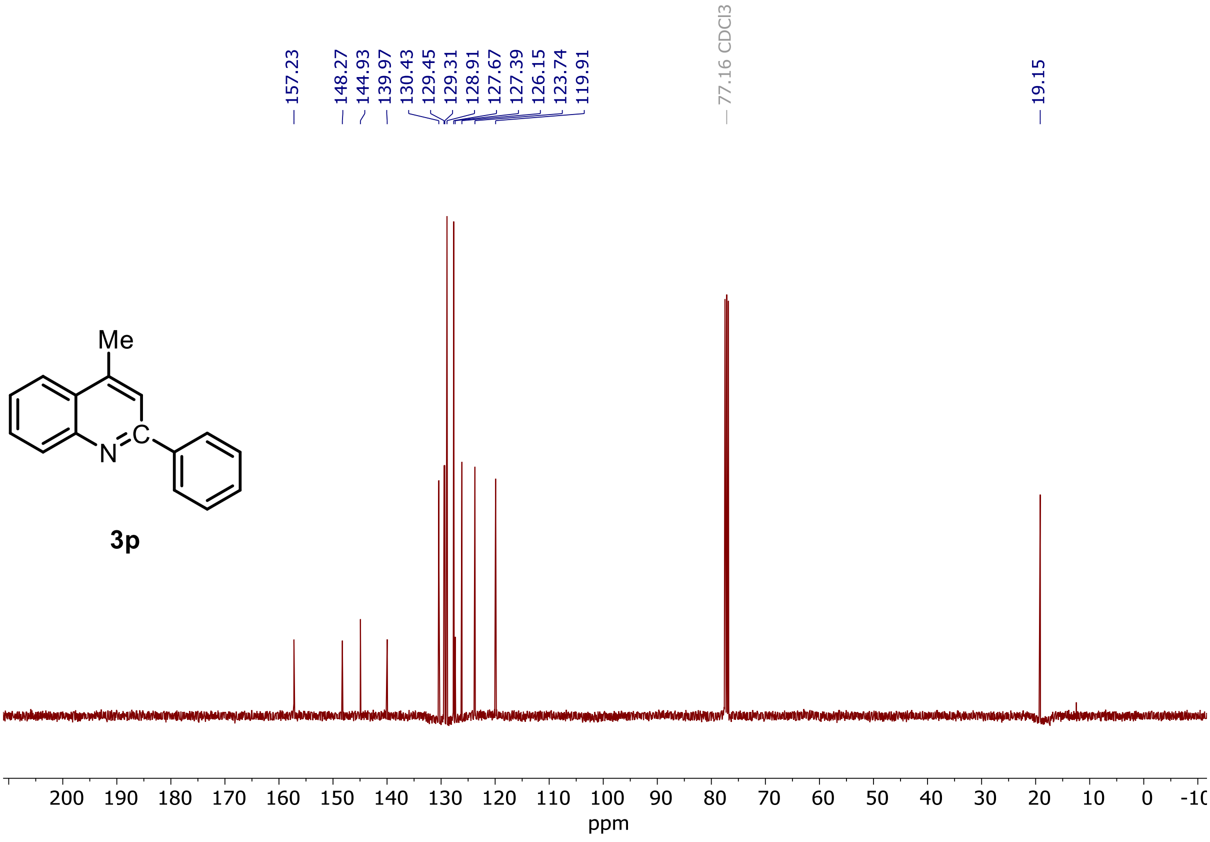
157.23
148.27
144.93
139.97
130.43
129.45
129.31
128.91
127.67
127.39
126.15
123.74
119.91
77.16 CDCl3
19.15
Me
N
C
3p
ppm

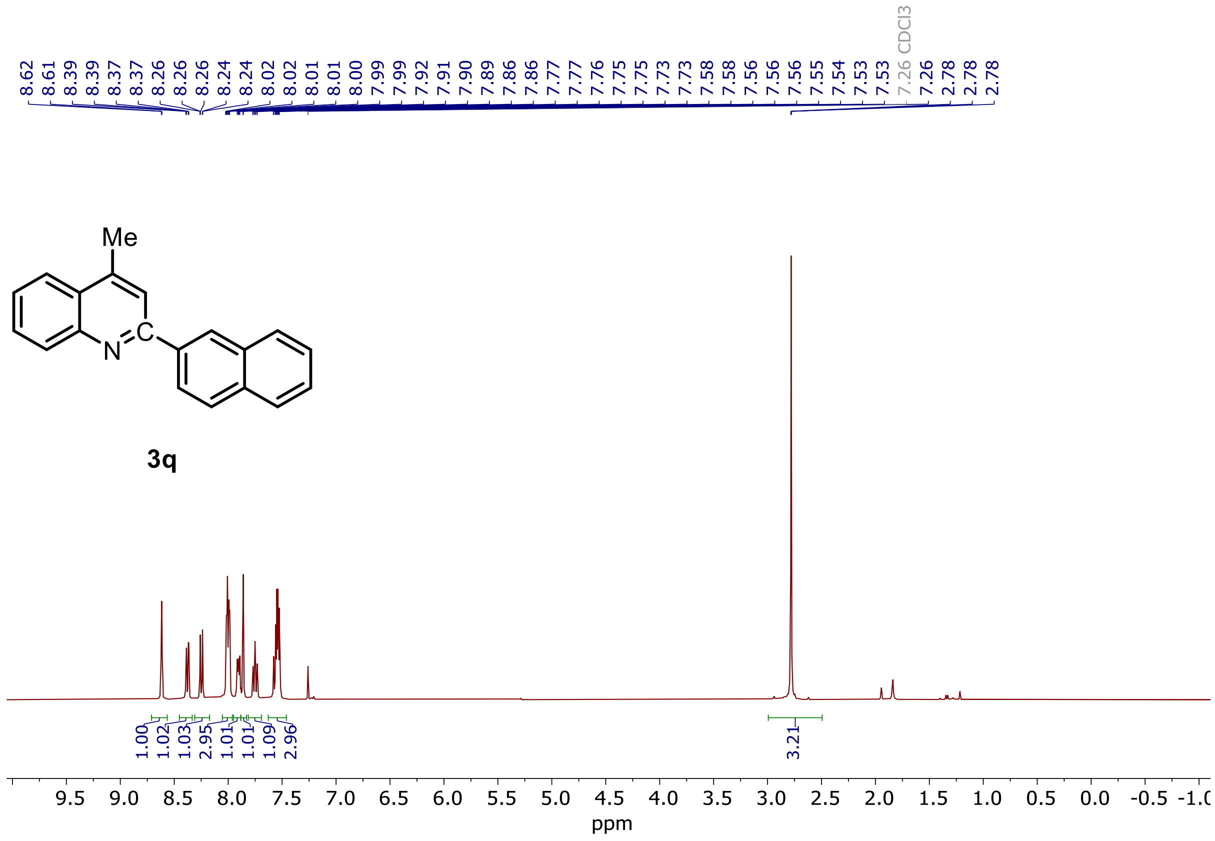

3q

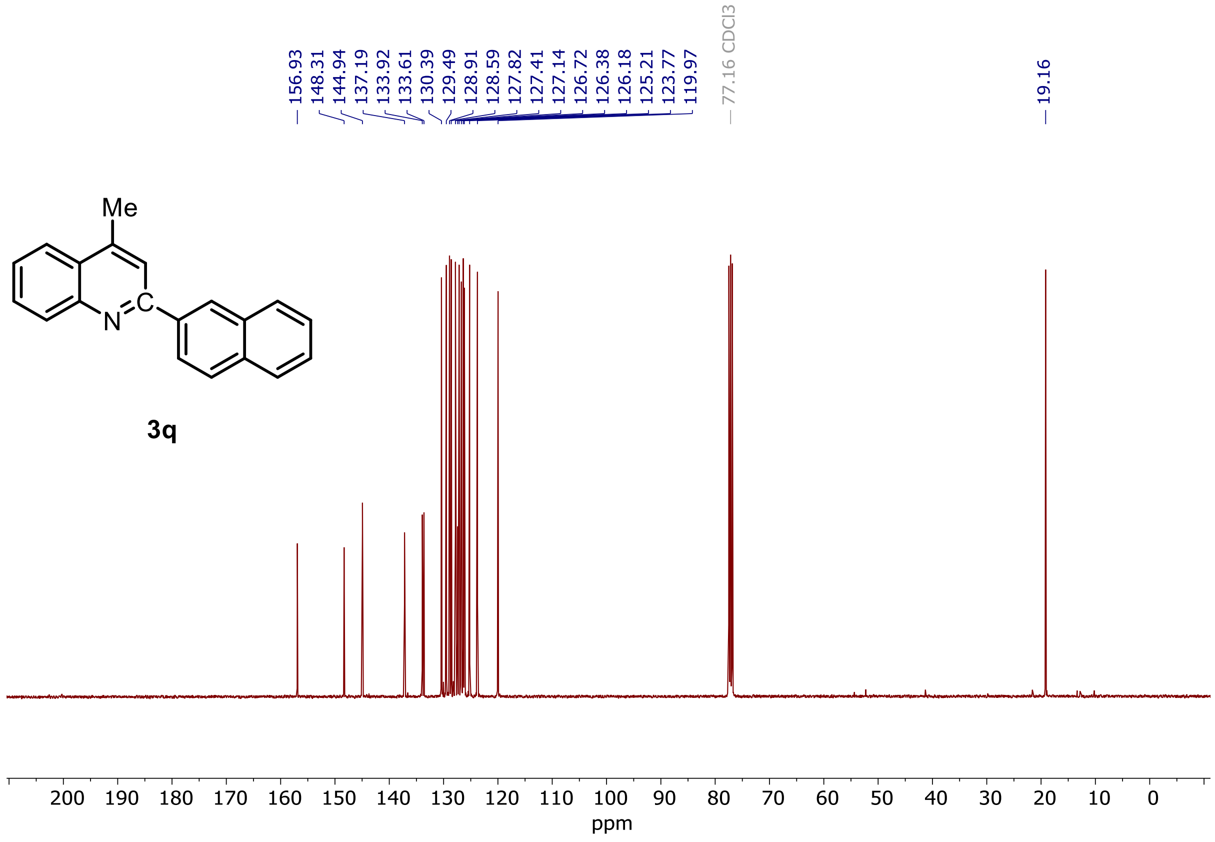

3q

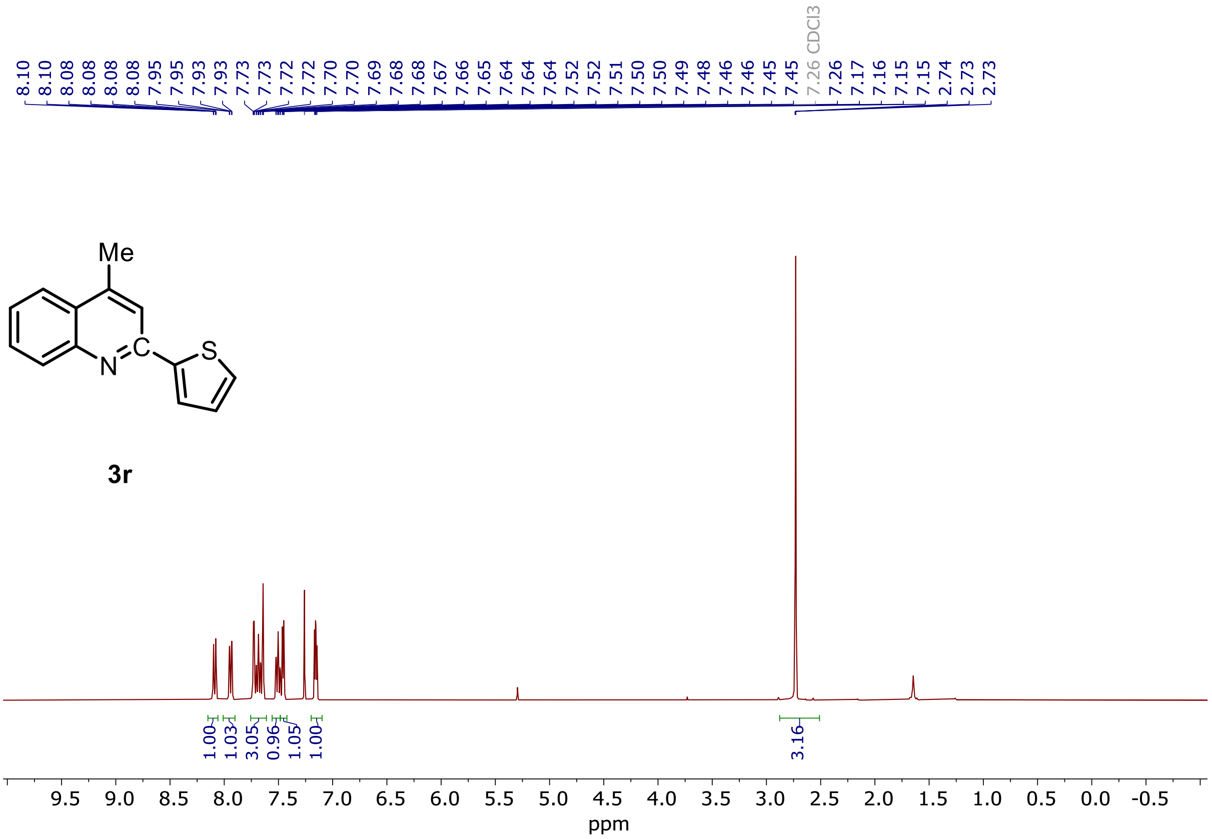
8.10 8.10 8.08 8.08 8.08 8.08 7.95 7.95 7.93 7.93 7.73 7.73 7.72 7.72 7.70 7.69 7.68 7.68 7.67 7.66 7.65 7.64 7.64 7.64 7.52 7.52 7.51 7.50 7.50 7.49 7.48 7.46 7.46 7.45 7.45 7.26 CDCl3 7.26 7.17 7.16 7.15 7.15 2.74 2.73 2.73
Me
N
C
S
1.00 1.03 3.05 0.96 1.05 1.00
3.16
9.5 9.0 8.5 8.0 7.5 7.0 6.5 6.0 5.5 5.0 4.5 4.0 3.5 3.0 2.5 2.0 1.5 1.0 0.5 0.0 -0.5
ppm

3r

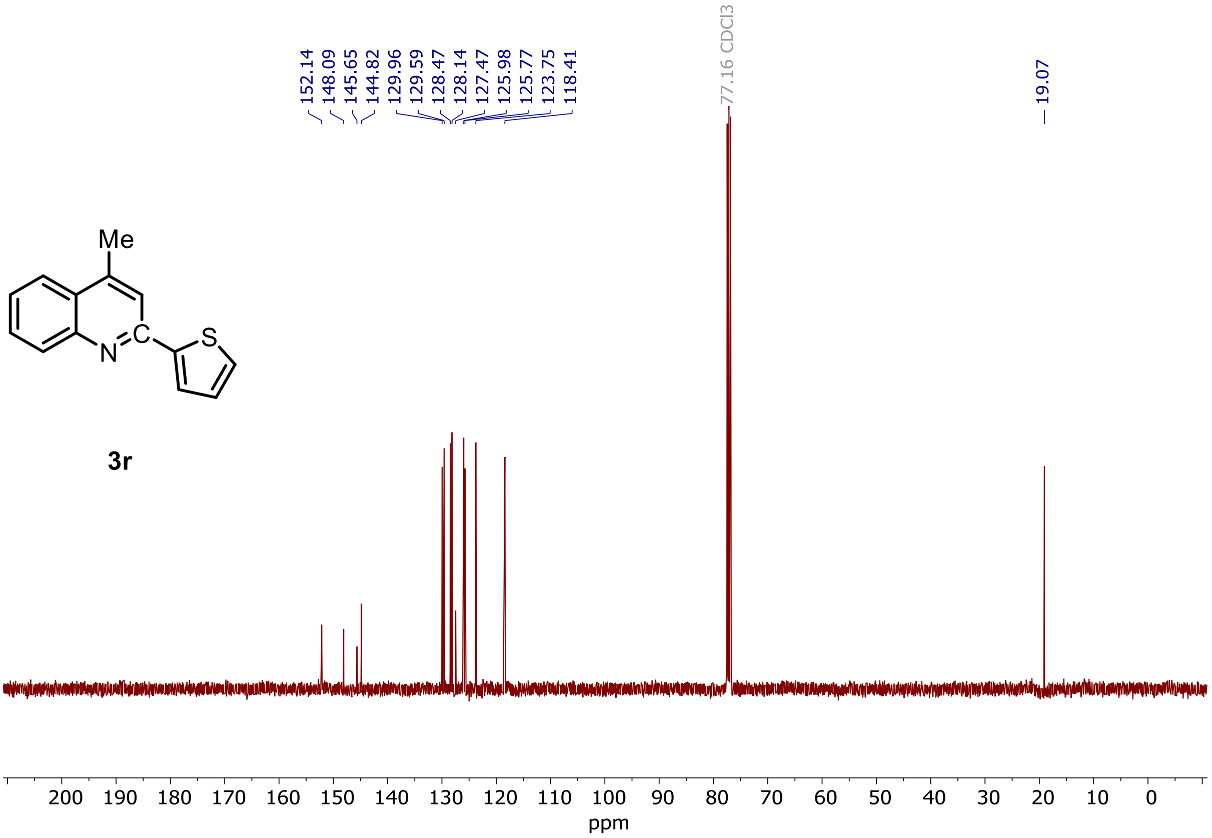
152.14 148.09 145.65 144.82 129.96 129.59 128.47 128.14 127.47 125.98 125.77 123.75 118.41
77.16 CDCl3
19.07
Me
N
C
S
200 190 180 170 160 150 140 130 120 110 100 90 80 70 60 50 40 30 20 10 0
ppm

3r

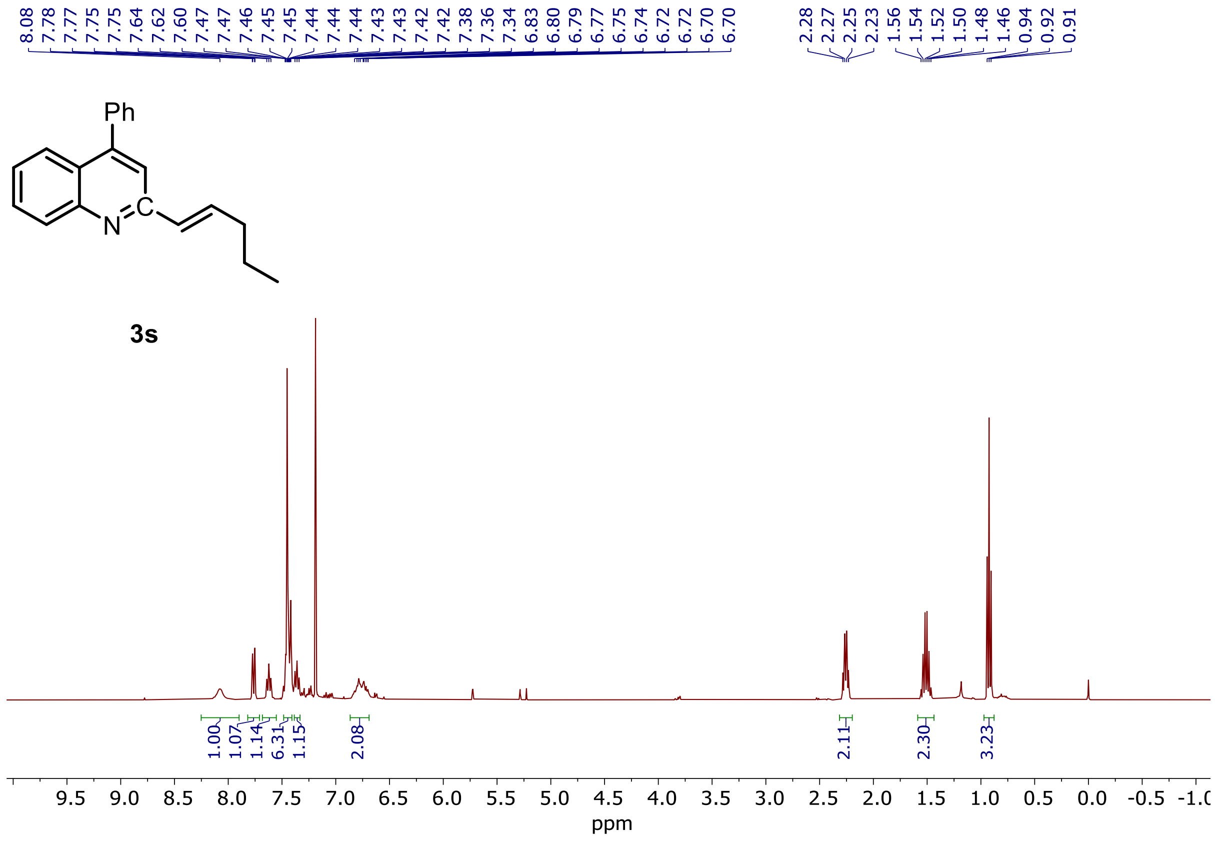

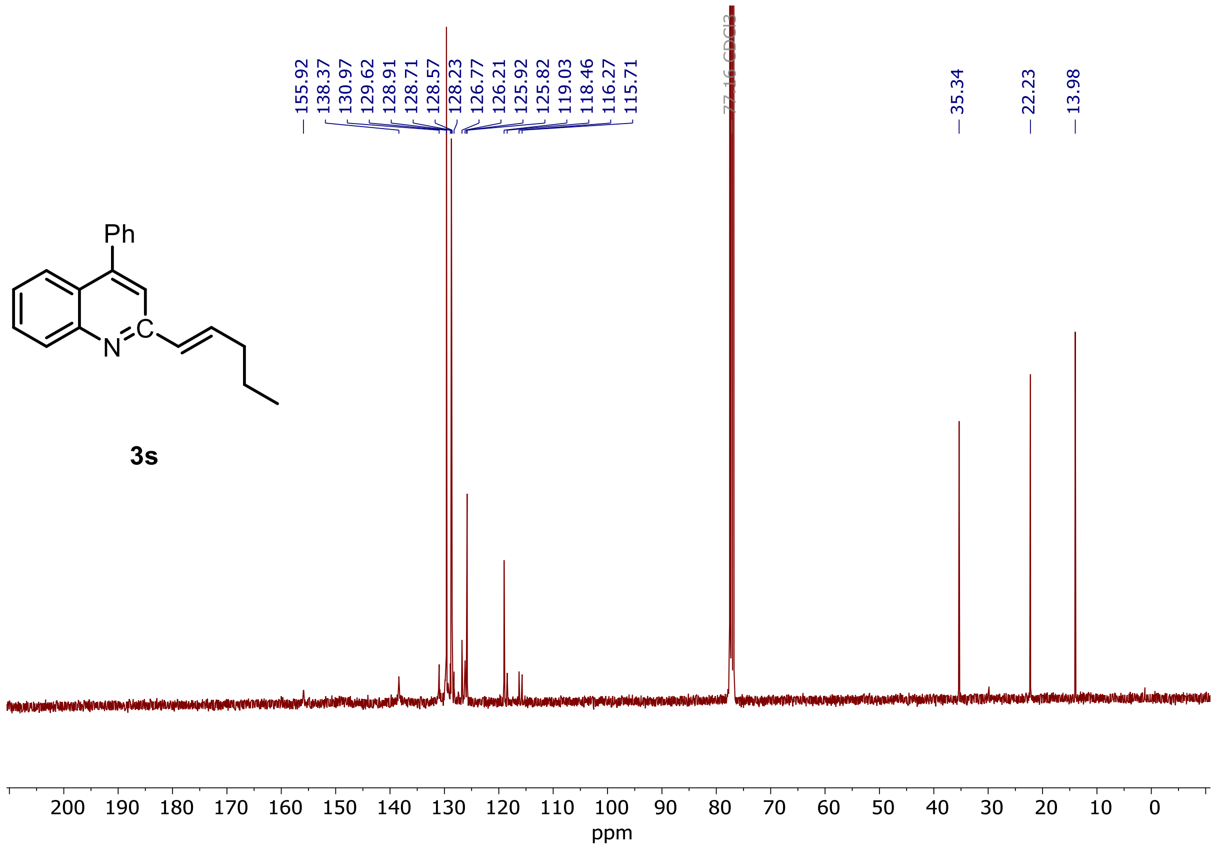

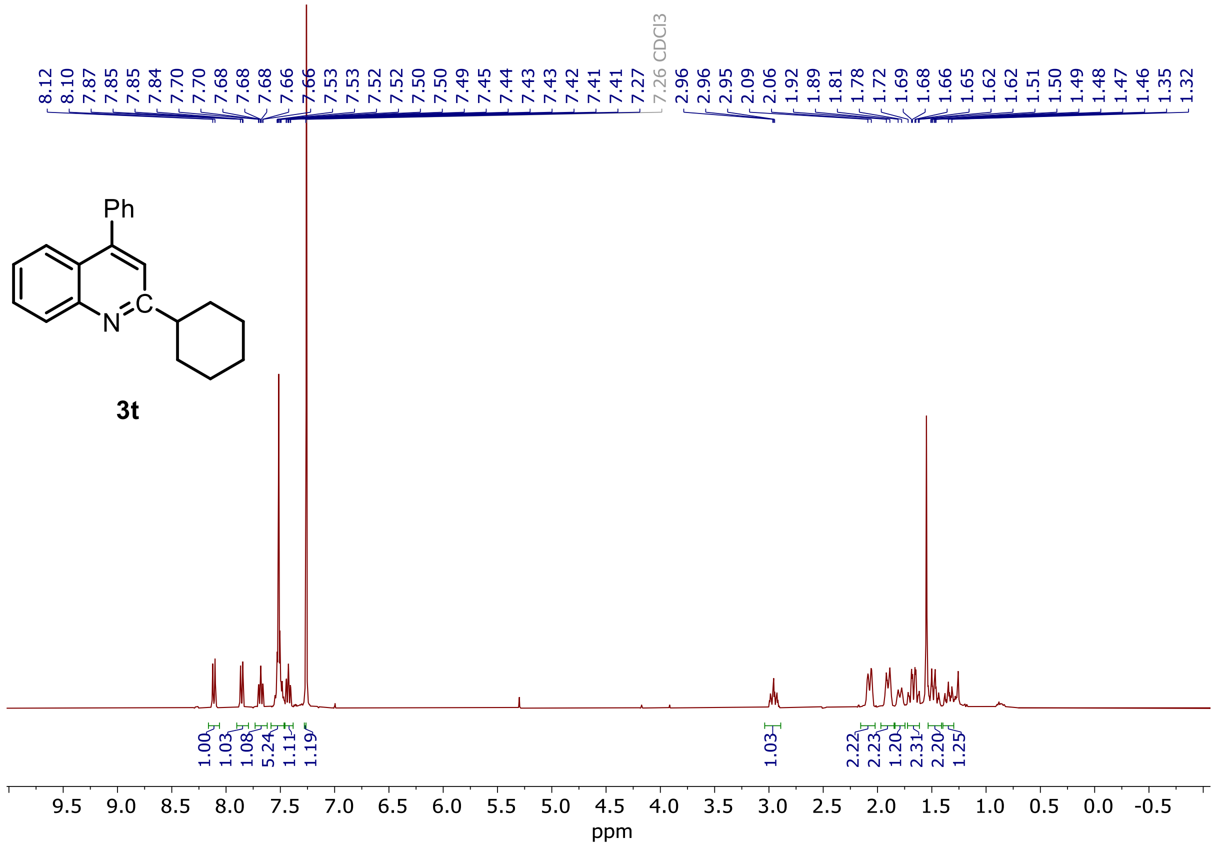

Ph
N
C
3t
ppm

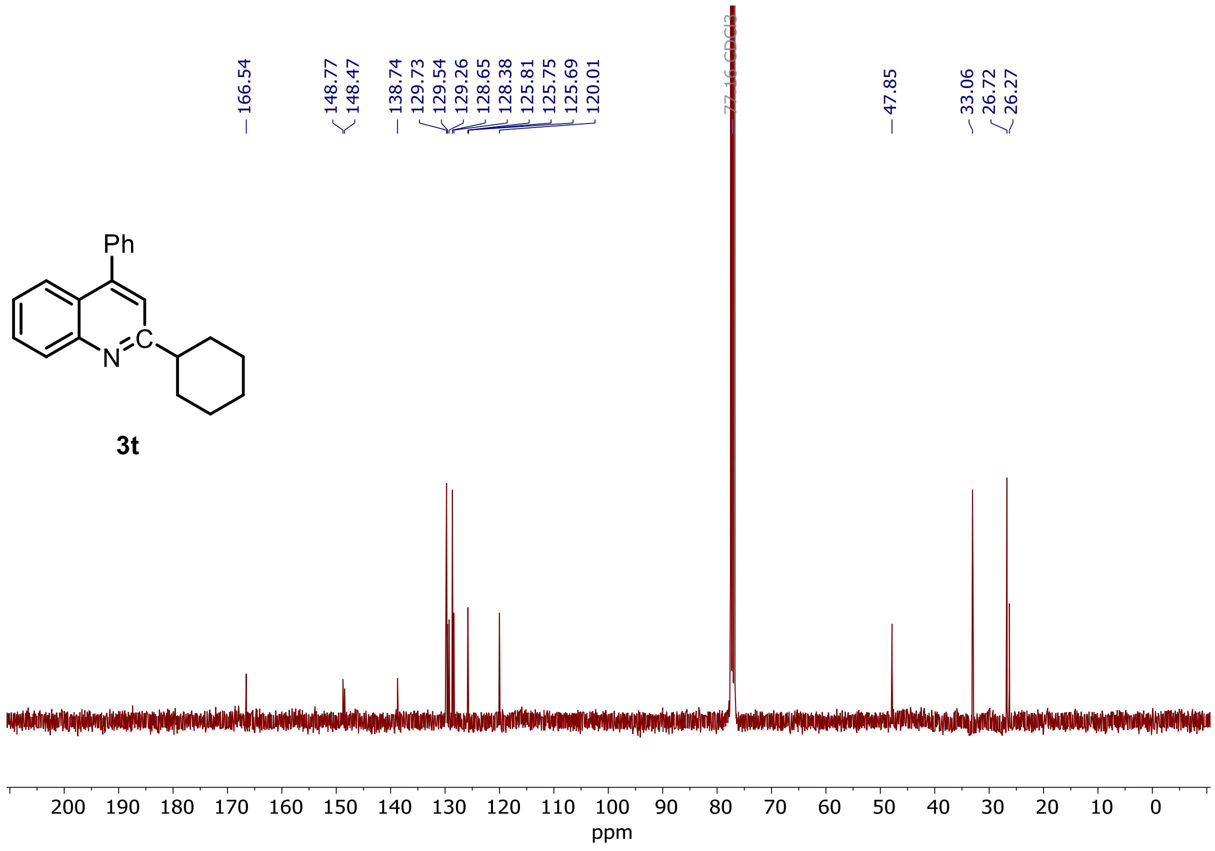

Ph
N
C
3t
ppm

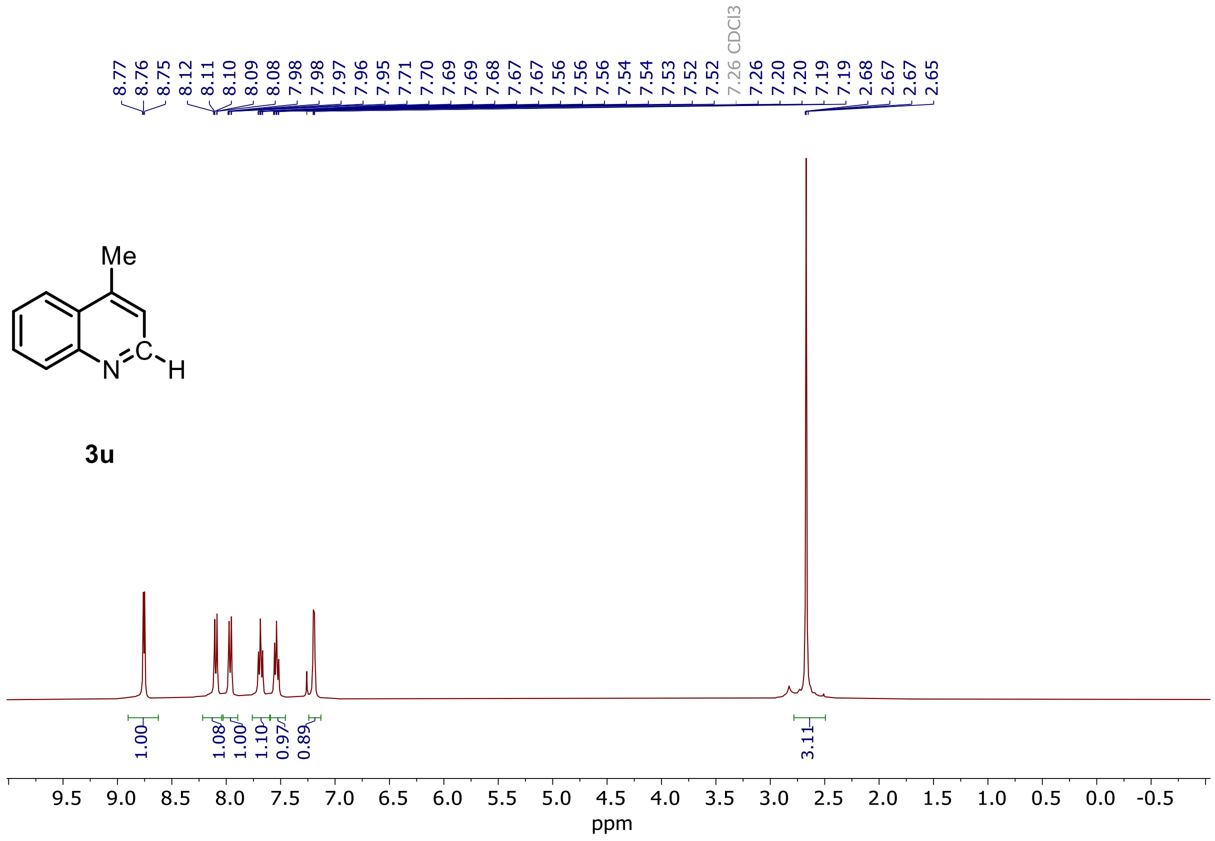
8.77
8.76
8.75
8.12
8.11
8.10
8.09
8.08
7.98
7.98
7.97
7.96
7.95
7.71
7.70
7.69
7.69
7.68
7.67
7.67
7.56
7.56
7.54
7.54
7.53
7.52
7.52
7.26 CDCl3
7.26
7.20
7.20
7.19
7.19
2.68
2.67
2.67
2.65
Me
N
C
H
3u
1.00
1.08
1.00
1.10
0.97
0.89
3.11
9.5 9.0 8.5 8.0 7.5 7.0 6.5 6.0 5.5 5.0 4.5 4.0 3.5 3.0 2.5 2.0 1.5 1.0 0.5 0.0 -0.5
ppm

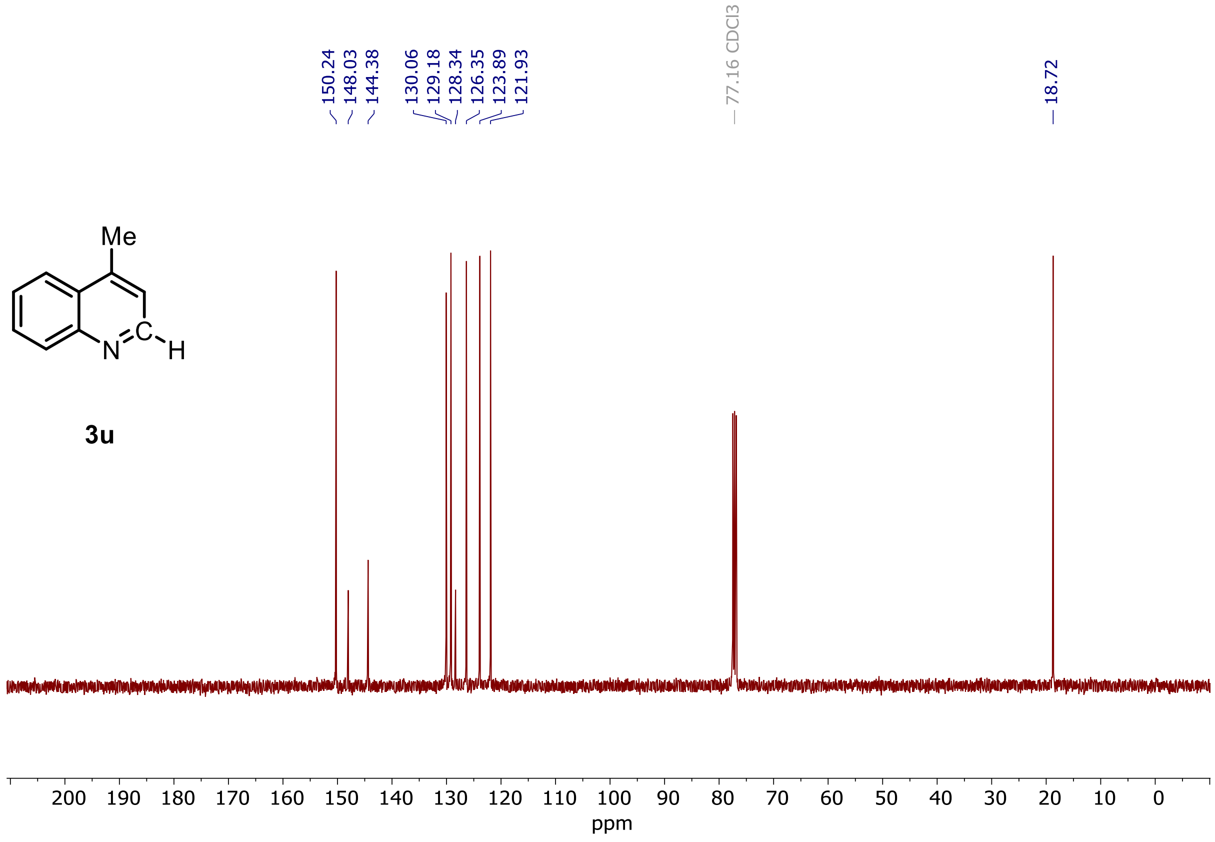
150.24
148.03
144.38
130.06
129.18
128.34
126.35
123.89
121.93
77.16 CDCl3
18.72
Me
N
C
H
3u
200 190 180 170 160 150 140 130 120 110 100 90 80 70 60 50 40 30 20 10 0
ppm

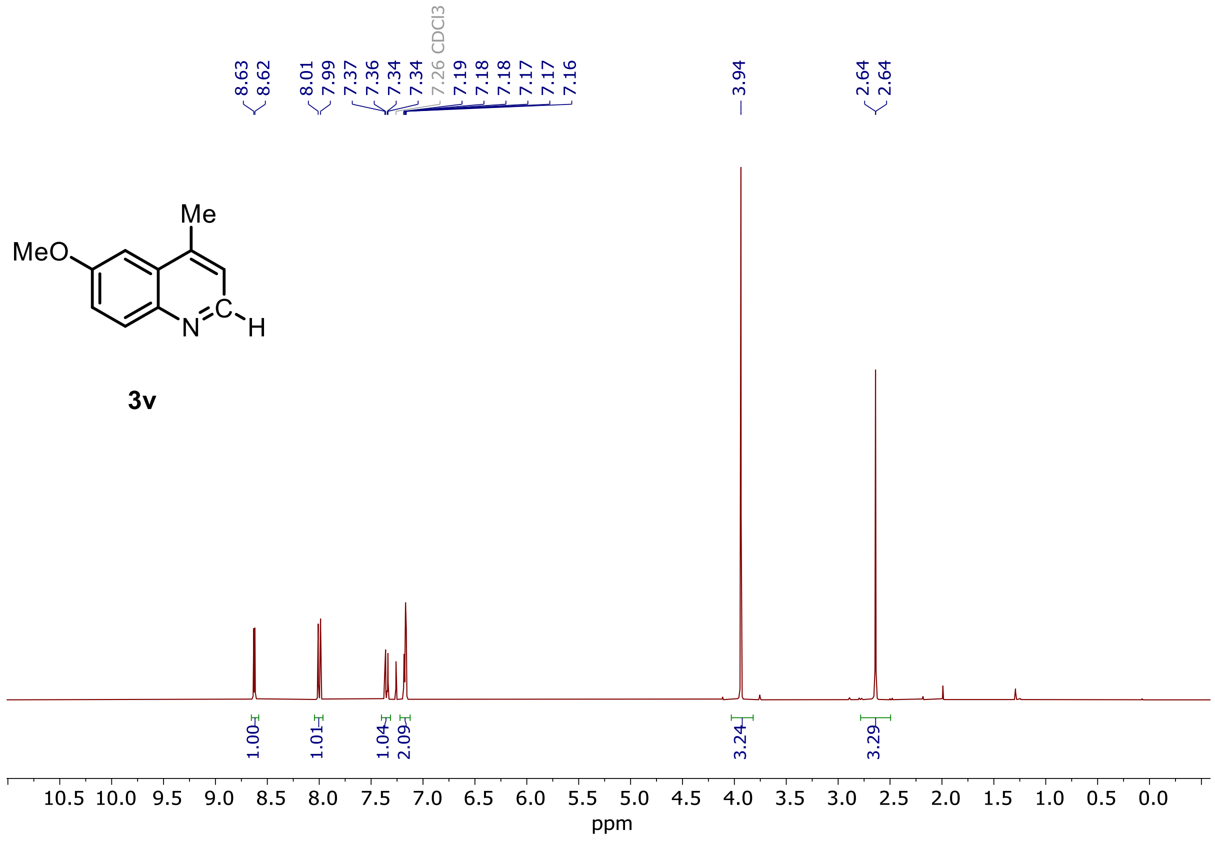
8.63
8.62
8.01
7.99
7.37
7.36
7.34
7.34
7.26 CDCl3
7.19
7.18
7.18
7.17
7.17
7.16
3.94
2.64
2.64
Me
MeO
N
C
H
3v
1.00
1.01
1.04
2.09
3.24
3.29
ppm

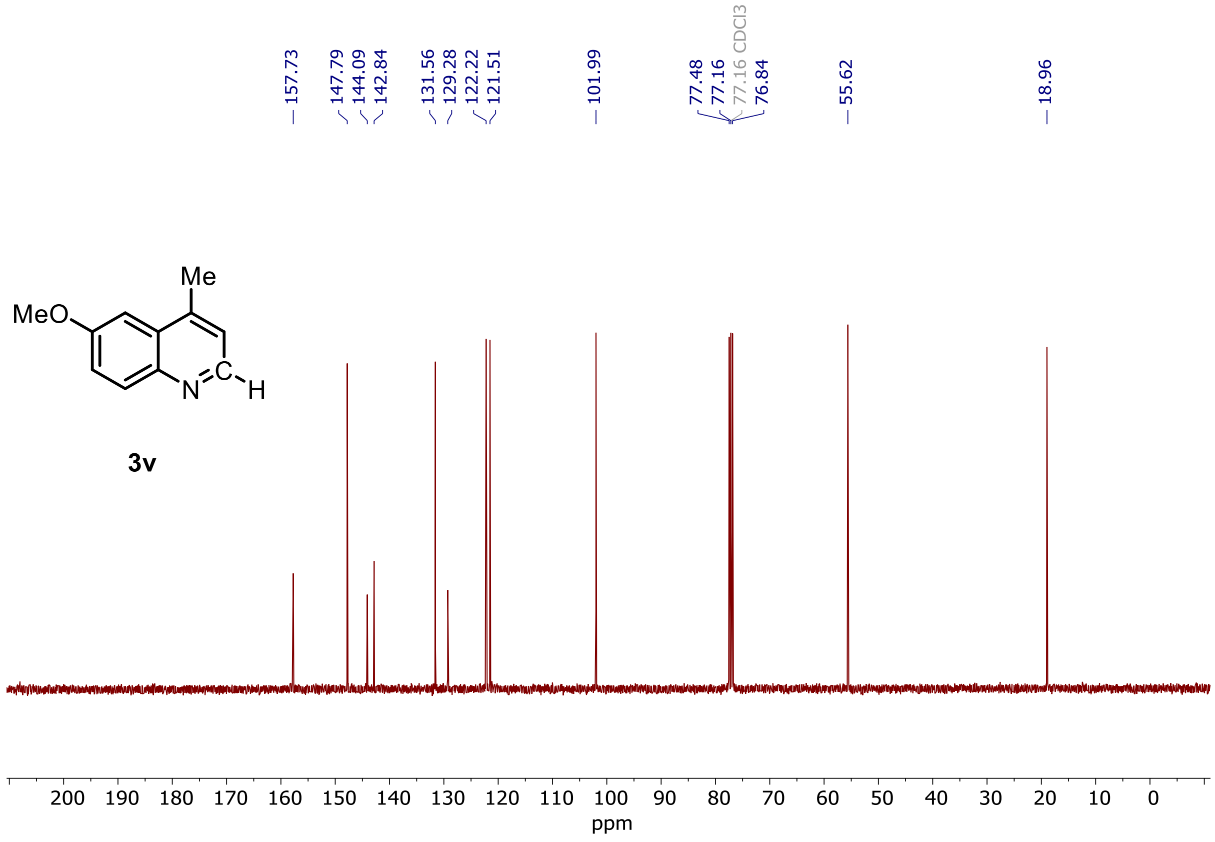
157.73
147.79
144.09
142.84
131.56
129.28
122.22
121.51
101.99
77.48
77.16
77.16 CDCl3
76.84
55.62
18.96
Me
MeO
N
C
H
3v
ppm

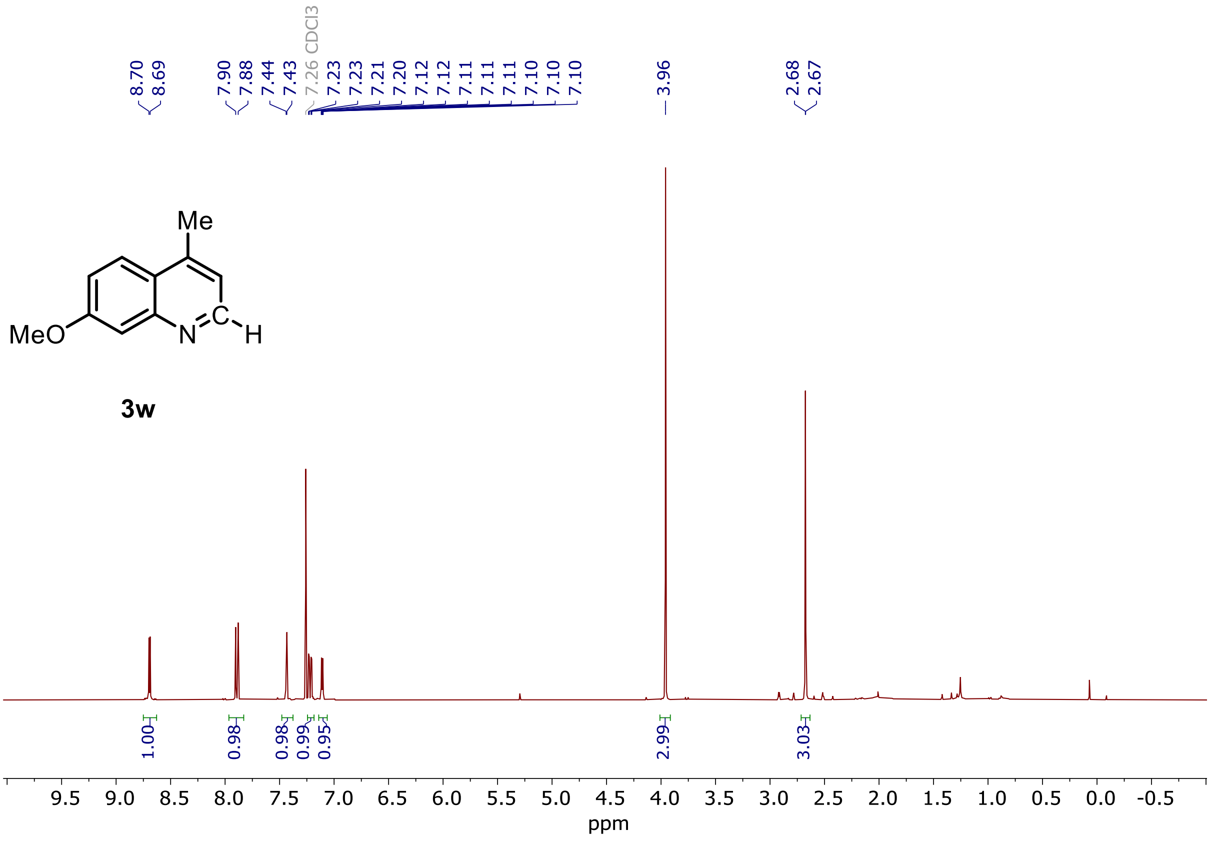
8.70
8.69
7.90
7.88
7.44
7.43
7.26 CDCl3
7.23
7.23
7.21
7.20
7.12
7.12
7.11
7.11
7.11
7.10
7.10
7.10
3.96
2.68
2.67
Me
MeO
N
C
H
3w
1.00
0.98
0.98
0.99
0.95
2.99
3.03
9.5 9.0 8.5 8.0 7.5 7.0 6.5 6.0 5.5 5.0 4.5 4.0 3.5 3.0 2.5 2.0 1.5 1.0 0.5 0.0 -0.5
ppm

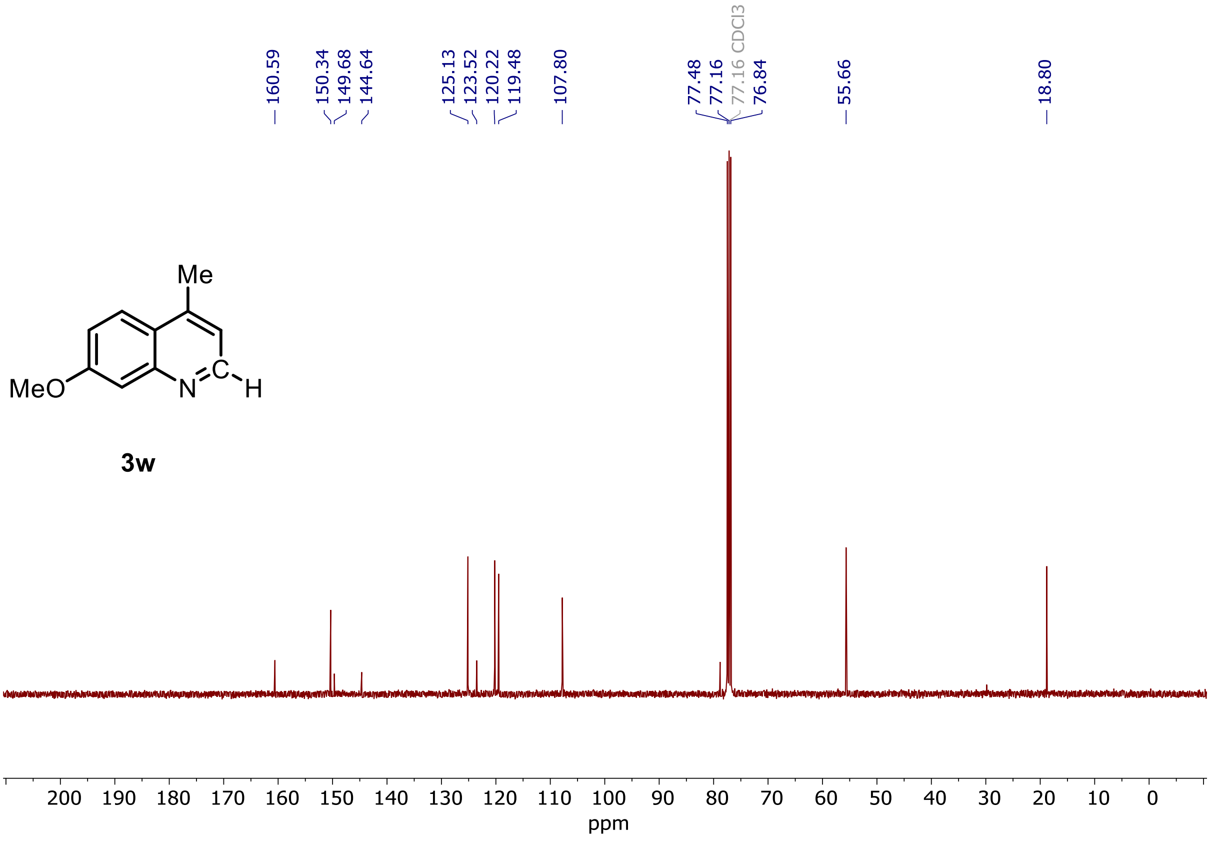
160.59
150.34
149.68
144.64
125.13
123.52
120.22
119.48
107.80
77.48
77.16
77.16 CDCl3
76.84
55.66
18.80
Me
MeO
N
C
H
3w
200 190 180 170 160 150 140 130 120 110 100 90 80 70 60 50 40 30 20 10 0
ppm

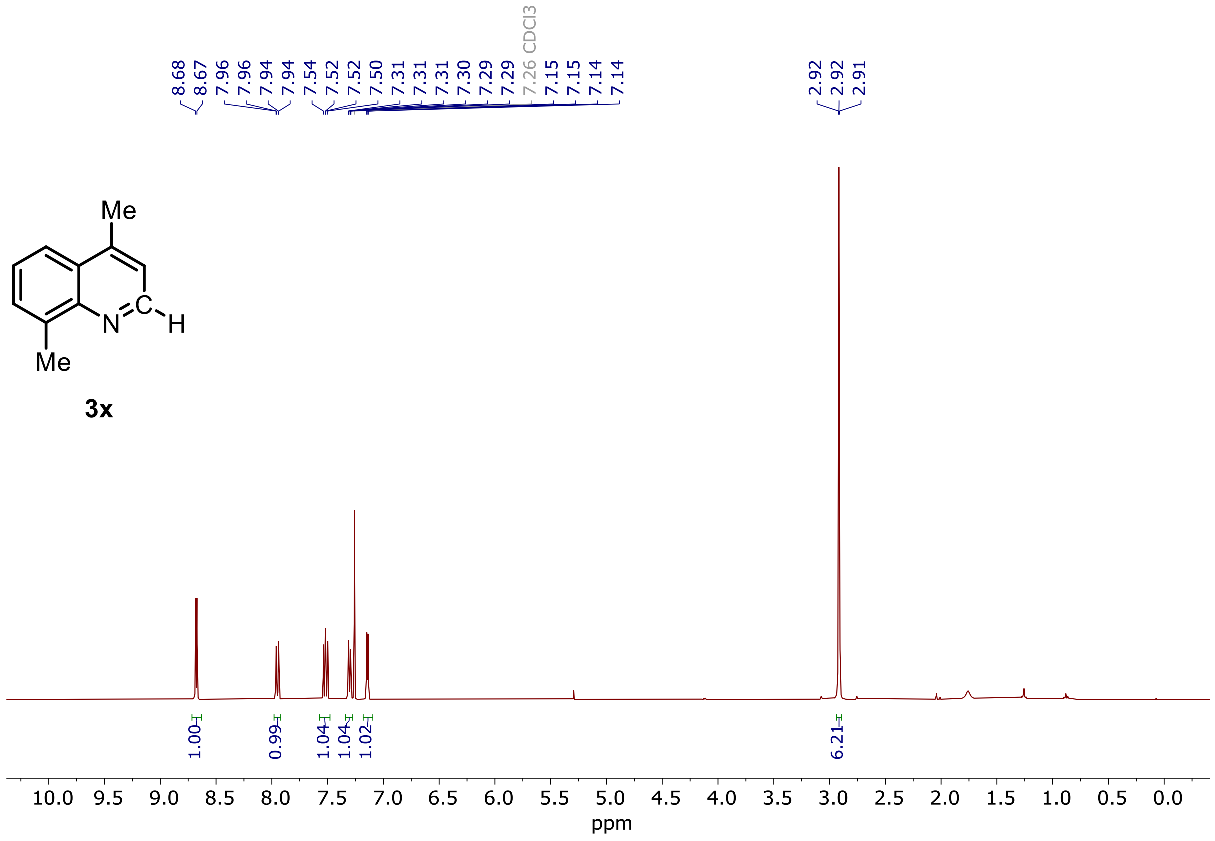
8.68
8.67
7.96
7.96
7.94
7.94
7.54
7.52
7.52
7.50
7.31
7.31
7.31
7.30
7.29
7.29
7.26 CDCl3
7.15
7.15
7.14
7.14
2.92
2.92
2.91
Me
N
C
H
Me
3x
1.00
0.99
1.04
1.04
1.02
6.21
10.0 9.5 9.0 8.5 8.0 7.5 7.0 6.5 6.0 5.5 5.0 4.5 4.0 3.5 3.0 2.5 2.0 1.5 1.0 0.5 0.0
ppm

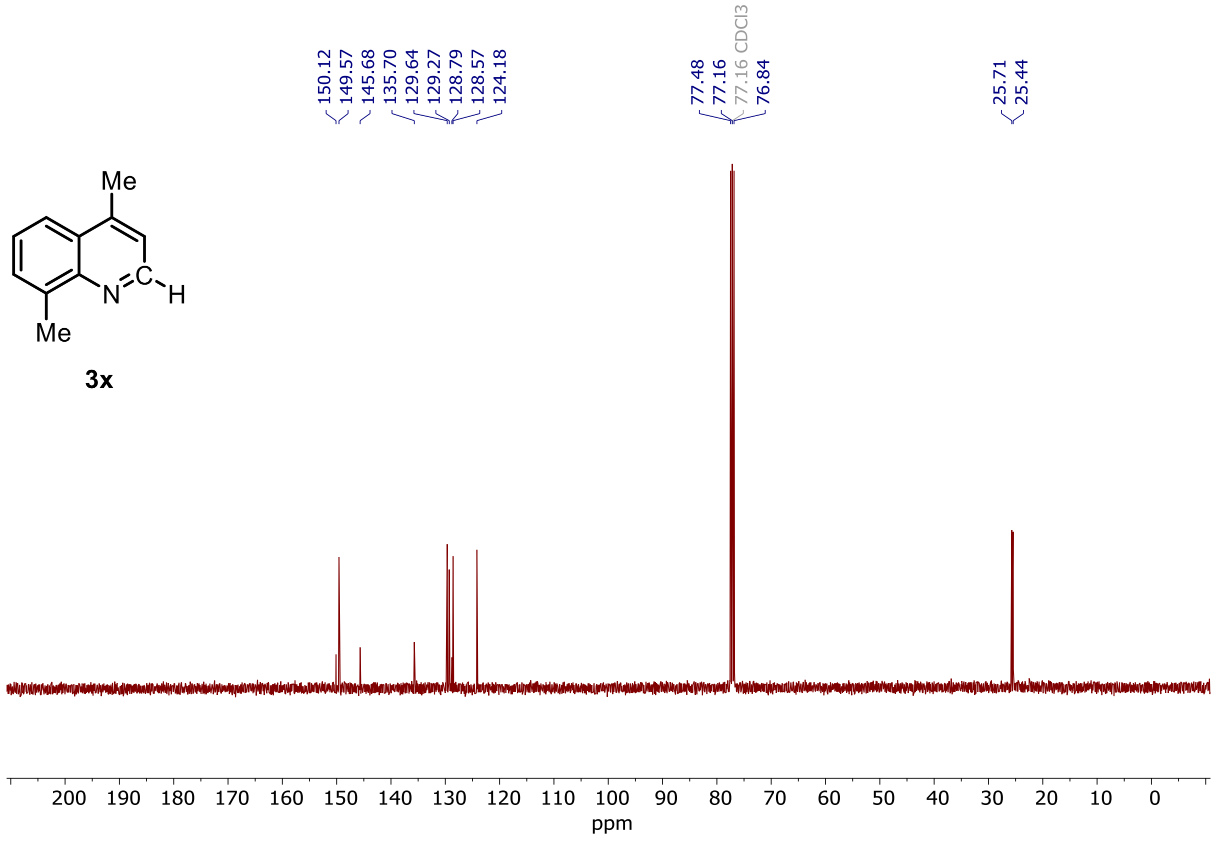
150.12
149.57
145.68
135.70
129.64
129.27
128.79
128.57
124.18
77.48
77.16
77.16 CDCl3
76.84
25.71
25.44
Me
N
C
H
Me
3x
200 190 180 170 160 150 140 130 120 110 100 90 80 70 60 50 40 30 20 10 0
ppm

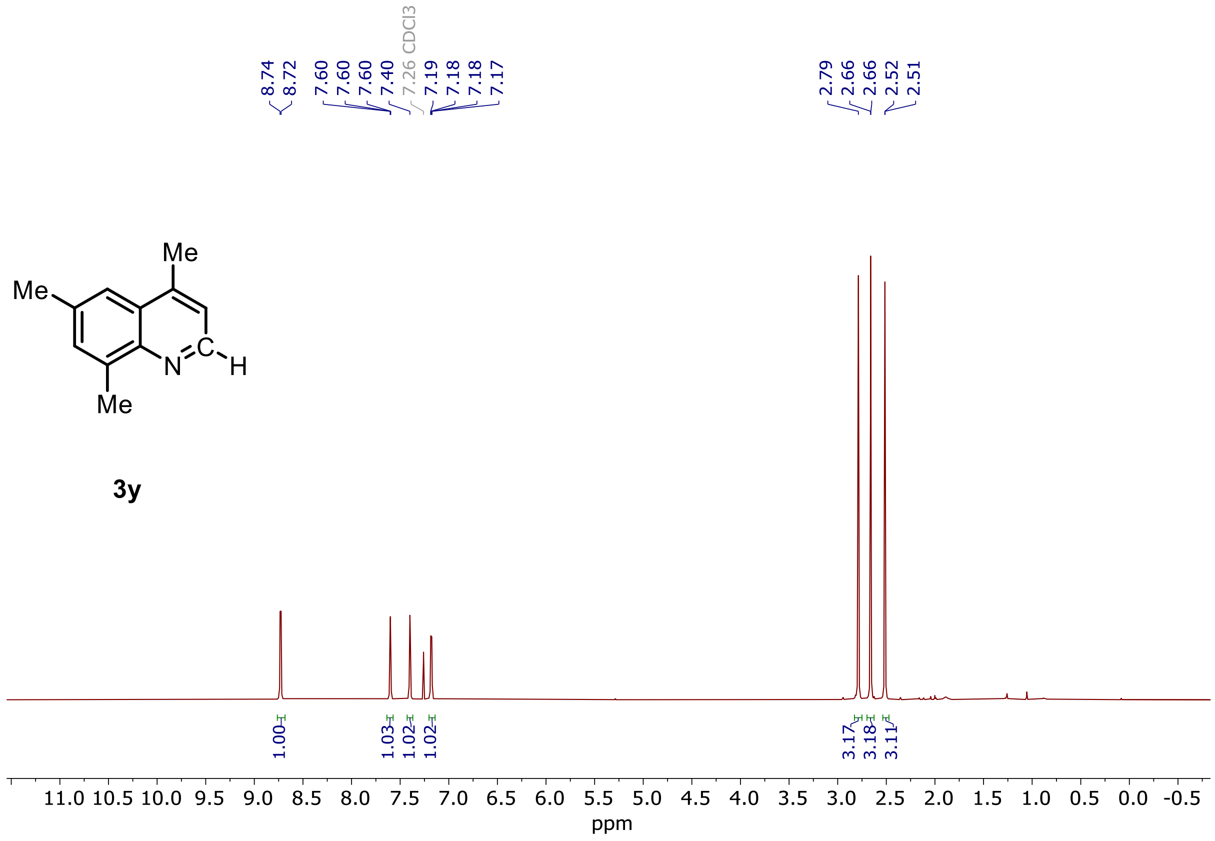

8.74
8.72
7.60
7.60
7.60
7.40
7.26 CDCl3
7.19
7.18
7.18
7.17
2.79
2.66
2.66
2.52
2.51
Me
Me
N
C
H
Me
3y
1.00
1.03
1.02
1.02
3.17
3.18
3.11
11.0 10.5 10.0 9.5 9.0 8.5 8.0 7.5 7.0 6.5 6.0 5.5 5.0 4.5 4.0 3.5 3.0 2.5 2.0 1.5 1.0 0.5 0.0 -0.5
ppm

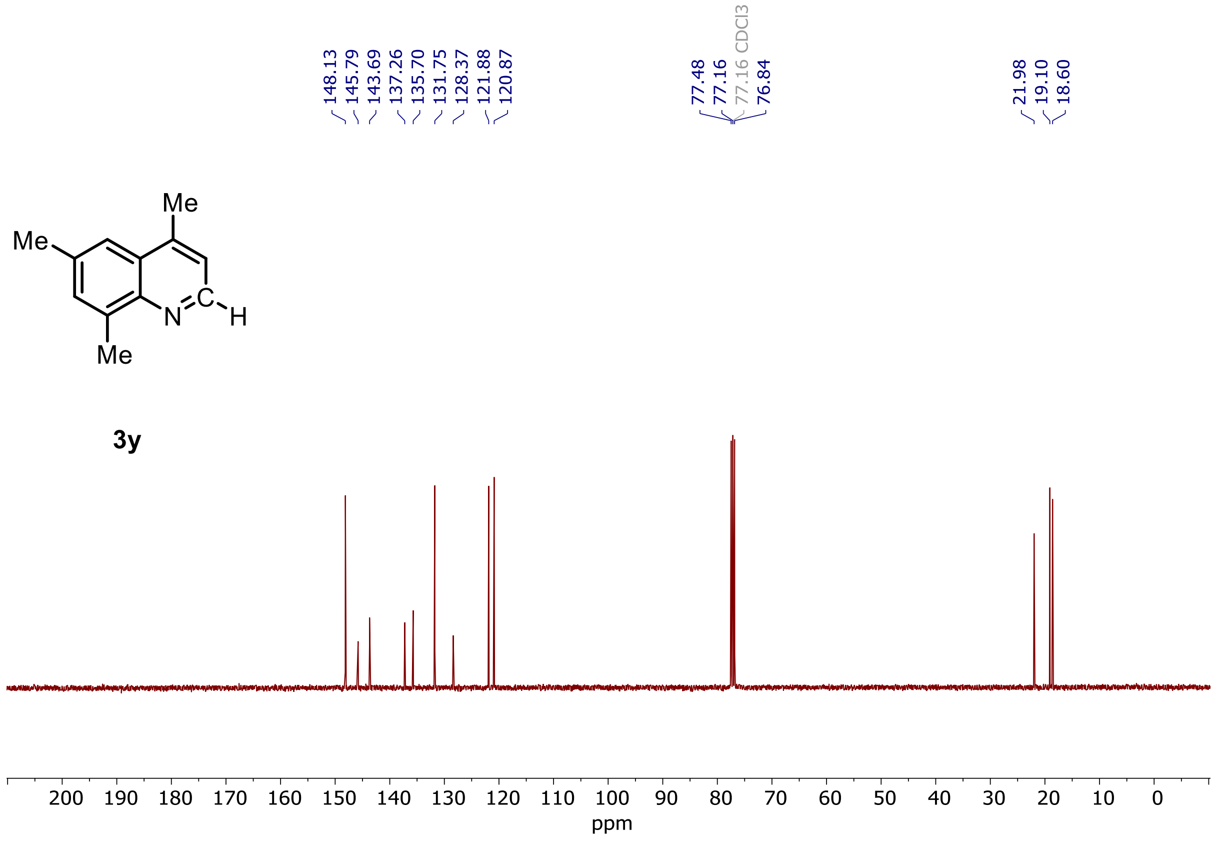

148.13
145.79
143.69
137.26
135.70
131.75
128.37
121.88
120.87
77.48
77.16
77.16 CDCl3
76.84
21.98
19.10
18.60
Me
Me
N
C
H
Me
3y
200 190 180 170 160 150 140 130 120 110 100 90 80 70 60 50 40 30 20 10 0
ppm

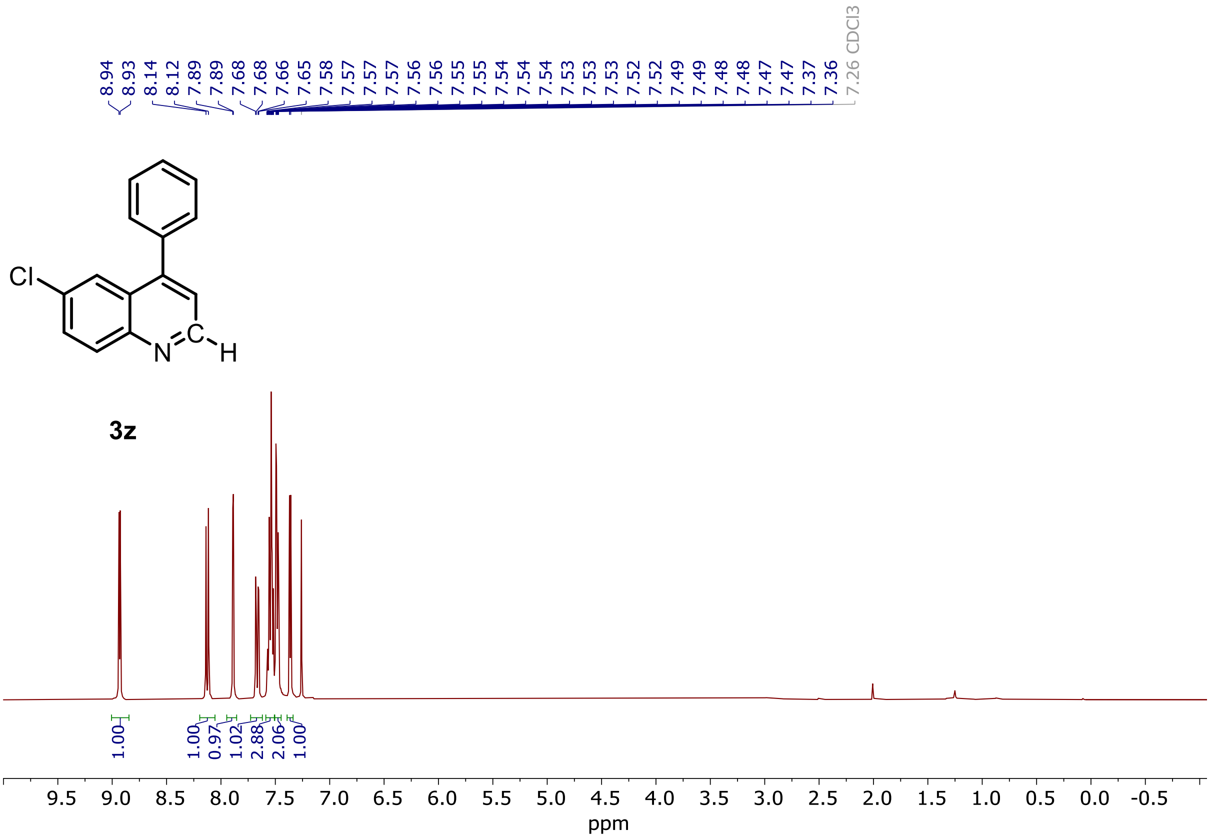
8.94
8.93
8.14
8.12
7.89
7.89
7.68
7.68
7.66
7.65
7.58
7.57
7.57
7.57
7.56
7.56
7.55
7.55
7.54
7.54
7.54
7.53
7.53
7.53
7.52
7.52
7.49
7.49
7.48
7.48
7.47
7.47
7.37
7.36
7.26 CDCl3
Cl
N
C
H
3z
1.00
1.00
0.97
1.02
2.88
2.06
1.00
9.5 9.0 8.5 8.0 7.5 7.0 6.5 6.0 5.5 5.0 4.5 4.0 3.5 3.0 2.5 2.0 1.5 1.0 0.5 0.0 -0.5
ppm

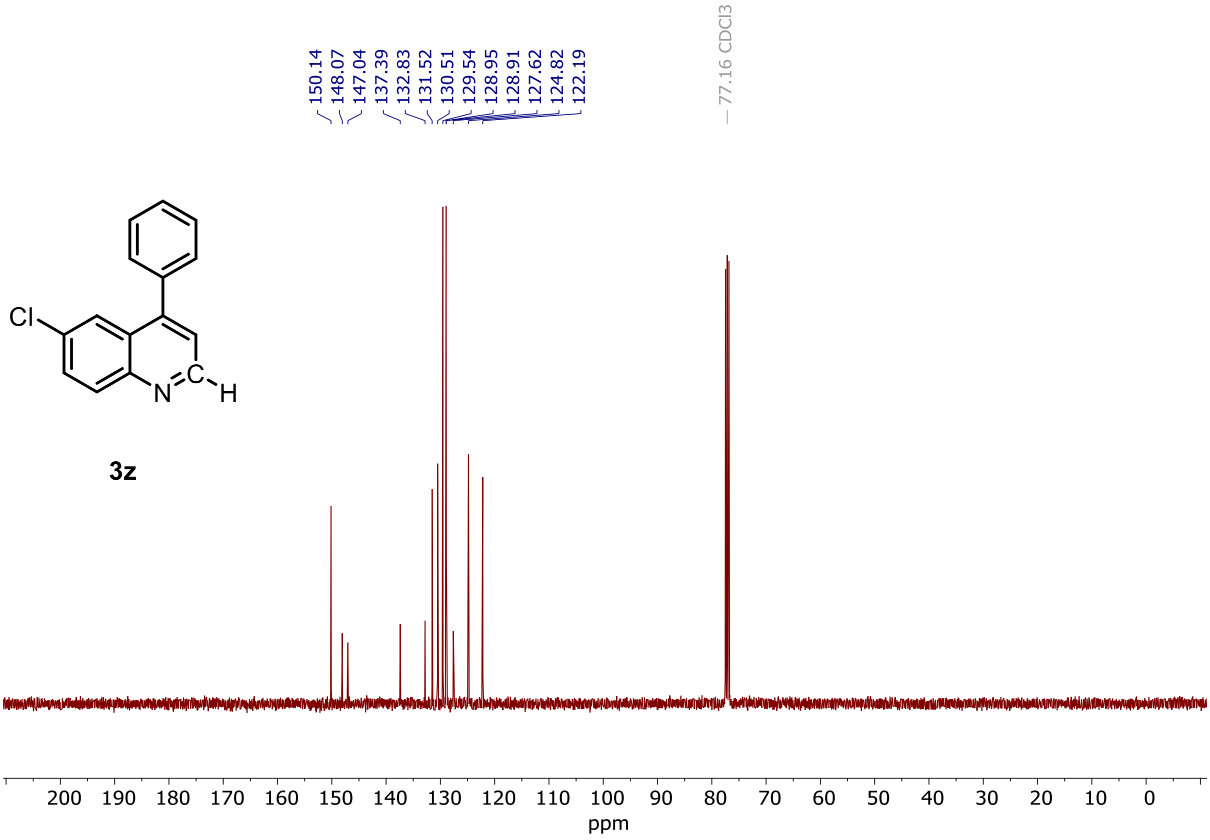
150.14
148.07
147.04
137.39
132.83
131.52
130.51
129.54
128.95
128.91
127.62
124.82
122.19
77.16 CDCl3
Cl
N
C
H
3z
200 190 180 170 160 150 140 130 120 110 100 90 80 70 60 50 40 30 20 10 0
ppm

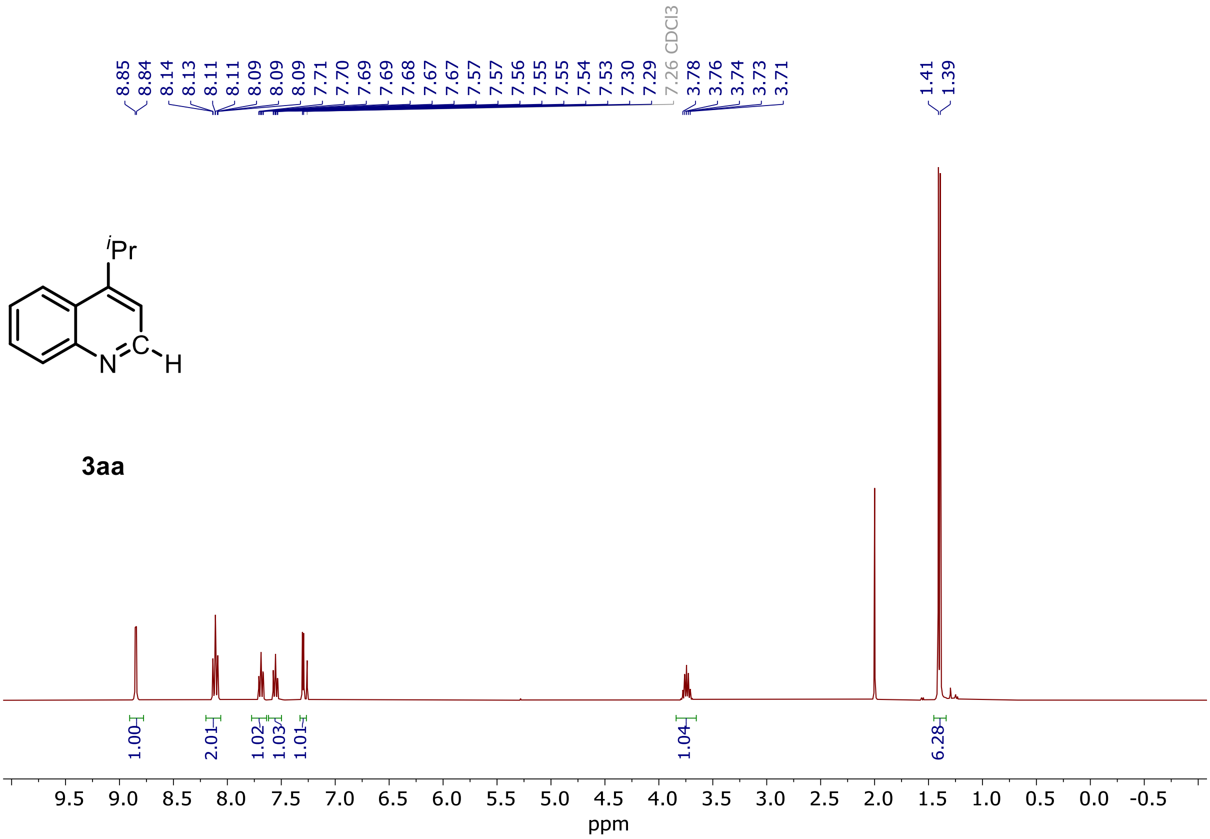

8.85
8.84
8.14
8.13
8.11
8.11
8.09
8.09
8.09
7.71
7.70
7.69
7.69
7.68
7.67
7.67
7.57
7.57
7.56
7.55
7.55
7.54
7.53
7.30
7.29
7.26 CDCl3
3.78
3.76
3.74
3.73
3.71
1.41
1.39
^{i}Pr
N
C
H
3aa
1.00
2.01
1.02
1.03
1.01
1.04
6.28
9.5 9.0 8.5 8.0 7.5 7.0 6.5 6.0 5.5 5.0 4.5 4.0 3.5 3.0 2.5 2.0 1.5 1.0 0.5 0.0 -0.5
ppm

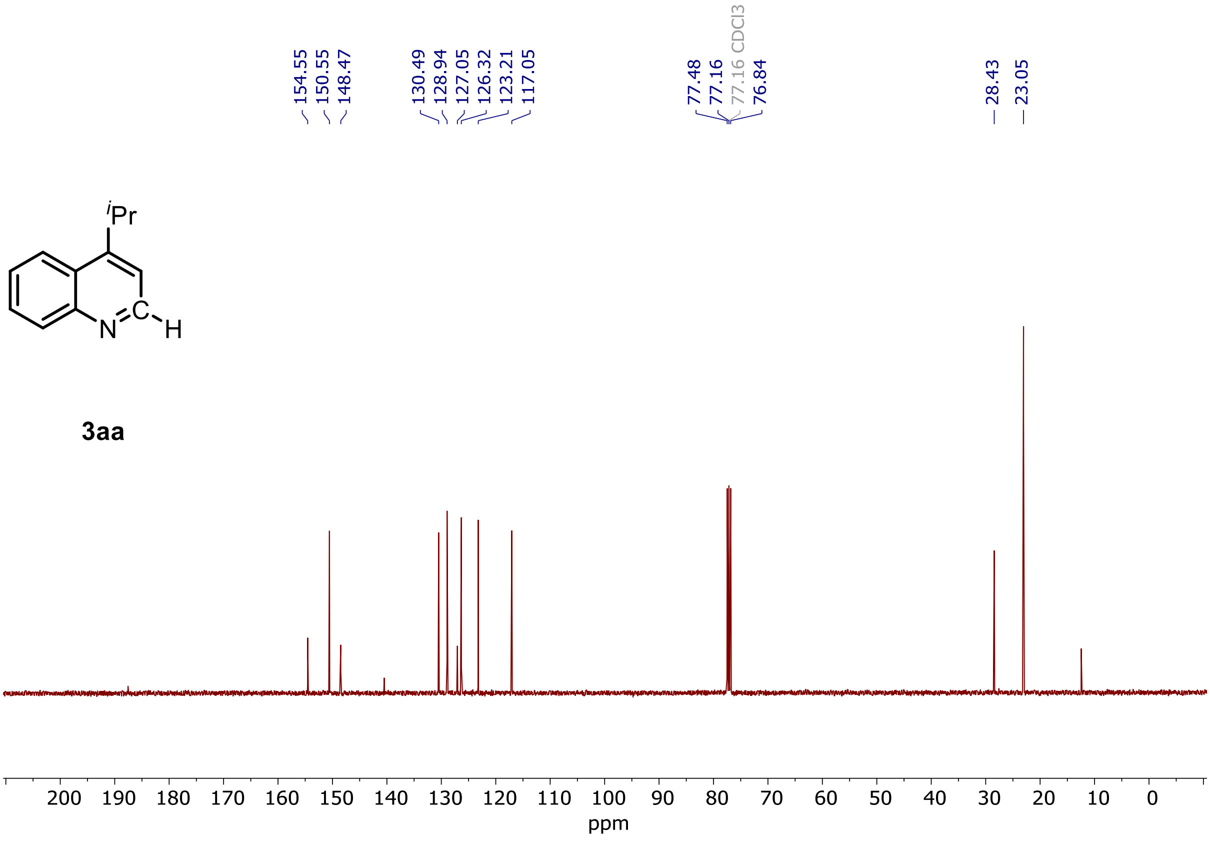

154.55
150.55
148.47
130.49
128.94
127.05
126.32
123.21
117.05
77.48
77.16
77.16 CDCl3
76.84
28.43
23.05
^{i}Pr
N
C
H
3aa
200 190 180 170 160 150 140 130 120 110 100 90 80 70 60 50 40 30 20 10 0
ppm

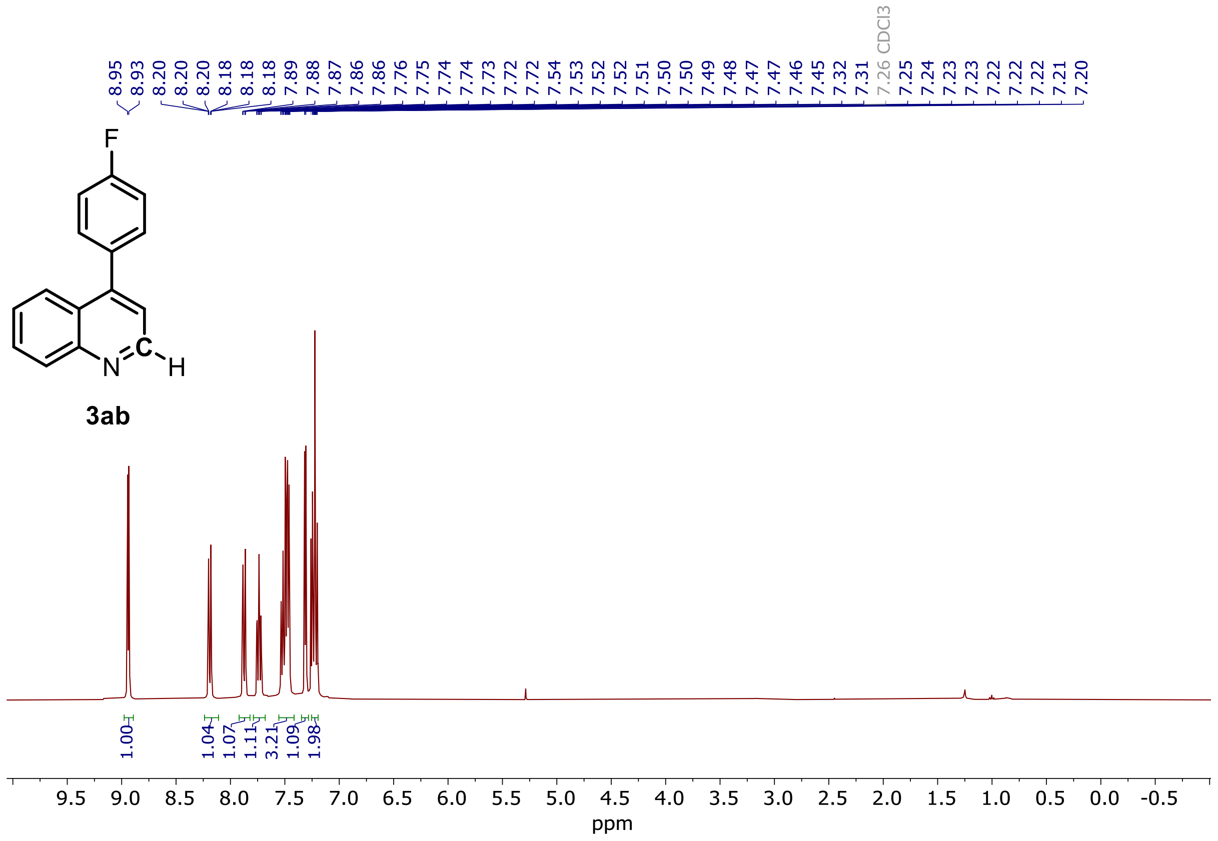
8.95
8.93
8.20
8.20
8.20
8.18
8.18
8.18
7.89
7.88
7.87
7.86
7.76
7.75
7.74
7.74
7.73
7.72
7.72
7.54
7.53
7.52
7.52
7.51
7.50
7.49
7.48
7.47
7.47
7.46
7.45
7.32
7.31
7.26 CDCl3
7.25
7.24
7.23
7.23
7.22
7.22
7.22
7.21
7.20
F
N
C
H
3ab
1.00
1.04
1.07
1.11
3.21
1.09
1.98
9.5 9.0 8.5 8.0 7.5 7.0 6.5 6.0 5.5 5.0 4.5 4.0 3.5 3.0 2.5 2.0 1.5 1.0 0.5 0.0 -0.5
ppm

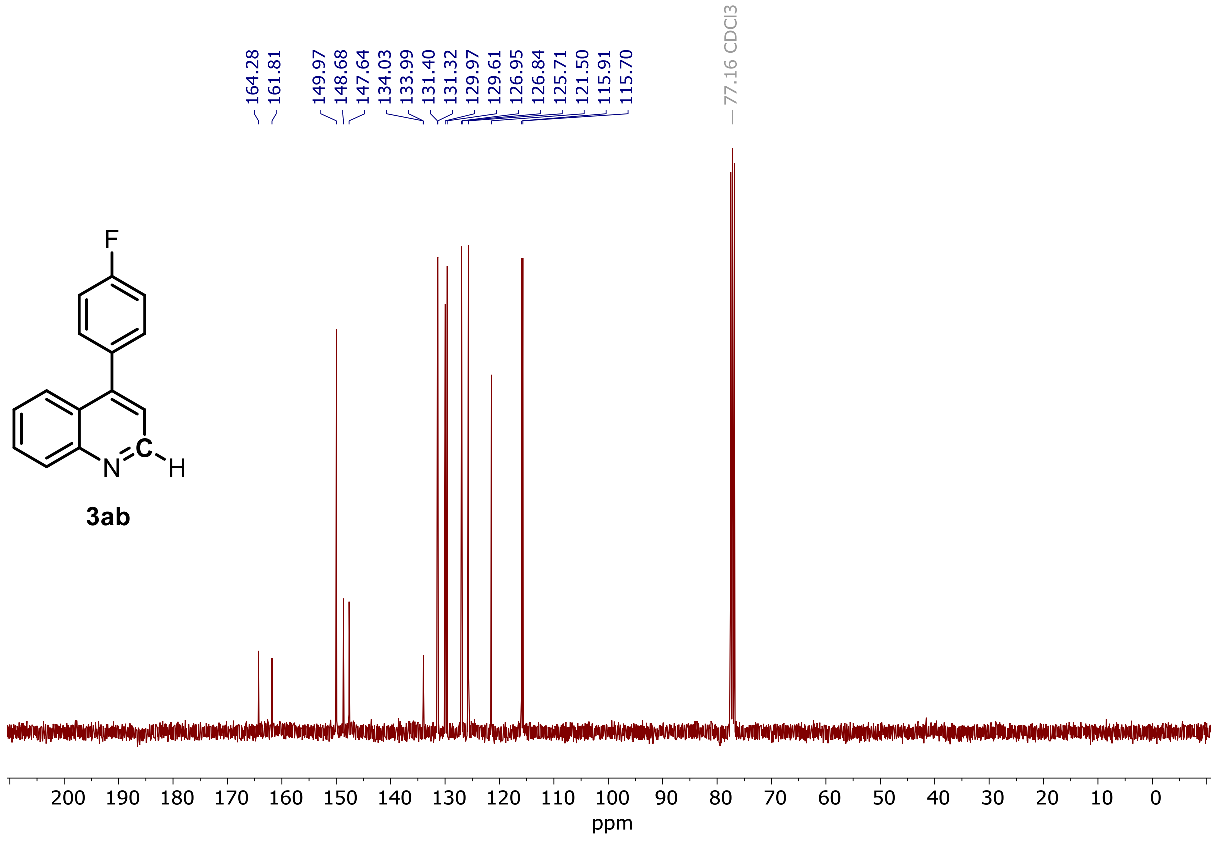
164.28
161.81
149.97
148.68
147.64
134.03
133.99
131.40
131.32
129.97
129.61
126.95
126.84
125.71
121.50
115.91
115.70
77.16 CDCl3
F
N
C
H
3ab
200 190 180 170 160 150 140 130 120 110 100 90 80 70 60 50 40 30 20 10 0
ppm

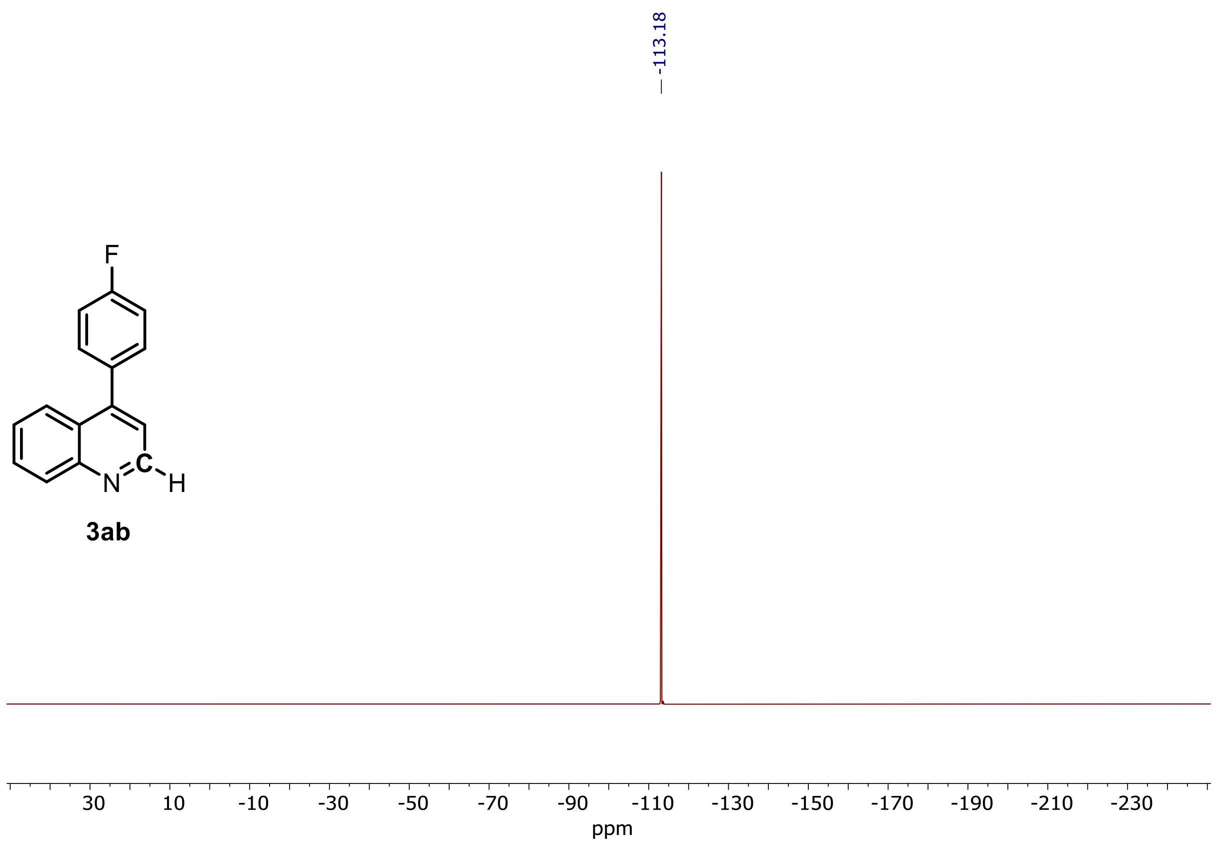
-113.18
F
N
C
H
3ab
30 10 -10 -30 -50 -70 -90 -110 -130 -150 -170 -190 -210 -230
ppm

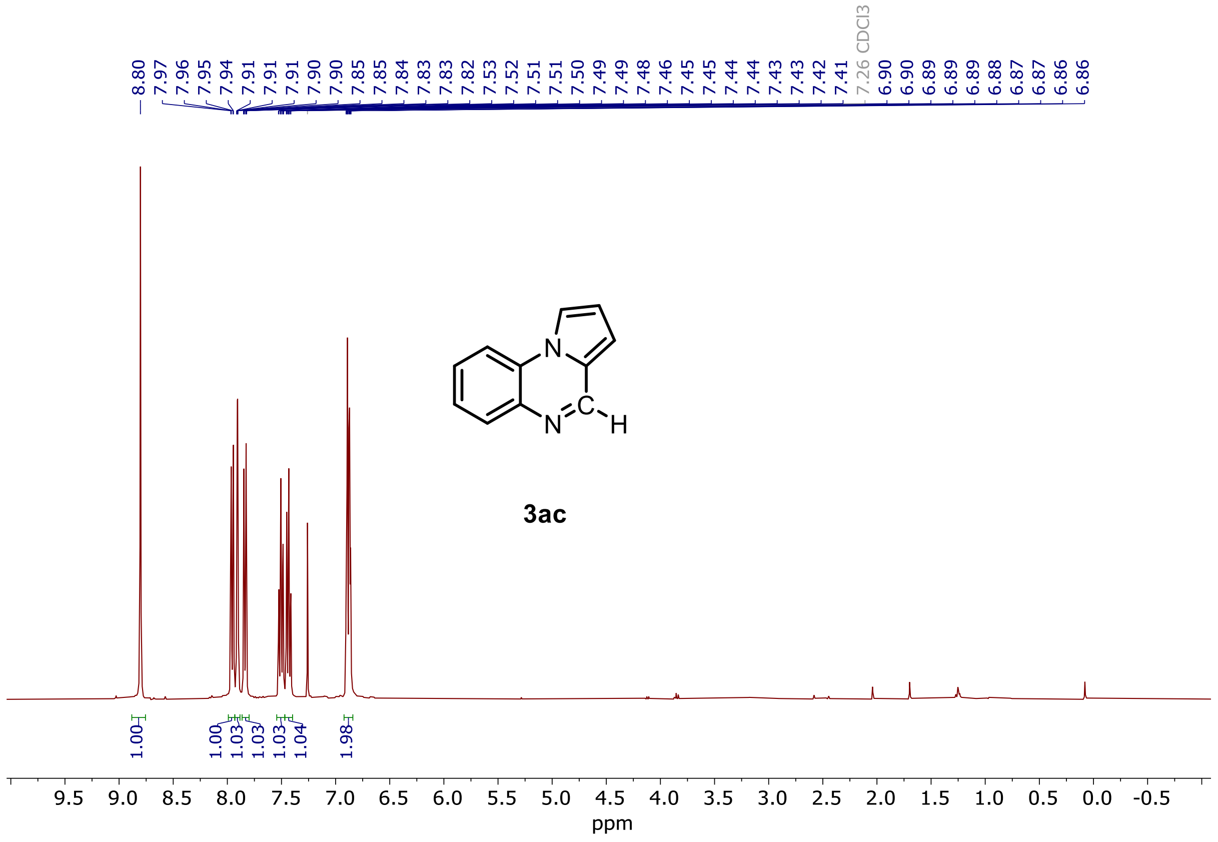
8.80 7.97 7.96 7.95 7.94 7.91 7.91 7.91 7.90 7.90 7.85 7.85 7.84 7.83 7.83 7.82 7.53 7.52 7.51 7.51 7.50 7.49 7.49 7.48 7.46 7.45 7.45 7.44 7.44 7.43 7.43 7.42 7.41 7.26 CDCl3 6.90 6.90 6.89 6.89 6.88 6.87 6.87 6.86 6.86
N
N
C
H
3ac
1.00 1.00 1.03 1.03 1.03 1.04 1.98
9.5 9.0 8.5 8.0 7.5 7.0 6.5 6.0 5.5 5.0 4.5 4.0 3.5 3.0 2.5 2.0 1.5 1.0 0.5 0.0 -0.5
ppm

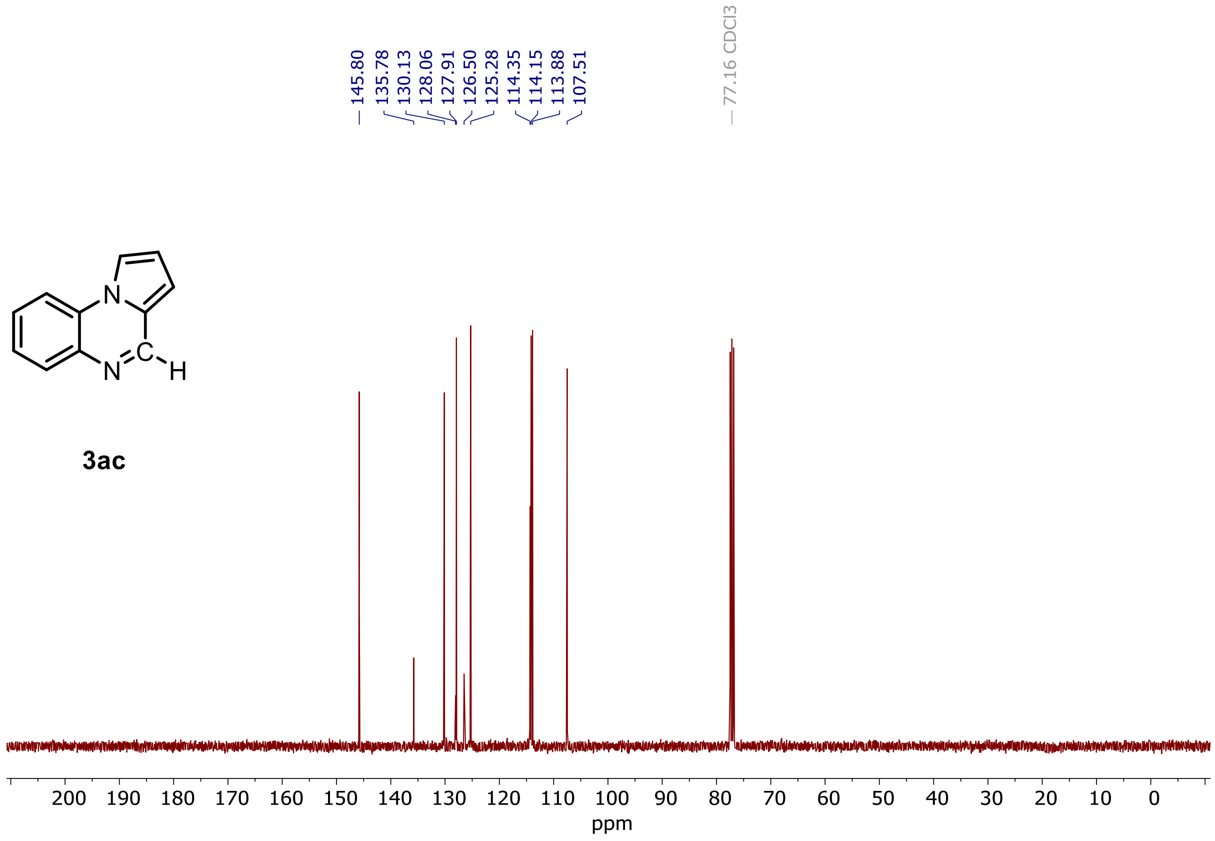
145.80
135.78
130.13
128.06
127.91
126.50
125.28
114.35
114.15
113.88
107.51
77.16 CDCl3
N
N
C
H
3ac
200 190 180 170 160 150 140 130 120 110 100 90 80 70 60 50 40 30 20 10 0
ppm

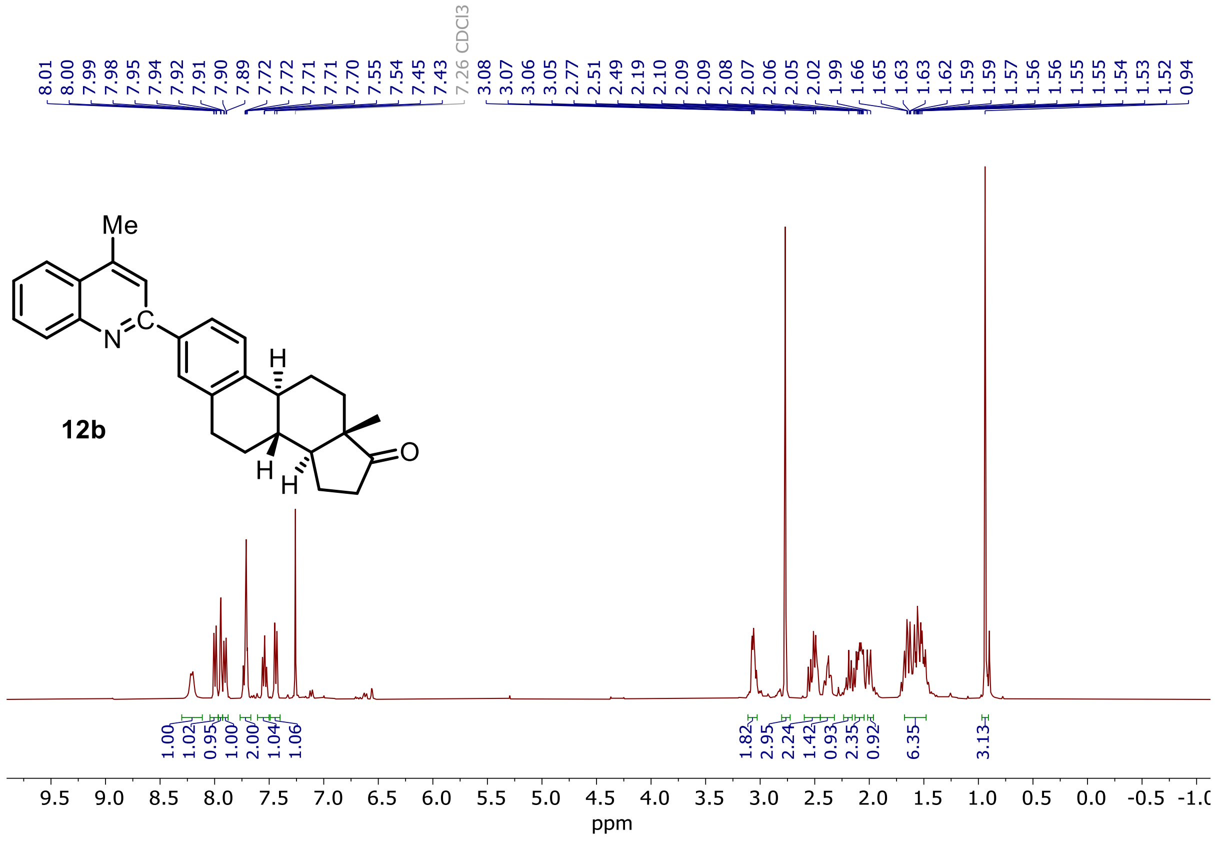
8.01
8.00
7.99
7.98
7.95
7.94
7.92
7.91
7.90
7.89
7.72
7.72
7.71
7.70
7.55
7.54
7.45
7.43
7.26 CDCl3
3.08
3.07
3.06
3.05
2.77
2.51
2.49
2.19
2.10
2.09
2.09
2.08
2.07
2.06
2.05
2.02
1.99
1.66
1.65
1.63
1.63
1.62
1.59
1.59
1.57
1.56
1.56
1.55
1.55
1.54
1.53
1.52
0.94
Me
N
C
H
H
H
O
12b
1.00
1.02
0.95
1.00
2.00
1.04
1.06
1.82
2.95
2.24
1.42
0.93
2.35
0.92
6.35
3.13
9.5 9.0 8.5 8.0 7.5 7.0 6.5 6.0 5.5 5.0 4.5 4.0 3.5 3.0 2.5 2.0 1.5 1.0 0.5 0.0 -0.5 -1.0
ppm

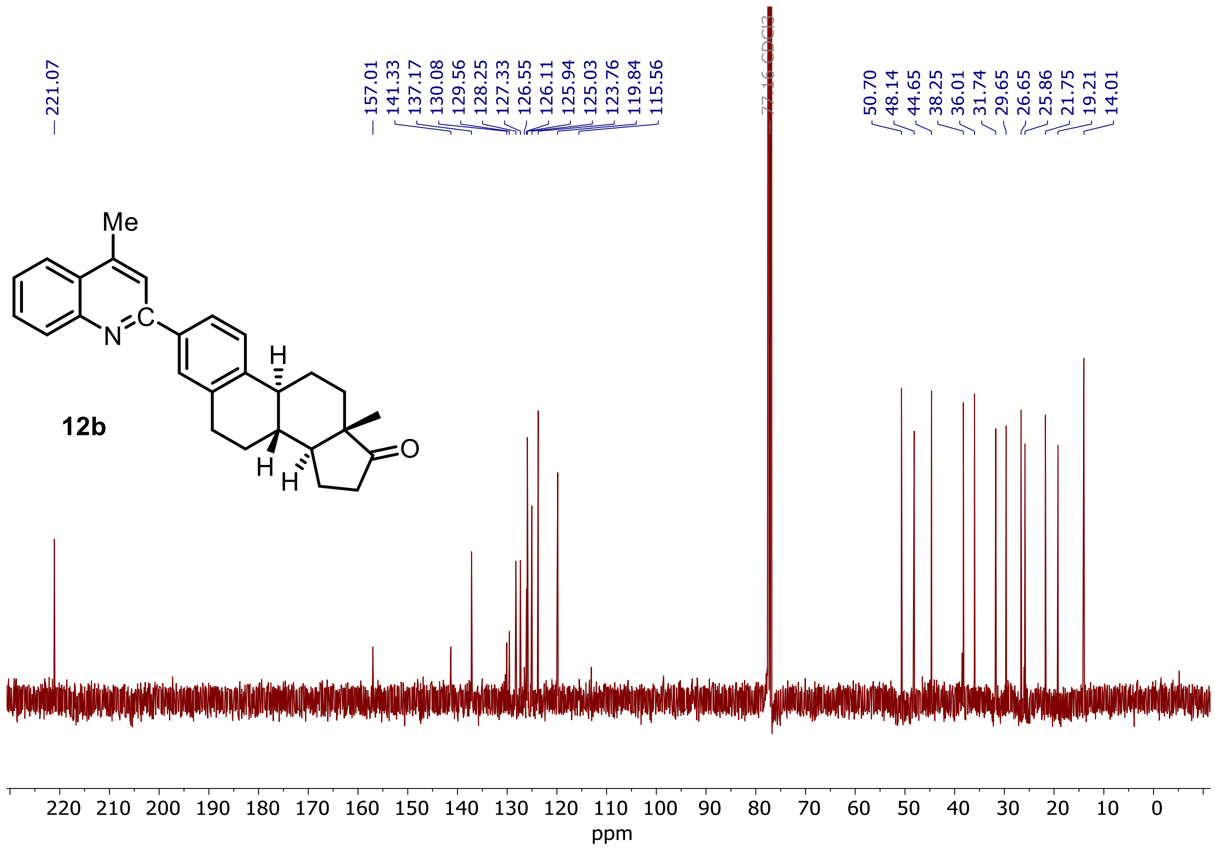

221.07
157.01
141.33
137.17
130.08
129.56
128.25
127.33
126.55
126.11
125.94
125.03
123.76
119.84
115.56
77.16 CDCl3
50.70
48.14
44.65
38.25
36.01
31.74
29.65
26.65
25.86
21.75
19.21
14.01
Me
N
C
H
H
H
O
12b
220 210 200 190 180 170 160 150 140 130 120 110 100 90 80 70 60 50 40 30 20 10 0
ppm

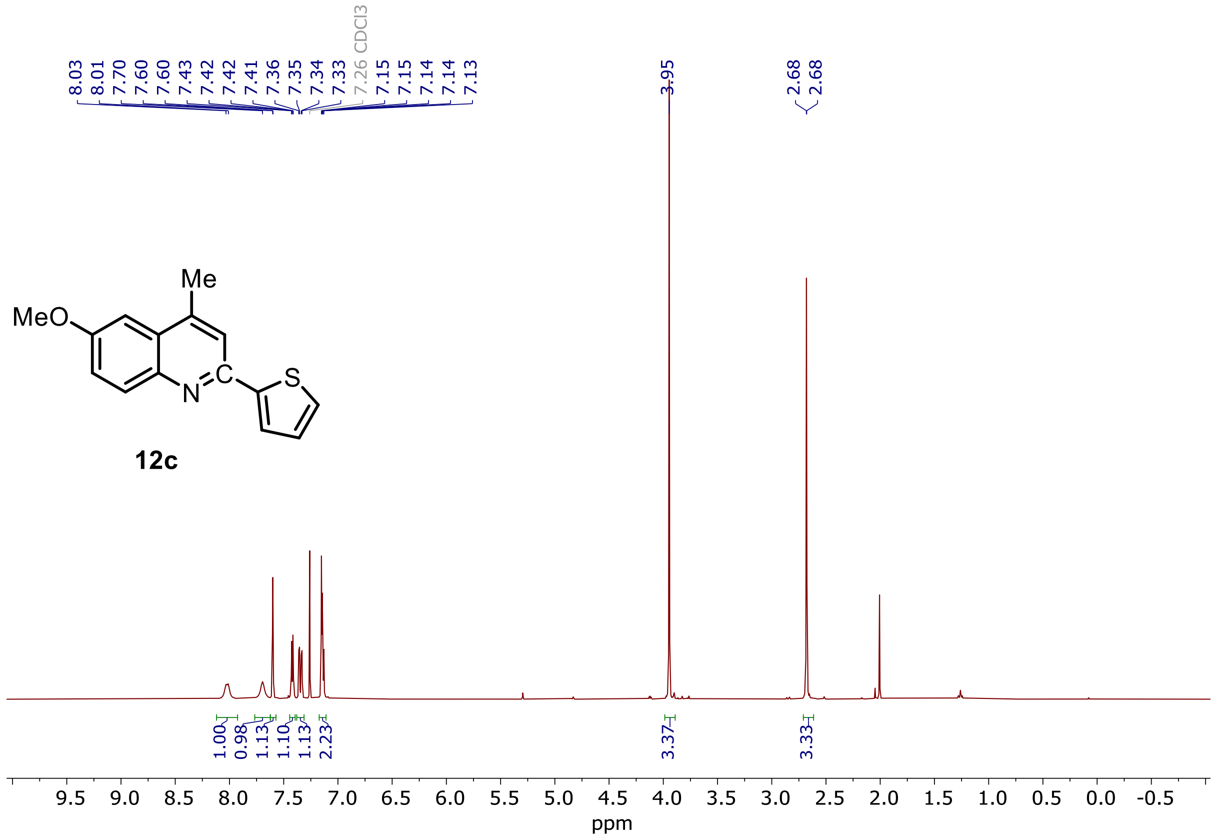

8.03
8.01
7.70
7.60
7.43
7.42
7.42
7.41
7.36
7.35
7.34
7.33
7.26 CDCl3
7.15
7.15
7.14
7.14
7.13
3.95
2.68
2.68
Me
MeO
N
C
S
12c
1.00
0.98
1.13
1.10
1.13
2.23
3.37
3.33
9.5 9.0 8.5 8.0 7.5 7.0 6.5 6.0 5.5 5.0 4.5 4.0 3.5 3.0 2.5 2.0 1.5 1.0 0.5 0.0 -0.5
ppm

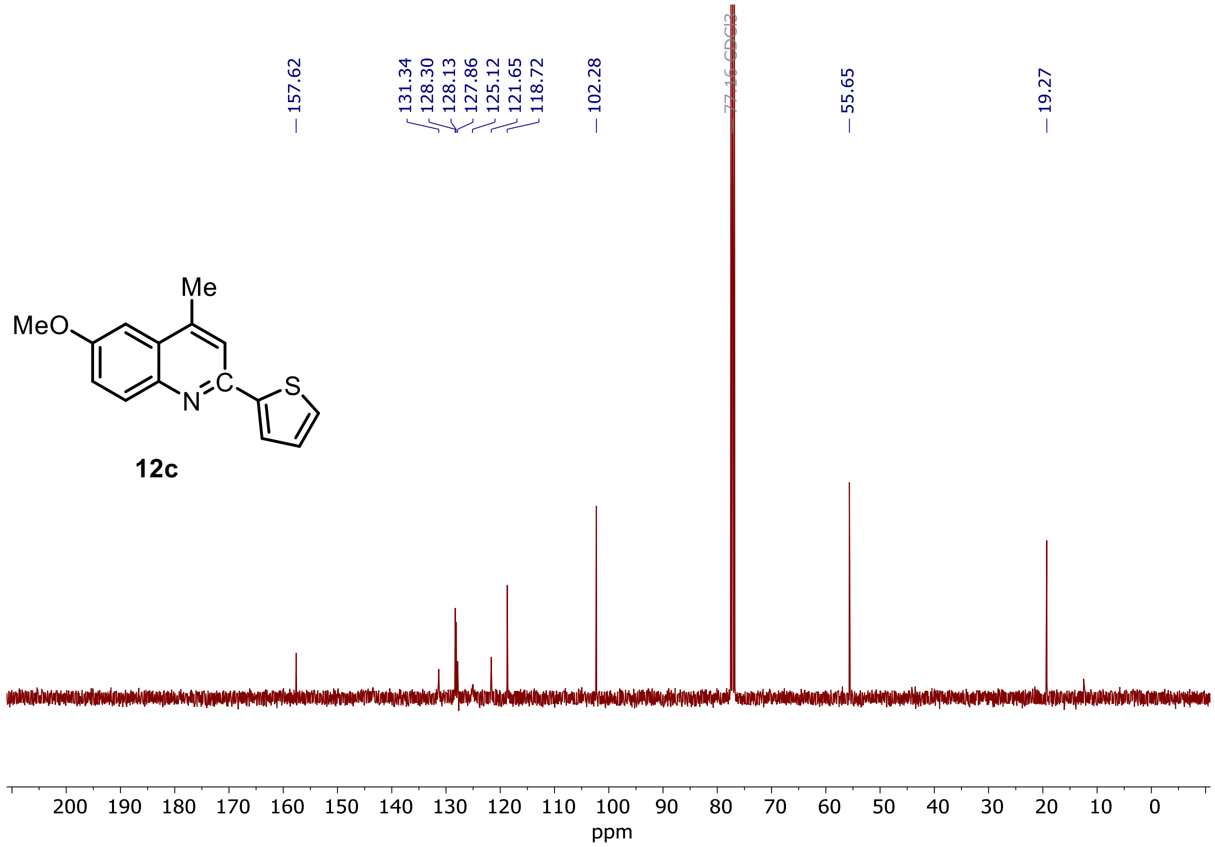
157.62
131.34
128.30
128.13
127.86
125.12
121.65
118.72
102.28
77.16 CDCl3
55.65
19.27
Me
MeO
N
C
S
200 190 180 170 160 150 140 130 120 110 100 90 80 70 60 50 40 30 20 10 0
ppm

12c

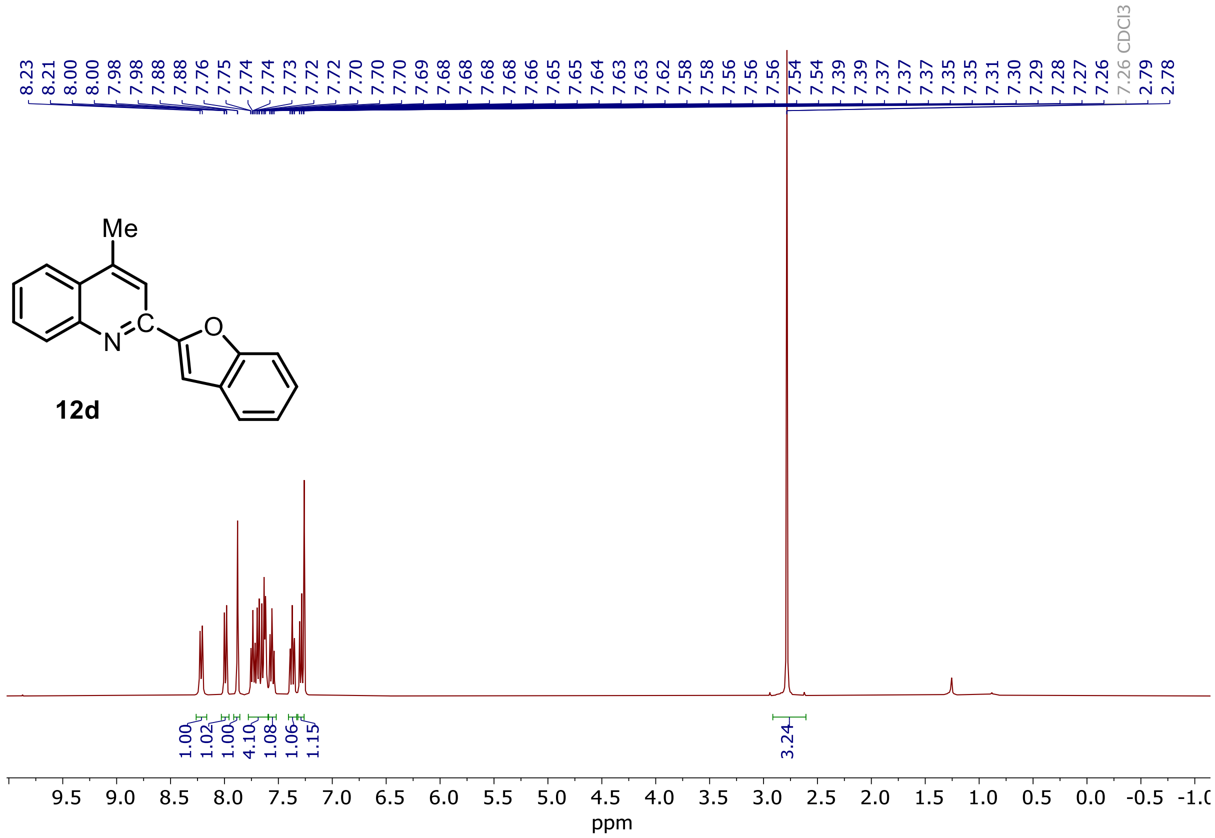
8.23
8.21
8.00
8.00
7.98
7.98
7.88
7.88
7.76
7.75
7.74
7.74
7.73
7.72
7.72
7.70
7.70
7.69
7.68
7.68
7.68
7.66
7.65
7.64
7.63
7.63
7.62
7.58
7.56
7.56
7.56
7.54
7.54
7.39
7.39
7.37
7.37
7.35
7.35
7.31
7.30
7.29
7.28
7.27
7.26
7.26 CDCl3
2.79
2.78
Me
N
C
O
1.00
1.02
1.00
4.10
1.08
1.06
1.15
3.24
9.5 9.0 8.5 8.0 7.5 7.0 6.5 6.0 5.5 5.0 4.5 4.0 3.5 3.0 2.5 2.0 1.5 1.0 0.5 0.0 -0.5 -1.0
ppm

12d

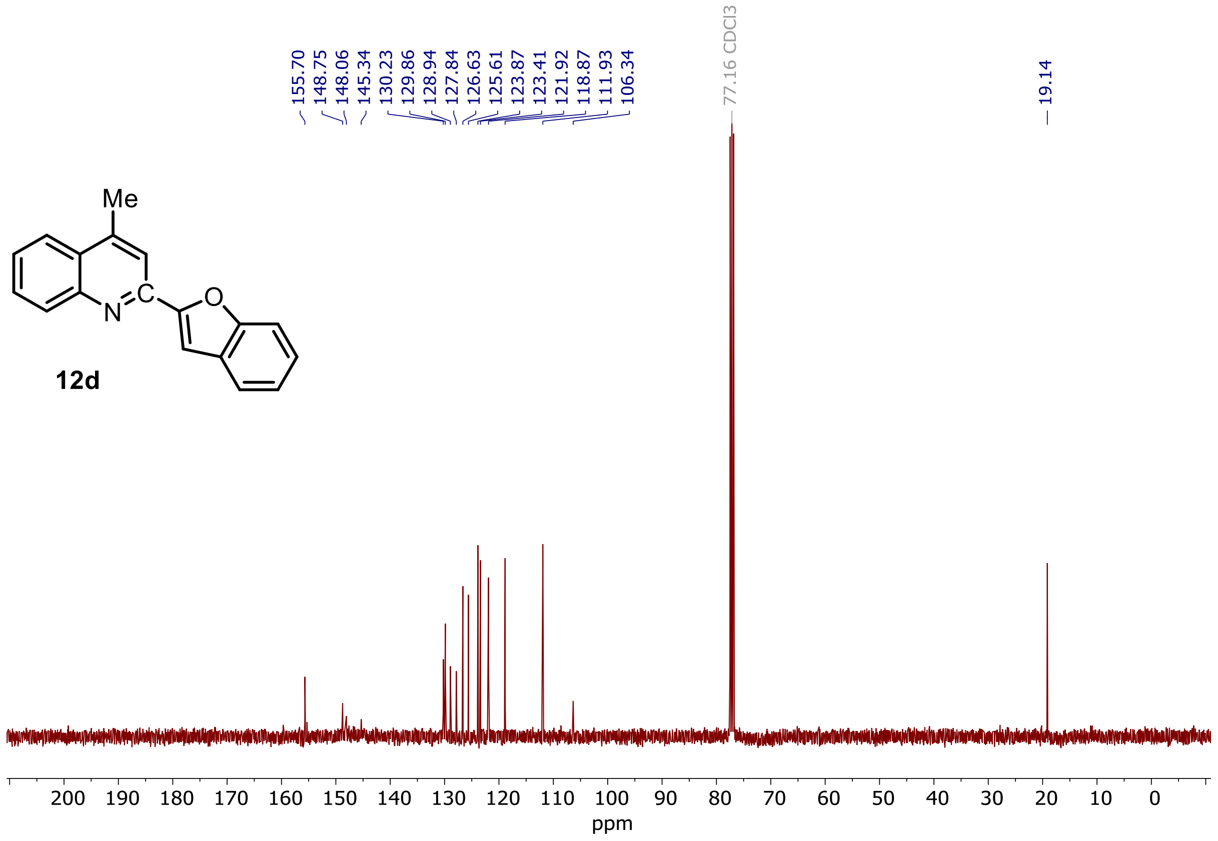
155.70
148.75
148.06
145.34
130.23
129.86
128.94
127.84
126.63
125.61
123.87
123.41
121.92
118.87
111.93
106.34
77.16 CDCl3
19.14
Me
N
C
O
200 190 180 170 160 150 140 130 120 110 100 90 80 70 60 50 40 30 20 10 0
ppm

12d

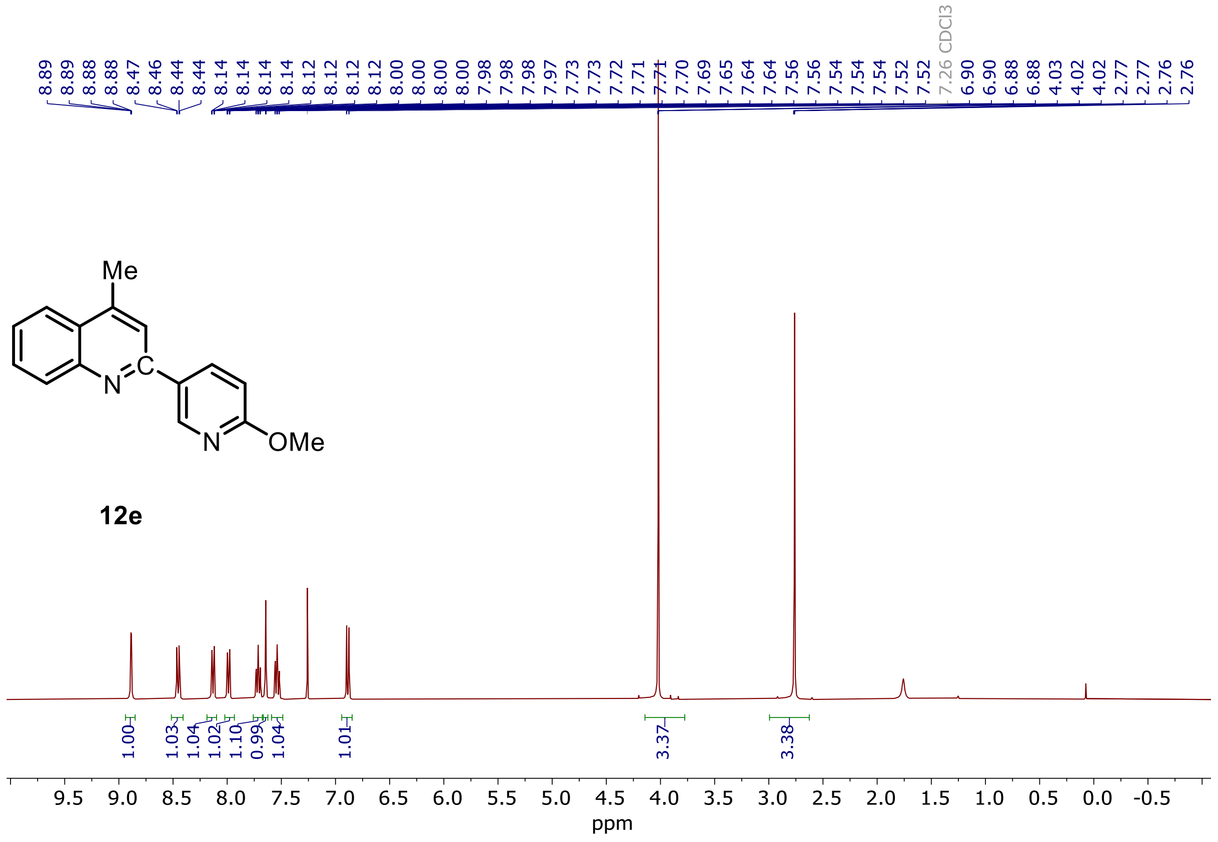
8.89
8.89
8.88
8.88
8.47
8.46
8.44
8.44
8.14
8.14
8.14
8.14
8.12
8.12
8.12
8.12
8.00
8.00
8.00
8.00
7.98
7.98
7.98
7.97
7.73
7.73
7.72
7.71
7.71
7.70
7.69
7.65
7.64
7.64
7.56
7.56
7.54
7.54
7.54
7.52
7.52
7.26 CDCl3
6.90
6.90
6.88
6.88
4.03
4.02
4.02
2.77
2.76
2.76
Me
N
C
N
OMe
1.00
1.03
1.04
1.02
1.10
0.99
1.04
1.01
3.37
3.38
9.5 9.0 8.5 8.0 7.5 7.0 6.5 6.0 5.5 5.0 4.5 4.0 3.5 3.0 2.5 2.0 1.5 1.0 0.5 0.0 -0.5
ppm

12e

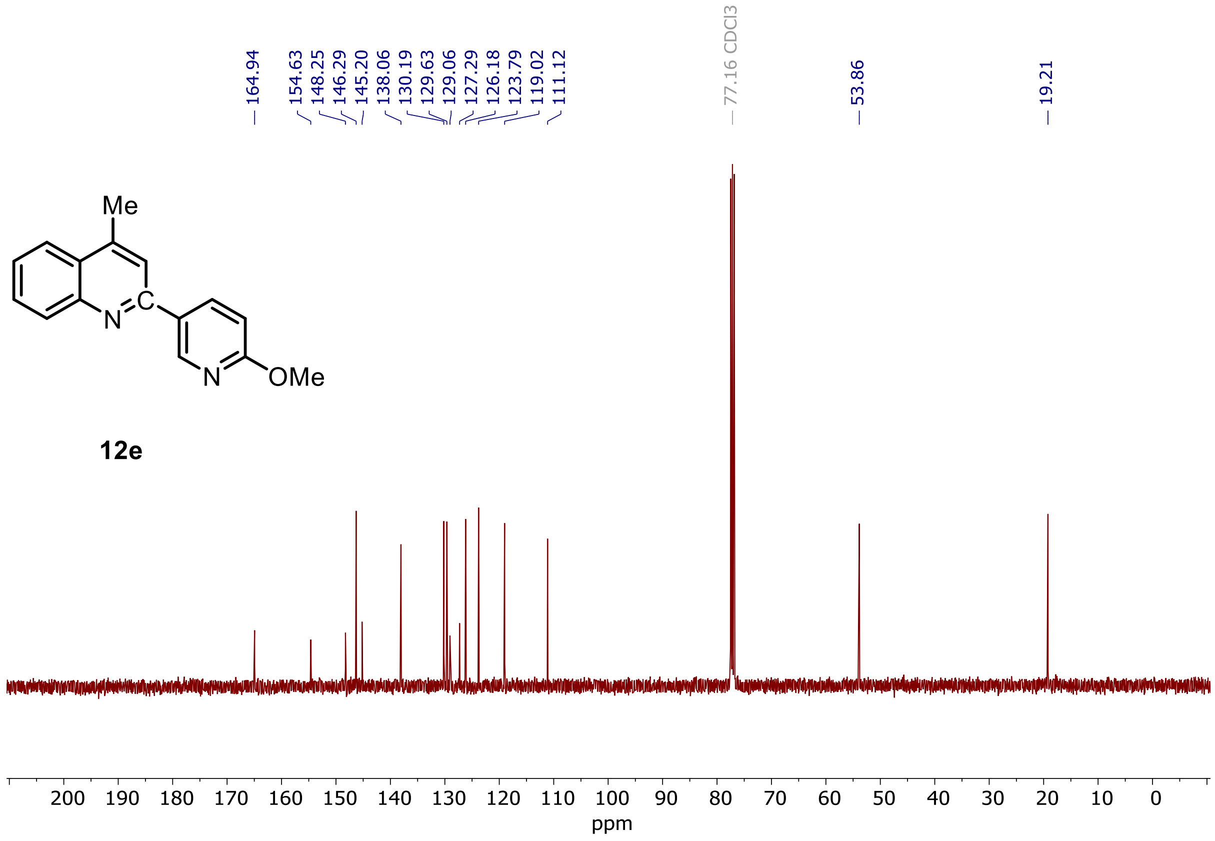
164.94
154.63
148.25
146.29
145.20
138.06
130.19
129.63
129.06
127.29
126.18
123.79
119.02
111.12
77.16 CDCl3
53.86
19.21
Me
N
C
N
OMe
200 190 180 170 160 150 140 130 120 110 100 90 80 70 60 50 40 30 20 10 0
ppm

12e

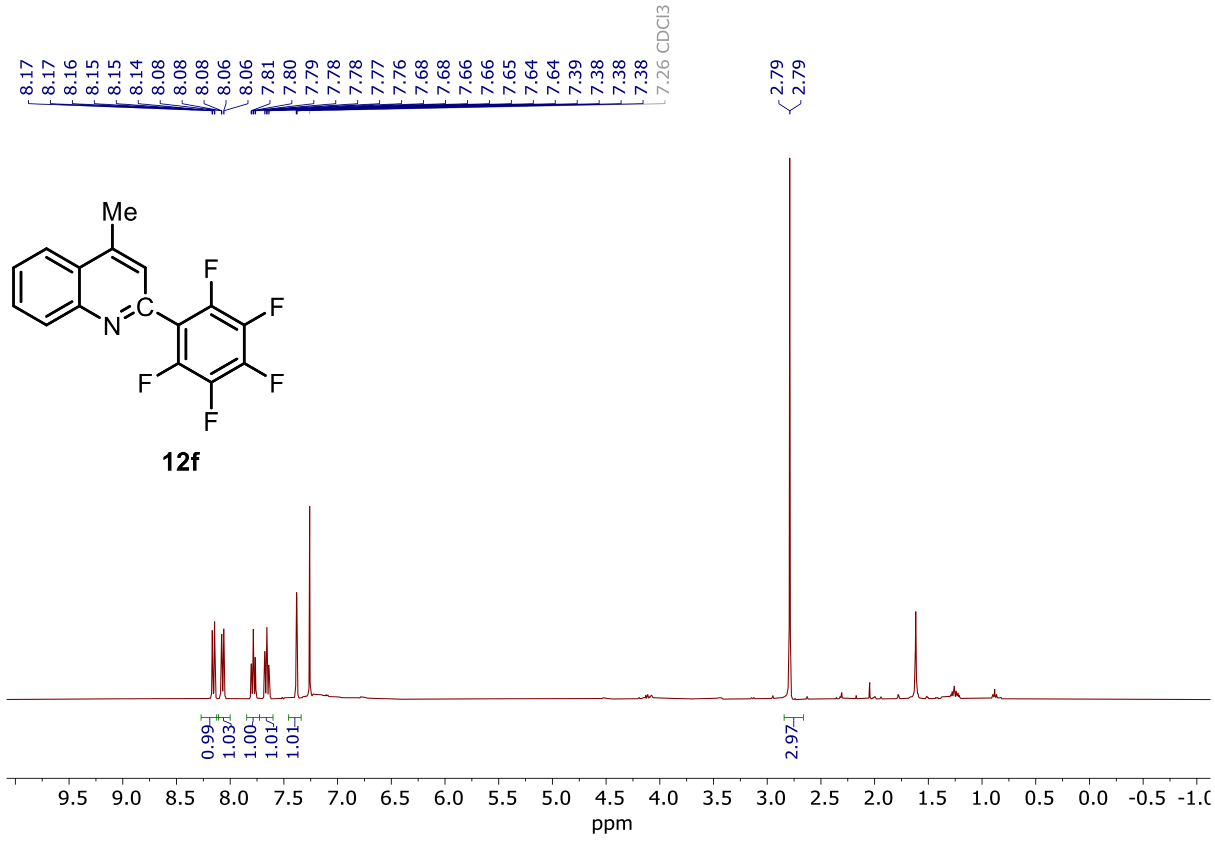
8.17
8.17
8.16
8.15
8.15
8.14
8.08
8.08
8.08
8.06
8.06
7.81
7.80
7.79
7.78
7.78
7.77
7.76
7.68
7.68
7.66
7.65
7.64
7.64
7.39
7.38
7.38
7.38
7.26 CDCl3
2.79
2.79
Me
N
C
F
F
F
F
F
0.99
1.03
1.00
1.01
1.01
2.97
9.5 9.0 8.5 8.0 7.5 7.0 6.5 6.0 5.5 5.0 4.5 4.0 3.5 3.0 2.5 2.0 1.5 1.0 0.5 0.0 -0.5 -1.0
ppm

12f

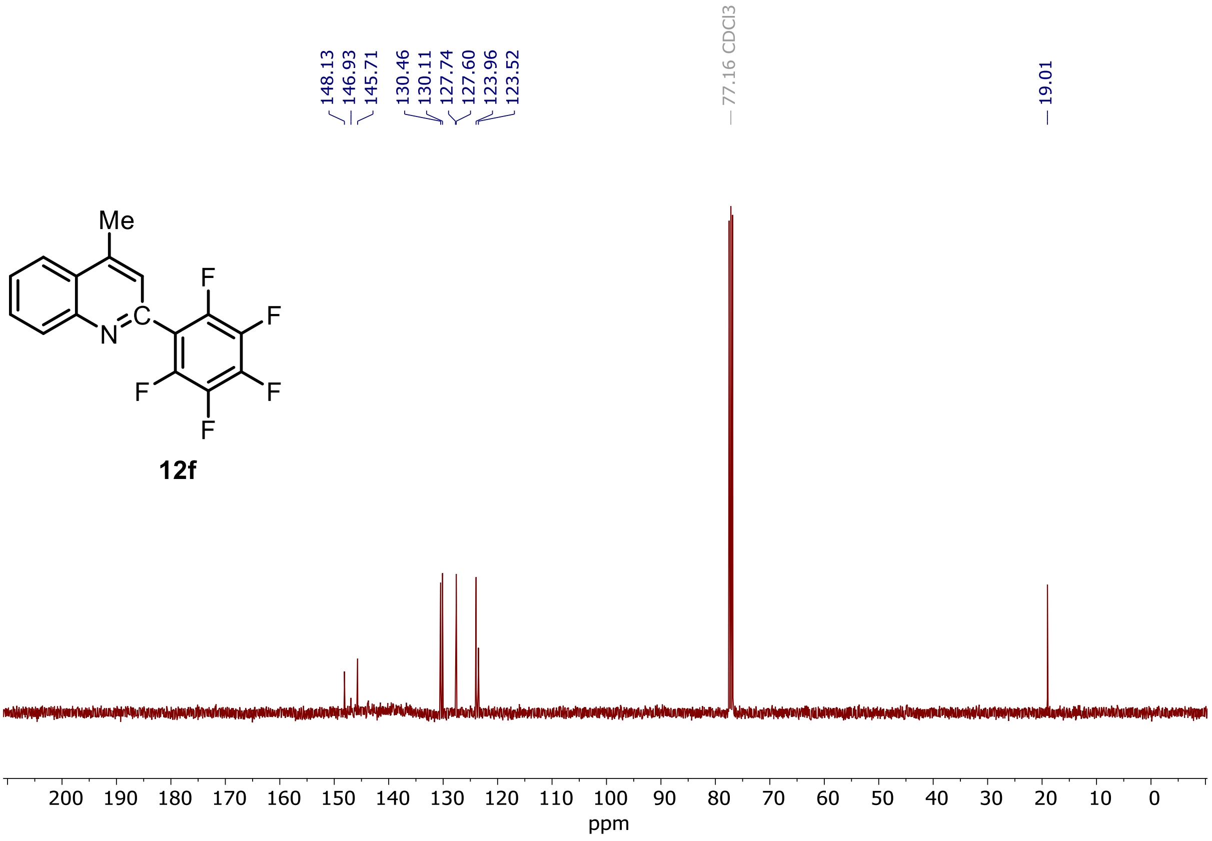
148.13
146.93
145.71
130.46
130.11
127.74
127.60
123.96
123.52
77.16 CDCl3
19.01
Me
N
C
F
F
F
F
F
12f
200 190 180 170 160 150 140 130 120 110 100 90 80 70 60 50 40 30 20 10 0
ppm

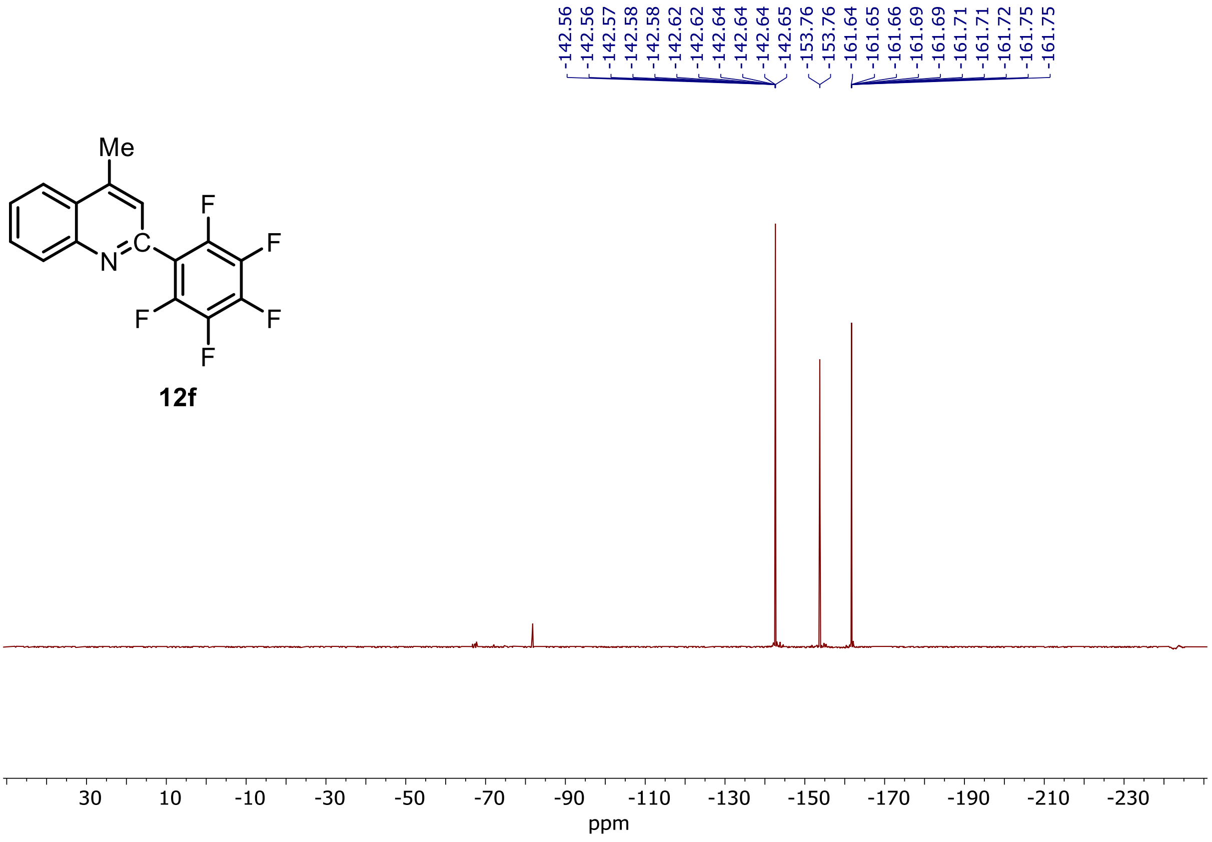
-142.56
-142.56
-142.57
-142.58
-142.62
-142.62
-142.64
-142.64
-142.64
-142.65
-153.76
-153.76
-161.64
-161.65
-161.66
-161.69
-161.69
-161.71
-161.72
-161.75
-161.75
Me
N
C
F
F
F
F
F
12f
30 10 -10 -30 -50 -70 -90 -110 -130 -150 -170 -190 -210 -230
ppm

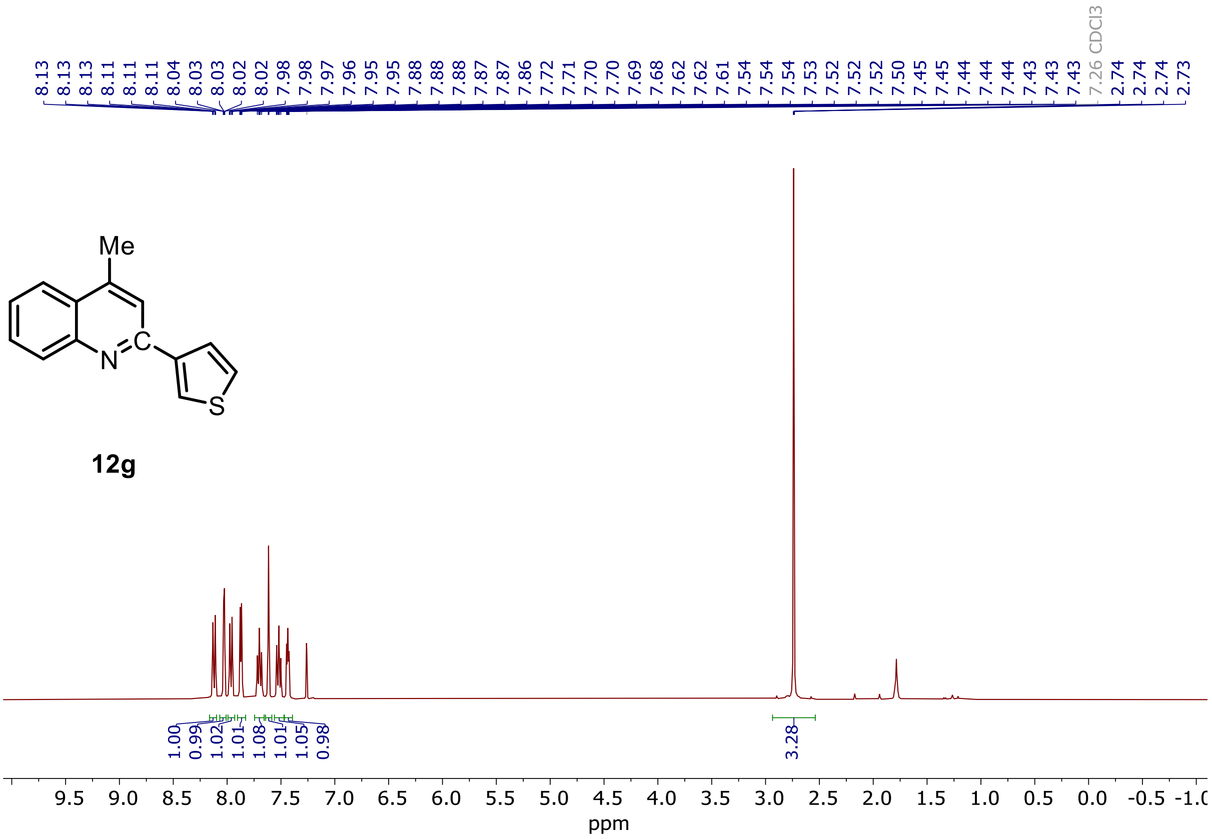
8.13 8.13 8.13 8.11 8.11 8.11 8.04 8.03 8.03 8.02 8.02 7.98 7.98 7.97 7.96 7.95 7.95 7.88 7.88 7.88 7.87 7.87 7.86 7.72 7.71 7.70 7.70 7.69 7.68 7.62 7.62 7.61 7.54 7.54 7.54 7.53 7.52 7.52 7.52 7.50 7.45 7.45 7.44 7.44 7.44 7.43 7.43 7.43 7.26 CDCl3 2.74 2.74 2.74 2.73
Me
N
C
S
12g
1.00 0.99 1.02 1.01 1.08 1.01 1.05 0.98
3.28
9.5 9.0 8.5 8.0 7.5 7.0 6.5 6.0 5.5 5.0 4.5 4.0 3.5 3.0 2.5 2.0 1.5 1.0 0.5 0.0 -0.5 -1.0
ppm

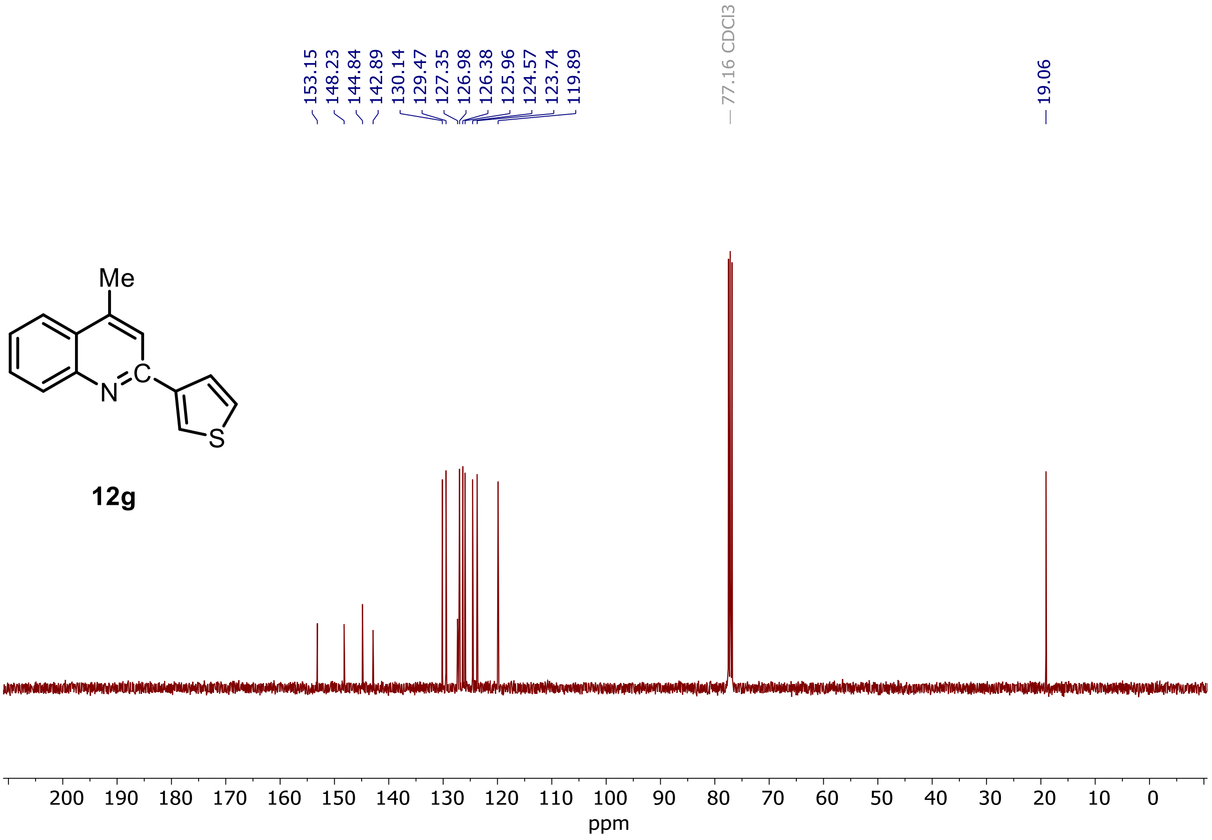
153.15 148.23 144.84 142.89 130.14 129.47 127.35 126.98 126.38 125.96 124.57 123.74 119.89
77.16 CDCl3
19.06
Me
N
C
S
12g
200 190 180 170 160 150 140 130 120 110 100 90 80 70 60 50 40 30 20 10 0
ppm

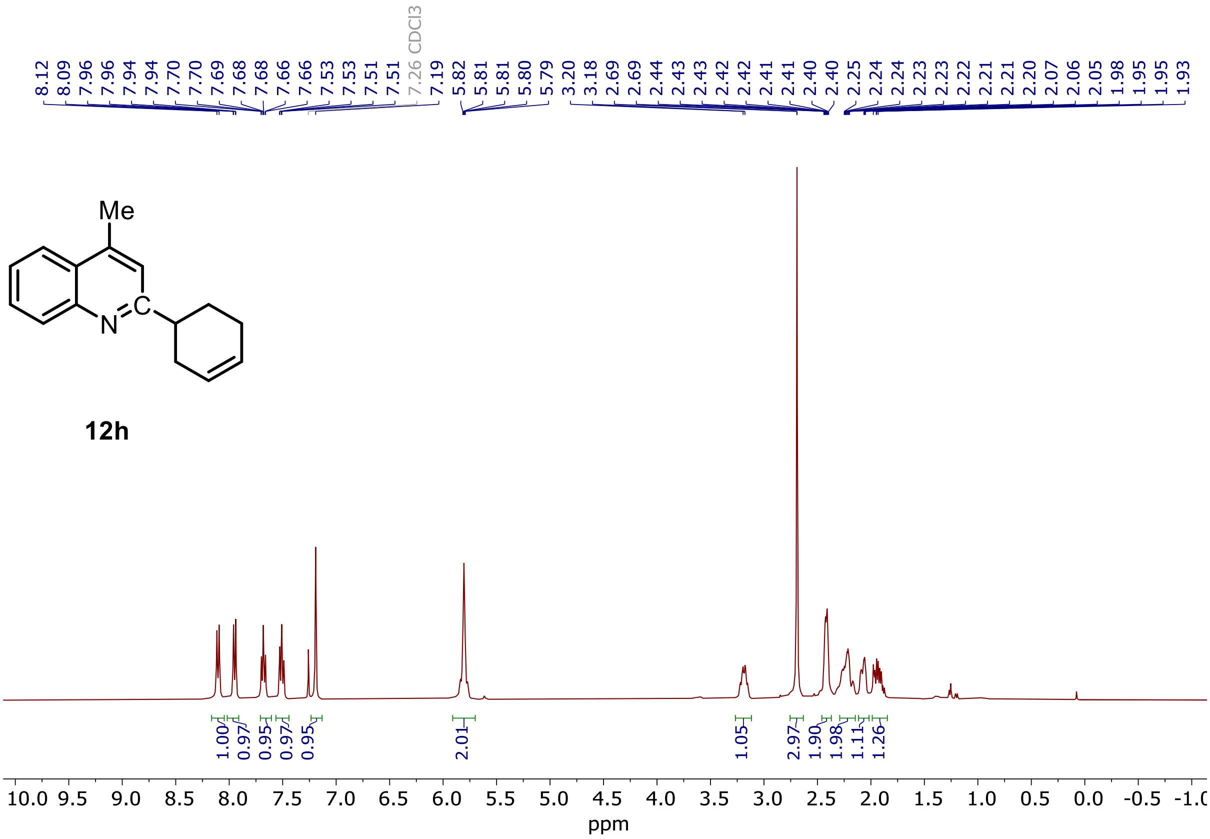
8.12 8.09 7.96 7.96 7.94 7.94 7.70 7.70 7.69 7.68 7.68 7.66 7.66 7.53 7.53 7.51 7.51 7.26 CDCl3 7.19 5.82 5.81 5.81 5.80 5.79 3.20 3.18 2.69 2.69 2.44 2.43 2.43 2.42 2.42 2.41 2.41 2.40 2.40 2.25 2.24 2.24 2.23 2.23 2.22 2.21 2.21 2.20 2.07 2.06 2.05 1.98 1.95 1.95 1.93
Me
N
C
12h
1.00 0.97 0.95 0.97 0.95 2.01 1.05 2.97 1.90 1.98 1.11 1.26
10.0 9.5 9.0 8.5 8.0 7.5 7.0 6.5 6.0 5.5 5.0 4.5 4.0 3.5 3.0 2.5 2.0 1.5 1.0 0.5 0.0 -0.5
ppm

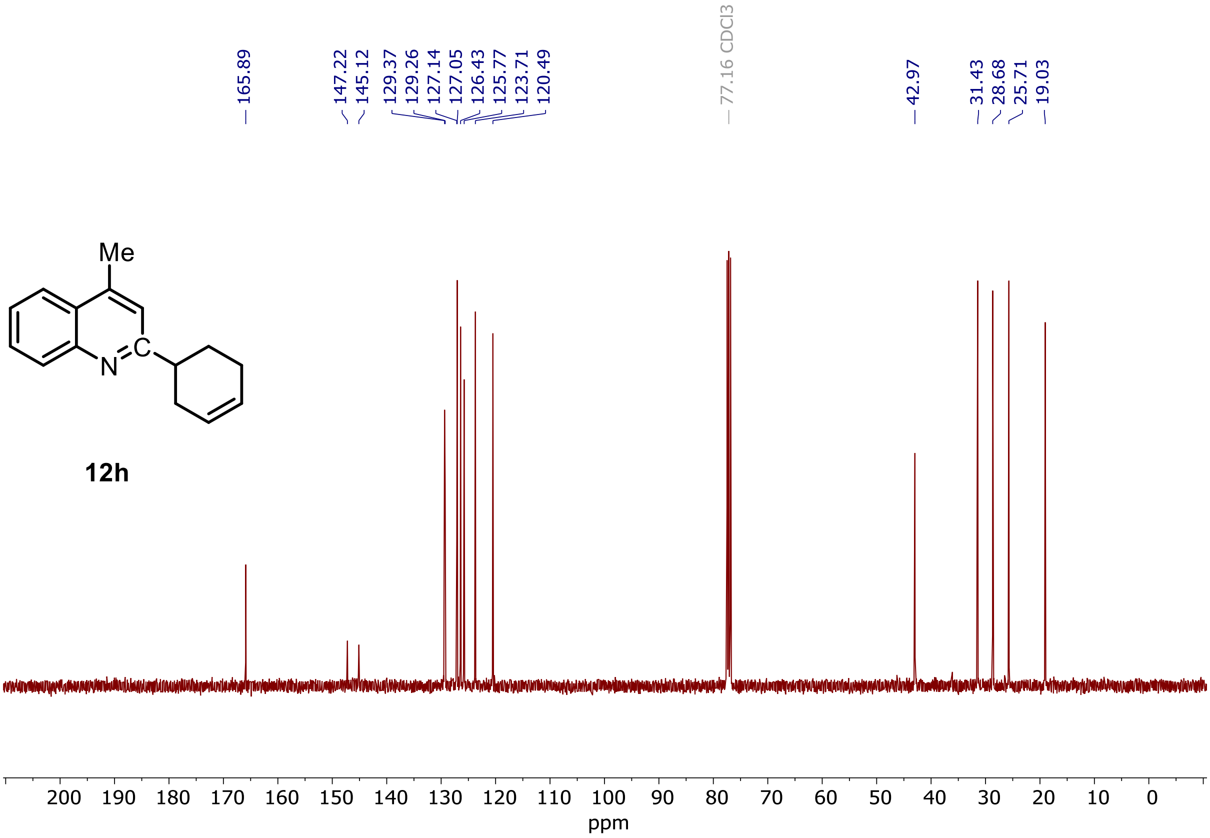
165.89 147.22 145.12 129.37 129.26 127.14 127.05 126.43 125.77 123.71 120.49 77.16 CDCl3 42.97 31.43 28.68 25.71 19.03
Me
N
C
12h
200 190 180 170 160 150 140 130 120 110 100 90 80 70 60 50 40 30 20 10 0
ppm

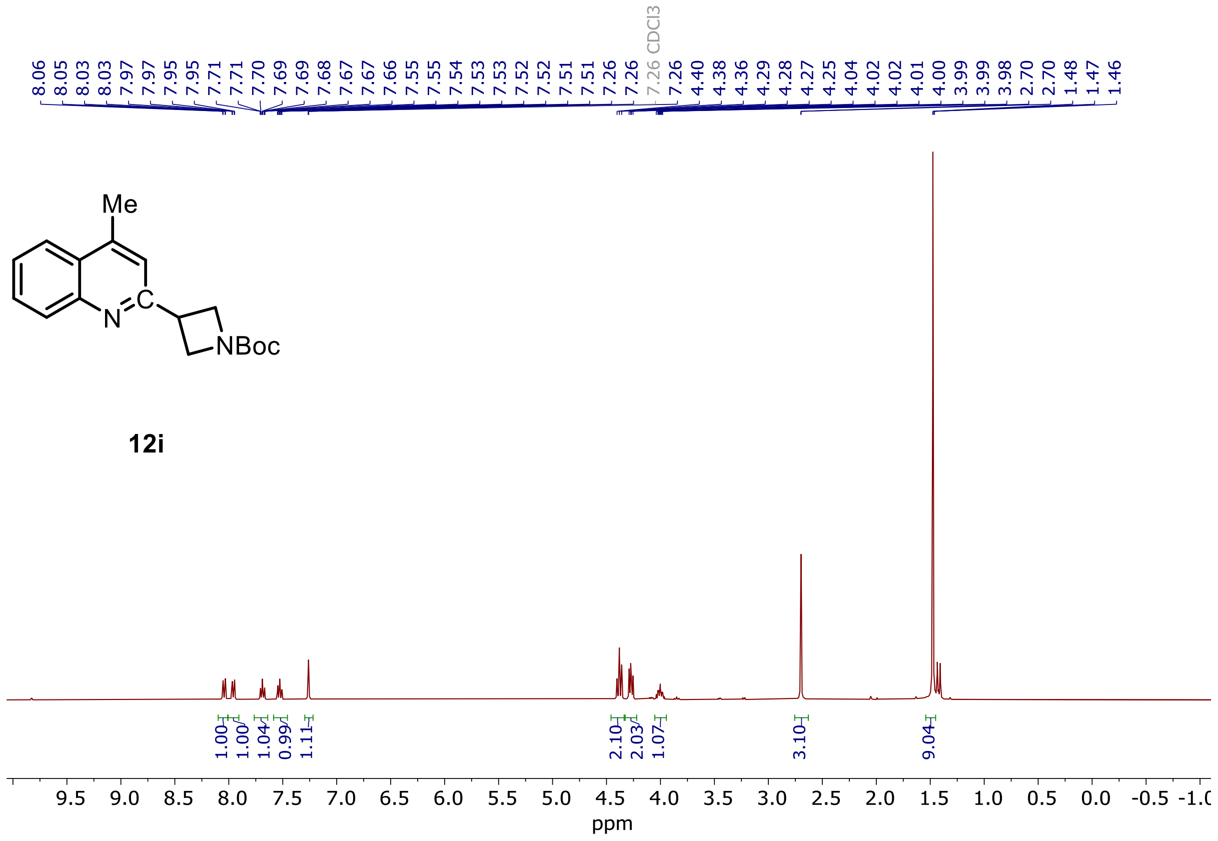
8.06 8.05 8.03 8.03 7.97 7.97 7.95 7.95 7.71 7.71 7.70 7.69 7.69 7.68 7.67 7.67 7.66 7.55 7.55 7.54 7.53 7.53 7.52 7.52 7.51 7.51 7.26 7.26 7.26 CDCl3 7.26 4.40 4.38 4.36 4.29 4.28 4.27 4.25 4.04 4.02 4.02 4.01 4.00 3.99 3.99 3.98 2.70 2.70 1.48 1.47 1.46
Me
N
C
NBoc
12i
1.00
1.00
1.04
0.99
1.11
2.10
2.03
1.07
3.10
9.04
9.5 9.0 8.5 8.0 7.5 7.0 6.5 6.0 5.5 5.0 4.5 4.0 3.5 3.0 2.5 2.0 1.5 1.0 0.5 0.0 -0.5
ppm

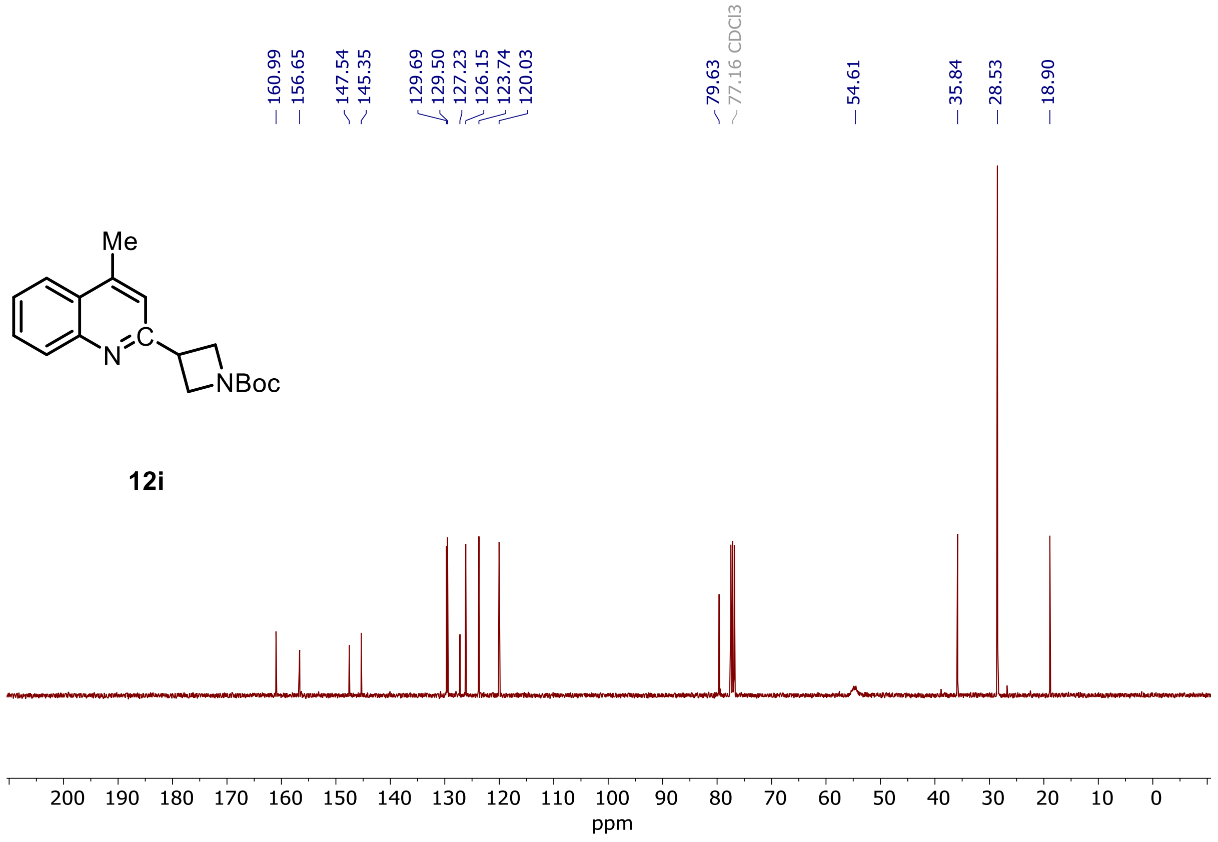
160.99 156.65 147.54 145.35 129.69 129.50 127.23 126.15 123.74 120.03 79.63 77.16 CDCl3 54.61 35.84 28.53 18.90
Me
N
C
NBoc
12i
200 190 180 170 160 150 140 130 120 110 100 90 80 70 60 50 40 30 20 10 0
ppm

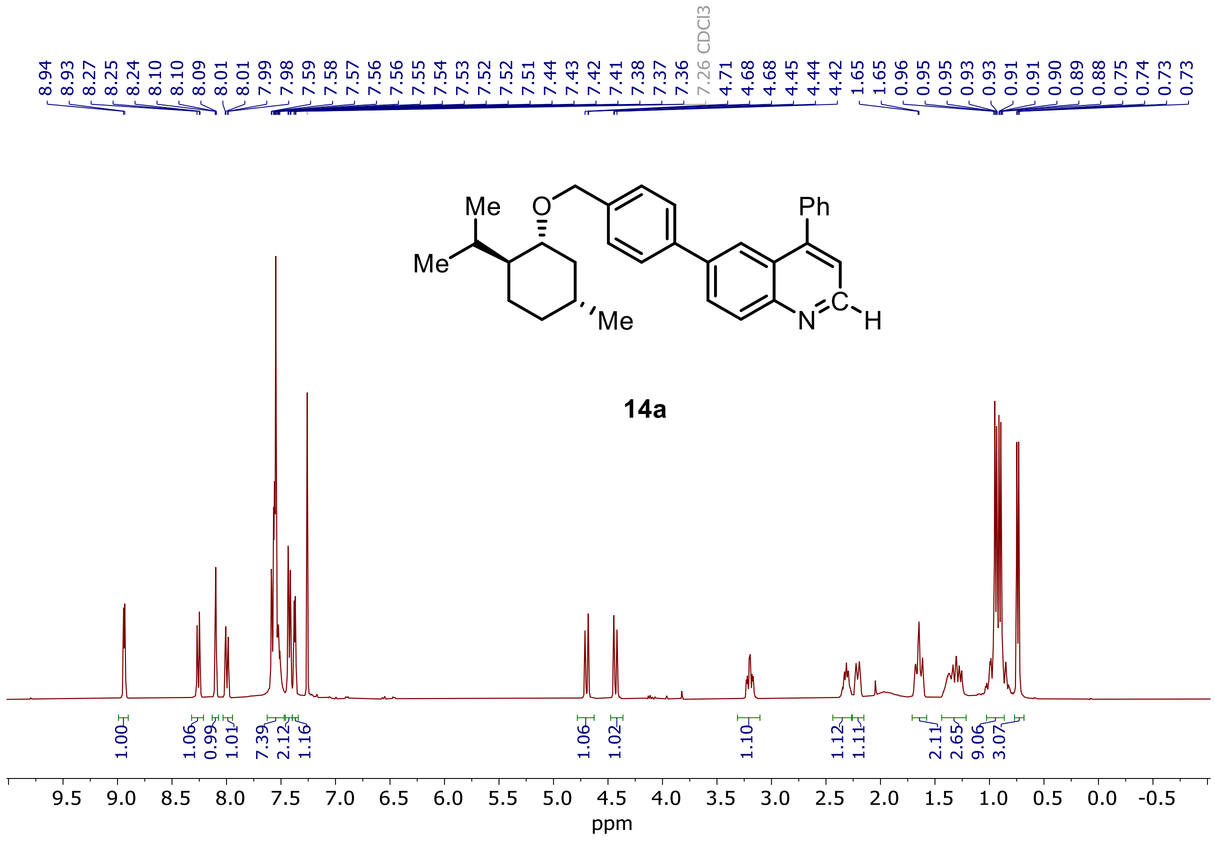

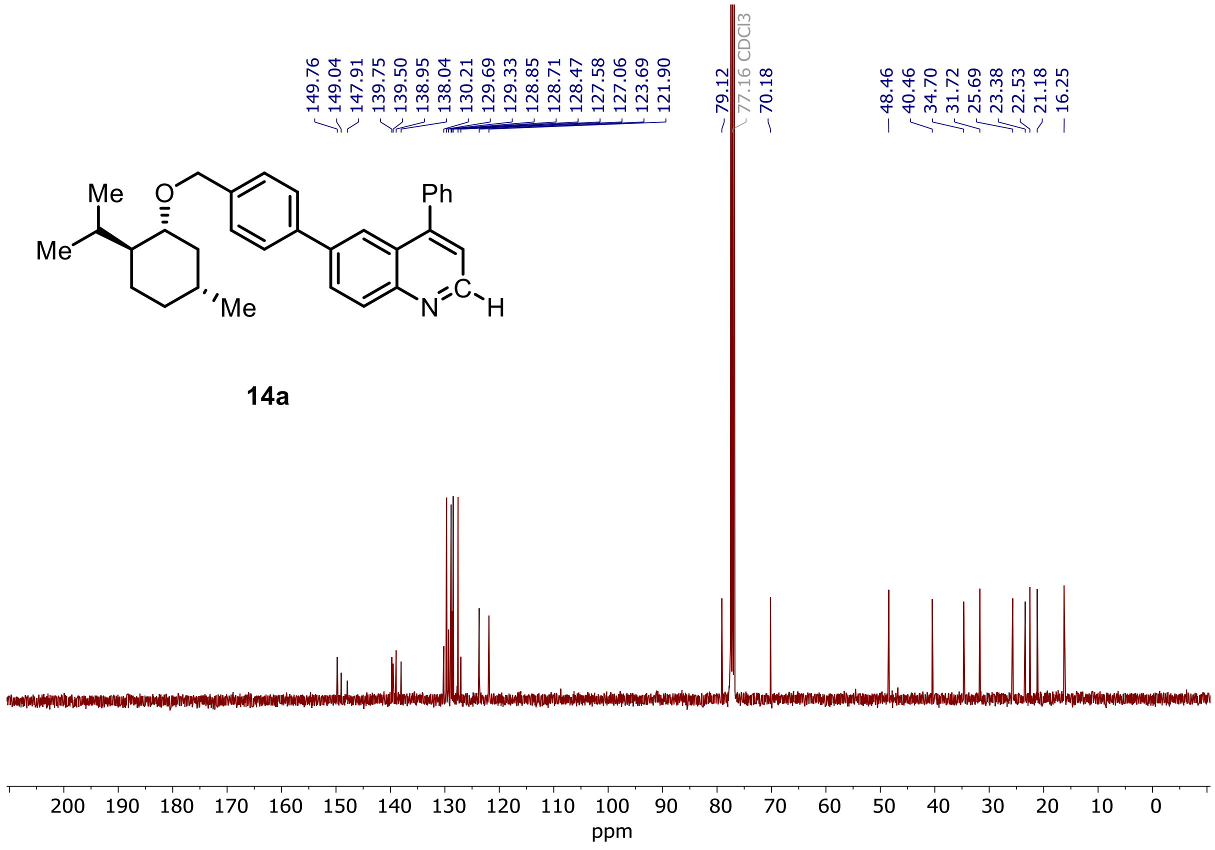

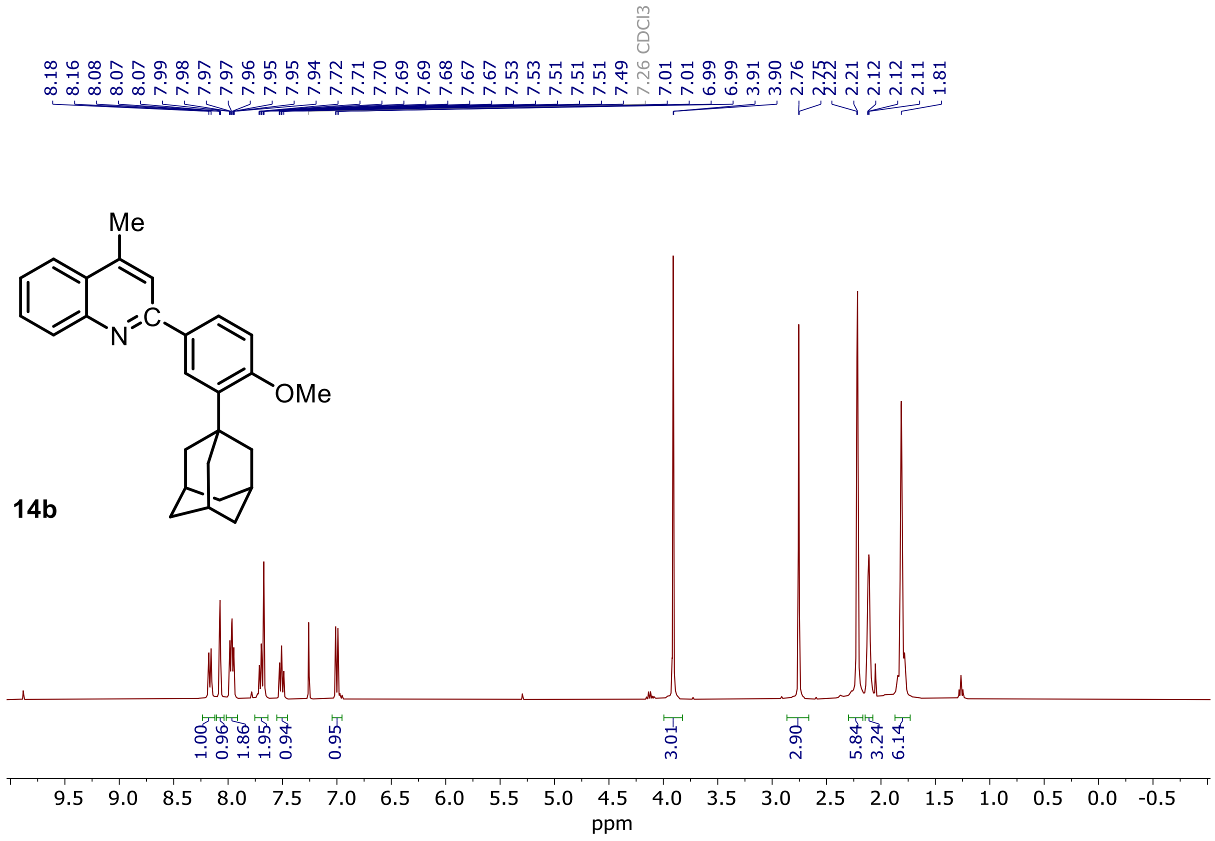
8.18 8.16 8.08 8.07 8.07 7.99 7.98 7.97 7.97 7.96 7.95 7.95 7.94 7.72 7.71 7.70 7.69 7.69 7.68 7.67 7.67 7.53 7.53 7.51 7.51 7.51 7.49 7.26 CDCl3 7.01 7.01 6.99 6.99 3.91 3.90 2.76 2.75 2.22 2.21 2.12 2.12 2.11 1.81
Me
N
C
OMe
14b
1.00 0.96 1.86 1.95 0.94 0.95 3.01 2.90 5.84 3.24 6.14
9.5 9.0 8.5 8.0 7.5 7.0 6.5 6.0 5.5 5.0 4.5 4.0 3.5 3.0 2.5 2.0 1.5 1.0 0.5 0.0 -0.5
ppm

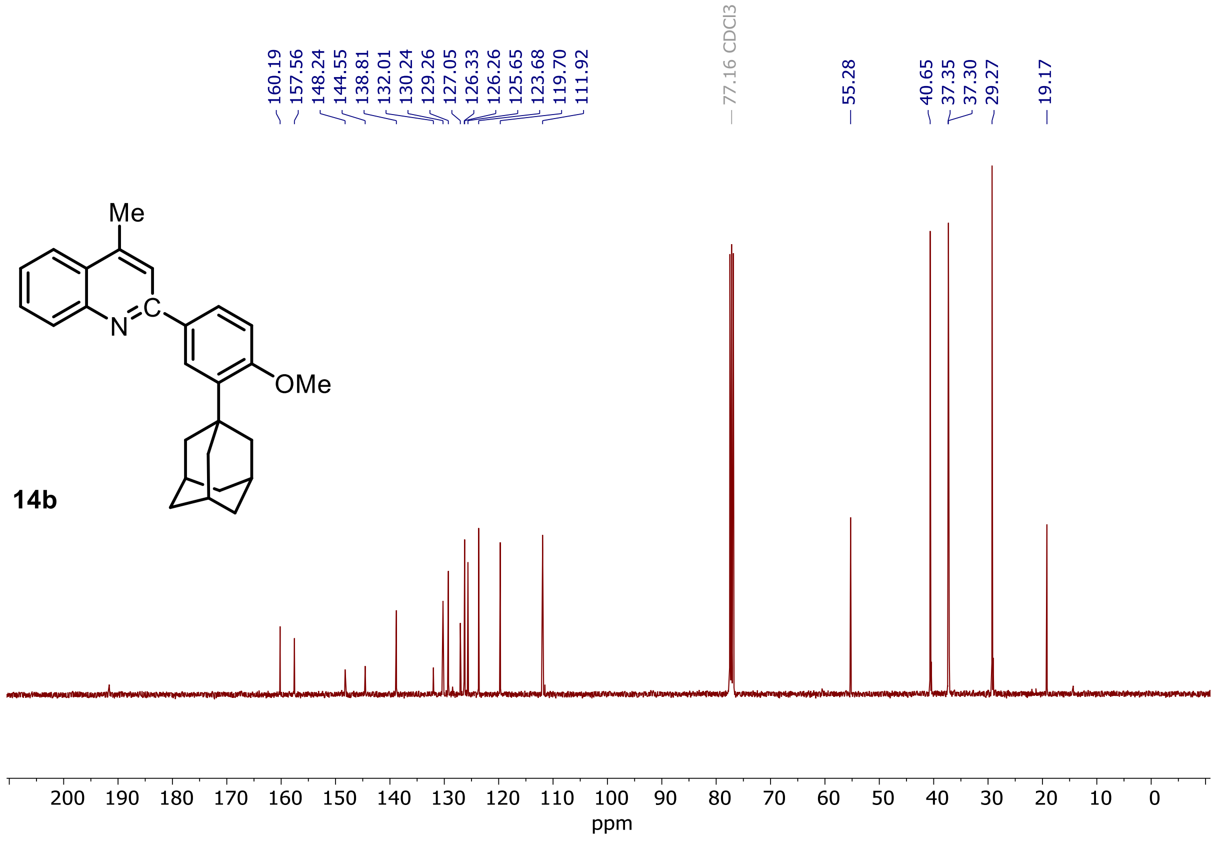
160.19 157.56 148.24 144.55 138.81 132.01 130.24 129.26 127.05 126.33 126.26 125.65 123.68 119.70 111.92
77.16 CDCl3
55.28
40.65 37.35 37.30 29.27
19.17
Me
N
C
OMe
14b
200 190 180 170 160 150 140 130 120 110 100 90 80 70 60 50 40 30 20 10 0
ppm

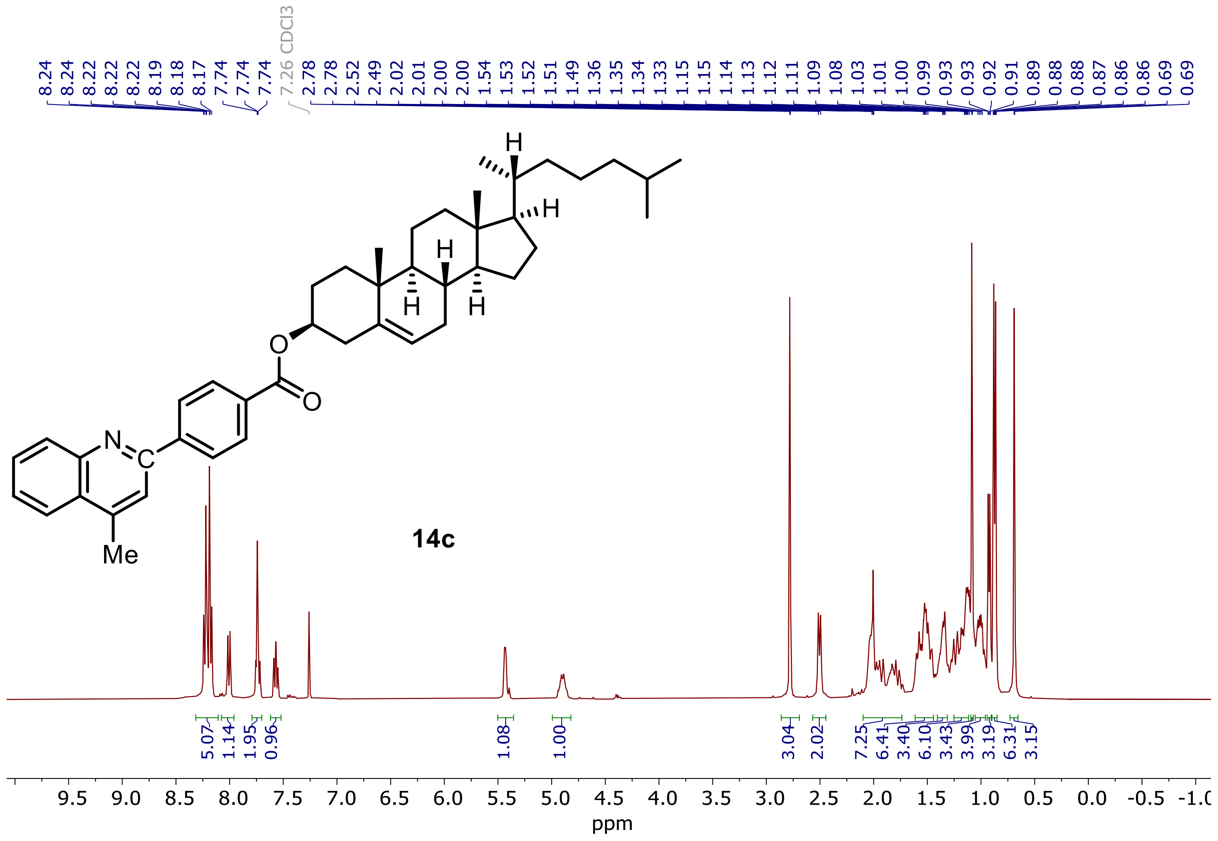

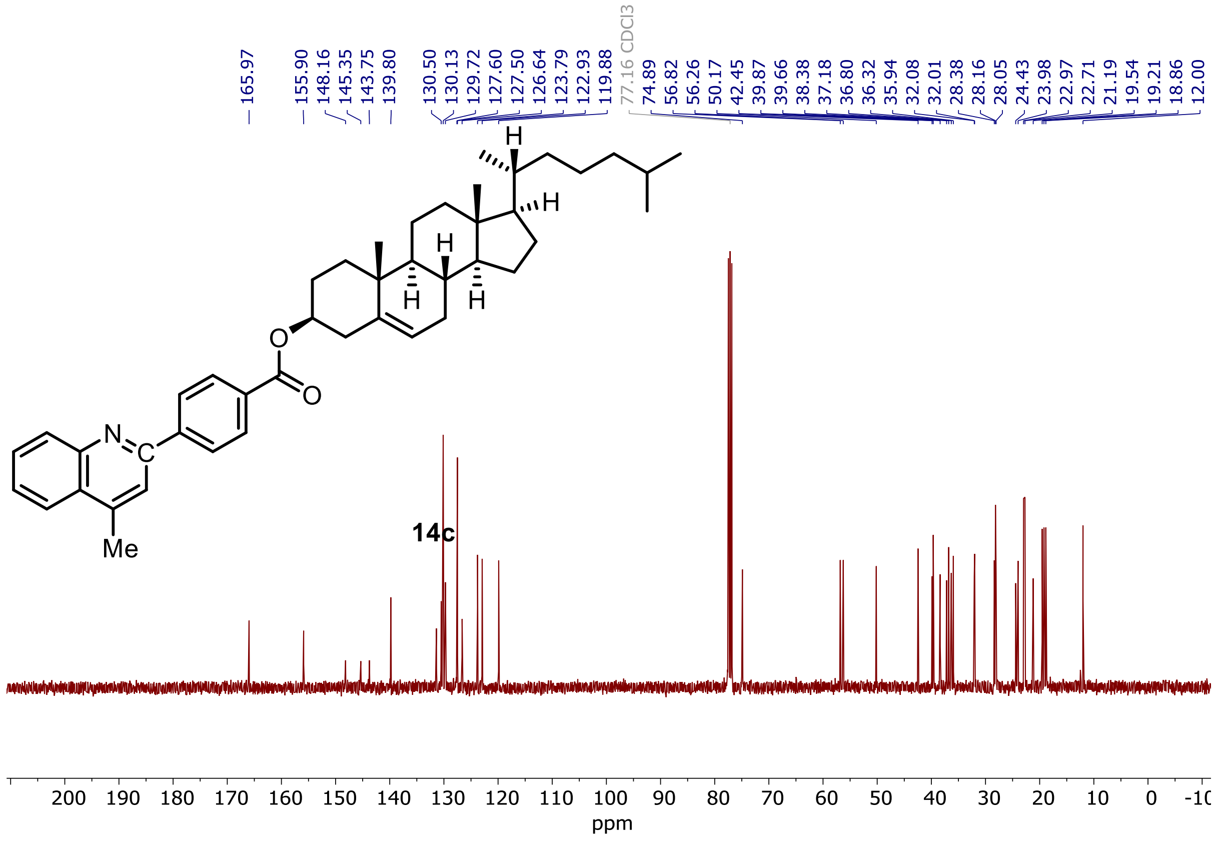

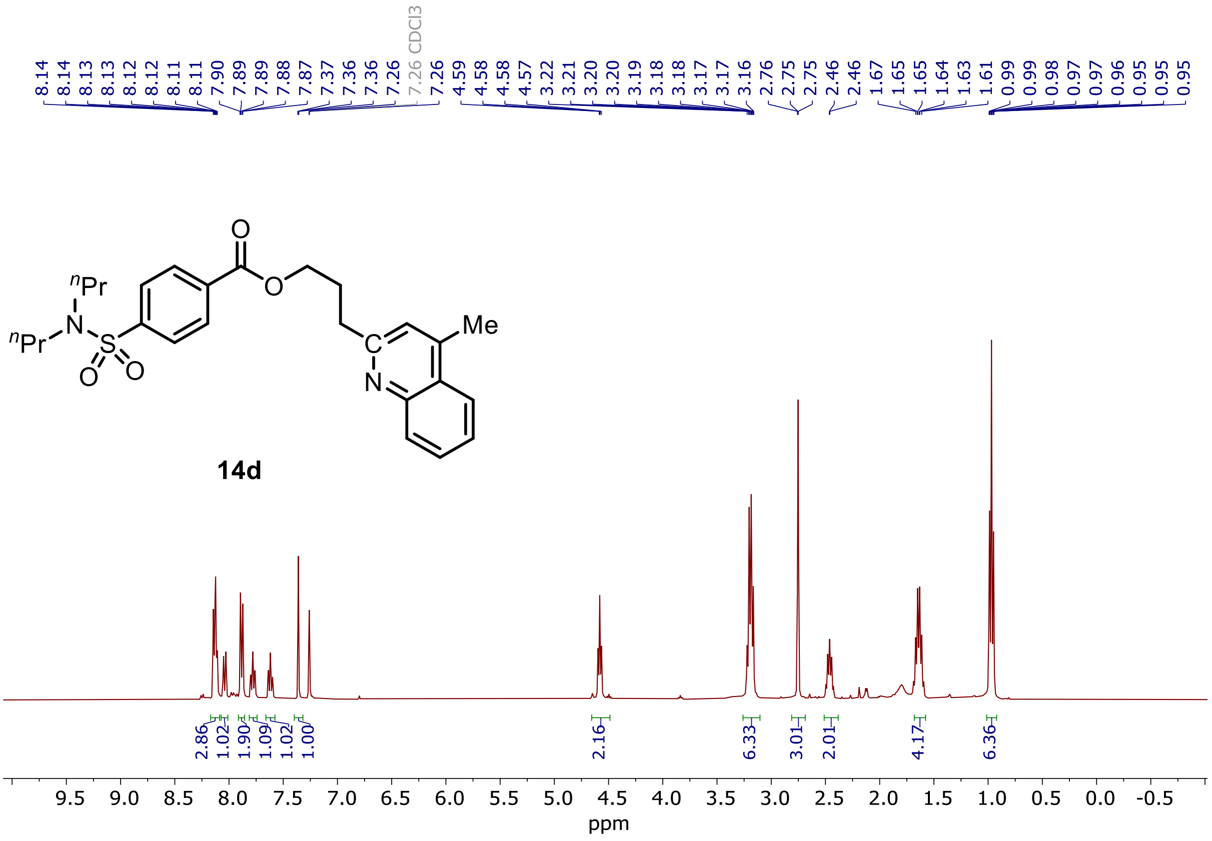
8.14
8.14
8.13
8.13
8.12
8.12
8.11
8.11
7.90
7.89
7.89
7.88
7.87
7.37
7.36
7.36
7.26 CDCl3
7.26
4.59
4.58
4.58
4.57
3.22
3.21
3.20
3.20
3.19
3.18
3.18
3.17
3.17
3.16
2.76
2.75
2.75
2.46
2.46
1.67
1.65
1.65
1.64
1.63
1.61
0.99
0.99
0.98
0.97
0.97
0.96
0.95
0.95
0.95
0.95
14d
2.86
1.02
1.90
1.09
1.02
1.00
2.16
6.33
3.01
2.01
4.17
6.36
ppm

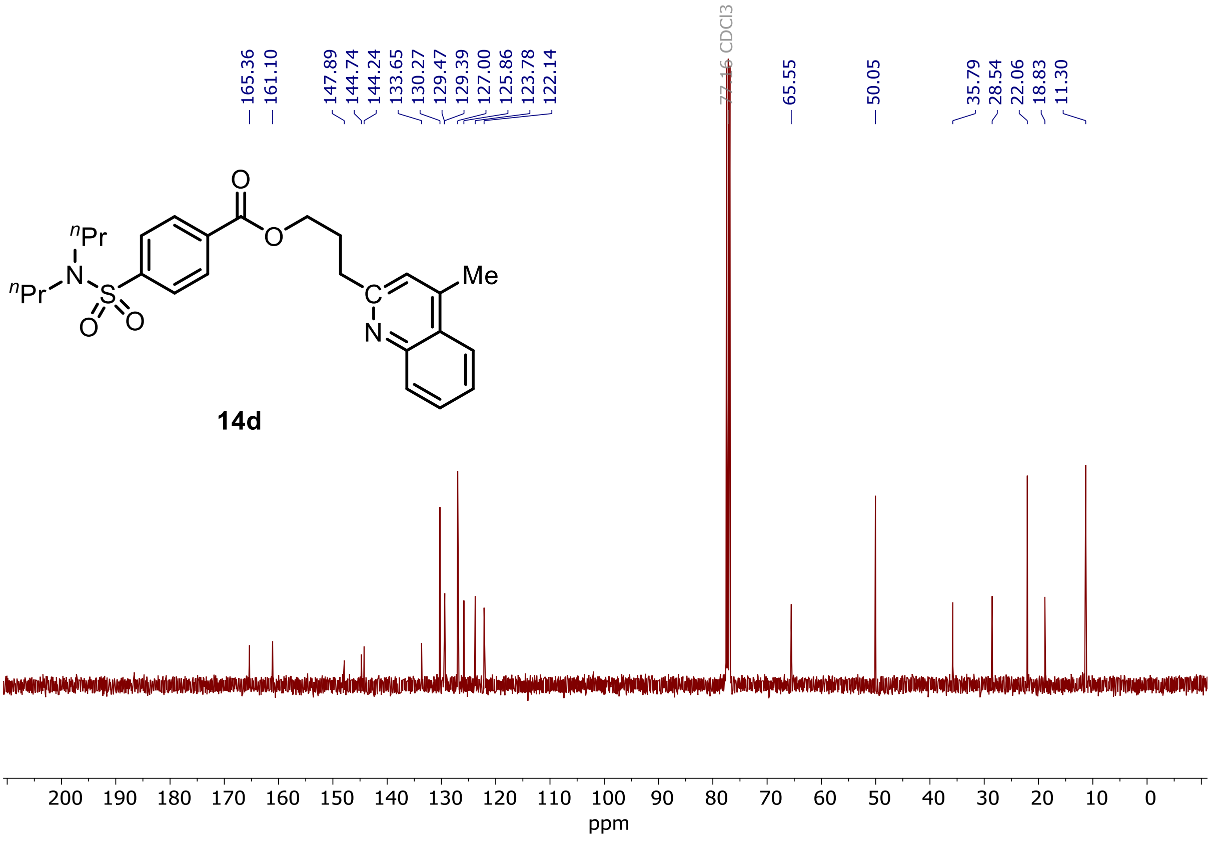
165.36
161.10
147.89
144.74
144.24
133.65
130.27
129.47
129.39
127.00
125.86
123.78
122.14
77.16 CDCl3
65.55
50.05
35.79
28.54
22.06
18.83
11.30
14d
ppm

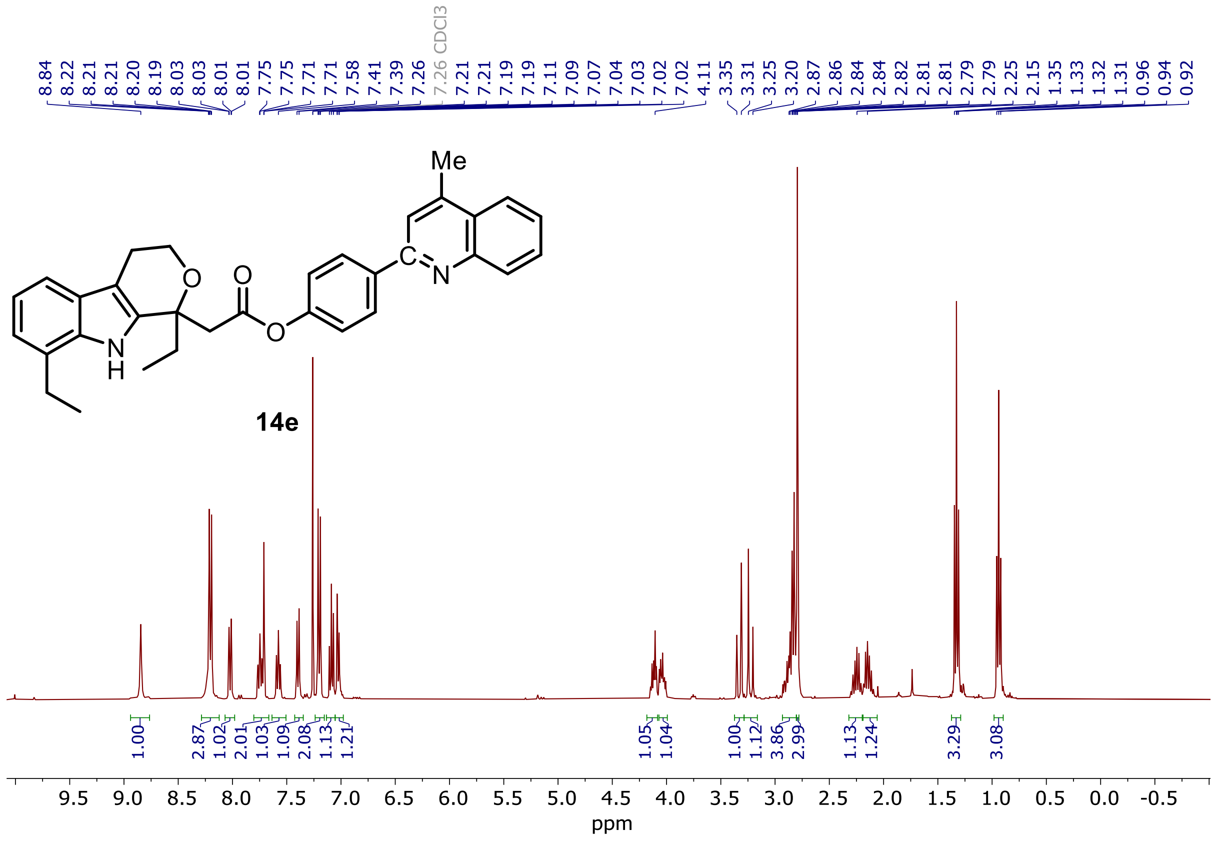

8.84 8.22 8.21 8.21 8.20 8.19 8.03 8.03 8.01 8.01 7.75 7.75 7.71 7.71 7.58 7.41 7.39 7.26 CDCl3 7.21 7.21 7.19 7.19 7.11 7.09 7.07 7.04 7.03 7.02 7.02 4.11 3.35 3.31 3.25 3.20 2.87 2.86 2.84 2.84 2.82 2.81 2.81 2.79 2.79 2.25 2.15 1.35 1.33 1.32 1.31 0.96 0.94 0.92
Me
O
O
O
N
NH
14e
1.00 2.87 1.02 2.01 1.03 1.09 2.08 1.13 1.21 1.05 1.04 1.00 1.12 3.86 2.99 1.13 1.24 3.29 3.08
9.5 9.0 8.5 8.0 7.5 7.0 6.5 6.0 5.5 5.0 4.5 4.0 3.5 3.0 2.5 2.0 1.5 1.0 0.5 0.0 -0.5
ppm

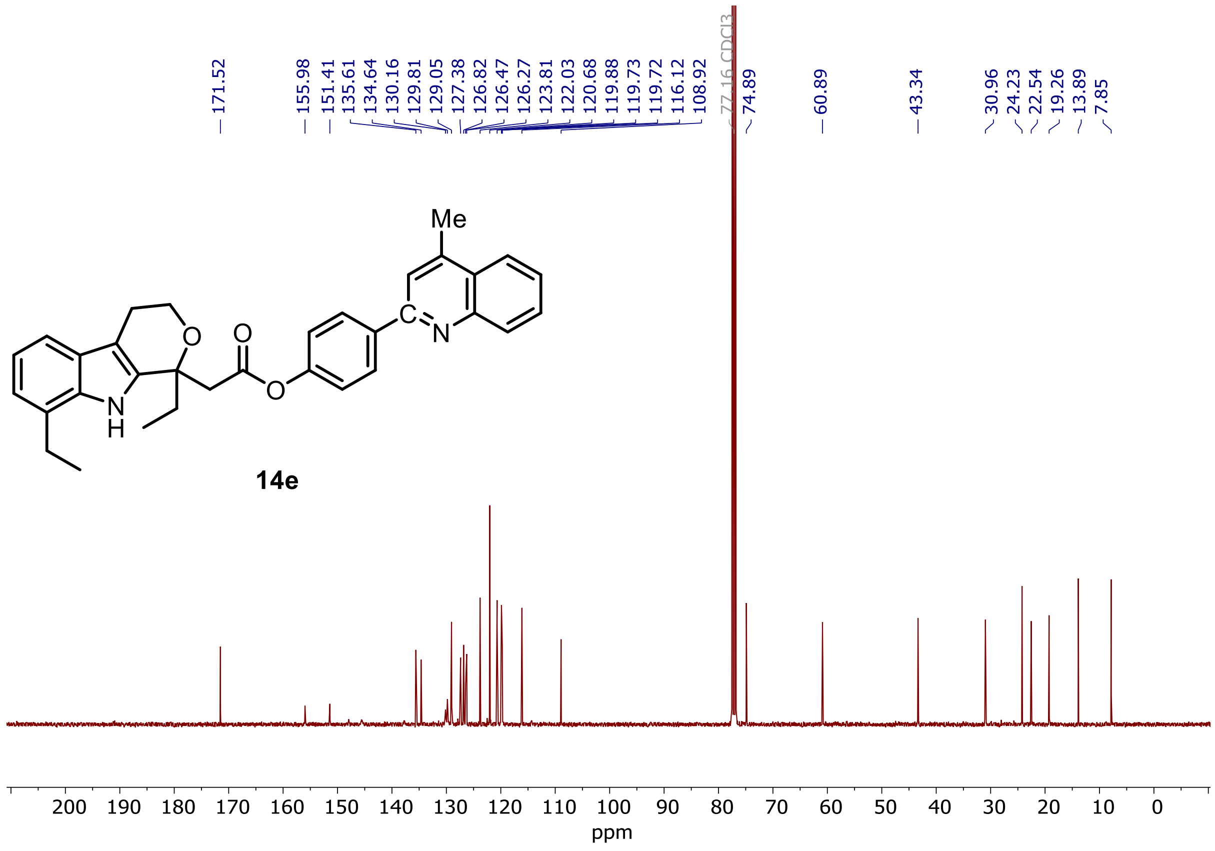

171.52 155.98 151.41 135.61 134.64 130.16 129.81 129.05 127.38 126.82 126.47 126.27 123.81 122.03 120.68 119.88 119.73 119.72 116.12 108.92 77.16 CDCl3 74.89 60.89 43.34 30.96 24.23 22.54 19.26 13.89 7.85
Me
O
O
O
N
NH
14e
200 190 180 170 160 150 140 130 120 110 100 90 80 70 60 50 40 30 20 10 0
ppm

9. Supplementary references

1. Li, Y.-B. et al. A General Three-Component Nozaki−Hiyama−Kishi-Type Reaction Enabled by Delayed Radical-Polar Crossover. *J. Am. Chem. Soc.* **147**, 2642−2652 (2025).

2. Matsuo, B. T., Oliveira, P. H. R., Correia, J. T. M. & Paixão, M. W. Carbamoylation of Azomethine Imines via Visible-Light Photoredox Catalysis. *Org. Lett.* **23**, 6775–6779 (2021).

3. Zhang, X.-L. et al. Stepwise Asymmetric Allylic Substitution-Isomerization Enabled Mimetic Synthesis of Axially Chiral B,N-Heterocycles. *Angew. Chem. Int. Ed.* **61**, e202210456 (2022).

4. Su, W. et al. Copper-catalysed asymmetric hydroboration of alkenes with 1,2-benzazaborines to access chiral naphthalene isosteres. *Nat. Chem.* **16**, 1312–1319 (2024).

5. Zhang, Z. et al. Pd-Catalyzed B−H Aryl/Alkenylation of 1,2-Azaborines. *ACS Catal.* **14**, 16996–17003 (2024).

6. Qiu, W. et al. Iridium-Catalyzed B–H/C–H Dehydrogenative Coupling to Enable Boron–Nitrogen-Embedded Polycyclic Aromatic Hydrocarbons. *J. Am. Chem. Soc.* **148**, 3015–3025 (2026).

7. Pitzer, L., Schäfers, F. & Glorius, F. Rapid Assessment of the Reaction-Condition-Based Sensitivity of Chemical Transformations. *Angew. Chem. Int. Ed.* **58**, 8572–8576 (2019).

8. Aranzaes, J. R., Daniel, M.-C. & Astruc, D. Metallocenes as references for the determination of redox potentials by cyclic voltammetry — Permethylated iron and cobalt sandwich complexes, inhibition by polyamine dendrimers, and the role of hydroxy-containing ferrocenes. *Can. J. Chem.* **84**, 288–299 (2006).

9. Connell, T. U. et al. The Tandem Photoredox Catalysis Mechanism of $[Ir(ppy)_2(dtbbpy)]^+$ Enabling Access to Energy Demanding Organic Substrates. *J. Am. Chem. Soc.* **141**, 17646–17658 (2019).

10. Markovič, M., Lopatka, P., Koóš, P. & Gracza, T. Glyoxylic Acid as a Carbon Monoxide Source for Carbonylation Reactions. *ChemistrySelect* **1**, 2454–2457 (2016).

11. Chu, L., Lipshultz, J. M. & MacMillan, D. W. C. Merging Photoredox and Nickel Catalysis: The Direct Synthesis of Ketones by the Decarboxylative Arylation of α-Oxo Acids. *Angew. Chem. Int. Ed.* **54**, 7929–7933 (2015).

12. Liu, Y. et al. Chromium(II)-Catalyzed Decarboxylative Alkyl Acylation under Visible Light Irradiation. *Org. Lett.* **27**, 6777–6782 (2025).

13. Bruker AXS (**2024**) *APEX6 Version 2024.9-0*, *SAINT Version 8.41* and *SADABS Bruker AXS area detector scaling and absorption correction Version 2016/2*, Bruker AXS Inc., Madison, Wisconsin, USA.

14. Sheldrick, G. M. *SHELXT–Integrated space-group and crystal-structure determination*. *Acta Cryst.* **A71**, 3-8 (2015).

15. Sheldrick, G. M. *Crystal structure refinement with SHELXL*. *Acta Cryst.* **C71**, 3–8 (2015).

16. Bruker AXS (**1998**) *XP–Interactive molecular graphics, Version 5.1,* Bruker AXS Inc., Madison, Wisconsin, USA.
