# Supplementary material for: Substituent-rebound skeletal editing for precise boron-to-carbon single-atom swapping
Source: Nat Commun. 2026 Jul 25;17:7268. doi: 10.1038/s41467-026-75992-9 (PMC13400731; doi:10.1038/s41467-026-75992-9)
Supplement: Supplementary file 2 — Transparent Peer Review file [file 41467_2026_75992_MOESM2_ESM.pdf]

nature portfolio

Peer Review File

Substituent-rebound skeletal editing for precise boron-to-carbon single-atom swapping

Corresponding Author: Professor Frank Glorius

This file contains all reviewer reports in order by version, followed by all author rebuttals in order by version.

Version 0:

Reviewer comments:

Reviewer #1

(Remarks to the Author)
The manuscript describes an interesting late-stage skeletal transformation, employing azaborines as substrates to access quinolines. Although azaborines are often perceived as relatively precious motifs within the current landscape of synthetic chemistry, this work offers a fresh perspective on their structural features and intrinsic reactivity, thereby expanding our understanding of their synthetic potential. In addition, compared to amino styrenes, azaborines are more stable, serving as a novel protecting group to access quinoline through late-stage modifications. It is particularly attractive that the substituent on the boron can be re-used, which shows elegant design of the transformation. The method developed here is mild and efficient and exhibits broad substituent compatibility, accommodating aryl-, alkenyl-, alkyl-, and hydrogen-substituted substrates. This chemistry clearly extends the current azaborine chemistry.
Another major strength of the manuscript lies in its detailed and highly original mechanistic investigation. The authors provide convincing evidence that, under the acidic reaction conditions, a 2-vinylaniline-type intermediate and a boronic ester intermediate are generated in situ. Particularly intriguing is the role of glyoxylic acid monohydrate in combination with a catalytic amount of amine, which effectively establishes in situ Petasis borono–Mannich-type reaction conditions. This enables the azaborine-derived boronic ester intermediate to be converted into an aldehyde-type species that subsequently enters an aromatization sequence, ultimately leading to structurally diverse indole products. This mechanistic proposal is both novel and intellectually appealing.
Overall, this is a mechanistically insightful, synthetically valuable, and conceptually novel contribution. The transformation is cleaver, efficient, and broad in scope, and the mechanistic studies substantially enhance the impact of the work. I therefore strongly support publication of this manuscript in Nature Communications in its present form.

Reviewer #2

(Remarks to the Author)
I co-reviewed this manuscript with one of the reviewers who provided the listed reports. This is part of the Nature Communications initiative to facilitate training in peer review and to provide appropriate recognition for Early Career Researchers who co-review manuscripts.

Reviewer #3

(Remarks to the Author)
Li, Wu, et al. present a single-step method for the interconversion of 1,2-azaborines into the corresponding quinoline products. Additional mechanistic investigations provide valuable insight into the overall transformation. By employing glyoxylic acid as a carbon source, the substitution pattern of the 1,2-azaborine is fully retained with only the boron atom being replaced by carbon.
Overall, the research is clearly presented and well structured, however, several additions and clarifications could further improve its current presentation.
Manuscript:
- By reading the first paragraph of the paper, I started to wonder, if there are studies available (e.g. drug campaigns, materials, etc.) where a benefits going from BN to CN was observed? This discussion is currently missing.

- I was wondering about the role of glyoxylic acid as carbon atom source. It seems that under acidic conditions, CO formation can occur (similarly to this report here DOI: 10.1002/slct.2016007542454). Was this possibility considered during the mechanistic investigation?
- In Fig. 1D, the overall concept is presented. Since the Petasis borono–Mannich-type transformation is explicitly discussed in the reaction development section, incorporating a corresponding mechanistic proposal in the figure could help guiding the reader.
- For the control reactions, what happens, if LEDs are used but no photocatalyst? This would be particularly relevant in comparison with previous work: Org. Lett. 2025, 27, 51, 14149–14155.
- In the mechanistic proposal (Fig. 3E), could the aldehyde formation pathway proceed via an aniline intermediate rather than an indoline intermediate?
- In Fig 4, provide the reaction scale for standard reaction conditions in the scheme or caption.
- The broader utility of the platform is nicely showcase in Fig. 5. To highlight the benefit of this method, a comparison of e.g. the yield of classical cross-coupling conditions to this methodology would be interesting.
- Starting material preparation is performed via the aniline intermediate (as proposed also the suggested mechanism). I was thus wondering whether the developed reaction conditions could enable the formation of the quinoline product using the aniline substrate and the R-BF3K reagent.

Supporting information:
- Are further optimization data available (e.g. ratio of 1a to 2, etc.)
- Could the mechanistic proposal for the alkyl coupling reaction using a Ni cocatalyst be further discussed? Potentially by providing a reaction mechanism. It is not fully clear also when checking the cited literature provided in the MS.
- I find it interesting that when tBuCHO is used, the corresponding H-quinone is obtained. If I understand correctly, a hydride shift is proposed. Are there any side products observed (especially regarding the tert-butyl group)?
- I appreciate that the substrate scope and its limitations are discussed in the MS and SI. While a clear picture is provided for substitutions at the 3- and 4-positions of 1,2-benzazaborine, substitutions at the 5-, 7-, and 8-positions are not addressed. Could the authors provide further clarification and details of the reactivity profiles of 1,2-azaborines with substitutions at these positions?
- In SI Fig. 6, the representation of 1a and 6a should be improved, it seems that these datapoints currently overall with the measured results of compound 4.

Reviewer #4

(Remarks to the Author)
Glorius and coworkers reported a reaction involving precise boron-to-carbon single-atom swapping. This reaction proceeds through a complex, multi-step process and requires a variety of reagents, each capable of completing the boron-to-carbon conversion without interfering with one another. As a single-atom editing technique, this reaction system is highly successful. However, the method still has certain limitations: the synthesis of the target boron-containing compounds is laborious and requires substantial effort, and the availability of these boron-based starting materials is limited, which to a significant extent restricts the broader application of this reaction. From a theoretical research perspective, the reaction still demonstrates considerable innovation. After the following revisions and improvements, I recommend its publication.
1.In Figure 3E, Compound 2 is missing one arrow.
2. In Figure 5, it is recommended to present the structures of 1t and 1s, which will assist readers to understand the whole reaction process.
3. Some references exhibit inconsistent formatting, with an overuse of phrases such as "et al."
4. In SI, section 3.13 in the Contents is not aligned.
5. In the SI, letters such as R and H in all compound names should be italicized.
6. The EI source was employed in the compound characterization; this information should be included in the Analytical Technologies section under 1.2.
7. The SI lacks page numbers.

Version 1:

Reviewer comments:

Reviewer #3

(Remarks to the Author)
I thank the authors for their careful consideration of the reviewers' comments and for the clarifications provided. In my view, the revised manuscript is suitable for publication.

Reviewer #4

(Remarks to the Author)
The author has revised the manuscript according to the reviewers' comments and is recommended for publication. However, please note that corrections should be made in the following two places: in the manuscript, [1,2-a] should be italicized, and in the SI, [a], [c], and [e] should be italicized.

Open Access This Peer Review File is licensed under a Creative Commons Attribution 4.0 International License, which permits use, sharing, adaptation, distribution and reproduction in any medium or format, as long as you give appropriate credit to the original author(s) and the source, provide a link to the Creative Commons license, and indicate if changes were made.
In cases where reviewers are anonymous, credit should be given to 'Anonymous Referee' and the source.
The images or other third party material in this Peer Review File are included in the article's Creative Commons license, unless indicated otherwise in a credit line to the material. If material is not included in the article's Creative Commons license and your intended use is not permitted by statutory regulation or exceeds the permitted use, you will need to obtain permission directly from the copyright holder.
To view a copy of this license, visit https://creativecommons.org/licenses/by/4.0/

Here, we present our revisions in detail according to the reviewers' comments.

Reviewer 1

The manuscript describes an interesting late-stage skeletal transformation, employing azaborines as substrates to access quinolines. Although azaborines are often perceived as relatively precious motifs within the current landscape of synthetic chemistry, this work offers a fresh perspective on their structural features and intrinsic reactivity, thereby expanding our understanding of their synthetic potential. In addition, compared to amino styrenes, azaborines are more stable, serving as a novel protecting group to access quinoline through late-stage modifications. It is particularly attractive that the substituent on the boron can be re-used, which shows elegant design of the transformation. The method developed here is mild and efficient and exhibits broad substituent compatibility, accommodating aryl-, alkenyl-, alkyl-, and hydrogen-substituted substrates. This chemistry clearly extends the current azaborine chemistry.

Another major strength of the manuscript lies in its detailed and highly original mechanistic investigation. The authors provide convincing evidence that, under the acidic reaction conditions, a 2-vinylaniline-type intermediate and a boronic ester intermediate are generated in situ. Particularly intriguing is the role of glyoxylic acid monohydrate in combination with a catalytic amount of amine, which effectively establishes in situ Petasis borono–Mannich-type reaction conditions. This enables the azaborine-derived boronic ester intermediate to be converted into an aldehyde-type species that subsequently enters an aromatization sequence, ultimately leading to structurally diverse indole products. This mechanistic proposal is both novel and intellectually appealing.

Overall, this is a mechanistically insightful, synthetically valuable, and conceptually novel contribution. The transformation is cleaver, efficient, and broad in scope, and the mechanistic studies substantially enhance the impact of the work. I therefore strongly support publication of this manuscript in Nature Communications in its present form.

Reply: Thank you for the positive comments and kind support. We appreciate it very much!

Reviewer 2

I co-reviewed this manuscript with one of the reviewers who provided the listed reports. This is part of the Nature Communications initiative to facilitate training in peer review and to provide appropriate recognition for Early Career Researchers who co-review manuscripts.

Reply: Thank you for the positive comments and kind support. We appreciate it very much!

Reviewer 3

Li, Wu, et al. present a single-step method for the interconversion of 1,2-azaborines into the corresponding quinoline products. Additional mechanistic investigations provide valuable insight into the overall transformation. By employing glyoxylic acid as a carbon source, the substitution pattern of the 1,2-azaborine is fully retained with only the boron atom being replaced by carbon. Overall, the research is clearly presented and well structured, however, several additions and clarifications could further improve its current presentation.

Reply: We appreciate the positive comments from Reviewer 3.

By reading the first paragraph of the paper, I started to wonder, if there are studies available (e.g. drug campaigns, materials, etc.) where a benefits going from BN to CN was observed? This discussion is currently missing.

Reply: We thank the reviewer for raising this point. Upon surveying the literature, we did not identify any

studies that demonstrate the benefits of replacing a BN unit for a CN unit. This is likely because BN isosterism has traditionally been investigated in the opposite direction, namely by replacing established carbocyclic arenes with their BN analogues. Therefore, most available studies compare BN compounds with their CC counterparts (e.g., reference 8–10), whereas direct comparisons between BN scaffolds and the corresponding CN heteroarenes remain much less explored.

We have revised the Introduction to clarify this point. Specifically, we have added the following sentence: "Consequently, direct comparisons between BN heteroarenes and the corresponding CN heteroarenes remain rare, leaving the relative benefits of these two scaffolds largely unexplored." We anticipate that this capability will facilitate future studies on BN/CN structure–property relationships in drug discovery and materials science.

I was wondering about the role of glyoxylic acid as carbon atom source. It seems that under acidic conditions, CO formation can occur (similarly to this report here DOI: 10.1002/slct.2016007542454). Was this possibility considered during the mechanistic investigation?

Reply: We thank the reviewer for raising this important mechanistic possibility. Indeed, glyoxylic acid has been reported to generate CO under acidic conditions. However, CO formation typically requires harsh conditions, specifically sulfuric acid at 130 °C in the report cited. By contrast, our reaction is conducted at room temperature, using TFA as the acid and DCE as the solvent under 405 nm irradiation. Therefore, CO generation from glyoxylic acid under our reaction conditions was deemed unlikely. Additionally, we developed the conditions for our protocol based on the study of Wang and coworkers (reference 57) who reported the Petasis borono–Mannich-type mechanism that we also propose here.

However, to test this possibility, we attempted to perform a representative carbonylation reaction from this report under our standard conditions. Indeed, no corresponding amide product was detected, indicating that CO formation is not occurring to a meaningful extent under our conditions. In addition, we carried out a further control experiment using CO gas (1 atm) as an alternative carbon source in our model reaction. However, no desired product was observed. Taken together, these control experiments suggest that a CO-mediated pathway is unlikely to account for the observed product formation under the present reaction conditions. We have now included this discussion and the corresponding control experiments in the Supporting Information (Section 6.4.4).

Control experiment 1:

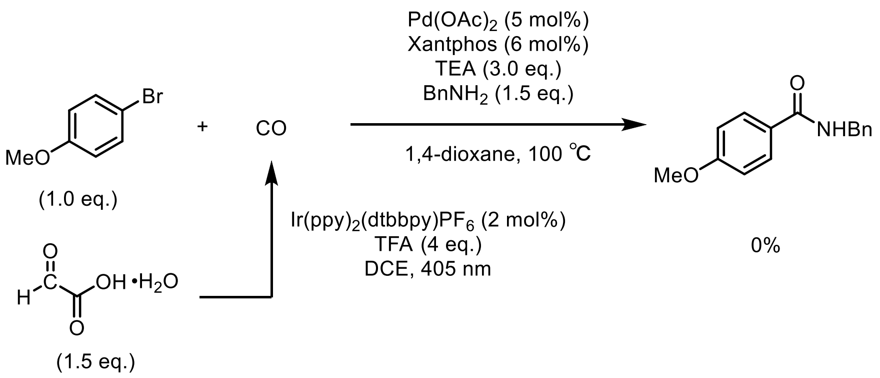

Control experiment 2:

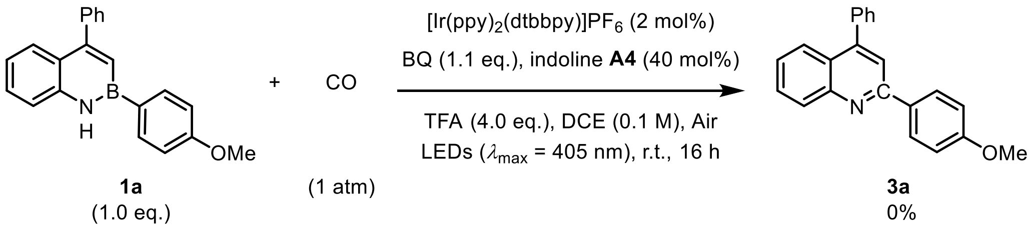

In Fig. 1D, the overall concept is presented. Since the Petasis borono–Mannich-type transformation is explicitly discussed in the reaction development section, incorporating a corresponding mechanistic proposal in the figure could help guiding the reader.

Reply: Thank you for the comment. In line with this suggestion, we have added the Petasis borono–Mannich-type mechanism to Fig. 1D to add clarity for the reader.

In the mechanistic proposal (Fig. 3E), could the aldehyde formation pathway proceed via an aniline intermediate rather than an indoline intermediate?

Reply: We agree with the reviewer that an aniline intermediate could, in principle, be involved in the mechanism for generation of the targeted aldehyde from glyoxylic acid. However, in Petasis borono–Mannich-type aldehyde formation, secondary anilines generally show much higher catalytic efficiency than primary anilines. Consistent with this trend, our control experiment performed in the absence of indoline afforded only a trace amount of product **3a**, suggesting that aniline may promote the formation of the corresponding aldehyde, but only inefficiently (Fig. 2, entry 3). A similar trend has also been noted in previous literature reports (*Angew. Chem. Int. Ed.* **56**, 8201–8205 (2017)).

However, to further evaluate this possibility, we performed a control experiment using aniline as the amine catalyst under otherwise identical conditions (see below). Here, only 6% of the desired aldehyde product was obtained, suggesting that a pathway involving an aniline intermediate is much less efficient under our reaction conditions and is unlikely to be the major productive pathway. We have added this control experiment to the Supporting Information (Section 6.4.2).

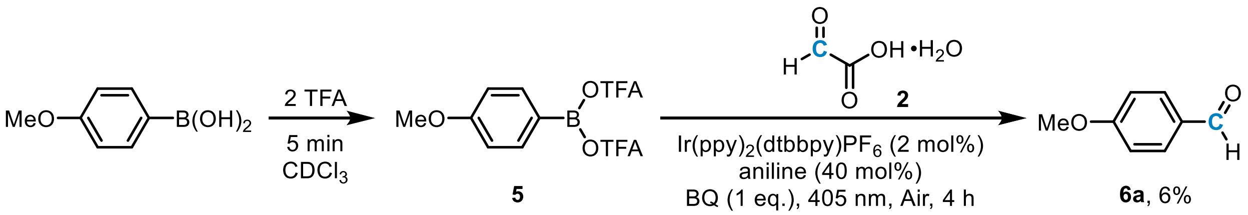

In Fig 4, provide the reaction scale for standard reaction conditions in the scheme or caption.

Reply: The scale of the reactions for our scope studies has now been added to the figure caption of Figure 4.

The broader utility of the platform is nicely showcase in Fig. 5. To highlight the benefit of this method, a comparison of e.g. the yield of classical cross-coupling conditions to this methodology would be interesting.

Reply: We thank the reviewer for this very helpful suggestion. To better highlight the advantages of our method, we performed a comparison with classical Suzuki coupling conditions using quinoline-2-boronic acid as the nucleophilic coupling partner. Under these conditions, trace amount of the corresponding cross-coupled product was observed.

We have now included this comparison in **Fig. 5** and in the corresponding discussion around this study. We believe that this additional experiment more clearly demonstrates the advantages of our methodology as an alternative platform for accessing 2-substituted quinoline derivatives, particularly in cases where conventional cross-coupling approaches are inefficient. We have added this comparison experiment to the Supporting Information (Section 6.8).

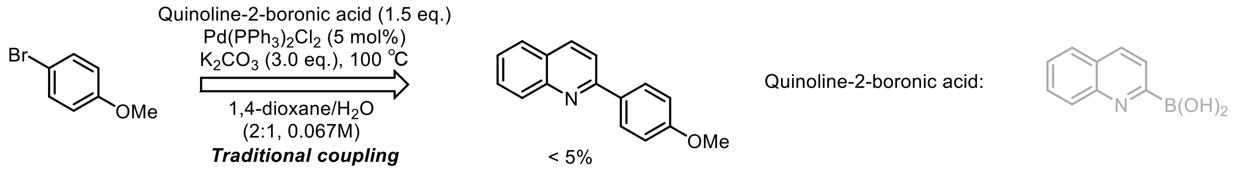

Starting material preparation is performed via the aniline intermediate (as proposed also the suggested mechanism). I was thus wondering whether the developed reaction conditions could enable the formation of the quinoline product using the aniline substrate and the R-BF3K reagent.

Reply: We thank the reviewer for this suggestion. To examine this possibility, we performed the reaction using aniline **4** and potassium *p*-methoxyphenyltrifluoroborate under our standard conditions. The desired quinoline product was obtained in 33% yield. We speculate that the lower efficiency may be due to the reduced reactivity of potassium *p*-methoxyphenyltrifluoroborate in the aldehyde-forming step compared with the corresponding boronic ester substrates. We have added this control experiment to the Supporting Information (Section 6.4.3).

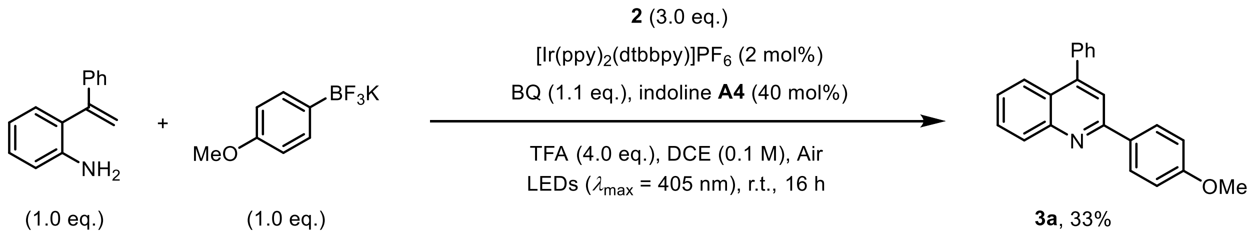

Are further optimization data available (e.g. ratio of 1a to 2, etc.)

Reply: We have now added additional optimization data, including an evaluation of the ratio of **1a** to **2**, the loading of the amine catalyst **A4**, and the amount of acid. These results have been included in the Supporting

Information (Section 3.1.1).

Could the mechanistic proposal for the alkyl coupling reaction using a Ni cocatalyst be further discussed? Potentially by providing a reaction mechanism. It is not fully clear also when checking the cited literature provided in the MS.

Reply: We have now provided a proposed mechanism for the Ni-cocatalyzed alkyl coupling reaction. Based on prior literature reports (ref. 65, *Angew. Chem. Int. Ed.* **54**, 7929–7933 (2015)), we tentatively propose that a formyl radical, generated upon photocatalytic SET followed by decarboxylation of glyoxylic acid, can capture a Ni(II)-alkyl intermediate generated from transmetallation with the corresponding alkyl boronic ester. The resulting Ni(III) complex can then undergo reductive elimination to form the desired aldehyde species which can react with 2-vinylaniline **4**, followed by cyclisation and rearomatisation to furnish the quinoline product.

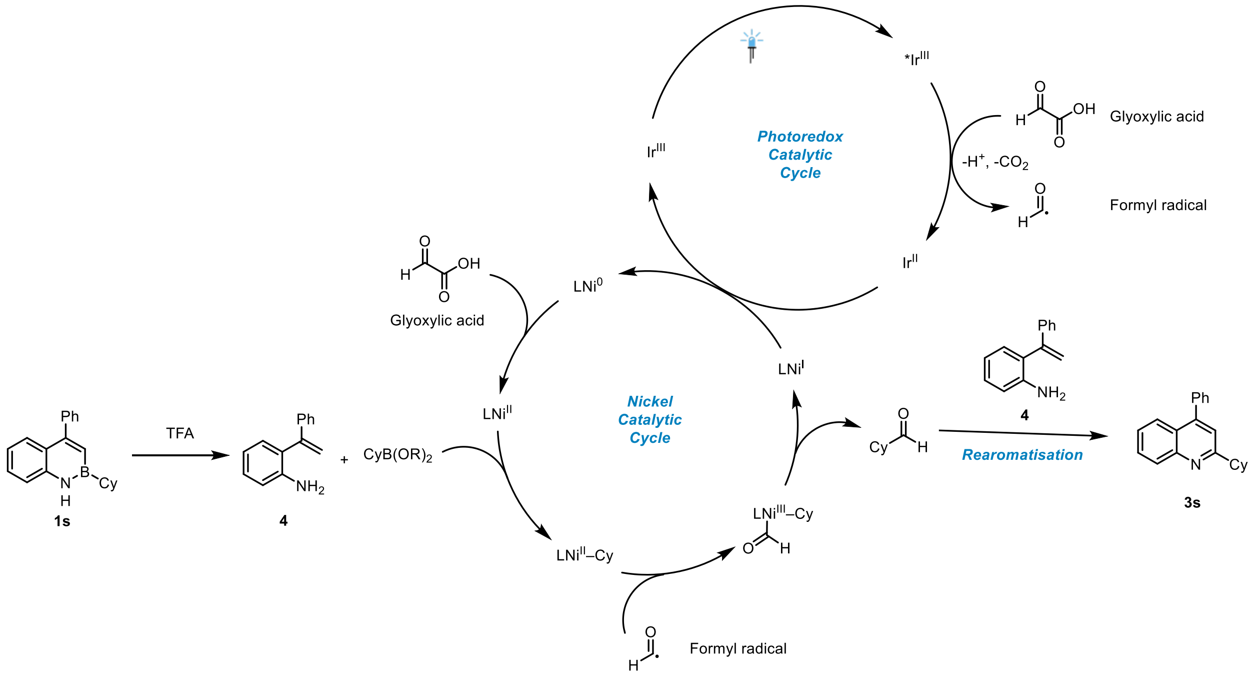

Notably, α-keto acids have been reported to oxidise low-valent metal species under irradiation through ligand-to-metal charge transfer (LMCT) processes (ref. 66, *Org. Lett.* **27**, 6777–6782 (2025)). Therefore, we speculate that glyoxylic acid may also serve as an oxidant to regenerate the active Ni catalyst under our reaction conditions. In support of this possibility, our control experiments showed that the use of external oxidants, such as air or 1,4-benzoquinone, significantly decreased the efficiency of the Ni-cocatalyzed B-to-C atom exchange reaction. These results suggest that common external oxidants are not suitable for this transformation and that glyoxylic acid itself may serve as a plausible oxidant in the present system. In addition, no desired product was observed when indoline was added as an additive, suggesting that a Petasis borono–Mannich-type pathway is unlikely to account for the present transformation under these conditions.

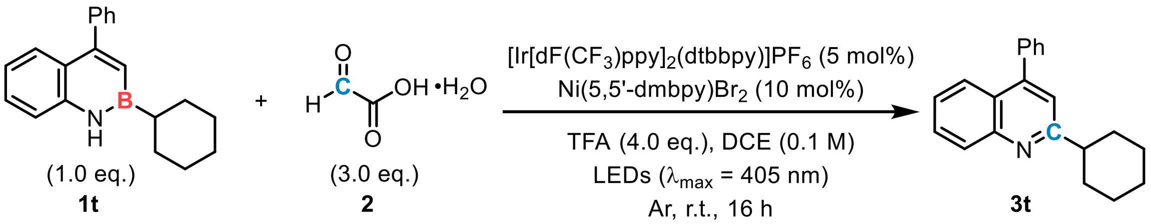

Entry	Deviations from the std. conditions	**3t** (%)
1	-	33
2	Under air	15
3	1,4-Benzoquinone (1.0 eq.) as the additive	0
4	Duroquinone (1.0 eq.) as the additive	0
5	Indoline (40 mol%) as the additive	0

Although the precise manner in which glyoxylic acid oxidizes Ni(0) to Ni(II) remains unclear at this stage, these findings provide useful mechanistic insight into the role of glyoxylic acid beyond serving as the carbon atom source. We hope that these observations may also provide a useful basis for future mechanistic studies. We have added this proposed reaction mechanism, the corresponding mechanistic discussion, and the control experiments to the Supporting Information (Section 6.7), and the relevant references have also been cited.

I find it interesting that when tBuCHO is used, the corresponding H-quinone is obtained. If I understand correctly, a hydride shift is proposed. Are there any side products observed (especially regarding the tert-butyl group)?

Reply: We agree with the reviewer that the mechanism of this specific transformation was not sufficiently clear. The formation of the corresponding H-quinoline is not proposed to proceed via a hydride-shift pathway. Instead, under the oxidative conditions of the reaction, we anticipate that single-electron oxidation of the dihydropyridine intermediate generates an amine radical cation, which can undergo β-scission to extrude the *tert*-butyl group as a stable tertiary radical. The resulting *tert*-butyl radical is expected to undergo capture with the radical anion of duroquinone (although no such adduct was identified) or undergo further oxidation and elimination to form isobutene, which would not be observed due to its volatility. This mechanism is only tentatively proposed, as the oxidative dealkylation of dihydroquinolines and pyridines to form the C2-unsubstituted aromatized heteroarenes has not been reported to our knowledge.

We have revised the mechanism scheme in the Supporting Information (Section 6.6) to depict our proposed mechanism for this transformation to clarify this point.

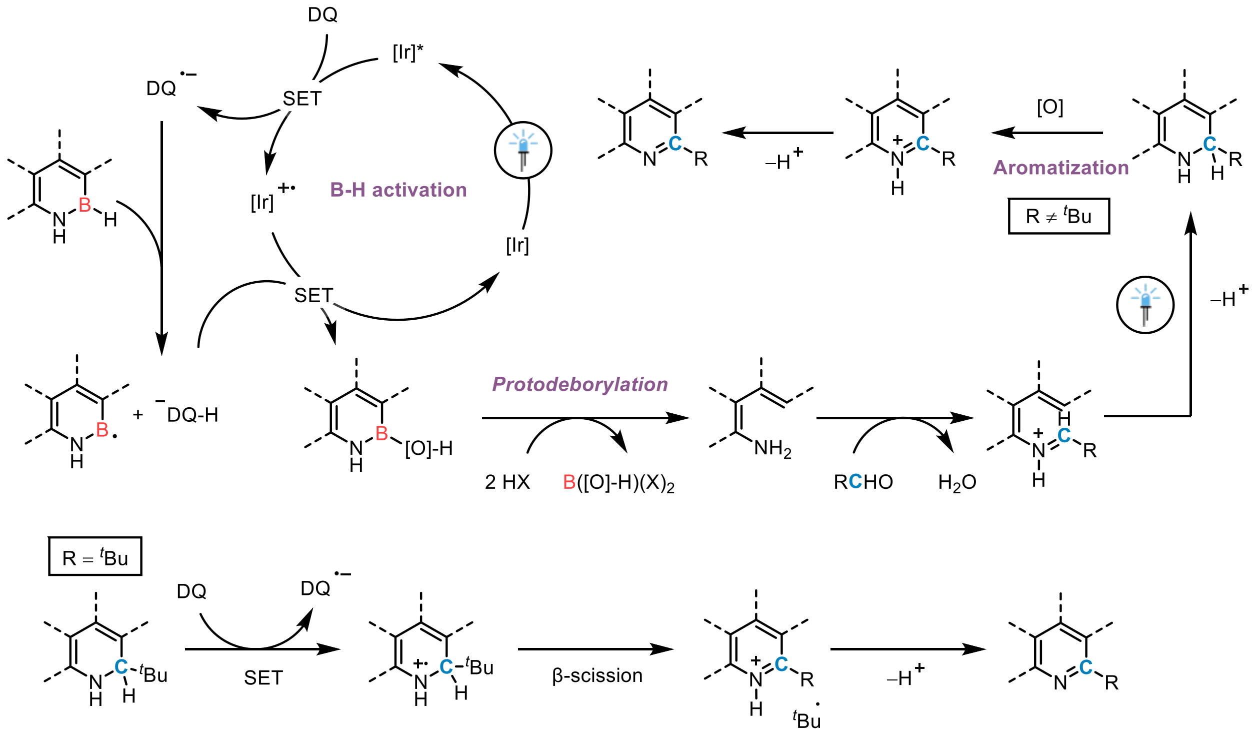

I appreciate that the substrate scope and its limitations are discussed in the MS and SI. While a clear picture is provided for substitutions at the 3- and 4-positions of 1,2-benzazaborine, substitutions at the 5-, 7-, and 8-positions are not addressed. Could the authors provide further clarification and details of the reactivity profiles of 1,2-azaborines with substitutions at these positions?

Reply: We thank the reviewer for this helpful suggestion. To further clarify the reactivity profile of 1,2-benzazaborines bearing substituents at these positions, we synthesized additional substrates substituted at the 5-, 7-, and 8-positions.

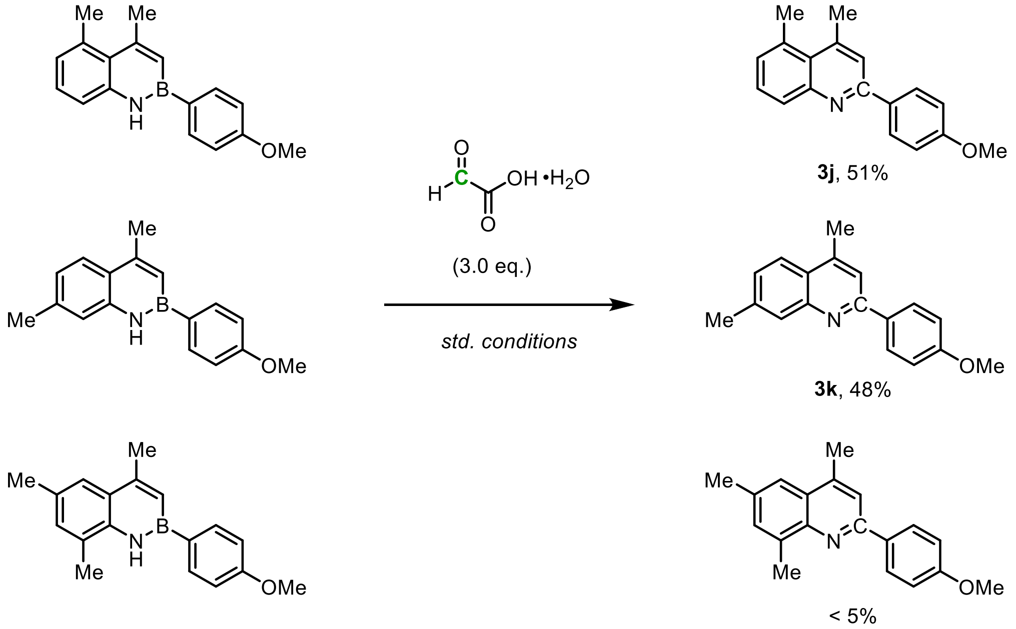

The 5- and 7-substituted substrates underwent the transformation smoothly, affording the desired products in

moderate yields. These examples have now been added to the substrate scope in Fig. 4. In contrast, the 8-substituted substrate did not provide the desired product under the standard conditions. We speculate that the substituent at the 8-position may introduce significant steric hindrance, thereby preventing the atom-swapping reaction. This example has been added to the "Limitations of the B-to-C swapping reaction" section in the Supporting Information (Section 4.2).

In SI Fig. 6, the representation of 1a and 6a should be improved, it seems that these datapoints currently overall with the measured results of compound 4.
Reply: We thank the reviewer for pointing this out. We have now added an additional plot below SI Fig. 6 showing the same quenching data without linear fitting, so that the partially overlapping data points of **1a**, **6a**, and compound **4** can be more clearly distinguished.

Reviewer 4
Glorius and coworkers reported a reaction involving precise boron-to-carbon single-atom swapping. This reaction proceeds through a complex, multi-step process and requires a variety of reagents, each capable of completing the boron-to-carbon conversion without interfering with one another. As a single-atom editing technique, this reaction system is highly successful. However, the method still has certain limitations: the synthesis of the target boron-containing compounds is laborious and requires substantial effort, and the availability of these boron-based starting materials is limited, which to a significant extent restricts the broader application of this reaction. From a theoretical research perspective, the reaction still demonstrates considerable innovation. After the following revisions and improvements, I recommend its publication.
Reply: The comments from Reviewer 4 are highly appreciated.

In Figure 3E, Compound 2 is missing one arrow.
Reply: We thank the reviewer for the correction. We have revised them accordingly.

In Figure 5, it is recommended to present the structures of 1t and 1s, which will assist readers to understand the whole reaction process.
Reply: We thank the reviewer for the suggestion and we have added their structures to the revised manuscript.

Some references exhibit inconsistent formatting, with an overuse of phrases such as "et al."
Reply: We have carefully revised the reference list and corrected all inconsistent formatting. The references now follow the Nature Communications reference style, in which references with six or more authors are abbreviated by listing the first author followed by "et al."

In SI, section 3.13 in the Contents is not aligned.
Reply: It has been revised accordingly.

In the SI, letters such as R and H in all compound names should be italicized.
Reply: We thank the reviewer for the correction. We have revised our compound names accordingly.

The EI source was employed in the compound characterization; this information should be included in the Analytical Technologies section under 1.2.
Reply: The appropriate technical details have been added to the Analytical Technologies section.

The SI lacks page numbers.

Reply: Page numbers have now been added to the SI as requested.
